# Supplementary material for: Immune gene expression networks in sepsis: A network biology approach
Source: PLoS One. 2021 Mar 5;16(3):e0247669. doi: 10.1371/journal.pone.0247669 (PMC7935325; doi:10.1371/journal.pone.0247669)
Supplement: S1 File — (DOCX) [file pone.0247669.s001.docx]

**SUPPLEMENTARY MATERIALS**

**Gene expression network in sepsis: A network biology approach**

Kyung Soo Kim^1^, Dong Wook Jekarl^2,4 *^, Jaeeun Yoo^3,4^, Seungok Lee^3,4^, Myungshin Kim^2,4^, Yonggoo Kim^2,4^

^1^Department of Thoracic and Cardiovascular Surgery**,** Seoul St. Mary’s Hospital, College of Medicine, The Catholic University of Korea, Seoul, Republic of Korea

^2^Department of Laboratory Medicine, Seoul St. Mary’s Hospital, College of Medicine, The Catholic University of Korea, Seoul, Republic of Korea

^3^Department of Laboratory Medicine**,** Incheon St. Mary’s Hospital, College of Medicine, The Catholic University of Korea, Seoul, Republic of Korea

^4^Laboratory for Development and Evaluation Center, Seoul St. Mary’s Hospital, College of Medicine, The Catholic University of Korea, Seoul, Republic of Korea

**SUPPLEMENTARY TABLES**

**Supplementary Table S1:** Recruited pathway from KEGG and assignment of the function

| KEGG pathway | Assignment |
| --- | --- |
| hematopoietic cell lineage (has04640) | Hematopoiesis (H) |
| complement and coagulation cascade (has04610) | Complement-Coagulation (C) |
| platelet activation (hsa04611) | Platelet (P) |
| Toll-like receptor signaling pathway (hsa04620) | Innate immunity (I) |
| Toll and Imd signalling pathway (hsa04624) | Innate immunity (I) |
| NOD-like receptor signaling pathway (hsa04621) | Innate immunity (I) |
| RIG-I-like receptor signaling (hsa04622) | Innate immunity (I) |
| Cytosolic DNA-sensing pathway (hsa04623) | Signaling (S) |
| C-type lectin receptor signaling pathway (hsa04625) | Innate immunity (I) |
| Natural killer cell mediated cytotoxicity (hsa04650) | NK cell (NK) |
| Antigen processing and presentation (hsa04612) | antigen presenting (APC) |
| T cell receptor signaling pathway (hsa04660) | Adaptive immunity (A) |
| Th1 and Th2 cell differentiation (04658) | Adaptive immunity (A) |
| Th17 cell differentiation (hsa04659) | Adaptive immunity (A) |
| B cell receptor signaling pathway (hsa04662) | Adaptive immunity (A) |
| Fc epsilon RI signaling pathway (hsa04664) | Adaptive immunity (A) |
| Fc gamma R-mediated phagocytosis (hsa04666) | Adaptive immunity (A) |
| Leukocyte transendothelial migration (hsa04670) | Leukocyte migration (LM) |
| Intestinal immune network for IgA production (hsa04672) | Adaptive immunity (A) |
| Chemokine signaling pathway (hsa04062) | Cytokine-Chemokine (K) |

**Supplementary Table S2:** Recruited genes from KEGG and the pathway

| GENE SYMBOL | PATHWAY |
| --- | --- |
| CD3D | T cell receptor SP |
| CD3E | T cell receptor SP |
| CD3G | T cell receptor SP |
| CD247 | T cell receptor SP |
| CD4 | T cell receptor SP |
| CD8A | T cell receptor SP |
| CD8B | T cell receptor SP |
| PTPRC | T cell receptor SP |
| LCK | T cell receptor SP |
| FYN | T cell receptor SP |
| ZAP70 | T cell receptor SP |
| LCP2 | T cell receptor SP |
| LAT | T cell receptor SP |
| ITK | T cell receptor SP |
| TEC | T cell receptor SP |
| NCK1 | T cell receptor SP |
| NCK2 | T cell receptor SP |
| VAV3 | T cell receptor SP |
| VAV1 | T cell receptor SP |
| VAV2 | T cell receptor SP |
| GRAP2 | T cell receptor SP |
| GRB2 | T cell receptor SP |
| PAK1 | T cell receptor SP |
| PAK2 | T cell receptor SP |
| PAK3 | T cell receptor SP |
| PAK4 | T cell receptor SP |
| PAK5 | T cell receptor SP |
| PAK6 | T cell receptor SP |
| BUB1B-PAK6 | T cell receptor SP |
| RHOA | T cell receptor SP |
| CDC42 | T cell receptor SP |
| DLG1 | T cell receptor SP |
| MAPK11 | T cell receptor SP |
| MAPK12 | T cell receptor SP |
| MAPK13 | T cell receptor SP |
| MAPK14 | T cell receptor SP |
| PLCG1 | T cell receptor SP |
| PPP3CA | T cell receptor SP |
| PPP3CB | T cell receptor SP |
| PPP3CC | T cell receptor SP |
| PPP3R1 | T cell receptor SP |
| PPP3R2 | T cell receptor SP |
| NFATC1 | T cell receptor SP |
| NFATC2 | T cell receptor SP |
| NFATC3 | T cell receptor SP |
| SOS1 | T cell receptor SP |
| SOS2 | T cell receptor SP |
| RASGRP1 | T cell receptor SP |
| HRAS | T cell receptor SP |
| KRAS | T cell receptor SP |
| NRAS | T cell receptor SP |
| RAF1 | T cell receptor SP |
| MAP2K1 | T cell receptor SP |
| MAP2K2 | T cell receptor SP |
| MAPK1 | T cell receptor SP |
| MAPK3 | T cell receptor SP |
| FOS | T cell receptor SP |
| JUN | T cell receptor SP |
| PRKCQ | T cell receptor SP |
| CARD11 | T cell receptor SP |
| BCL10 | T cell receptor SP |
| MALT1 | T cell receptor SP |
| MAP3K7 | T cell receptor SP |
| MAP2K7 | T cell receptor SP |
| CHUK | T cell receptor SP |
| IKBKB | T cell receptor SP |
| IKBKG | T cell receptor SP |
| NFKB1 | T cell receptor SP |
| RELA | T cell receptor SP |
| NFKBIA | T cell receptor SP |
| NFKBIB | T cell receptor SP |
| NFKBIE | T cell receptor SP |
| CD28 | T cell receptor SP |
| ICOS | T cell receptor SP |
| CD40LG | T cell receptor SP |
| PIK3R1 | T cell receptor SP |
| PIK3R2 | T cell receptor SP |
| PIK3R3 | T cell receptor SP |
| PIK3CA | T cell receptor SP |
| PIK3CD | T cell receptor SP |
| PIK3CB | T cell receptor SP |
| PDPK1 | T cell receptor SP |
| AKT1 | T cell receptor SP |
| AKT2 | T cell receptor SP |
| AKT3 | T cell receptor SP |
| MAP3K8 | T cell receptor SP |
| MAP3K14 | T cell receptor SP |
| GSK3B | T cell receptor SP |
| PDCD1 | T cell receptor SP |
| CTLA4 | T cell receptor SP |
| PTPN6 | T cell receptor SP |
| CBLB | T cell receptor SP |
| IL2 | T cell receptor SP |
| IL4 | T cell receptor SP |
| IL5 | T cell receptor SP |
| IL10 | T cell receptor SP |
| IFNG | antigen processing and presentation |
| CSF2 | antigen processing and presentation |
| TNF | antigen processing and presentation |
| CDK4 | antigen processing and presentation |
| MAPK9 | antigen processing and presentation |
| IFNG | antigen processing and presentation |
| TNF | antigen processing and presentation |
| PSME1 | antigen processing and presentation |
| PSME2 | antigen processing and presentation |
| PSME3 | antigen processing and presentation |
| HSPA8 | antigen processing and presentation |
| HSPA1A | antigen processing and presentation |
| HSPA2 | antigen processing and presentation |
| HSPA1L | antigen processing and presentation |
| HSPA1B | antigen processing and presentation |
| HSPA6 | antigen processing and presentation |
| HSPA4 | antigen processing and presentation |
| HSP90AA1 | antigen processing and presentation |
| HSP90AB1 | antigen processing and presentation |
| HLA-A | NK cell mediated cytotoxicity |
| HLA-B | NK cell mediated cytotoxicity |
| HLA-C | NK cell mediated cytotoxicity |
| HLA-F | NK cell mediated cytotoxicity |
| HLA-G | NK cell mediated cytotoxicity |
| HLA-E | NK cell mediated cytotoxicity |
| CANX | NK cell mediated cytotoxicity |
| B2M | NK cell mediated cytotoxicity |
| PDIA3 | NK cell mediated cytotoxicity |
| CALR | NK cell mediated cytotoxicity |
| TAPBP | NK cell mediated cytotoxicity |
| TAP1 | NK cell mediated cytotoxicity |
| TAP2 | NK cell mediated cytotoxicity |
| CD8A | NK cell mediated cytotoxicity |
| CD8B | NK cell mediated cytotoxicity |
| KIR3DL2 | NK cell mediated cytotoxicity |
| KIR3DL1 | NK cell mediated cytotoxicity |
| KIR3DL3 | NK cell mediated cytotoxicity |
| KIR2DL2 | NK cell mediated cytotoxicity |
| KIR2DL1 | NK cell mediated cytotoxicity |
| KIR2DL3 | NK cell mediated cytotoxicity |
| KIR2DL4 | NK cell mediated cytotoxicity |
| KIR2DL5A | NK cell mediated cytotoxicity |
| KLRC1 | NK cell mediated cytotoxicity |
| KLRC2 | NK cell mediated cytotoxicity |
| KLRC3 | NK cell mediated cytotoxicity |
| KLRC4 | NK cell mediated cytotoxicity |
| KLRD1 | NK cell mediated cytotoxicity |
| KIR2DS1 | NK cell mediated cytotoxicity |
| KIR2DS3 | NK cell mediated cytotoxicity |
| KIR2DS4 | NK cell mediated cytotoxicity |
| KIR2DS5 | NK cell mediated cytotoxicity |
| KIR2DS2 | NK cell mediated cytotoxicity |
| IFI30 | NK cell mediated cytotoxicity |
| LGMN | NK cell mediated cytotoxicity |
| CTSB | NK cell mediated cytotoxicity |
| HLA-DMA | NK cell mediated cytotoxicity |
| HLA-DMB | NK cell mediated cytotoxicity |
| HLA-DOA | NK cell mediated cytotoxicity |
| HLA-DOB | NK cell mediated cytotoxicity |
| HLA-DPA1 | NK cell mediated cytotoxicity |
| HLA-DPB1 | NK cell mediated cytotoxicity |
| HLA-DQA1 | NK cell mediated cytotoxicity |
| HLA-DQA2 | NK cell mediated cytotoxicity |
| HLA-DQB1 | NK cell mediated cytotoxicity |
| HLA-DRA | NK cell mediated cytotoxicity |
| HLA-DRB1 | NK cell mediated cytotoxicity |
| HLA-DRB3 | NK cell mediated cytotoxicity |
| HLA-DRB4 | NK cell mediated cytotoxicity |
| HLA-DRB5 | NK cell mediated cytotoxicity |
| CD74 | NK cell mediated cytotoxicity |
| CTSL | NK cell mediated cytotoxicity |
| CTSS | NK cell mediated cytotoxicity |
| CD4 | NK cell mediated cytotoxicity |
| CIITA | NK cell mediated cytotoxicity |
| RFX5 | NK cell mediated cytotoxicity |
| RFXANK | NK cell mediated cytotoxicity |
| RFXAP | NK cell mediated cytotoxicity |
| CREB1 | NK cell mediated cytotoxicity |
| NFYA | NK cell mediated cytotoxicity |
| NFYB | NK cell mediated cytotoxicity |
| NFYC | NK cell mediated cytotoxicity |
| HSPA5 | NK cell mediated cytotoxicity |
| HLA-A | NK cell mediated cytotoxicity |
| HLA-B | NK cell mediated cytotoxicity |
| HLA-C | NK cell mediated cytotoxicity |
| HLA-G | NK cell mediated cytotoxicity |
| HLA-E | NK cell mediated cytotoxicity |
| KIR3DL2 | NK cell mediated cytotoxicity |
| KIR3DL1 | NK cell mediated cytotoxicity |
| KIR2DL2 | NK cell mediated cytotoxicity |
| KIR2DL1 | NK cell mediated cytotoxicity |
| KIR2DL3 | NK cell mediated cytotoxicity |
| KIR2DL4 | NK cell mediated cytotoxicity |
| KIR2DL5A | NK cell mediated cytotoxicity |
| KLRC1 | NK cell mediated cytotoxicity |
| KLRC2 | NK cell mediated cytotoxicity |
| KLRC3 | NK cell mediated cytotoxicity |
| KLRD1 | NK cell mediated cytotoxicity |
| PTPN6 | NK cell mediated cytotoxicity |
| PTPN11 | NK cell mediated cytotoxicity |
| ICAM1 | NK cell mediated cytotoxicity |
| ICAM2 | NK cell mediated cytotoxicity |
| ITGAL | NK cell mediated cytotoxicity |
| ITGB2 | NK cell mediated cytotoxicity |
| PTK2B | NK cell mediated cytotoxicity |
| VAV3 | NK cell mediated cytotoxicity |
| VAV1 | NK cell mediated cytotoxicity |
| VAV2 | NK cell mediated cytotoxicity |
| RAC1 | NK cell mediated cytotoxicity |
| RAC2 | NK cell mediated cytotoxicity |
| RAC3 | NK cell mediated cytotoxicity |
| PAK1 | NK cell mediated cytotoxicity |
| MAP2K1 | NK cell mediated cytotoxicity |
| MAP2K2 | NK cell mediated cytotoxicity |
| MAPK1 | NK cell mediated cytotoxicity |
| MAPK3 | NK cell mediated cytotoxicity |
| TNF | NK cell mediated cytotoxicity |
| CSF2 | NK cell mediated cytotoxicity |
| IFNG | NK cell mediated cytotoxicity |
| KIR2DS1 | NK cell mediated cytotoxicity |
| KIR2DS3 | NK cell mediated cytotoxicity |
| KIR2DS4 | NK cell mediated cytotoxicity |
| KIR2DS5 | NK cell mediated cytotoxicity |
| KIR2DS2 | NK cell mediated cytotoxicity |
| NCR2 | NK cell mediated cytotoxicity |
| TYROBP | NK cell mediated cytotoxicity |
| LCK | NK cell mediated cytotoxicity |
| IGH | NK cell mediated cytotoxicity |
| FCGR3A | NK cell mediated cytotoxicity |
| FCGR3B | NK cell mediated cytotoxicity |
| NCR1 | NK cell mediated cytotoxicity |
| NCR3 | NK cell mediated cytotoxicity |
| FCER1G | NK cell mediated cytotoxicity |
| CD247 | NK cell mediated cytotoxicity |
| ZAP70 | NK cell mediated cytotoxicity |
| SYK | NK cell mediated cytotoxicity |
| LCP2 | NK cell mediated cytotoxicity |
| LAT | NK cell mediated cytotoxicity |
| PLCG1 | NK cell mediated cytotoxicity |
| PLCG2 | NK cell mediated cytotoxicity |
| SH3BP2 | NK cell mediated cytotoxicity |
| PIK3CA | NK cell mediated cytotoxicity |
| PIK3CD | NK cell mediated cytotoxicity |
| PIK3CB | NK cell mediated cytotoxicity |
| PIK3R1 | NK cell mediated cytotoxicity |
| PIK3R2 | NK cell mediated cytotoxicity |
| PIK3R3 | NK cell mediated cytotoxicity |
| FYN | NK cell mediated cytotoxicity |
| SHC1 | NK cell mediated cytotoxicity |
| SHC2 | NK cell mediated cytotoxicity |
| SHC3 | NK cell mediated cytotoxicity |
| SHC4 | NK cell mediated cytotoxicity |
| GRB2 | NK cell mediated cytotoxicity |
| SOS1 | NK cell mediated cytotoxicity |
| SOS2 | NK cell mediated cytotoxicity |
| HRAS | NK cell mediated cytotoxicity |
| KRAS | NK cell mediated cytotoxicity |
| NRAS | NK cell mediated cytotoxicity |
| ARAF | NK cell mediated cytotoxicity |
| BRAF | NK cell mediated cytotoxicity |
| RAF1 | NK cell mediated cytotoxicity |
| MICB | NK cell mediated cytotoxicity |
| MICA | NK cell mediated cytotoxicity |
| ULBP1 | NK cell mediated cytotoxicity |
| ULBP2 | NK cell mediated cytotoxicity |
| ULBP3 | NK cell mediated cytotoxicity |
| RAET1G | NK cell mediated cytotoxicity |
| RAET1L | NK cell mediated cytotoxicity |
| RAET1E | NK cell mediated cytotoxicity |
| KLRK1 | NK cell mediated cytotoxicity |
| KLRC4-KLRK1 | NK cell mediated cytotoxicity |
| HCST | NK cell mediated cytotoxicity |
| CD48 | NK cell mediated cytotoxicity |
| CD244 | NK cell mediated cytotoxicity |
| PPP3CA | NK cell mediated cytotoxicity |
| PPP3CB | NK cell mediated cytotoxicity |
| PPP3CC | NK cell mediated cytotoxicity |
| PPP3R1 | NK cell mediated cytotoxicity |
| PPP3R2 | NK cell mediated cytotoxicity |
| NFATC1 | NK cell mediated cytotoxicity |
| NFATC2 | NK cell mediated cytotoxicity |
| PRKCA | NK cell mediated cytotoxicity |
| PRKCB | NK cell mediated cytotoxicity |
| PRKCG | NK cell mediated cytotoxicity |
| SH2D1B | NK cell mediated cytotoxicity |
| SH2D1A | NK cell mediated cytotoxicity |
| IFNGR1 | NK cell mediated cytotoxicity |
| IFNGR2 | NK cell mediated cytotoxicity |
| IFNA1 | NK cell mediated cytotoxicity |
| IFNA2 | NK cell mediated cytotoxicity |
| IFNA4 | NK cell mediated cytotoxicity |
| IFNA5 | NK cell mediated cytotoxicity |
| IFNA6 | NK cell mediated cytotoxicity |
| IFNA7 | NK cell mediated cytotoxicity |
| IFNA8 | NK cell mediated cytotoxicity |
| IFNA10 | NK cell mediated cytotoxicity |
| IFNA13 | NK cell mediated cytotoxicity |
| IFNA14 | NK cell mediated cytotoxicity |
| IFNA16 | NK cell mediated cytotoxicity |
| IFNA17 | NK cell mediated cytotoxicity |
| IFNA21 | NK cell mediated cytotoxicity |
| IFNB1 | NK cell mediated cytotoxicity |
| IFNAR1 | NK cell mediated cytotoxicity |
| IFNAR2 | NK cell mediated cytotoxicity |
| TNFSF10 | NK cell mediated cytotoxicity |
| TNFRSF10A | NK cell mediated cytotoxicity |
| TNFRSF10B | NK cell mediated cytotoxicity |
| FASLG | NK cell mediated cytotoxicity |
| FAS | NK cell mediated cytotoxicity |
| GZMB | NK cell mediated cytotoxicity |
| PRF1 | NK cell mediated cytotoxicity |
| CASP3 | NK cell mediated cytotoxicity |
| BID | NK cell mediated cytotoxicity |
| DLL3 | Th1 and Th2 differentiation |
| DLL1 | Th1 and Th2 differentiation |
| DLL4 | Th1 and Th2 differentiation |
| NOTCH3 | Th1 and Th2 differentiation |
| MAML3 | Th1 and Th2 differentiation |
| MAML2 | Th1 and Th2 differentiation |
| MAML1 | Th1 and Th2 differentiation |
| RBPJL | Th1 and Th2 differentiation |
| RBPJ | Th1 and Th2 differentiation |
| NFKB1 | Th1 and Th2 differentiation |
| RELA | Th1 and Th2 differentiation |
| IFNG | Th1 and Th2 differentiation |
| IFNGR1 | Th1 and Th2 differentiation |
| IFNGR2 | Th1 and Th2 differentiation |
| JAK1 | Th1 and Th2 differentiation |
| JAK2 | Th1 and Th2 differentiation |
| STAT1 | Th1 and Th2 differentiation |
| IL12A | Th1 and Th2 differentiation |
| IL12B | Th1 and Th2 differentiation |
| IL12RB1 | Th1 and Th2 differentiation |
| IL12RB2 | Th1 and Th2 differentiation |
| TYK2 | Th1 and Th2 differentiation |
| STAT4 | Th1 and Th2 differentiation |
| TBX21 | Th1 and Th2 differentiation |
| RUNX3 | Th1 and Th2 differentiation |
| HLA-DMA | Th1 and Th2 differentiation |
| HLA-DMB | Th1 and Th2 differentiation |
| HLA-DOA | Th1 and Th2 differentiation |
| HLA-DOB | Th1 and Th2 differentiation |
| HLA-DPA1 | Th1 and Th2 differentiation |
| HLA-DPB1 | Th1 and Th2 differentiation |
| HLA-DQA1 | Th1 and Th2 differentiation |
| HLA-DQA2 | Th1 and Th2 differentiation |
| HLA-DQB1 | Th1 and Th2 differentiation |
| HLA-DRA | Th1 and Th2 differentiation |
| HLA-DRB1 | Th1 and Th2 differentiation |
| HLA-DRB3 | Th1 and Th2 differentiation |
| HLA-DRB4 | Th1 and Th2 differentiation |
| HLA-DRB5 | Th1 and Th2 differentiation |
| CD4 | Th1 and Th2 differentiation |
| LCK | Th1 and Th2 differentiation |
| CD3E | Th1 and Th2 differentiation |
| CD3G | Th1 and Th2 differentiation |
| CD247 | Th1 and Th2 differentiation |
| CD3D | Th1 and Th2 differentiation |
| ZAP70 | Th1 and Th2 differentiation |
| LAT | Th1 and Th2 differentiation |
| PLCG1 | Th1 and Th2 differentiation |
| PPP3CA | Th1 and Th2 differentiation |
| PPP3CB | Th1 and Th2 differentiation |
| PPP3CC | Th1 and Th2 differentiation |
| PPP3R1 | Th1 and Th2 differentiation |
| PPP3R2 | Th1 and Th2 differentiation |
| NFATC1 | Th1 and Th2 differentiation |
| NFATC2 | Th1 and Th2 differentiation |
| NFATC3 | Th1 and Th2 differentiation |
| PRKCQ | Th1 and Th2 differentiation |
| CHUK | Th1 and Th2 differentiation |
| IKBKB | Th1 and Th2 differentiation |
| IKBKG | Th1 and Th2 differentiation |
| NFKBIA | Th1 and Th2 differentiation |
| NFKBIB | Th1 and Th2 differentiation |
| NFKBIE | Th1 and Th2 differentiation |
| MAPK1 | Th1 and Th2 differentiation |
| MAPK3 | Th1 and Th2 differentiation |
| FOS | Th1 and Th2 differentiation |
| MAPK11 | Th1 and Th2 differentiation |
| MAPK12 | Th1 and Th2 differentiation |
| MAPK13 | Th1 and Th2 differentiation |
| MAPK14 | Th1 and Th2 differentiation |
| MAPK8 | Th1 and Th2 differentiation |
| MAPK10 | Th1 and Th2 differentiation |
| MAPK9 | Th1 and Th2 differentiation |
| JUN | Th1 and Th2 differentiation |
| IL2 | Th1 and Th2 differentiation |
| IL2RA | Th1 and Th2 differentiation |
| IL2RB | Th1 and Th2 differentiation |
| IL2RG | Th1 and Th2 differentiation |
| JAK3 | Th1 and Th2 differentiation |
| STAT5A | Th1 and Th2 differentiation |
| STAT5B | Th1 and Th2 differentiation |
| IL4 | Th1 and Th2 differentiation |
| IL4R | Th1 and Th2 differentiation |
| STAT6 | Th1 and Th2 differentiation |
| GATA3 | Th1 and Th2 differentiation |
| IL5 | Th1 and Th2 differentiation |
| IL13 | Th1 and Th2 differentiation |
| MAF | Th1 and Th2 differentiation |
| JAG1 | Th1 and Th2 differentiation |
| JAG2 | Th1 and Th2 differentiation |
| NOTCH1 | Th1 and Th2 differentiation |
| NOTCH2 | Th1 and Th2 differentiation |
| IL1B | Th17 differentiation |
| IL1R1 | Th17 differentiation |
| IL1RAP | Th17 differentiation |
| MAPK11 | Th17 differentiation |
| MAPK12 | Th17 differentiation |
| MAPK13 | Th17 differentiation |
| MAPK14 | Th17 differentiation |
| MTOR | Th17 differentiation |
| IRF4 | Th17 differentiation |
| TGFB1 | Th17 differentiation |
| TGFBR1 | Th17 differentiation |
| TGFBR2 | Th17 differentiation |
| SMAD2 | Th17 differentiation |
| SMAD3 | Th17 differentiation |
| SMAD4 | Th17 differentiation |
| IL21 | Th17 differentiation |
| IL21R | Th17 differentiation |
| IL2RG | Th17 differentiation |
| JAK1 | Th17 differentiation |
| JAK3 | Th17 differentiation |
| IL6 | Th17 differentiation |
| IL6R | Th17 differentiation |
| IL6ST | Th17 differentiation |
| JAK2 | Th17 differentiation |
| IL23A | Th17 differentiation |
| IL23R | Th17 differentiation |
| IL12RB1 | Th17 differentiation |
| TYK2 | Th17 differentiation |
| STAT3 | Th17 differentiation |
| RORC | Th17 differentiation |
| RORA | Th17 differentiation |
| HIF1A | Th17 differentiation |
| HSP90AA1 | Th17 differentiation |
| HSP90AB1 | Th17 differentiation |
| AHR | Th17 differentiation |
| IL17A | Th17 differentiation |
| IL17F | Th17 differentiation |
| IL22 | Th17 differentiation |
| HLA-DMA | Th17 differentiation |
| HLA-DMB | Th17 differentiation |
| HLA-DOA | Th17 differentiation |
| HLA-DOB | Th17 differentiation |
| HLA-DPA1 | Th17 differentiation |
| HLA-DPB1 | Th17 differentiation |
| HLA-DQA1 | Th17 differentiation |
| HLA-DQA2 | Th17 differentiation |
| HLA-DQB1 | Th17 differentiation |
| HLA-DRA | Th17 differentiation |
| HLA-DRB1 | Th17 differentiation |
| HLA-DRB3 | Th17 differentiation |
| HLA-DRB4 | Th17 differentiation |
| HLA-DRB5 | Th17 differentiation |
| CD4 | Th17 differentiation |
| LCK | Th17 differentiation |
| CD3E | Th17 differentiation |
| CD3G | Th17 differentiation |
| CD247 | Th17 differentiation |
| CD3D | Th17 differentiation |
| ZAP70 | Th17 differentiation |
| LAT | Th17 differentiation |
| PLCG1 | Th17 differentiation |
| PPP3CA | Th17 differentiation |
| PPP3CB | Th17 differentiation |
| PPP3CC | Th17 differentiation |
| PPP3R1 | Th17 differentiation |
| PPP3R2 | Th17 differentiation |
| NFATC1 | Th17 differentiation |
| NFATC2 | Th17 differentiation |
| NFATC3 | Th17 differentiation |
| PRKCQ | Th17 differentiation |
| CHUK | Th17 differentiation |
| IKBKB | Th17 differentiation |
| IKBKG | Th17 differentiation |
| NFKBIA | Th17 differentiation |
| NFKBIB | Th17 differentiation |
| NFKBIE | Th17 differentiation |
| NFKB1 | Th17 differentiation |
| RELA | Th17 differentiation |
| MAPK1 | Th17 differentiation |
| MAPK3 | Th17 differentiation |
| FOS | Th17 differentiation |
| MAPK8 | Th17 differentiation |
| MAPK10 | Th17 differentiation |
| MAPK9 | Th17 differentiation |
| JUN | Th17 differentiation |
| IL4 | Th17 differentiation |
| IL4R | Th17 differentiation |
| STAT6 | Th17 differentiation |
| GATA3 | Th17 differentiation |
| RUNX1 | Th17 differentiation |
| IL17D | Th17 differentiation |
| IL27RA | Th17 differentiation |
| IFNG | Th17 differentiation |
| IFNGR1 | Th17 differentiation |
| IFNGR2 | Th17 differentiation |
| STAT1 | Th17 differentiation |
| TBX21 | Th17 differentiation |
| IL2 | Th17 differentiation |
| IL2RA | Th17 differentiation |
| IL2RB | Th17 differentiation |
| STAT5A | Th17 differentiation |
| STAT5B | Th17 differentiation |
| FOXP3 | Th17 differentiation |
| RARA | Th17 differentiation |
| RXRA | Th17 differentiation |
| RXRB | Th17 differentiation |
| RXRG | Th17 differentiation |
| IGH | Bcell receptor SP |
| CD79A | Bcell receptor SP |
| CD79B | Bcell receptor SP |
| LYN | Bcell receptor SP |
| SYK | Bcell receptor SP |
| BTK | Bcell receptor SP |
| DAPP1 | Bcell receptor SP |
| BLNK | Bcell receptor SP |
| VAV3 | Bcell receptor SP |
| VAV1 | Bcell receptor SP |
| VAV2 | Bcell receptor SP |
| RAC1 | Bcell receptor SP |
| RAC2 | Bcell receptor SP |
| RAC3 | Bcell receptor SP |
| PLCG2 | Bcell receptor SP |
| PPP3CA | Bcell receptor SP |
| PPP3CB | Bcell receptor SP |
| PPP3CC | Bcell receptor SP |
| PPP3R1 | Bcell receptor SP |
| PPP3R2 | Bcell receptor SP |
| NFATC1 | Bcell receptor SP |
| NFATC2 | Bcell receptor SP |
| NFATC3 | Bcell receptor SP |
| GRB2 | Bcell receptor SP |
| SOS1 | Bcell receptor SP |
| SOS2 | Bcell receptor SP |
| RASGRP3 | Bcell receptor SP |
| HRAS | Bcell receptor SP |
| KRAS | Bcell receptor SP |
| NRAS | Bcell receptor SP |
| RAF1 | Bcell receptor SP |
| MAP2K1 | Bcell receptor SP |
| MAP2K2 | Bcell receptor SP |
| MAPK1 | Bcell receptor SP |
| MAPK3 | Bcell receptor SP |
| FOS | Bcell receptor SP |
| JUN | Bcell receptor SP |
| PRKCB | Bcell receptor SP |
| CARD11 | Bcell receptor SP |
| BCL10 | Bcell receptor SP |
| MALT1 | Bcell receptor SP |
| CHUK | Bcell receptor SP |
| IKBKB | Bcell receptor SP |
| IKBKG | Bcell receptor SP |
| NFKB1 | Bcell receptor SP |
| RELA | Bcell receptor SP |
| NFKBIA | Bcell receptor SP |
| NFKBIB | Bcell receptor SP |
| NFKBIE | Bcell receptor SP |
| IFITM1 | Bcell receptor SP |
| CD81 | Bcell receptor SP |
| CD19 | Bcell receptor SP |
| CR2 | Bcell receptor SP |
| PIK3R1 | Bcell receptor SP |
| PIK3R2 | Bcell receptor SP |
| PIK3R3 | Bcell receptor SP |
| PIK3CA | Bcell receptor SP |
| PIK3CD | Bcell receptor SP |
| PIK3CB | Bcell receptor SP |
| AKT1 | Bcell receptor SP |
| AKT2 | Bcell receptor SP |
| AKT3 | Bcell receptor SP |
| GSK3B | Bcell receptor SP |
| FCGR2B | Bcell receptor SP |
| INPP5D | Bcell receptor SP |
| INPPL1 | Bcell receptor SP |
| CD22 | Bcell receptor SP |
| CD72 | Bcell receptor SP |
| PTPN6 | Bcell receptor SP |
| PIK3AP1 | Bcell receptor SP |
| LILRB3 | Bcell receptor SP |
| IGH | Fc epsilon RI SP |
| FCER1A | Fc epsilon RI SP |
| MS4A2 | Fc epsilon RI SP |
| FCER1G | Fc epsilon RI SP |
| SYK | Fc epsilon RI SP |
| LYN | Fc epsilon RI SP |
| BTK | Fc epsilon RI SP |
| INPP5D | Fc epsilon RI SP |
| PLCG1 | Fc epsilon RI SP |
| PLCG2 | Fc epsilon RI SP |
| PRKCA | Fc epsilon RI SP |
| PIK3CA | Fc epsilon RI SP |
| PIK3CD | Fc epsilon RI SP |
| PIK3CB | Fc epsilon RI SP |
| PIK3R1 | Fc epsilon RI SP |
| PIK3R2 | Fc epsilon RI SP |
| PIK3R3 | Fc epsilon RI SP |
| PDPK1 | Fc epsilon RI SP |
| AKT1 | Fc epsilon RI SP |
| AKT2 | Fc epsilon RI SP |
| AKT3 | Fc epsilon RI SP |
| RAC1 | Fc epsilon RI SP |
| RAC2 | Fc epsilon RI SP |
| RAC3 | Fc epsilon RI SP |
| MAP2K4 | Fc epsilon RI SP |
| MAP2K7 | Fc epsilon RI SP |
| MAP2K3 | Fc epsilon RI SP |
| MAP2K6 | Fc epsilon RI SP |
| MAPK8 | Fc epsilon RI SP |
| MAPK10 | Fc epsilon RI SP |
| MAPK9 | Fc epsilon RI SP |
| MAPK11 | Fc epsilon RI SP |
| MAPK12 | Fc epsilon RI SP |
| MAPK13 | Fc epsilon RI SP |
| MAPK14 | Fc epsilon RI SP |
| IL4 | Fc epsilon RI SP |
| IL13 | Fc epsilon RI SP |
| IL3 | Fc epsilon RI SP |
| IL5 | Fc epsilon RI SP |
| CSF2 | Fc epsilon RI SP |
| TNF | Fc epsilon RI SP |
| LCP2 | Fc epsilon RI SP |
| VAV3 | Fc epsilon RI SP |
| VAV1 | Fc epsilon RI SP |
| VAV2 | Fc epsilon RI SP |
| FYN | Fc epsilon RI SP |
| GAB2 | Fc epsilon RI SP |
| LAT | Fc epsilon RI SP |
| GRB2 | Fc epsilon RI SP |
| SOS1 | Fc epsilon RI SP |
| SOS2 | Fc epsilon RI SP |
| HRAS | Fc epsilon RI SP |
| KRAS | Fc epsilon RI SP |
| NRAS | Fc epsilon RI SP |
| RAF1 | Fc epsilon RI SP |
| MAP2K1 | Fc epsilon RI SP |
| MAP2K2 | Fc epsilon RI SP |
| MAPK1 | Fc epsilon RI SP |
| MAPK3 | Fc epsilon RI SP |
| PLA2G4E | Fc epsilon RI SP |
| PLA2G4A | Fc epsilon RI SP |
| JMJD7-PLA2G4B | Fc epsilon RI SP |
| PLA2G4B | Fc epsilon RI SP |
| PLA2G4C | Fc epsilon RI SP |
| PLA2G4D | Fc epsilon RI SP |
| PLA2G4F | Fc epsilon RI SP |
| ALOX5 | Fc epsilon RI SP |
| ALOX5AP | Fc epsilon RI SP |
| IGH | FC gamma R mediated phagocytosis |
| FCGR1A | FC gamma R mediated phagocytosis |
| FCGR2A | FC gamma R mediated phagocytosis |
| PTPRC | FC gamma R mediated phagocytosis |
| HCK | FC gamma R mediated phagocytosis |
| LYN | FC gamma R mediated phagocytosis |
| SYK | FC gamma R mediated phagocytosis |
| PIK3CA | FC gamma R mediated phagocytosis |
| PIK3CD | FC gamma R mediated phagocytosis |
| PIK3CB | FC gamma R mediated phagocytosis |
| PIK3R1 | FC gamma R mediated phagocytosis |
| PIK3R2 | FC gamma R mediated phagocytosis |
| PIK3R3 | FC gamma R mediated phagocytosis |
| AKT1 | FC gamma R mediated phagocytosis |
| AKT2 | FC gamma R mediated phagocytosis |
| AKT3 | FC gamma R mediated phagocytosis |
| RPS6KB1 | FC gamma R mediated phagocytosis |
| RPS6KB2 | FC gamma R mediated phagocytosis |
| PLCG1 | FC gamma R mediated phagocytosis |
| PLCG2 | FC gamma R mediated phagocytosis |
| PRKCD | FC gamma R mediated phagocytosis |
| PRKCE | FC gamma R mediated phagocytosis |
| RAF1 | FC gamma R mediated phagocytosis |
| MAP2K1 | FC gamma R mediated phagocytosis |
| MAPK1 | FC gamma R mediated phagocytosis |
| MAPK3 | FC gamma R mediated phagocytosis |
| MARCKS | FC gamma R mediated phagocytosis |
| MARCKSL1 | FC gamma R mediated phagocytosis |
| PLD1 | FC gamma R mediated phagocytosis |
| PLD2 | FC gamma R mediated phagocytosis |
| PLPP1 | FC gamma R mediated phagocytosis |
| PLPP3 | FC gamma R mediated phagocytosis |
| PLPP2 | FC gamma R mediated phagocytosis |
| SPHK1 | FC gamma R mediated phagocytosis |
| SPHK2 | FC gamma R mediated phagocytosis |
| PRKCA | FC gamma R mediated phagocytosis |
| PRKCB | FC gamma R mediated phagocytosis |
| PRKCG | FC gamma R mediated phagocytosis |
| NCF1 | FC gamma R mediated phagocytosis |
| GSN | FC gamma R mediated phagocytosis |
| SCIN | FC gamma R mediated phagocytosis |
| VAV3 | FC gamma R mediated phagocytosis |
| VAV1 | FC gamma R mediated phagocytosis |
| VAV2 | FC gamma R mediated phagocytosis |
| CDC42 | FC gamma R mediated phagocytosis |
| WAS | FC gamma R mediated phagocytosis |
| WASL | FC gamma R mediated phagocytosis |
| VASP | FC gamma R mediated phagocytosis |
| ARPC5 | FC gamma R mediated phagocytosis |
| ARPC5L | FC gamma R mediated phagocytosis |
| ARPC4 | FC gamma R mediated phagocytosis |
| ARPC3 | FC gamma R mediated phagocytosis |
| ARPC1B | FC gamma R mediated phagocytosis |
| ARPC1A | FC gamma R mediated phagocytosis |
| ARPC2 | FC gamma R mediated phagocytosis |
| RAC1 | FC gamma R mediated phagocytosis |
| RAC2 | FC gamma R mediated phagocytosis |
| WASF1 | FC gamma R mediated phagocytosis |
| WASF2 | FC gamma R mediated phagocytosis |
| WASF3 | FC gamma R mediated phagocytosis |
| PAK1 | FC gamma R mediated phagocytosis |
| LIMK1 | FC gamma R mediated phagocytosis |
| LIMK2 | FC gamma R mediated phagocytosis |
| CFL1 | FC gamma R mediated phagocytosis |
| CFL2 | FC gamma R mediated phagocytosis |
| PIP5K1C | FC gamma R mediated phagocytosis |
| PIP5K1A | FC gamma R mediated phagocytosis |
| PIP5K1B | FC gamma R mediated phagocytosis |
| ARF6 | FC gamma R mediated phagocytosis |
| CRK | FC gamma R mediated phagocytosis |
| CRKL | FC gamma R mediated phagocytosis |
| DOCK2 | FC gamma R mediated phagocytosis |
| ASAP1 | FC gamma R mediated phagocytosis |
| ASAP3 | FC gamma R mediated phagocytosis |
| ASAP2 | FC gamma R mediated phagocytosis |
| FCGR2B | FC gamma R mediated phagocytosis |
| INPP5D | FC gamma R mediated phagocytosis |
| INPPL1 | FC gamma R mediated phagocytosis |
| GAB2 | FC gamma R mediated phagocytosis |
| LAT | FC gamma R mediated phagocytosis |
| AMPH | FC gamma R mediated phagocytosis |
| BIN1 | FC gamma R mediated phagocytosis |
| MYO10 | FC gamma R mediated phagocytosis |
| PLA2G4B | FC gamma R mediated phagocytosis |
| PLA2G4E | FC gamma R mediated phagocytosis |
| DNM2 | FC gamma R mediated phagocytosis |
| FCGR3A | FC gamma R mediated phagocytosis |
| PLA2G4F | FC gamma R mediated phagocytosis |
| PLA2G4D | FC gamma R mediated phagocytosis |
| PLA2G4A | FC gamma R mediated phagocytosis |
| PLA2G6 | FC gamma R mediated phagocytosis |
| JAM3 | Leukocyte transendothelial migration |
| ITGAM | Leukocyte transendothelial migration |
| ITGB2 | Leukocyte transendothelial migration |
| JAM2 | Leukocyte transendothelial migration |
| ITGA4 | Leukocyte transendothelial migration |
| ITGB1 | Leukocyte transendothelial migration |
| PECAM1 | Leukocyte transendothelial migration |
| CD99 | Leukocyte transendothelial migration |
| ITGAL | Leukocyte transendothelial migration |
| F11R | Leukocyte transendothelial migration |
| CDH5 | Leukocyte transendothelial migration |
| CLDN4 | Leukocyte transendothelial migration |
| CLDN3 | Leukocyte transendothelial migration |
| CLDN7 | Leukocyte transendothelial migration |
| CLDN19 | Leukocyte transendothelial migration |
| CLDN16 | Leukocyte transendothelial migration |
| CLDN14 | Leukocyte transendothelial migration |
| CLDN15 | Leukocyte transendothelial migration |
| CLDN17 | Leukocyte transendothelial migration |
| CLDN20 | Leukocyte transendothelial migration |
| CLDN11 | Leukocyte transendothelial migration |
| CLDN18 | Leukocyte transendothelial migration |
| CLDN22 | Leukocyte transendothelial migration |
| CLDN5 | Leukocyte transendothelial migration |
| CLDN10 | Leukocyte transendothelial migration |
| CLDN8 | Leukocyte transendothelial migration |
| CLDN6 | Leukocyte transendothelial migration |
| CLDN2 | Leukocyte transendothelial migration |
| CLDN1 | Leukocyte transendothelial migration |
| CLDN9 | Leukocyte transendothelial migration |
| CLDN23 | Leukocyte transendothelial migration |
| CLDN25 | Leukocyte transendothelial migration |
| CLDN24 | Leukocyte transendothelial migration |
| OCLN | Leukocyte transendothelial migration |
| ESAM | Leukocyte transendothelial migration |
| VCAM1 | Leukocyte transendothelial migration |
| EZR | Leukocyte transendothelial migration |
| MSN | Leukocyte transendothelial migration |
| ACTB | Leukocyte transendothelial migration |
| ACTG1 | Leukocyte transendothelial migration |
| PIK3CA | Leukocyte transendothelial migration |
| PIK3CD | Leukocyte transendothelial migration |
| PIK3CB | Leukocyte transendothelial migration |
| PIK3R1 | Leukocyte transendothelial migration |
| PIK3R2 | Leukocyte transendothelial migration |
| PIK3R3 | Leukocyte transendothelial migration |
| RAC1 | Leukocyte transendothelial migration |
| CYBB | Leukocyte transendothelial migration |
| CYBA | Leukocyte transendothelial migration |
| NCF2 | Leukocyte transendothelial migration |
| NCF1 | Leukocyte transendothelial migration |
| NCF4 | Leukocyte transendothelial migration |
| CTNNB1 | Leukocyte transendothelial migration |
| CTNND1 | Leukocyte transendothelial migration |
| CTNNA3 | Leukocyte transendothelial migration |
| CTNNA1 | Leukocyte transendothelial migration |
| CTNNA2 | Leukocyte transendothelial migration |
| PTPN11 | Leukocyte transendothelial migration |
| MMP2 | Leukocyte transendothelial migration |
| MMP9 | Leukocyte transendothelial migration |
| MAPK11 | Leukocyte transendothelial migration |
| MAPK12 | Leukocyte transendothelial migration |
| MAPK13 | Leukocyte transendothelial migration |
| MAPK14 | Leukocyte transendothelial migration |
| ICAM1 | Leukocyte transendothelial migration |
| PLCG1 | Leukocyte transendothelial migration |
| PLCG2 | Leukocyte transendothelial migration |
| PRKCA | Leukocyte transendothelial migration |
| PRKCB | Leukocyte transendothelial migration |
| PRKCG | Leukocyte transendothelial migration |
| PTK2 | Leukocyte transendothelial migration |
| PXN | Leukocyte transendothelial migration |
| BCAR1 | Leukocyte transendothelial migration |
| THY1 | Leukocyte transendothelial migration |
| ARHGAP35 | Leukocyte transendothelial migration |
| ARHGAP5 | Leukocyte transendothelial migration |
| RHOA | Leukocyte transendothelial migration |
| ROCK1 | Leukocyte transendothelial migration |
| ROCK2 | Leukocyte transendothelial migration |
| MYL2 | Leukocyte transendothelial migration |
| MYL5 | Leukocyte transendothelial migration |
| MYL7 | Leukocyte transendothelial migration |
| MYL9 | Leukocyte transendothelial migration |
| MYL10 | Leukocyte transendothelial migration |
| MYL12B | Leukocyte transendothelial migration |
| MYL12A | Leukocyte transendothelial migration |
| MYLPF | Leukocyte transendothelial migration |
| AFDN | Leukocyte transendothelial migration |
| RAP1A | Leukocyte transendothelial migration |
| RAP1B | Leukocyte transendothelial migration |
| SIPA1 | Leukocyte transendothelial migration |
| VASP | Leukocyte transendothelial migration |
| ACTN1 | Leukocyte transendothelial migration |
| ACTN4 | Leukocyte transendothelial migration |
| VCL | Leukocyte transendothelial migration |
| CXCL12 | Leukocyte transendothelial migration |
| CXCR4 | Leukocyte transendothelial migration |
| GNAI1 | Leukocyte transendothelial migration |
| GNAI3 | Leukocyte transendothelial migration |
| GNAI2 | Leukocyte transendothelial migration |
| RAPGEF3 | Leukocyte transendothelial migration |
| RAPGEF4 | Leukocyte transendothelial migration |
| RASSF5 | Leukocyte transendothelial migration |
| PTK2B | Leukocyte transendothelial migration |
| ITK | Leukocyte transendothelial migration |
| TXK | Leukocyte transendothelial migration |
| VAV3 | Leukocyte transendothelial migration |
| VAV1 | Leukocyte transendothelial migration |
| VAV2 | Leukocyte transendothelial migration |
| RAC2 | Leukocyte transendothelial migration |
| CDC42 | Leukocyte transendothelial migration |
| RHOH | Leukocyte transendothelial migration |
| CD80 | Intestinal immune network for IgA |
| CD86 | Intestinal immune network for IgA |
| HLA-DMA | Intestinal immune network for IgA |
| HLA-DMB | Intestinal immune network for IgA |
| HLA-DOA | Intestinal immune network for IgA |
| HLA-DOB | Intestinal immune network for IgA |
| HLA-DPA1 | Intestinal immune network for IgA |
| HLA-DPB1 | Intestinal immune network for IgA |
| HLA-DQA1 | Intestinal immune network for IgA |
| HLA-DQA2 | Intestinal immune network for IgA |
| HLA-DQB1 | Intestinal immune network for IgA |
| HLA-DRA | Intestinal immune network for IgA |
| HLA-DRB1 | Intestinal immune network for IgA |
| HLA-DRB3 | Intestinal immune network for IgA |
| HLA-DRB4 | Intestinal immune network for IgA |
| HLA-DRB5 | Intestinal immune network for IgA |
| CD28 | Intestinal immune network for IgA |
| IL2 | Intestinal immune network for IgA |
| IL4 | Intestinal immune network for IgA |
| IL5 | Intestinal immune network for IgA |
| IL6 | Intestinal immune network for IgA |
| IL10 | Intestinal immune network for IgA |
| TGFB1 | Intestinal immune network for IgA |
| TNFSF13 | Intestinal immune network for IgA |
| TNFSF13B | Intestinal immune network for IgA |
| TNFRSF13B | Intestinal immune network for IgA |
| TNFRSF17 | Intestinal immune network for IgA |
| TNFRSF13C | Intestinal immune network for IgA |
| IGH | Intestinal immune network for IgA |
| AICDA | Intestinal immune network for IgA |
| CD40 | Intestinal immune network for IgA |
| CD40LG | Intestinal immune network for IgA |
| ICOS | Intestinal immune network for IgA |
| ICOSLG | Intestinal immune network for IgA |
| ICOS | Intestinal immune network for IgA |
| CCR9 | Intestinal immune network for IgA |
| ITGA4 | Intestinal immune network for IgA |
| ITGB7 | Intestinal immune network for IgA |
| CXCL12 | Intestinal immune network for IgA |
| CXCR4 | Intestinal immune network for IgA |
| CCL28 | Intestinal immune network for IgA |
| CCR10 | Intestinal immune network for IgA |
| CCL25 | Intestinal immune network for IgA |
| MADCAM1 | Intestinal immune network for IgA |
| LTBR | Intestinal immune network for IgA |
| MAP3K14 | Intestinal immune network for IgA |
| IL15 | Intestinal immune network for IgA |
| IL15RA | Intestinal immune network for IgA |
| PIGR | Intestinal immune network for IgA |
| CXCL1 | chemokine SP |
| CXCL2 | chemokine SP |
| CXCL3 | chemokine SP |
| CXCL5 | chemokine SP |
| CXCL6 | chemokine SP |
| PPBP | chemokine SP |
| CXCL8 | chemokine SP |
| CXCL9 | chemokine SP |
| CXCL10 | chemokine SP |
| CXCL11 | chemokine SP |
| CXCL12 | chemokine SP |
| CXCL13 | chemokine SP |
| CXCL16 | chemokine SP |
| PF4 | chemokine SP |
| PF4V1 | chemokine SP |
| CXCL14 | chemokine SP |
| XCL1 | chemokine SP |
| XCL2 | chemokine SP |
| CX3CL1 | chemokine SP |
| CCL1 | chemokine SP |
| CCL2 | chemokine SP |
| CCL3 | chemokine SP |
| CCL3L1 | chemokine SP |
| CCL3L3 | chemokine SP |
| CCL4 | chemokine SP |
| CCL4L2 | chemokine SP |
| CCL4L1 | chemokine SP |
| CCL5 | chemokine SP |
| CCL7 | chemokine SP |
| CCL8 | chemokine SP |
| CCL11 | chemokine SP |
| CCL13 | chemokine SP |
| CCL14 | chemokine SP |
| CCL15 | chemokine SP |
| CCL23 | chemokine SP |
| CCL16 | chemokine SP |
| CCL17 | chemokine SP |
| CCL18 | chemokine SP |
| CCL19 | chemokine SP |
| CCL20 | chemokine SP |
| CCL21 | chemokine SP |
| CCL22 | chemokine SP |
| CCL24 | chemokine SP |
| CCL25 | chemokine SP |
| CCL26 | chemokine SP |
| CCL27 | chemokine SP |
| CCL28 | chemokine SP |
| CXCR2 | chemokine SP |
| CXCR1 | chemokine SP |
| CXCR3 | chemokine SP |
| CXCR4 | chemokine SP |
| CXCR5 | chemokine SP |
| CXCR6 | chemokine SP |
| XCR1 | chemokine SP |
| CX3CR1 | chemokine SP |
| CCR8 | chemokine SP |
| CCR6 | chemokine SP |
| CCR9 | chemokine SP |
| CCR4 | chemokine SP |
| CCR7 | chemokine SP |
| CCR2 | chemokine SP |
| CCR5 | chemokine SP |
| CCR1 | chemokine SP |
| CCR3 | chemokine SP |
| CCR10 | chemokine SP |
| JAK2 | chemokine SP |
| JAK3 | chemokine SP |
| STAT1 | chemokine SP |
| STAT2 | chemokine SP |
| STAT3 | chemokine SP |
| STAT5B | chemokine SP |
| GNAI1 | chemokine SP |
| GNAI3 | chemokine SP |
| GNAI2 | chemokine SP |
| ADCY1 | chemokine SP |
| ADCY2 | chemokine SP |
| ADCY3 | chemokine SP |
| ADCY4 | chemokine SP |
| ADCY5 | chemokine SP |
| ADCY6 | chemokine SP |
| ADCY7 | chemokine SP |
| ADCY8 | chemokine SP |
| ADCY9 | chemokine SP |
| PRKACA | chemokine SP |
| PRKACB | chemokine SP |
| PRKACG | chemokine SP |
| LYN | chemokine SP |
| HCK | chemokine SP |
| FGR | chemokine SP |
| SRC | chemokine SP |
| SHC1 | chemokine SP |
| SHC2 | chemokine SP |
| SHC3 | chemokine SP |
| SHC4 | chemokine SP |
| GRB2 | chemokine SP |
| SOS1 | chemokine SP |
| SOS2 | chemokine SP |
| HRAS | chemokine SP |
| KRAS | chemokine SP |
| NRAS | chemokine SP |
| RAF1 | chemokine SP |
| BRAF | chemokine SP |
| MAP2K1 | chemokine SP |
| MAPK1 | chemokine SP |
| MAPK3 | chemokine SP |
| PIK3CA | chemokine SP |
| PIK3CD | chemokine SP |
| PIK3CB | chemokine SP |
| PIK3R1 | chemokine SP |
| PIK3R2 | chemokine SP |
| PIK3R3 | chemokine SP |
| PIK3CG | chemokine SP |
| PIK3R5 | chemokine SP |
| PIK3R6 | chemokine SP |
| PRKCZ | chemokine SP |
| AKT1 | chemokine SP |
| AKT2 | chemokine SP |
| AKT3 | chemokine SP |
| FOXO3 | chemokine SP |
| CHUK | chemokine SP |
| IKBKB | chemokine SP |
| IKBKG | chemokine SP |
| NFKBIA | chemokine SP |
| NFKBIB | chemokine SP |
| NFKB1 | chemokine SP |
| RELA | chemokine SP |
| BAD | chemokine SP |
| GSK3A | chemokine SP |
| GSK3B | chemokine SP |
| ITK | chemokine SP |
| VAV3 | chemokine SP |
| VAV1 | chemokine SP |
| VAV2 | chemokine SP |
| RAC1 | chemokine SP |
| RAC2 | chemokine SP |
| RAC3 | chemokine SP |
| PAK1 | chemokine SP |
| CDC42 | chemokine SP |
| WAS | chemokine SP |
| WASL | chemokine SP |
| RHOA | chemokine SP |
| ROCK1 | chemokine SP |
| ROCK2 | chemokine SP |
| GNB1 | chemokine SP |
| GNB2 | chemokine SP |
| GNB3 | chemokine SP |
| GNB4 | chemokine SP |
| GNB5 | chemokine SP |
| GNG2 | chemokine SP |
| GNG3 | chemokine SP |
| GNG4 | chemokine SP |
| GNG5 | chemokine SP |
| GNG7 | chemokine SP |
| GNG8 | chemokine SP |
| GNG10 | chemokine SP |
| GNG11 | chemokine SP |
| GNG12 | chemokine SP |
| GNG13 | chemokine SP |
| GNGT1 | chemokine SP |
| GNGT2 | chemokine SP |
| PREX1 | chemokine SP |
| ELMO1 | chemokine SP |
| DOCK2 | chemokine SP |
| PTK2 | chemokine SP |
| PXN | chemokine SP |
| BCAR1 | chemokine SP |
| CRK | chemokine SP |
| CRKL | chemokine SP |
| PTK2B | chemokine SP |
| PLCB1 | chemokine SP |
| PLCB2 | chemokine SP |
| PLCB3 | chemokine SP |
| PLCB4 | chemokine SP |
| RASGRP2 | chemokine SP |
| RAP1A | chemokine SP |
| RAP1B | chemokine SP |
| PARD3 | chemokine SP |
| TIAM1 | chemokine SP |
| PRKCB | chemokine SP |
| PRKCD | chemokine SP |
| NCF1 | chemokine SP |
| GRK7 | chemokine SP |
| GRK1 | chemokine SP |
| GRK2 | chemokine SP |
| GRK3 | chemokine SP |
| GRK4 | chemokine SP |
| GRK5 | chemokine SP |
| GRK6 | chemokine SP |
| ARRB1 | chemokine SP |
| ARRB2 | chemokine SP |
| CLEC7A | C-type lectin receptor SP |
| LSP1 | C-type lectin receptor SP |
| HRAS | C-type lectin receptor SP |
| KRAS | C-type lectin receptor SP |
| NRAS | C-type lectin receptor SP |
| MRAS | C-type lectin receptor SP |
| RRAS | C-type lectin receptor SP |
| RRAS2 | C-type lectin receptor SP |
| RAF1 | C-type lectin receptor SP |
| RELA | C-type lectin receptor SP |
| RELB | C-type lectin receptor SP |
| IL12A | C-type lectin receptor SP |
| IL12B | C-type lectin receptor SP |
| IL1B | C-type lectin receptor SP |
| SYK | C-type lectin receptor SP |
| PTPN11 | C-type lectin receptor SP |
| MAP3K14 | C-type lectin receptor SP |
| CHUK | C-type lectin receptor SP |
| NFKB2 | C-type lectin receptor SP |
| PLCG2 | C-type lectin receptor SP |
| ITPR1 | C-type lectin receptor SP |
| ITPR2 | C-type lectin receptor SP |
| ITPR3 | C-type lectin receptor SP |
| CALML3 | C-type lectin receptor SP |
| CALM2 | C-type lectin receptor SP |
| CALM3 | C-type lectin receptor SP |
| CALM1 | C-type lectin receptor SP |
| CALML6 | C-type lectin receptor SP |
| CALML5 | C-type lectin receptor SP |
| CALML4 | C-type lectin receptor SP |
| PPP3CA | C-type lectin receptor SP |
| PPP3CB | C-type lectin receptor SP |
| PPP3CC | C-type lectin receptor SP |
| PPP3R1 | C-type lectin receptor SP |
| PPP3R2 | C-type lectin receptor SP |
| NFATC1 | C-type lectin receptor SP |
| NFATC2 | C-type lectin receptor SP |
| NFATC3 | C-type lectin receptor SP |
| NFATC4 | C-type lectin receptor SP |
| IL2 | C-type lectin receptor SP |
| IL10 | C-type lectin receptor SP |
| EGR2 | C-type lectin receptor SP |
| EGR3 | C-type lectin receptor SP |
| PTGS2 | C-type lectin receptor SP |
| PRKCD | C-type lectin receptor SP |
| MAPK1 | C-type lectin receptor SP |
| MAPK3 | C-type lectin receptor SP |
| NLRP3 | C-type lectin receptor SP |
| PYCARD | C-type lectin receptor SP |
| CASP1 | C-type lectin receptor SP |
| CARD9 | C-type lectin receptor SP |
| BCL10 | C-type lectin receptor SP |
| MALT1 | C-type lectin receptor SP |
| CASP8 | C-type lectin receptor SP |
| IKBKG | C-type lectin receptor SP |
| IKBKB | C-type lectin receptor SP |
| NFKBIA | C-type lectin receptor SP |
| NFKB1 | C-type lectin receptor SP |
| TNF | C-type lectin receptor SP |
| IL6 | C-type lectin receptor SP |
| IL23A | C-type lectin receptor SP |
| MAPK11 | C-type lectin receptor SP |
| MAPK12 | C-type lectin receptor SP |
| MAPK13 | C-type lectin receptor SP |
| MAPK14 | C-type lectin receptor SP |
| MAPK8 | C-type lectin receptor SP |
| MAPK10 | C-type lectin receptor SP |
| MAPK9 | C-type lectin receptor SP |
| JUN | C-type lectin receptor SP |
| CBLB | C-type lectin receptor SP |
| CLEC6A | C-type lectin receptor SP |
| FCER1G | C-type lectin receptor SP |
| CLEC4M | C-type lectin receptor SP |
| CD209 | C-type lectin receptor SP |
| ARHGEF12 | C-type lectin receptor SP |
| RHOA | C-type lectin receptor SP |
| PAK1 | C-type lectin receptor SP |
| KSR1 | C-type lectin receptor SP |
| PLK3 | C-type lectin receptor SP |
| SRC | C-type lectin receptor SP |
| IKBKE | C-type lectin receptor SP |
| STAT1 | C-type lectin receptor SP |
| IRF9 | C-type lectin receptor SP |
| STAT2 | C-type lectin receptor SP |
| IL17D | C-type lectin receptor SP |
| CYLD | C-type lectin receptor SP |
| BCL3 | C-type lectin receptor SP |
| CCL17 | C-type lectin receptor SP |
| CCL22 | C-type lectin receptor SP |
| MAPKAPK2 | C-type lectin receptor SP |
| CLEC4E | C-type lectin receptor SP |
| PIK3CA | C-type lectin receptor SP |
| PIK3CD | C-type lectin receptor SP |
| PIK3CB | C-type lectin receptor SP |
| PIK3R1 | C-type lectin receptor SP |
| PIK3R2 | C-type lectin receptor SP |
| PIK3R3 | C-type lectin receptor SP |
| AKT1 | C-type lectin receptor SP |
| AKT2 | C-type lectin receptor SP |
| AKT3 | C-type lectin receptor SP |
| MDM2 | C-type lectin receptor SP |
| IRF1 | C-type lectin receptor SP |
| CLEC4D | C-type lectin receptor SP |
| CLEC1B | C-type lectin receptor SP |
| POLR3A | cytosolic DNA sensing pathway |
| POLR3B | cytosolic DNA sensing pathway |
| POLR3C | cytosolic DNA sensing pathway |
| POLR3D | cytosolic DNA sensing pathway |
| POLR3E | cytosolic DNA sensing pathway |
| POLR1C | cytosolic DNA sensing pathway |
| POLR3K | cytosolic DNA sensing pathway |
| POLR1D | cytosolic DNA sensing pathway |
| POLR3H | cytosolic DNA sensing pathway |
| POLR3GL | cytosolic DNA sensing pathway |
| POLR3G | cytosolic DNA sensing pathway |
| POLR3F | cytosolic DNA sensing pathway |
| POLR2E | cytosolic DNA sensing pathway |
| POLR2F | cytosolic DNA sensing pathway |
| POLR2H | cytosolic DNA sensing pathway |
| POLR2K | cytosolic DNA sensing pathway |
| POLR2L | cytosolic DNA sensing pathway |
| DDX58 | cytosolic DNA sensing pathway |
| MAVS | cytosolic DNA sensing pathway |
| NFKB1 | cytosolic DNA sensing pathway |
| RELA | cytosolic DNA sensing pathway |
| IL6 | cytosolic DNA sensing pathway |
| CGAS | cytosolic DNA sensing pathway |
| TMEM173 | cytosolic DNA sensing pathway |
| TBK1 | cytosolic DNA sensing pathway |
| IKBKE | cytosolic DNA sensing pathway |
| IRF3 | cytosolic DNA sensing pathway |
| IRF7 | cytosolic DNA sensing pathway |
| IFNA1 | cytosolic DNA sensing pathway |
| IFNA2 | cytosolic DNA sensing pathway |
| IFNA4 | cytosolic DNA sensing pathway |
| IFNA5 | cytosolic DNA sensing pathway |
| IFNA6 | cytosolic DNA sensing pathway |
| IFNA7 | cytosolic DNA sensing pathway |
| IFNA8 | cytosolic DNA sensing pathway |
| IFNA10 | cytosolic DNA sensing pathway |
| IFNA13 | cytosolic DNA sensing pathway |
| IFNA14 | cytosolic DNA sensing pathway |
| IFNA16 | cytosolic DNA sensing pathway |
| IFNA17 | cytosolic DNA sensing pathway |
| IFNA21 | cytosolic DNA sensing pathway |
| IFNB1 | cytosolic DNA sensing pathway |
| ZBP1 | cytosolic DNA sensing pathway |
| RIPK1 | cytosolic DNA sensing pathway |
| RIPK3 | cytosolic DNA sensing pathway |
| IKBKG | cytosolic DNA sensing pathway |
| CHUK | cytosolic DNA sensing pathway |
| IKBKB | cytosolic DNA sensing pathway |
| NFKBIB | cytosolic DNA sensing pathway |
| NFKBIA | cytosolic DNA sensing pathway |
| CCL4 | cytosolic DNA sensing pathway |
| CCL4L2 | cytosolic DNA sensing pathway |
| CCL4L1 | cytosolic DNA sensing pathway |
| CCL5 | cytosolic DNA sensing pathway |
| CXCL10 | cytosolic DNA sensing pathway |
| AIM2 | cytosolic DNA sensing pathway |
| PYCARD | cytosolic DNA sensing pathway |
| CASP1 | cytosolic DNA sensing pathway |
| IL1B | cytosolic DNA sensing pathway |
| IL18 | cytosolic DNA sensing pathway |
| IL33 | cytosolic DNA sensing pathway |
| TREX1 | cytosolic DNA sensing pathway |
| ADAR | cytosolic DNA sensing pathway |
| DDX58 | RIG-I-like receptor SP |
| IFIH1 | RIG-I-like receptor SP |
| MAVS | RIG-I-like receptor SP |
| DHX58 | RIG-I-like receptor SP |
| TRAF3 | RIG-I-like receptor SP |
| TANK | RIG-I-like receptor SP |
| AZI2 | RIG-I-like receptor SP |
| TBKBP1 | RIG-I-like receptor SP |
| IKBKG | RIG-I-like receptor SP |
| TBK1 | RIG-I-like receptor SP |
| IKBKE | RIG-I-like receptor SP |
| IRF3 | RIG-I-like receptor SP |
| IRF7 | RIG-I-like receptor SP |
| IFNA1 | RIG-I-like receptor SP |
| IFNA2 | RIG-I-like receptor SP |
| IFNA4 | RIG-I-like receptor SP |
| IFNA5 | RIG-I-like receptor SP |
| IFNA6 | RIG-I-like receptor SP |
| IFNA7 | RIG-I-like receptor SP |
| IFNA8 | RIG-I-like receptor SP |
| IFNA10 | RIG-I-like receptor SP |
| IFNA13 | RIG-I-like receptor SP |
| IFNA14 | RIG-I-like receptor SP |
| IFNA16 | RIG-I-like receptor SP |
| IFNA17 | RIG-I-like receptor SP |
| IFNA21 | RIG-I-like receptor SP |
| IFNB1 | RIG-I-like receptor SP |
| IFNW1 | RIG-I-like receptor SP |
| IFNE | RIG-I-like receptor SP |
| IFNK | RIG-I-like receptor SP |
| TRADD | RIG-I-like receptor SP |
| FADD | RIG-I-like receptor SP |
| RIPK1 | RIG-I-like receptor SP |
| CASP8 | RIG-I-like receptor SP |
| CASP10 | RIG-I-like receptor SP |
| CHUK | RIG-I-like receptor SP |
| IKBKB | RIG-I-like receptor SP |
| NFKBIB | RIG-I-like receptor SP |
| NFKBIA | RIG-I-like receptor SP |
| NFKB1 | RIG-I-like receptor SP |
| RELA | RIG-I-like receptor SP |
| TRAF2 | RIG-I-like receptor SP |
| MAP3K7 | RIG-I-like receptor SP |
| TRAF6 | RIG-I-like receptor SP |
| MAP3K1 | RIG-I-like receptor SP |
| MAPK8 | RIG-I-like receptor SP |
| MAPK10 | RIG-I-like receptor SP |
| MAPK9 | RIG-I-like receptor SP |
| MAPK11 | RIG-I-like receptor SP |
| MAPK12 | RIG-I-like receptor SP |
| MAPK13 | RIG-I-like receptor SP |
| MAPK14 | RIG-I-like receptor SP |
| CXCL8 | RIG-I-like receptor SP |
| TNF | RIG-I-like receptor SP |
| IL12A | RIG-I-like receptor SP |
| IL12B | RIG-I-like receptor SP |
| CXCL10 | RIG-I-like receptor SP |
| TRIM25 | RIG-I-like receptor SP |
| CYLD | RIG-I-like receptor SP |
| RNF125 | RIG-I-like receptor SP |
| ISG15 | RIG-I-like receptor SP |
| ATG5 | RIG-I-like receptor SP |
| ATG12 | RIG-I-like receptor SP |
| NLRX1 | RIG-I-like receptor SP |
| TMEM173 | RIG-I-like receptor SP |
| OTUD5 | RIG-I-like receptor SP |
| SIKE1 | RIG-I-like receptor SP |
| DDX3X | RIG-I-like receptor SP |
| PIN1 | RIG-I-like receptor SP |
| TKFC | RIG-I-like receptor SP |
| NOD1 | NOD like receptor SP |
| RIPK2 | NOD like receptor SP |
| IKBKG | NOD like receptor SP |
| CHUK | NOD like receptor SP |
| IKBKB | NOD like receptor SP |
| NFKBIB | NOD like receptor SP |
| NFKBIA | NOD like receptor SP |
| NFKB1 | NOD like receptor SP |
| RELA | NOD like receptor SP |
| IL1B | NOD like receptor SP |
| IL18 | NOD like receptor SP |
| IL6 | NOD like receptor SP |
| TNF | NOD like receptor SP |
| CXCL8 | NOD like receptor SP |
| CXCL1 | NOD like receptor SP |
| CXCL2 | NOD like receptor SP |
| CXCL3 | NOD like receptor SP |
| CCL2 | NOD like receptor SP |
| CCL5 | NOD like receptor SP |
| CAMP | NOD like receptor SP |
| DEFA1 | NOD like receptor SP |
| DEFA3 | NOD like receptor SP |
| DEFA4 | NOD like receptor SP |
| DEFA5 | NOD like receptor SP |
| DEFA6 | NOD like receptor SP |
| DEFA1B | NOD like receptor SP |
| DEFB4A | NOD like receptor SP |
| DEFB4B | NOD like receptor SP |
| DEFB103A | NOD like receptor SP |
| DEFB103B | NOD like receptor SP |
| NOD2 | NOD like receptor SP |
| MAP3K7 | NOD like receptor SP |
| TAB1 | NOD like receptor SP |
| TAB2 | NOD like receptor SP |
| TAB3 | NOD like receptor SP |
| MAPK1 | NOD like receptor SP |
| MAPK3 | NOD like receptor SP |
| MAPK8 | NOD like receptor SP |
| MAPK10 | NOD like receptor SP |
| MAPK9 | NOD like receptor SP |
| MAPK11 | NOD like receptor SP |
| MAPK12 | NOD like receptor SP |
| MAPK13 | NOD like receptor SP |
| MAPK14 | NOD like receptor SP |
| JUN | NOD like receptor SP |
| ATG16L1 | NOD like receptor SP |
| ATG5 | NOD like receptor SP |
| ATG12 | NOD like receptor SP |
| GABARAP | NOD like receptor SP |
| GABARAPL1 | NOD like receptor SP |
| GABARAPL2 | NOD like receptor SP |
| SUGT1 | NOD like receptor SP |
| SHARPIN | NOD like receptor SP |
| RBCK1 | NOD like receptor SP |
| RNF31 | NOD like receptor SP |
| XIAP | NOD like receptor SP |
| CARD6 | NOD like receptor SP |
| TRIP6 | NOD like receptor SP |
| ERBIN | NOD like receptor SP |
| BIRC2 | NOD like receptor SP |
| BIRC3 | NOD like receptor SP |
| TRAF2 | NOD like receptor SP |
| TRAF5 | NOD like receptor SP |
| TRAF6 | NOD like receptor SP |
| CARD9 | NOD like receptor SP |
| TNFAIP3 | NOD like receptor SP |
| MAVS | NOD like receptor SP |
| TRAF3 | NOD like receptor SP |
| TBK1 | NOD like receptor SP |
| IKBKE | NOD like receptor SP |
| TANK | NOD like receptor SP |
| IRF3 | NOD like receptor SP |
| IRF7 | NOD like receptor SP |
| IFNA1 | NOD like receptor SP |
| IFNA2 | NOD like receptor SP |
| IFNA4 | NOD like receptor SP |
| IFNA5 | NOD like receptor SP |
| IFNA6 | NOD like receptor SP |
| IFNA7 | NOD like receptor SP |
| IFNA8 | NOD like receptor SP |
| IFNA10 | NOD like receptor SP |
| IFNA13 | NOD like receptor SP |
| IFNA14 | NOD like receptor SP |
| IFNA16 | NOD like receptor SP |
| IFNA17 | NOD like receptor SP |
| IFNA21 | NOD like receptor SP |
| IFNB1 | NOD like receptor SP |
| NLRX1 | NOD like receptor SP |
| ANTXR2 | NOD like receptor SP |
| ANTXR1 | NOD like receptor SP |
| NLRP1 | NOD like receptor SP |
| PYCARD | NOD like receptor SP |
| CASP1 | NOD like receptor SP |
| BCL2 | NOD like receptor SP |
| BCL2L1 | NOD like receptor SP |
| CTSB | NOD like receptor SP |
| NLRP3 | NOD like receptor SP |
| PANX1 | NOD like receptor SP |
| P2RX7 | NOD like receptor SP |
| TRPM2 | NOD like receptor SP |
| TRPM7 | NOD like receptor SP |
| TRPV2 | NOD like receptor SP |
| CASR | NOD like receptor SP |
| GPRC6A | NOD like receptor SP |
| PLCB1 | NOD like receptor SP |
| PLCB2 | NOD like receptor SP |
| PLCB3 | NOD like receptor SP |
| PLCB4 | NOD like receptor SP |
| ITPR1 | NOD like receptor SP |
| ITPR2 | NOD like receptor SP |
| ITPR3 | NOD like receptor SP |
| VDAC1 | NOD like receptor SP |
| VDAC2 | NOD like receptor SP |
| VDAC3 | NOD like receptor SP |
| MCU | NOD like receptor SP |
| OAS1 | NOD like receptor SP |
| OAS2 | NOD like receptor SP |
| OAS3 | NOD like receptor SP |
| RNASEL | NOD like receptor SP |
| DHX33 | NOD like receptor SP |
| MFN1 | NOD like receptor SP |
| MFN2 | NOD like receptor SP |
| RIPK1 | NOD like receptor SP |
| RIPK3 | NOD like receptor SP |
| DNM1L | NOD like receptor SP |
| NAMPT | NOD like receptor SP |
| CYBB | NOD like receptor SP |
| CYBA | NOD like receptor SP |
| TXNIP | NOD like receptor SP |
| TXN | NOD like receptor SP |
| TXN2 | NOD like receptor SP |
| NEK7 | NOD like receptor SP |
| BRCC3 | NOD like receptor SP |
| HSP90AA1 | NOD like receptor SP |
| HSP90AB1 | NOD like receptor SP |
| CARD8 | NOD like receptor SP |
| PYDC1 | NOD like receptor SP |
| PYDC2 | NOD like receptor SP |
| MEFV | NOD like receptor SP |
| PSTPIP1 | NOD like receptor SP |
| CASP8 | NOD like receptor SP |
| FADD | NOD like receptor SP |
| CASP12 | NOD like receptor SP |
| CARD18 | NOD like receptor SP |
| CARD17 | NOD like receptor SP |
| CARD16 | NOD like receptor SP |
| NLRP6 | NOD like receptor SP |
| NLRP7 | NOD like receptor SP |
| NLRP12 | NOD like receptor SP |
| NAIP | NOD like receptor SP |
| NLRC4 | NOD like receptor SP |
| PRKCD | NOD like receptor SP |
| GBP2 | NOD like receptor SP |
| GBP5 | NOD like receptor SP |
| AIM2 | NOD like receptor SP |
| IFI16 | NOD like receptor SP |
| TP53BP1 | NOD like receptor SP |
| PYDC5 | NOD like receptor SP |
| RHOA | NOD like receptor SP |
| GBP1 | NOD like receptor SP |
| GBP3 | NOD like receptor SP |
| GBP4 | NOD like receptor SP |
| GBP7 | NOD like receptor SP |
| CASP5 | NOD like receptor SP |
| CASP4 | NOD like receptor SP |
| GSDMD | NOD like receptor SP |
| IFNAR1 | NOD like receptor SP |
| IFNAR2 | NOD like receptor SP |
| JAK1 | NOD like receptor SP |
| TYK2 | NOD like receptor SP |
| STAT1 | NOD like receptor SP |
| STAT2 | NOD like receptor SP |
| IRF9 | NOD like receptor SP |
| TLR4 | NOD like receptor SP |
| TICAM1 | NOD like receptor SP |
| MYD88 | NOD like receptor SP |
| IRAK4 | NOD like receptor SP |
| TMEM173 | NOD like receptor SP |
| TLR1 | Toll like receptor SP |
| TLR2 | Toll like receptor SP |
| TLR6 | Toll like receptor SP |
| LBP | Toll like receptor SP |
| CD14 | Toll like receptor SP |
| LY96 | Toll like receptor SP |
| TLR3 | Toll like receptor SP |
| TLR4 | Toll like receptor SP |
| TLR5 | Toll like receptor SP |
| TLR7 | Toll like receptor SP |
| TLR8 | Toll like receptor SP |
| CTSK | Toll like receptor SP |
| TLR9 | Toll like receptor SP |
| RAC1 | Toll like receptor SP |
| PIK3CA | Toll like receptor SP |
| PIK3CD | Toll like receptor SP |
| PIK3CB | Toll like receptor SP |
| PIK3R1 | Toll like receptor SP |
| PIK3R2 | Toll like receptor SP |
| PIK3R3 | Toll like receptor SP |
| AKT1 | Toll like receptor SP |
| AKT2 | Toll like receptor SP |
| AKT3 | Toll like receptor SP |
| TOLLIP | Toll like receptor SP |
| MYD88 | Toll like receptor SP |
| TIRAP | Toll like receptor SP |
| FADD | Toll like receptor SP |
| CASP8 | Toll like receptor SP |
| IRAK4 | Toll like receptor SP |
| IRAK1 | Toll like receptor SP |
| TRAF6 | Toll like receptor SP |
| TAB1 | Toll like receptor SP |
| TAB2 | Toll like receptor SP |
| MAP3K7 | Toll like receptor SP |
| IKBKG | Toll like receptor SP |
| CHUK | Toll like receptor SP |
| IKBKB | Toll like receptor SP |
| NFKBIA | Toll like receptor SP |
| NFKB1 | Toll like receptor SP |
| RELA | Toll like receptor SP |
| MAP3K8 | Toll like receptor SP |
| MAP2K1 | Toll like receptor SP |
| MAP2K2 | Toll like receptor SP |
| MAPK1 | Toll like receptor SP |
| MAPK3 | Toll like receptor SP |
| MAP2K3 | Toll like receptor SP |
| MAP2K6 | Toll like receptor SP |
| MAP2K4 | Toll like receptor SP |
| MAP2K7 | Toll like receptor SP |
| MAPK11 | Toll like receptor SP |
| MAPK12 | Toll like receptor SP |
| MAPK13 | Toll like receptor SP |
| MAPK14 | Toll like receptor SP |
| MAPK8 | Toll like receptor SP |
| MAPK10 | Toll like receptor SP |
| MAPK9 | Toll like receptor SP |
| JUN | Toll like receptor SP |
| FOS | Toll like receptor SP |
| TNF | Toll like receptor SP |
| IL1B | Toll like receptor SP |
| IL6 | Toll like receptor SP |
| IL12A | Toll like receptor SP |
| IL12B | Toll like receptor SP |
| CXCL8 | Toll like receptor SP |
| CCL5 | Toll like receptor SP |
| CCL3 | Toll like receptor SP |
| CCL3L1 | Toll like receptor SP |
| CCL3L3 | Toll like receptor SP |
| CCL4 | Toll like receptor SP |
| CCL4L2 | Toll like receptor SP |
| CCL4L1 | Toll like receptor SP |
| TICAM2 | Toll like receptor SP |
| TICAM1 | Toll like receptor SP |
| RIPK1 | Toll like receptor SP |
| IRF5 | Toll like receptor SP |
| IRF7 | Toll like receptor SP |
| SPP1 | Toll like receptor SP |
| IKBKE | Toll like receptor SP |
| TBK1 | Toll like receptor SP |
| TRAF3 | Toll like receptor SP |
| IRF3 | Toll like receptor SP |
| CD40 | Toll like receptor SP |
| CD80 | Toll like receptor SP |
| CD86 | Toll like receptor SP |
| IFNA1 | Toll like receptor SP |
| IFNA2 | Toll like receptor SP |
| IFNA4 | Toll like receptor SP |
| IFNA5 | Toll like receptor SP |
| IFNA6 | Toll like receptor SP |
| IFNA7 | Toll like receptor SP |
| IFNA8 | Toll like receptor SP |
| IFNA10 | Toll like receptor SP |
| IFNA13 | Toll like receptor SP |
| IFNA14 | Toll like receptor SP |
| IFNA16 | Toll like receptor SP |
| IFNA17 | Toll like receptor SP |
| IFNA21 | Toll like receptor SP |
| IFNB1 | Toll like receptor SP |
| IFNAR1 | Toll like receptor SP |
| IFNAR2 | Toll like receptor SP |
| STAT1 | Toll like receptor SP |
| CXCL10 | Toll like receptor SP |
| CXCL9 | Toll like receptor SP |
| CXCL11 | Toll like receptor SP |
| TBXA2R | platelet activation |
| F2 | platelet activation |
| F2R | platelet activation |
| F2RL3 | platelet activation |
| GNA13 | platelet activation |
| ARHGEF1 | platelet activation |
| ARHGEF12 | platelet activation |
| RHOA | platelet activation |
| ROCK1 | platelet activation |
| ROCK2 | platelet activation |
| PPP1CA | platelet activation |
| PPP1CB | platelet activation |
| PPP1CC | platelet activation |
| PPP1R12A | platelet activation |
| MYL12B | platelet activation |
| MYL12A | platelet activation |
| P2RX1 | platelet activation |
| ORAI1 | platelet activation |
| ITPR1 | platelet activation |
| ITPR2 | platelet activation |
| ITPR3 | platelet activation |
| STIM1 | platelet activation |
| MYLK | platelet activation |
| MYLK2 | platelet activation |
| MYLK3 | platelet activation |
| MYLK4 | platelet activation |
| P2RY1 | platelet activation |
| GNAQ | platelet activation |
| PLCB1 | platelet activation |
| PLCB2 | platelet activation |
| PLCB3 | platelet activation |
| PLCB4 | platelet activation |
| PRKCZ | platelet activation |
| PRKCI | platelet activation |
| RASGRP1 | platelet activation |
| RASGRP2 | platelet activation |
| RAP1A | platelet activation |
| RAP1B | platelet activation |
| APBB1IP | platelet activation |
| TLN1 | platelet activation |
| TLN2 | platelet activation |
| ITGA2B | platelet activation |
| ITGB3 | platelet activation |
| FERMT3 | platelet activation |
| FGA | platelet activation |
| FGB | platelet activation |
| FGG | platelet activation |
| P2RY12 | platelet activation |
| GNAI1 | platelet activation |
| GNAI3 | platelet activation |
| GNAI2 | platelet activation |
| ADCY1 | platelet activation |
| ADCY2 | platelet activation |
| ADCY3 | platelet activation |
| ADCY4 | platelet activation |
| ADCY5 | platelet activation |
| ADCY6 | platelet activation |
| ADCY7 | platelet activation |
| ADCY8 | platelet activation |
| ADCY9 | platelet activation |
| PTGIR | platelet activation |
| GNAS | platelet activation |
| PIK3CG | platelet activation |
| PIK3R5 | platelet activation |
| PIK3R6 | platelet activation |
| PRKACA | platelet activation |
| PRKACB | platelet activation |
| PRKACG | platelet activation |
| VASP | platelet activation |
| ACTB | platelet activation |
| ACTG1 | platelet activation |
| SRC | platelet activation |
| ARHGAP35 | platelet activation |
| FCGR2A | platelet activation |
| SYK | platelet activation |
| COL1A1 | platelet activation |
| COL1A2 | platelet activation |
| COL3A1 | platelet activation |
| GP6 | platelet activation |
| FCER1G | platelet activation |
| LYN | platelet activation |
| FYN | platelet activation |
| ITGA2 | platelet activation |
| ITGB1 | platelet activation |
| PIK3CA | platelet activation |
| PIK3CD | platelet activation |
| PIK3CB | platelet activation |
| PIK3R1 | platelet activation |
| PIK3R2 | platelet activation |
| PIK3R3 | platelet activation |
| LCP2 | platelet activation |
| PLCG2 | platelet activation |
| BTK | platelet activation |
| VWF | platelet activation |
| GP5 | platelet activation |
| GP1BA | platelet activation |
| GP1BB | platelet activation |
| GP9 | platelet activation |
| AKT1 | platelet activation |
| AKT2 | platelet activation |
| AKT3 | platelet activation |
| NOS3 | platelet activation |
| GUCY1A2 | platelet activation |
| GUCY1A1 | platelet activation |
| GUCY1B1 | platelet activation |
| PRKG1 | platelet activation |
| PRKG2 | platelet activation |
| MAPK11 | platelet activation |
| MAPK12 | platelet activation |
| MAPK13 | platelet activation |
| MAPK14 | platelet activation |
| MAPK1 | platelet activation |
| MAPK3 | platelet activation |
| PLA2G4E | platelet activation |
| PLA2G4A | platelet activation |
| JMJD7-PLA2G4B | platelet activation |
| PLA2G4B | platelet activation |
| PLA2G4C | platelet activation |
| PLA2G4D | platelet activation |
| PLA2G4F | platelet activation |
| PTGS1 | platelet activation |
| TBXAS1 | platelet activation |
| SNAP23 | platelet activation |
| VAMP8 | platelet activation |
| F3 | Complement and coagulation cascade |
| F7 | Complement and coagulation cascade |
| F10 | Complement and coagulation cascade |
| F5 | Complement and coagulation cascade |
| F2 | Complement and coagulation cascade |
| F12 | Complement and coagulation cascade |
| F11 | Complement and coagulation cascade |
| F9 | Complement and coagulation cascade |
| VWF | Complement and coagulation cascade |
| F8 | Complement and coagulation cascade |
| THBD | Complement and coagulation cascade |
| PROCR | Complement and coagulation cascade |
| PROC | Complement and coagulation cascade |
| F2R | Complement and coagulation cascade |
| F2RL2 | Complement and coagulation cascade |
| F2RL3 | Complement and coagulation cascade |
| F13A1 | Complement and coagulation cascade |
| F13B | Complement and coagulation cascade |
| CPB2 | Complement and coagulation cascade |
| FGA | Complement and coagulation cascade |
| FGB | Complement and coagulation cascade |
| FGG | Complement and coagulation cascade |
| KLKB1 | Complement and coagulation cascade |
| KNG1 | Complement and coagulation cascade |
| BDKRB1 | Complement and coagulation cascade |
| BDKRB2 | Complement and coagulation cascade |
| PLG | Complement and coagulation cascade |
| TFPI | Complement and coagulation cascade |
| SERPINC1 | Complement and coagulation cascade |
| SERPIND1 | Complement and coagulation cascade |
| SERPINA5 | Complement and coagulation cascade |
| PROS1 | Complement and coagulation cascade |
| SERPINE1 | Complement and coagulation cascade |
| SERPINB2 | Complement and coagulation cascade |
| PLAT | Complement and coagulation cascade |
| PLAU | Complement and coagulation cascade |
| PLAUR | Complement and coagulation cascade |
| SERPINA1 | Complement and coagulation cascade |
| SERPINF2 | Complement and coagulation cascade |
| A2M | Complement and coagulation cascade |
| CFB | Complement and coagulation cascade |
| CFD | Complement and coagulation cascade |
| C3 | Complement and coagulation cascade |
| C5 | Complement and coagulation cascade |
| C6 | Complement and coagulation cascade |
| C7 | Complement and coagulation cascade |
| C8A | Complement and coagulation cascade |
| C8B | Complement and coagulation cascade |
| C8G | Complement and coagulation cascade |
| C9 | Complement and coagulation cascade |
| C1QA | Complement and coagulation cascade |
| C1QB | Complement and coagulation cascade |
| C1QC | Complement and coagulation cascade |
| C1R | Complement and coagulation cascade |
| C1S | Complement and coagulation cascade |
| MBL2 | Complement and coagulation cascade |
| MASP1 | Complement and coagulation cascade |
| MASP2 | Complement and coagulation cascade |
| C2 | Complement and coagulation cascade |
| C4A | Complement and coagulation cascade |
| C4B | Complement and coagulation cascade |
| C3AR1 | Complement and coagulation cascade |
| VSIG4 | Complement and coagulation cascade |
| CR1 | Complement and coagulation cascade |
| CR2 | Complement and coagulation cascade |
| ITGAM | Complement and coagulation cascade |
| ITGB2 | Complement and coagulation cascade |
| ITGAX | Complement and coagulation cascade |
| C5AR1 | Complement and coagulation cascade |
| CFH | Complement and coagulation cascade |
| CFI | Complement and coagulation cascade |
| SERPING1 | Complement and coagulation cascade |
| CD55 | Complement and coagulation cascade |
| CD46 | Complement and coagulation cascade |
| C4BPA | Complement and coagulation cascade |
| C4BPB | Complement and coagulation cascade |
| CD59 | Complement and coagulation cascade |
| CLU | Complement and coagulation cascade |
| VTN | Complement and coagulation cascade |
| KITLG | hematopoiesis |
| IL7 | hematopoiesis |
| IL4 | hematopoiesis |
| CSF2 | hematopoiesis |
| FLT3LG | hematopoiesis |
| IL5 | hematopoiesis |
| CSF3 | hematopoiesis |
| IL3 | hematopoiesis |
| IL6 | hematopoiesis |
| IL11 | hematopoiesis |
| IL1A | hematopoiesis |
| IL1B | hematopoiesis |
| TNF | hematopoiesis |
| CSF1 | hematopoiesis |
| EPO | hematopoiesis |
| THPO | hematopoiesis |
| CD34 | hematopoiesis |
| FLT3 | hematopoiesis |
| DNTT | hematopoiesis |
| HLA-DMA | hematopoiesis |
| HLA-DMB | hematopoiesis |
| HLA-DOA | hematopoiesis |
| HLA-DOB | hematopoiesis |
| HLA-DPA1 | hematopoiesis |
| HLA-DPB1 | hematopoiesis |
| HLA-DQA1 | hematopoiesis |
| HLA-DQA2 | hematopoiesis |
| HLA-DQB1 | hematopoiesis |
| HLA-DRA | hematopoiesis |
| HLA-DRB1 | hematopoiesis |
| HLA-DRB3 | hematopoiesis |
| HLA-DRB4 | hematopoiesis |
| HLA-DRB5 | hematopoiesis |
| CD44 | hematopoiesis |
| KIT | hematopoiesis |
| IL2RA | hematopoiesis |
| IL7R | hematopoiesis |
| TFRC | hematopoiesis |
| CD38 | hematopoiesis |
| CD7 | hematopoiesis |
| CD2 | hematopoiesis |
| CD5 | hematopoiesis |
| CD1A | hematopoiesis |
| CD1B | hematopoiesis |
| CD1C | hematopoiesis |
| CD1D | hematopoiesis |
| CD1E | hematopoiesis |
| CD4 | hematopoiesis |
| CD8A | hematopoiesis |
| CD8B | hematopoiesis |
| CD3D | hematopoiesis |
| CD3E | hematopoiesis |
| CD3G | hematopoiesis |
| MME | hematopoiesis |
| CD9 | hematopoiesis |
| CD19 | hematopoiesis |
| CD22 | hematopoiesis |
| CD24 | hematopoiesis |
| MS4A1 | hematopoiesis |
| CR2 | hematopoiesis |
| CD37 | hematopoiesis |
| IGH | hematopoiesis |
| FCER2 | hematopoiesis |
| CR1 | hematopoiesis |
| CSF2RA | hematopoiesis |
| IL3RA | hematopoiesis |
| CD33 | hematopoiesis |
| IL4R | hematopoiesis |
| IL6R | hematopoiesis |
| FCGR1A | hematopoiesis |
| CSF1R | hematopoiesis |
| ANPEP | hematopoiesis |
| ITGAM | hematopoiesis |
| CD14 | hematopoiesis |
| IL9R | hematopoiesis |
| IL1R1 | hematopoiesis |
| IL1R2 | hematopoiesis |
| CSF3R | hematopoiesis |
| IL5RA | hematopoiesis |
| EPOR | hematopoiesis |
| CD36 | hematopoiesis |
| GYPA | hematopoiesis |
| CD55 | hematopoiesis |
| CD59 | hematopoiesis |
| IL11RA | hematopoiesis |
| ITGB3 | hematopoiesis |
| ITGA2B | hematopoiesis |
| GP9 | hematopoiesis |
| GP1BA | hematopoiesis |
| GP1BB | hematopoiesis |
| GP5 | hematopoiesis |
| ITGA1 | hematopoiesis |
| ITGA2 | hematopoiesis |
| ITGA3 | hematopoiesis |
| ITGA4 | hematopoiesis |
| ITGA5 | hematopoiesis |
| ITGA6 | hematopoiesis |

**Supplementary Table S3:** Gene assignment from KEGG, Gene Ontology and Reactome

| ### Pathway Legend | |  | |  | |  | |  | | |  | |  | |  |  | | | |  | |  | |  | |  | | |  | |  |  | |  | |  | |  | |  | |  | |  |  |  |
| --- | --- | --- | --- | --- | --- | --- | --- | --- | --- | --- | --- | --- | --- | --- | --- | --- | --- | --- | --- | --- | --- | --- | --- | --- | --- | --- | --- | --- | --- | --- | --- | --- | --- | --- | --- | --- | --- | --- | --- | --- | --- | --- | --- | --- | --- | --- |
|  |  | |  | |  | |  | |  |  | |  | |  | | |  |  |  | |  | |  | |  | |  |  | |  | |  |  | |  | |  | |  | |  | |  | |
| APC | Antigen presentation | | | | | | | | |  | |  | |  | | |  |  |  | |  | |  | |  | |  |  | |  | |  |  | |  | |  | |  | |  | |  | |
| C | Complement, coagulation cascade | | | | | | | | | | | | | | | |  |  |  | |  | |  | |  | |  |  | |  | |  |  | |  | |  | |  | |  | |  | |
| I | Innate immunity | | | | | | | |  |  | |  | |  | | |  |  |  | |  | |  | |  | |  |  | |  | |  |  | |  | |  | |  | |  | |  | |
| A | Adaptive immunity | | | | | | | | |  | |  | |  | | |  |  |  | |  | |  | |  | |  |  | |  | |  |  | |  | |  | |  | |  | |  | |
| P | Platelet | | | |  | |  | |  |  | |  | |  | | |  |  |  | |  | |  | |  | |  |  | |  | |  |  | |  | |  | |  | |  | |  | |
| S | Signal transduction | | | | | | | |  |  | |  | |  | | |  |  |  | |  | |  | |  | |  |  | |  | |  |  | |  | |  | |  | |  | |  | |
| H | Hematopoiesis | | | | | |  | |  |  | |  | |  | | |  |  |  | |  | |  | |  | |  |  | |  | |  |  | |  | |  | |  | |  | |  | |
| NK | NK cell | | | |  | |  | |  |  | |  | |  | | |  |  |  | |  | |  | |  | |  |  | |  | |  |  | |  | |  | |  | |  | |  | |
| K | Chemokine-cytokine | | | | | | | | |  | |  | |  | | |  |  |  | |  | |  | |  | |  |  | |  | |  |  | |  | |  | |  | |  | |  | |
| LM | Leukocyte migration | | | | | | | | |  | |  | |  | | |  |  |  | |  | |  | |  | |  |  | |  | |  |  | |  | |  | |  | |  | |  | |
| U | Undetermined | | | | | |  | |  |  | |  | |  | | |  |  |  | |  | |  | |  | |  |  | |  | |  |  | |  | |  | |  | |  | |  | |

| gene | Assign | KEGG pathways | | | | | | | | | | | | GO pathways | | | | | Reactome pathways | | | | | |
| --- | --- | --- | --- | --- | --- | --- | --- | --- | --- | --- | --- | --- | --- | --- | --- | --- | --- | --- | --- | --- | --- | --- | --- | --- |
|  |  | 1 | 2 | 3 | 4 | 5 | 6 | 7 | 8 | 9 | 10 | 11 | 12 | A | I | LM | S | C | A | I | S | C | P | Co |
| A2M | C | C |  |  |  |  |  |  |  |  |  |  |  |  | I |  |  | C |  |  |  |  | P | C |
| ACTB | U | LM | P |  |  |  |  |  |  |  |  |  |  |  |  |  |  |  |  | I | S |  |  |  |
| ACTG1 | I | LM | P |  |  |  |  |  |  |  |  |  |  |  | I |  |  |  |  | I | S |  |  |  |
| ACTN1 | U | LM |  |  |  |  |  |  |  |  |  |  |  |  |  |  |  |  |  |  |  |  | P |  |
| ACTN4 | U | LM |  |  |  |  |  |  |  |  |  |  |  |  |  |  |  |  |  |  |  |  | P |  |
| ADAR | I | I |  |  |  |  |  |  |  |  |  |  |  |  | I |  |  |  |  |  | S |  |  |  |
| ADCY1 | P | K | P |  |  |  |  |  |  |  |  |  |  |  |  |  |  |  |  |  |  |  |  |  |
| ADCY2 | P | K | P |  |  |  |  |  |  |  |  |  |  |  |  |  |  |  |  |  |  |  |  |  |
| ADCY3 | P | K | P |  |  |  |  |  |  |  |  |  |  |  |  |  |  |  |  |  |  |  |  |  |
| ADCY4 | P | K | P |  |  |  |  |  |  |  |  |  |  |  |  |  |  |  |  |  |  |  |  |  |
| ADCY5 | P | K | P |  |  |  |  |  |  |  |  |  |  |  |  |  |  |  |  |  |  |  |  |  |
| ADCY6 | P | K | P |  |  |  |  |  |  |  |  |  |  |  |  |  |  |  |  |  |  |  |  |  |
| ADCY7 | U | K | P |  |  |  |  |  |  |  |  |  |  | A |  |  |  |  |  |  |  |  |  |  |
| ADCY8 | P | K | P |  |  |  |  |  |  |  |  |  |  |  |  |  |  |  |  |  |  |  |  |  |
| ADCY9 | P | K | P |  |  |  |  |  |  |  |  |  |  |  |  |  |  |  |  |  |  |  |  |  |
| AFDN | LM | LM |  |  |  |  |  |  |  |  |  |  |  |  |  |  |  |  |  |  |  |  |  |  |
| AHR | U | A |  |  |  |  |  |  |  |  |  |  |  |  |  | LM |  |  |  |  |  |  |  |  |
| AICDA | A | A |  |  |  |  |  |  |  |  |  |  |  | A |  | LM |  |  |  |  |  |  |  |  |
| AIM2 | I | I | I |  |  |  |  |  |  |  |  |  |  |  | I |  |  |  |  | I |  |  |  |  |
| AKT1 | S | S |  |  |  |  |  |  |  |  |  |  |  |  |  |  |  |  | A |  | S |  | P |  |
| AKT2 | S | S |  |  |  |  |  |  |  |  |  |  |  |  |  |  |  |  | A |  | S |  |  |  |
| AKT3 | A | S |  |  |  |  |  |  |  |  |  |  |  |  |  |  |  |  | A |  |  |  |  |  |
| ALOX5 | U | A |  |  |  |  |  |  |  |  |  |  |  |  |  |  |  |  |  | I | S |  |  |  |
| ALOX5AP | A | A |  |  |  |  |  |  |  |  |  |  |  |  |  |  |  |  |  |  |  |  |  |  |
| AMPH | A | A |  |  |  |  |  |  |  |  |  |  |  |  |  |  |  |  |  |  |  |  |  |  |
| ANPEP | I | H |  |  |  |  |  |  |  |  |  |  |  |  |  |  |  |  |  | I |  |  |  |  |
| ANTXR1 | I | I |  |  |  |  |  |  |  |  |  |  |  |  |  |  |  |  |  |  |  |  |  |  |
| ANTXR2 | I | I |  |  |  |  |  |  |  |  |  |  |  |  |  |  |  |  |  |  |  |  |  |  |
| APBB1IP | P | P |  |  |  |  |  |  |  |  |  |  |  |  |  | LM |  |  |  |  | S |  | P |  |
| ARAF | NK | NK |  |  |  |  |  |  |  |  |  |  |  |  |  |  |  |  |  |  | S |  |  |  |
| ARF6 | U | A |  |  |  |  |  |  |  |  |  |  |  |  | I |  | S |  |  |  |  |  |  |  |
| ARHGAP35 | U | LM | P |  |  |  |  |  |  |  |  |  |  |  |  |  |  |  |  |  |  |  |  |  |
| ARHGAP5 | LM | LM |  |  |  |  |  |  |  |  |  |  |  |  |  |  |  |  |  |  |  |  |  |  |
| ARHGEF1 | P | P |  |  |  |  |  |  |  |  |  |  |  |  |  |  |  |  |  |  |  |  |  |  |
| ARHGEF12 | U | I | P |  |  |  |  |  |  |  |  |  |  |  |  |  |  |  |  |  |  |  |  |  |
| ARPC1A | U | A |  |  |  |  |  |  |  |  |  |  |  |  |  |  |  |  |  | I |  |  |  |  |
| ARPC1B | U | A |  |  |  |  |  |  |  |  |  |  |  |  |  |  |  |  |  | I |  |  |  |  |
| ARPC2 | U | A |  |  |  |  |  |  |  |  |  |  |  |  |  |  |  |  |  | I |  |  |  |  |
| ARPC3 | U | A |  |  |  |  |  |  |  |  |  |  |  |  |  |  |  |  |  | I |  |  |  |  |
| ARPC4 | U | A |  |  |  |  |  |  |  |  |  |  |  |  |  |  |  |  |  | I |  |  |  |  |
| ARPC5 | U | A |  |  |  |  |  |  |  |  |  |  |  |  |  |  |  |  |  | I |  |  |  |  |
| ARPC5L | A | A |  |  |  |  |  |  |  |  |  |  |  |  |  |  |  |  |  |  |  |  |  |  |
| ARRB1 | P | K |  |  |  |  |  |  |  |  |  |  |  |  |  |  |  |  |  |  | S |  | P |  |
| ARRB2 | S | K |  |  |  |  |  |  |  |  |  |  |  |  | I |  | S |  |  |  | S |  | P |  |
| ASAP1 | A | A |  |  |  |  |  |  |  |  |  |  |  |  |  |  |  |  |  |  |  |  |  |  |
| ASAP2 | A | A |  |  |  |  |  |  |  |  |  |  |  |  |  |  |  |  |  |  |  |  |  |  |
| ASAP3 | A | A |  |  |  |  |  |  |  |  |  |  |  |  |  |  |  |  |  |  |  |  |  |  |
| ATG12 | I | I | I |  |  |  |  |  |  |  |  |  |  |  |  |  |  |  |  | I |  |  |  |  |
| ATG16L1 | I | I |  |  |  |  |  |  |  |  |  |  |  |  |  |  |  |  |  |  |  |  |  |  |
| ATG5 | I | I | I |  |  |  |  |  |  |  |  |  |  |  |  | LM |  |  |  | I |  |  |  |  |
| AZI2 | U | I |  |  |  |  |  |  |  |  |  |  |  |  |  | LM |  |  |  |  |  |  |  |  |
| B2M | A | NK |  |  |  |  |  |  |  |  |  |  |  | A | I | LM |  |  | A | I | S |  |  |  |
| BAD | LM | K |  |  |  |  |  |  |  |  |  |  |  |  |  | LM |  |  |  |  |  |  |  |  |
| BCAR1 | U | LM | K |  |  |  |  |  |  |  |  |  |  |  |  |  | S |  |  |  |  |  | P |  |
| BCL10 | A | A | A | I |  |  |  |  |  |  |  |  |  | A | I | LM | S |  | A | I |  |  |  |  |
| BCL2 | I | I |  |  |  |  |  |  |  |  |  |  |  |  |  | LM | S |  |  | I | S |  |  |  |
| BCL2L1 | I | I |  |  |  |  |  |  |  |  |  |  |  |  |  |  |  |  |  | I | S |  |  |  |
| BCL3 | U | I |  |  |  |  |  |  |  |  |  |  |  | A |  | LM |  |  |  |  |  |  |  |  |
| BDKRB1 | C | C |  |  |  |  |  |  |  |  |  |  |  |  |  |  |  |  |  |  |  |  |  |  |
| BDKRB2 | C | C |  |  |  |  |  |  |  |  |  |  |  |  |  |  |  |  |  |  |  |  |  |  |
| BID | NK | NK |  |  |  |  |  |  |  |  |  |  |  |  |  |  |  |  |  |  |  |  |  |  |
| BIN1 | A | A |  |  |  |  |  |  |  |  |  |  |  |  |  |  |  |  |  |  |  |  |  |  |
| BIRC2 | I | I |  |  |  |  |  |  |  |  |  |  |  |  |  |  |  |  |  | I | S |  |  |  |
| BIRC3 | I | I |  |  |  |  |  |  |  |  |  |  |  |  |  |  |  |  |  | I | S |  |  |  |
| BLNK | A | A |  |  |  |  |  |  |  |  |  |  |  |  |  | LM |  |  | A |  | S |  |  |  |
| BRAF | S | NK | K |  |  |  |  |  |  |  |  |  |  |  |  | LM | S |  |  |  | S |  |  |  |
| BRCC3 | I | I |  |  |  |  |  |  |  |  |  |  |  |  |  |  |  |  |  |  |  |  |  |  |
| BTK | A | A | A | P |  |  |  |  |  |  |  |  |  | A | I | LM |  |  | A | I |  |  |  |  |
| BUB1B-PAK6 | A | A |  |  |  |  |  |  |  |  |  |  |  |  |  |  |  |  |  |  |  |  |  |  |
| C1QA | C | C |  |  |  |  |  |  |  |  |  |  |  | A | I | LM |  | C |  | I |  | C |  |  |
| C1QB | C | C |  |  |  |  |  |  |  |  |  |  |  | A | I |  |  | C |  | I |  | C |  |  |
| C1QC | C | C |  |  |  |  |  |  |  |  |  |  |  | A | I |  |  | C |  | I |  | C |  |  |
| C1R | C | C |  |  |  |  |  |  |  |  |  |  |  |  |  |  |  |  |  | I |  | C |  |  |
| C1S | C | C |  |  |  |  |  |  |  |  |  |  |  |  |  |  |  |  |  | I |  | C |  |  |
| C2 | C | C |  |  |  |  |  |  |  |  |  |  |  | A | I |  |  | C |  | I |  | C |  |  |
| C3 | C | C |  |  |  |  |  |  |  |  |  |  |  | A | I |  |  | C | A | I |  | C |  |  |
| C3AR1 | C | C |  |  |  |  |  |  |  |  |  |  |  | A |  |  | S |  |  | I |  | C |  |  |
| C4A | C | C |  |  |  |  |  |  |  |  |  |  |  |  |  |  |  | C |  | I |  | C |  |  |
| C4B | C | C |  |  |  |  |  |  |  |  |  |  |  | A |  |  |  | C |  | I |  | C |  |  |
| C4BPA | C | C |  |  |  |  |  |  |  |  |  |  |  |  |  |  |  |  |  | I |  | C |  |  |
| C4BPB | C | C |  |  |  |  |  |  |  |  |  |  |  |  |  |  |  |  |  | I |  | C |  |  |
| C5 | C | C |  |  |  |  |  |  |  |  |  |  |  |  |  |  |  |  |  | I |  | C |  |  |
| C5AR1 | C | C |  |  |  |  |  |  |  |  |  |  |  |  |  | LM | S |  |  | I |  | C |  |  |
| C6 | C | C |  |  |  |  |  |  |  |  |  |  |  |  |  |  |  | C |  | I |  | C |  |  |
| C7 | C | C |  |  |  |  |  |  |  |  |  |  |  |  |  |  |  |  |  | I |  | C |  |  |
| C8A | C | C |  |  |  |  |  |  |  |  |  |  |  | A | I |  |  | C |  | I |  | C |  |  |
| C8B | C | C |  |  |  |  |  |  |  |  |  |  |  | A | I |  |  | C |  | I |  | C |  |  |
| C8G | C | C |  |  |  |  |  |  |  |  |  |  |  | A | I |  |  | C |  | I |  | C |  |  |
| C9 | C | C |  |  |  |  |  |  |  |  |  |  |  | A | I |  |  | C |  | I |  | C |  |  |
| CALM1 | I | I |  |  |  |  |  |  |  |  |  |  |  |  |  |  |  |  | A | I | S |  | P |  |
| CALM2 | I | I |  |  |  |  |  |  |  |  |  |  |  |  |  |  |  |  |  |  |  |  |  |  |
| CALM3 | I | I |  |  |  |  |  |  |  |  |  |  |  |  |  |  |  |  |  |  |  |  |  |  |
| CALML3 | I | I |  |  |  |  |  |  |  |  |  |  |  |  |  |  |  |  |  |  |  |  |  |  |
| CALML4 | I | I |  |  |  |  |  |  |  |  |  |  |  |  |  |  |  |  |  |  |  |  |  |  |
| CALML5 | I | I |  |  |  |  |  |  |  |  |  |  |  |  |  |  |  |  |  | I |  |  |  |  |
| CALML6 | I | I |  |  |  |  |  |  |  |  |  |  |  |  |  |  |  |  |  |  |  |  |  |  |
| CALR | U | NK |  |  |  |  |  |  |  |  |  |  |  |  |  |  |  |  | A |  |  |  |  |  |
| CAMP | I | I |  |  |  |  |  |  |  |  |  |  |  |  | I |  |  |  |  | I |  |  |  |  |
| CANX | U | NK |  |  |  |  |  |  |  |  |  |  |  |  |  |  |  |  | A |  | S |  |  |  |
| CARD11 | A | A | A |  |  |  |  |  |  |  |  |  |  |  |  | LM |  |  | A | I |  |  |  |  |
| CARD16 | I | I |  |  |  |  |  |  |  |  |  |  |  |  |  |  |  |  |  |  |  |  |  |  |
| CARD17 | I | I |  |  |  |  |  |  |  |  |  |  |  |  |  |  |  |  |  |  |  |  |  |  |
| CARD18 | I | I |  |  |  |  |  |  |  |  |  |  |  |  |  |  |  |  |  |  |  |  |  |  |
| CARD6 | I | I |  |  |  |  |  |  |  |  |  |  |  |  |  |  |  |  |  |  |  |  |  |  |
| CARD8 | I | I |  |  |  |  |  |  |  |  |  |  |  |  |  |  |  |  |  |  |  |  |  |  |
| CARD9 | I | I | I |  |  |  |  |  |  |  |  |  |  |  | I |  |  |  |  | I |  |  |  |  |
| CASP1 | I | I | I | I |  |  |  |  |  |  |  |  |  |  | I | LM |  |  |  | I | S |  |  |  |
| CASP10 | I | I |  |  |  |  |  |  |  |  |  |  |  |  |  |  |  |  |  | I |  |  |  |  |
| CASP12 | I | I |  |  |  |  |  |  |  |  |  |  |  |  |  |  |  |  |  |  |  |  |  |  |
| CASP3 | U | NK |  |  |  |  |  |  |  |  |  |  |  |  |  | LM |  |  |  |  | S |  |  |  |
| CASP4 | I | I |  |  |  |  |  |  |  |  |  |  |  |  | I |  |  |  |  | I |  |  |  |  |
| CASP5 | I | I |  |  |  |  |  |  |  |  |  |  |  |  |  |  |  |  |  |  |  |  |  |  |
| CASP8 | I | I | I | I | I |  |  |  |  |  |  |  |  |  |  |  |  |  |  | I |  |  |  |  |
| CASR | I | I |  |  |  |  |  |  |  |  |  |  |  |  |  |  |  |  |  |  |  |  |  |  |
| CBLB | A | A | I |  |  |  |  |  |  |  |  |  |  |  |  | LM | S |  | A |  |  |  |  |  |
| CCL1 | I | K |  |  |  |  |  |  |  |  |  |  |  |  | I |  |  |  |  |  |  |  |  |  |
| CCL11 | I | K |  |  |  |  |  |  |  |  |  |  |  |  | I |  |  |  |  |  | S |  |  |  |
| CCL13 | K | K |  |  |  |  |  |  |  |  |  |  |  |  |  |  |  |  |  |  |  |  |  |  |
| CCL14 | K | K |  |  |  |  |  |  |  |  |  |  |  |  |  |  |  |  |  |  |  |  |  |  |
| CCL15 | K | K |  |  |  |  |  |  |  |  |  |  |  |  |  |  |  |  |  |  |  |  |  |  |
| CCL16 | K | K |  |  |  |  |  |  |  |  |  |  |  |  |  |  |  |  |  |  |  |  |  |  |
| CCL17 | I | K | I |  |  |  |  |  |  |  |  |  |  |  | I |  |  |  |  | I |  |  |  |  |
| CCL18 | K | K |  |  |  |  |  |  |  |  |  |  |  |  |  |  |  |  |  |  |  |  |  |  |
| CCL19 | U | K |  |  |  |  |  |  |  |  |  |  |  | A | I | LM |  |  |  |  | S |  |  |  |
| CCL2 | I | K | I |  |  |  |  |  |  |  |  |  |  |  | I | LM |  |  |  |  | S |  |  |  |
| CCL20 | U | K |  |  |  |  |  |  |  |  |  |  |  | A | I | LM |  |  |  |  | S |  |  |  |
| CCL21 | K | K |  |  |  |  |  |  |  |  |  |  |  |  |  |  |  |  |  |  |  |  |  |  |
| CCL22 | I | K | I |  |  |  |  |  |  |  |  |  |  |  | I |  |  |  |  | I | S |  |  |  |
| CCL23 | K | K |  |  |  |  |  |  |  |  |  |  |  |  |  |  |  |  |  |  |  |  |  |  |
| CCL24 | I | K |  |  |  |  |  |  |  |  |  |  |  |  | I |  |  |  |  |  |  |  |  |  |
| CCL25 | U | A | K |  |  |  |  |  |  |  |  |  |  |  | I |  |  |  |  |  |  |  |  |  |
| CCL26 | I | K |  |  |  |  |  |  |  |  |  |  |  |  | I |  |  |  |  |  |  |  |  |  |
| CCL27 | K | K |  |  |  |  |  |  |  |  |  |  |  |  |  |  |  |  |  |  |  |  |  |  |
| CCL28 | A | A | K |  |  |  |  |  |  |  |  |  |  |  |  |  |  |  |  |  |  |  |  |  |
| CCL3 | I | K | I |  |  |  |  |  |  |  |  |  |  |  | I |  |  |  |  |  | S |  |  |  |
| CCL3L1 | I | K | I |  |  |  |  |  |  |  |  |  |  |  |  |  |  |  |  |  | S |  |  |  |
| CCL3L3 | I | K | I |  |  |  |  |  |  |  |  |  |  |  |  |  |  |  |  |  |  |  |  |  |
| CCL4 | I | K | I | I |  |  |  |  |  |  |  |  |  |  | I |  |  |  |  |  | S |  |  |  |
| CCL4L1 | I | K | I | I |  |  |  |  |  |  |  |  |  |  |  |  |  |  |  |  |  |  |  |  |
| CCL4L2 | I | K | I | I |  |  |  |  |  |  |  |  |  |  |  |  |  |  |  |  |  |  |  |  |
| CCL5 | I | K | I | I | I |  |  |  |  |  |  |  |  |  | I | LM |  |  |  |  | S |  |  |  |
| CCL7 | I | K |  |  |  |  |  |  |  |  |  |  |  |  | I |  |  |  |  |  |  |  |  |  |
| CCL8 | I | K |  |  |  |  |  |  |  |  |  |  |  |  | I |  |  |  |  |  |  |  |  |  |
| CCR1 | I | K |  |  |  |  |  |  |  |  |  |  |  |  | I |  |  |  |  |  | S |  |  |  |
| CCR10 | A | A | K |  |  |  |  |  |  |  |  |  |  |  |  |  |  |  |  |  |  |  |  |  |
| CCR2 | U | K |  |  |  |  |  |  |  |  |  |  |  | A |  | LM |  |  |  | I | S |  |  |  |
| CCR3 | K | K |  |  |  |  |  |  |  |  |  |  |  |  |  |  |  |  |  |  |  |  |  |  |
| CCR4 | K | K |  |  |  |  |  |  |  |  |  |  |  |  |  |  |  |  |  |  |  |  |  |  |
| CCR5 | U | K |  |  |  |  |  |  |  |  |  |  |  |  |  |  |  |  |  |  | S |  |  |  |
| CCR6 | U | K |  |  |  |  |  |  |  |  |  |  |  | A |  | LM |  |  |  | I |  |  |  |  |
| CCR7 | A | K |  |  |  |  |  |  |  |  |  |  |  | A |  | LM | S |  |  |  |  |  |  |  |
| CCR8 | K | K |  |  |  |  |  |  |  |  |  |  |  |  |  |  |  |  |  |  |  |  |  |  |
| CCR9 | U | A | K |  |  |  |  |  |  |  |  |  |  |  |  | LM |  |  |  |  |  |  |  |  |
| CD14 | I | I | H |  |  |  |  |  |  |  |  |  |  |  | I |  | S |  | A | I |  |  |  |  |
| CD19 | A | A | H |  |  |  |  |  |  |  |  |  |  | A |  | LM | S |  | A | I |  | C |  |  |
| CD1A | A | H |  |  |  |  |  |  |  |  |  |  |  |  |  |  |  |  | A |  |  |  |  |  |
| CD1B | A | H |  |  |  |  |  |  |  |  |  |  |  |  |  |  |  |  | A |  |  |  |  |  |
| CD1C | A | H |  |  |  |  |  |  |  |  |  |  |  |  |  |  |  |  | A |  |  |  |  |  |
| CD1D | A | H |  |  |  |  |  |  |  |  |  |  |  |  |  |  |  |  | A |  |  |  |  |  |
| CD1E | H | H |  |  |  |  |  |  |  |  |  |  |  |  |  |  |  |  |  |  |  |  |  |  |
| CD2 | U | H |  |  |  |  |  |  |  |  |  |  |  |  |  | LM |  |  |  |  |  |  |  | C |
| CD209 | I | I |  |  |  |  |  |  |  |  |  |  |  |  |  |  |  |  | A | I |  |  |  |  |
| CD22 | A | A | H |  |  |  |  |  |  |  |  |  |  |  |  | LM | S |  | A |  |  |  |  |  |
| CD24 | H | H |  |  |  |  |  |  |  |  |  |  |  |  |  |  |  |  |  |  |  |  |  |  |
| CD244 | NK | NK |  |  |  |  |  |  |  |  |  |  |  |  |  |  |  |  |  |  |  |  |  | C |
| CD247 | A | A | NK | A | A |  |  |  |  |  |  |  |  | A |  |  | S |  | A | I |  |  |  |  |
| CD28 | A | A | A |  |  |  |  |  |  |  |  |  |  | A |  | LM | S |  | A |  |  |  |  |  |
| CD33 | U | H |  |  |  |  |  |  |  |  |  |  |  |  |  |  |  |  | A | I |  |  |  |  |
| CD34 | A | H |  |  |  |  |  |  |  |  |  |  |  |  |  |  |  |  | A |  |  |  |  |  |
| CD36 | I | H |  |  |  |  |  |  |  |  |  |  |  |  | I |  | S |  | A | I | S |  | P |  |
| CD37 | LM | H |  |  |  |  |  |  |  |  |  |  |  |  |  | LM |  |  |  |  |  |  |  |  |
| CD38 | LM | H |  |  |  |  |  |  |  |  |  |  |  |  |  | LM | S |  |  |  |  |  |  |  |
| CD3D | A | A | A | A | H |  |  |  |  |  |  |  |  | A |  | LM |  |  | A |  |  |  |  |  |
| CD3E | A | A | A | A | H |  |  |  |  |  |  |  |  | A |  | LM | S |  | A |  |  |  |  |  |
| CD3G | A | A | A | A | H |  |  |  |  |  |  |  |  | A |  | LM |  |  | A | I |  |  |  |  |
| CD4 | A | A | NK | A | A | H |  |  |  |  |  |  |  | A |  | LM |  |  | A | I | S |  |  |  |
| CD40 | A | A | I |  |  |  |  |  |  |  |  |  |  | A |  | LM |  |  | A |  | S |  |  |  |
| CD40LG | A | A | A |  |  |  |  |  |  |  |  |  |  | A |  | LM |  |  | A |  | S |  |  |  |
| CD44 | A | H |  |  |  |  |  |  |  |  |  |  |  | A |  | LM |  |  |  | I | S |  |  | C |
| CD46 | C | C |  |  |  |  |  |  |  |  |  |  |  | A |  | LM |  | C |  | I |  | C |  |  |
| CD48 | A | NK |  |  |  |  |  |  |  |  |  |  |  | A |  | LM |  |  |  |  |  |  |  | C |
| CD5 | LM | H |  |  |  |  |  |  |  |  |  |  |  |  |  | LM |  |  |  |  |  |  |  |  |
| CD55 | C | C | H |  |  |  |  |  |  |  |  |  |  | A | I |  |  | C |  | I |  | C |  |  |
| CD59 | C | C | H |  |  |  |  |  |  |  |  |  |  |  |  |  |  |  |  | I |  | C |  |  |
| CD7 | A | H |  |  |  |  |  |  |  |  |  |  |  | A |  |  |  |  |  |  |  |  |  |  |
| CD72 | A | A |  |  |  |  |  |  |  |  |  |  |  |  |  |  |  |  |  |  |  |  |  |  |
| CD74 | A | NK |  |  |  |  |  |  |  |  |  |  |  | A | I | LM |  |  | A |  |  |  |  | C |
| CD79A | A | A |  |  |  |  |  |  |  |  |  |  |  | A |  | LM | S |  | A |  |  |  |  |  |
| CD79B | A | A |  |  |  |  |  |  |  |  |  |  |  | A |  | LM | S |  | A |  |  |  |  |  |
| CD80 | A | A | I |  |  |  |  |  |  |  |  |  |  |  |  | LM |  |  | A |  | S |  |  |  |
| CD81 | A | A |  |  |  |  |  |  |  |  |  |  |  | A |  | LM | S |  | A | I |  | C |  |  |
| CD86 | A | A | I |  |  |  |  |  |  |  |  |  |  | A | I | LM | S |  | A |  | S |  |  |  |
| CD8A | A | A | NK | H |  |  |  |  |  |  |  |  |  | A |  | LM |  |  | A |  |  |  |  |  |
| CD8B | A | A | NK | H |  |  |  |  |  |  |  |  |  |  |  |  |  |  | A |  |  |  |  |  |
| CD9 | P | H |  |  |  |  |  |  |  |  |  |  |  |  |  |  |  |  |  |  |  |  | P |  |
| CD99 | LM | LM |  |  |  |  |  |  |  |  |  |  |  |  |  | LM |  |  | A |  |  |  |  | C |
| CDC42 | A | A | A | LM | K |  |  |  |  |  |  |  |  |  | I |  |  |  | A | I | S |  | P |  |
| CDH5 | LM | LM |  |  |  |  |  |  |  |  |  |  |  |  |  |  |  |  |  |  |  |  |  |  |
| CDK4 | APC | APC |  |  |  |  |  |  |  |  |  |  |  |  |  |  |  |  |  |  |  |  |  |  |
| CFB | C | C |  |  |  |  |  |  |  |  |  |  |  |  | I |  |  | C |  | I |  | C |  |  |
| CFD | C | C |  |  |  |  |  |  |  |  |  |  |  |  | I |  |  | C |  | I |  | C | P |  |
| CFH | C | C |  |  |  |  |  |  |  |  |  |  |  |  | I |  |  | C |  | I |  | C |  |  |
| CFI | C | C |  |  |  |  |  |  |  |  |  |  |  | A | I |  |  | C |  | I |  | C |  |  |
| CFL1 | U | A |  |  |  |  |  |  |  |  |  |  |  |  |  |  |  |  |  | I | S |  | P |  |
| CFL2 | A | A |  |  |  |  |  |  |  |  |  |  |  |  |  |  |  |  |  |  |  |  |  |  |
| CGAS | I | I |  |  |  |  |  |  |  |  |  |  |  |  | I | LM |  |  |  | I |  |  |  |  |
| CHUK | I | A | A | A | A | K | I | I | I | I | I |  |  |  |  |  |  |  | A | I | S |  |  |  |
| CIITA | U | NK |  |  |  |  |  |  |  |  |  |  |  |  | I |  |  |  |  |  | S |  |  |  |
| CLDN1 | LM | LM |  |  |  |  |  |  |  |  |  |  |  |  |  |  |  |  |  |  |  |  |  |  |
| CLDN10 | LM | LM |  |  |  |  |  |  |  |  |  |  |  |  |  |  |  |  |  |  |  |  |  |  |
| CLDN11 | LM | LM |  |  |  |  |  |  |  |  |  |  |  |  |  |  |  |  |  |  |  |  |  |  |
| CLDN14 | LM | LM |  |  |  |  |  |  |  |  |  |  |  |  |  |  |  |  |  |  |  |  |  |  |
| CLDN15 | LM | LM |  |  |  |  |  |  |  |  |  |  |  |  |  |  |  |  |  |  |  |  |  |  |
| CLDN16 | LM | LM |  |  |  |  |  |  |  |  |  |  |  |  |  |  |  |  |  |  |  |  |  |  |
| CLDN17 | LM | LM |  |  |  |  |  |  |  |  |  |  |  |  |  |  |  |  |  |  |  |  |  |  |
| CLDN18 | LM | LM |  |  |  |  |  |  |  |  |  |  |  |  |  |  |  |  |  |  |  |  |  |  |
| CLDN19 | LM | LM |  |  |  |  |  |  |  |  |  |  |  |  |  |  |  |  |  |  |  |  |  |  |
| CLDN2 | LM | LM |  |  |  |  |  |  |  |  |  |  |  |  |  |  |  |  |  |  |  |  |  |  |
| CLDN20 | LM | LM |  |  |  |  |  |  |  |  |  |  |  |  |  |  |  |  |  |  |  |  |  |  |
| CLDN22 | LM | LM |  |  |  |  |  |  |  |  |  |  |  |  |  |  |  |  |  |  |  |  |  |  |
| CLDN23 | LM | LM |  |  |  |  |  |  |  |  |  |  |  |  |  |  |  |  |  |  |  |  |  |  |
| CLDN24 | LM | LM |  |  |  |  |  |  |  |  |  |  |  |  |  |  |  |  |  |  |  |  |  |  |
| CLDN25 | LM | LM |  |  |  |  |  |  |  |  |  |  |  |  |  |  |  |  |  |  |  |  |  |  |
| CLDN3 | LM | LM |  |  |  |  |  |  |  |  |  |  |  |  |  |  |  |  |  |  |  |  |  |  |
| CLDN4 | LM | LM |  |  |  |  |  |  |  |  |  |  |  |  |  |  |  |  |  |  |  |  |  |  |
| CLDN5 | LM | LM |  |  |  |  |  |  |  |  |  |  |  |  |  |  |  |  |  |  |  |  |  |  |
| CLDN6 | LM | LM |  |  |  |  |  |  |  |  |  |  |  |  |  |  |  |  |  |  |  |  |  |  |
| CLDN7 | LM | LM |  |  |  |  |  |  |  |  |  |  |  |  |  |  |  |  |  |  |  |  |  |  |
| CLDN8 | LM | LM |  |  |  |  |  |  |  |  |  |  |  |  |  |  |  |  |  |  |  |  |  |  |
| CLDN9 | LM | LM |  |  |  |  |  |  |  |  |  |  |  |  |  |  |  |  |  |  |  |  |  |  |
| CLEC1B | U | I |  |  |  |  |  |  |  |  |  |  |  |  |  |  |  |  |  |  |  |  | P |  |
| CLEC4D | I | I |  |  |  |  |  |  |  |  |  |  |  | A | I | LM |  |  |  | I |  |  |  |  |
| CLEC4E | I | I |  |  |  |  |  |  |  |  |  |  |  |  | I | LM | S |  |  | I |  |  |  |  |
| CLEC4M | I | I |  |  |  |  |  |  |  |  |  |  |  |  |  |  |  |  |  |  |  |  |  |  |
| CLEC6A | I | I |  |  |  |  |  |  |  |  |  |  |  |  |  |  |  |  |  | I |  |  |  |  |
| CLEC7A | I | I |  |  |  |  |  |  |  |  |  |  |  |  | I |  | S |  |  | I |  |  |  |  |
| CLU | C | C |  |  |  |  |  |  |  |  |  |  |  |  |  | LM |  |  |  | I |  | C | P |  |
| COL1A1 | P | P |  |  |  |  |  |  |  |  |  |  |  |  |  |  |  |  | A |  |  |  | P | C |
| COL1A2 | P | P |  |  |  |  |  |  |  |  |  |  |  |  |  |  |  |  | A |  | S |  | P | C |
| COL3A1 | U | P |  |  |  |  |  |  |  |  |  |  |  |  |  |  |  |  | A |  |  |  |  |  |
| CPB2 | C | C |  |  |  |  |  |  |  |  |  |  |  |  |  |  |  |  |  | I |  | C |  |  |
| CR1 | C | C | H |  |  |  |  |  |  |  |  |  |  |  |  |  |  |  |  | I |  | C |  |  |
| CR2 | C | A | C | H |  |  |  |  |  |  |  |  |  | A | I | LM |  | C |  | I |  | C |  |  |
| CREB1 | U | NK |  |  |  |  |  |  |  |  |  |  |  |  |  |  |  |  |  | I | S |  |  |  |
| CRK | I | A | K |  |  |  |  |  |  |  |  |  |  |  | I |  |  |  |  | I | S |  | P |  |
| CRKL | S | A | K |  |  |  |  |  |  |  |  |  |  |  |  |  | S |  |  |  | S |  |  |  |
| CSF1 | I | H |  |  |  |  |  |  |  |  |  |  |  |  | I |  |  |  |  |  | S |  |  |  |
| CSF1R | I | H |  |  |  |  |  |  |  |  |  |  |  |  | I |  |  |  |  |  | S |  |  |  |
| CSF2 | U | APC | NK | A | H |  |  |  |  |  |  |  |  |  |  | LM |  |  |  |  | S |  |  |  |
| CSF2RA | U | H |  |  |  |  |  |  |  |  |  |  |  |  |  |  |  |  |  |  | S |  |  |  |
| CSF3 | U | H |  |  |  |  |  |  |  |  |  |  |  |  |  |  |  |  |  |  | S |  |  |  |
| CSF3R | U | H |  |  |  |  |  |  |  |  |  |  |  |  |  |  |  |  |  |  | S |  |  |  |
| CTLA4 | A | A |  |  |  |  |  |  |  |  |  |  |  | A |  | LM | S |  | A |  |  |  |  |  |
| CTNNA1 | LM | LM |  |  |  |  |  |  |  |  |  |  |  |  |  |  |  |  |  |  |  |  |  |  |
| CTNNA2 | LM | LM |  |  |  |  |  |  |  |  |  |  |  |  |  |  |  |  |  |  |  |  |  |  |
| CTNNA3 | LM | LM |  |  |  |  |  |  |  |  |  |  |  |  |  |  |  |  |  |  |  |  |  |  |
| CTNNB1 | LM | LM |  |  |  |  |  |  |  |  |  |  |  |  |  | LM |  |  |  | I |  |  |  |  |
| CTNND1 | LM | LM |  |  |  |  |  |  |  |  |  |  |  |  |  |  |  |  |  |  |  |  |  |  |
| CTSB | I | NK | I |  |  |  |  |  |  |  |  |  |  |  |  |  |  |  | A | I |  |  |  |  |
| CTSK | I | I |  |  |  |  |  |  |  |  |  |  |  |  |  |  |  |  | A | I |  |  |  |  |
| CTSL | U | NK |  |  |  |  |  |  |  |  |  |  |  |  |  |  |  |  | A | I |  |  |  |  |
| CTSS | U | NK |  |  |  |  |  |  |  |  |  |  |  |  |  |  |  |  | A | I |  |  |  |  |
| CX3CL1 | I | K |  |  |  |  |  |  |  |  |  |  |  |  | I | LM |  |  |  |  |  |  |  |  |
| CX3CR1 | I | K |  |  |  |  |  |  |  |  |  |  |  |  | I | LM |  |  |  |  |  |  |  |  |
| CXCL1 | I | K | I |  |  |  |  |  |  |  |  |  |  |  |  |  |  |  |  | I | S |  |  |  |
| CXCL10 | I | K | I | I | I |  |  |  |  |  |  |  |  |  |  |  |  |  |  |  | S |  |  |  |
| CXCL11 | I | K | I |  |  |  |  |  |  |  |  |  |  |  |  |  |  |  |  |  |  |  |  |  |
| CXCL12 | LM | LM | A | K |  |  |  |  |  |  |  |  |  |  |  | LM |  |  |  |  |  |  |  |  |
| CXCL13 | K | K |  |  |  |  |  |  |  |  |  |  |  |  |  |  |  |  |  |  |  |  |  |  |
| CXCL14 | K | K |  |  |  |  |  |  |  |  |  |  |  |  |  |  |  |  |  |  |  |  |  |  |
| CXCL16 | I | K |  |  |  |  |  |  |  |  |  |  |  |  | I |  |  |  |  |  |  |  |  |  |
| CXCL2 | I | K | I |  |  |  |  |  |  |  |  |  |  |  |  |  |  |  |  |  | S |  |  |  |
| CXCL3 | I | K | I |  |  |  |  |  |  |  |  |  |  |  |  |  |  |  |  |  |  |  |  |  |
| CXCL5 | LM | K |  |  |  |  |  |  |  |  |  |  |  |  |  | LM |  |  |  |  |  |  |  |  |
| CXCL6 | K | K |  |  |  |  |  |  |  |  |  |  |  |  |  |  |  |  |  |  |  |  |  |  |
| CXCL8 | I | K | I | I | I |  |  |  |  |  |  |  |  |  |  |  |  |  |  |  | S |  |  |  |
| CXCL9 | I | K | I |  |  |  |  |  |  |  |  |  |  |  |  |  |  |  |  |  |  |  |  |  |
| CXCR1 | I | K |  |  |  |  |  |  |  |  |  |  |  |  |  |  |  |  |  | I |  |  |  |  |
| CXCR2 | U | K |  |  |  |  |  |  |  |  |  |  |  |  |  | LM |  |  |  | I |  |  |  |  |
| CXCR3 | K | K |  |  |  |  |  |  |  |  |  |  |  |  |  |  |  |  |  |  |  |  |  |  |
| CXCR4 | LM | LM | A | K |  |  |  |  |  |  |  |  |  |  |  | LM |  |  |  |  |  |  |  |  |
| CXCR5 | LM | K |  |  |  |  |  |  |  |  |  |  |  |  |  | LM |  |  |  |  |  |  |  |  |
| CXCR6 | K | K |  |  |  |  |  |  |  |  |  |  |  |  |  |  |  |  |  |  |  |  |  |  |
| CYBA | I | LM | I |  |  |  |  |  |  |  |  |  |  |  | I |  | S |  | A | I |  |  |  |  |
| CYBB | I | LM | I |  |  |  |  |  |  |  |  |  |  |  | I |  |  |  | A | I |  |  |  |  |
| CYLD | I | I | I |  |  |  |  |  |  |  |  |  |  |  | I | LM | S |  |  | I |  |  |  |  |
| DAPP1 | A | A |  |  |  |  |  |  |  |  |  |  |  |  |  |  |  |  | A |  |  |  |  |  |
| DDX3X | I | I |  |  |  |  |  |  |  |  |  |  |  |  | I |  |  |  |  | I |  |  |  |  |
| DDX58 | I | I | I |  |  |  |  |  |  |  |  |  |  |  | I |  | S |  |  | I | S |  |  |  |
| DEFA1 | I | I |  |  |  |  |  |  |  |  |  |  |  |  | I |  |  |  |  | I |  |  |  |  |
| DEFA1B | I | I |  |  |  |  |  |  |  |  |  |  |  |  |  |  |  |  |  |  |  |  |  |  |
| DEFA3 | I | I |  |  |  |  |  |  |  |  |  |  |  |  | I |  |  |  |  | I |  |  |  |  |
| DEFA4 | I | I |  |  |  |  |  |  |  |  |  |  |  |  | I |  |  |  |  | I |  |  |  |  |
| DEFA5 | I | I |  |  |  |  |  |  |  |  |  |  |  |  | I |  |  |  |  | I |  |  |  |  |
| DEFA6 | I | I |  |  |  |  |  |  |  |  |  |  |  |  | I |  |  |  |  | I |  |  |  |  |
| DEFB103A | I | I |  |  |  |  |  |  |  |  |  |  |  |  |  |  |  |  |  | I |  |  |  |  |
| DEFB103B | I | I |  |  |  |  |  |  |  |  |  |  |  |  |  |  |  |  |  |  |  |  |  |  |
| DEFB4A | I | I |  |  |  |  |  |  |  |  |  |  |  |  |  |  |  |  |  | I |  |  |  |  |
| DEFB4B | I | I |  |  |  |  |  |  |  |  |  |  |  |  |  |  |  |  |  |  |  |  |  |  |
| DHX33 | I | I |  |  |  |  |  |  |  |  |  |  |  |  |  |  |  |  |  |  |  |  |  |  |
| DHX58 | I | I |  |  |  |  |  |  |  |  |  |  |  |  | I |  | S |  |  | I |  |  |  |  |
| DLG1 | A | A |  |  |  |  |  |  |  |  |  |  |  | A |  | LM |  |  |  |  | S |  |  |  |
| DLL1 | U | A |  |  |  |  |  |  |  |  |  |  |  |  |  | LM |  |  |  |  |  |  |  |  |
| DLL3 | A | A |  |  |  |  |  |  |  |  |  |  |  |  |  |  |  |  |  |  |  |  |  |  |
| DLL4 | U | A |  |  |  |  |  |  |  |  |  |  |  |  |  | LM |  |  |  |  |  |  |  |  |
| DNM1L | I | I |  |  |  |  |  |  |  |  |  |  |  |  |  |  |  |  |  |  |  |  |  |  |
| DNM2 | A | A |  |  |  |  |  |  |  |  |  |  |  |  |  |  |  |  | A | I |  |  |  |  |
| DNTT | H | H |  |  |  |  |  |  |  |  |  |  |  |  |  |  |  |  |  |  |  |  |  |  |
| DOCK2 | U | A | K |  |  |  |  |  |  |  |  |  |  |  |  | LM |  |  |  | I |  |  |  |  |
| EGR2 | I | I |  |  |  |  |  |  |  |  |  |  |  |  |  |  |  |  |  |  |  |  |  |  |
| EGR3 | U | I |  |  |  |  |  |  |  |  |  |  |  |  |  | LM |  |  |  |  |  |  |  |  |
| ELMO1 | I | K |  |  |  |  |  |  |  |  |  |  |  |  |  |  |  |  |  | I |  |  |  |  |
| EPO | LM | H |  |  |  |  |  |  |  |  |  |  |  |  |  | LM |  |  |  |  |  |  |  |  |
| EPOR | H | H |  |  |  |  |  |  |  |  |  |  |  |  |  |  |  |  |  |  |  |  |  |  |
| ERBIN | I | I |  |  |  |  |  |  |  |  |  |  |  |  | I |  | S |  |  |  |  |  |  |  |
| ESAM | LM | LM |  |  |  |  |  |  |  |  |  |  |  |  |  |  |  |  |  |  |  |  |  | C |
| EZR | LM | LM |  |  |  |  |  |  |  |  |  |  |  |  |  |  | S |  |  |  |  |  |  |  |
| F10 | C | C |  |  |  |  |  |  |  |  |  |  |  |  |  |  |  |  |  |  |  |  |  | C |
| F11 | C | C |  |  |  |  |  |  |  |  |  |  |  |  |  |  |  |  |  |  |  |  |  | C |
| F11R | LM | LM |  |  |  |  |  |  |  |  |  |  |  |  |  |  |  |  |  |  |  |  |  | C |
| F12 | C | C |  |  |  |  |  |  |  |  |  |  |  |  |  |  |  |  |  |  |  |  |  | C |
| F13A1 | C | C |  |  |  |  |  |  |  |  |  |  |  |  |  |  |  |  |  |  | S |  | P | C |
| F13B | C | C |  |  |  |  |  |  |  |  |  |  |  |  |  |  |  |  |  |  |  |  |  | C |
| F2 | C | P | C |  |  |  |  |  |  |  |  |  |  |  |  |  |  |  |  | I |  | C | P | C |
| F2R | U | P | C |  |  |  |  |  |  |  |  |  |  |  |  |  |  |  |  |  |  |  | P | C |
| F2RL2 | C | C |  |  |  |  |  |  |  |  |  |  |  |  |  |  |  |  |  |  |  |  | P |  |
| F2RL3 | P | P | C |  |  |  |  |  |  |  |  |  |  |  |  |  |  |  |  |  |  |  | P |  |
| F3 | C | C |  |  |  |  |  |  |  |  |  |  |  |  |  |  |  |  |  |  |  |  |  | C |
| F5 | C | C |  |  |  |  |  |  |  |  |  |  |  |  |  |  |  |  |  |  |  |  | P | C |
| F7 | C | C |  |  |  |  |  |  |  |  |  |  |  |  |  |  |  |  |  |  |  |  |  | C |
| F8 | C | C |  |  |  |  |  |  |  |  |  |  |  |  |  |  |  |  |  |  |  |  | P | C |
| F9 | C | C |  |  |  |  |  |  |  |  |  |  |  |  |  |  |  |  |  |  |  |  |  | C |
| FADD | I | I | I | I |  |  |  |  |  |  |  |  |  | A | I | LM |  |  |  | I |  |  |  |  |
| FAS | U | NK |  |  |  |  |  |  |  |  |  |  |  | A |  | LM |  |  |  |  |  |  |  |  |
| FASLG | U | NK |  |  |  |  |  |  |  |  |  |  |  |  |  |  |  |  |  |  | S |  |  |  |
| FCER1A | A | A |  |  |  |  |  |  |  |  |  |  |  | A |  | LM |  |  |  | I |  |  |  |  |
| FCER1G | I | NK | A | I | P |  |  |  |  |  |  |  |  | A | I | LM | S |  |  | I |  |  | P | C |
| FCER2 | S | H |  |  |  |  |  |  |  |  |  |  |  |  |  |  |  |  |  |  | S |  |  |  |
| FCGR1A | A | A | H |  |  |  |  |  |  |  |  |  |  |  |  |  |  |  | A | I | S |  |  |  |
| FCGR2A | U | A | P |  |  |  |  |  |  |  |  |  |  |  |  |  |  |  |  | I |  |  |  |  |
| FCGR2B | A | A | A |  |  |  |  |  |  |  |  |  |  | A |  | LM | S |  | A |  |  |  |  |  |
| FCGR3A | A | NK | A |  |  |  |  |  |  |  |  |  |  |  |  |  |  |  | A | I |  |  |  |  |
| FCGR3B | U | NK |  |  |  |  |  |  |  |  |  |  |  |  |  |  |  |  |  | I |  |  |  |  |
| FERMT3 | P | P |  |  |  |  |  |  |  |  |  |  |  |  |  |  |  |  |  |  |  |  | P |  |
| FGA | U | P | C |  |  |  |  |  |  |  |  |  |  | A | I |  |  |  |  | I | S |  | P | C |
| FGB | U | P | C |  |  |  |  |  |  |  |  |  |  | A | I |  |  |  |  | I | S |  | P | C |
| FGG | U | P | C |  |  |  |  |  |  |  |  |  |  | A | I |  |  |  |  | I | S |  | P | C |
| FGR | I | K |  |  |  |  |  |  |  |  |  |  |  |  | I | LM |  |  |  | I |  |  | P |  |
| FLT3 | LM | H |  |  |  |  |  |  |  |  |  |  |  |  |  | LM |  |  |  |  | S |  |  |  |
| FLT3LG | U | H |  |  |  |  |  |  |  |  |  |  |  |  |  |  |  |  |  |  | S |  |  |  |
| FOS | A | A | A | A | A | I |  |  |  |  |  |  |  |  |  |  |  |  |  | I | S |  |  |  |
| FOXO3 | U | K |  |  |  |  |  |  |  |  |  |  |  |  |  |  |  |  |  |  | S |  |  |  |
| FOXP3 | A | A |  |  |  |  |  |  |  |  |  |  |  | A |  | LM | S |  |  |  |  |  |  |  |
| FYN | A | A | NK | A | P |  |  |  |  |  |  |  |  | A |  | LM | S |  | A | I | S |  | P | C |
| GAB2 | A | A | A |  |  |  |  |  |  |  |  |  |  |  |  | LM |  |  |  | I | S |  |  |  |
| GABARAP | I | I |  |  |  |  |  |  |  |  |  |  |  |  |  |  |  |  |  |  |  |  |  |  |
| GABARAPL1 | I | I |  |  |  |  |  |  |  |  |  |  |  |  |  |  |  |  |  |  |  |  |  |  |
| GABARAPL2 | I | I |  |  |  |  |  |  |  |  |  |  |  |  |  |  |  |  |  |  |  |  |  |  |
| GATA3 | A | A | A |  |  |  |  |  |  |  |  |  |  | A | I | LM | S |  |  |  | S |  |  |  |
| GBP1 | I | I |  |  |  |  |  |  |  |  |  |  |  |  |  |  |  |  |  |  | S |  |  |  |
| GBP2 | I | I |  |  |  |  |  |  |  |  |  |  |  |  | I |  |  |  |  |  | S |  |  |  |
| GBP3 | I | I |  |  |  |  |  |  |  |  |  |  |  |  | I |  |  |  |  |  | S |  |  |  |
| GBP4 | I | I |  |  |  |  |  |  |  |  |  |  |  |  | I |  |  |  |  |  | S |  |  |  |
| GBP5 | I | I |  |  |  |  |  |  |  |  |  |  |  |  | I |  |  |  |  |  | S |  |  |  |
| GBP7 | I | I |  |  |  |  |  |  |  |  |  |  |  |  | I |  |  |  |  |  | S |  |  |  |
| GNA13 | P | P |  |  |  |  |  |  |  |  |  |  |  |  |  |  |  |  |  |  |  |  | P |  |
| GNAI1 | P | LM | K | P |  |  |  |  |  |  |  |  |  |  |  |  |  |  |  |  |  |  | P |  |
| GNAI2 | P | LM | K | P |  |  |  |  |  |  |  |  |  |  |  |  |  |  |  |  |  |  | P |  |
| GNAI3 | P | LM | K | P |  |  |  |  |  |  |  |  |  |  |  |  |  |  |  |  |  |  | P |  |
| GNAQ | P | P |  |  |  |  |  |  |  |  |  |  |  |  |  |  |  |  |  |  |  |  | P |  |
| GNAS | P | P |  |  |  |  |  |  |  |  |  |  |  |  |  |  |  |  |  |  |  |  | P |  |
| GNB1 | P | K |  |  |  |  |  |  |  |  |  |  |  |  |  |  |  |  |  |  |  |  | P |  |
| GNB2 | P | K |  |  |  |  |  |  |  |  |  |  |  |  |  |  |  |  |  |  |  |  | P |  |
| GNB3 | P | K |  |  |  |  |  |  |  |  |  |  |  |  |  |  |  |  |  |  |  |  | P |  |
| GNB4 | P | K |  |  |  |  |  |  |  |  |  |  |  |  |  |  |  |  |  |  |  |  | P |  |
| GNB5 | P | K |  |  |  |  |  |  |  |  |  |  |  |  |  |  |  |  |  |  |  |  | P |  |
| GNG10 | P | K |  |  |  |  |  |  |  |  |  |  |  |  |  |  |  |  |  |  |  |  | P |  |
| GNG11 | P | K |  |  |  |  |  |  |  |  |  |  |  |  |  |  |  |  |  |  |  |  | P |  |
| GNG12 | P | K |  |  |  |  |  |  |  |  |  |  |  |  |  |  |  |  |  |  |  |  | P |  |
| GNG13 | P | K |  |  |  |  |  |  |  |  |  |  |  |  |  |  |  |  |  |  |  |  | P |  |
| GNG2 | P | K |  |  |  |  |  |  |  |  |  |  |  |  |  |  |  |  |  |  |  |  | P |  |
| GNG3 | P | K |  |  |  |  |  |  |  |  |  |  |  |  |  |  |  |  |  |  |  |  | P |  |
| GNG4 | P | K |  |  |  |  |  |  |  |  |  |  |  |  |  |  |  |  |  |  |  |  | P |  |
| GNG5 | P | K |  |  |  |  |  |  |  |  |  |  |  |  |  |  |  |  |  |  |  |  | P |  |
| GNG7 | P | K |  |  |  |  |  |  |  |  |  |  |  |  |  |  |  |  |  |  |  |  | P |  |
| GNG8 | P | K |  |  |  |  |  |  |  |  |  |  |  |  |  |  |  |  |  |  |  |  | P |  |
| GNGT1 | P | K |  |  |  |  |  |  |  |  |  |  |  |  |  |  |  |  |  |  |  |  | P |  |
| GNGT2 | P | K |  |  |  |  |  |  |  |  |  |  |  |  |  |  |  |  |  |  |  |  | P |  |
| GP1BA | P | P | H |  |  |  |  |  |  |  |  |  |  |  |  |  |  |  |  |  |  |  | P | C |
| GP1BB | P | P | H |  |  |  |  |  |  |  |  |  |  |  |  |  |  |  |  |  |  |  | P | C |
| GP5 | P | P | H |  |  |  |  |  |  |  |  |  |  |  |  |  |  |  |  |  |  |  | P | C |
| GP6 | P | P |  |  |  |  |  |  |  |  |  |  |  |  |  |  |  |  |  |  |  |  | P | C |
| GP9 | P | P | H |  |  |  |  |  |  |  |  |  |  |  |  |  |  |  |  |  |  |  | P | C |
| GPRC6A | I | I |  |  |  |  |  |  |  |  |  |  |  |  |  |  |  |  |  |  |  |  |  |  |
| GRAP2 | A | A |  |  |  |  |  |  |  |  |  |  |  |  |  |  |  |  | A | I |  |  |  |  |
| GRB2 | A | A | NK | A | A | K |  |  |  |  |  |  |  |  |  |  |  |  | A | I | S |  | P | C |
| GRK1 | K | K |  |  |  |  |  |  |  |  |  |  |  |  |  |  |  |  |  |  |  |  |  |  |
| GRK2 | K | K |  |  |  |  |  |  |  |  |  |  |  |  |  |  |  |  |  |  |  |  |  |  |
| GRK3 | K | K |  |  |  |  |  |  |  |  |  |  |  |  |  |  |  |  |  |  |  |  |  |  |
| GRK4 | K | K |  |  |  |  |  |  |  |  |  |  |  |  |  |  |  |  |  |  |  |  |  |  |
| GRK5 | K | K |  |  |  |  |  |  |  |  |  |  |  |  |  |  |  |  |  |  |  |  |  |  |
| GRK6 | K | K |  |  |  |  |  |  |  |  |  |  |  |  |  |  |  |  |  |  |  |  |  |  |
| GRK7 | K | K |  |  |  |  |  |  |  |  |  |  |  |  |  |  |  |  |  |  |  |  |  |  |
| GSDMD | I | I |  |  |  |  |  |  |  |  |  |  |  |  | I |  |  |  |  | I |  |  |  |  |
| GSK3A | K | K |  |  |  |  |  |  |  |  |  |  |  |  |  |  |  |  |  |  |  |  |  |  |
| GSK3B | A | A | A | K |  |  |  |  |  |  |  |  |  |  |  |  |  |  |  |  |  |  |  |  |
| GSN | I | A |  |  |  |  |  |  |  |  |  |  |  |  | I | LM |  |  |  | I |  |  |  |  |
| GUCY1A1 | P | P |  |  |  |  |  |  |  |  |  |  |  |  |  |  |  |  |  |  |  |  | P |  |
| GUCY1A2 | P | P |  |  |  |  |  |  |  |  |  |  |  |  |  |  |  |  |  |  |  |  | P |  |
| GUCY1B1 | P | P |  |  |  |  |  |  |  |  |  |  |  |  |  |  |  |  |  |  |  |  | P |  |
| GYPA | C | H |  |  |  |  |  |  |  |  |  |  |  |  |  |  |  |  |  |  |  |  |  | C |
| GZMB | U | NK |  |  |  |  |  |  |  |  |  |  |  | A |  |  |  |  |  |  |  |  |  |  |
| HCK | I | A | K |  |  |  |  |  |  |  |  |  |  |  | I |  |  |  |  | I | S |  |  |  |
| HCST | U | NK |  |  |  |  |  |  |  |  |  |  |  |  |  |  |  |  | A |  |  |  |  |  |
| HIF1A | A | A |  |  |  |  |  |  |  |  |  |  |  |  |  |  |  |  |  |  | S |  |  |  |
| HLA-A | NK | NK | NK |  |  |  |  |  |  |  |  |  |  |  |  |  |  |  | A | I | S |  |  |  |
| HLA-B | NK | NK | NK |  |  |  |  |  |  |  |  |  |  |  |  |  |  |  | A | I | S |  |  |  |
| HLA-C | NK | NK | NK |  |  |  |  |  |  |  |  |  |  |  |  |  |  |  | A | I | S |  |  |  |
| HLA-DMA | A | NK | A | A | A | H |  |  |  |  |  |  |  |  |  |  |  |  | A |  |  |  |  |  |
| HLA-DMB | A | NK | A | A | A | H |  |  |  |  |  |  |  |  |  |  |  |  | A |  |  |  |  |  |
| HLA-DOA | A | NK | A | A | A | H |  |  |  |  |  |  |  |  |  |  |  |  | A |  |  |  |  |  |
| HLA-DOB | A | NK | A | A | A | H |  |  |  |  |  |  |  |  |  |  |  |  | A |  |  |  |  |  |
| HLA-DPA1 | A | NK | A | A | A | H |  |  |  |  |  |  |  |  |  |  |  |  | A |  | S |  |  |  |
| HLA-DPB1 | A | NK | A | A | A | H |  |  |  |  |  |  |  |  |  |  |  |  | A |  | S |  |  |  |
| HLA-DQA1 | A | NK | A | A | A | H |  |  |  |  |  |  |  |  |  |  |  |  | A |  | S |  |  |  |
| HLA-DQA2 | A | NK | A | A | A | H |  |  |  |  |  |  |  |  |  |  |  |  | A |  | S |  |  |  |
| HLA-DQB1 | A | NK | A | A | A | H |  |  |  |  |  |  |  |  |  |  |  |  | A |  | S |  |  |  |
| HLA-DRA | A | NK | A | A | A | H |  |  |  |  |  |  |  |  |  |  |  |  | A |  | S |  |  |  |
| HLA-DRB1 | A | NK | A | A | A | H |  |  |  |  |  |  |  |  |  |  |  |  | A |  | S |  |  |  |
| HLA-DRB3 | A | NK | A | A | A | H |  |  |  |  |  |  |  |  |  |  |  |  | A |  | S |  |  |  |
| HLA-DRB4 | A | NK | A | A | A | H |  |  |  |  |  |  |  |  |  |  |  |  | A |  | S |  |  |  |
| HLA-DRB5 | A | NK | A | A | A | H |  |  |  |  |  |  |  |  |  |  |  |  | A |  | S |  |  |  |
| HLA-E | NK | NK | NK |  |  |  |  |  |  |  |  |  |  |  |  |  |  |  | A | I | S |  |  |  |
| HLA-F | A | NK |  |  |  |  |  |  |  |  |  |  |  |  |  |  |  |  | A |  | S |  |  |  |
| HLA-G | NK | NK | NK |  |  |  |  |  |  |  |  |  |  |  |  |  |  |  | A |  | S |  |  |  |
| HRAS | A | A | NK | A | A | K | I |  |  |  |  |  |  | A |  |  | S |  | A | I | S |  |  | C |
| HSP90AA1 | I | APC | A | I |  |  |  |  |  |  |  |  |  |  |  | LM |  |  |  | I | S |  |  |  |
| HSP90AB1 | I | APC | A | I |  |  |  |  |  |  |  |  |  |  |  |  |  |  |  | I |  |  |  |  |
| HSPA1A | U | APC |  |  |  |  |  |  |  |  |  |  |  |  |  |  |  |  |  | I |  |  |  |  |
| HSPA1B | U | APC |  |  |  |  |  |  |  |  |  |  |  |  |  |  |  |  |  | I |  |  |  |  |
| HSPA1L | APC | APC |  |  |  |  |  |  |  |  |  |  |  |  |  |  |  |  |  |  |  |  |  |  |
| HSPA2 | APC | APC |  |  |  |  |  |  |  |  |  |  |  |  |  |  |  |  |  |  |  |  |  |  |
| HSPA4 | APC | APC |  |  |  |  |  |  |  |  |  |  |  |  |  |  |  |  |  |  |  |  |  |  |
| HSPA5 | U | NK |  |  |  |  |  |  |  |  |  |  |  |  |  |  |  |  | A |  |  |  | P |  |
| HSPA6 | U | APC |  |  |  |  |  |  |  |  |  |  |  |  |  |  |  |  |  | I |  |  |  |  |
| HSPA8 | U | APC |  |  |  |  |  |  |  |  |  |  |  | A |  |  |  |  |  | I | S |  |  |  |
| ICAM1 | A | NK | LM |  |  |  |  |  |  |  |  |  |  | A |  | LM |  |  | A |  | S |  |  |  |
| ICAM2 | U | NK |  |  |  |  |  |  |  |  |  |  |  |  |  |  |  |  | A | I |  |  |  |  |
| ICOS | A | A | A |  |  |  |  |  |  |  |  |  |  |  |  | LM |  |  | A |  |  |  |  |  |
| ICOSLG | A | A |  |  |  |  |  |  |  |  |  |  |  |  |  |  |  |  | A |  |  |  |  |  |
| IFI16 | I | I |  |  |  |  |  |  |  |  |  |  |  |  |  |  |  |  |  | I |  |  |  |  |
| IFI30 | U | NK |  |  |  |  |  |  |  |  |  |  |  |  |  |  |  |  | A |  | S |  |  |  |
| IFIH1 | I | I |  |  |  |  |  |  |  |  |  |  |  |  | I |  | S |  |  | I |  |  |  |  |
| IFITM1 | A | A |  |  |  |  |  |  |  |  |  |  |  |  | I |  |  |  | A |  | S |  |  |  |
| IFNA1 | I | NK | I | I | I | I |  |  |  |  |  |  |  | A | I | LM |  |  |  | I | S |  |  |  |
| IFNA10 | I | NK | I | I | I | I |  |  |  |  |  |  |  |  |  |  |  |  |  | I | S |  |  |  |
| IFNA13 | I | NK | I | I | I | I |  |  |  |  |  |  |  | A |  | LM |  |  |  |  |  |  |  |  |
| IFNA14 | I | NK | I | I | I | I |  |  |  |  |  |  |  | A |  | LM |  |  |  | I | S |  |  |  |
| IFNA16 | I | NK | I | I | I | I |  |  |  |  |  |  |  | A |  | LM |  |  |  | I | S |  |  |  |
| IFNA17 | I | NK | I | I | I | I |  |  |  |  |  |  |  |  |  |  |  |  |  | I | S |  |  |  |
| IFNA2 | I | NK | I | I | I | I |  |  |  |  |  |  |  | A |  | LM |  |  |  | I | S |  |  |  |
| IFNA21 | I | NK | I | I | I | I |  |  |  |  |  |  |  |  |  |  |  |  |  | I | S |  |  |  |
| IFNA4 | I | NK | I | I | I | I |  |  |  |  |  |  |  | A |  | LM |  |  |  | I | S |  |  |  |
| IFNA5 | I | NK | I | I | I | I |  |  |  |  |  |  |  | A |  | LM |  |  |  | I | S |  |  |  |
| IFNA6 | I | NK | I | I | I | I |  |  |  |  |  |  |  | A |  | LM |  |  |  | I | S |  |  |  |
| IFNA7 | I | NK | I | I | I | I |  |  |  |  |  |  |  | A |  | LM |  |  |  | I | S |  |  |  |
| IFNA8 | I | NK | I | I | I | I |  |  |  |  |  |  |  |  |  |  |  |  |  | I | S |  |  |  |
| IFNAR1 | I | NK | I | I |  |  |  |  |  |  |  |  |  |  | I | LM |  |  |  |  | S |  |  |  |
| IFNAR2 | I | NK | I | I |  |  |  |  |  |  |  |  |  |  | I |  |  |  |  |  | S |  |  |  |
| IFNB1 | I | NK | I | I | I | I |  |  |  |  |  |  |  | A | I | LM |  |  |  | I | S |  |  |  |
| IFNE | A | I |  |  |  |  |  |  |  |  |  |  |  | A |  | LM |  |  |  |  |  |  |  |  |
| IFNG | A | APC | APC | NK | A | A |  |  |  |  |  |  |  | A | I | LM | S |  |  |  | S |  |  |  |
| IFNGR1 | A | NK | A | A |  |  |  |  |  |  |  |  |  |  |  | LM |  |  |  |  | S |  |  |  |
| IFNGR2 | A | NK | A | A |  |  |  |  |  |  |  |  |  |  |  | LM |  |  |  |  | S |  |  |  |
| IFNK | A | I |  |  |  |  |  |  |  |  |  |  |  | A |  | LM |  |  |  |  |  |  |  |  |
| IFNW1 | I | I |  |  |  |  |  |  |  |  |  |  |  |  |  |  |  |  |  |  |  |  |  |  |
| IGH | A | NK | A | A | A | A | H |  |  |  |  |  |  |  |  |  |  |  |  |  |  |  |  |  |
| IKBKB | I | A | A | A | A | K | I | I | I | I | I |  |  |  |  |  |  |  | A | I | S |  |  |  |
| IKBKE | I | I | I | I | I | I |  |  |  |  |  |  |  |  | I |  |  |  |  | I |  |  |  |  |
| IKBKG | I | A | A | A | A | K | I | I | I | I | I |  |  |  |  |  |  |  | A | I | S |  |  |  |
| IL10 | A | A | A | I |  |  |  |  |  |  |  |  |  |  |  | LM |  |  |  |  | S |  |  |  |
| IL11 | U | H |  |  |  |  |  |  |  |  |  |  |  |  |  |  |  |  |  |  | S |  |  |  |
| IL11RA | U | H |  |  |  |  |  |  |  |  |  |  |  |  |  |  |  |  |  |  | S |  |  |  |
| IL12A | I | A | I | I | I |  |  |  |  |  |  |  |  | A | I | LM |  |  |  |  | S |  |  |  |
| IL12B | I | A | I | I | I |  |  |  |  |  |  |  |  | A | I | LM |  |  |  |  | S |  |  |  |
| IL12RB1 | A | A | A |  |  |  |  |  |  |  |  |  |  | A | I | LM |  |  |  |  | S |  |  |  |
| IL12RB2 | A | A |  |  |  |  |  |  |  |  |  |  |  |  |  |  |  |  |  |  | S |  |  |  |
| IL13 | U | A | A |  |  |  |  |  |  |  |  |  |  |  |  | LM |  |  |  |  | S |  |  |  |
| IL15 | U | A |  |  |  |  |  |  |  |  |  |  |  |  |  | LM |  |  |  |  | S |  |  |  |
| IL15RA | U | A |  |  |  |  |  |  |  |  |  |  |  |  |  | LM |  |  |  |  | S |  |  |  |
| IL17A | A | A |  |  |  |  |  |  |  |  |  |  |  |  |  |  |  |  |  |  | S |  |  |  |
| IL17D | U | A | I |  |  |  |  |  |  |  |  |  |  |  |  |  |  |  |  |  |  |  |  |  |
| IL17F | A | A |  |  |  |  |  |  |  |  |  |  |  |  |  |  |  |  |  |  | S |  |  |  |
| IL18 | I | I | I |  |  |  |  |  |  |  |  |  |  | A | I | LM |  |  |  |  | S |  |  |  |
| IL1A | U | H |  |  |  |  |  |  |  |  |  |  |  |  |  |  |  |  |  |  | S |  |  |  |
| IL1B | I | A | I | I | I | I | H |  |  |  |  |  |  | A |  | LM |  | C |  | I | S |  |  |  |
| IL1R1 | A | A | H |  |  |  |  |  |  |  |  |  |  | A |  |  |  |  |  |  | S |  |  |  |
| IL1R2 | U | H |  |  |  |  |  |  |  |  |  |  |  |  |  |  |  |  |  |  | S |  |  |  |
| IL1RAP | U | A |  |  |  |  |  |  |  |  |  |  |  |  | I |  |  |  |  |  | S |  |  |  |
| IL2 | A | A | A | A | A | I |  |  |  |  |  |  |  | A |  | LM |  |  |  |  | S |  |  |  |
| IL21 | U | A |  |  |  |  |  |  |  |  |  |  |  |  | I | LM |  |  |  |  | S |  |  |  |
| IL21R | A | A |  |  |  |  |  |  |  |  |  |  |  |  |  |  |  |  |  |  | S |  |  |  |
| IL22 | A | A |  |  |  |  |  |  |  |  |  |  |  |  |  |  |  |  |  |  | S |  |  |  |
| IL23A | A | A | I |  |  |  |  |  |  |  |  |  |  | A | I | LM |  |  |  |  | S |  |  |  |
| IL23R | A | A |  |  |  |  |  |  |  |  |  |  |  | A | I |  |  |  |  |  | S |  |  |  |
| IL27RA | A | A |  |  |  |  |  |  |  |  |  |  |  | A |  | LM |  |  |  |  | S |  |  |  |
| IL2RA | A | A | A | H |  |  |  |  |  |  |  |  |  |  |  | LM |  |  |  |  | S |  |  |  |
| IL2RB | A | A | A |  |  |  |  |  |  |  |  |  |  |  |  | LM |  |  |  |  | S |  |  |  |
| IL2RG | A | A | A |  |  |  |  |  |  |  |  |  |  |  |  | LM |  |  |  |  | S |  |  |  |
| IL3 | A | A | H |  |  |  |  |  |  |  |  |  |  |  |  |  |  |  |  |  | S |  |  |  |
| IL33 | U | I |  |  |  |  |  |  |  |  |  |  |  | A |  | LM |  |  |  |  | S |  |  |  |
| IL3RA | U | H |  |  |  |  |  |  |  |  |  |  |  |  |  |  |  |  |  |  | S |  |  |  |
| IL4 | A | A | A | A | A | A | H |  |  |  |  |  |  | A | I | LM |  |  |  |  | S |  |  |  |
| IL4R | A | A | A | H |  |  |  |  |  |  |  |  |  |  |  |  |  |  |  |  | S |  |  |  |
| IL5 | A | A | A | A | A | H |  |  |  |  |  |  |  |  |  | LM |  |  |  |  | S |  |  |  |
| IL5RA | U | H |  |  |  |  |  |  |  |  |  |  |  |  |  |  |  |  |  |  | S |  |  |  |
| IL6 | I | A | A | I | I | I | I | H |  |  |  |  |  | A |  | LM |  |  |  |  | S |  |  |  |
| IL6R | A | A | H |  |  |  |  |  |  |  |  |  |  |  |  |  |  |  |  |  | S |  |  |  |
| IL6ST | A | A |  |  |  |  |  |  |  |  |  |  |  |  |  | LM |  |  |  |  | S |  |  |  |
| IL7 | LM | H |  |  |  |  |  |  |  |  |  |  |  |  |  | LM |  |  |  |  | S |  |  |  |
| IL7R | U | H |  |  |  |  |  |  |  |  |  |  |  | A |  | LM |  |  |  |  | S |  |  |  |
| IL9R | U | H |  |  |  |  |  |  |  |  |  |  |  |  |  |  |  |  |  |  | S |  |  |  |
| INPP5D | A | A | A | A |  |  |  |  |  |  |  |  |  | A |  | LM |  |  | A |  | S |  |  | C |
| INPPL1 | A | A | A |  |  |  |  |  |  |  |  |  |  |  |  |  |  |  |  |  | S |  |  |  |
| IRAK1 | I | I |  |  |  |  |  |  |  |  |  |  |  |  | I |  | S |  |  | I | S |  |  |  |
| IRAK4 | I | I | I |  |  |  |  |  |  |  |  |  |  |  | I |  |  |  |  | I | S |  |  |  |
| IRF1 | U | I |  |  |  |  |  |  |  |  |  |  |  | A | I | LM | S |  |  |  | S |  |  |  |
| IRF3 | I | I | I | I | I |  |  |  |  |  |  |  |  |  | I |  | S |  |  | I | S |  |  |  |
| IRF4 | A | A |  |  |  |  |  |  |  |  |  |  |  | A | I | LM | S |  |  |  | S |  |  |  |
| IRF5 | I | I |  |  |  |  |  |  |  |  |  |  |  |  | I |  |  |  |  |  | S |  |  |  |
| IRF7 | I | I | I | I | I |  |  |  |  |  |  |  |  | A | I |  | S |  |  | I | S |  |  |  |
| IRF9 | I | I | I |  |  |  |  |  |  |  |  |  |  |  |  |  |  |  |  |  | S |  |  |  |
| ISG15 | I | I |  |  |  |  |  |  |  |  |  |  |  |  | I |  |  |  |  | I | S |  |  |  |
| ITGA1 | P | H |  |  |  |  |  |  |  |  |  |  |  |  |  |  |  |  |  |  |  |  | P |  |
| ITGA2 | P | P | H |  |  |  |  |  |  |  |  |  |  |  |  |  |  |  |  |  |  |  | P |  |
| ITGA2B | P | P | H |  |  |  |  |  |  |  |  |  |  |  |  |  |  |  |  |  | S |  | P |  |
| ITGA3 | C | H |  |  |  |  |  |  |  |  |  |  |  |  |  |  |  |  |  |  |  |  |  | C |
| ITGA4 | A | LM | A | H |  |  |  |  |  |  |  |  |  |  |  |  |  |  | A |  |  |  |  | C |
| ITGA5 | C | H |  |  |  |  |  |  |  |  |  |  |  |  |  |  |  |  |  |  |  |  |  | C |
| ITGA6 | C | H |  |  |  |  |  |  |  |  |  |  |  |  |  |  |  |  |  |  |  |  |  | C |
| ITGAL | LM | NK | LM |  |  |  |  |  |  |  |  |  |  |  |  | LM |  |  | A | I |  |  |  | C |
| ITGAM | LM | LM | C | H |  |  |  |  |  |  |  |  |  |  |  | LM |  |  |  | I | S |  |  | C |
| ITGAX | C | C |  |  |  |  |  |  |  |  |  |  |  |  |  | LM |  |  |  | I | S |  |  | C |
| ITGB1 | P | LM | P |  |  |  |  |  |  |  |  |  |  |  |  |  |  |  | A |  | S |  | P | C |
| ITGB2 | LM | NK | LM | C |  |  |  |  |  |  |  |  |  |  |  | LM |  |  | A | I | S |  |  | C |
| ITGB3 | P | P | H |  |  |  |  |  |  |  |  |  |  |  |  |  |  |  |  |  | S |  | P | C |
| ITGB7 | A | A |  |  |  |  |  |  |  |  |  |  |  |  |  |  |  |  | A |  |  |  |  |  |
| ITK | A | A | LM | K |  |  |  |  |  |  |  |  |  | A |  | LM | S |  | A | I |  |  |  |  |
| ITPR1 | I | I | I | P |  |  |  |  |  |  |  |  |  |  |  |  |  |  | A | I |  |  | P |  |
| ITPR2 | I | I | I | P |  |  |  |  |  |  |  |  |  |  |  |  |  |  | A | I |  |  | P |  |
| ITPR3 | I | I | I | P |  |  |  |  |  |  |  |  |  |  |  |  |  |  | A | I |  |  | P |  |
| JAG1 | A | A |  |  |  |  |  |  |  |  |  |  |  | A |  |  |  |  |  |  |  |  |  |  |
| JAG2 | U | A |  |  |  |  |  |  |  |  |  |  |  |  |  | LM |  |  |  |  |  |  |  |  |
| JAK1 | A | A | A | I |  |  |  |  |  |  |  |  |  |  |  |  |  |  |  |  | S |  |  |  |
| JAK2 | A | A | A | K |  |  |  |  |  |  |  |  |  | A | I | LM |  |  |  |  | S |  |  |  |
| JAK3 | A | A | A | K |  |  |  |  |  |  |  |  |  | A | I | LM |  |  |  |  | S |  |  |  |
| JAM2 | U | LM |  |  |  |  |  |  |  |  |  |  |  |  |  |  |  |  |  |  |  |  |  | C |
| JAM3 | U | LM |  |  |  |  |  |  |  |  |  |  |  | A |  |  |  |  |  |  |  |  |  | C |
| JMJD7-PLA2G4B | U | A | P |  |  |  |  |  |  |  |  |  |  |  |  |  |  |  |  |  |  |  |  |  |
| JUN | U | A | A | A | A | I | I | I |  |  |  |  |  |  |  | LM |  |  |  | I | S |  |  |  |
| KIR2DL1 | NK | NK | NK |  |  |  |  |  |  |  |  |  |  |  |  |  |  |  | A |  |  |  |  |  |
| KIR2DL2 | NK | NK | NK |  |  |  |  |  |  |  |  |  |  |  |  |  |  |  | A |  |  |  |  |  |
| KIR2DL3 | NK | NK | NK |  |  |  |  |  |  |  |  |  |  |  |  |  |  |  | A |  |  |  |  |  |
| KIR2DL4 | NK | NK | NK |  |  |  |  |  |  |  |  |  |  |  |  |  |  |  | A |  |  |  |  |  |
| KIR2DL5A | NK | NK | NK |  |  |  |  |  |  |  |  |  |  |  |  |  |  |  |  |  |  |  |  |  |
| KIR2DS1 | NK | NK | NK |  |  |  |  |  |  |  |  |  |  |  |  |  |  |  | A | I |  |  |  |  |
| KIR2DS2 | NK | NK | NK |  |  |  |  |  |  |  |  |  |  |  |  |  |  |  | A | I |  |  |  |  |
| KIR2DS3 | NK | NK | NK |  |  |  |  |  |  |  |  |  |  |  |  |  |  |  |  |  |  |  |  |  |
| KIR2DS4 | NK | NK | NK |  |  |  |  |  |  |  |  |  |  |  |  |  |  |  |  | I |  |  |  |  |
| KIR2DS5 | NK | NK | NK |  |  |  |  |  |  |  |  |  |  |  |  |  |  |  |  | I |  |  |  |  |
| KIR3DL1 | NK | NK | NK |  |  |  |  |  |  |  |  |  |  |  |  |  |  |  | A |  |  |  |  |  |
| KIR3DL2 | NK | NK | NK |  |  |  |  |  |  |  |  |  |  |  |  |  |  |  | A |  |  |  |  |  |
| KIR3DL3 | NK | NK |  |  |  |  |  |  |  |  |  |  |  |  |  |  |  |  |  |  |  |  |  |  |
| KIT | LM | H |  |  |  |  |  |  |  |  |  |  |  |  |  | LM |  |  |  |  | S |  |  |  |
| KITLG | U | H |  |  |  |  |  |  |  |  |  |  |  |  |  |  |  |  |  |  | S |  |  |  |
| KLKB1 | C | C |  |  |  |  |  |  |  |  |  |  |  |  |  |  |  |  |  |  |  |  |  | C |
| KLRC1 | NK | NK | NK |  |  |  |  |  |  |  |  |  |  |  |  |  |  |  | A |  |  |  |  |  |
| KLRC2 | NK | NK | NK |  |  |  |  |  |  |  |  |  |  |  |  |  |  |  |  | I |  |  |  |  |
| KLRC3 | NK | NK | NK |  |  |  |  |  |  |  |  |  |  |  |  |  |  |  |  |  |  |  |  |  |
| KLRC4 | NK | NK |  |  |  |  |  |  |  |  |  |  |  |  |  |  |  |  |  |  |  |  |  |  |
| KLRC4-KLRK1 | NK | NK |  |  |  |  |  |  |  |  |  |  |  |  |  |  |  |  |  |  |  |  |  |  |
| KLRD1 | U | NK | NK |  |  |  |  |  |  |  |  |  |  |  | I |  |  |  | A | I |  |  |  |  |
| KLRK1 | A | NK |  |  |  |  |  |  |  |  |  |  |  | A | I | LM | S |  | A | I |  |  |  |  |
| KNG1 | C | C |  |  |  |  |  |  |  |  |  |  |  |  |  |  |  |  |  |  |  |  | P | C |
| KRAS | A | A | NK | A | A | K | I |  |  |  |  |  |  |  |  |  |  |  | A | I | S |  |  | C |
| KSR1 | I | I |  |  |  |  |  |  |  |  |  |  |  |  |  |  |  |  |  |  | S |  |  |  |
| LAT | A | A | NK | A | A | A | A |  |  |  |  |  |  | A |  | LM |  |  | A | I | S |  | P |  |
| LBP | I | I |  |  |  |  |  |  |  |  |  |  |  |  | I | LM | S |  |  | I | S |  |  |  |
| LCK | A | A | NK | A | A |  |  |  |  |  |  |  |  |  |  | LM | S |  | A | I | S |  | P | C |
| LCP2 | A | A | NK | A | P |  |  |  |  |  |  |  |  |  |  | LM | S |  | A | I |  |  | P |  |
| LGMN | U | NK |  |  |  |  |  |  |  |  |  |  |  |  |  |  |  |  | A | I |  |  |  |  |
| LILRB3 | A | A |  |  |  |  |  |  |  |  |  |  |  |  |  |  |  |  | A | I |  |  |  |  |
| LIMK1 | U | A |  |  |  |  |  |  |  |  |  |  |  |  |  |  |  |  |  | I |  |  |  |  |
| LIMK2 | A | A |  |  |  |  |  |  |  |  |  |  |  |  |  |  |  |  |  |  |  |  |  |  |
| LSP1 | I | I |  |  |  |  |  |  |  |  |  |  |  |  |  |  |  |  |  |  |  |  |  |  |
| LTBR | U | A |  |  |  |  |  |  |  |  |  |  |  |  |  | LM |  |  |  |  | S |  |  |  |
| LY96 | I | I |  |  |  |  |  |  |  |  |  |  |  |  | I |  | S |  | A | I |  |  |  |  |
| LYN | A | A | A | A | K | P |  |  |  |  |  |  |  | A | I | LM | S |  | A | I | S |  | P | C |
| MADCAM1 | A | A |  |  |  |  |  |  |  |  |  |  |  |  |  |  |  |  | A |  |  |  |  |  |
| MAF | A | A |  |  |  |  |  |  |  |  |  |  |  |  |  |  |  |  |  |  |  |  |  |  |
| MALT1 | A | A | A | I |  |  |  |  |  |  |  |  |  | A | I | LM | S |  | A | I |  |  |  |  |
| MAML1 | A | A |  |  |  |  |  |  |  |  |  |  |  |  |  |  |  |  |  |  |  |  |  |  |
| MAML2 | A | A |  |  |  |  |  |  |  |  |  |  |  |  |  |  |  |  |  |  |  |  |  |  |
| MAML3 | A | A |  |  |  |  |  |  |  |  |  |  |  |  |  |  |  |  |  |  |  |  |  |  |
| MAP2K1 | A | A | NK | A | A | A | K | I |  |  |  |  |  |  |  |  |  |  |  | I | S |  |  |  |
| MAP2K2 | A | A | NK | A | A | I |  |  |  |  |  |  |  |  |  |  |  |  |  |  | S |  |  |  |
| MAP2K3 | I | A | I |  |  |  |  |  |  |  |  |  |  |  |  |  |  |  |  | I | S |  |  |  |
| MAP2K4 | I | A | I |  |  |  |  |  |  |  |  |  |  |  |  |  |  |  |  | I | S |  |  |  |
| MAP2K6 | I | A | I |  |  |  |  |  |  |  |  |  |  |  |  |  |  |  |  | I | S |  |  |  |
| MAP2K7 | A | A | A | I |  |  |  |  |  |  |  |  |  |  |  |  |  |  |  | I | S |  |  |  |
| MAP3K1 | I | I |  |  |  |  |  |  |  |  |  |  |  |  |  |  |  |  |  | I |  |  |  |  |
| MAP3K14 | A | A | A | I |  |  |  |  |  |  |  |  |  |  |  |  |  |  | A | I | S |  |  |  |
| MAP3K7 | I | A | I | I | I |  |  |  |  |  |  |  |  |  |  |  |  |  | A | I | S |  |  |  |
| MAP3K8 | U | A | I |  |  |  |  |  |  |  |  |  |  |  |  |  |  |  | A | I | S |  |  |  |
| MAPK1 | A | A | NK | A | A | A | A | A | K | I | I | I | P |  |  |  | S |  |  | I | S |  | P |  |
| MAPK10 | I | A | A | A | I | I | I | I |  |  |  |  |  |  |  |  |  |  |  | I | S |  |  |  |
| MAPK11 | I | A | A | A | A | LM | I | I | I | I | P |  |  |  |  |  |  |  |  | I | S |  |  |  |
| MAPK12 | I | A | A | A | A | LM | I | I | I | I | P |  |  |  |  |  |  |  |  | I |  |  |  |  |
| MAPK13 | I | A | A | A | A | LM | I | I | I | I | P |  |  |  |  |  |  |  |  | I |  |  |  |  |
| MAPK14 | I | A | A | A | A | LM | I | I | I | I | P |  |  |  |  |  |  |  |  | I | S |  | P |  |
| MAPK3 | A | A | NK | A | A | A | A | A | K | I | I | I | P |  |  |  |  |  |  | I | S |  | P |  |
| MAPK8 | I | A | A | A | I | I | I | I |  |  |  |  |  |  |  |  |  |  |  | I | S |  |  |  |
| MAPK9 | I | APC | A | A | A | I | I | I | I |  |  |  |  |  |  |  |  |  |  | I | S |  |  |  |
| MAPKAPK2 | I | I |  |  |  |  |  |  |  |  |  |  |  |  | I |  | S |  |  | I | S |  |  |  |
| MARCKS | A | A |  |  |  |  |  |  |  |  |  |  |  |  |  |  |  |  |  |  |  |  |  |  |
| MARCKSL1 | A | A |  |  |  |  |  |  |  |  |  |  |  |  |  |  |  |  |  |  |  |  |  |  |
| MASP1 | C | C |  |  |  |  |  |  |  |  |  |  |  |  | I |  |  | C |  | I |  | C |  |  |
| MASP2 | C | C |  |  |  |  |  |  |  |  |  |  |  | A | I |  |  | C |  | I |  | C |  |  |
| MAVS | I | I | I | I |  |  |  |  |  |  |  |  |  |  | I |  | S |  |  | I |  |  |  |  |
| MBL2 | C | C |  |  |  |  |  |  |  |  |  |  |  | A | I |  |  | C |  | I |  | C |  |  |
| MCU | I | I |  |  |  |  |  |  |  |  |  |  |  |  |  |  |  |  |  |  |  |  |  |  |
| MDM2 | I | I |  |  |  |  |  |  |  |  |  |  |  |  |  |  |  |  |  |  |  |  |  |  |
| MEFV | I | I |  |  |  |  |  |  |  |  |  |  |  |  | I |  |  |  |  | I |  |  |  |  |
| MFN1 | I | I |  |  |  |  |  |  |  |  |  |  |  |  |  |  |  |  |  |  |  |  |  |  |
| MFN2 | I | I |  |  |  |  |  |  |  |  |  |  |  |  |  |  |  |  |  |  |  |  |  |  |
| MICA | U | NK |  |  |  |  |  |  |  |  |  |  |  |  |  |  |  |  | A |  |  |  |  |  |
| MICB | U | NK |  |  |  |  |  |  |  |  |  |  |  |  |  |  |  |  | A |  |  |  |  |  |
| MME | I | H |  |  |  |  |  |  |  |  |  |  |  |  |  |  |  |  |  | I |  |  |  |  |
| MMP2 | U | LM |  |  |  |  |  |  |  |  |  |  |  |  | I |  |  |  |  |  | S |  |  |  |
| MMP9 | U | LM |  |  |  |  |  |  |  |  |  |  |  |  |  |  |  |  |  | I | S |  |  |  |
| MRAS | I | I |  |  |  |  |  |  |  |  |  |  |  |  |  |  |  |  |  |  |  |  |  |  |
| MS4A1 | LM | H |  |  |  |  |  |  |  |  |  |  |  |  |  | LM | S |  |  |  |  |  |  |  |
| MS4A2 | U | A |  |  |  |  |  |  |  |  |  |  |  |  |  | LM |  |  |  | I |  |  |  |  |
| MSN | LM | LM |  |  |  |  |  |  |  |  |  |  |  |  |  | LM |  |  |  |  | S |  |  |  |
| MTOR | A | A |  |  |  |  |  |  |  |  |  |  |  | A |  | LM |  |  | A |  |  |  |  |  |
| MYD88 | I | I | I |  |  |  |  |  |  |  |  |  |  | A | I | LM | S |  | A | I | S |  |  |  |
| MYL10 | LM | LM |  |  |  |  |  |  |  |  |  |  |  |  |  |  |  |  |  |  |  |  |  |  |
| MYL12A | U | LM | P |  |  |  |  |  |  |  |  |  |  |  |  |  |  |  |  |  |  |  |  |  |
| MYL12B | U | LM | P |  |  |  |  |  |  |  |  |  |  |  |  |  |  |  |  |  |  |  |  |  |
| MYL2 | LM | LM |  |  |  |  |  |  |  |  |  |  |  |  |  |  |  |  |  |  |  |  |  |  |
| MYL5 | LM | LM |  |  |  |  |  |  |  |  |  |  |  |  |  |  |  |  |  |  |  |  |  |  |
| MYL7 | LM | LM |  |  |  |  |  |  |  |  |  |  |  |  |  |  |  |  |  |  |  |  |  |  |
| MYL9 | LM | LM |  |  |  |  |  |  |  |  |  |  |  |  |  |  |  |  |  |  |  |  |  |  |
| MYLK | P | P |  |  |  |  |  |  |  |  |  |  |  |  |  |  |  |  |  |  |  |  |  |  |
| MYLK2 | P | P |  |  |  |  |  |  |  |  |  |  |  |  |  |  |  |  |  |  |  |  |  |  |
| MYLK3 | P | P |  |  |  |  |  |  |  |  |  |  |  |  |  |  |  |  |  |  |  |  |  |  |
| MYLK4 | P | P |  |  |  |  |  |  |  |  |  |  |  |  |  |  |  |  |  |  |  |  |  |  |
| MYLPF | LM | LM |  |  |  |  |  |  |  |  |  |  |  |  |  |  |  |  |  |  |  |  |  |  |
| MYO10 | U | A |  |  |  |  |  |  |  |  |  |  |  |  |  |  |  |  |  | I |  |  |  |  |
| NAIP | I | I |  |  |  |  |  |  |  |  |  |  |  |  |  |  |  |  |  |  |  |  |  |  |
| NAMPT | U | I |  |  |  |  |  |  |  |  |  |  |  |  |  | LM |  |  |  |  |  |  |  |  |
| NCF1 | A | A | LM | K |  |  |  |  |  |  |  |  |  |  |  |  |  |  | A | I |  |  |  |  |
| NCF2 | A | LM |  |  |  |  |  |  |  |  |  |  |  |  |  |  |  |  | A | I |  |  |  |  |
| NCF4 | A | LM |  |  |  |  |  |  |  |  |  |  |  |  |  |  |  |  | A | I |  |  |  |  |
| NCK1 | A | A |  |  |  |  |  |  |  |  |  |  |  |  |  | LM |  |  | A | I |  |  |  |  |
| NCK2 | U | A |  |  |  |  |  |  |  |  |  |  |  |  |  | LM |  |  |  |  |  |  |  |  |
| NCR1 | U | NK |  |  |  |  |  |  |  |  |  |  |  |  |  |  |  |  | A |  |  |  |  |  |
| NCR2 | U | NK |  |  |  |  |  |  |  |  |  |  |  |  |  |  |  |  | A | I |  |  |  |  |
| NCR3 | U | NK |  |  |  |  |  |  |  |  |  |  |  |  |  |  |  |  | A |  |  |  |  |  |
| NEK7 | I | I |  |  |  |  |  |  |  |  |  |  |  |  |  |  |  |  |  |  |  |  |  |  |
| NFATC1 | A | A | NK | A | A | A | I |  |  |  |  |  |  |  |  | LM |  |  | A | I |  |  |  |  |
| NFATC2 | A | A | NK | A | A | A | I |  |  |  |  |  |  |  |  | LM | S |  | A | I |  |  |  |  |
| NFATC3 | A | A | A | A | A | I |  |  |  |  |  |  |  |  |  | LM |  |  | A | I |  |  |  |  |
| NFATC4 | I | I |  |  |  |  |  |  |  |  |  |  |  |  |  |  |  |  |  |  |  |  |  |  |
| NFKB1 | I | A | A | A | A | K | I | I | I | I | I |  |  |  | I |  |  |  | A | I | S |  |  |  |
| NFKB2 | I | I |  |  |  |  |  |  |  |  |  |  |  | A | I |  |  |  |  | I | S |  |  |  |
| NFKBIA | I | A | A | A | A | K | I | I | I | I | I |  |  |  | I |  | S |  | A | I | S |  |  |  |
| NFKBIB | A | A | A | A | A | K | I | I | I |  |  |  |  |  |  |  |  |  | A | I | S |  |  |  |
| NFKBIE | A | A | A | A | A |  |  |  |  |  |  |  |  |  |  |  |  |  | A |  |  |  |  |  |
| NFYA | NK | NK |  |  |  |  |  |  |  |  |  |  |  |  |  |  |  |  |  |  |  |  |  |  |
| NFYB | NK | NK |  |  |  |  |  |  |  |  |  |  |  |  |  |  |  |  |  |  |  |  |  |  |
| NFYC | NK | NK |  |  |  |  |  |  |  |  |  |  |  |  |  |  |  |  |  |  |  |  |  |  |
| NLRC4 | I | I |  |  |  |  |  |  |  |  |  |  |  |  | I |  |  |  |  | I |  |  |  |  |
| NLRP1 | I | I |  |  |  |  |  |  |  |  |  |  |  |  |  |  |  |  |  | I |  |  |  |  |
| NLRP12 | I | I |  |  |  |  |  |  |  |  |  |  |  |  |  |  |  |  |  |  |  |  |  |  |
| NLRP3 | I | I | I |  |  |  |  |  |  |  |  |  |  | A | I | LM |  |  |  | I |  |  |  |  |
| NLRP6 | I | I |  |  |  |  |  |  |  |  |  |  |  |  | I |  | S |  |  |  |  |  |  |  |
| NLRP7 | I | I |  |  |  |  |  |  |  |  |  |  |  |  |  |  |  |  |  |  |  |  |  |  |
| NLRX1 | I | I | I |  |  |  |  |  |  |  |  |  |  |  | I |  | S |  |  | I |  |  |  |  |
| NOD1 | I | I |  |  |  |  |  |  |  |  |  |  |  |  | I |  |  |  |  | I | S |  |  |  |
| NOD2 | I | I |  |  |  |  |  |  |  |  |  |  |  | A | I | LM | S |  |  | I | S |  |  |  |
| NOS3 | P | P |  |  |  |  |  |  |  |  |  |  |  |  |  |  |  |  |  | I |  |  | P |  |
| NOTCH1 | A | A |  |  |  |  |  |  |  |  |  |  |  |  |  |  |  |  |  |  |  |  |  |  |
| NOTCH2 | U | A |  |  |  |  |  |  |  |  |  |  |  |  |  | LM |  |  |  |  |  |  |  |  |
| NOTCH3 | A | A |  |  |  |  |  |  |  |  |  |  |  |  |  |  |  |  |  |  |  |  |  |  |
| NRAS | A | A | NK | A | A | K | I |  |  |  |  |  |  | A |  |  | S |  | A | I | S |  |  | C |
| OAS1 | I | I |  |  |  |  |  |  |  |  |  |  |  |  |  |  |  |  |  |  | S |  |  |  |
| OAS2 | I | I |  |  |  |  |  |  |  |  |  |  |  |  | I |  |  |  |  |  | S |  |  |  |
| OAS3 | I | I |  |  |  |  |  |  |  |  |  |  |  |  | I |  |  |  |  |  | S |  |  |  |
| OCLN | LM | LM |  |  |  |  |  |  |  |  |  |  |  |  |  |  |  |  |  |  |  |  |  |  |
| ORAI1 | A | P |  |  |  |  |  |  |  |  |  |  |  | A |  |  |  |  | A |  |  |  | P |  |
| OTUD5 | I | I |  |  |  |  |  |  |  |  |  |  |  | A |  | LM |  |  |  | I |  |  |  |  |
| P2RX1 | P | P |  |  |  |  |  |  |  |  |  |  |  |  |  |  |  |  |  | I |  |  | P |  |
| P2RX7 | I | I |  |  |  |  |  |  |  |  |  |  |  | A |  | LM |  |  |  | I |  |  | P |  |
| P2RY1 | P | P |  |  |  |  |  |  |  |  |  |  |  |  |  |  |  |  |  |  |  |  | P |  |
| P2RY12 | P | P |  |  |  |  |  |  |  |  |  |  |  |  |  |  |  |  |  |  |  |  | P |  |
| PAK1 | A | A | NK | A | K | I |  |  |  |  |  |  |  |  |  |  |  |  | A | I |  |  |  |  |
| PAK2 | A | A |  |  |  |  |  |  |  |  |  |  |  |  |  |  |  |  | A | I | S |  |  |  |
| PAK3 | A | A |  |  |  |  |  |  |  |  |  |  |  |  |  |  |  |  | A | I |  |  |  |  |
| PAK4 | A | A |  |  |  |  |  |  |  |  |  |  |  |  |  |  |  |  |  |  |  |  |  |  |
| PAK5 | A | A |  |  |  |  |  |  |  |  |  |  |  |  |  |  |  |  |  |  |  |  |  |  |
| PAK6 | A | A |  |  |  |  |  |  |  |  |  |  |  |  |  |  |  |  |  |  |  |  |  |  |
| PANX1 | I | I |  |  |  |  |  |  |  |  |  |  |  |  |  |  |  |  |  | I |  |  |  |  |
| PARD3 | K | K |  |  |  |  |  |  |  |  |  |  |  |  |  |  |  |  |  |  |  |  |  |  |
| PDCD1 | A | A |  |  |  |  |  |  |  |  |  |  |  | A |  |  |  |  | A |  |  |  |  |  |
| PDIA3 | U | NK |  |  |  |  |  |  |  |  |  |  |  |  |  |  |  |  | A |  |  |  |  |  |
| PDPK1 | A | A | A |  |  |  |  |  |  |  |  |  |  |  | I | LM | S |  | A | I | S |  | P |  |
| PECAM1 | U | LM |  |  |  |  |  |  |  |  |  |  |  |  |  |  |  |  |  | I |  |  | P | C |
| PF4 | U | K |  |  |  |  |  |  |  |  |  |  |  |  |  |  |  |  |  |  |  |  | P | C |
| PF4V1 | C | K |  |  |  |  |  |  |  |  |  |  |  |  |  |  |  |  |  |  |  |  |  | C |
| PIGR | U | A |  |  |  |  |  |  |  |  |  |  |  |  |  |  |  |  |  | I |  |  |  |  |
| PIK3AP1 | A | A |  |  |  |  |  |  |  |  |  |  |  |  | I |  | S |  | A |  |  |  |  |  |
| PIK3CA | A | A | NK | A | A | A | LM | K | I | I | P |  |  |  |  |  |  |  | A | I | S |  | P | C |
| PIK3CB | A | A | NK | A | A | A | LM | K | I | I | P |  |  |  |  |  |  |  | A | I | S |  | P | C |
| PIK3CD | A | A | NK | A | A | A | LM | K | I | I | P |  |  | A | I | LM |  |  | A |  | S |  |  |  |
| PIK3CG | P | K | P |  |  |  |  |  |  |  |  |  |  |  |  |  |  |  |  |  |  |  | P |  |
| PIK3R1 | A | A | NK | A | A | A | LM | K | I | I | P |  |  |  |  | LM |  |  | A | I | S |  | P | C |
| PIK3R2 | A | A | NK | A | A | A | LM | K | I | I | P |  |  |  |  |  |  |  | A | I | S |  | P | C |
| PIK3R3 | A | A | NK | A | A | A | LM | K | I | I | P |  |  |  |  |  |  |  | A |  | S |  | P |  |
| PIK3R5 | P | K | P |  |  |  |  |  |  |  |  |  |  |  |  |  |  |  |  |  |  |  | P |  |
| PIK3R6 | P | K | P |  |  |  |  |  |  |  |  |  |  |  | I | LM |  |  |  |  |  |  | P |  |
| PIN1 | I | I |  |  |  |  |  |  |  |  |  |  |  |  |  |  |  |  |  | I | S |  |  |  |
| PIP5K1A | A | A |  |  |  |  |  |  |  |  |  |  |  |  |  |  |  |  |  |  |  |  |  |  |
| PIP5K1B | A | A |  |  |  |  |  |  |  |  |  |  |  |  |  |  |  |  |  |  |  |  |  |  |
| PIP5K1C | A | A |  |  |  |  |  |  |  |  |  |  |  |  |  |  |  |  |  |  |  |  |  |  |
| PLA2G4A | A | A | A | P |  |  |  |  |  |  |  |  |  |  |  |  |  |  |  |  |  |  | P |  |
| PLA2G4B | A | A | A | P |  |  |  |  |  |  |  |  |  |  |  |  |  |  |  |  |  |  |  |  |
| PLA2G4C | A | A | P |  |  |  |  |  |  |  |  |  |  |  |  |  |  |  |  |  |  |  |  |  |
| PLA2G4D | A | A | A | P |  |  |  |  |  |  |  |  |  |  |  |  |  |  |  |  |  |  |  |  |
| PLA2G4E | A | A | A | P |  |  |  |  |  |  |  |  |  |  |  |  |  |  |  |  |  |  |  |  |
| PLA2G4F | A | A | A | P |  |  |  |  |  |  |  |  |  |  |  |  |  |  |  |  |  |  |  |  |
| PLA2G6 | U | A |  |  |  |  |  |  |  |  |  |  |  |  |  |  |  |  |  | I |  |  |  |  |
| PLAT | C | C |  |  |  |  |  |  |  |  |  |  |  |  |  |  |  |  |  |  |  |  |  | C |
| PLAU | C | C |  |  |  |  |  |  |  |  |  |  |  |  |  |  |  |  |  | I |  |  |  | C |
| PLAUR | C | C |  |  |  |  |  |  |  |  |  |  |  |  |  |  |  |  |  | I |  |  |  | C |
| PLCB1 | U | K | I | P |  |  |  |  |  |  |  |  |  |  |  |  |  |  |  |  |  |  |  |  |
| PLCB2 | U | K | I | P |  |  |  |  |  |  |  |  |  |  |  |  |  |  |  |  |  |  |  |  |
| PLCB3 | U | K | I | P |  |  |  |  |  |  |  |  |  |  |  |  |  |  |  |  |  |  |  |  |
| PLCB4 | U | K | I | P |  |  |  |  |  |  |  |  |  |  |  |  |  |  |  |  |  |  |  |  |
| PLCG1 | A | A | NK | A | A | A | A | LM |  |  |  |  |  |  |  |  | S |  | A | I | S |  |  | C |
| PLCG2 | A | NK | A | A | A | LM | I | P |  |  |  |  |  |  |  | LM | S |  | A | I |  |  | P |  |
| PLD1 | U | A |  |  |  |  |  |  |  |  |  |  |  |  |  |  |  |  |  | I |  |  |  |  |
| PLD2 | U | A |  |  |  |  |  |  |  |  |  |  |  |  |  | LM |  |  |  | I |  |  |  |  |
| PLG | C | C |  |  |  |  |  |  |  |  |  |  |  |  |  |  |  |  |  |  |  |  | P | C |
| PLK3 | I | I |  |  |  |  |  |  |  |  |  |  |  |  |  |  |  |  |  |  |  |  |  |  |
| PLPP1 | A | A |  |  |  |  |  |  |  |  |  |  |  |  |  |  |  |  |  |  |  |  |  |  |
| PLPP2 | A | A |  |  |  |  |  |  |  |  |  |  |  |  |  |  |  |  |  |  |  |  |  |  |
| PLPP3 | A | A |  |  |  |  |  |  |  |  |  |  |  |  |  |  |  |  |  |  |  |  |  |  |
| POLR1C | I | I |  |  |  |  |  |  |  |  |  |  |  |  |  |  |  |  |  | I |  |  |  |  |
| POLR1D | I | I |  |  |  |  |  |  |  |  |  |  |  |  |  |  |  |  |  | I |  |  |  |  |
| POLR2E | I | I |  |  |  |  |  |  |  |  |  |  |  |  |  |  |  |  |  | I |  |  |  |  |
| POLR2F | I | I |  |  |  |  |  |  |  |  |  |  |  |  |  |  |  |  |  | I |  |  |  |  |
| POLR2H | I | I |  |  |  |  |  |  |  |  |  |  |  |  |  |  |  |  |  | I |  |  |  |  |
| POLR2K | I | I |  |  |  |  |  |  |  |  |  |  |  |  |  |  |  |  |  | I |  |  |  |  |
| POLR2L | I | I |  |  |  |  |  |  |  |  |  |  |  |  |  |  |  |  |  | I |  |  |  |  |
| POLR3A | I | I |  |  |  |  |  |  |  |  |  |  |  |  | I |  |  |  |  | I |  |  |  |  |
| POLR3B | I | I |  |  |  |  |  |  |  |  |  |  |  |  | I |  |  |  |  | I |  |  |  |  |
| POLR3C | I | I |  |  |  |  |  |  |  |  |  |  |  |  | I |  |  |  |  | I |  |  |  |  |
| POLR3D | I | I |  |  |  |  |  |  |  |  |  |  |  |  | I |  |  |  |  | I |  |  |  |  |
| POLR3E | I | I |  |  |  |  |  |  |  |  |  |  |  |  | I |  |  |  |  | I |  |  |  |  |
| POLR3F | I | I |  |  |  |  |  |  |  |  |  |  |  |  | I |  |  |  |  | I |  |  |  |  |
| POLR3G | I | I |  |  |  |  |  |  |  |  |  |  |  |  | I |  |  |  |  | I |  |  |  |  |
| POLR3GL | I | I |  |  |  |  |  |  |  |  |  |  |  |  |  |  |  |  |  | I |  |  |  |  |
| POLR3H | I | I |  |  |  |  |  |  |  |  |  |  |  |  | I |  |  |  |  | I |  |  |  |  |
| POLR3K | I | I |  |  |  |  |  |  |  |  |  |  |  |  | I |  |  |  |  | I |  |  |  |  |
| PPBP | U | K |  |  |  |  |  |  |  |  |  |  |  |  |  |  |  |  |  | I |  |  | P |  |
| PPP1CA | P | P |  |  |  |  |  |  |  |  |  |  |  |  |  |  |  |  |  |  |  |  |  |  |
| PPP1CB | P | P |  |  |  |  |  |  |  |  |  |  |  |  |  |  |  |  |  |  |  |  |  |  |
| PPP1CC | P | P |  |  |  |  |  |  |  |  |  |  |  |  |  |  |  |  |  |  |  |  |  |  |
| PPP1R12A | P | P |  |  |  |  |  |  |  |  |  |  |  |  |  |  |  |  |  |  |  |  |  |  |
| PPP3CA | A | A | NK | A | A | A | I |  |  |  |  |  |  |  |  |  |  |  | A | I |  |  |  |  |
| PPP3CB | A | A | NK | A | A | A | I |  |  |  |  |  |  | A |  | LM |  |  | A | I |  |  |  |  |
| PPP3CC | A | A | NK | A | A | A | I |  |  |  |  |  |  |  |  |  |  |  |  |  |  |  |  |  |
| PPP3R1 | A | A | NK | A | A | A | I |  |  |  |  |  |  |  |  |  |  |  | A | I |  |  |  |  |
| PPP3R2 | A | A | NK | A | A | A | I |  |  |  |  |  |  |  |  |  |  |  |  |  |  |  |  |  |
| PREX1 | LM | K |  |  |  |  |  |  |  |  |  |  |  |  |  | LM |  |  |  |  |  |  |  |  |
| PRF1 | U | NK |  |  |  |  |  |  |  |  |  |  |  | A |  | LM |  |  |  |  |  |  |  |  |
| PRKACA | U | K | P |  |  |  |  |  |  |  |  |  |  |  |  |  |  |  | A | I | S |  |  |  |
| PRKACB | U | K | P |  |  |  |  |  |  |  |  |  |  |  |  |  |  |  | A | I |  |  |  |  |
| PRKACG | U | K | P |  |  |  |  |  |  |  |  |  |  |  |  |  |  |  | A | I |  |  |  |  |
| PRKCA | A | NK | A | A | LM |  |  |  |  |  |  |  |  |  |  |  |  |  |  |  |  |  | P |  |
| PRKCB | A | NK | A | A | LM | K |  |  |  |  |  |  |  | A |  | LM | S |  | A |  |  |  | P |  |
| PRKCD | I | A | K | I | I |  |  |  |  |  |  |  |  | A |  | LM |  |  |  | I | S |  | P |  |
| PRKCE | I | A |  |  |  |  |  |  |  |  |  |  |  |  | I | LM | S |  |  | I |  |  | P |  |
| PRKCG | U | NK | A | LM |  |  |  |  |  |  |  |  |  |  |  |  |  |  |  |  |  |  | P |  |
| PRKCI | P | P |  |  |  |  |  |  |  |  |  |  |  |  |  |  |  |  |  |  |  |  |  |  |
| PRKCQ | A | A | A | A |  |  |  |  |  |  |  |  |  | A |  | LM |  |  | A | I |  |  | P |  |
| PRKCZ | P | K | P |  |  |  |  |  |  |  |  |  |  | A |  | LM |  |  |  |  |  |  | P |  |
| PRKG1 | P | P |  |  |  |  |  |  |  |  |  |  |  |  |  |  |  |  | A |  |  |  | P |  |
| PRKG2 | P | P |  |  |  |  |  |  |  |  |  |  |  |  |  |  |  |  |  |  |  |  | P |  |
| PROC | C | C |  |  |  |  |  |  |  |  |  |  |  |  |  |  |  |  |  |  |  |  |  | C |
| PROCR | C | C |  |  |  |  |  |  |  |  |  |  |  |  |  |  |  |  |  |  |  |  |  | C |
| PROS1 | C | C |  |  |  |  |  |  |  |  |  |  |  |  |  |  |  |  |  | I |  | C | P | C |
| PSME1 | U | APC |  |  |  |  |  |  |  |  |  |  |  |  |  |  |  |  | A | I | S |  |  |  |
| PSME2 | U | APC |  |  |  |  |  |  |  |  |  |  |  |  |  |  |  |  | A | I | S |  |  |  |
| PSME3 | U | APC |  |  |  |  |  |  |  |  |  |  |  |  |  |  |  |  | A | I | S |  |  |  |
| PSTPIP1 | I | I |  |  |  |  |  |  |  |  |  |  |  |  | I |  |  |  |  | I |  |  |  |  |
| PTGIR | P | P |  |  |  |  |  |  |  |  |  |  |  |  |  |  |  |  |  |  |  |  | P |  |
| PTGS1 | P | P |  |  |  |  |  |  |  |  |  |  |  |  |  |  |  |  |  |  |  |  |  |  |
| PTGS2 | I | I |  |  |  |  |  |  |  |  |  |  |  |  |  |  |  |  |  |  | S |  |  |  |
| PTK2 | U | LM | K |  |  |  |  |  |  |  |  |  |  |  |  |  |  |  |  | I | S |  | P |  |
| PTK2B | LM | NK | LM | K |  |  |  |  |  |  |  |  |  | A |  | LM |  |  |  |  | S |  |  |  |
| PTPN11 | I | NK | LM | I |  |  |  |  |  |  |  |  |  |  |  |  |  |  | A | I | S |  | P | C |
| PTPN6 | A | A | NK | A |  |  |  |  |  |  |  |  |  | A | I | LM | S |  | A | I | S |  | P | C |
| PTPRC | A | A | A |  |  |  |  |  |  |  |  |  |  | A |  | LM | S |  | A | I |  |  |  |  |
| PXN | LM | LM | K |  |  |  |  |  |  |  |  |  |  |  |  |  |  |  |  |  |  |  |  |  |
| PYCARD | I | I | I | I |  |  |  |  |  |  |  |  |  | A | I | LM |  |  |  | I |  |  |  |  |
| PYDC1 | I | I |  |  |  |  |  |  |  |  |  |  |  |  |  |  |  |  |  |  |  |  |  |  |
| PYDC2 | I | I |  |  |  |  |  |  |  |  |  |  |  |  |  |  |  |  |  |  |  |  |  |  |
| PYDC5 | I | I |  |  |  |  |  |  |  |  |  |  |  |  |  |  |  |  |  |  |  |  |  |  |
| RAC1 | A | NK | A | A | A | LM | K | I |  |  |  |  |  |  |  |  |  |  | A | I |  |  | P |  |
| RAC2 | A | NK | A | A | A | LM | K |  |  |  |  |  |  |  |  | LM |  |  |  | I |  |  | P |  |
| RAC3 | A | NK | A | A | K |  |  |  |  |  |  |  |  |  |  |  |  |  |  |  |  |  |  |  |
| RAET1E | A | NK |  |  |  |  |  |  |  |  |  |  |  | A |  |  |  |  | A |  |  |  |  |  |
| RAET1G | NK | NK |  |  |  |  |  |  |  |  |  |  |  |  |  |  |  |  |  |  |  |  |  |  |
| RAET1L | NK | NK |  |  |  |  |  |  |  |  |  |  |  |  |  |  |  |  |  |  |  |  |  |  |
| RAF1 | A | A | NK | A | A | A | K | I |  |  |  |  |  |  |  |  |  |  | A | I | S |  | P |  |
| RAP1A | P | LM | K | P |  |  |  |  |  |  |  |  |  |  |  |  | S |  | A | I | S |  | P |  |
| RAP1B | P | LM | K | P |  |  |  |  |  |  |  |  |  |  |  |  |  |  | A | I | S |  | P |  |
| RAPGEF3 | U | LM |  |  |  |  |  |  |  |  |  |  |  |  |  |  |  |  | A |  |  |  | P |  |
| RAPGEF4 | U | LM |  |  |  |  |  |  |  |  |  |  |  |  |  |  |  |  | A |  |  |  | P |  |
| RARA | U | A |  |  |  |  |  |  |  |  |  |  |  |  |  | LM |  |  |  |  |  |  |  |  |
| RASGRP1 | U | A | P |  |  |  |  |  |  |  |  |  |  |  | I | LM |  |  | A | I | S |  | P |  |
| RASGRP2 | P | K | P |  |  |  |  |  |  |  |  |  |  |  |  |  |  |  | A | I |  |  | P |  |
| RASGRP3 | A | A |  |  |  |  |  |  |  |  |  |  |  |  |  |  |  |  | A |  | S |  |  |  |
| RASSF5 | LM | LM |  |  |  |  |  |  |  |  |  |  |  |  |  |  |  |  |  |  |  |  |  |  |
| RBCK1 | U | I |  |  |  |  |  |  |  |  |  |  |  |  |  |  | S |  | A |  |  |  |  |  |
| RBPJ | U | A |  |  |  |  |  |  |  |  |  |  |  |  |  | LM |  |  |  |  |  |  |  |  |
| RBPJL | A | A |  |  |  |  |  |  |  |  |  |  |  |  |  |  |  |  |  |  |  |  |  |  |
| RELA | I | A | A | A | A | K | I | I | I | I | I |  |  |  | I |  | S |  | A | I | S |  |  |  |
| RELB | I | I |  |  |  |  |  |  |  |  |  |  |  | A | I | LM |  |  |  | I | S |  |  |  |
| RFX5 | NK | NK |  |  |  |  |  |  |  |  |  |  |  |  |  |  |  |  |  |  |  |  |  |  |
| RFXANK | NK | NK |  |  |  |  |  |  |  |  |  |  |  |  |  |  |  |  |  |  |  |  |  |  |
| RFXAP | NK | NK |  |  |  |  |  |  |  |  |  |  |  |  |  |  |  |  |  |  |  |  |  |  |
| RHOA | I | A | LM | K | I | I | P |  |  |  |  |  |  |  |  | LM |  |  |  | I |  |  | P |  |
| RHOH | LM | LM |  |  |  |  |  |  |  |  |  |  |  |  |  | LM |  |  |  |  |  |  |  |  |
| RIPK1 | I | I | I | I | I |  |  |  |  |  |  |  |  |  |  |  |  |  |  | I |  |  |  |  |
| RIPK2 | I | I |  |  |  |  |  |  |  |  |  |  |  | A | I | LM | S |  | A | I | S |  |  |  |
| RIPK3 | I | I | I |  |  |  |  |  |  |  |  |  |  | A |  | LM |  |  |  | I |  |  |  |  |
| RNASEL | I | I |  |  |  |  |  |  |  |  |  |  |  |  |  |  |  |  |  |  | S |  |  |  |
| RNF125 | I | I |  |  |  |  |  |  |  |  |  |  |  | A |  |  |  |  |  | I |  |  |  |  |
| RNF31 | I | I |  |  |  |  |  |  |  |  |  |  |  |  |  |  | S |  |  |  |  |  |  |  |
| ROCK1 | U | LM | K | P |  |  |  |  |  |  |  |  |  |  |  |  |  |  |  | I |  |  |  |  |
| ROCK2 | U | LM | K | P |  |  |  |  |  |  |  |  |  |  |  |  |  |  |  |  |  |  |  |  |
| RORA | A | A |  |  |  |  |  |  |  |  |  |  |  | A |  | LM |  |  |  |  | S |  |  |  |
| RORC | A | A |  |  |  |  |  |  |  |  |  |  |  | A |  | LM |  |  |  |  | S |  |  |  |
| RPS6KB1 | U | A |  |  |  |  |  |  |  |  |  |  |  |  | I |  |  |  |  |  |  |  |  |  |
| RPS6KB2 | A | A |  |  |  |  |  |  |  |  |  |  |  |  |  |  |  |  |  |  |  |  |  |  |
| RRAS | I | I |  |  |  |  |  |  |  |  |  |  |  |  |  |  |  |  |  |  |  |  |  |  |
| RRAS2 | I | I |  |  |  |  |  |  |  |  |  |  |  |  |  |  |  |  |  |  |  |  |  |  |
| RUNX1 | U | A |  |  |  |  |  |  |  |  |  |  |  |  |  | LM |  |  |  |  |  |  |  |  |
| RUNX3 | U | A |  |  |  |  |  |  |  |  |  |  |  |  |  | LM |  |  |  |  |  |  |  |  |
| RXRA | A | A |  |  |  |  |  |  |  |  |  |  |  |  |  |  |  |  |  |  |  |  |  |  |
| RXRB | A | A |  |  |  |  |  |  |  |  |  |  |  |  |  |  |  |  |  |  |  |  |  |  |
| RXRG | A | A |  |  |  |  |  |  |  |  |  |  |  |  |  |  |  |  |  |  |  |  |  |  |
| SCIN | A | A |  |  |  |  |  |  |  |  |  |  |  |  |  |  |  |  |  |  |  |  |  |  |
| SERPINA1 | U | C |  |  |  |  |  |  |  |  |  |  |  |  |  |  |  |  |  | I |  |  | P |  |
| SERPINA5 | C | C |  |  |  |  |  |  |  |  |  |  |  |  |  |  |  |  |  |  |  |  |  | C |
| SERPINB2 | C | C |  |  |  |  |  |  |  |  |  |  |  |  |  |  |  |  |  |  | S |  |  | C |
| SERPINC1 | C | C |  |  |  |  |  |  |  |  |  |  |  |  |  |  |  |  |  |  |  |  |  | C |
| SERPIND1 | C | C |  |  |  |  |  |  |  |  |  |  |  |  |  |  |  |  |  |  |  |  |  | C |
| SERPINE1 | C | C |  |  |  |  |  |  |  |  |  |  |  |  |  |  |  |  |  |  |  |  | P | C |
| SERPINF2 | C | C |  |  |  |  |  |  |  |  |  |  |  |  |  |  |  |  |  |  |  |  | P | C |
| SERPING1 | C | C |  |  |  |  |  |  |  |  |  |  |  | A | I |  |  | C |  | I |  | C | P | C |
| SH2D1A | A | NK |  |  |  |  |  |  |  |  |  |  |  | A | I |  |  |  | A |  |  |  |  |  |
| SH2D1B | U | NK |  |  |  |  |  |  |  |  |  |  |  |  |  |  |  |  | A |  |  |  |  |  |
| SH3BP2 | NK | NK |  |  |  |  |  |  |  |  |  |  |  |  |  |  |  |  |  |  |  |  |  |  |
| SHARPIN | I | I |  |  |  |  |  |  |  |  |  |  |  |  |  |  |  |  |  |  |  |  |  |  |
| SHC1 | U | NK | K |  |  |  |  |  |  |  |  |  |  |  |  |  |  |  |  | I | S |  | P | C |
| SHC2 | U | NK | K |  |  |  |  |  |  |  |  |  |  |  |  |  |  |  |  |  | S |  |  |  |
| SHC3 | U | NK | K |  |  |  |  |  |  |  |  |  |  |  |  |  |  |  |  |  | S |  |  |  |
| SHC4 | NK | NK | K |  |  |  |  |  |  |  |  |  |  |  |  |  |  |  |  |  |  |  |  |  |
| SIKE1 | I | I |  |  |  |  |  |  |  |  |  |  |  |  |  |  |  |  |  | I |  |  |  |  |
| SIPA1 | U | LM |  |  |  |  |  |  |  |  |  |  |  |  |  |  |  |  | A |  |  |  |  |  |
| SMAD2 | A | A |  |  |  |  |  |  |  |  |  |  |  |  |  |  |  |  |  |  |  |  |  |  |
| SMAD3 | U | A |  |  |  |  |  |  |  |  |  |  |  |  |  | LM |  |  |  |  | S |  |  |  |
| SMAD4 | A | A |  |  |  |  |  |  |  |  |  |  |  |  |  |  |  |  |  |  |  |  |  |  |
| SNAP23 | U | P |  |  |  |  |  |  |  |  |  |  |  |  |  | LM |  |  | A | I |  |  |  |  |
| SOS1 | A | A | NK | A | A | K |  |  |  |  |  |  |  |  |  | LM |  |  | A | I | S |  | P | C |
| SOS2 | A | A | NK | A | A | K |  |  |  |  |  |  |  |  |  | LM |  |  |  |  | S |  |  |  |
| SPHK1 | U | A |  |  |  |  |  |  |  |  |  |  |  |  |  | LM |  |  |  |  |  |  |  |  |
| SPHK2 | U | A |  |  |  |  |  |  |  |  |  |  |  |  |  | LM |  |  |  |  |  |  |  |  |
| SPP1 | I | I |  |  |  |  |  |  |  |  |  |  |  |  |  |  |  |  |  |  |  |  |  |  |
| SRC | I | K | I | P |  |  |  |  |  |  |  |  |  |  |  |  |  |  | A | I | S |  | P | C |
| STAT1 | I | A | A | K | I | I | I |  |  |  |  |  |  |  | I |  |  |  |  |  | S |  |  |  |
| STAT2 | I | K | I | I |  |  |  |  |  |  |  |  |  |  | I |  |  |  |  |  | S |  |  |  |
| STAT3 | A | A | K |  |  |  |  |  |  |  |  |  |  | A |  | LM |  |  |  |  | S |  |  |  |
| STAT4 | A | A |  |  |  |  |  |  |  |  |  |  |  |  |  |  |  |  |  |  | S |  |  |  |
| STAT5A | A | A | A |  |  |  |  |  |  |  |  |  |  |  | I | LM |  |  |  |  | S |  |  |  |
| STAT5B | A | A | A | K |  |  |  |  |  |  |  |  |  |  | I | LM |  |  |  |  | S |  |  |  |
| STAT6 | A | A | A |  |  |  |  |  |  |  |  |  |  | A |  | LM |  |  |  | I | S |  |  |  |
| STIM1 | P | P |  |  |  |  |  |  |  |  |  |  |  |  |  |  |  |  | A |  |  |  | P |  |
| SUGT1 | I | I |  |  |  |  |  |  |  |  |  |  |  |  |  |  |  |  |  | I |  |  |  |  |
| SYK | A | NK | A | A | A | I | P |  |  |  |  |  |  | A | I | LM | S |  | A | I | S |  | P |  |
| TAB1 | I | I | I |  |  |  |  |  |  |  |  |  |  |  |  |  |  |  |  | I | S |  |  |  |
| TAB2 | I | I | I |  |  |  |  |  |  |  |  |  |  |  |  |  |  |  | A | I | S |  |  |  |
| TAB3 | I | I |  |  |  |  |  |  |  |  |  |  |  |  |  |  |  |  |  | I | S |  |  |  |
| TANK | I | I | I |  |  |  |  |  |  |  |  |  |  |  |  |  |  |  |  | I |  |  |  |  |
| TAP1 | A | NK |  |  |  |  |  |  |  |  |  |  |  | A | I |  |  |  | A |  |  |  |  |  |
| TAP2 | A | NK |  |  |  |  |  |  |  |  |  |  |  | A | I |  |  |  | A |  |  |  |  |  |
| TAPBP | U | NK |  |  |  |  |  |  |  |  |  |  |  |  |  |  |  |  | A |  |  |  |  |  |
| TBK1 | I | I | I | I | I |  |  |  |  |  |  |  |  |  | I | LM |  |  |  | I | S |  |  |  |
| TBKBP1 | I | I |  |  |  |  |  |  |  |  |  |  |  |  | I |  |  |  |  |  |  |  |  |  |
| TBX21 | A | A | A |  |  |  |  |  |  |  |  |  |  | A |  | LM |  |  |  |  |  |  |  |  |
| TBXA2R | P | P |  |  |  |  |  |  |  |  |  |  |  |  |  |  |  |  |  |  |  |  | P |  |
| TBXAS1 | P | P |  |  |  |  |  |  |  |  |  |  |  |  |  |  |  |  |  |  |  |  |  |  |
| TEC | A | A |  |  |  |  |  |  |  |  |  |  |  | A |  |  | S |  |  | I | S |  |  |  |
| TFPI | C | C |  |  |  |  |  |  |  |  |  |  |  |  |  |  |  |  |  |  |  |  |  | C |
| TFRC | A | H |  |  |  |  |  |  |  |  |  |  |  | A |  |  |  |  |  |  |  |  |  |  |
| TGFB1 | A | A | A |  |  |  |  |  |  |  |  |  |  | A | I |  |  |  |  |  | S |  | P | C |
| TGFBR1 | A | A |  |  |  |  |  |  |  |  |  |  |  |  |  |  |  |  |  |  |  |  |  |  |
| TGFBR2 | A | A |  |  |  |  |  |  |  |  |  |  |  |  |  |  |  |  |  |  |  |  |  |  |
| THBD | C | C |  |  |  |  |  |  |  |  |  |  |  |  |  |  |  |  |  |  |  |  |  | C |
| THPO | P | H |  |  |  |  |  |  |  |  |  |  |  |  |  |  |  |  |  |  |  |  | P |  |
| THY1 | LM | LM |  |  |  |  |  |  |  |  |  |  |  |  |  |  | S |  |  |  |  |  |  |  |
| TIAM1 | K | K |  |  |  |  |  |  |  |  |  |  |  |  |  |  |  |  |  |  |  |  |  |  |
| TICAM1 | I | I | I |  |  |  |  |  |  |  |  |  |  |  | I |  | S |  |  | I |  |  |  |  |
| TICAM2 | I | I |  |  |  |  |  |  |  |  |  |  |  |  | I |  | S |  |  | I |  |  |  |  |
| TIRAP | I | I |  |  |  |  |  |  |  |  |  |  |  |  | I |  | S |  | A | I |  |  |  |  |
| TKFC | I | I |  |  |  |  |  |  |  |  |  |  |  |  | I |  | S |  |  | I |  |  |  |  |
| TLN1 | P | P |  |  |  |  |  |  |  |  |  |  |  |  |  |  |  |  |  |  | S |  | P |  |
| TLN2 | P | P |  |  |  |  |  |  |  |  |  |  |  |  |  |  |  |  |  |  |  |  |  |  |
| TLR1 | I | I |  |  |  |  |  |  |  |  |  |  |  |  | I |  | S |  | A | I |  |  |  |  |
| TLR2 | I | I |  |  |  |  |  |  |  |  |  |  |  |  | I |  | S |  | A | I |  |  |  |  |
| TLR3 | I | I |  |  |  |  |  |  |  |  |  |  |  |  | I |  | S |  |  | I |  |  |  |  |
| TLR4 | I | I | I |  |  |  |  |  |  |  |  |  |  |  | I |  | S |  | A | I |  |  |  |  |
| TLR5 | I | I |  |  |  |  |  |  |  |  |  |  |  |  | I |  | S |  |  | I |  |  |  |  |
| TLR6 | I | I |  |  |  |  |  |  |  |  |  |  |  |  | I |  | S |  | A | I |  |  |  |  |
| TLR7 | I | I |  |  |  |  |  |  |  |  |  |  |  |  | I |  | S |  |  | I |  |  |  |  |
| TLR8 | I | I |  |  |  |  |  |  |  |  |  |  |  |  | I |  | S |  |  | I |  |  |  |  |
| TLR9 | I | I |  |  |  |  |  |  |  |  |  |  |  |  | I |  | S |  |  | I | S |  |  |  |
| TMEM173 | I | I | I | I |  |  |  |  |  |  |  |  |  |  |  |  |  |  |  | I |  |  |  |  |
| TNF | I | APC | APC | NK | A | I | I | I | I | H |  |  |  | A | I |  | S |  |  |  | S |  |  |  |
| TNFAIP3 | I | I |  |  |  |  |  |  |  |  |  |  |  | A | I |  | S |  |  | I |  |  |  |  |
| TNFRSF10A | NK | NK |  |  |  |  |  |  |  |  |  |  |  |  |  |  |  |  |  |  |  |  |  | C |
| TNFRSF10B | NK | NK |  |  |  |  |  |  |  |  |  |  |  |  |  |  |  |  |  |  |  |  |  | C |
| TNFRSF13B | A | A |  |  |  |  |  |  |  |  |  |  |  | A |  |  |  |  |  |  | S |  |  |  |
| TNFRSF13C | A | A |  |  |  |  |  |  |  |  |  |  |  | A |  |  |  |  |  |  | S |  |  |  |
| TNFRSF17 | A | A |  |  |  |  |  |  |  |  |  |  |  | A |  |  |  |  |  |  | S |  |  |  |
| TNFSF10 | NK | NK |  |  |  |  |  |  |  |  |  |  |  |  |  |  |  |  |  |  |  |  |  |  |
| TNFSF13 | A | A |  |  |  |  |  |  |  |  |  |  |  | A |  |  |  |  |  |  | S |  |  |  |
| TNFSF13B | A | A |  |  |  |  |  |  |  |  |  |  |  | A |  |  |  |  |  |  | S |  |  |  |
| TOLLIP | I | I |  |  |  |  |  |  |  |  |  |  |  |  | I |  |  |  |  | I | S |  |  |  |
| TP53BP1 | I | I |  |  |  |  |  |  |  |  |  |  |  |  |  |  |  |  |  |  |  |  |  |  |
| TRADD | I | I |  |  |  |  |  |  |  |  |  |  |  |  |  |  |  |  |  |  |  |  |  |  |
| TRAF2 | I | I | I |  |  |  |  |  |  |  |  |  |  | A |  |  |  |  |  | I | S |  |  |  |
| TRAF3 | I | I | I | I |  |  |  |  |  |  |  |  |  |  | I |  | S |  |  | I | S |  |  |  |
| TRAF5 | I | I |  |  |  |  |  |  |  |  |  |  |  |  |  |  |  |  |  |  |  |  |  |  |
| TRAF6 | I | I | I | I |  |  |  |  |  |  |  |  |  | A |  |  | S |  | A | I | S |  |  |  |
| TREX1 | I | I |  |  |  |  |  |  |  |  |  |  |  | A | I |  |  |  |  | I |  |  |  |  |
| TRIM25 | I | I |  |  |  |  |  |  |  |  |  |  |  |  | I |  |  |  |  | I | S |  |  |  |
| TRIP6 | I | I |  |  |  |  |  |  |  |  |  |  |  |  |  |  |  |  |  |  |  |  |  |  |
| TRPM2 | I | I |  |  |  |  |  |  |  |  |  |  |  |  |  |  |  |  |  | I |  |  |  |  |
| TRPM7 | I | I |  |  |  |  |  |  |  |  |  |  |  |  |  |  |  |  |  |  |  |  |  |  |
| TRPV2 | I | I |  |  |  |  |  |  |  |  |  |  |  |  |  |  |  |  |  |  |  |  |  |  |
| TXK | I | LM |  |  |  |  |  |  |  |  |  |  |  | A | I |  | S |  |  | I |  |  |  |  |
| TXN | I | I |  |  |  |  |  |  |  |  |  |  |  |  |  |  |  |  |  | I |  |  |  |  |
| TXN2 | I | I |  |  |  |  |  |  |  |  |  |  |  |  |  |  |  |  |  |  |  |  |  |  |
| TXNIP | I | I |  |  |  |  |  |  |  |  |  |  |  |  |  |  |  |  |  | I |  |  |  |  |
| TYK2 | A | A | A | I |  |  |  |  |  |  |  |  |  |  |  |  |  |  |  |  | S |  |  |  |
| TYROBP | U | NK |  |  |  |  |  |  |  |  |  |  |  |  |  |  |  |  | A | I |  |  |  |  |
| ULBP1 | U | NK |  |  |  |  |  |  |  |  |  |  |  |  | I |  |  |  | A |  |  |  |  |  |
| ULBP2 | NK | NK |  |  |  |  |  |  |  |  |  |  |  |  |  |  |  |  |  |  |  |  |  |  |
| ULBP3 | U | NK |  |  |  |  |  |  |  |  |  |  |  |  |  |  |  |  | A |  |  |  |  |  |
| VAMP8 | I | P |  |  |  |  |  |  |  |  |  |  |  |  | I |  |  |  | A | I |  |  |  |  |
| VASP | A | A | LM | P |  |  |  |  |  |  |  |  |  |  |  |  |  |  | A |  |  |  |  |  |
| VAV1 | A | A | NK | A | A | A | LM | K |  |  |  |  |  |  | I |  |  |  | A | I | S |  | P |  |
| VAV2 | A | A | NK | A | A | A | LM | K |  |  |  |  |  |  |  |  |  |  |  | I |  |  | P |  |
| VAV3 | A | A | NK | A | A | A | LM | K |  |  |  |  |  |  |  |  | S |  |  | I |  |  | P |  |
| VCAM1 | U | LM |  |  |  |  |  |  |  |  |  |  |  |  |  |  |  |  | A |  | S |  |  |  |
| VCL | U | LM |  |  |  |  |  |  |  |  |  |  |  |  |  |  |  |  |  | I | S |  | P |  |
| VDAC1 | I | I |  |  |  |  |  |  |  |  |  |  |  |  |  |  |  |  |  |  |  |  |  |  |
| VDAC2 | I | I |  |  |  |  |  |  |  |  |  |  |  |  |  |  |  |  |  |  |  |  |  |  |
| VDAC3 | I | I |  |  |  |  |  |  |  |  |  |  |  |  |  |  |  |  |  |  |  |  |  |  |
| VSIG4 | C | C |  |  |  |  |  |  |  |  |  |  |  |  | I |  |  | C |  |  |  |  |  |  |
| VTN | C | C |  |  |  |  |  |  |  |  |  |  |  |  |  |  |  |  |  | I |  | C |  |  |
| VWF | C | P | C |  |  |  |  |  |  |  |  |  |  |  |  |  |  |  |  |  | S |  | P | C |
| WAS | A | A | K |  |  |  |  |  |  |  |  |  |  | A | I |  |  |  | A | I |  |  |  |  |
| WASF1 | U | A |  |  |  |  |  |  |  |  |  |  |  |  |  |  |  |  |  | I |  |  |  |  |
| WASF2 | U | A |  |  |  |  |  |  |  |  |  |  |  |  |  |  |  |  |  | I |  |  |  |  |
| WASF3 | U | A |  |  |  |  |  |  |  |  |  |  |  |  |  |  |  |  |  | I |  |  |  |  |
| WASL | U | A | K |  |  |  |  |  |  |  |  |  |  |  |  |  |  |  |  | I |  |  |  |  |
| XCL1 | A | K |  |  |  |  |  |  |  |  |  |  |  | A | I |  |  |  |  |  |  |  |  |  |
| XCL2 | K | K |  |  |  |  |  |  |  |  |  |  |  |  |  |  |  |  |  |  |  |  |  |  |
| XCR1 | K | K |  |  |  |  |  |  |  |  |  |  |  |  |  |  |  |  |  |  |  |  |  |  |
| XIAP | I | I |  |  |  |  |  |  |  |  |  |  |  |  |  |  |  |  |  |  |  |  |  |  |
| ZAP70 | A | A | NK | A | A |  |  |  |  |  |  |  |  | A |  |  | S |  | A |  |  |  |  |  |
| ZBP1 | I | I |  |  |  |  |  |  |  |  |  |  |  |  | I |  |  |  |  |  |  |  |  |  |

**Supplementary Table S4: Gene set enrichment data and pathway analysis of 6 datasets**

| **GSE54514** | **p.geomean** | **stat.mean** | **p.val** | **q.val** | **set.**  **size** |
| --- | --- | --- | --- | --- | --- |
|  | none | none | none | none | none |
| **GSE57065** | **p.geomean** | **stat.mean** | **p.val** | **q.val** | **set.**  **size** |
| hsa04610 Complement and coagulation cascades | 0.006323695 | 2.557470086 | 2.86E-34 | 3.61E-32 | 22 |
| hsa03320 PPAR signaling pathway | 0.009484718 | 2.423348559 | 1.51E-31 | 9.49E-30 | 23 |
| hsa04512 ECM-receptor interaction | 0.033066077 | 1.752947614 | 5.78E-18 | 2.43E-16 | 32 |
| hsa00190 Oxidative phosphorylation | 0.033995879 | 1.648845137 | 2.61E-16 | 8.22E-15 | 55 |
| hsa04810 Regulation of actin cytoskeleton | 0.04412825 | 1.619092984 | 4.58E-16 | 1.16E-14 | 87 |
| hsa00480 Glutathione metabolism | 0.073983063 | 1.440816773 | 9.07E-13 | 1.90E-11 | 22 |
| hsa00980 Metabolism of xenobiotics by cytochrome P450 | 0.09832949 | 1.261096653 | 4.55E-10 | 8.19E-09 | 17 |
| hsa04510 Focal adhesion | 0.094310615 | 1.219373069 | 6.82E-10 | 1.07E-08 | 77 |
| hsa04115 p53 signaling pathway | 0.105763393 | 1.185104354 | 2.45E-09 | 3.43E-08 | 35 |
| hsa00564 Glycerophospholipid metabolism | 0.110800687 | 1.178702969 | 2.82E-09 | 3.55E-08 | 36 |
| hsa04270 Vascular smooth muscle contraction | 0.112105143 | 1.158940797 | 4.64E-09 | 5.31E-08 | 46 |
| hsa04110 Cell cycle | 0.084875981 | 1.156511344 | 5.05E-09 | 5.31E-08 | 73 |
| hsa04114 Oocyte meiosis | 0.106659615 | 1.148173946 | 6.18E-09 | 5.99E-08 | 57 |
| hsa04621 NOD-like receptor signaling pathway | 0.117444921 | 1.131738888 | 1.34E-08 | 1.21E-07 | 24 |
| hsa04666 Fc gamma R-mediated phagocytosis | 0.120512095 | 1.079777444 | 4.43E-08 | 3.72E-07 | 48 |
| hsa00760 Nicotinate and nicotinamide metabolism | 0.135047197 | 1.093036465 | 6.12E-08 | 4.82E-07 | 12 |
| hsa04142 Lysosome | 0.122132829 | 1.062334826 | 7.07E-08 | 5.24E-07 | 51 |
| hsa03050 Proteasome | 0.104879832 | 1.077954357 | 9.06E-08 | 6.34E-07 | 20 |
| hsa04975 Fat digestion and absorption | 0.139709561 | 1.076401023 | 1.01E-07 | 6.67E-07 | 11 |
| hsa00982 Drug metabolism - cytochrome P450 | 0.132741497 | 1.047449477 | 1.63E-07 | 1.03E-06 | 18 |
| hsa00512 Mucin type O-Glycan biosynthesis | 0.145104296 | 1.03887001 | 1.86E-07 | 1.12E-06 | 16 |
| hsa00983 Drug metabolism - other enzymes | 0.153295604 | 1.017635843 | 3.37E-07 | 1.93E-06 | 14 |
| hsa00600 Sphingolipid metabolism | 0.145321237 | 0.996496531 | 5.46E-07 | 2.99E-06 | 18 |
| hsa04914 Progesterone-mediated oocyte maturation | 0.155146284 | 0.963241312 | 8.63E-07 | 4.53E-06 | 48 |
| hsa04130 SNARE interactions in vesicular transport | 0.157969674 | 0.927354124 | 3.10E-06 | 1.56E-05 | 19 |
| hsa04620 Toll-like receptor signaling pathway | 0.154301705 | 0.906496796 | 3.63E-06 | 1.76E-05 | 40 |
| hsa00010 Glycolysis / Gluconeogenesis | 0.176676377 | 0.882224723 | 6.30E-06 | 2.94E-05 | 32 |
| hsa04380 Osteoclast differentiation | 0.174794935 | 0.837186175 | 1.57E-05 | 7.08E-05 | 63 |
| hsa04540 Gap junction | 0.190927257 | 0.822060927 | 2.33E-05 | 0.000101035 | 33 |
| hsa04910 Insulin signaling pathway | 0.190240471 | 0.814558066 | 2.54E-05 | 0.000106635 | 61 |
| hsa04740 Olfactory transduction | 0.181676931 | 0.795203478 | 4.80E-05 | 0.000194961 | 21 |
| hsa04920 Adipocytokine signaling pathway | 0.208744286 | 0.77741916 | 5.86E-05 | 0.000230558 | 30 |
| hsa00260 Glycine, serine and threonine metabolism | 0.186618432 | 0.793688228 | 6.11E-05 | 0.000233218 | 13 |
| hsa00565 Ether lipid metabolism | 0.210741096 | 0.772445822 | 7.57E-05 | 0.000280648 | 15 |
| hsa04730 Long-term depression | 0.229508887 | 0.71919779 | 0.000189212 | 0.000681163 | 22 |
| hsa04145 Phagosome | 0.201873607 | 0.700067041 | 0.000250791 | 0.00087777 | 66 |
| hsa04912 GnRH signaling pathway | 0.235485716 | 0.692392594 | 0.000290031 | 0.000987672 | 38 |
| hsa04020 Calcium signaling pathway | 0.226863057 | 0.665419209 | 0.00046548 | 0.001543434 | 60 |
| hsa04622 RIG-I-like receptor signaling pathway | 0.226783165 | 0.66978891 | 0.000496501 | 0.00160408 | 19 |
| hsa04260 Cardiac muscle contraction | 0.236662851 | 0.664122781 | 0.0005113 | 0.001610595 | 26 |
| hsa00052 Galactose metabolism | 0.241525952 | 0.658613474 | 0.000603231 | 0.001853831 | 16 |
| hsa04141 Protein processing in endoplasmic reticulum | 0.21008418 | 0.64753844 | 0.000641139 | 0.001923416 | 73 |
| hsa04976 Bile secretion | 0.22367835 | 0.613242698 | 0.001270444 | 0.003722698 | 24 |
| hsa00520 Amino sugar and nucleotide sugar metabolism | 0.238195543 | 0.606883483 | 0.001355407 | 0.003881393 | 26 |
| hsa04966 Collecting duct acid secretion | 0.264173444 | 0.607748261 | 0.001447386 | 0.004052679 | 12 |
| hsa00051 Fructose and mannose metabolism | 0.275016633 | 0.556020365 | 0.003126938 | 0.008565092 | 15 |
| hsa04370 VEGF signaling pathway | 0.276809143 | 0.535029288 | 0.003947382 | 0.010582342 | 35 |
| hsa04144 Endocytosis | 0.269651939 | 0.495980721 | 0.006748619 | 0.017715124 | 83 |
| hsa04972 Pancreatic secretion | 0.297339047 | 0.483228191 | 0.008221347 | 0.021140605 | 32 |
| hsa00240 Pyrimidine metabolism | 0.284675211 | 0.466165507 | 0.010305233 | 0.025969188 | 41 |
| hsa00380 Tryptophan metabolism | 0.298913997 | 0.450467062 | 0.013536764 | 0.033443769 | 14 |
| hsa04120 Ubiquitin mediated proteolysis | 0.266374455 | 0.430095275 | 0.016161947 | 0.039116059 | 67 |
| hsa00500 Starch and sucrose metabolism | 0.242862861 | 0.438474035 | 0.016453581 | 0.039116059 | 22 |
| hsa00330 Arginine and proline metabolism | 0.307542608 | 0.413545829 | 0.020406246 | 0.047614574 | 25 |
| hsa04623 Cytosolic DNA-sensing pathway | 0.306670012 | 0.411779465 | 0.021530656 | 0.049324777 | 16 |
| hsa04670 Leukocyte transendothelial migration | 0.316107805 | 0.397233084 | 0.024100342 | 0.05422577 | 44 |
| hsa00030 Pentose phosphate pathway | 0.333170004 | 0.372203862 | 0.034033496 | 0.075231938 | 13 |
| hsa00590 Arachidonic acid metabolism | 0.323238716 | 0.351848932 | 0.040796998 | 0.08862796 | 23 |
| hsa03440 Homologous recombination | 0.335745663 | 0.353219158 | 0.041536054 | 0.088704116 | 14 |
| hsa04664 Fc epsilon RI signaling pathway | 0.337001288 | 0.346755923 | 0.04244902 | 0.089142941 | 37 |
| hsa04146 Peroxisome | 0.334309761 | 0.331725449 | 0.049778762 | 0.102821704 | 38 |
| hsa04010 MAPK signaling pathway | 0.349075014 | 0.325789019 | 0.051986252 | 0.10564948 | 115 |
| hsa00534 Glycosaminoglycan biosynthesis - heparan sulfate | 0.350372309 | 0.317377344 | 0.059747911 | 0.119495823 | 13 |
| hsa04630 Jak-STAT signaling pathway | 0.337957504 | 0.307136844 | 0.063172461 | 0.124370783 | 58 |
| hsa00670 One carbon pool by folate | 0.355395341 | 0.287184159 | 0.079626267 | 0.154352456 | 12 |
| hsa04210 Apoptosis | 0.449418067 | 0.056735492 | 0.388846053 | 0.739940075 | 46 |
| hsa04722 Neurotrophin signaling pathway | 0.431430029 | 0.054288695 | 0.393460199 | 0.739940075 | 57 |
| hsa03022 Basal transcription factors | 0.451147091 | 0.033928731 | 0.432168589 | 0.800782974 | 18 |
| hsa03060 Protein export | 0.425263488 | 0.024222386 | 0.449584292 | 0.820980012 | 10 |
| hsa04012 ErbB signaling pathway | 0.462441166 | 0.017319915 | 0.465410076 | 0.837738138 | 35 |
| hsa02010 ABC transporters | 0.459138946 | 0.002992497 | 0.495453384 | 0.879255301 | 14 |
| hsa00071 Fatty acid metabolism | 0.482006474 | -0.012001475 | 0.523663412 | 0.916410972 | 17 |
| hsa04530 Tight junction | 0.489833858 | -0.019501103 | 0.538595435 | 0.929630477 | 44 |
| hsa04360 Axon guidance | 0.460183028 | -0.041949749 | 0.582766049 | 0.992277327 | 47 |
| hsa04974 Protein digestion and absorption | 0.443277041 | -0.055794664 | 0.607741176 | 1 | 22 |
| hsa00350 Tyrosine metabolism | 0.47591606 | -0.09133216 | 0.673147094 | 1 | 18 |
| hsa00230 Purine metabolism | 0.508506455 | -0.122534621 | 0.729318147 | 1 | 68 |
| hsa04962 Vasopressin-regulated water reabsorption | 0.538837605 | -0.160162354 | 0.785621423 | 1 | 17 |
| hsa04720 Long-term potentiation | 0.551346292 | -0.180977424 | 0.815553104 | 1 | 27 |
| hsa00514 Other types of O-glycan biosynthesis | 0.555359733 | -0.213419928 | 0.852991089 | 1 | 14 |
| hsa00561 Glycerolipid metabolism | 0.554455185 | -0.21241531 | 0.853152495 | 1 | 17 |
| hsa04971 Gastric acid secretion | 0.560430398 | -0.226702517 | 0.869182106 | 1 | 22 |
| hsa04150 mTOR signaling pathway | 0.56730353 | -0.234823538 | 0.877524828 | 1 | 21 |
| hsa04742 Taste transduction | 0.553295103 | -0.256586374 | 0.893726374 | 1 | 10 |
| hsa04970 Salivary secretion | 0.557646711 | -0.253868279 | 0.89585237 | 1 | 31 |
| hsa00830 Retinol metabolism | 0.576146507 | -0.281975685 | 0.917646471 | 1 | 16 |
| hsa00640 Propanoate metabolism | 0.588731209 | -0.284453623 | 0.920097185 | 1 | 18 |
| hsa00340 Histidine metabolism | 0.556999019 | -0.310346216 | 0.934645239 | 1 | 13 |
| hsa00620 Pyruvate metabolism | 0.569929301 | -0.310559692 | 0.937656104 | 1 | 23 |
| hsa03420 Nucleotide excision repair | 0.564507152 | -0.323576625 | 0.944667413 | 1 | 25 |
| hsa04350 TGF-beta signaling pathway | 0.603247234 | -0.336026743 | 0.95255432 | 1 | 35 |
| hsa04520 Adherens junction | 0.606326925 | -0.373886164 | 0.967849527 | 1 | 24 |
| hsa04330 Notch signaling pathway | 0.59429091 | -0.379344691 | 0.969324297 | 1 | 24 |
| hsa04960 Aldosterone-regulated sodium reabsorption | 0.632741574 | -0.416445033 | 0.978932102 | 1 | 10 |
| hsa00310 Lysine degradation | 0.630090024 | -0.462072457 | 0.988689747 | 1 | 22 |
| hsa04916 Melanogenesis | 0.646485588 | -0.460283933 | 0.988879279 | 1 | 34 |
| hsa04973 Carbohydrate digestion and absorption | 0.563684556 | -0.470555615 | 0.989102108 | 1 | 18 |
| hsa00532 Glycosaminoglycan biosynthesis - chondroitin sulfate | 0.658066183 | -0.503942878 | 0.993264607 | 1 | 13 |
| hsa00270 Cysteine and methionine metabolism | 0.679659665 | -0.544775497 | 0.996385101 | 1 | 17 |
| hsa04340 Hedgehog signaling pathway | 0.678342919 | -0.600543156 | 0.998269544 | 1 | 11 |
| hsa03430 Mismatch repair | 0.698681355 | -0.630980814 | 0.998908488 | 1 | 11 |
| hsa00562 Inositol phosphate metabolism | 0.719329022 | -0.639779258 | 0.999231949 | 1 | 26 |
| hsa04070 Phosphatidylinositol signaling system | 0.752229232 | -0.78148155 | 0.999946204 | 1 | 34 |
| hsa00510 N-Glycan biosynthesis | 0.742642409 | -0.804923087 | 0.999964123 | 1 | 24 |
| hsa00563 Glycosylphosphatidylinositol(GPI)-anchor biosynthesis | 0.754347074 | -0.827410274 | 0.99996613 | 1 | 10 |
| hsa04062 Chemokine signaling pathway | 0.725827791 | -0.831323797 | 0.999982373 | 1 | 76 |
| hsa03030 DNA replication | 0.72731904 | -0.855610501 | 0.999984305 | 1 | 17 |
| hsa04640 Hematopoietic cell lineage | 0.732854786 | -0.849686685 | 0.999987777 | 1 | 58 |
| hsa04662 B cell receptor signaling pathway | 0.760958496 | -0.898786315 | 0.999995839 | 1 | 44 |
| hsa00020 Citrate cycle (TCA cycle) | 0.742066421 | -0.929314515 | 0.999996686 | 1 | 17 |
| hsa03018 RNA degradation | 0.746401894 | -0.916010176 | 0.999997028 | 1 | 40 |
| hsa00280 Valine, leucine and isoleucine degradation | 0.760691731 | -0.957904813 | 0.999998633 | 1 | 21 |
| hsa00410 beta-Alanine metabolism | 0.769873659 | -0.98251582 | 0.999998816 | 1 | 11 |
| hsa00970 Aminoacyl-tRNA biosynthesis | 0.830592233 | -1.07549933 | 0.999999915 | 1 | 15 |
| hsa03410 Base excision repair | 0.809762962 | -1.079736532 | 0.999999921 | 1 | 15 |
| hsa04310 Wnt signaling pathway | 0.829752605 | -1.075144023 | 0.999999955 | 1 | 62 |
| hsa03010 Ribosome | 0.600564096 | -1.103220314 | 0.999999967 | 1 | 50 |
| hsa03015 mRNA surveillance pathway | 0.854753573 | -1.17004162 | 0.999999997 | 1 | 43 |
| hsa03040 Spliceosome | 0.882692491 | -1.608389136 | 1 | 1 | 65 |
| hsa03008 Ribosome biogenesis in eukaryotes | 0.933492962 | -2.697995688 | 1 | 1 | 44 |
| hsa03013 RNA transport | 0.902985199 | -2.195454485 | 1 | 1 | 83 |
| hsa04514 Cell adhesion molecules (CAMs) | 0.949170919 | -2.153334239 | 1 | 1 | 57 |
| hsa04612 Antigen processing and presentation | 0.952883458 | -3.234270584 | 1 | 1 | 52 |
| hsa04650 Natural killer cell mediated cytotoxicity | 0.782493364 | -1.926989937 | 1 | 1 | 62 |
| hsa04660 T cell receptor signaling pathway | 0.928005785 | -1.862981781 | 1 | 1 | 60 |
| hsa04672 Intestinal immune network for IgA production | 0.982274511 | -2.737004834 | 1 | 1 | 29 |
| hsa00040 Pentose and glucuronate interconversions | NA | NA | NA | NA | 8 |
| hsa00053 Ascorbate and aldarate metabolism | NA | NA | NA | NA | 6 |
| hsa00061 Fatty acid biosynthesis | NA | NA | NA | NA | 2 |
| hsa00072 Synthesis and degradation of ketone bodies | NA | NA | NA | NA | 2 |
| hsa00100 Steroid biosynthesis | NA | NA | NA | NA | 6 |
| hsa00120 Primary bile acid biosynthesis | NA | NA | NA | NA | 4 |
| hsa00130 Ubiquinone and other terpenoid-quinone biosynthesis | NA | NA | NA | NA | 5 |
| hsa00140 Steroid hormone biosynthesis | NA | NA | NA | NA | 8 |
| hsa00232 Caffeine metabolism | NA | NA | NA | NA | 2 |
| hsa00250 Alanine, aspartate and glutamate metabolism | NA | NA | NA | NA | 8 |
| hsa00290 Valine, leucine and isoleucine biosynthesis | NA | NA | NA | NA | 5 |
| hsa00300 Lysine biosynthesis | NA | NA | NA | NA | 1 |
| hsa00360 Phenylalanine metabolism | NA | NA | NA | NA | 9 |
| hsa00400 Phenylalanine, tyrosine and tryptophan biosynthesis | NA | NA | NA | NA | 2 |
| hsa00430 Taurine and hypotaurine metabolism | NA | NA | NA | NA | 6 |
| hsa00450 Selenocompound metabolism | NA | NA | NA | NA | 6 |
| hsa00460 Cyanoamino acid metabolism | NA | NA | NA | NA | 4 |
| hsa00471 D-Glutamine and D-glutamate metabolism | NA | NA | NA | NA | 2 |
| hsa00472 D-Arginine and D-ornithine metabolism | NA | NA | NA | NA | 0 |
| hsa00511 Other glycan degradation | NA | NA | NA | NA | 6 |
| hsa00531 Glycosaminoglycan degradation | NA | NA | NA | NA | 5 |
| hsa00533 Glycosaminoglycan biosynthesis - keratan sulfate | NA | NA | NA | NA | 6 |
| hsa00591 Linoleic acid metabolism | NA | NA | NA | NA | 8 |
| hsa00592 alpha-Linolenic acid metabolism | NA | NA | NA | NA | 6 |
| hsa00601 Glycosphingolipid biosynthesis - lacto and neolacto series | NA | NA | NA | NA | 9 |
| hsa00603 Glycosphingolipid biosynthesis - globo series | NA | NA | NA | NA | 6 |
| hsa00604 Glycosphingolipid biosynthesis - ganglio series | NA | NA | NA | NA | 8 |
| hsa00630 Glyoxylate and dicarboxylate metabolism | NA | NA | NA | NA | 7 |
| hsa00650 Butanoate metabolism | NA | NA | NA | NA | 8 |
| hsa00730 Thiamine metabolism | NA | NA | NA | NA | 1 |
| hsa00740 Riboflavin metabolism | NA | NA | NA | NA | 4 |
| hsa00750 Vitamin B6 metabolism | NA | NA | NA | NA | 4 |
| hsa00770 Pantothenate and CoA biosynthesis | NA | NA | NA | NA | 9 |
| hsa00780 Biotin metabolism | NA | NA | NA | NA | 1 |
| hsa00785 Lipoic acid metabolism | NA | NA | NA | NA | 1 |
| hsa00790 Folate biosynthesis | NA | NA | NA | NA | 6 |
| hsa00860 Porphyrin and chlorophyll metabolism | NA | NA | NA | NA | 8 |
| hsa00900 Terpenoid backbone biosynthesis | NA | NA | NA | NA | 6 |
| hsa00910 Nitrogen metabolism | NA | NA | NA | NA | 7 |
| hsa00920 Sulfur metabolism | NA | NA | NA | NA | 5 |
| hsa01040 Biosynthesis of unsaturated fatty acids | NA | NA | NA | NA | 5 |
| hsa03020 RNA polymerase | NA | NA | NA | NA | 7 |
| hsa03450 Non-homologous end-joining | NA | NA | NA | NA | 7 |
| hsa04122 Sulfur relay system | NA | NA | NA | NA | 4 |
| hsa04140 Regulation of autophagy | NA | NA | NA | NA | 9 |
| hsa04320 Dorso-ventral axis formation | NA | NA | NA | NA | 9 |
| hsa04614 Renin-angiotensin system | NA | NA | NA | NA | 7 |
| hsa04710 Circadian rhythm - mammal | NA | NA | NA | NA | 8 |
| hsa04744 Phototransduction | NA | NA | NA | NA | 7 |
| hsa04964 Proximal tubule bicarbonate reclamation | NA | NA | NA | NA | 8 |
| hsa04977 Vitamin digestion and absorption | NA | NA | NA | NA | 6 |
| hsa04612 Antigen processing and presentation | 0.000523855 | -3.234270584 | 2.90E-55 | 3.66E-53 | 52 |
| hsa04672 Intestinal immune network for IgA production | 0.003608133 | -2.737004834 | 1.45E-39 | 9.14E-38 | 29 |
| hsa03008 Ribosome biogenesis in eukaryotes | 0.002848282 | -2.697995688 | 7.18E-39 | 3.01E-37 | 44 |
| hsa03013 RNA transport | 0.008861591 | -2.195454485 | 1.67E-27 | 5.26E-26 | 83 |
| hsa04514 Cell adhesion molecules (CAMs) | 0.013269218 | -2.153334239 | 1.64E-26 | 4.13E-25 | 57 |
| hsa04650 Natural killer cell mediated cytotoxicity | 0.016188012 | -1.926989937 | 1.29E-21 | 2.71E-20 | 62 |
| hsa04660 T cell receptor signaling pathway | 0.027671058 | -1.862981781 | 1.71E-20 | 3.08E-19 | 60 |
| hsa03040 Spliceosome | 0.043947735 | -1.608389136 | 1.03E-15 | 1.62E-14 | 65 |
| hsa03015 mRNA surveillance pathway | 0.115837243 | -1.17004162 | 3.47E-09 | 4.86E-08 | 43 |
| hsa03010 Ribosome | 0.07335328 | -1.103220314 | 3.33E-08 | 4.19E-07 | 50 |
| hsa04310 Wnt signaling pathway | 0.1326302 | -1.075144023 | 4.54E-08 | 5.20E-07 | 62 |
| hsa03410 Base excision repair | 0.131498851 | -1.079736532 | 7.90E-08 | 8.26E-07 | 15 |
| hsa00970 Aminoacyl-tRNA biosynthesis | 0.139709149 | -1.07549933 | 8.52E-08 | 8.26E-07 | 15 |
| hsa00410 beta-Alanine metabolism | 0.14997983 | -0.98251582 | 1.18E-06 | 1.07E-05 | 11 |
| hsa00280 Valine, leucine and isoleucine degradation | 0.149152517 | -0.957904813 | 1.37E-06 | 1.15E-05 | 21 |
| hsa03018 RNA degradation | 0.153556353 | -0.916010176 | 2.97E-06 | 2.34E-05 | 40 |
| hsa00020 Citrate cycle (TCA cycle) | 0.152560813 | -0.929314515 | 3.31E-06 | 2.46E-05 | 17 |
| hsa04662 B cell receptor signaling pathway | 0.164107074 | -0.898786315 | 4.16E-06 | 2.91E-05 | 44 |
| hsa04640 Hematopoietic cell lineage | 0.170597846 | -0.849686685 | 1.22E-05 | 8.11E-05 | 58 |
| hsa03030 DNA replication | 0.171875001 | -0.855610501 | 1.57E-05 | 9.89E-05 | 17 |
| hsa04062 Chemokine signaling pathway | 0.173611772 | -0.831323797 | 1.76E-05 | 0.000105764 | 76 |
| hsa00563 Glycosylphosphatidylinositol(GPI)-anchor biosynthesis | 0.197394403 | -0.827410274 | 3.39E-05 | 0.000193981 | 10 |
| hsa00510 N-Glycan biosynthesis | 0.194260047 | -0.804923087 | 3.59E-05 | 0.000196544 | 24 |
| hsa04070 Phosphatidylinositol signaling system | 0.205451724 | -0.78148155 | 5.38E-05 | 0.00028243 | 34 |
| hsa00562 Inositol phosphate metabolism | 0.253258602 | -0.639779258 | 0.000768051 | 0.003870975 | 26 |
| hsa03430 Mismatch repair | 0.252554997 | -0.630980814 | 0.001091512 | 0.005289635 | 11 |
| hsa04340 Hedgehog signaling pathway | 0.257368442 | -0.600543156 | 0.001730456 | 0.008075459 | 11 |
| hsa00270 Cysteine and methionine metabolism | 0.280645536 | -0.544775497 | 0.003614899 | 0.016267044 | 17 |
| hsa00532 Glycosaminoglycan biosynthesis - chondroitin sulfate | 0.291398593 | -0.503942878 | 0.006735393 | 0.02926412 | 13 |
| hsa04973 Carbohydrate digestion and absorption | 0.254854847 | -0.470555615 | 0.010897892 | 0.044534123 | 18 |
| hsa04916 Melanogenesis | 0.304475636 | -0.460283933 | 0.011120721 | 0.044534123 | 34 |
| hsa00310 Lysine degradation | 0.295033806 | -0.462072457 | 0.011310253 | 0.044534123 | 22 |
| hsa04960 Aldosterone-regulated sodium reabsorption | 0.324334045 | -0.416445033 | 0.021067898 | 0.080441067 | 10 |
| hsa04330 Notch signaling pathway | 0.317887841 | -0.379344691 | 0.030675703 | 0.113680548 | 24 |
| hsa04520 Adherens junction | 0.328442497 | -0.373886164 | 0.032150473 | 0.115741704 | 24 |
| hsa04350 TGF-beta signaling pathway | 0.349250592 | -0.336026743 | 0.04744568 | 0.166059879 | 35 |
| hsa03420 Nucleotide excision repair | 0.329832375 | -0.323576625 | 0.055332587 | 0.18842989 | 25 |
| hsa00620 Pyruvate metabolism | 0.342572971 | -0.310559692 | 0.062343896 | 0.206719234 | 23 |
| hsa00340 Histidine metabolism | 0.333928116 | -0.310346216 | 0.065354761 | 0.211146149 | 13 |
| hsa00640 Propanoate metabolism | 0.372682622 | -0.284453623 | 0.079902815 | 0.251693867 | 18 |
| hsa00830 Retinol metabolism | 0.364943768 | -0.281975685 | 0.082353529 | 0.253086455 | 16 |
| hsa04970 Salivary secretion | 0.367057709 | -0.253868279 | 0.10414763 | 0.311406438 | 31 |
| hsa04742 Taste transduction | 0.3658902 | -0.256586374 | 0.106273626 | 0.311406438 | 10 |
| hsa04150 mTOR signaling pathway | 0.389306737 | -0.234823538 | 0.122475172 | 0.350724356 | 21 |
| hsa04971 Gastric acid secretion | 0.388325221 | -0.226702517 | 0.130817894 | 0.366290102 | 22 |
| hsa00561 Glycerolipid metabolism | 0.394173247 | -0.21241531 | 0.146847505 | 0.394108996 | 17 |
| hsa00514 Other types of O-glycan biosynthesis | 0.393926675 | -0.213419928 | 0.147008911 | 0.394108996 | 14 |
| hsa04720 Long-term potentiation | 0.411840939 | -0.180977424 | 0.184446896 | 0.484173102 | 27 |
| hsa04962 Vasopressin-regulated water reabsorption | 0.416474537 | -0.160162354 | 0.214378577 | 0.551259198 | 17 |
| hsa00230 Purine metabolism | 0.416052114 | -0.122534621 | 0.270681853 | 0.68211827 | 68 |
| hsa00350 Tyrosine metabolism | 0.409470461 | -0.09133216 | 0.326852906 | 0.807518943 | 18 |
| hsa04974 Protein digestion and absorption | 0.403771361 | -0.055794664 | 0.392258824 | 0.950473304 | 22 |
| hsa04360 Axon guidance | 0.430196156 | -0.041949749 | 0.417233951 | 0.991914676 | 47 |
| hsa04530 Tight junction | 0.474244569 | -0.019501103 | 0.461404565 | 1 | 44 |
| hsa00071 Fatty acid metabolism | 0.472677313 | -0.012001475 | 0.476336588 | 1 | 17 |
| hsa02010 ABC transporters | 0.46215154 | 0.002992497 | 0.504546616 | 1 | 14 |
| hsa04012 ErbB signaling pathway | 0.474935018 | 0.017319915 | 0.534589924 | 1 | 35 |
| hsa03060 Protein export | 0.443777006 | 0.024222386 | 0.550415708 | 1 | 10 |
| hsa03022 Basal transcription factors | 0.474863816 | 0.033928731 | 0.567831411 | 1 | 18 |
| hsa04722 Neurotrophin signaling pathway | 0.472238096 | 0.054288695 | 0.606539801 | 1 | 57 |
| hsa04210 Apoptosis | 0.493081991 | 0.056735492 | 0.611153947 | 1 | 46 |
| hsa00670 One carbon pool by folate | 0.564890872 | 0.287184159 | 0.920373733 | 1 | 12 |
| hsa04630 Jak-STAT signaling pathway | 0.563463798 | 0.307136844 | 0.936827539 | 1 | 58 |
| hsa00534 Glycosaminoglycan biosynthesis - heparan sulfate | 0.585617582 | 0.317377344 | 0.940252089 | 1 | 13 |
| hsa04010 MAPK signaling pathway | 0.596063 | 0.325789019 | 0.948013748 | 1 | 115 |
| hsa04146 Peroxisome | 0.579722145 | 0.331725449 | 0.950221238 | 1 | 38 |
| hsa04664 Fc epsilon RI signaling pathway | 0.595183688 | 0.346755923 | 0.95755098 | 1 | 37 |
| hsa03440 Homologous recombination | 0.595740168 | 0.353219158 | 0.958463946 | 1 | 14 |
| hsa00590 Arachidonic acid metabolism | 0.573712725 | 0.351848932 | 0.959203002 | 1 | 23 |
| hsa00030 Pentose phosphate pathway | 0.608276192 | 0.372203862 | 0.965966504 | 1 | 13 |
| hsa04670 Leukocyte transendothelial migration | 0.607902507 | 0.397233084 | 0.975899658 | 1 | 44 |
| hsa04623 Cytosolic DNA-sensing pathway | 0.602867187 | 0.411779465 | 0.978469344 | 1 | 16 |
| hsa00330 Arginine and proline metabolism | 0.60728957 | 0.413545829 | 0.979593754 | 1 | 25 |
| hsa00500 Starch and sucrose metabolism | 0.531845906 | 0.438474035 | 0.983546419 | 1 | 22 |
| hsa04120 Ubiquitin mediated proteolysis | 0.550911734 | 0.430095275 | 0.983838053 | 1 | 67 |
| hsa00380 Tryptophan metabolism | 0.62017177 | 0.450467062 | 0.986463236 | 1 | 14 |
| hsa00240 Pyrimidine metabolism | 0.619162397 | 0.466165507 | 0.989694767 | 1 | 41 |
| hsa04972 Pancreatic secretion | 0.655654595 | 0.483228191 | 0.991778653 | 1 | 32 |
| hsa04144 Endocytosis | 0.623815554 | 0.495980721 | 0.993251381 | 1 | 83 |
| hsa04370 VEGF signaling pathway | 0.666834121 | 0.535029288 | 0.996052618 | 1 | 35 |
| hsa00051 Fructose and mannose metabolism | 0.678419297 | 0.556020365 | 0.996873062 | 1 | 15 |
| hsa04966 Collecting duct acid secretion | 0.704166055 | 0.607748261 | 0.998552614 | 1 | 12 |
| hsa00520 Amino sugar and nucleotide sugar metabolism | 0.656216144 | 0.606883483 | 0.998644593 | 1 | 26 |
| hsa04976 Bile secretion | 0.628433114 | 0.613242698 | 0.998729556 | 1 | 24 |
| hsa04141 Protein processing in endoplasmic reticulum | 0.644324968 | 0.64753844 | 0.999358861 | 1 | 73 |
| hsa00052 Galactose metabolism | 0.707882858 | 0.658613474 | 0.999396769 | 1 | 16 |
| hsa04260 Cardiac muscle contraction | 0.71065169 | 0.664122781 | 0.9994887 | 1 | 26 |
| hsa04622 RIG-I-like receptor signaling pathway | 0.692420987 | 0.66978891 | 0.999503499 | 1 | 19 |
| hsa04020 Calcium signaling pathway | 0.694595944 | 0.665419209 | 0.99953452 | 1 | 60 |
| hsa04912 GnRH signaling pathway | 0.734398781 | 0.692392594 | 0.999709969 | 1 | 38 |
| hsa04145 Phagosome | 0.675665804 | 0.700067041 | 0.999749209 | 1 | 66 |
| hsa04730 Long-term depression | 0.743032726 | 0.71919779 | 0.999810788 | 1 | 22 |
| hsa00565 Ether lipid metabolism | 0.747723017 | 0.772445822 | 0.999924269 | 1 | 15 |
| hsa00260 Glycine, serine and threonine metabolism | 0.704776342 | 0.793688228 | 0.999938919 | 1 | 13 |
| hsa04920 Adipocytokine signaling pathway | 0.755692937 | 0.77741916 | 0.999941446 | 1 | 30 |
| hsa04740 Olfactory transduction | 0.703626954 | 0.795203478 | 0.999952033 | 1 | 21 |
| hsa04910 Insulin signaling pathway | 0.750087893 | 0.814558066 | 0.999974611 | 1 | 61 |
| hsa04540 Gap junction | 0.756074517 | 0.822060927 | 0.999976746 | 1 | 33 |
| hsa04380 Osteoclast differentiation | 0.732672682 | 0.837186175 | 0.99998426 | 1 | 63 |
| hsa00010 Glycolysis / Gluconeogenesis | 0.776599182 | 0.882224723 | 0.999993698 | 1 | 32 |
| hsa04620 Toll-like receptor signaling pathway | 0.741048659 | 0.906496796 | 0.999996367 | 1 | 40 |
| hsa04130 SNARE interactions in vesicular transport | 0.756803545 | 0.927354124 | 0.999996903 | 1 | 19 |
| hsa04914 Progesterone-mediated oocyte maturation | 0.794016914 | 0.963241312 | 0.999999137 | 1 | 48 |
| hsa00600 Sphingolipid metabolism | 0.784293464 | 0.996496531 | 0.999999454 | 1 | 18 |
| hsa00983 Drug metabolism - other enzymes | 0.822685832 | 1.017635843 | 0.999999663 | 1 | 14 |
| hsa00512 Mucin type O-Glycan biosynthesis | 0.820575761 | 1.03887001 | 0.999999814 | 1 | 16 |
| hsa00982 Drug metabolism - cytochrome P450 | 0.789039557 | 1.047449477 | 0.999999837 | 1 | 18 |
| hsa04975 Fat digestion and absorption | 0.826605566 | 1.076401023 | 0.999999899 | 1 | 11 |
| hsa03050 Proteasome | 0.716775376 | 1.077954357 | 0.999999909 | 1 | 20 |
| hsa04142 Lysosome | 0.78760498 | 1.062334826 | 0.999999929 | 1 | 51 |
| hsa00760 Nicotinate and nicotinamide metabolism | 0.827350269 | 1.093036465 | 0.999999939 | 1 | 12 |
| hsa04666 Fc gamma R-mediated phagocytosis | 0.794899718 | 1.079777444 | 0.999999956 | 1 | 48 |
| hsa04621 NOD-like receptor signaling pathway | 0.821443303 | 1.131738888 | 0.999999987 | 1 | 24 |
| hsa04114 Oocyte meiosis | 0.805424104 | 1.148173946 | 0.999999994 | 1 | 57 |
| hsa04110 Cell cycle | 0.737795564 | 1.156511344 | 0.999999995 | 1 | 73 |
| hsa04270 Vascular smooth muscle contraction | 0.834560817 | 1.158940797 | 0.999999995 | 1 | 46 |
| hsa00564 Glycerophospholipid metabolism | 0.845579225 | 1.178702969 | 0.999999997 | 1 | 36 |
| hsa04115 p53 signaling pathway | 0.82909321 | 1.185104354 | 0.999999998 | 1 | 35 |
| hsa04510 Focal adhesion | 0.821324739 | 1.219373069 | 0.999999999 | 1 | 77 |
| hsa00980 Metabolism of xenobiotics by cytochrome P450 | 0.849546822 | 1.261096653 | 1 | 1 | 17 |
| hsa00480 Glutathione metabolism | 0.901997925 | 1.440816773 | 1 | 1 | 22 |
| hsa04810 Regulation of actin cytoskeleton | 0.888693247 | 1.619092984 | 1 | 1 | 87 |
| hsa00190 Oxidative phosphorylation | 0.839757082 | 1.648845137 | 1 | 1 | 55 |
| hsa03320 PPAR signaling pathway | 0.985572277 | 2.423348559 | 1 | 1 | 23 |
| hsa04512 ECM-receptor interaction | 0.889855434 | 1.752947614 | 1 | 1 | 32 |
| hsa04610 Complement and coagulation cascades | 0.975002192 | 2.557470086 | 1 | 1 | 22 |
| hsa00040 Pentose and glucuronate interconversions | NA | NA | NA | NA | 8 |
| hsa00053 Ascorbate and aldarate metabolism | NA | NA | NA | NA | 6 |
| hsa00061 Fatty acid biosynthesis | NA | NA | NA | NA | 2 |
| hsa00072 Synthesis and degradation of ketone bodies | NA | NA | NA | NA | 2 |
| hsa00100 Steroid biosynthesis | NA | NA | NA | NA | 6 |
| hsa00120 Primary bile acid biosynthesis | NA | NA | NA | NA | 4 |
| hsa00130 Ubiquinone and other terpenoid-quinone biosynthesis | NA | NA | NA | NA | 5 |
| hsa00140 Steroid hormone biosynthesis | NA | NA | NA | NA | 8 |
| hsa00232 Caffeine metabolism | NA | NA | NA | NA | 2 |
| hsa00250 Alanine, aspartate and glutamate metabolism | NA | NA | NA | NA | 8 |
| hsa00290 Valine, leucine and isoleucine biosynthesis | NA | NA | NA | NA | 5 |
| hsa00300 Lysine biosynthesis | NA | NA | NA | NA | 1 |
| hsa00360 Phenylalanine metabolism | NA | NA | NA | NA | 9 |
| hsa00400 Phenylalanine, tyrosine and tryptophan biosynthesis | NA | NA | NA | NA | 2 |
| hsa00430 Taurine and hypotaurine metabolism | NA | NA | NA | NA | 6 |
| hsa00450 Selenocompound metabolism | NA | NA | NA | NA | 6 |
| hsa00460 Cyanoamino acid metabolism | NA | NA | NA | NA | 4 |
| hsa00471 D-Glutamine and D-glutamate metabolism | NA | NA | NA | NA | 2 |
| hsa00472 D-Arginine and D-ornithine metabolism | NA | NA | NA | NA | 0 |
| hsa00511 Other glycan degradation | NA | NA | NA | NA | 6 |
| hsa00531 Glycosaminoglycan degradation | NA | NA | NA | NA | 5 |
| hsa00533 Glycosaminoglycan biosynthesis - keratan sulfate | NA | NA | NA | NA | 6 |
| hsa00591 Linoleic acid metabolism | NA | NA | NA | NA | 8 |
| hsa00592 alpha-Linolenic acid metabolism | NA | NA | NA | NA | 6 |
| hsa00601 Glycosphingolipid biosynthesis - lacto and neolacto series | NA | NA | NA | NA | 9 |
| hsa00603 Glycosphingolipid biosynthesis - globo series | NA | NA | NA | NA | 6 |
| hsa00604 Glycosphingolipid biosynthesis - ganglio series | NA | NA | NA | NA | 8 |
| hsa00630 Glyoxylate and dicarboxylate metabolism | NA | NA | NA | NA | 7 |
| hsa00650 Butanoate metabolism | NA | NA | NA | NA | 8 |
| hsa00730 Thiamine metabolism | NA | NA | NA | NA | 1 |
| hsa00740 Riboflavin metabolism | NA | NA | NA | NA | 4 |
| hsa00750 Vitamin B6 metabolism | NA | NA | NA | NA | 4 |
| hsa00770 Pantothenate and CoA biosynthesis | NA | NA | NA | NA | 9 |
| hsa00780 Biotin metabolism | NA | NA | NA | NA | 1 |
| hsa00785 Lipoic acid metabolism | NA | NA | NA | NA | 1 |
| hsa00790 Folate biosynthesis | NA | NA | NA | NA | 6 |
| hsa00860 Porphyrin and chlorophyll metabolism | NA | NA | NA | NA | 8 |
| hsa00900 Terpenoid backbone biosynthesis | NA | NA | NA | NA | 6 |
| hsa00910 Nitrogen metabolism | NA | NA | NA | NA | 7 |
| hsa00920 Sulfur metabolism | NA | NA | NA | NA | 5 |
| hsa01040 Biosynthesis of unsaturated fatty acids | NA | NA | NA | NA | 5 |
| hsa03020 RNA polymerase | NA | NA | NA | NA | 7 |
| hsa03450 Non-homologous end-joining | NA | NA | NA | NA | 7 |
| hsa04122 Sulfur relay system | NA | NA | NA | NA | 4 |
| hsa04140 Regulation of autophagy | NA | NA | NA | NA | 9 |
| hsa04320 Dorso-ventral axis formation | NA | NA | NA | NA | 9 |
| hsa04614 Renin-angiotensin system | NA | NA | NA | NA | 7 |
| hsa04710 Circadian rhythm - mammal | NA | NA | NA | NA | 8 |
| hsa04744 Phototransduction | NA | NA | NA | NA | 7 |
| hsa04964 Proximal tubule bicarbonate reclamation | NA | NA | NA | NA | 8 |
| hsa04977 Vitamin digestion and absorption | NA | NA | NA | NA | 6 |
|  |  |  |  |  |  |
| **GSE95233** | **p.geomean** | **stat.mean** | **p.val** | **q.val** | **set.**  **size** |
| hsa00190 Oxidative phosphorylation | 0.002405527 | 2.799196786 | 5.20E-38 | 6.45E-36 | 63 |
| hsa03320 PPAR signaling pathway | 0.023874351 | 2.041525896 | 3.13E-20 | 1.94E-18 | 17 |
| hsa04610 Complement and coagulation cascades | 0.027170072 | 1.925687416 | 1.70E-18 | 7.01E-17 | 20 |
| hsa00600 Sphingolipid metabolism | 0.043531155 | 1.739475885 | 1.97E-15 | 6.09E-14 | 16 |
| hsa04115 p53 signaling pathway | 0.059317356 | 1.532608268 | 8.32E-13 | 2.06E-11 | 30 |
| hsa04260 Cardiac muscle contraction | 0.064059077 | 1.478909164 | 4.89E-12 | 1.01E-10 | 30 |
| hsa00480 Glutathione metabolism | 0.074180722 | 1.419136235 | 4.36E-11 | 7.72E-10 | 20 |
| hsa04621 NOD-like receptor signaling pathway | 0.082022662 | 1.347229837 | 2.47E-10 | 3.71E-09 | 31 |
| hsa04114 Oocyte meiosis | 0.0802321 | 1.336928384 | 2.69E-10 | 3.71E-09 | 49 |
| hsa00770 Pantothenate and CoA biosynthesis | 0.09051072 | 1.367442579 | 4.36E-10 | 5.40E-09 | 10 |
| hsa04110 Cell cycle | 0.077352396 | 1.293514089 | 9.25E-10 | 1.04E-08 | 64 |
| hsa04130 SNARE interactions in vesicular transport | 0.120251197 | 1.166149092 | 4.17E-08 | 4.31E-07 | 23 |
| hsa04966 Collecting duct acid secretion | 0.121037232 | 1.181083 | 5.34E-08 | 5.09E-07 | 10 |
| hsa00564 Glycerophospholipid metabolism | 0.11804511 | 1.137647858 | 6.87E-08 | 6.08E-07 | 31 |
| hsa00512 Mucin type O-Glycan biosynthesis | 0.12296791 | 1.128623959 | 1.37E-07 | 1.13E-06 | 15 |
| hsa04142 Lysosome | 0.147791752 | 0.96042121 | 3.92E-06 | 3.04E-05 | 52 |
| hsa03010 Ribosome | 0.101761727 | 0.952292575 | 5.58E-06 | 4.07E-05 | 57 |
| hsa03050 Proteasome | 0.155425607 | 0.916588863 | 1.18E-05 | 8.13E-05 | 28 |
| hsa04810 Regulation of actin cytoskeleton | 0.153795464 | 0.902471843 | 1.28E-05 | 8.34E-05 | 74 |
| hsa00760 Nicotinate and nicotinamide metabolism | 0.172569009 | 0.918897089 | 1.65E-05 | 0.000102243 | 11 |
| hsa00500 Starch and sucrose metabolism | 0.176279294 | 0.896219269 | 2.13E-05 | 0.00012579 | 13 |
| hsa04622 RIG-I-like receptor signaling pathway | 0.18326514 | 0.832021411 | 5.99E-05 | 0.000337554 | 25 |
| hsa04914 Progesterone-mediated oocyte maturation | 0.187167838 | 0.820907802 | 6.61E-05 | 0.000355288 | 44 |
| hsa04540 Gap junction | 0.190249119 | 0.822086341 | 6.88E-05 | 0.000355288 | 27 |
| hsa04512 ECM-receptor interaction | 0.198250317 | 0.776777836 | 0.000184832 | 0.000916769 | 16 |
| hsa04620 Toll-like receptor signaling pathway | 0.16949466 | 0.757073757 | 0.000220714 | 0.001052638 | 43 |
| hsa00983 Drug metabolism - other enzymes | 0.225893716 | 0.733123885 | 0.000357773 | 0.001643104 | 16 |
| hsa00010 Glycolysis / Gluconeogenesis | 0.223996747 | 0.71951314 | 0.000418399 | 0.001852911 | 28 |
| hsa00565 Ether lipid metabolism | 0.229568047 | 0.699884077 | 0.000680638 | 0.002910313 | 12 |
| hsa04141 Protein processing in endoplasmic reticulum | 0.226173816 | 0.681900788 | 0.000719969 | 0.002975871 | 77 |
| hsa04666 Fc gamma R-mediated phagocytosis | 0.229237667 | 0.65880283 | 0.001072398 | 0.004289592 | 45 |
| hsa03060 Protein export | 0.249549271 | 0.640082208 | 0.00175819 | 0.006812988 | 12 |
| hsa04510 Focal adhesion | 0.260932042 | 0.562041878 | 0.004324374 | 0.015891748 | 65 |
| hsa04910 Insulin signaling pathway | 0.24335982 | 0.561929824 | 0.004416662 | 0.015891748 | 53 |
| hsa04730 Long-term depression | 0.271049583 | 0.564432997 | 0.004485574 | 0.015891748 | 19 |
| hsa00240 Pyrimidine metabolism | 0.267918513 | 0.549812329 | 0.005175318 | 0.017826094 | 46 |
| hsa04912 GnRH signaling pathway | 0.280525461 | 0.546837186 | 0.005397301 | 0.018088251 | 35 |
| hsa00052 Galactose metabolism | 0.290773336 | 0.536632657 | 0.006730104 | 0.021509737 | 12 |
| hsa04120 Ubiquitin mediated proteolysis | 0.268014629 | 0.528831177 | 0.006765159 | 0.021509737 | 62 |
| hsa03022 Basal transcription factors | 0.30664421 | 0.476814175 | 0.01358191 | 0.042103921 | 19 |
| hsa00520 Amino sugar and nucleotide sugar metabolism | 0.304550057 | 0.469809265 | 0.014674645 | 0.044381854 | 20 |
| hsa04270 Vascular smooth muscle contraction | 0.292864477 | 0.459014684 | 0.016213707 | 0.04786904 | 40 |
| hsa04140 Regulation of autophagy | 0.304982413 | 0.463150401 | 0.01724683 | 0.049735045 | 10 |
| hsa04920 Adipocytokine signaling pathway | 0.31954882 | 0.419574248 | 0.025854521 | 0.07286274 | 19 |
| hsa00982 Drug metabolism - cytochrome P450 | 0.32054652 | 0.375061263 | 0.042630916 | 0.117471858 | 15 |
| hsa04145 Phagosome | 0.305152604 | 0.355111418 | 0.048713175 | 0.131313776 | 63 |
| hsa04976 Bile secretion | 0.361439108 | 0.292586915 | 0.087524971 | 0.230916944 | 19 |
| hsa00410 beta-Alanine metabolism | 0.371970908 | 0.285064325 | 0.094677734 | 0.244584147 | 10 |
| hsa00670 One carbon pool by folate | 0.364183609 | 0.262694129 | 0.114236267 | 0.289087696 | 10 |
| hsa04623 Cytosolic DNA-sensing pathway | 0.366978383 | 0.254134941 | 0.119304662 | 0.293481185 | 23 |
| hsa00590 Arachidonic acid metabolism | 0.378843844 | 0.25495875 | 0.120705971 | 0.293481185 | 11 |
| hsa00051 Fructose and mannose metabolism | 0.408307276 | 0.181185009 | 0.200480688 | 0.46459696 | 17 |
| hsa00140 Steroid hormone biosynthesis | 0.392742352 | 0.182230976 | 0.201691477 | 0.46459696 | 11 |
| hsa04144 Endocytosis | 0.385298326 | 0.178315479 | 0.202324483 | 0.46459696 | 80 |
| hsa00330 Arginine and proline metabolism | 0.400007749 | 0.174296707 | 0.209125745 | 0.471483497 | 23 |
| hsa04380 Osteoclast differentiation | 0.370299683 | 0.164313504 | 0.221069318 | 0.489510633 | 68 |
| hsa00030 Pentose phosphate pathway | 0.432383801 | 0.144500958 | 0.252945098 | 0.550266529 | 10 |
| hsa04146 Peroxisome | 0.429049467 | 0.130951373 | 0.270787726 | 0.578048747 | 31 |
| hsa04010 MAPK signaling pathway | 0.399691392 | 0.12767921 | 0.275039323 | 0.578048747 | 100 |
| hsa03420 Nucleotide excision repair | 0.421770752 | 0.111723024 | 0.301650743 | 0.623411536 | 26 |
| hsa03430 Mismatch repair | 0.425176367 | 0.105504754 | 0.314511617 | 0.639335091 | 11 |
| hsa04974 Protein digestion and absorption | 0.409096433 | 0.086254184 | 0.345501396 | 0.680383033 | 17 |
| hsa04664 Fc epsilon RI signaling pathway | 0.430407946 | 0.085270771 | 0.345678477 | 0.680383033 | 30 |
| hsa04972 Pancreatic secretion | 0.451248965 | 0.060689464 | 0.389054944 | 0.753793954 | 22 |
| hsa04962 Vasopressin-regulated water reabsorption | 0.471824615 | 0.03268503 | 0.439642548 | 0.838702707 | 19 |
| hsa04740 Olfactory transduction | 0.461171799 | 0.00897602 | 0.483279626 | 0.907979902 | 13 |
| hsa04960 Aldosterone-regulated sodium reabsorption | 0.476469895 | 0.001433239 | 0.496890405 | 0.919618063 | 14 |
| hsa03015 mRNA surveillance pathway | 0.483397082 | -0.01291453 | 0.523870469 | 0.949097087 | 28 |
| hsa00980 Metabolism of xenobiotics by cytochrome P450 | 0.46091798 | -0.017370575 | 0.532495006 | 0.949097087 | 15 |
| hsa04150 mTOR signaling pathway | 0.47292885 | -0.019289802 | 0.535780613 | 0.949097087 | 23 |
| hsa04340 Hedgehog signaling pathway | 0.462662989 | -0.031613012 | 0.557810197 | 0.970953798 | 14 |
| hsa00620 Pyruvate metabolism | 0.500093423 | -0.037140756 | 0.568413811 | 0.970953798 | 16 |
| hsa00640 Propanoate metabolism | 0.493372052 | -0.039010701 | 0.571609897 | 0.970953798 | 14 |
| hsa04330 Notch signaling pathway | 0.486978011 | -0.045831183 | 0.583663284 | 0.971335763 | 16 |
| hsa03018 RNA degradation | 0.491614107 | -0.047445635 | 0.587501469 | 0.971335763 | 31 |
| hsa00020 Citrate cycle (TCA cycle) | 0.505988796 | -0.05475885 | 0.600121557 | 0.977247166 | 17 |
| hsa00071 Fatty acid metabolism | 0.504199231 | -0.058851455 | 0.606838966 | 0.977247166 | 16 |
| hsa04020 Calcium signaling pathway | 0.516302166 | -0.153175556 | 0.762847733 | 1 | 54 |
| hsa00380 Tryptophan metabolism | 0.518422045 | -0.175853273 | 0.788310377 | 1 | 12 |
| hsa04360 Axon guidance | 0.509780843 | -0.18935995 | 0.811219373 | 1 | 41 |
| hsa04210 Apoptosis | 0.536704845 | -0.190111157 | 0.812507383 | 1 | 49 |
| hsa04973 Carbohydrate digestion and absorption | 0.546063843 | -0.196097416 | 0.817571972 | 1 | 14 |
| hsa04971 Gastric acid secretion | 0.54938446 | -0.211472955 | 0.836938788 | 1 | 24 |
| hsa04370 VEGF signaling pathway | 0.550486729 | -0.212760948 | 0.839042436 | 1 | 31 |
| hsa04720 Long-term potentiation | 0.557502193 | -0.255018734 | 0.881460693 | 1 | 29 |
| hsa03030 DNA replication | 0.539718677 | -0.280035079 | 0.902772456 | 1 | 19 |
| hsa03040 Spliceosome | 0.565471284 | -0.314034859 | 0.928633923 | 1 | 62 |
| hsa00561 Glycerolipid metabolism | 0.604228446 | -0.34748943 | 0.945763894 | 1 | 16 |
| hsa04670 Leukocyte transendothelial migration | 0.594623185 | -0.3498243 | 0.948501202 | 1 | 41 |
| hsa00230 Purine metabolism | 0.60064389 | -0.398751409 | 0.968850921 | 1 | 67 |
| hsa00280 Valine, leucine and isoleucine degradation | 0.61161551 | -0.405330006 | 0.969706615 | 1 | 22 |
| hsa03020 RNA polymerase | 0.627935963 | -0.420896642 | 0.973794645 | 1 | 15 |
| hsa00563 Glycosylphosphatidylinositol(GPI)-anchor biosynthesis | 0.629197644 | -0.425756424 | 0.973819205 | 1 | 10 |
| hsa04916 Melanogenesis | 0.62085355 | -0.424410724 | 0.97578663 | 1 | 31 |
| hsa00270 Cysteine and methionine metabolism | 0.64261013 | -0.435500686 | 0.978174685 | 1 | 18 |
| hsa04970 Salivary secretion | 0.655957267 | -0.481615545 | 0.987497686 | 1 | 29 |
| hsa00514 Other types of O-glycan biosynthesis | 0.666225585 | -0.517012131 | 0.991443934 | 1 | 14 |
| hsa04662 B cell receptor signaling pathway | 0.640081314 | -0.517485781 | 0.991968065 | 1 | 41 |
| hsa03410 Base excision repair | 0.653636769 | -0.526089022 | 0.992113123 | 1 | 14 |
| hsa00310 Lysine degradation | 0.66470473 | -0.528728928 | 0.992689874 | 1 | 17 |
| hsa04350 TGF-beta signaling pathway | 0.695985624 | -0.566944806 | 0.99582273 | 1 | 26 |
| hsa00510 N-Glycan biosynthesis | 0.668029074 | -0.573956449 | 0.996087483 | 1 | 22 |
| hsa00830 Retinol metabolism | 0.70030628 | -0.612940663 | 0.997594785 | 1 | 13 |
| hsa04710 Circadian rhythm - mammal | 0.707529521 | -0.62694447 | 0.997934098 | 1 | 10 |
| hsa04520 Adherens junction | 0.70653717 | -0.627616024 | 0.998206007 | 1 | 22 |
| hsa04722 Neurotrophin signaling pathway | 0.677203681 | -0.643469671 | 0.998638926 | 1 | 50 |
| hsa00532 Glycosaminoglycan biosynthesis - chondroitin sulfate | 0.724090055 | -0.693820692 | 0.999216523 | 1 | 10 |
| hsa04012 ErbB signaling pathway | 0.713986543 | -0.715104655 | 0.999546754 | 1 | 30 |
| hsa00562 Inositol phosphate metabolism | 0.757191001 | -0.786642798 | 0.999872327 | 1 | 31 |
| hsa04742 Taste transduction | 0.802124012 | -0.968677766 | 0.999994491 | 1 | 11 |
| hsa04630 Jak-STAT signaling pathway | 0.788789658 | -0.94752164 | 0.999995032 | 1 | 60 |
| hsa04070 Phosphatidylinositol signaling system | 0.811839185 | -0.962674659 | 0.999996316 | 1 | 42 |
| hsa04530 Tight junction | 0.802053756 | -1.008323496 | 0.99999856 | 1 | 34 |
| hsa04310 Wnt signaling pathway | 0.816982134 | -1.034466898 | 0.999999277 | 1 | 49 |
| hsa00970 Aminoacyl-tRNA biosynthesis | 0.825350021 | -1.099066804 | 0.999999584 | 1 | 10 |
| hsa04062 Chemokine signaling pathway | 0.808304824 | -1.110360608 | 0.999999888 | 1 | 76 |
| hsa04640 Hematopoietic cell lineage | 0.889669695 | -1.499443554 | 1 | 1 | 46 |
| hsa03013 RNA transport | 0.899836378 | -1.557272524 | 1 | 1 | 61 |
| hsa03008 Ribosome biogenesis in eukaryotes | 0.889365877 | -1.642285908 | 1 | 1 | 37 |
| hsa04514 Cell adhesion molecules (CAMs) | 0.99642481 | -3.492975858 | 1 | 1 | 56 |
| hsa04612 Antigen processing and presentation | 0.996742098 | -4.19632946 | 1 | 1 | 48 |
| hsa04650 Natural killer cell mediated cytotoxicity | 0.956887957 | -2.589426258 | 1 | 1 | 69 |
| hsa04660 T cell receptor signaling pathway | 0.984081578 | -2.447563982 | 1 | 1 | 57 |
| hsa04672 Intestinal immune network for IgA production | 0.986454584 | -2.868405959 | 1 | 1 | 25 |
| hsa00040 Pentose and glucuronate interconversions | NA | NA | NA | NA | 5 |
| hsa00053 Ascorbate and aldarate metabolism | NA | NA | NA | NA | 4 |
| hsa00061 Fatty acid biosynthesis | NA | NA | NA | NA | 1 |
| hsa00072 Synthesis and degradation of ketone bodies | NA | NA | NA | NA | 3 |
| hsa00100 Steroid biosynthesis | NA | NA | NA | NA | 8 |
| hsa00120 Primary bile acid biosynthesis | NA | NA | NA | NA | 2 |
| hsa00130 Ubiquinone and other terpenoid-quinone biosynthesis | NA | NA | NA | NA | 5 |
| hsa00232 Caffeine metabolism | NA | NA | NA | NA | 3 |
| hsa00250 Alanine, aspartate and glutamate metabolism | NA | NA | NA | NA | 8 |
| hsa00260 Glycine, serine and threonine metabolism | NA | NA | NA | NA | 9 |
| hsa00290 Valine, leucine and isoleucine biosynthesis | NA | NA | NA | NA | 3 |
| hsa00300 Lysine biosynthesis | NA | NA | NA | NA | 1 |
| hsa00340 Histidine metabolism | NA | NA | NA | NA | 7 |
| hsa00350 Tyrosine metabolism | NA | NA | NA | NA | 8 |
| hsa00360 Phenylalanine metabolism | NA | NA | NA | NA | 5 |
| hsa00400 Phenylalanine, tyrosine and tryptophan biosynthesis | NA | NA | NA | NA | 2 |
| hsa00430 Taurine and hypotaurine metabolism | NA | NA | NA | NA | 1 |
| hsa00450 Selenocompound metabolism | NA | NA | NA | NA | 4 |
| hsa00460 Cyanoamino acid metabolism | NA | NA | NA | NA | 3 |
| hsa00471 D-Glutamine and D-glutamate metabolism | NA | NA | NA | NA | 2 |
| hsa00472 D-Arginine and D-ornithine metabolism | NA | NA | NA | NA | 0 |
| hsa00511 Other glycan degradation | NA | NA | NA | NA | 7 |
| hsa00531 Glycosaminoglycan degradation | NA | NA | NA | NA | 7 |
| hsa00533 Glycosaminoglycan biosynthesis - keratan sulfate | NA | NA | NA | NA | 6 |
| hsa00534 Glycosaminoglycan biosynthesis - heparan sulfate | NA | NA | NA | NA | 8 |
| hsa00591 Linoleic acid metabolism | NA | NA | NA | NA | 6 |
| hsa00592 alpha-Linolenic acid metabolism | NA | NA | NA | NA | 4 |
| hsa00601 Glycosphingolipid biosynthesis - lacto and neolacto series | NA | NA | NA | NA | 9 |
| hsa00603 Glycosphingolipid biosynthesis - globo series | NA | NA | NA | NA | 6 |
| hsa00604 Glycosphingolipid biosynthesis - ganglio series | NA | NA | NA | NA | 8 |
| hsa00630 Glyoxylate and dicarboxylate metabolism | NA | NA | NA | NA | 9 |
| hsa00650 Butanoate metabolism | NA | NA | NA | NA | 5 |
| hsa00730 Thiamine metabolism | NA | NA | NA | NA | 1 |
| hsa00740 Riboflavin metabolism | NA | NA | NA | NA | 3 |
| hsa00750 Vitamin B6 metabolism | NA | NA | NA | NA | 2 |
| hsa00780 Biotin metabolism | NA | NA | NA | NA | 0 |
| hsa00785 Lipoic acid metabolism | NA | NA | NA | NA | 1 |
| hsa00790 Folate biosynthesis | NA | NA | NA | NA | 4 |
| hsa00860 Porphyrin and chlorophyll metabolism | NA | NA | NA | NA | 6 |
| hsa00900 Terpenoid backbone biosynthesis | NA | NA | NA | NA | 7 |
| hsa00910 Nitrogen metabolism | NA | NA | NA | NA | 7 |
| hsa00920 Sulfur metabolism | NA | NA | NA | NA | 4 |
| hsa01040 Biosynthesis of unsaturated fatty acids | NA | NA | NA | NA | 7 |
| hsa02010 ABC transporters | NA | NA | NA | NA | 9 |
| hsa03440 Homologous recombination | NA | NA | NA | NA | 8 |
| hsa03450 Non-homologous end-joining | NA | NA | NA | NA | 7 |
| hsa04122 Sulfur relay system | NA | NA | NA | NA | 2 |
| hsa04320 Dorso-ventral axis formation | NA | NA | NA | NA | 7 |
| hsa04614 Renin-angiotensin system | NA | NA | NA | NA | 5 |
| hsa04744 Phototransduction | NA | NA | NA | NA | 8 |
| hsa04964 Proximal tubule bicarbonate reclamation | NA | NA | NA | NA | 7 |
| hsa04975 Fat digestion and absorption | NA | NA | NA | NA | 9 |
| hsa04977 Vitamin digestion and absorption | NA | NA | NA | NA | 7 |
| hsa04612 Antigen processing and presentation | 2.27E-05 | -4.19632946 | 7.01E-78 | 8.69E-76 | 48 |
| hsa04514 Cell adhesion molecules (CAMs) | 0.000275167 | -3.492975858 | 7.12E-57 | 4.41E-55 | 56 |
| hsa04672 Intestinal immune network for IgA production | 0.002645237 | -2.868405959 | 1.68E-37 | 6.93E-36 | 25 |
| hsa04650 Natural killer cell mediated cytotoxicity | 0.003747811 | -2.589426258 | 4.69E-33 | 1.45E-31 | 69 |
| hsa04660 T cell receptor signaling pathway | 0.007193874 | -2.447563982 | 8.09E-30 | 2.01E-28 | 57 |
| hsa03008 Ribosome biogenesis in eukaryotes | 0.043118563 | -1.642285908 | 2.12E-14 | 4.38E-13 | 37 |
| hsa03013 RNA transport | 0.053971972 | -1.557272524 | 2.42E-13 | 4.30E-12 | 61 |
| hsa04640 Hematopoietic cell lineage | 0.058596705 | -1.499443554 | 1.99E-12 | 3.08E-11 | 46 |
| hsa04062 Chemokine signaling pathway | 0.114471919 | -1.110360608 | 1.12E-07 | 1.55E-06 | 76 |
| hsa00970 Aminoacyl-tRNA biosynthesis | 0.136777386 | -1.099066804 | 4.16E-07 | 5.16E-06 | 10 |
| hsa04310 Wnt signaling pathway | 0.140968001 | -1.034466898 | 7.23E-07 | 8.15E-06 | 49 |
| hsa04530 Tight junction | 0.144967977 | -1.008323496 | 1.44E-06 | 1.49E-05 | 34 |
| hsa04070 Phosphatidylinositol signaling system | 0.161567494 | -0.962674659 | 3.68E-06 | 3.51E-05 | 42 |
| hsa04630 Jak-STAT signaling pathway | 0.156512192 | -0.94752164 | 4.97E-06 | 4.40E-05 | 60 |
| hsa04742 Taste transduction | 0.164815459 | -0.968677766 | 5.51E-06 | 4.55E-05 | 11 |
| hsa00562 Inositol phosphate metabolism | 0.20563815 | -0.786642798 | 0.000127673 | 0.000989466 | 31 |
| hsa04012 ErbB signaling pathway | 0.215333087 | -0.715104655 | 0.000453246 | 0.003306029 | 30 |
| hsa00532 Glycosaminoglycan biosynthesis - chondroitin sulfate | 0.236119334 | -0.693820692 | 0.000783477 | 0.005397286 | 10 |
| hsa04722 Neurotrophin signaling pathway | 0.227714622 | -0.643469671 | 0.001361074 | 0.008882797 | 50 |
| hsa04520 Adherens junction | 0.253445218 | -0.627616024 | 0.001793993 | 0.011122755 | 22 |
| hsa04710 Circadian rhythm - mammal | 0.258587199 | -0.62694447 | 0.002065902 | 0.012198657 | 10 |
| hsa00830 Retinol metabolism | 0.259222479 | -0.612940663 | 0.002405215 | 0.013556667 | 13 |
| hsa00510 N-Glycan biosynthesis | 0.260816553 | -0.573956449 | 0.003912517 | 0.021093567 | 22 |
| hsa04350 TGF-beta signaling pathway | 0.276808272 | -0.566944806 | 0.00417727 | 0.021582563 | 26 |
| hsa00310 Lysine degradation | 0.280948854 | -0.528728928 | 0.007310126 | 0.036258226 | 17 |
| hsa03410 Base excision repair | 0.277480592 | -0.526089022 | 0.007886877 | 0.036887404 | 14 |
| hsa04662 B cell receptor signaling pathway | 0.267297743 | -0.517485781 | 0.008031935 | 0.036887404 | 41 |
| hsa00514 Other types of O-glycan biosynthesis | 0.288745698 | -0.517012131 | 0.008556066 | 0.037891148 | 14 |
| hsa04970 Salivary secretion | 0.298667629 | -0.481615545 | 0.012502314 | 0.053458172 | 29 |
| hsa00270 Cysteine and methionine metabolism | 0.31779388 | -0.435500686 | 0.021825315 | 0.090211301 | 18 |
| hsa04916 Melanogenesis | 0.307809028 | -0.424410724 | 0.02421337 | 0.096853478 | 31 |
| hsa00563 Glycosylphosphatidylinositol(GPI)-anchor biosynthesis | 0.318205481 | -0.425756424 | 0.026180795 | 0.098468607 | 10 |
| hsa03020 RNA polymerase | 0.318128709 | -0.420896642 | 0.026205355 | 0.098468607 | 15 |
| hsa00280 Valine, leucine and isoleucine degradation | 0.313740567 | -0.405330006 | 0.030293385 | 0.110356737 | 22 |
| hsa00230 Purine metabolism | 0.312231752 | -0.398751409 | 0.031149079 | 0.110356737 | 67 |
| hsa04670 Leukocyte transendothelial migration | 0.333252451 | -0.3498243 | 0.051498798 | 0.17738475 | 41 |
| hsa00561 Glycerolipid metabolism | 0.344661037 | -0.34748943 | 0.054236106 | 0.181764247 | 16 |
| hsa03040 Spliceosome | 0.33695989 | -0.314034859 | 0.071366077 | 0.232878777 | 62 |
| hsa03030 DNA replication | 0.342349169 | -0.280035079 | 0.097227544 | 0.30913373 | 19 |
| hsa04720 Long-term potentiation | 0.364436535 | -0.255018734 | 0.118539307 | 0.367471851 | 29 |
| hsa04370 VEGF signaling pathway | 0.388621546 | -0.212760948 | 0.160957564 | 0.481418815 | 31 |
| hsa04971 Gastric acid secretion | 0.388819729 | -0.211472955 | 0.163061212 | 0.481418815 | 24 |
| hsa04973 Carbohydrate digestion and absorption | 0.398617215 | -0.196097416 | 0.182428028 | 0.520195506 | 14 |
| hsa04210 Apoptosis | 0.392313712 | -0.190111157 | 0.187492617 | 0.520195506 | 49 |
| hsa04360 Axon guidance | 0.372183603 | -0.18935995 | 0.188780627 | 0.520195506 | 41 |
| hsa00380 Tryptophan metabolism | 0.388325157 | -0.175853273 | 0.211689623 | 0.570641592 | 12 |
| hsa04020 Calcium signaling pathway | 0.401436606 | -0.153175556 | 0.237152267 | 0.625678321 | 54 |
| hsa00071 Fatty acid metabolism | 0.458066187 | -0.058851455 | 0.393161034 | 1 | 16 |
| hsa00020 Citrate cycle (TCA cycle) | 0.463937459 | -0.05475885 | 0.399878443 | 1 | 17 |
| hsa03018 RNA degradation | 0.455178294 | -0.047445635 | 0.412498531 | 1 | 31 |
| hsa04330 Notch signaling pathway | 0.452373772 | -0.045831183 | 0.416336716 | 1 | 16 |
| hsa00640 Propanoate metabolism | 0.46349701 | -0.039010701 | 0.428390103 | 1 | 14 |
| hsa00620 Pyruvate metabolism | 0.471219028 | -0.037140756 | 0.431586189 | 1 | 16 |
| hsa04340 Hedgehog signaling pathway | 0.440340955 | -0.031613012 | 0.442189803 | 1 | 14 |
| hsa04150 mTOR signaling pathway | 0.458495402 | -0.019289802 | 0.464219387 | 1 | 23 |
| hsa00980 Metabolism of xenobiotics by cytochrome P450 | 0.449064886 | -0.017370575 | 0.467504994 | 1 | 15 |
| hsa03015 mRNA surveillance pathway | 0.473359166 | -0.01291453 | 0.476129531 | 1 | 28 |
| hsa04960 Aldosterone-regulated sodium reabsorption | 0.477092799 | 0.001433239 | 0.503109595 | 1 | 14 |
| hsa04740 Olfactory transduction | 0.468390194 | 0.00897602 | 0.516720374 | 1 | 13 |
| hsa04962 Vasopressin-regulated water reabsorption | 0.497051442 | 0.03268503 | 0.560357452 | 1 | 19 |
| hsa04972 Pancreatic secretion | 0.498085967 | 0.060689464 | 0.610945056 | 1 | 22 |
| hsa04664 Fc epsilon RI signaling pathway | 0.494742064 | 0.085270771 | 0.654321523 | 1 | 30 |
| hsa04974 Protein digestion and absorption | 0.47183504 | 0.086254184 | 0.654498604 | 1 | 17 |
| hsa03430 Mismatch repair | 0.504461898 | 0.105504754 | 0.685488383 | 1 | 11 |
| hsa03420 Nucleotide excision repair | 0.505818768 | 0.111723024 | 0.698349257 | 1 | 26 |
| hsa04010 MAPK signaling pathway | 0.492553192 | 0.12767921 | 0.724960677 | 1 | 100 |
| hsa04146 Peroxisome | 0.530199964 | 0.130951373 | 0.729212274 | 1 | 31 |
| hsa00030 Pentose phosphate pathway | 0.543688226 | 0.144500958 | 0.747054902 | 1 | 10 |
| hsa04380 Osteoclast differentiation | 0.483918119 | 0.164313504 | 0.778930682 | 1 | 68 |
| hsa00330 Arginine and proline metabolism | 0.530166547 | 0.174296707 | 0.790874255 | 1 | 23 |
| hsa04144 Endocytosis | 0.519600384 | 0.178315479 | 0.797675517 | 1 | 80 |
| hsa00140 Steroid hormone biosynthesis | 0.526475503 | 0.182230976 | 0.798308523 | 1 | 11 |
| hsa00051 Fructose and mannose metabolism | 0.546679988 | 0.181185009 | 0.799519312 | 1 | 17 |
| hsa00590 Arachidonic acid metabolism | 0.570427709 | 0.25495875 | 0.879294029 | 1 | 11 |
| hsa04623 Cytosolic DNA-sensing pathway | 0.557675072 | 0.254134941 | 0.880695338 | 1 | 23 |
| hsa00670 One carbon pool by folate | 0.554795924 | 0.262694129 | 0.885763733 | 1 | 10 |
| hsa00410 beta-Alanine metabolism | 0.585168381 | 0.285064325 | 0.905322266 | 1 | 10 |
| hsa04976 Bile secretion | 0.580590188 | 0.292586915 | 0.912475029 | 1 | 19 |
| hsa04145 Phagosome | 0.555574469 | 0.355111418 | 0.951286825 | 1 | 63 |
| hsa00982 Drug metabolism - cytochrome P450 | 0.592457984 | 0.375061263 | 0.957369084 | 1 | 15 |
| hsa04920 Adipocytokine signaling pathway | 0.630891889 | 0.419574248 | 0.974145479 | 1 | 19 |
| hsa04140 Regulation of autophagy | 0.642203171 | 0.463150401 | 0.98275317 | 1 | 10 |
| hsa04270 Vascular smooth muscle contraction | 0.626300305 | 0.459014684 | 0.983786293 | 1 | 40 |
| hsa00520 Amino sugar and nucleotide sugar metabolism | 0.653491632 | 0.469809265 | 0.985325355 | 1 | 20 |
| hsa03022 Basal transcription factors | 0.661041852 | 0.476814175 | 0.98641809 | 1 | 19 |
| hsa04120 Ubiquitin mediated proteolysis | 0.646291348 | 0.528831177 | 0.993234841 | 1 | 62 |
| hsa00052 Galactose metabolism | 0.687462737 | 0.536632657 | 0.993269896 | 1 | 12 |
| hsa04912 GnRH signaling pathway | 0.684718997 | 0.546837186 | 0.994602699 | 1 | 35 |
| hsa00240 Pyrimidine metabolism | 0.668038916 | 0.549812329 | 0.994824682 | 1 | 46 |
| hsa04730 Long-term depression | 0.680593855 | 0.564432997 | 0.995514426 | 1 | 19 |
| hsa04910 Insulin signaling pathway | 0.633543422 | 0.561929824 | 0.995583338 | 1 | 53 |
| hsa04510 Focal adhesion | 0.664757309 | 0.562041878 | 0.995675626 | 1 | 65 |
| hsa03060 Protein export | 0.701516917 | 0.640082208 | 0.99824181 | 1 | 12 |
| hsa04666 Fc gamma R-mediated phagocytosis | 0.691801397 | 0.65880283 | 0.998927602 | 1 | 45 |
| hsa04141 Protein processing in endoplasmic reticulum | 0.707083743 | 0.681900788 | 0.999280031 | 1 | 77 |
| hsa00565 Ether lipid metabolism | 0.718036268 | 0.699884077 | 0.999319362 | 1 | 12 |
| hsa00010 Glycolysis / Gluconeogenesis | 0.735564369 | 0.71951314 | 0.999581601 | 1 | 28 |
| hsa00983 Drug metabolism - other enzymes | 0.747265109 | 0.733123885 | 0.999642227 | 1 | 16 |
| hsa04620 Toll-like receptor signaling pathway | 0.638668924 | 0.757073757 | 0.999779286 | 1 | 43 |
| hsa04512 ECM-receptor interaction | 0.719223739 | 0.776777836 | 0.999815168 | 1 | 16 |
| hsa04540 Gap junction | 0.75049247 | 0.822086341 | 0.999931235 | 1 | 27 |
| hsa04914 Progesterone-mediated oocyte maturation | 0.746126178 | 0.820907802 | 0.999933856 | 1 | 44 |
| hsa04622 RIG-I-like receptor signaling pathway | 0.742904404 | 0.832021411 | 0.999940111 | 1 | 25 |
| hsa00500 Starch and sucrose metabolism | 0.775115118 | 0.896219269 | 0.999978697 | 1 | 13 |
| hsa00760 Nicotinate and nicotinamide metabolism | 0.778282497 | 0.918897089 | 0.999983509 | 1 | 11 |
| hsa04810 Regulation of actin cytoskeleton | 0.739967183 | 0.902471843 | 0.999987218 | 1 | 74 |
| hsa03050 Proteasome | 0.750495362 | 0.916588863 | 0.9999882 | 1 | 28 |
| hsa03010 Ribosome | 0.646488497 | 0.952292575 | 0.999994417 | 1 | 57 |
| hsa04142 Lysosome | 0.776112597 | 0.96042121 | 0.999996077 | 1 | 52 |
| hsa00512 Mucin type O-Glycan biosynthesis | 0.827482899 | 1.128623959 | 0.999999863 | 1 | 15 |
| hsa00564 Glycerophospholipid metabolism | 0.829897708 | 1.137647858 | 0.999999931 | 1 | 31 |
| hsa04966 Collecting duct acid secretion | 0.850884406 | 1.181083 | 0.999999947 | 1 | 10 |
| hsa04130 SNARE interactions in vesicular transport | 0.856609981 | 1.166149092 | 0.999999958 | 1 | 23 |
| hsa04110 Cell cycle | 0.816463858 | 1.293514089 | 0.999999999 | 1 | 64 |
| hsa00770 Pantothenate and CoA biosynthesis | 0.882714031 | 1.367442579 | 1 | 1 | 10 |
| hsa04114 Oocyte meiosis | 0.861120216 | 1.336928384 | 1 | 1 | 49 |
| hsa04621 NOD-like receptor signaling pathway | 0.870466033 | 1.347229837 | 1 | 1 | 31 |
| hsa00480 Glutathione metabolism | 0.883171355 | 1.419136235 | 1 | 1 | 20 |
| hsa04260 Cardiac muscle contraction | 0.890214928 | 1.478909164 | 1 | 1 | 30 |
| hsa04115 p53 signaling pathway | 0.902428216 | 1.532608268 | 1 | 1 | 30 |
| hsa00600 Sphingolipid metabolism | 0.936396717 | 1.739475885 | 1 | 1 | 16 |
| hsa00190 Oxidative phosphorylation | 0.987908268 | 2.799196786 | 1 | 1 | 63 |
| hsa03320 PPAR signaling pathway | 0.962964986 | 2.041525896 | 1 | 1 | 17 |
| hsa04610 Complement and coagulation cascades | 0.937569232 | 1.925687416 | 1 | 1 | 20 |
| hsa00040 Pentose and glucuronate interconversions | NA | NA | NA | NA | 5 |
| hsa00053 Ascorbate and aldarate metabolism | NA | NA | NA | NA | 4 |
| hsa00061 Fatty acid biosynthesis | NA | NA | NA | NA | 1 |
| hsa00072 Synthesis and degradation of ketone bodies | NA | NA | NA | NA | 3 |
| hsa00100 Steroid biosynthesis | NA | NA | NA | NA | 8 |
| hsa00120 Primary bile acid biosynthesis | NA | NA | NA | NA | 2 |
| hsa00130 Ubiquinone and other terpenoid-quinone biosynthesis | NA | NA | NA | NA | 5 |
| hsa00232 Caffeine metabolism | NA | NA | NA | NA | 3 |
| hsa00250 Alanine, aspartate and glutamate metabolism | NA | NA | NA | NA | 8 |
| hsa00260 Glycine, serine and threonine metabolism | NA | NA | NA | NA | 9 |
| hsa00290 Valine, leucine and isoleucine biosynthesis | NA | NA | NA | NA | 3 |
| hsa00300 Lysine biosynthesis | NA | NA | NA | NA | 1 |
| hsa00340 Histidine metabolism | NA | NA | NA | NA | 7 |
| hsa00350 Tyrosine metabolism | NA | NA | NA | NA | 8 |
| hsa00360 Phenylalanine metabolism | NA | NA | NA | NA | 5 |
| hsa00400 Phenylalanine, tyrosine and tryptophan biosynthesis | NA | NA | NA | NA | 2 |
| hsa00430 Taurine and hypotaurine metabolism | NA | NA | NA | NA | 1 |
| hsa00450 Selenocompound metabolism | NA | NA | NA | NA | 4 |
| hsa00460 Cyanoamino acid metabolism | NA | NA | NA | NA | 3 |
| hsa00471 D-Glutamine and D-glutamate metabolism | NA | NA | NA | NA | 2 |
| hsa00472 D-Arginine and D-ornithine metabolism | NA | NA | NA | NA | 0 |
| hsa00511 Other glycan degradation | NA | NA | NA | NA | 7 |
| hsa00531 Glycosaminoglycan degradation | NA | NA | NA | NA | 7 |
| hsa00533 Glycosaminoglycan biosynthesis - keratan sulfate | NA | NA | NA | NA | 6 |
| hsa00534 Glycosaminoglycan biosynthesis - heparan sulfate | NA | NA | NA | NA | 8 |
| hsa00591 Linoleic acid metabolism | NA | NA | NA | NA | 6 |
| hsa00592 alpha-Linolenic acid metabolism | NA | NA | NA | NA | 4 |
| hsa00601 Glycosphingolipid biosynthesis - lacto and neolacto series | NA | NA | NA | NA | 9 |
| hsa00603 Glycosphingolipid biosynthesis - globo series | NA | NA | NA | NA | 6 |
| hsa00604 Glycosphingolipid biosynthesis - ganglio series | NA | NA | NA | NA | 8 |
| hsa00630 Glyoxylate and dicarboxylate metabolism | NA | NA | NA | NA | 9 |
| hsa00650 Butanoate metabolism | NA | NA | NA | NA | 5 |
| hsa00730 Thiamine metabolism | NA | NA | NA | NA | 1 |
| hsa00740 Riboflavin metabolism | NA | NA | NA | NA | 3 |
| hsa00750 Vitamin B6 metabolism | NA | NA | NA | NA | 2 |
| hsa00780 Biotin metabolism | NA | NA | NA | NA | 0 |
| hsa00785 Lipoic acid metabolism | NA | NA | NA | NA | 1 |
| hsa00790 Folate biosynthesis | NA | NA | NA | NA | 4 |
| hsa00860 Porphyrin and chlorophyll metabolism | NA | NA | NA | NA | 6 |
| hsa00900 Terpenoid backbone biosynthesis | NA | NA | NA | NA | 7 |
| hsa00910 Nitrogen metabolism | NA | NA | NA | NA | 7 |
| hsa00920 Sulfur metabolism | NA | NA | NA | NA | 4 |
| hsa01040 Biosynthesis of unsaturated fatty acids | NA | NA | NA | NA | 7 |
| hsa02010 ABC transporters | NA | NA | NA | NA | 9 |
| hsa03440 Homologous recombination | NA | NA | NA | NA | 8 |
| hsa03450 Non-homologous end-joining | NA | NA | NA | NA | 7 |
| hsa04122 Sulfur relay system | NA | NA | NA | NA | 2 |
| hsa04320 Dorso-ventral axis formation | NA | NA | NA | NA | 7 |
| hsa04614 Renin-angiotensin system | NA | NA | NA | NA | 5 |
| hsa04744 Phototransduction | NA | NA | NA | NA | 8 |
| hsa04964 Proximal tubule bicarbonate reclamation | NA | NA | NA | NA | 7 |
| hsa04975 Fat digestion and absorption | NA | NA | NA | NA | 9 |
| hsa04977 Vitamin digestion and absorption | NA | NA | NA | NA | 7 |
|  |  |  |  |  |  |
| **GSE64456** | **p.geomean** | **stat.mean** | **p.val** | **q.val** | **set.**  **size** |
| hsa04810 Regulation of actin cytoskeleton | 0.024980698 | 1.900312857 | 1.38E-16 | 1.58E-14 | 71 |
| hsa04380 Osteoclast differentiation | 0.023553361 | 1.784762018 | 9.44E-15 | 5.38E-13 | 65 |
| hsa04670 Leukocyte transendothelial migration | 0.035758481 | 1.760501027 | 3.10E-14 | 1.18E-12 | 40 |
| hsa04666 Fc gamma R-mediated phagocytosis | 0.045133485 | 1.649307533 | 6.41E-13 | 1.83E-11 | 47 |
| hsa04650 Natural killer cell mediated cytotoxicity | 0.072225444 | 1.368939285 | 2.04E-09 | 4.64E-08 | 44 |
| hsa04664 Fc epsilon RI signaling pathway | 0.105157532 | 1.199401081 | 1.43E-07 | 2.50E-06 | 26 |
| hsa04142 Lysosome | 0.097778324 | 1.186075718 | 1.53E-07 | 2.50E-06 | 51 |
| hsa04620 Toll-like receptor signaling pathway | 0.111964363 | 1.141764999 | 4.56E-07 | 6.50E-06 | 36 |
| hsa04510 Focal adhesion | 0.125720461 | 1.109655708 | 7.59E-07 | 9.61E-06 | 64 |
| hsa04010 MAPK signaling pathway | 0.12788067 | 1.078335 | 1.42E-06 | 1.62E-05 | 97 |
| hsa00500 Starch and sucrose metabolism | 0.147212645 | 1.060429956 | 3.46E-06 | 3.59E-05 | 12 |
| hsa04062 Chemokine signaling pathway | 0.124300391 | 1.031280151 | 4.04E-06 | 3.84E-05 | 72 |
| hsa04520 Adherens junction | 0.156783987 | 0.993747894 | 9.14E-06 | 7.85E-05 | 31 |
| hsa00051 Fructose and mannose metabolism | 0.154399738 | 1.004180356 | 9.63E-06 | 7.85E-05 | 15 |
| hsa04920 Adipocytokine signaling pathway | 0.153578219 | 0.980627912 | 1.23E-05 | 9.33E-05 | 27 |
| hsa00030 Pentose phosphate pathway | 0.134763581 | 1.000753343 | 1.46E-05 | 0.000104057 | 13 |
| hsa04910 Insulin signaling pathway | 0.17037218 | 0.922536153 | 3.23E-05 | 0.000216303 | 50 |
| hsa04530 Tight junction | 0.177743253 | 0.889621278 | 5.93E-05 | 0.000375845 | 41 |
| hsa00010 Glycolysis / Gluconeogenesis | 0.165722382 | 0.881968186 | 7.36E-05 | 0.000441622 | 30 |
| hsa04210 Apoptosis | 0.180736565 | 0.849188966 | 0.000121182 | 0.00069074 | 42 |
| hsa04145 Phagosome | 0.143549486 | 0.840558448 | 0.000139443 | 0.000756978 | 72 |
| hsa04722 Neurotrophin signaling pathway | 0.200912653 | 0.798404883 | 0.000272888 | 0.001414054 | 49 |
| hsa04971 Gastric acid secretion | 0.217670806 | 0.761693351 | 0.000529168 | 0.002519363 | 21 |
| hsa04976 Bile secretion | 0.215490917 | 0.762650629 | 0.000530392 | 0.002519363 | 19 |
| hsa03320 PPAR signaling pathway | 0.198346671 | 0.750264896 | 0.000669556 | 0.002987829 | 19 |
| hsa04144 Endocytosis | 0.207710515 | 0.738460837 | 0.000681435 | 0.002987829 | 67 |
| hsa00052 Galactose metabolism | 0.230275804 | 0.731257184 | 0.000936234 | 0.003952986 | 11 |
| hsa04973 Carbohydrate digestion and absorption | 0.24191186 | 0.701405749 | 0.001407961 | 0.005732411 | 11 |
| hsa04610 Complement and coagulation cascades | 0.245060211 | 0.659420134 | 0.002422761 | 0.009523956 | 16 |
| hsa04360 Axon guidance | 0.248083663 | 0.634750607 | 0.002988767 | 0.011357316 | 39 |
| hsa04621 NOD-like receptor signaling pathway | 0.227490129 | 0.624613615 | 0.003626699 | 0.013336891 | 30 |
| hsa04370 VEGF signaling pathway | 0.273274253 | 0.582740232 | 0.005923901 | 0.021103896 | 25 |
| hsa04730 Long-term depression | 0.27623823 | 0.56216917 | 0.00769666 | 0.026588463 | 20 |
| hsa00564 Glycerophospholipid metabolism | 0.290202996 | 0.531137092 | 0.01098721 | 0.036839469 | 27 |
| hsa00520 Amino sugar and nucleotide sugar metabolism | 0.294049878 | 0.518430093 | 0.012883231 | 0.041962525 | 18 |
| hsa00590 Arachidonic acid metabolism | 0.283143841 | 0.510081145 | 0.014354733 | 0.045456654 | 15 |
| hsa04630 Jak-STAT signaling pathway | 0.235728391 | 0.500166708 | 0.01536124 | 0.047329226 | 50 |
| hsa00983 Drug metabolism - other enzymes | 0.318271059 | 0.458430004 | 0.02518885 | 0.075333421 | 11 |
| hsa04115 p53 signaling pathway | 0.311111894 | 0.450529732 | 0.02577196 | 0.075333421 | 28 |
| hsa00600 Sphingolipid metabolism | 0.319834909 | 0.447575713 | 0.027971255 | 0.079718078 | 15 |
| hsa04540 Gap junction | 0.305646155 | 0.433600064 | 0.030430748 | 0.084612325 | 31 |
| hsa00860 Porphyrin and chlorophyll metabolism | 0.286189662 | 0.421130695 | 0.037565435 | 0.101963323 | 11 |
| hsa04150 mTOR signaling pathway | 0.340950455 | 0.397427429 | 0.04350669 | 0.113117877 | 17 |
| hsa04310 Wnt signaling pathway | 0.331469051 | 0.394569796 | 0.043659531 | 0.113117877 | 33 |
| hsa04662 B cell receptor signaling pathway | 0.303354669 | 0.340558377 | 0.070081134 | 0.177538873 | 36 |
| hsa00532 Glycosaminoglycan biosynthesis - chondroitin sulfate | 0.368164857 | 0.324222579 | 0.083395241 | 0.206631215 | 10 |
| hsa04141 Protein processing in endoplasmic reticulum | 0.331189279 | 0.316661248 | 0.085190062 | 0.206631215 | 55 |
| hsa03050 Proteasome | 0.359031213 | 0.268075669 | 0.127058766 | 0.30176457 | 15 |
| hsa04960 Aldosterone-regulated sodium reabsorption | 0.390996471 | 0.265854694 | 0.130219016 | 0.302958528 | 10 |
| hsa04146 Peroxisome | 0.389150368 | 0.23346118 | 0.156056346 | 0.355808468 | 27 |
| hsa04622 RIG-I-like receptor signaling pathway | 0.363319151 | 0.230038908 | 0.160749313 | 0.359321993 | 19 |
| hsa00562 Inositol phosphate metabolism | 0.411370149 | 0.184126116 | 0.215220712 | 0.471830023 | 16 |
| hsa04270 Vascular smooth muscle contraction | 0.401505332 | 0.175943223 | 0.223055223 | 0.479779158 | 32 |
| hsa04974 Protein digestion and absorption | 0.389797521 | 0.148973549 | 0.261125277 | 0.551264473 | 20 |
| hsa04623 Cytosolic DNA-sensing pathway | 0.36800736 | 0.124878059 | 0.300262436 | 0.622362141 | 17 |
| hsa04914 Progesterone-mediated oocyte maturation | 0.44240046 | 0.103737445 | 0.326500689 | 0.664662117 | 37 |
| hsa04512 ECM-receptor interaction | 0.436009472 | 0.061837572 | 0.392897886 | 0.777391009 | 17 |
| hsa04130 SNARE interactions in vesicular transport | 0.459979913 | 0.059400836 | 0.399168389 | 0.777391009 | 17 |
| hsa04320 Dorso-ventral axis formation | 0.460532672 | 0.056790719 | 0.406367605 | 0.777391009 | 10 |
| hsa00480 Glutathione metabolism | 0.431579726 | 0.053231095 | 0.409153163 | 0.777391009 | 18 |
| hsa04912 GnRH signaling pathway | 0.479846099 | 0.033999205 | 0.441562404 | 0.824213327 | 28 |
| hsa00330 Arginine and proline metabolism | 0.452189972 | 0.027380649 | 0.452115639 | 0.824213327 | 17 |
| hsa04640 Hematopoietic cell lineage | 0.45550092 | 0.025942863 | 0.455486312 | 0.824213327 | 49 |
| hsa04975 Fat digestion and absorption | 0.483864467 | 0.013198276 | 0.477428233 | 0.850419041 | 11 |
| hsa04962 Vasopressin-regulated water reabsorption | 0.490358214 | 0.002697565 | 0.495347886 | 0.868763984 | 15 |
| hsa00565 Ether lipid metabolism | 0.49525664 | -0.006552329 | 0.510954746 | 0.872449515 | 11 |
| hsa04916 Melanogenesis | 0.49429824 | -0.007438543 | 0.512755417 | 0.872449515 | 25 |
| hsa04972 Pancreatic secretion | 0.504725422 | -0.025481841 | 0.543806231 | 0.908570002 | 20 |
| hsa00071 Fatty acid metabolism | 0.481760323 | -0.03007917 | 0.549923948 | 0.908570002 | 14 |
| hsa04012 ErbB signaling pathway | 0.509151509 | -0.041425859 | 0.571024586 | 0.929954325 | 28 |
| hsa04340 Hedgehog signaling pathway | 0.515430457 | -0.051318852 | 0.586236862 | 0.941281722 | 11 |
| hsa04970 Salivary secretion | 0.52262667 | -0.071986586 | 0.621631808 | 0.984250362 | 16 |
| hsa04740 Olfactory transduction | 0.507316273 | -0.078865103 | 0.632770843 | 0.988162687 | 26 |
| hsa00534 Glycosaminoglycan biosynthesis - heparan sulfate | 0.528288504 | -0.093792889 | 0.655177429 | 1 | 13 |
| hsa00561 Glycerolipid metabolism | 0.533062754 | -0.099841366 | 0.667028534 | 1 | 21 |
| hsa00640 Propanoate metabolism | 0.533575311 | -0.125014024 | 0.704416472 | 1 | 12 |
| hsa04070 Phosphatidylinositol signaling system | 0.540308736 | -0.132137598 | 0.715115518 | 1 | 21 |
| hsa04720 Long-term potentiation | 0.546122583 | -0.133416835 | 0.717423883 | 1 | 23 |
| hsa03022 Basal transcription factors | 0.545041553 | -0.159747559 | 0.751115205 | 1 | 13 |
| hsa00310 Lysine degradation | 0.557897978 | -0.199252793 | 0.804005476 | 1 | 14 |
| hsa04350 TGF-beta signaling pathway | 0.56592326 | -0.209497793 | 0.816282057 | 1 | 23 |
| hsa00350 Tyrosine metabolism | 0.574378348 | -0.222558116 | 0.827279636 | 1 | 11 |
| hsa00982 Drug metabolism - cytochrome P450 | 0.577643382 | -0.258736332 | 0.864660474 | 1 | 10 |
| hsa04110 Cell cycle | 0.546692757 | -0.261309618 | 0.870768802 | 1 | 41 |
| hsa04114 Oocyte meiosis | 0.592320415 | -0.265090918 | 0.8745427 | 1 | 41 |
| hsa03015 mRNA surveillance pathway | 0.595717721 | -0.297152628 | 0.89864673 | 1 | 20 |
| hsa04020 Calcium signaling pathway | 0.604055818 | -0.295711026 | 0.899774828 | 1 | 38 |
| hsa00380 Tryptophan metabolism | 0.602509326 | -0.314256078 | 0.909773844 | 1 | 11 |
| hsa04330 Notch signaling pathway | 0.609621025 | -0.385076473 | 0.950341119 | 1 | 13 |
| hsa03440 Homologous recombination | 0.642460278 | -0.418375221 | 0.961313798 | 1 | 10 |
| hsa00240 Pyrimidine metabolism | 0.646417304 | -0.421613293 | 0.966005857 | 1 | 35 |
| hsa00340 Histidine metabolism | 0.649452139 | -0.437050794 | 0.968744737 | 1 | 11 |
| hsa00620 Pyruvate metabolism | 0.626413943 | -0.437595398 | 0.970432029 | 1 | 22 |
| hsa00280 Valine, leucine and isoleucine degradation | 0.651704934 | -0.44308936 | 0.971465917 | 1 | 18 |
| hsa00510 N-Glycan biosynthesis | 0.655610754 | -0.464390398 | 0.975679095 | 1 | 11 |
| hsa04120 Ubiquitin mediated proteolysis | 0.662805312 | -0.508989698 | 0.985995059 | 1 | 38 |
| hsa04660 T cell receptor signaling pathway | 0.618600677 | -0.531364514 | 0.989352139 | 1 | 52 |
| hsa03018 RNA degradation | 0.663623155 | -0.558974754 | 0.99204445 | 1 | 31 |
| hsa00514 Other types of O-glycan biosynthesis | 0.699843767 | -0.598736591 | 0.994492248 | 1 | 12 |
| hsa03420 Nucleotide excision repair | 0.698970853 | -0.626920967 | 0.996132789 | 1 | 14 |
| hsa04260 Cardiac muscle contraction | 0.630022903 | -0.635745402 | 0.996721052 | 1 | 19 |
| hsa00020 Citrate cycle (TCA cycle) | 0.703246737 | -0.681577417 | 0.998044929 | 1 | 12 |
| hsa03410 Base excision repair | 0.738252551 | -0.710759384 | 0.998729203 | 1 | 14 |
| hsa03030 DNA replication | 0.715694883 | -0.722475813 | 0.998874325 | 1 | 13 |
| hsa00970 Aminoacyl-tRNA biosynthesis | 0.694849798 | -0.765389945 | 0.999459538 | 1 | 16 |
| hsa03008 Ribosome biogenesis in eukaryotes | 0.718654093 | -0.82675526 | 0.999797242 | 1 | 25 |
| hsa00230 Purine metabolism | 0.766696857 | -0.926353324 | 0.99996939 | 1 | 53 |
| hsa04514 Cell adhesion molecules (CAMs) | 0.783995938 | -1.153409151 | 0.999999634 | 1 | 43 |
| hsa03040 Spliceosome | 0.818204218 | -1.211272899 | 0.999999911 | 1 | 55 |
| hsa00190 Oxidative phosphorylation | 0.518124003 | -1.286456727 | 0.99999998 | 1 | 50 |
| hsa03013 RNA transport | 0.81606492 | -1.4303563 | 1 | 1 | 52 |
| hsa04612 Antigen processing and presentation | 0.846477013 | -1.471357121 | 1 | 1 | 35 |
| hsa04672 Intestinal immune network for IgA production | 0.920134709 | -1.923000744 | 1 | 1 | 28 |
| hsa03010 Ribosome | 0.67474983 | -4.82383644 | 1 | 1 | 44 |
| hsa00040 Pentose and glucuronate interconversions | NA | NA | NA | NA | 6 |
| hsa00053 Ascorbate and aldarate metabolism | NA | NA | NA | NA | 4 |
| hsa00061 Fatty acid biosynthesis | NA | NA | NA | NA | 2 |
| hsa00072 Synthesis and degradation of ketone bodies | NA | NA | NA | NA | 1 |
| hsa00100 Steroid biosynthesis | NA | NA | NA | NA | 6 |
| hsa00120 Primary bile acid biosynthesis | NA | NA | NA | NA | 2 |
| hsa00130 Ubiquinone and other terpenoid-quinone biosynthesis | NA | NA | NA | NA | 4 |
| hsa00140 Steroid hormone biosynthesis | NA | NA | NA | NA | 5 |
| hsa00232 Caffeine metabolism | NA | NA | NA | NA | 0 |
| hsa00250 Alanine, aspartate and glutamate metabolism | NA | NA | NA | NA | 7 |
| hsa00260 Glycine, serine and threonine metabolism | NA | NA | NA | NA | 9 |
| hsa00270 Cysteine and methionine metabolism | NA | NA | NA | NA | 9 |
| hsa00290 Valine, leucine and isoleucine biosynthesis | NA | NA | NA | NA | 7 |
| hsa00300 Lysine biosynthesis | NA | NA | NA | NA | 0 |
| hsa00360 Phenylalanine metabolism | NA | NA | NA | NA | 5 |
| hsa00400 Phenylalanine, tyrosine and tryptophan biosynthesis | NA | NA | NA | NA | 1 |
| hsa00410 beta-Alanine metabolism | NA | NA | NA | NA | 7 |
| hsa00430 Taurine and hypotaurine metabolism | NA | NA | NA | NA | 1 |
| hsa00450 Selenocompound metabolism | NA | NA | NA | NA | 6 |
| hsa00460 Cyanoamino acid metabolism | NA | NA | NA | NA | 3 |
| hsa00471 D-Glutamine and D-glutamate metabolism | NA | NA | NA | NA | 0 |
| hsa00472 D-Arginine and D-ornithine metabolism | NA | NA | NA | NA | 0 |
| hsa00511 Other glycan degradation | NA | NA | NA | NA | 2 |
| hsa00512 Mucin type O-Glycan biosynthesis | NA | NA | NA | NA | 8 |
| hsa00531 Glycosaminoglycan degradation | NA | NA | NA | NA | 7 |
| hsa00533 Glycosaminoglycan biosynthesis - keratan sulfate | NA | NA | NA | NA | 4 |
| hsa00563 Glycosylphosphatidylinositol(GPI)-anchor biosynthesis | NA | NA | NA | NA | 5 |
| hsa00591 Linoleic acid metabolism | NA | NA | NA | NA | 4 |
| hsa00592 alpha-Linolenic acid metabolism | NA | NA | NA | NA | 3 |
| hsa00601 Glycosphingolipid biosynthesis - lacto and neolacto series | NA | NA | NA | NA | 9 |
| hsa00603 Glycosphingolipid biosynthesis - globo series | NA | NA | NA | NA | 4 |
| hsa00604 Glycosphingolipid biosynthesis - ganglio series | NA | NA | NA | NA | 6 |
| hsa00630 Glyoxylate and dicarboxylate metabolism | NA | NA | NA | NA | 8 |
| hsa00650 Butanoate metabolism | NA | NA | NA | NA | 7 |
| hsa00670 One carbon pool by folate | NA | NA | NA | NA | 5 |
| hsa00730 Thiamine metabolism | NA | NA | NA | NA | 1 |
| hsa00740 Riboflavin metabolism | NA | NA | NA | NA | 5 |
| hsa00750 Vitamin B6 metabolism | NA | NA | NA | NA | 2 |
| hsa00760 Nicotinate and nicotinamide metabolism | NA | NA | NA | NA | 5 |
| hsa00770 Pantothenate and CoA biosynthesis | NA | NA | NA | NA | 8 |
| hsa00780 Biotin metabolism | NA | NA | NA | NA | 0 |
| hsa00785 Lipoic acid metabolism | NA | NA | NA | NA | 2 |
| hsa00790 Folate biosynthesis | NA | NA | NA | NA | 3 |
| hsa00830 Retinol metabolism | NA | NA | NA | NA | 5 |
| hsa00900 Terpenoid backbone biosynthesis | NA | NA | NA | NA | 4 |
| hsa00910 Nitrogen metabolism | NA | NA | NA | NA | 5 |
| hsa00920 Sulfur metabolism | NA | NA | NA | NA | 6 |
| hsa00980 Metabolism of xenobiotics by cytochrome P450 | NA | NA | NA | NA | 9 |
| hsa01040 Biosynthesis of unsaturated fatty acids | NA | NA | NA | NA | 9 |
| hsa02010 ABC transporters | NA | NA | NA | NA | 7 |
| hsa03020 RNA polymerase | NA | NA | NA | NA | 8 |
| hsa03060 Protein export | NA | NA | NA | NA | 7 |
| hsa03430 Mismatch repair | NA | NA | NA | NA | 7 |
| hsa03450 Non-homologous end-joining | NA | NA | NA | NA | 3 |
| hsa04122 Sulfur relay system | NA | NA | NA | NA | 1 |
| hsa04140 Regulation of autophagy | NA | NA | NA | NA | 6 |
| hsa04614 Renin-angiotensin system | NA | NA | NA | NA | 5 |
| hsa04710 Circadian rhythm - mammal | NA | NA | NA | NA | 5 |
| hsa04742 Taste transduction | NA | NA | NA | NA | 8 |
| hsa04744 Phototransduction | NA | NA | NA | NA | 4 |
| hsa04964 Proximal tubule bicarbonate reclamation | NA | NA | NA | NA | 4 |
| hsa04966 Collecting duct acid secretion | NA | NA | NA | NA | 9 |
| hsa04977 Vitamin digestion and absorption | NA | NA | NA | NA | 4 |
| hsa03010 Ribosome | 7.77E-07 | -4.82383644 | 3.43E-78 | 3.91E-76 | 44 |
| hsa04672 Intestinal immune network for IgA production | 0.025613824 | -1.923000744 | 2.27E-16 | 1.29E-14 | 28 |
| hsa04612 Antigen processing and presentation | 0.059175179 | -1.471357121 | 1.65E-10 | 6.27E-09 | 35 |
| hsa03013 RNA transport | 0.062799083 | -1.4303563 | 3.80E-10 | 1.08E-08 | 52 |
| hsa00190 Oxidative phosphorylation | 0.039003681 | -1.286456727 | 2.00E-08 | 4.56E-07 | 50 |
| hsa03040 Spliceosome | 0.097913217 | -1.211272899 | 8.89E-08 | 1.69E-06 | 55 |
| hsa04514 Cell adhesion molecules (CAMs) | 0.101366727 | -1.153409151 | 3.66E-07 | 5.95E-06 | 43 |
| hsa00230 Purine metabolism | 0.159594793 | -0.926353324 | 3.06E-05 | 0.000436199 | 53 |
| hsa03008 Ribosome biogenesis in eukaryotes | 0.182118354 | -0.82675526 | 0.000202758 | 0.002568272 | 25 |
| hsa00970 Aminoacyl-tRNA biosynthesis | 0.199499524 | -0.765389945 | 0.000540462 | 0.006161265 | 16 |
| hsa03030 DNA replication | 0.22562444 | -0.722475813 | 0.001125675 | 0.011666083 | 13 |
| hsa03410 Base excision repair | 0.236561864 | -0.710759384 | 0.001270797 | 0.012072575 | 14 |
| hsa00020 Citrate cycle (TCA cycle) | 0.234045694 | -0.681577417 | 0.001955071 | 0.017144473 | 12 |
| hsa04260 Cardiac muscle contraction | 0.218658227 | -0.635745402 | 0.003278948 | 0.026700001 | 19 |
| hsa03420 Nucleotide excision repair | 0.257102696 | -0.626920967 | 0.003867211 | 0.029390805 | 14 |
| hsa00514 Other types of O-glycan biosynthesis | 0.268746322 | -0.598736591 | 0.005507752 | 0.039242736 | 12 |
| hsa03018 RNA degradation | 0.264004808 | -0.558974754 | 0.00795555 | 0.053348984 | 31 |
| hsa04660 T cell receptor signaling pathway | 0.256566763 | -0.531364514 | 0.010647861 | 0.067436456 | 52 |
| hsa04120 Ubiquitin mediated proteolysis | 0.289484996 | -0.508989698 | 0.014004941 | 0.084029648 | 38 |
| hsa00510 N-Glycan biosynthesis | 0.314209112 | -0.464390398 | 0.024320905 | 0.13862916 | 11 |
| hsa00280 Valine, leucine and isoleucine degradation | 0.320869632 | -0.44308936 | 0.028534083 | 0.153215852 | 18 |
| hsa00620 Pyruvate metabolism | 0.308438214 | -0.437595398 | 0.029567971 | 0.153215852 | 22 |
| hsa00340 Histidine metabolism | 0.324736621 | -0.437050794 | 0.031255263 | 0.154917388 | 11 |
| hsa00240 Pyrimidine metabolism | 0.327540915 | -0.421613293 | 0.033994143 | 0.161472178 | 35 |
| hsa03440 Homologous recombination | 0.333293763 | -0.418375221 | 0.038686202 | 0.176409082 | 10 |
| hsa04330 Notch signaling pathway | 0.32867722 | -0.385076473 | 0.049658881 | 0.217735092 | 13 |
| hsa00380 Tryptophan metabolism | 0.366118955 | -0.314256078 | 0.090226156 | 0.380954879 | 11 |
| hsa04020 Calcium signaling pathway | 0.376159732 | -0.295711026 | 0.100225172 | 0.398423199 | 38 |
| hsa03015 mRNA surveillance pathway | 0.370820501 | -0.297152628 | 0.10135327 | 0.398423199 | 20 |
| hsa04114 Oocyte meiosis | 0.387222526 | -0.265090918 | 0.1254573 | 0.475237309 | 41 |
| hsa04110 Cell cycle | 0.355021303 | -0.261309618 | 0.129231198 | 0.475237309 | 41 |
| hsa00982 Drug metabolism - cytochrome P450 | 0.383153567 | -0.258736332 | 0.135339526 | 0.48214706 | 10 |
| hsa00350 Tyrosine metabolism | 0.40522162 | -0.222558116 | 0.172720364 | 0.596670348 | 11 |
| hsa04350 TGF-beta signaling pathway | 0.404982156 | -0.209497793 | 0.183717943 | 0.615995455 | 23 |
| hsa00310 Lysine degradation | 0.406758356 | -0.199252793 | 0.195994524 | 0.638382163 | 14 |
| hsa03022 Basal transcription factors | 0.423949328 | -0.159747559 | 0.248884795 | 0.788135185 | 13 |
| hsa04720 Long-term potentiation | 0.442021952 | -0.133416835 | 0.282576117 | 0.854653447 | 23 |
| hsa04070 Phosphatidylinositol signaling system | 0.437884934 | -0.132137598 | 0.284884482 | 0.854653447 | 21 |
| hsa00640 Propanoate metabolism | 0.437630008 | -0.125014024 | 0.295583528 | 0.864013388 | 12 |
| hsa00561 Glycerolipid metabolism | 0.454933697 | -0.099841366 | 0.332971466 | 0.948968679 | 21 |
| hsa00534 Glycosaminoglycan biosynthesis - heparan sulfate | 0.455986632 | -0.093792889 | 0.344822571 | 0.958774953 | 13 |
| hsa04740 Olfactory transduction | 0.447156016 | -0.078865103 | 0.367229157 | 0.996764854 | 26 |
| hsa04970 Salivary secretion | 0.466445299 | -0.071986586 | 0.378368192 | 1 | 16 |
| hsa04340 Hedgehog signaling pathway | 0.475748551 | -0.051318852 | 0.413763138 | 1 | 11 |
| hsa04012 ErbB signaling pathway | 0.476830067 | -0.041425859 | 0.428975414 | 1 | 28 |
| hsa00071 Fatty acid metabolism | 0.459233451 | -0.03007917 | 0.450076052 | 1 | 14 |
| hsa04972 Pancreatic secretion | 0.484830862 | -0.025481841 | 0.456193769 | 1 | 20 |
| hsa04916 Melanogenesis | 0.488260643 | -0.007438543 | 0.487244583 | 1 | 25 |
| hsa00565 Ether lipid metabolism | 0.490277284 | -0.006552329 | 0.489045254 | 1 | 11 |
| hsa04962 Vasopressin-regulated water reabsorption | 0.492493948 | 0.002697565 | 0.504652114 | 1 | 15 |
| hsa04975 Fat digestion and absorption | 0.494143544 | 0.013198276 | 0.522571767 | 1 | 11 |
| hsa04640 Hematopoietic cell lineage | 0.475761832 | 0.025942863 | 0.544513688 | 1 | 49 |
| hsa00330 Arginine and proline metabolism | 0.472275028 | 0.027380649 | 0.547884361 | 1 | 17 |
| hsa04912 GnRH signaling pathway | 0.506558087 | 0.033999205 | 0.558437596 | 1 | 28 |
| hsa00480 Glutathione metabolism | 0.469997712 | 0.053231095 | 0.590846837 | 1 | 18 |
| hsa04320 Dorso-ventral axis formation | 0.503600092 | 0.056790719 | 0.593632395 | 1 | 10 |
| hsa04130 SNARE interactions in vesicular transport | 0.506161127 | 0.059400836 | 0.600831611 | 1 | 17 |
| hsa04512 ECM-receptor interaction | 0.481232165 | 0.061837572 | 0.607102114 | 1 | 17 |
| hsa04914 Progesterone-mediated oocyte maturation | 0.523878029 | 0.103737445 | 0.673499311 | 1 | 37 |
| hsa04623 Cytosolic DNA-sensing pathway | 0.455529536 | 0.124878059 | 0.699737564 | 1 | 17 |
| hsa04974 Protein digestion and absorption | 0.497807315 | 0.148973549 | 0.738874723 | 1 | 20 |
| hsa04270 Vascular smooth muscle contraction | 0.5332949 | 0.175943223 | 0.776944777 | 1 | 32 |
| hsa00562 Inositol phosphate metabolism | 0.550981155 | 0.184126116 | 0.784779288 | 1 | 16 |
| hsa04622 RIG-I-like receptor signaling pathway | 0.528116453 | 0.230038908 | 0.839250687 | 1 | 19 |
| hsa04146 Peroxisome | 0.567072048 | 0.23346118 | 0.843943654 | 1 | 27 |
| hsa04960 Aldosterone-regulated sodium reabsorption | 0.592768032 | 0.265854694 | 0.869780984 | 1 | 10 |
| hsa03050 Proteasome | 0.557960616 | 0.268075669 | 0.872941234 | 1 | 15 |
| hsa04141 Protein processing in endoplasmic reticulum | 0.564622289 | 0.316661248 | 0.914809938 | 1 | 55 |
| hsa00532 Glycosaminoglycan biosynthesis - chondroitin sulfate | 0.613941996 | 0.324222579 | 0.916604759 | 1 | 10 |
| hsa04662 B cell receptor signaling pathway | 0.524227981 | 0.340558377 | 0.929918866 | 1 | 36 |
| hsa04310 Wnt signaling pathway | 0.627708378 | 0.394569796 | 0.956340469 | 1 | 33 |
| hsa04150 mTOR signaling pathway | 0.643424277 | 0.397427429 | 0.95649331 | 1 | 17 |
| hsa00860 Porphyrin and chlorophyll metabolism | 0.565545027 | 0.421130695 | 0.962434565 | 1 | 11 |
| hsa04540 Gap junction | 0.62150259 | 0.433600064 | 0.969569252 | 1 | 31 |
| hsa00600 Sphingolipid metabolism | 0.652074913 | 0.447575713 | 0.972028745 | 1 | 15 |
| hsa04115 p53 signaling pathway | 0.646496094 | 0.450529732 | 0.97422804 | 1 | 28 |
| hsa00983 Drug metabolism - other enzymes | 0.659874293 | 0.458430004 | 0.97481115 | 1 | 11 |
| hsa04630 Jak-STAT signaling pathway | 0.558502179 | 0.500166708 | 0.98463876 | 1 | 50 |
| hsa00590 Arachidonic acid metabolism | 0.646474592 | 0.510081145 | 0.985645267 | 1 | 15 |
| hsa00520 Amino sugar and nucleotide sugar metabolism | 0.679184835 | 0.518430093 | 0.987116769 | 1 | 18 |
| hsa00564 Glycerophospholipid metabolism | 0.683459054 | 0.531137092 | 0.98901279 | 1 | 27 |
| hsa04730 Long-term depression | 0.686237904 | 0.56216917 | 0.99230334 | 1 | 20 |
| hsa04370 VEGF signaling pathway | 0.702699545 | 0.582740232 | 0.994076099 | 1 | 25 |
| hsa04621 NOD-like receptor signaling pathway | 0.65854571 | 0.624613615 | 0.996373301 | 1 | 30 |
| hsa04360 Axon guidance | 0.704632114 | 0.634750607 | 0.997011233 | 1 | 39 |
| hsa04610 Complement and coagulation cascades | 0.711659505 | 0.659420134 | 0.997577239 | 1 | 16 |
| hsa04973 Carbohydrate digestion and absorption | 0.744492304 | 0.701405749 | 0.998592039 | 1 | 11 |
| hsa00052 Galactose metabolism | 0.74752235 | 0.731257184 | 0.999063766 | 1 | 11 |
| hsa04144 Endocytosis | 0.719766219 | 0.738460837 | 0.999318565 | 1 | 67 |
| hsa03320 PPAR signaling pathway | 0.694428157 | 0.750264896 | 0.999330444 | 1 | 19 |
| hsa04976 Bile secretion | 0.744542264 | 0.762650629 | 0.999469608 | 1 | 19 |
| hsa04971 Gastric acid secretion | 0.752953653 | 0.761693351 | 0.999470832 | 1 | 21 |
| hsa04722 Neurotrophin signaling pathway | 0.75834553 | 0.798404883 | 0.999727112 | 1 | 49 |
| hsa04145 Phagosome | 0.626506377 | 0.840558448 | 0.999860557 | 1 | 72 |
| hsa04210 Apoptosis | 0.752340516 | 0.849188966 | 0.999878818 | 1 | 42 |
| hsa00010 Glycolysis / Gluconeogenesis | 0.742930964 | 0.881968186 | 0.999926396 | 1 | 30 |
| hsa04530 Tight junction | 0.782808536 | 0.889621278 | 0.999940656 | 1 | 41 |
| hsa04910 Insulin signaling pathway | 0.796855098 | 0.922536153 | 0.999967744 | 1 | 50 |
| hsa00030 Pentose phosphate pathway | 0.727141546 | 1.000753343 | 0.999985395 | 1 | 13 |
| hsa04920 Adipocytokine signaling pathway | 0.794788087 | 0.980627912 | 0.99998773 | 1 | 27 |
| hsa00051 Fructose and mannose metabolism | 0.813247994 | 1.004180356 | 0.999990366 | 1 | 15 |
| hsa04520 Adherens junction | 0.819767155 | 0.993747894 | 0.999990863 | 1 | 31 |
| hsa04062 Chemokine signaling pathway | 0.767920201 | 1.031280151 | 0.99999596 | 1 | 72 |
| hsa00500 Starch and sucrose metabolism | 0.837331324 | 1.060429956 | 0.999996538 | 1 | 12 |
| hsa04010 MAPK signaling pathway | 0.81590566 | 1.078335 | 0.999998581 | 1 | 97 |
| hsa04510 Focal adhesion | 0.835612855 | 1.109655708 | 0.999999241 | 1 | 64 |
| hsa04620 Toll-like receptor signaling pathway | 0.81025081 | 1.141764999 | 0.999999544 | 1 | 36 |
| hsa04142 Lysosome | 0.79660643 | 1.186075718 | 0.999999847 | 1 | 51 |
| hsa04664 Fc epsilon RI signaling pathway | 0.814987923 | 1.199401081 | 0.999999857 | 1 | 26 |
| hsa04650 Natural killer cell mediated cytotoxicity | 0.815759204 | 1.368939285 | 0.999999998 | 1 | 44 |
| hsa04666 Fc gamma R-mediated phagocytosis | 0.907783818 | 1.649307533 | 1 | 1 | 47 |
| hsa04670 Leukocyte transendothelial migration | 0.898114304 | 1.760501027 | 1 | 1 | 40 |
| hsa04380 Osteoclast differentiation | 0.777852778 | 1.784762018 | 1 | 1 | 65 |
| hsa04810 Regulation of actin cytoskeleton | 0.916930682 | 1.900312857 | 1 | 1 | 71 |
| hsa00040 Pentose and glucuronate interconversions | NA | NA | NA | NA | 6 |
| hsa00053 Ascorbate and aldarate metabolism | NA | NA | NA | NA | 4 |
| hsa00061 Fatty acid biosynthesis | NA | NA | NA | NA | 2 |
| hsa00072 Synthesis and degradation of ketone bodies | NA | NA | NA | NA | 1 |
| hsa00100 Steroid biosynthesis | NA | NA | NA | NA | 6 |
| hsa00120 Primary bile acid biosynthesis | NA | NA | NA | NA | 2 |
| hsa00130 Ubiquinone and other terpenoid-quinone biosynthesis | NA | NA | NA | NA | 4 |
| hsa00140 Steroid hormone biosynthesis | NA | NA | NA | NA | 5 |
| hsa00232 Caffeine metabolism | NA | NA | NA | NA | 0 |
| hsa00250 Alanine, aspartate and glutamate metabolism | NA | NA | NA | NA | 7 |
| hsa00260 Glycine, serine and threonine metabolism | NA | NA | NA | NA | 9 |
| hsa00270 Cysteine and methionine metabolism | NA | NA | NA | NA | 9 |
| hsa00290 Valine, leucine and isoleucine biosynthesis | NA | NA | NA | NA | 7 |
| hsa00300 Lysine biosynthesis | NA | NA | NA | NA | 0 |
| hsa00360 Phenylalanine metabolism | NA | NA | NA | NA | 5 |
| hsa00400 Phenylalanine, tyrosine and tryptophan biosynthesis | NA | NA | NA | NA | 1 |
| hsa00410 beta-Alanine metabolism | NA | NA | NA | NA | 7 |
| hsa00430 Taurine and hypotaurine metabolism | NA | NA | NA | NA | 1 |
| hsa00450 Selenocompound metabolism | NA | NA | NA | NA | 6 |
| hsa00460 Cyanoamino acid metabolism | NA | NA | NA | NA | 3 |
| hsa00471 D-Glutamine and D-glutamate metabolism | NA | NA | NA | NA | 0 |
| hsa00472 D-Arginine and D-ornithine metabolism | NA | NA | NA | NA | 0 |
| hsa00511 Other glycan degradation | NA | NA | NA | NA | 2 |
| hsa00512 Mucin type O-Glycan biosynthesis | NA | NA | NA | NA | 8 |
| hsa00531 Glycosaminoglycan degradation | NA | NA | NA | NA | 7 |
| hsa00533 Glycosaminoglycan biosynthesis - keratan sulfate | NA | NA | NA | NA | 4 |
| hsa00563 Glycosylphosphatidylinositol(GPI)-anchor biosynthesis | NA | NA | NA | NA | 5 |
| hsa00591 Linoleic acid metabolism | NA | NA | NA | NA | 4 |
| hsa00592 alpha-Linolenic acid metabolism | NA | NA | NA | NA | 3 |
| hsa00601 Glycosphingolipid biosynthesis - lacto and neolacto series | NA | NA | NA | NA | 9 |
| hsa00603 Glycosphingolipid biosynthesis - globo series | NA | NA | NA | NA | 4 |
| hsa00604 Glycosphingolipid biosynthesis - ganglio series | NA | NA | NA | NA | 6 |
| hsa00630 Glyoxylate and dicarboxylate metabolism | NA | NA | NA | NA | 8 |
| hsa00650 Butanoate metabolism | NA | NA | NA | NA | 7 |
| hsa00670 One carbon pool by folate | NA | NA | NA | NA | 5 |
| hsa00730 Thiamine metabolism | NA | NA | NA | NA | 1 |
| hsa00740 Riboflavin metabolism | NA | NA | NA | NA | 5 |
| hsa00750 Vitamin B6 metabolism | NA | NA | NA | NA | 2 |
| hsa00760 Nicotinate and nicotinamide metabolism | NA | NA | NA | NA | 5 |
| hsa00770 Pantothenate and CoA biosynthesis | NA | NA | NA | NA | 8 |
| hsa00780 Biotin metabolism | NA | NA | NA | NA | 0 |
| hsa00785 Lipoic acid metabolism | NA | NA | NA | NA | 2 |
| hsa00790 Folate biosynthesis | NA | NA | NA | NA | 3 |
| hsa00830 Retinol metabolism | NA | NA | NA | NA | 5 |
| hsa00900 Terpenoid backbone biosynthesis | NA | NA | NA | NA | 4 |
| hsa00910 Nitrogen metabolism | NA | NA | NA | NA | 5 |
| hsa00920 Sulfur metabolism | NA | NA | NA | NA | 6 |
| hsa00980 Metabolism of xenobiotics by cytochrome P450 | NA | NA | NA | NA | 9 |
| hsa01040 Biosynthesis of unsaturated fatty acids | NA | NA | NA | NA | 9 |
| hsa02010 ABC transporters | NA | NA | NA | NA | 7 |
| hsa03020 RNA polymerase | NA | NA | NA | NA | 8 |
| hsa03060 Protein export | NA | NA | NA | NA | 7 |
| hsa03430 Mismatch repair | NA | NA | NA | NA | 7 |
| hsa03450 Non-homologous end-joining | NA | NA | NA | NA | 3 |
| hsa04122 Sulfur relay system | NA | NA | NA | NA | 1 |
| hsa04140 Regulation of autophagy | NA | NA | NA | NA | 6 |
| hsa04614 Renin-angiotensin system | NA | NA | NA | NA | 5 |
| hsa04710 Circadian rhythm - mammal | NA | NA | NA | NA | 5 |
| hsa04742 Taste transduction | NA | NA | NA | NA | 8 |
| hsa04744 Phototransduction | NA | NA | NA | NA | 4 |
| hsa04964 Proximal tubule bicarbonate reclamation | NA | NA | NA | NA | 4 |
| hsa04966 Collecting duct acid secretion | NA | NA | NA | NA | 9 |
| hsa04977 Vitamin digestion and absorption | NA | NA | NA | NA | 4 |
|  |  |  |  |  |  |
| **GSE66099** | **p.geomean** | **stat.mean** | **p.val** | **q.val** | **set.**  **size** |
| hsa04610 Complement and coagulation cascades | 0.003705049 | 2.627115289 | 3.01E-68 | 3.85E-66 | 27 |
| hsa04142 Lysosome | 0.01266978 | 2.027149078 | 2.33E-44 | 1.49E-42 | 59 |
| hsa04380 Osteoclast differentiation | 0.012269396 | 1.948024403 | 3.73E-41 | 1.59E-39 | 65 |
| hsa04810 Regulation of actin cytoskeleton | 0.017974084 | 1.886943364 | 3.68E-39 | 1.18E-37 | 77 |
| hsa04666 Fc gamma R-mediated phagocytosis | 0.029131543 | 1.692698376 | 1.66E-31 | 4.26E-30 | 45 |
| hsa03320 PPAR signaling pathway | 0.050250178 | 1.597233745 | 6.17E-28 | 1.32E-26 | 24 |
| hsa04621 NOD-like receptor signaling pathway | 0.049312688 | 1.507315834 | 5.97E-25 | 1.07E-23 | 25 |
| hsa04510 Focal adhesion | 0.042514625 | 1.478517677 | 6.67E-25 | 1.07E-23 | 76 |
| hsa00010 Glycolysis / Gluconeogenesis | 0.065143465 | 1.438619389 | 2.31E-23 | 3.28E-22 | 31 |
| hsa00983 Drug metabolism - other enzymes | 0.050625477 | 1.452537004 | 8.92E-23 | 1.14E-21 | 23 |
| hsa04620 Toll-like receptor signaling pathway | 0.054148099 | 1.404108552 | 3.23E-22 | 3.76E-21 | 35 |
| hsa04910 Insulin signaling pathway | 0.060899682 | 1.37837677 | 6.64E-22 | 7.08E-21 | 61 |
| hsa04145 Phagosome | 0.052262049 | 1.302658365 | 1.04E-19 | 1.02E-18 | 73 |
| hsa04512 ECM-receptor interaction | 0.060938568 | 1.290258349 | 6.09E-19 | 5.57E-18 | 33 |
| hsa04740 Olfactory transduction | 0.043112696 | 1.286221584 | 2.75E-18 | 2.29E-17 | 29 |
| hsa00052 Galactose metabolism | 0.093603146 | 1.272071517 | 2.87E-18 | 2.29E-17 | 16 |
| hsa00531 Glycosaminoglycan degradation | 0.098164887 | 1.257329942 | 7.38E-17 | 5.55E-16 | 10 |
| hsa00051 Fructose and mannose metabolism | 0.11236772 | 1.172167665 | 6.24E-16 | 4.44E-15 | 17 |
| hsa00480 Glutathione metabolism | 0.115759676 | 1.110178902 | 1.18E-14 | 7.92E-14 | 24 |
| hsa04920 Adipocytokine signaling pathway | 0.121086301 | 1.089798802 | 2.54E-14 | 1.58E-13 | 33 |
| hsa00190 Oxidative phosphorylation | 0.105760971 | 1.089125289 | 2.60E-14 | 1.58E-13 | 44 |
| hsa04020 Calcium signaling pathway | 0.098336386 | 1.071985624 | 5.23E-14 | 3.04E-13 | 64 |
| hsa00982 Drug metabolism - cytochrome P450 | 0.081431459 | 1.087563285 | 1.44E-13 | 7.93E-13 | 23 |
| hsa04010 MAPK signaling pathway | 0.10579043 | 1.04808766 | 1.49E-13 | 7.93E-13 | 98 |
| hsa04670 Leukocyte transendothelial migration | 0.107371889 | 1.039236046 | 3.31E-13 | 1.70E-12 | 46 |
| hsa04144 Endocytosis | 0.115257715 | 0.971773613 | 6.94E-12 | 3.42E-11 | 81 |
| hsa00564 Glycerophospholipid metabolism | 0.129797271 | 0.976771438 | 7.98E-12 | 3.78E-11 | 31 |
| hsa00140 Steroid hormone biosynthesis | 0.108621819 | 0.999920789 | 8.37E-12 | 3.83E-11 | 18 |
| hsa00860 Porphyrin and chlorophyll metabolism | 0.134304384 | 0.982959373 | 1.26E-11 | 5.54E-11 | 19 |
| hsa00980 Metabolism of xenobiotics by cytochrome P450 | 0.121269955 | 0.973182501 | 1.51E-11 | 6.46E-11 | 23 |
| hsa00600 Sphingolipid metabolism | 0.143705695 | 0.958750688 | 3.00E-11 | 1.24E-10 | 17 |
| hsa00030 Pentose phosphate pathway | 0.150939894 | 0.952746064 | 3.82E-11 | 1.53E-10 | 17 |
| hsa04320 Dorso-ventral axis formation | 0.160274512 | 0.935747774 | 1.42E-10 | 5.52E-10 | 11 |
| hsa04540 Gap junction | 0.140440959 | 0.894186191 | 3.45E-10 | 1.30E-09 | 36 |
| hsa04114 Oocyte meiosis | 0.133707655 | 0.888103923 | 3.92E-10 | 1.43E-09 | 50 |
| hsa04130 SNARE interactions in vesicular transport | 0.150579419 | 0.883380558 | 1.01E-09 | 3.61E-09 | 16 |
| hsa04270 Vascular smooth muscle contraction | 0.176069445 | 0.8137631 | 8.35E-09 | 2.89E-08 | 41 |
| hsa04912 GnRH signaling pathway | 0.170971979 | 0.808486958 | 1.05E-08 | 3.55E-08 | 39 |
| hsa04914 Progesterone-mediated oocyte maturation | 0.172100632 | 0.804150142 | 1.26E-08 | 4.13E-08 | 42 |
| hsa00512 Mucin type O-Glycan biosynthesis | 0.19059102 | 0.810585824 | 1.52E-08 | 4.85E-08 | 14 |
| hsa04722 Neurotrophin signaling pathway | 0.165526478 | 0.795151256 | 1.72E-08 | 5.37E-08 | 49 |
| hsa00500 Starch and sucrose metabolism | 0.129188063 | 0.76812641 | 8.44E-08 | 2.57E-07 | 29 |
| hsa00760 Nicotinate and nicotinamide metabolism | 0.211669604 | 0.753077767 | 1.68E-07 | 5.02E-07 | 10 |
| hsa04966 Collecting duct acid secretion | 0.207210052 | 0.740427687 | 2.33E-07 | 6.77E-07 | 12 |
| hsa00330 Arginine and proline metabolism | 0.208270356 | 0.729924788 | 2.39E-07 | 6.79E-07 | 23 |
| hsa04141 Protein processing in endoplasmic reticulum | 0.189196825 | 0.715726473 | 3.23E-07 | 8.99E-07 | 60 |
| hsa04115 p53 signaling pathway | 0.212291319 | 0.695100274 | 7.24E-07 | 1.97E-06 | 35 |
| hsa04370 VEGF signaling pathway | 0.200775123 | 0.695719348 | 7.84E-07 | 2.09E-06 | 29 |
| hsa04622 RIG-I-like receptor signaling pathway | 0.224190694 | 0.683065578 | 1.29E-06 | 3.32E-06 | 18 |
| hsa04976 Bile secretion | 0.196657044 | 0.683424679 | 1.30E-06 | 3.32E-06 | 24 |
| hsa04630 Jak-STAT signaling pathway | 0.200846678 | 0.648508842 | 3.25E-06 | 8.15E-06 | 59 |
| hsa00520 Amino sugar and nucleotide sugar metabolism | 0.222656696 | 0.622511145 | 8.59E-06 | 2.11E-05 | 29 |
| hsa04360 Axon guidance | 0.21054589 | 0.591536051 | 2.00E-05 | 4.82E-05 | 52 |
| hsa04260 Cardiac muscle contraction | 0.235501332 | 0.590618725 | 2.36E-05 | 5.59E-05 | 24 |
| hsa04975 Fat digestion and absorption | 0.25565959 | 0.578036426 | 4.33E-05 | 0.000100817 | 10 |
| hsa04664 Fc epsilon RI signaling pathway | 0.234623663 | 0.560978645 | 5.14E-05 | 0.000117457 | 31 |
| hsa04210 Apoptosis | 0.234016116 | 0.555656094 | 5.64E-05 | 0.000126666 | 46 |
| hsa00830 Retinol metabolism | 0.169950294 | 0.563733787 | 5.83E-05 | 0.000128713 | 26 |
| hsa03050 Proteasome | 0.23694637 | 0.543064357 | 0.000124335 | 0.000266461 | 13 |
| hsa04530 Tight junction | 0.263604072 | 0.526600436 | 0.000124904 | 0.000266461 | 44 |
| hsa00514 Other types of O-glycan biosynthesis | 0.237020502 | 0.4881476 | 0.000418067 | 0.000877256 | 18 |
| hsa04012 ErbB signaling pathway | 0.275335156 | 0.460884513 | 0.00069433 | 0.001433455 | 33 |
| hsa00310 Lysine degradation | 0.302109833 | 0.463259641 | 0.000740288 | 0.001504077 | 13 |
| hsa00380 Tryptophan metabolism | 0.307501964 | 0.448949223 | 0.001038137 | 0.002076273 | 13 |
| hsa04971 Gastric acid secretion | 0.280461857 | 0.439202628 | 0.001193463 | 0.002350204 | 26 |
| hsa04110 Cell cycle | 0.174672074 | 0.438409807 | 0.001290934 | 0.00250363 | 67 |
| hsa00053 Ascorbate and aldarate metabolism | 0.244022689 | 0.446737097 | 0.001436722 | 0.002744782 | 12 |
| hsa04520 Adherens junction | 0.280661701 | 0.415702695 | 0.001965147 | 0.0036991 | 31 |
| hsa04916 Melanogenesis | 0.301552839 | 0.362558429 | 0.00600789 | 0.011145072 | 33 |
| hsa00565 Ether lipid metabolism | 0.323048551 | 0.361186826 | 0.006930128 | 0.012672234 | 11 |
| hsa04962 Vasopressin-regulated water reabsorption | 0.334798032 | 0.341723509 | 0.009086183 | 0.016380725 | 19 |
| hsa00040 Pentose and glucuronate interconversions | 0.289787808 | 0.345056996 | 0.009290416 | 0.016516295 | 15 |
| hsa00561 Glycerolipid metabolism | 0.335207685 | 0.332713947 | 0.010704146 | 0.018768913 | 19 |
| hsa04150 mTOR signaling pathway | 0.337606934 | 0.320797255 | 0.01321614 | 0.022860349 | 23 |
| hsa04742 Taste transduction | 0.291028874 | 0.318718039 | 0.014402291 | 0.024579911 | 16 |
| hsa04730 Long-term depression | 0.332568569 | 0.306091725 | 0.017434524 | 0.029363409 | 21 |
| hsa00590 Arachidonic acid metabolism | 0.343245314 | 0.263932337 | 0.034058501 | 0.056616729 | 20 |
| hsa04623 Cytosolic DNA-sensing pathway | 0.354542664 | 0.24402704 | 0.04715838 | 0.077388111 | 15 |
| hsa04330 Notch signaling pathway | 0.35982807 | 0.192659157 | 0.091907442 | 0.148913325 | 20 |
| hsa03440 Homologous recombination | 0.374767582 | 0.189320952 | 0.098329508 | 0.157327212 | 14 |
| hsa04062 Chemokine signaling pathway | 0.292295649 | 0.15879599 | 0.133791923 | 0.211424273 | 89 |
| hsa04960 Aldosterone-regulated sodium reabsorption | 0.390562423 | 0.147898997 | 0.1562751 | 0.243941619 | 13 |
| hsa04973 Carbohydrate digestion and absorption | 0.331954464 | 0.146179308 | 0.158477206 | 0.244398583 | 21 |
| hsa04146 Peroxisome | 0.372717362 | 0.119390199 | 0.204114911 | 0.311032246 | 38 |
| hsa00534 Glycosaminoglycan biosynthesis - heparan sulfate | 0.442049424 | 0.093139652 | 0.261343285 | 0.393552241 | 10 |
| hsa00532 Glycosaminoglycan biosynthesis - chondroitin sulfate | 0.437849575 | 0.066691854 | 0.322068246 | 0.479357389 | 12 |
| hsa04720 Long-term potentiation | 0.440235062 | 0.039588775 | 0.391737188 | 0.576348967 | 24 |
| hsa04972 Pancreatic secretion | 0.428445132 | 0.032656772 | 0.409181868 | 0.595173626 | 33 |
| hsa00071 Fatty acid metabolism | 0.413265823 | 0.022840238 | 0.437035673 | 0.628545687 | 20 |
| hsa04974 Protein digestion and absorption | 0.400587787 | -0.00438383 | 0.512075141 | 0.728284645 | 19 |
| hsa04310 Wnt signaling pathway | 0.437700838 | -0.072120803 | 0.692043725 | 0.973424141 | 57 |
| hsa04662 B cell receptor signaling pathway | 0.36103007 | -0.07710529 | 0.703307374 | 0.978514608 | 39 |
| hsa04970 Salivary secretion | 0.512955548 | -0.202513164 | 0.919414566 | 1 | 30 |
| hsa00562 Inositol phosphate metabolism | 0.545419933 | -0.209184316 | 0.925845588 | 1 | 20 |
| hsa00340 Histidine metabolism | 0.544360095 | -0.232828907 | 0.943784408 | 1 | 11 |
| hsa00350 Tyrosine metabolism | 0.592645522 | -0.30800538 | 0.982976014 | 1 | 14 |
| hsa00240 Pyrimidine metabolism | 0.535418227 | -0.304257045 | 0.983005963 | 1 | 45 |
| hsa00640 Propanoate metabolism | 0.567389887 | -0.32318709 | 0.9869299 | 1 | 15 |
| hsa02010 ABC transporters | 0.571371839 | -0.33534975 | 0.98958063 | 1 | 19 |
| hsa04120 Ubiquitin mediated proteolysis | 0.477484887 | -0.33461628 | 0.990023117 | 1 | 60 |
| hsa04350 TGF-beta signaling pathway | 0.586336612 | -0.347386422 | 0.99214281 | 1 | 38 |
| hsa00620 Pyruvate metabolism | 0.543545294 | -0.363893801 | 0.993642594 | 1 | 16 |
| hsa00020 Citrate cycle (TCA cycle) | 0.548225146 | -0.373598925 | 0.994268151 | 1 | 10 |
| hsa00270 Cysteine and methionine metabolism | 0.582681441 | -0.366283307 | 0.994290773 | 1 | 18 |
| hsa04070 Phosphatidylinositol signaling system | 0.621304624 | -0.456833384 | 0.999213644 | 1 | 26 |
| hsa04640 Hematopoietic cell lineage | 0.560465997 | -0.468457781 | 0.999437017 | 1 | 51 |
| hsa00230 Purine metabolism | 0.624657536 | -0.501315713 | 0.999763164 | 1 | 71 |
| hsa03060 Protein export | 0.573804743 | -0.547418273 | 0.999875767 | 1 | 12 |
| hsa00563 Glycosylphosphatidylinositol(GPI)-anchor biosynthesis | 0.679893069 | -0.765756219 | 0.999999882 | 1 | 12 |
| hsa03410 Base excision repair | 0.649822419 | -0.782178347 | 0.999999944 | 1 | 15 |
| hsa03022 Basal transcription factors | 0.694202219 | -0.787481501 | 0.999999947 | 1 | 13 |
| hsa03430 Mismatch repair | 0.672068333 | -0.802861444 | 0.999999969 | 1 | 12 |
| hsa03015 mRNA surveillance pathway | 0.738013337 | -0.875760598 | 0.999999999 | 1 | 40 |
| hsa00510 N-Glycan biosynthesis | 0.735702206 | -0.926550133 | 1 | 1 | 20 |
| hsa00280 Valine, leucine and isoleucine degradation | 0.684717543 | -0.95942875 | 1 | 1 | 20 |
| hsa03420 Nucleotide excision repair | 0.637905123 | -0.963139829 | 1 | 1 | 22 |
| hsa03030 DNA replication | 0.610906963 | -0.976498889 | 1 | 1 | 18 |
| hsa00970 Aminoacyl-tRNA biosynthesis | 0.708900242 | -0.989717566 | 1 | 1 | 13 |
| hsa04514 Cell adhesion molecules (CAMs) | 0.664625853 | -1.052692299 | 1 | 1 | 59 |
| hsa04660 T cell receptor signaling pathway | 0.665854995 | -1.108616638 | 1 | 1 | 56 |
| hsa04650 Natural killer cell mediated cytotoxicity | 0.654357518 | -1.116430068 | 1 | 1 | 49 |
| hsa03008 Ribosome biogenesis in eukaryotes | 0.616574079 | -1.913281458 | 1 | 1 | 48 |
| hsa03010 Ribosome | 0.478525766 | -1.58007293 | 1 | 1 | 52 |
| hsa03013 RNA transport | 0.628940109 | -2.356686231 | 1 | 1 | 78 |
| hsa03018 RNA degradation | 0.722605049 | -1.260348082 | 1 | 1 | 35 |
| hsa03040 Spliceosome | 0.682745561 | -1.611086829 | 1 | 1 | 58 |
| hsa04612 Antigen processing and presentation | 0.834803569 | -2.239990543 | 1 | 1 | 40 |
| hsa04672 Intestinal immune network for IgA production | 0.790429155 | -1.866945746 | 1 | 1 | 31 |
| hsa00061 Fatty acid biosynthesis | NA | NA | NA | NA | 2 |
| hsa00072 Synthesis and degradation of ketone bodies | NA | NA | NA | NA | 3 |
| hsa00100 Steroid biosynthesis | NA | NA | NA | NA | 6 |
| hsa00120 Primary bile acid biosynthesis | NA | NA | NA | NA | 3 |
| hsa00130 Ubiquinone and other terpenoid-quinone biosynthesis | NA | NA | NA | NA | 4 |
| hsa00232 Caffeine metabolism | NA | NA | NA | NA | 1 |
| hsa00250 Alanine, aspartate and glutamate metabolism | NA | NA | NA | NA | 9 |
| hsa00260 Glycine, serine and threonine metabolism | NA | NA | NA | NA | 7 |
| hsa00290 Valine, leucine and isoleucine biosynthesis | NA | NA | NA | NA | 3 |
| hsa00300 Lysine biosynthesis | NA | NA | NA | NA | 0 |
| hsa00360 Phenylalanine metabolism | NA | NA | NA | NA | 5 |
| hsa00400 Phenylalanine, tyrosine and tryptophan biosynthesis | NA | NA | NA | NA | 1 |
| hsa00410 beta-Alanine metabolism | NA | NA | NA | NA | 9 |
| hsa00430 Taurine and hypotaurine metabolism | NA | NA | NA | NA | 4 |
| hsa00450 Selenocompound metabolism | NA | NA | NA | NA | 9 |
| hsa00460 Cyanoamino acid metabolism | NA | NA | NA | NA | 2 |
| hsa00471 D-Glutamine and D-glutamate metabolism | NA | NA | NA | NA | 1 |
| hsa00472 D-Arginine and D-ornithine metabolism | NA | NA | NA | NA | 0 |
| hsa00511 Other glycan degradation | NA | NA | NA | NA | 4 |
| hsa00533 Glycosaminoglycan biosynthesis - keratan sulfate | NA | NA | NA | NA | 6 |
| hsa00591 Linoleic acid metabolism | NA | NA | NA | NA | 6 |
| hsa00592 alpha-Linolenic acid metabolism | NA | NA | NA | NA | 5 |
| hsa00601 Glycosphingolipid biosynthesis - lacto and neolacto series | NA | NA | NA | NA | 9 |
| hsa00603 Glycosphingolipid biosynthesis - globo series | NA | NA | NA | NA | 6 |
| hsa00604 Glycosphingolipid biosynthesis - ganglio series | NA | NA | NA | NA | 9 |
| hsa00630 Glyoxylate and dicarboxylate metabolism | NA | NA | NA | NA | 6 |
| hsa00650 Butanoate metabolism | NA | NA | NA | NA | 9 |
| hsa00670 One carbon pool by folate | NA | NA | NA | NA | 8 |
| hsa00730 Thiamine metabolism | NA | NA | NA | NA | 0 |
| hsa00740 Riboflavin metabolism | NA | NA | NA | NA | 5 |
| hsa00750 Vitamin B6 metabolism | NA | NA | NA | NA | 4 |
| hsa00770 Pantothenate and CoA biosynthesis | NA | NA | NA | NA | 6 |
| hsa00780 Biotin metabolism | NA | NA | NA | NA | 0 |
| hsa00785 Lipoic acid metabolism | NA | NA | NA | NA | 2 |
| hsa00790 Folate biosynthesis | NA | NA | NA | NA | 5 |
| hsa00900 Terpenoid backbone biosynthesis | NA | NA | NA | NA | 4 |
| hsa00910 Nitrogen metabolism | NA | NA | NA | NA | 7 |
| hsa00920 Sulfur metabolism | NA | NA | NA | NA | 5 |
| hsa01040 Biosynthesis of unsaturated fatty acids | NA | NA | NA | NA | 8 |
| hsa03020 RNA polymerase | NA | NA | NA | NA | 9 |
| hsa03450 Non-homologous end-joining | NA | NA | NA | NA | 5 |
| hsa04122 Sulfur relay system | NA | NA | NA | NA | 3 |
| hsa04140 Regulation of autophagy | NA | NA | NA | NA | 9 |
| hsa04340 Hedgehog signaling pathway | NA | NA | NA | NA | 9 |
| hsa04614 Renin-angiotensin system | NA | NA | NA | NA | 5 |
| hsa04710 Circadian rhythm - mammal | NA | NA | NA | NA | 7 |
| hsa04744 Phototransduction | NA | NA | NA | NA | 9 |
| hsa04964 Proximal tubule bicarbonate reclamation | NA | NA | NA | NA | 6 |
| hsa04977 Vitamin digestion and absorption | NA | NA | NA | NA | 8 |
| hsa03013 RNA transport | 0.00224852 | -2.356686231 | 4.45E-58 | 5.69E-56 | 78 |
| hsa04612 Antigen processing and presentation | 0.007962322 | -2.239990543 | 2.80E-52 | 1.79E-50 | 40 |
| hsa03008 Ribosome biogenesis in eukaryotes | 0.010169063 | -1.913281458 | 9.27E-39 | 3.95E-37 | 48 |
| hsa04672 Intestinal immune network for IgA production | 0.019476949 | -1.866945746 | 8.33E-37 | 2.67E-35 | 31 |
| hsa03040 Spliceosome | 0.023118923 | -1.611086829 | 1.93E-28 | 4.93E-27 | 58 |
| hsa03010 Ribosome | 0.014619546 | -1.58007293 | 3.80E-26 | 8.11E-25 | 52 |
| hsa03018 RNA degradation | 0.06964811 | -1.260348082 | 3.16E-18 | 5.78E-17 | 35 |
| hsa04650 Natural killer cell mediated cytotoxicity | 0.082410013 | -1.116430068 | 7.03E-15 | 1.12E-13 | 49 |
| hsa04660 T cell receptor signaling pathway | 0.084384899 | -1.108616638 | 8.87E-15 | 1.26E-13 | 56 |
| hsa04514 Cell adhesion molecules (CAMs) | 0.09348904 | -1.052692299 | 1.62E-13 | 2.07E-12 | 59 |
| hsa00970 Aminoacyl-tRNA biosynthesis | 0.133072857 | -0.989717566 | 2.16E-11 | 2.52E-10 | 13 |
| hsa03030 DNA replication | 0.109155318 | -0.976498889 | 2.36E-11 | 2.52E-10 | 18 |
| hsa03420 Nucleotide excision repair | 0.115046907 | -0.963139829 | 2.97E-11 | 2.93E-10 | 22 |
| hsa00280 Valine, leucine and isoleucine degradation | 0.128816843 | -0.95942875 | 3.21E-11 | 2.93E-10 | 20 |
| hsa00510 N-Glycan biosynthesis | 0.152023099 | -0.926550133 | 1.07E-10 | 9.17E-10 | 20 |
| hsa03015 mRNA surveillance pathway | 0.165892281 | -0.875760598 | 6.44E-10 | 5.15E-09 | 40 |
| hsa03430 Mismatch repair | 0.175299738 | -0.802861444 | 3.09E-08 | 2.32E-07 | 12 |
| hsa03022 Basal transcription factors | 0.188589289 | -0.787481501 | 5.30E-08 | 3.77E-07 | 13 |
| hsa03410 Base excision repair | 0.173489164 | -0.782178347 | 5.62E-08 | 3.78E-07 | 15 |
| hsa00563 Glycosylphosphatidylinositol(GPI)-anchor biosynthesis | 0.19078658 | -0.765756219 | 1.18E-07 | 7.55E-07 | 12 |
| hsa03060 Protein export | 0.232311418 | -0.547418273 | 0.000124233 | 0.000757228 | 12 |
| hsa00230 Purine metabolism | 0.270034866 | -0.501315713 | 0.000236836 | 0.001377954 | 71 |
| hsa04640 Hematopoietic cell lineage | 0.254676694 | -0.468457781 | 0.000562983 | 0.003133122 | 51 |
| hsa04070 Phosphatidylinositol signaling system | 0.291470638 | -0.456833384 | 0.000786356 | 0.004193896 | 26 |
| hsa00270 Cysteine and methionine metabolism | 0.321081727 | -0.366283307 | 0.005709227 | 0.028218335 | 18 |
| hsa00020 Citrate cycle (TCA cycle) | 0.298123638 | -0.373598925 | 0.005731849 | 0.028218335 | 10 |
| hsa00620 Pyruvate metabolism | 0.295943726 | -0.363893801 | 0.006357406 | 0.030138814 | 16 |
| hsa04350 TGF-beta signaling pathway | 0.330278115 | -0.347386422 | 0.00785719 | 0.035918582 | 38 |
| hsa04120 Ubiquitin mediated proteolysis | 0.268074308 | -0.33461628 | 0.009976883 | 0.044035897 | 60 |
| hsa02010 ABC transporters | 0.328365142 | -0.33534975 | 0.01041937 | 0.044455978 | 19 |
| hsa00640 Propanoate metabolism | 0.335237773 | -0.32318709 | 0.0130701 | 0.053966863 | 15 |
| hsa00240 Pyrimidine metabolism | 0.326085856 | -0.304257045 | 0.016994037 | 0.066032429 | 45 |
| hsa00350 Tyrosine metabolism | 0.360644035 | -0.30800538 | 0.017023986 | 0.066032429 | 14 |
| hsa00340 Histidine metabolism | 0.37328606 | -0.232828907 | 0.056215592 | 0.211635171 | 11 |
| hsa00562 Inositol phosphate metabolism | 0.387769636 | -0.209184316 | 0.074154412 | 0.271193278 | 20 |
| hsa04970 Salivary secretion | 0.365046672 | -0.202513164 | 0.080585434 | 0.286525988 | 30 |
| hsa04662 B cell receptor signaling pathway | 0.316557592 | -0.07710529 | 0.296692626 | 1 | 39 |
| hsa04310 Wnt signaling pathway | 0.386978715 | -0.072120803 | 0.307956275 | 1 | 57 |
| hsa04974 Protein digestion and absorption | 0.398761984 | -0.00438383 | 0.487924859 | 1 | 19 |
| hsa00071 Fatty acid metabolism | 0.428667747 | 0.022840238 | 0.562964327 | 1 | 20 |
| hsa04972 Pancreatic secretion | 0.449222421 | 0.032656772 | 0.590818132 | 1 | 33 |
| hsa04720 Long-term potentiation | 0.469431029 | 0.039588775 | 0.608262812 | 1 | 24 |
| hsa00532 Glycosaminoglycan biosynthesis - chondroitin sulfate | 0.486384279 | 0.066691854 | 0.677931754 | 1 | 12 |
| hsa00534 Glycosaminoglycan biosynthesis - heparan sulfate | 0.512465415 | 0.093139652 | 0.738656715 | 1 | 10 |
| hsa04146 Peroxisome | 0.457986174 | 0.119390199 | 0.795885089 | 1 | 38 |
| hsa04973 Carbohydrate digestion and absorption | 0.430683001 | 0.146179308 | 0.841522794 | 1 | 21 |
| hsa04960 Aldosterone-regulated sodium reabsorption | 0.497436247 | 0.147898997 | 0.8437249 | 1 | 13 |
| hsa04062 Chemokine signaling pathway | 0.380277652 | 0.15879599 | 0.866208077 | 1 | 89 |
| hsa03440 Homologous recombination | 0.513956383 | 0.189320952 | 0.901670492 | 1 | 14 |
| hsa04330 Notch signaling pathway | 0.494678063 | 0.192659157 | 0.908092558 | 1 | 20 |
| hsa04623 Cytosolic DNA-sensing pathway | 0.529832268 | 0.24402704 | 0.95284162 | 1 | 15 |
| hsa00590 Arachidonic acid metabolism | 0.528832108 | 0.263932337 | 0.965941499 | 1 | 20 |
| hsa04730 Long-term depression | 0.550441643 | 0.306091725 | 0.982565476 | 1 | 21 |
| hsa04742 Taste transduction | 0.495743941 | 0.318718039 | 0.985597709 | 1 | 16 |
| hsa04150 mTOR signaling pathway | 0.570644265 | 0.320797255 | 0.98678386 | 1 | 23 |
| hsa00561 Glycerolipid metabolism | 0.576153301 | 0.332713947 | 0.989295854 | 1 | 19 |
| hsa00040 Pentose and glucuronate interconversions | 0.513728483 | 0.345056996 | 0.990709584 | 1 | 15 |
| hsa04962 Vasopressin-regulated water reabsorption | 0.584312457 | 0.341723509 | 0.990913817 | 1 | 19 |
| hsa00565 Ether lipid metabolism | 0.580006712 | 0.361186826 | 0.993069872 | 1 | 11 |
| hsa04916 Melanogenesis | 0.556507152 | 0.362558429 | 0.99399211 | 1 | 33 |
| hsa04520 Adherens junction | 0.559181787 | 0.415702695 | 0.998034853 | 1 | 31 |
| hsa00053 Ascorbate and aldarate metabolism | 0.513443249 | 0.446737097 | 0.998563278 | 1 | 12 |
| hsa04110 Cell cycle | 0.435995418 | 0.438409807 | 0.998709066 | 1 | 67 |
| hsa04971 Gastric acid secretion | 0.58475622 | 0.439202628 | 0.998806537 | 1 | 26 |
| hsa00380 Tryptophan metabolism | 0.635066091 | 0.448949223 | 0.998961863 | 1 | 13 |
| hsa00310 Lysine degradation | 0.639111986 | 0.463259641 | 0.999259712 | 1 | 13 |
| hsa04012 ErbB signaling pathway | 0.592629829 | 0.460884513 | 0.99930567 | 1 | 33 |
| hsa00514 Other types of O-glycan biosynthesis | 0.540895545 | 0.4881476 | 0.999581933 | 1 | 18 |
| hsa04530 Tight junction | 0.633836273 | 0.526600436 | 0.999875096 | 1 | 44 |
| hsa03050 Proteasome | 0.591292167 | 0.543064357 | 0.999875665 | 1 | 13 |
| hsa00830 Retinol metabolism | 0.468670082 | 0.563733787 | 0.999941677 | 1 | 26 |
| hsa04210 Apoptosis | 0.601301566 | 0.555656094 | 0.999943594 | 1 | 46 |
| hsa04664 Fc epsilon RI signaling pathway | 0.604192424 | 0.560978645 | 0.999948613 | 1 | 31 |
| hsa04975 Fat digestion and absorption | 0.652024693 | 0.578036426 | 0.99995668 | 1 | 10 |
| hsa04260 Cardiac muscle contraction | 0.637269495 | 0.590618725 | 0.999976426 | 1 | 24 |
| hsa04360 Axon guidance | 0.586093277 | 0.591536051 | 0.999980033 | 1 | 52 |
| hsa00520 Amino sugar and nucleotide sugar metabolism | 0.646334324 | 0.622511145 | 0.999991412 | 1 | 29 |
| hsa04630 Jak-STAT signaling pathway | 0.616445633 | 0.648508842 | 0.999996753 | 1 | 59 |
| hsa04976 Bile secretion | 0.635464081 | 0.683424679 | 0.999998703 | 1 | 24 |
| hsa04622 RIG-I-like receptor signaling pathway | 0.693807151 | 0.683065578 | 0.999998707 | 1 | 18 |
| hsa04370 VEGF signaling pathway | 0.656028461 | 0.695719348 | 0.999999216 | 1 | 29 |
| hsa04115 p53 signaling pathway | 0.688345993 | 0.695100274 | 0.999999276 | 1 | 35 |
| hsa04141 Protein processing in endoplasmic reticulum | 0.653812985 | 0.715726473 | 0.999999677 | 1 | 60 |
| hsa00330 Arginine and proline metabolism | 0.707152267 | 0.729924788 | 0.999999761 | 1 | 23 |
| hsa04966 Collecting duct acid secretion | 0.697630823 | 0.740427687 | 0.999999767 | 1 | 12 |
| hsa00760 Nicotinate and nicotinamide metabolism | 0.720020429 | 0.753077767 | 0.999999832 | 1 | 10 |
| hsa00500 Starch and sucrose metabolism | 0.548600828 | 0.76812641 | 0.999999916 | 1 | 29 |
| hsa04722 Neurotrophin signaling pathway | 0.663390448 | 0.795151256 | 0.999999983 | 1 | 49 |
| hsa00512 Mucin type O-Glycan biosynthesis | 0.727336763 | 0.810585824 | 0.999999985 | 1 | 14 |
| hsa04914 Progesterone-mediated oocyte maturation | 0.696081054 | 0.804150142 | 0.999999987 | 1 | 42 |
| hsa04912 GnRH signaling pathway | 0.686409867 | 0.808486958 | 0.999999989 | 1 | 39 |
| hsa04270 Vascular smooth muscle contraction | 0.71107199 | 0.8137631 | 0.999999992 | 1 | 41 |
| hsa04130 SNARE interactions in vesicular transport | 0.686226057 | 0.883380558 | 0.999999999 | 1 | 16 |
| hsa04114 Oocyte meiosis | 0.662166189 | 0.888103923 | 1 | 1 | 50 |
| hsa04540 Gap junction | 0.68400657 | 0.894186191 | 1 | 1 | 36 |
| hsa04320 Dorso-ventral axis formation | 0.750619375 | 0.935747774 | 1 | 1 | 11 |
| hsa00030 Pentose phosphate pathway | 0.753237135 | 0.952746064 | 1 | 1 | 17 |
| hsa00600 Sphingolipid metabolism | 0.734002849 | 0.958750688 | 1 | 1 | 17 |
| hsa00980 Metabolism of xenobiotics by cytochrome P450 | 0.675621088 | 0.973182501 | 1 | 1 | 23 |
| hsa00860 Porphyrin and chlorophyll metabolism | 0.728729034 | 0.982959373 | 1 | 1 | 19 |
| hsa00140 Steroid hormone biosynthesis | 0.641876744 | 0.999920789 | 1 | 1 | 18 |
| hsa00564 Glycerophospholipid metabolism | 0.70791611 | 0.976771438 | 1 | 1 | 31 |
| hsa04144 Endocytosis | 0.6613336 | 0.971773613 | 1 | 1 | 81 |
| hsa04670 Leukocyte transendothelial migration | 0.697138615 | 1.039236046 | 1 | 1 | 46 |
| hsa04010 MAPK signaling pathway | 0.707186444 | 1.04808766 | 1 | 1 | 98 |
| hsa00982 Drug metabolism - cytochrome P450 | 0.610342455 | 1.087563285 | 1 | 1 | 23 |
| hsa04020 Calcium signaling pathway | 0.695878714 | 1.071985624 | 1 | 1 | 64 |
| hsa00190 Oxidative phosphorylation | 0.737152037 | 1.089125289 | 1 | 1 | 44 |
| hsa04920 Adipocytokine signaling pathway | 0.796193616 | 1.089798802 | 1 | 1 | 33 |
| hsa00480 Glutathione metabolism | 0.786642536 | 1.110178902 | 1 | 1 | 24 |
| hsa00051 Fructose and mannose metabolism | 0.823922855 | 1.172167665 | 1 | 1 | 17 |
| hsa00531 Glycosaminoglycan degradation | 0.803065014 | 1.257329942 | 1 | 1 | 10 |
| hsa00010 Glycolysis / Gluconeogenesis | 0.849968102 | 1.438619389 | 1 | 1 | 31 |
| hsa00052 Galactose metabolism | 0.829285731 | 1.272071517 | 1 | 1 | 16 |
| hsa00983 Drug metabolism - other enzymes | 0.749096419 | 1.452537004 | 1 | 1 | 23 |
| hsa03320 PPAR signaling pathway | 0.886542926 | 1.597233745 | 1 | 1 | 24 |
| hsa04142 Lysosome | 0.818320689 | 2.027149078 | 1 | 1 | 59 |
| hsa04145 Phagosome | 0.668260621 | 1.302658365 | 1 | 1 | 73 |
| hsa04380 Osteoclast differentiation | 0.733913776 | 1.948024403 | 1 | 1 | 65 |
| hsa04510 Focal adhesion | 0.74311781 | 1.478517677 | 1 | 1 | 76 |
| hsa04512 ECM-receptor interaction | 0.69877176 | 1.290258349 | 1 | 1 | 33 |
| hsa04610 Complement and coagulation cascades | 0.899216217 | 2.627115289 | 1 | 1 | 27 |
| hsa04620 Toll-like receptor signaling pathway | 0.75078068 | 1.404108552 | 1 | 1 | 35 |
| hsa04621 NOD-like receptor signaling pathway | 0.790571784 | 1.507315834 | 1 | 1 | 25 |
| hsa04666 Fc gamma R-mediated phagocytosis | 0.781807072 | 1.692698376 | 1 | 1 | 45 |
| hsa04740 Olfactory transduction | 0.577770189 | 1.286221584 | 1 | 1 | 29 |
| hsa04810 Regulation of actin cytoskeleton | 0.811449739 | 1.886943364 | 1 | 1 | 77 |
| hsa04910 Insulin signaling pathway | 0.783235592 | 1.37837677 | 1 | 1 | 61 |
| hsa00061 Fatty acid biosynthesis | NA | NA | NA | NA | 2 |
| hsa00072 Synthesis and degradation of ketone bodies | NA | NA | NA | NA | 3 |
| hsa00100 Steroid biosynthesis | NA | NA | NA | NA | 6 |
| hsa00120 Primary bile acid biosynthesis | NA | NA | NA | NA | 3 |
| hsa00130 Ubiquinone and other terpenoid-quinone biosynthesis | NA | NA | NA | NA | 4 |
| hsa00232 Caffeine metabolism | NA | NA | NA | NA | 1 |
| hsa00250 Alanine, aspartate and glutamate metabolism | NA | NA | NA | NA | 9 |
| hsa00260 Glycine, serine and threonine metabolism | NA | NA | NA | NA | 7 |
| hsa00290 Valine, leucine and isoleucine biosynthesis | NA | NA | NA | NA | 3 |
| hsa00300 Lysine biosynthesis | NA | NA | NA | NA | 0 |
| hsa00360 Phenylalanine metabolism | NA | NA | NA | NA | 5 |
| hsa00400 Phenylalanine, tyrosine and tryptophan biosynthesis | NA | NA | NA | NA | 1 |
| hsa00410 beta-Alanine metabolism | NA | NA | NA | NA | 9 |
| hsa00430 Taurine and hypotaurine metabolism | NA | NA | NA | NA | 4 |
| hsa00450 Selenocompound metabolism | NA | NA | NA | NA | 9 |
| hsa00460 Cyanoamino acid metabolism | NA | NA | NA | NA | 2 |
| hsa00471 D-Glutamine and D-glutamate metabolism | NA | NA | NA | NA | 1 |
| hsa00472 D-Arginine and D-ornithine metabolism | NA | NA | NA | NA | 0 |
| hsa00511 Other glycan degradation | NA | NA | NA | NA | 4 |
| hsa00533 Glycosaminoglycan biosynthesis - keratan sulfate | NA | NA | NA | NA | 6 |
| hsa00591 Linoleic acid metabolism | NA | NA | NA | NA | 6 |
| hsa00592 alpha-Linolenic acid metabolism | NA | NA | NA | NA | 5 |
| hsa00601 Glycosphingolipid biosynthesis - lacto and neolacto series | NA | NA | NA | NA | 9 |
| hsa00603 Glycosphingolipid biosynthesis - globo series | NA | NA | NA | NA | 6 |
| hsa00604 Glycosphingolipid biosynthesis - ganglio series | NA | NA | NA | NA | 9 |
| hsa00630 Glyoxylate and dicarboxylate metabolism | NA | NA | NA | NA | 6 |
| hsa00650 Butanoate metabolism | NA | NA | NA | NA | 9 |
| hsa00670 One carbon pool by folate | NA | NA | NA | NA | 8 |
| hsa00730 Thiamine metabolism | NA | NA | NA | NA | 0 |
| hsa00740 Riboflavin metabolism | NA | NA | NA | NA | 5 |
| hsa00750 Vitamin B6 metabolism | NA | NA | NA | NA | 4 |
| hsa00770 Pantothenate and CoA biosynthesis | NA | NA | NA | NA | 6 |
| hsa00780 Biotin metabolism | NA | NA | NA | NA | 0 |
| hsa00785 Lipoic acid metabolism | NA | NA | NA | NA | 2 |
| hsa00790 Folate biosynthesis | NA | NA | NA | NA | 5 |
| hsa00900 Terpenoid backbone biosynthesis | NA | NA | NA | NA | 4 |
| hsa00910 Nitrogen metabolism | NA | NA | NA | NA | 7 |
| hsa00920 Sulfur metabolism | NA | NA | NA | NA | 5 |
| hsa01040 Biosynthesis of unsaturated fatty acids | NA | NA | NA | NA | 8 |
| hsa03020 RNA polymerase | NA | NA | NA | NA | 9 |
| hsa03450 Non-homologous end-joining | NA | NA | NA | NA | 5 |
| hsa04122 Sulfur relay system | NA | NA | NA | NA | 3 |
| hsa04140 Regulation of autophagy | NA | NA | NA | NA | 9 |
| hsa04340 Hedgehog signaling pathway | NA | NA | NA | NA | 9 |
| hsa04614 Renin-angiotensin system | NA | NA | NA | NA | 5 |
| hsa04710 Circadian rhythm - mammal | NA | NA | NA | NA | 7 |
| hsa04744 Phototransduction | NA | NA | NA | NA | 9 |
| hsa04964 Proximal tubule bicarbonate reclamation | NA | NA | NA | NA | 6 |
| hsa04977 Vitamin digestion and absorption | NA | NA | NA | NA | 8 |
|  |  |  |  |  |  |
| **GSE72829** | **p.geomean** | **stat.mean** | **p.val** | **q.val** | **set.**  **size** |
| hsa04670 Leukocyte transendothelial migration | 0.05415705 | 1.300194212 | 2.01E-20 | 2.73E-18 | 51 |
| hsa04810 Regulation of actin cytoskeleton | 0.048998803 | 1.27237948 | 5.85E-20 | 3.98E-18 | 91 |
| hsa04142 Lysosome | 0.084005127 | 1.204835267 | 3.40E-18 | 1.54E-16 | 74 |
| hsa04666 Fc gamma R-mediated phagocytosis | 0.061311067 | 1.166895344 | 6.26E-17 | 2.13E-15 | 51 |
| hsa04380 Osteoclast differentiation | 0.061344043 | 1.083941709 | 5.54E-15 | 1.51E-13 | 76 |
| hsa00030 Pentose phosphate pathway | 0.128313338 | 0.990613986 | 2.73E-12 | 6.18E-11 | 16 |
| hsa04664 Fc epsilon RI signaling pathway | 0.135860182 | 0.913638652 | 4.95E-11 | 9.62E-10 | 32 |
| hsa04145 Phagosome | 0.133686986 | 0.758400951 | 2.76E-08 | 4.69E-07 | 82 |
| hsa00010 Glycolysis / Gluconeogenesis | 0.20366167 | 0.739123308 | 6.59E-08 | 9.96E-07 | 35 |
| hsa00500 Starch and sucrose metabolism | 0.230093425 | 0.705126812 | 2.63E-07 | 3.58E-06 | 20 |
| hsa04966 Collecting duct acid secretion | 0.213661309 | 0.704455122 | 3.65E-07 | 4.51E-06 | 13 |
| hsa04650 Natural killer cell mediated cytotoxicity | 0.168056547 | 0.681314767 | 5.55E-07 | 6.29E-06 | 65 |
| hsa04976 Bile secretion | 0.228454012 | 0.676990106 | 7.91E-07 | 8.27E-06 | 19 |
| hsa00190 Oxidative phosphorylation | 0.154362526 | 0.668569919 | 9.97E-07 | 9.69E-06 | 57 |
| hsa00590 Arachidonic acid metabolism | 0.24434838 | 0.654543094 | 1.53E-06 | 1.39E-05 | 22 |
| hsa04662 B cell receptor signaling pathway | 0.154654558 | 0.651501074 | 1.75E-06 | 1.49E-05 | 48 |
| hsa04510 Focal adhesion | 0.188401339 | 0.641011783 | 2.16E-06 | 1.73E-05 | 77 |
| hsa04722 Neurotrophin signaling pathway | 0.21096455 | 0.636079059 | 2.56E-06 | 1.94E-05 | 62 |
| hsa04520 Adherens junction | 0.182161148 | 0.639337348 | 2.75E-06 | 1.97E-05 | 34 |
| hsa00531 Glycosaminoglycan degradation | 0.255402943 | 0.633352137 | 3.55E-06 | 2.42E-05 | 15 |
| hsa04910 Insulin signaling pathway | 0.253927411 | 0.602859056 | 7.45E-06 | 4.83E-05 | 64 |
| hsa00860 Porphyrin and chlorophyll metabolism | 0.232700362 | 0.62124119 | 7.93E-06 | 4.90E-05 | 12 |
| hsa00520 Amino sugar and nucleotide sugar metabolism | 0.269205274 | 0.598621439 | 9.98E-06 | 5.90E-05 | 24 |
| hsa04620 Toll-like receptor signaling pathway | 0.245260934 | 0.552207624 | 3.88E-05 | 0.000219832 | 46 |
| hsa00480 Glutathione metabolism | 0.273746242 | 0.547138149 | 5.02E-05 | 0.000273148 | 19 |
| hsa04210 Apoptosis | 0.253284213 | 0.523650063 | 8.82E-05 | 0.000461596 | 50 |
| hsa04971 Gastric acid secretion | 0.264769995 | 0.518522828 | 0.000109677 | 0.000552449 | 27 |
| hsa04920 Adipocytokine signaling pathway | 0.252463312 | 0.510432064 | 0.000131522 | 0.00063882 | 37 |
| hsa04270 Vascular smooth muscle contraction | 0.286367247 | 0.505650324 | 0.000148116 | 0.000694611 | 37 |
| hsa04062 Chemokine signaling pathway | 0.180036176 | 0.497523935 | 0.000179582 | 0.000814105 | 93 |
| hsa00770 Pantothenate and CoA biosynthesis | 0.29851147 | 0.489354291 | 0.000280391 | 0.001230102 | 11 |
| hsa00051 Fructose and mannose metabolism | 0.311024424 | 0.465713336 | 0.000453299 | 0.001926519 | 20 |
| hsa04530 Tight junction | 0.237591825 | 0.433256405 | 0.000986309 | 0.004064789 | 49 |
| hsa04730 Long-term depression | 0.285485043 | 0.427601115 | 0.001197421 | 0.004789683 | 19 |
| hsa00052 Galactose metabolism | 0.346320092 | 0.383642286 | 0.003168516 | 0.012311946 | 17 |
| hsa04962 Vasopressin-regulated water reabsorption | 0.318861612 | 0.374360219 | 0.003867763 | 0.01461155 | 20 |
| hsa03320 PPAR signaling pathway | 0.344239701 | 0.370554856 | 0.0040354 | 0.014832821 | 29 |
| hsa04622 RIG-I-like receptor signaling pathway | 0.334721092 | 0.367593145 | 0.004338377 | 0.015526822 | 24 |
| hsa00564 Glycerophospholipid metabolism | 0.353347659 | 0.349951851 | 0.006080495 | 0.021203777 | 40 |
| hsa00980 Metabolism of xenobiotics by cytochrome P450 | 0.347086045 | 0.344640139 | 0.007433866 | 0.025275145 | 12 |
| hsa04146 Peroxisome | 0.357941218 | 0.336681878 | 0.007852141 | 0.026046126 | 43 |
| hsa04010 MAPK signaling pathway | 0.303773303 | 0.326024378 | 0.009486739 | 0.030718964 | 116 |
| hsa04540 Gap junction | 0.35262357 | 0.306802879 | 0.014069983 | 0.044500412 | 33 |
| hsa00983 Drug metabolism - other enzymes | 0.369557312 | 0.307722038 | 0.014480895 | 0.044759131 | 16 |
| hsa00330 Arginine and proline metabolism | 0.369542922 | 0.296236364 | 0.017017578 | 0.051430903 | 25 |
| hsa04610 Complement and coagulation cascades | 0.332372122 | 0.293370135 | 0.018308377 | 0.054085628 | 25 |
| hsa00600 Sphingolipid metabolism | 0.382277084 | 0.291911068 | 0.018691357 | 0.054085628 | 23 |
| hsa04144 Endocytosis | 0.338752752 | 0.28779002 | 0.019277297 | 0.054619009 | 83 |
| hsa04260 Cardiac muscle contraction | 0.313122423 | 0.286526928 | 0.021167507 | 0.058750631 | 24 |
| hsa04115 p53 signaling pathway | 0.37829683 | 0.264931243 | 0.028799736 | 0.078335282 | 35 |
| hsa00760 Nicotinate and nicotinamide metabolism | 0.380364897 | 0.252157541 | 0.037371028 | 0.099656075 | 11 |
| hsa00512 Mucin type O-Glycan biosynthesis | 0.398577935 | 0.246671028 | 0.040446026 | 0.105781914 | 11 |
| hsa00982 Drug metabolism - cytochrome P450 | 0.400001444 | 0.206047237 | 0.073351914 | 0.188223778 | 10 |
| hsa04370 VEGF signaling pathway | 0.386378166 | 0.192329988 | 0.083921656 | 0.20857907 | 35 |
| hsa04973 Carbohydrate digestion and absorption | 0.418162638 | 0.193062855 | 0.08435183 | 0.20857907 | 18 |
| hsa00511 Other glycan degradation | 0.419016152 | 0.182453464 | 0.099354195 | 0.241288759 | 11 |
| hsa04975 Fat digestion and absorption | 0.438299 | 0.144159942 | 0.1534701 | 0.366174274 | 14 |
| hsa04912 GnRH signaling pathway | 0.451066488 | 0.105164164 | 0.225725679 | 0.5292878 | 34 |
| hsa04360 Axon guidance | 0.356112742 | 0.093695189 | 0.249742443 | 0.575677495 | 58 |
| hsa04630 Jak-STAT signaling pathway | 0.369759818 | 0.077546745 | 0.287398899 | 0.638999628 | 64 |
| hsa04972 Pancreatic secretion | 0.460986032 | 0.078237041 | 0.28776535 | 0.638999628 | 29 |
| hsa04621 NOD-like receptor signaling pathway | 0.417932398 | 0.076897348 | 0.291308654 | 0.638999628 | 33 |
| hsa00071 Fatty acid metabolism | 0.462888562 | 0.068292168 | 0.312546346 | 0.674703223 | 22 |
| hsa02010 ABC transporters | 0.472205352 | 0.053002746 | 0.353234104 | 0.750622471 | 13 |
| hsa00562 Inositol phosphate metabolism | 0.464587509 | 0.044131808 | 0.376372083 | 0.773007771 | 23 |
| hsa04130 SNARE interactions in vesicular transport | 0.46712152 | 0.044084385 | 0.376543896 | 0.773007771 | 22 |
| hsa04974 Protein digestion and absorption | 0.476241193 | 0.042573966 | 0.380820005 | 0.773007771 | 21 |
| hsa04150 mTOR signaling pathway | 0.461384546 | 0.02970994 | 0.415831959 | 0.831663918 | 25 |
| hsa00532 Glycosaminoglycan biosynthesis - chondroitin sulfate | 0.475815539 | 0.022498477 | 0.436063778 | 0.859488026 | 14 |
| hsa04114 Oocyte meiosis | 0.452100421 | 0.014563241 | 0.458823796 | 0.891429089 | 53 |
| hsa04960 Aldosterone-regulated sodium reabsorption | 0.4899527 | 0.006472858 | 0.481575601 | 0.915739302 | 13 |
| hsa00640 Propanoate metabolism | 0.470580566 | 0.001655696 | 0.495325236 | 0.915739302 | 18 |
| hsa01040 Biosynthesis of unsaturated fatty acids | 0.49627936 | 0.000527438 | 0.498599457 | 0.915739302 | 13 |
| hsa00380 Tryptophan metabolism | 0.489786822 | -0.000565913 | 0.501391975 | 0.915739302 | 17 |
| hsa00450 Selenocompound metabolism | 0.497639791 | -0.001773829 | 0.505003291 | 0.915739302 | 12 |
| hsa00601 Glycosphingolipid biosynthesis - lacto and neolacto series | 0.501935428 | -0.010456592 | 0.529230933 | 0.938909975 | 10 |
| hsa04623 Cytosolic DNA-sensing pathway | 0.446327511 | -0.011807675 | 0.53436575 | 0.938909975 | 22 |
| hsa04310 Wnt signaling pathway | 0.431892011 | -0.013720991 | 0.538492486 | 0.938909975 | 70 |
| hsa04320 Dorso-ventral axis formation | 0.498647009 | -0.023521742 | 0.566589646 | 0.965592759 | 11 |
| hsa00565 Ether lipid metabolism | 0.504964205 | -0.026166305 | 0.573383612 | 0.965592759 | 15 |
| hsa04914 Progesterone-mediated oocyte maturation | 0.498039317 | -0.028208371 | 0.580086656 | 0.965592759 | 42 |
| hsa04640 Hematopoietic cell lineage | 0.457126988 | -0.029081356 | 0.582195634 | 0.965592759 | 56 |
| hsa00604 Glycosphingolipid biosynthesis - ganglio series | 0.515383567 | -0.055600076 | 0.651622018 | 1 | 11 |
| hsa04110 Cell cycle | 0.469470205 | -0.067187099 | 0.68558538 | 1 | 70 |
| hsa00310 Lysine degradation | 0.518944513 | -0.071687324 | 0.695507903 | 1 | 23 |
| hsa00240 Pyrimidine metabolism | 0.511212106 | -0.077453642 | 0.710719124 | 1 | 50 |
| hsa04710 Circadian rhythm - mammal | 0.517044399 | -0.082909105 | 0.719708411 | 1 | 13 |
| hsa04512 ECM-receptor interaction | 0.520742941 | -0.081874779 | 0.720578404 | 1 | 27 |
| hsa00260 Glycine, serine and threonine metabolism | 0.521665791 | -0.08724246 | 0.73171996 | 1 | 16 |
| hsa00561 Glycerolipid metabolism | 0.529267397 | -0.091483179 | 0.743411647 | 1 | 27 |
| hsa00830 Retinol metabolism | 0.531488923 | -0.095501208 | 0.74959857 | 1 | 13 |
| hsa04012 ErbB signaling pathway | 0.531334814 | -0.108659929 | 0.781558867 | 1 | 36 |
| hsa04916 Melanogenesis | 0.536788558 | -0.116275365 | 0.79776179 | 1 | 43 |
| hsa04720 Long-term potentiation | 0.539812588 | -0.122464462 | 0.808898973 | 1 | 25 |
| hsa03022 Basal transcription factors | 0.546697669 | -0.133308704 | 0.826859015 | 1 | 14 |
| hsa04740 Olfactory transduction | 0.546439763 | -0.134106761 | 0.831112177 | 1 | 31 |
| hsa00534 Glycosaminoglycan biosynthesis - heparan sulfate | 0.553232201 | -0.143383745 | 0.844185555 | 1 | 13 |
| hsa03050 Proteasome | 0.375344809 | -0.141609659 | 0.850481622 | 1 | 23 |
| hsa00270 Cysteine and methionine metabolism | 0.513154296 | -0.151542624 | 0.859221979 | 1 | 18 |
| hsa04070 Phosphatidylinositol signaling system | 0.541313547 | -0.1573775 | 0.869816489 | 1 | 32 |
| hsa04742 Taste transduction | 0.566138028 | -0.179815313 | 0.897661298 | 1 | 13 |
| hsa00563 Glycosylphosphatidylinositol(GPI)-anchor biosynthesis | 0.565878474 | -0.181485207 | 0.898275033 | 1 | 10 |
| hsa00350 Tyrosine metabolism | 0.556096966 | -0.188749709 | 0.90935875 | 1 | 17 |
| hsa03060 Protein export | 0.516360908 | -0.198999719 | 0.920339465 | 1 | 14 |
| hsa00650 Butanoate metabolism | 0.566745718 | -0.207254297 | 0.927911669 | 1 | 13 |
| hsa04141 Protein processing in endoplasmic reticulum | 0.531407778 | -0.206011985 | 0.930676734 | 1 | 75 |
| hsa04020 Calcium signaling pathway | 0.570299867 | -0.215597936 | 0.939172555 | 1 | 58 |
| hsa04970 Salivary secretion | 0.526780031 | -0.225270502 | 0.946310651 | 1 | 23 |
| hsa04340 Hedgehog signaling pathway | 0.583940654 | -0.228833175 | 0.947791429 | 1 | 17 |
| hsa00280 Valine, leucine and isoleucine degradation | 0.579311776 | -0.235482498 | 0.953612291 | 1 | 28 |
| hsa00340 Histidine metabolism | 0.582973922 | -0.243246524 | 0.95705023 | 1 | 13 |
| hsa03020 RNA polymerase | 0.580728054 | -0.248585184 | 0.959928142 | 1 | 13 |
| hsa03420 Nucleotide excision repair | 0.554329955 | -0.252428913 | 0.96352221 | 1 | 24 |
| hsa00620 Pyruvate metabolism | 0.544684903 | -0.263946904 | 0.970046305 | 1 | 22 |
| hsa04120 Ubiquitin mediated proteolysis | 0.552684596 | -0.272689134 | 0.974702814 | 1 | 62 |
| hsa03410 Base excision repair | 0.575019955 | -0.275968942 | 0.974751055 | 1 | 19 |
| hsa00020 Citrate cycle (TCA cycle) | 0.563113544 | -0.283547069 | 0.977726892 | 1 | 16 |
| hsa04330 Notch signaling pathway | 0.598547966 | -0.285039521 | 0.978917046 | 1 | 23 |
| hsa03440 Homologous recombination | 0.609275818 | -0.308974306 | 0.984705069 | 1 | 10 |
| hsa00510 N-Glycan biosynthesis | 0.604102455 | -0.304665233 | 0.984745201 | 1 | 20 |
| hsa03430 Mismatch repair | 0.610380943 | -0.314208095 | 0.986416668 | 1 | 12 |
| hsa03030 DNA replication | 0.595469255 | -0.343968193 | 0.992555544 | 1 | 19 |
| hsa03008 Ribosome biogenesis in eukaryotes | 0.593444661 | -0.388030206 | 0.99722464 | 1 | 38 |
| hsa04350 TGF-beta signaling pathway | 0.619756171 | -0.392880015 | 0.997558426 | 1 | 41 |
| hsa00970 Aminoacyl-tRNA biosynthesis | 0.622543257 | -0.422848601 | 0.998675768 | 1 | 24 |
| hsa00230 Purine metabolism | 0.632716287 | -0.450409799 | 0.999391342 | 1 | 74 |
| hsa00514 Other types of O-glycan biosynthesis | 0.665157296 | -0.472557863 | 0.99958388 | 1 | 15 |
| hsa03015 mRNA surveillance pathway | 0.675444585 | -0.545651016 | 0.999951037 | 1 | 33 |
| hsa04660 T cell receptor signaling pathway | 0.66128095 | -0.605689364 | 0.999993102 | 1 | 68 |
| hsa03018 RNA degradation | 0.67967229 | -0.646287141 | 0.999998053 | 1 | 35 |
| hsa04514 Cell adhesion molecules (CAMs) | 0.639242597 | -0.707047442 | 0.999999778 | 1 | 57 |
| hsa03013 RNA transport | 0.679393562 | -1.044284155 | 1 | 1 | 69 |
| hsa03040 Spliceosome | 0.748589683 | -1.058781283 | 1 | 1 | 76 |
| hsa03010 Ribosome | 0.320420663 | -4.234576248 | 1 | 1 | 50 |
| hsa04612 Antigen processing and presentation | 0.743070708 | -1.247614409 | 1 | 1 | 48 |
| hsa04672 Intestinal immune network for IgA production | 0.83971879 | -1.555921998 | 1 | 1 | 29 |
| hsa00040 Pentose and glucuronate interconversions | NA | NA | NA | NA | 8 |
| hsa00053 Ascorbate and aldarate metabolism | NA | NA | NA | NA | 3 |
| hsa00061 Fatty acid biosynthesis | NA | NA | NA | NA | 4 |
| hsa00072 Synthesis and degradation of ketone bodies | NA | NA | NA | NA | 4 |
| hsa00100 Steroid biosynthesis | NA | NA | NA | NA | 6 |
| hsa00120 Primary bile acid biosynthesis | NA | NA | NA | NA | 5 |
| hsa00130 Ubiquinone and other terpenoid-quinone biosynthesis | NA | NA | NA | NA | 4 |
| hsa00140 Steroid hormone biosynthesis | NA | NA | NA | NA | 7 |
| hsa00232 Caffeine metabolism | NA | NA | NA | NA | 1 |
| hsa00250 Alanine, aspartate and glutamate metabolism | NA | NA | NA | NA | 8 |
| hsa00290 Valine, leucine and isoleucine biosynthesis | NA | NA | NA | NA | 8 |
| hsa00300 Lysine biosynthesis | NA | NA | NA | NA | 0 |
| hsa00360 Phenylalanine metabolism | NA | NA | NA | NA | 6 |
| hsa00400 Phenylalanine, tyrosine and tryptophan biosynthesis | NA | NA | NA | NA | 2 |
| hsa00410 beta-Alanine metabolism | NA | NA | NA | NA | 9 |
| hsa00430 Taurine and hypotaurine metabolism | NA | NA | NA | NA | 3 |
| hsa00460 Cyanoamino acid metabolism | NA | NA | NA | NA | 3 |
| hsa00471 D-Glutamine and D-glutamate metabolism | NA | NA | NA | NA | 1 |
| hsa00472 D-Arginine and D-ornithine metabolism | NA | NA | NA | NA | 1 |
| hsa00533 Glycosaminoglycan biosynthesis - keratan sulfate | NA | NA | NA | NA | 6 |
| hsa00591 Linoleic acid metabolism | NA | NA | NA | NA | 5 |
| hsa00592 alpha-Linolenic acid metabolism | NA | NA | NA | NA | 3 |
| hsa00603 Glycosphingolipid biosynthesis - globo series | NA | NA | NA | NA | 9 |
| hsa00630 Glyoxylate and dicarboxylate metabolism | NA | NA | NA | NA | 6 |
| hsa00670 One carbon pool by folate | NA | NA | NA | NA | 8 |
| hsa00730 Thiamine metabolism | NA | NA | NA | NA | 2 |
| hsa00740 Riboflavin metabolism | NA | NA | NA | NA | 5 |
| hsa00750 Vitamin B6 metabolism | NA | NA | NA | NA | 4 |
| hsa00780 Biotin metabolism | NA | NA | NA | NA | 1 |
| hsa00785 Lipoic acid metabolism | NA | NA | NA | NA | 2 |
| hsa00790 Folate biosynthesis | NA | NA | NA | NA | 4 |
| hsa00900 Terpenoid backbone biosynthesis | NA | NA | NA | NA | 6 |
| hsa00910 Nitrogen metabolism | NA | NA | NA | NA | 7 |
| hsa00920 Sulfur metabolism | NA | NA | NA | NA | 8 |
| hsa03450 Non-homologous end-joining | NA | NA | NA | NA | 5 |
| hsa04122 Sulfur relay system | NA | NA | NA | NA | 5 |
| hsa04140 Regulation of autophagy | NA | NA | NA | NA | 8 |
| hsa04614 Renin-angiotensin system | NA | NA | NA | NA | 7 |
| hsa04744 Phototransduction | NA | NA | NA | NA | 7 |
| hsa04964 Proximal tubule bicarbonate reclamation | NA | NA | NA | NA | 4 |
| hsa04977 Vitamin digestion and absorption | NA | NA | NA | NA | 6 |
| hsa03010 Ribosome | 9.17E-07 | -4.234576248 | 5.32E-154 | 7.24E-152 | 50 |
| hsa04672 Intestinal immune network for IgA production | 0.04881873 | -1.555921998 | 3.72E-28 | 2.53E-26 | 29 |
| hsa04612 Antigen processing and presentation | 0.073141503 | -1.247614409 | 4.33E-19 | 1.96E-17 | 48 |
| hsa03040 Spliceosome | 0.118116448 | -1.058781283 | 1.87E-14 | 6.36E-13 | 76 |
| hsa03013 RNA transport | 0.104722919 | -1.044284155 | 4.33E-14 | 1.18E-12 | 69 |
| hsa04514 Cell adhesion molecules (CAMs) | 0.181617266 | -0.707047442 | 2.22E-07 | 5.03E-06 | 57 |
| hsa03018 RNA degradation | 0.232682495 | -0.646287141 | 1.95E-06 | 3.78E-05 | 35 |
| hsa04660 T cell receptor signaling pathway | 0.239198359 | -0.605689364 | 6.90E-06 | 0.000117272 | 68 |
| hsa03015 mRNA surveillance pathway | 0.278293382 | -0.545651016 | 4.90E-05 | 0.000739888 | 33 |
| hsa00514 Other types of O-glycan biosynthesis | 0.313532887 | -0.472557863 | 0.00041612 | 0.005659234 | 15 |
| hsa00230 Purine metabolism | 0.303521584 | -0.450409799 | 0.000608658 | 0.007525229 | 74 |
| hsa00970 Aminoacyl-tRNA biosynthesis | 0.315071711 | -0.422848601 | 0.001324232 | 0.015007958 | 24 |
| hsa04350 TGF-beta signaling pathway | 0.327643526 | -0.392880015 | 0.002441574 | 0.025542624 | 41 |
| hsa03008 Ribosome biogenesis in eukaryotes | 0.315995839 | -0.388030206 | 0.00277536 | 0.026960636 | 38 |
| hsa03030 DNA replication | 0.343720855 | -0.343968193 | 0.007444456 | 0.067496399 | 19 |
| hsa03430 Mismatch repair | 0.372674369 | -0.314208095 | 0.013583332 | 0.115458325 | 12 |
| hsa00510 N-Glycan biosynthesis | 0.372324425 | -0.304665233 | 0.015254799 | 0.115561699 | 20 |
| hsa03440 Homologous recombination | 0.376001956 | -0.308974306 | 0.015294931 | 0.115561699 | 10 |
| hsa04330 Notch signaling pathway | 0.379533174 | -0.285039521 | 0.021082954 | 0.150909564 | 23 |
| hsa00020 Citrate cycle (TCA cycle) | 0.358399472 | -0.283547069 | 0.022273108 | 0.151457134 | 16 |
| hsa03410 Base excision repair | 0.370392003 | -0.275968942 | 0.025248945 | 0.156382607 | 19 |
| hsa04120 Ubiquitin mediated proteolysis | 0.355060165 | -0.272689134 | 0.025297186 | 0.156382607 | 62 |
| hsa00620 Pyruvate metabolism | 0.35635919 | -0.263946904 | 0.029953695 | 0.177117501 | 22 |
| hsa03420 Nucleotide excision repair | 0.36979153 | -0.252428913 | 0.03647779 | 0.206707474 | 24 |
| hsa03020 RNA polymerase | 0.392774662 | -0.248585184 | 0.040071858 | 0.217990907 | 13 |
| hsa00340 Histidine metabolism | 0.397288327 | -0.243246524 | 0.04294977 | 0.224660335 | 13 |
| hsa00280 Valine, leucine and isoleucine degradation | 0.398078308 | -0.235482498 | 0.046387709 | 0.233656608 | 28 |
| hsa04340 Hedgehog signaling pathway | 0.406776113 | -0.228833175 | 0.052208571 | 0.251784533 | 17 |
| hsa04970 Salivary secretion | 0.366581271 | -0.225270502 | 0.053689349 | 0.251784533 | 23 |
| hsa04020 Calcium signaling pathway | 0.403703432 | -0.215597936 | 0.060827445 | 0.275751086 | 58 |
| hsa04141 Protein processing in endoplasmic reticulum | 0.381702908 | -0.206011985 | 0.069323266 | 0.304127877 | 75 |
| hsa00650 Butanoate metabolism | 0.409377434 | -0.207254297 | 0.072088331 | 0.306375407 | 13 |
| hsa03060 Protein export | 0.377675947 | -0.198999719 | 0.079660535 | 0.328297964 | 14 |
| hsa00350 Tyrosine metabolism | 0.412770751 | -0.188749709 | 0.09064125 | 0.362565001 | 17 |
| hsa00563 Glycosylphosphatidylinositol(GPI)-anchor biosynthesis | 0.426613556 | -0.181485207 | 0.101724967 | 0.386612873 | 10 |
| hsa04742 Taste transduction | 0.427173797 | -0.179815313 | 0.102338702 | 0.386612873 | 13 |
| hsa04070 Phosphatidylinositol signaling system | 0.420503382 | -0.1573775 | 0.130183511 | 0.478512363 | 32 |
| hsa00270 Cysteine and methionine metabolism | 0.403625067 | -0.151542624 | 0.140778021 | 0.503837127 | 18 |
| hsa03050 Proteasome | 0.30919273 | -0.141609659 | 0.149518378 | 0.521397421 | 23 |
| hsa00534 Glycosaminoglycan biosynthesis - heparan sulfate | 0.44199671 | -0.143383745 | 0.155814445 | 0.529769115 | 13 |
| hsa04740 Olfactory transduction | 0.441576533 | -0.134106761 | 0.168887823 | 0.560213266 | 31 |
| hsa03022 Basal transcription factors | 0.443549634 | -0.133308704 | 0.173140985 | 0.560646998 | 14 |
| hsa04720 Long-term potentiation | 0.444469694 | -0.122464462 | 0.191101027 | 0.604412552 | 25 |
| hsa04916 Melanogenesis | 0.445697524 | -0.116275365 | 0.20223821 | 0.625099923 | 43 |
| hsa04012 ErbB signaling pathway | 0.446963275 | -0.108659929 | 0.218441133 | 0.660177645 | 36 |
| hsa00830 Retinol metabolism | 0.457717745 | -0.095501208 | 0.25040143 | 0.740317271 | 13 |
| hsa00561 Glycerolipid metabolism | 0.4576984 | -0.091483179 | 0.256588353 | 0.742468426 | 27 |
| hsa00260 Glycine, serine and threonine metabolism | 0.455112992 | -0.08724246 | 0.26828004 | 0.760126779 | 16 |
| hsa04512 ECM-receptor interaction | 0.457427865 | -0.081874779 | 0.279421596 | 0.762393123 | 27 |
| hsa04710 Circadian rhythm - mammal | 0.45427317 | -0.082909105 | 0.280291589 | 0.762393123 | 13 |
| hsa00240 Pyrimidine metabolism | 0.451698641 | -0.077453642 | 0.289280876 | 0.77141567 | 50 |
| hsa00310 Lysine degradation | 0.463147847 | -0.071687324 | 0.304492097 | 0.796363946 | 23 |
| hsa04110 Cell cycle | 0.424928972 | -0.067187099 | 0.31441462 | 0.80679978 | 70 |
| hsa00604 Glycosphingolipid biosynthesis - ganglio series | 0.472727794 | -0.055600076 | 0.348377982 | 0.8773964 | 11 |
| hsa04640 Hematopoietic cell lineage | 0.432301457 | -0.029081356 | 0.417804366 | 1 | 56 |
| hsa04914 Progesterone-mediated oocyte maturation | 0.476273347 | -0.028208371 | 0.419913344 | 1 | 42 |
| hsa00565 Ether lipid metabolism | 0.484641547 | -0.026166305 | 0.426616388 | 1 | 15 |
| hsa04320 Dorso-ventral axis formation | 0.480447046 | -0.023521742 | 0.433410354 | 1 | 11 |
| hsa04310 Wnt signaling pathway | 0.419394903 | -0.013720991 | 0.461507514 | 1 | 70 |
| hsa04623 Cytosolic DNA-sensing pathway | 0.438963293 | -0.011807675 | 0.46563425 | 1 | 22 |
| hsa00601 Glycosphingolipid biosynthesis - lacto and neolacto series | 0.493861413 | -0.010456592 | 0.470769067 | 1 | 10 |
| hsa00450 Selenocompound metabolism | 0.49627572 | -0.001773829 | 0.494996709 | 1 | 12 |
| hsa00380 Tryptophan metabolism | 0.489696095 | -0.000565913 | 0.498608025 | 1 | 17 |
| hsa01040 Biosynthesis of unsaturated fatty acids | 0.496729691 | 0.000527438 | 0.501400543 | 1 | 13 |
| hsa00640 Propanoate metabolism | 0.473172547 | 0.001655696 | 0.504674764 | 1 | 18 |
| hsa04960 Aldosterone-regulated sodium reabsorption | 0.495021092 | 0.006472858 | 0.518424399 | 1 | 13 |
| hsa04114 Oocyte meiosis | 0.466606892 | 0.014563241 | 0.541176204 | 1 | 53 |
| hsa00532 Glycosaminoglycan biosynthesis - chondroitin sulfate | 0.49316074 | 0.022498477 | 0.563936222 | 1 | 14 |
| hsa04150 mTOR signaling pathway | 0.483898981 | 0.02970994 | 0.584168041 | 1 | 25 |
| hsa04974 Protein digestion and absorption | 0.509542926 | 0.042573966 | 0.619179995 | 1 | 21 |
| hsa04130 SNARE interactions in vesicular transport | 0.501382719 | 0.044084385 | 0.623456104 | 1 | 22 |
| hsa00562 Inositol phosphate metabolism | 0.498536373 | 0.044131808 | 0.623627917 | 1 | 23 |
| hsa02010 ABC transporters | 0.513567334 | 0.053002746 | 0.646765896 | 1 | 13 |
| hsa00071 Fatty acid metabolism | 0.516120141 | 0.068292168 | 0.687453654 | 1 | 22 |
| hsa04621 NOD-like receptor signaling pathway | 0.474619639 | 0.076897348 | 0.708691346 | 1 | 33 |
| hsa04972 Pancreatic secretion | 0.522192854 | 0.078237041 | 0.71223465 | 1 | 29 |
| hsa04630 Jak-STAT signaling pathway | 0.41328336 | 0.077546745 | 0.712601101 | 1 | 64 |
| hsa04360 Axon guidance | 0.413139664 | 0.093695189 | 0.750257557 | 1 | 58 |
| hsa04912 GnRH signaling pathway | 0.533390471 | 0.105164164 | 0.774274321 | 1 | 34 |
| hsa04975 Fat digestion and absorption | 0.550249731 | 0.144159942 | 0.8465299 | 1 | 14 |
| hsa00511 Other glycan degradation | 0.55853026 | 0.182453464 | 0.900645805 | 1 | 11 |
| hsa04973 Carbohydrate digestion and absorption | 0.568165781 | 0.193062855 | 0.91564817 | 1 | 18 |
| hsa04370 VEGF signaling pathway | 0.527348675 | 0.192329988 | 0.916078344 | 1 | 35 |
| hsa00982 Drug metabolism - cytochrome P450 | 0.55530716 | 0.206047237 | 0.926648086 | 1 | 10 |
| hsa00512 Mucin type O-Glycan biosynthesis | 0.588221709 | 0.246671028 | 0.959553974 | 1 | 11 |
| hsa00760 Nicotinate and nicotinamide metabolism | 0.567379195 | 0.252157541 | 0.962628972 | 1 | 11 |
| hsa04115 p53 signaling pathway | 0.58016617 | 0.264931243 | 0.971200264 | 1 | 35 |
| hsa04260 Cardiac muscle contraction | 0.508816415 | 0.286526928 | 0.978832493 | 1 | 24 |
| hsa04144 Endocytosis | 0.543355757 | 0.28779002 | 0.980722703 | 1 | 83 |
| hsa00600 Sphingolipid metabolism | 0.608181201 | 0.291911068 | 0.981308643 | 1 | 23 |
| hsa04610 Complement and coagulation cascades | 0.541208764 | 0.293370135 | 0.981691623 | 1 | 25 |
| hsa00330 Arginine and proline metabolism | 0.595473579 | 0.296236364 | 0.982982422 | 1 | 25 |
| hsa00983 Drug metabolism - other enzymes | 0.603809476 | 0.307722038 | 0.985519105 | 1 | 16 |
| hsa04540 Gap junction | 0.579858035 | 0.306802879 | 0.985930017 | 1 | 33 |
| hsa04010 MAPK signaling pathway | 0.520784553 | 0.326024378 | 0.990513261 | 1 | 116 |
| hsa04146 Peroxisome | 0.615680193 | 0.336681878 | 0.992147859 | 1 | 43 |
| hsa00980 Metabolism of xenobiotics by cytochrome P450 | 0.603305618 | 0.344640139 | 0.992566134 | 1 | 12 |
| hsa00564 Glycerophospholipid metabolism | 0.620620284 | 0.349951851 | 0.993919505 | 1 | 40 |
| hsa04622 RIG-I-like receptor signaling pathway | 0.606541439 | 0.367593145 | 0.995661623 | 1 | 24 |
| hsa03320 PPAR signaling pathway | 0.624806784 | 0.370554856 | 0.9959646 | 1 | 29 |
| hsa04962 Vasopressin-regulated water reabsorption | 0.584585048 | 0.374360219 | 0.996132237 | 1 | 20 |
| hsa00052 Galactose metabolism | 0.63845918 | 0.383642286 | 0.996831484 | 1 | 17 |
| hsa04730 Long-term depression | 0.573727796 | 0.427601115 | 0.998802579 | 1 | 19 |
| hsa04530 Tight junction | 0.498026244 | 0.433256405 | 0.999013691 | 1 | 49 |
| hsa00051 Fructose and mannose metabolism | 0.658391143 | 0.465713336 | 0.999546701 | 1 | 20 |
| hsa00770 Pantothenate and CoA biosynthesis | 0.653033847 | 0.489354291 | 0.999719609 | 1 | 11 |
| hsa04062 Chemokine signaling pathway | 0.43477825 | 0.497523935 | 0.999820418 | 1 | 93 |
| hsa04270 Vascular smooth muscle contraction | 0.653822166 | 0.505650324 | 0.999851884 | 1 | 37 |
| hsa04920 Adipocytokine signaling pathway | 0.592032085 | 0.510432064 | 0.999868478 | 1 | 37 |
| hsa04971 Gastric acid secretion | 0.618065663 | 0.518522828 | 0.999890323 | 1 | 27 |
| hsa04210 Apoptosis | 0.610866122 | 0.523650063 | 0.999911754 | 1 | 50 |
| hsa00480 Glutathione metabolism | 0.669833063 | 0.547138149 | 0.999949789 | 1 | 19 |
| hsa04620 Toll-like receptor signaling pathway | 0.617618464 | 0.552207624 | 0.999961206 | 1 | 46 |
| hsa00520 Amino sugar and nucleotide sugar metabolism | 0.708634695 | 0.598621439 | 0.999990023 | 1 | 24 |
| hsa00860 Porphyrin and chlorophyll metabolism | 0.631675707 | 0.62124119 | 0.999992068 | 1 | 12 |
| hsa04910 Insulin signaling pathway | 0.687013218 | 0.602859056 | 0.999992549 | 1 | 64 |
| hsa00531 Glycosaminoglycan degradation | 0.711055963 | 0.633352137 | 0.999996447 | 1 | 15 |
| hsa04520 Adherens junction | 0.537875253 | 0.639337348 | 0.999997254 | 1 | 34 |
| hsa04722 Neurotrophin signaling pathway | 0.620863687 | 0.636079059 | 0.999997438 | 1 | 62 |
| hsa04510 Focal adhesion | 0.570032152 | 0.641011783 | 0.99999784 | 1 | 77 |
| hsa04662 B cell receptor signaling pathway | 0.482772868 | 0.651501074 | 0.999998246 | 1 | 48 |
| hsa00590 Arachidonic acid metabolism | 0.71040965 | 0.654543094 | 0.999998467 | 1 | 22 |
| hsa00190 Oxidative phosphorylation | 0.54379846 | 0.668569919 | 0.999999003 | 1 | 57 |
| hsa04976 Bile secretion | 0.691975954 | 0.676990106 | 0.999999209 | 1 | 19 |
| hsa04650 Natural killer cell mediated cytotoxicity | 0.539077201 | 0.681314767 | 0.999999445 | 1 | 65 |
| hsa04966 Collecting duct acid secretion | 0.677815621 | 0.704455122 | 0.999999635 | 1 | 13 |
| hsa00500 Starch and sucrose metabolism | 0.728003334 | 0.705126812 | 0.999999737 | 1 | 20 |
| hsa00010 Glycolysis / Gluconeogenesis | 0.708824854 | 0.739123308 | 0.999999934 | 1 | 35 |
| hsa04145 Phagosome | 0.494910222 | 0.758400951 | 0.999999972 | 1 | 82 |
| hsa04664 Fc epsilon RI signaling pathway | 0.654002099 | 0.913638652 | 1 | 1 | 32 |
| hsa00030 Pentose phosphate pathway | 0.689858521 | 0.990613986 | 1 | 1 | 16 |
| hsa04380 Osteoclast differentiation | 0.480740605 | 1.083941709 | 1 | 1 | 76 |
| hsa04666 Fc gamma R-mediated phagocytosis | 0.559549688 | 1.166895344 | 1 | 1 | 51 |
| hsa04142 Lysosome | 0.745016658 | 1.204835267 | 1 | 1 | 74 |
| hsa04670 Leukocyte transendothelial migration | 0.612557078 | 1.300194212 | 1 | 1 | 51 |
| hsa04810 Regulation of actin cytoskeleton | 0.560717439 | 1.27237948 | 1 | 1 | 91 |
| hsa00040 Pentose and glucuronate interconversions | NA | NA | NA | NA | 8 |
| hsa00053 Ascorbate and aldarate metabolism | NA | NA | NA | NA | 3 |
| hsa00061 Fatty acid biosynthesis | NA | NA | NA | NA | 4 |
| hsa00072 Synthesis and degradation of ketone bodies | NA | NA | NA | NA | 4 |
| hsa00100 Steroid biosynthesis | NA | NA | NA | NA | 6 |
| hsa00120 Primary bile acid biosynthesis | NA | NA | NA | NA | 5 |
| hsa00130 Ubiquinone and other terpenoid-quinone biosynthesis | NA | NA | NA | NA | 4 |
| hsa00140 Steroid hormone biosynthesis | NA | NA | NA | NA | 7 |
| hsa00232 Caffeine metabolism | NA | NA | NA | NA | 1 |
| hsa00250 Alanine, aspartate and glutamate metabolism | NA | NA | NA | NA | 8 |
| hsa00290 Valine, leucine and isoleucine biosynthesis | NA | NA | NA | NA | 8 |
| hsa00300 Lysine biosynthesis | NA | NA | NA | NA | 0 |
| hsa00360 Phenylalanine metabolism | NA | NA | NA | NA | 6 |
| hsa00400 Phenylalanine, tyrosine and tryptophan biosynthesis | NA | NA | NA | NA | 2 |
| hsa00410 beta-Alanine metabolism | NA | NA | NA | NA | 9 |
| hsa00430 Taurine and hypotaurine metabolism | NA | NA | NA | NA | 3 |
| hsa00460 Cyanoamino acid metabolism | NA | NA | NA | NA | 3 |
| hsa00471 D-Glutamine and D-glutamate metabolism | NA | NA | NA | NA | 1 |
| hsa00472 D-Arginine and D-ornithine metabolism | NA | NA | NA | NA | 1 |
| hsa00533 Glycosaminoglycan biosynthesis - keratan sulfate | NA | NA | NA | NA | 6 |
| hsa00591 Linoleic acid metabolism | NA | NA | NA | NA | 5 |
| hsa00592 alpha-Linolenic acid metabolism | NA | NA | NA | NA | 3 |
| hsa00603 Glycosphingolipid biosynthesis - globo series | NA | NA | NA | NA | 9 |
| hsa00630 Glyoxylate and dicarboxylate metabolism | NA | NA | NA | NA | 6 |
| hsa00670 One carbon pool by folate | NA | NA | NA | NA | 8 |
| hsa00730 Thiamine metabolism | NA | NA | NA | NA | 2 |
| hsa00740 Riboflavin metabolism | NA | NA | NA | NA | 5 |
| hsa00750 Vitamin B6 metabolism | NA | NA | NA | NA | 4 |
| hsa00780 Biotin metabolism | NA | NA | NA | NA | 1 |
| hsa00785 Lipoic acid metabolism | NA | NA | NA | NA | 2 |
| hsa00790 Folate biosynthesis | NA | NA | NA | NA | 4 |
| hsa00900 Terpenoid backbone biosynthesis | NA | NA | NA | NA | 6 |
| hsa00910 Nitrogen metabolism | NA | NA | NA | NA | 7 |
| hsa00920 Sulfur metabolism | NA | NA | NA | NA | 8 |
| hsa03450 Non-homologous end-joining | NA | NA | NA | NA | 5 |
| hsa04122 Sulfur relay system | NA | NA | NA | NA | 5 |
| hsa04140 Regulation of autophagy | NA | NA | NA | NA | 8 |
| hsa04614 Renin-angiotensin system | NA | NA | NA | NA | 7 |
| hsa04744 Phototransduction | NA | NA | NA | NA | 7 |
| hsa04964 Proximal tubule bicarbonate reclamation | NA | NA | NA | NA | 4 |
| hsa04977 Vitamin digestion and absorption | NA | NA | NA | NA | 6 |

**Supplementary Table S5:** Gene included in isolated and prominent network cluster

| GSE54514 | GSE57065 | GSE64456 | GSE66099 | GSE72829 | GSE95233 | GSE95233-1 |  |
| --- | --- | --- | --- | --- | --- | --- | --- |
| Gene | Gene | Gene | Gene | Gene | Gene | Survivor | Nonsurvivor |
| ARHGAP5 | BIN1 | AKT1 | BIRC3 | ARRB1 | ACTB | ADCY1 | BIRC3 |
| ARHGEF12 | CBLB | BCL2 | BLNK | BCL2 | ACTN1 | ADCY2 | CARD11 |
| ARRB1 | CCL5 | BIN1 | CBLB | BIN1 | ACTN4 | ADCY5 | CBLB |
| BCL2 | CD1D | CALM3 | CCR6 | BIRC3 | ARPC1B | ADCY6 | CCL28 |
| BDKRB1 | CD2 | CARD11 | CD19 | CARD11 | ARPC4 | ANTXR1 | CCL4 |
| BIN1 | CD244 | CBLB | CD22 | CARD9 | BAD | ASAP3 | CD2 |
| CALM3 | CD247 | CCL23 | CD247 | CBLB | BCL3 | BDKRB2 | CD247 |
| CARD11 | CD33 | CCR7 | CD3D | CCL5 | CALM1 | C6 | CD28 |
| CCL19 | CD3D | CD2 | CD3G | CCR7 | CALM3 | C7 | CD3D |
| CCL5 | CD3E | CD244 | CD72 | CD19 | CARD11 | CARD18 | CD3G |
| CCR6 | CD3G | CD247 | CD79A | CD1C | CBLB | CASP12 | CD5 |
| CCR7 | CD7 | CD28 | CD8A | CD2 | CCL28 | CASR | CD8A |
| CD19 | CD81 | CD3D | CD8B | CD22 | CD2 | CCL2 | CDK4 |
| CD2 | CD86 | CD3E | FCER2 | CD247 | CD244 | CCL25 | CTLA4 |
| CD22 | CD8A | CD3G | GATA3 | CD28 | CD247 | CCL28 | FASLG |
| CD244 | CX3CR1 | CD4 | HLA-DMA | CD33 | CD37 | CCL8 | GATA3 |
| CD247 | FLT3LG | CD40LG | HLA-DMB | CD3D | CD3G | CCR8 | GNG7 |
| CD3D | FYN | CD5 | HLA-DPA1 | CD3E | CD8A | CCR9 | GZMB |
| CD3E | GATA3 | CD7 | HLA-DQA2 | CD3G | CD8B | CD209 | HLA-DQA2 |
| CD40LG | GZMB | CD8A | HLA-DRA | CD4 | CDK4 | CD80 | HLA-DQB1 |
| CD5 | HLA-DMA | CD8B | HLA-DRB1 | CD40LG | CYBA | CD8B | HSP90AA1 |
| CD7 | HLA-DMB | CTSK | HLA-DRB3 | CD5 | DNM2 | CDH5 | HSP90AB1 |
| CD72 | HLA-DPA1 | CXCR3 | HLA-DRB4 | CD7 | FYN | CFI | ICOS |
| CD74 | ICAM2 | DOCK2 | HLA-DRB5 | CD72 | GATA3 | CLDN1 | IFNG |
| CD79A | IL2RB | FYN | ICOS | CD74 | GNAI2 | CLDN11 | IGH |
| CD79B | IL7R | HCST | IL23A | CD79A | GNB1 | CLDN16 | IL10 |
| CD81 | ITGB7 | HLA-DPB1 | ITK | CD79B | GRB2 | CLDN19 | IL12RB2 |
| CD8A | ITK | ICOS | KLRK1 | CD8A | GSK3A | CLDN2 | IL23A |
| CD99 | KLRD1 | IKBKB | LAT | CD8B | GSN | CLEC4M | IRF3 |
| CDK4 | KLRK1 | IL11RA | LCK | CD99 | GZMB | COL1A1 | ITGB7 |
| CLDN18 | LAT | IL12RB1 | MS4A1 | CDK4 | ICAM2 | COL1A2 | ITK |
| CLDN5 | LCK | IL21R | PRKCQ | CIITA | IKBKG | COL3A1 | KLRD1 |
| CLDN9 | MAF | IL23A | RASGRP1 | CR2 | IL23A | CR2 | KLRK1 |
| CLEC1B | MAP3K7 | IL5RA | RNASEL | CXCR5 | IL2RB | CSF3 | LCK |
| CXCL10 | NFATC2 | IL7R | RRAS2 | CYLD | IL7R | CTNNA2 | MAF |
| CXCL5 | NFATC3 | ITK | SH2D1A | FLT3LG | IRAK1 | CXCL13 | NFATC2 |
| CXCR5 | PRF1 | JAK1 | TRAF5 | FYN | IRF4 | CXCL14 | PDIA3 |
| ESAM | PRKCQ | LAT | ZAP70 | GATA3 | ITGB2 | CXCL6 | PLCG1 |
| F13A1 | RASGRP1 | LCK |  | GNG7 | ITGB7 | CXCL9 | POLR1C |
| F2R | RNF125 | MARCKSL1 |  | HLA-DMA | ITK | DEFB4A | POLR2H |
| F2RL3 | RORA | NCR3 |  | HLA-DMB | JAK3 | DEFB4B | POLR3D |
| FCER1A | RRAS2 | NFATC3 |  | HLA-DOA | KLRD1 | EGR2 | POLR3E |
| FCER2 | RUNX3 | NLRP1 |  | HLA-DPA1 | KLRK1 | F11 | PPP3CC |
| FCGR2B | SH2D1A | NOS3 |  | HLA-DPB1 | LAT | FGA | PRF1 |
| FCGR3A | SH2D1B | PIN1 |  | HLA-DQA1 | LCK | FGG | PRKACB |
| FERMT3 | STAT4 | PLCB2 |  | HLA-DRA | MAF | GNAI1 | RAP1B |
| FGA | TBX21 | PLCG1 |  | HLA-DRB3 | MAP2K2 | GNG12 | RASGRP1 |
| FLT3LG | TLR7 | POLR1C |  | ICAM2 | MAPK3 | GNG4 | RHOH |
| FYN | TRAF5 | POLR2H |  | ICOS | MAPKAPK2 | GP5 | RORA |
| GATA3 | XCL1 | POLR3A |  | ICOSLG | MFN2 | GUCY1A2 | RRAS2 |
| GBP1 | XCL2 | PRKCQ |  | IL11RA | NCR3 | IFNA17 | RUNX3 |
| GBP4 | ZAP70 | PTPN11 |  | IL27RA | PIK3CD | IFNA4 | STAT4 |
| GNB5 |  | RASSF5 |  | IL2RB | PLCG1 | IFNA5 | SUGT1 |
| GNG11 |  | RHOH |  | IL7R | POLR2H | IFNE | TBX21 |
| GNG7 |  | RNF125 |  | ITGB7 | POLR3E | IL12B | TNFRSF13B |
| GNG8 |  | RORA |  | ITK | PPP1CA | IL1A | TRAF5 |
| GP1BA |  | RUNX3 |  | ITPR3 | PPP3CC | IL21 | ZAP70 |
| GP6 |  | TP53BP1 |  | KLRD1 | PRF1 | IL23R |  |
| GRAP2 |  | TYK2 |  | LAT | PRKACA | IL3 |  |
| GRK5 |  | ZAP70 |  | LCK | PRKCD | IL5 |  |
| GZMB |  |  |  | MAF | PTK2B | KITLG |  |
| HCST |  |  |  | MARCKSL1 | RAC2 | MAPK10 |  |
| HLA-DMA |  |  |  | MS4A1 | RASGRP1 | MAPK12 |  |
| HLA-DMB |  |  |  | NCR3 | RFXANK | MASP1 |  |
| HLA-DOA |  |  |  | PLCG1 | RORA | MBL2 |  |
| HLA-DOB |  |  |  | POLR1C | RPS6KB2 | MYLK2 |  |
| HLA-DPA1 |  |  |  | POLR2H | RUNX1 | MYLK3 |  |
| HLA-DQA1 |  |  |  | POLR3C | SH2D1B | MYLK4 |  |
| HLA-DQB1 |  |  |  | PPP1CC | SHC1 | PIK3R3 |  |
| HLA-DRA |  |  |  | PPP3CC | SIPA1 | PLAT |  |
| HSP90AB1 |  |  |  | PRF1 | SPHK2 | PRKACG |  |
| HSPA8 |  |  |  | PRKCQ | STIM1 | PRKG1 |  |
| ICAM2 |  |  |  | PTK2 | TBKBP1 | SCIN |  |
| ICOS |  |  |  | RASGRP1 | TBX21 | SERPIND1 |  |
| IL11RA |  |  |  | RNF125 | TGFB1 | SERPINE1 |  |
| IL27RA |  |  |  | RORA | TOLLIP | TLR3 |  |
| IL2RB |  |  |  | RRAS2 | TRAF2 | TRIP6 |  |
| IL7R |  |  |  | RUNX3 | TRPM2 | ULBP2 |  |
| IRF3 |  |  |  | SH2D1A |  | XCR1 |  |
| IRF5 |  |  |  | SH2D1B |  |  |  |
| ITGA2B |  |  |  | SMAD3 |  |  |  |
| ITGA3 |  |  |  | TIAM1 |  |  |  |
| ITGB3 |  |  |  | TLR7 |  |  |  |
| ITGB7 |  |  |  | TNFRSF13C |  |  |  |
| ITK |  |  |  | TRAF3 |  |  |  |
| ITPR3 |  |  |  | TXK |  |  |  |
| JAM3 |  |  |  | ZAP70 |  |  |  |
| KIR3DL2 |  |  |  |  |  |  |  |
| LAT |  |  |  |  |  |  |  |
| LCK |  |  |  |  |  |  |  |
| MAF |  |  |  |  |  |  |  |
| MS4A1 |  |  |  |  |  |  |  |
| MYL9 |  |  |  |  |  |  |  |
| MYLK |  |  |  |  |  |  |  |
| NCK2 |  |  |  |  |  |  |  |
| NCR3 |  |  |  |  |  |  |  |
| NFATC1 |  |  |  |  |  |  |  |
| NFATC3 |  |  |  |  |  |  |  |
| NOD1 |  |  |  |  |  |  |  |
| P2RX7 |  |  |  |  |  |  |  |
| PF4V1 |  |  |  |  |  |  |  |
| PIP5K1C |  |  |  |  |  |  |  |
| PLA2G4C |  |  |  |  |  |  |  |
| PLCG1 |  |  |  |  |  |  |  |
| POLR1C |  |  |  |  |  |  |  |
| POLR2H |  |  |  |  |  |  |  |
| PPBP |  |  |  |  |  |  |  |
| PRF1 |  |  |  |  |  |  |  |
| PROS1 |  |  |  |  |  |  |  |
| PSME2 |  |  |  |  |  |  |  |
| PTGIR |  |  |  |  |  |  |  |
| PTGS1 |  |  |  |  |  |  |  |
| PTK2 |  |  |  |  |  |  |  |
| RASGRP1 |  |  |  |  |  |  |  |
| RASGRP3 |  |  |  |  |  |  |  |
| RFX5 |  |  |  |  |  |  |  |
| RRAS |  |  |  |  |  |  |  |
| RUNX3 |  |  |  |  |  |  |  |
| SH2D1A |  |  |  |  |  |  |  |
| SHARPIN |  |  |  |  |  |  |  |
| SMAD3 |  |  |  |  |  |  |  |
| SPHK1 |  |  |  |  |  |  |  |
| SRC |  |  |  |  |  |  |  |
| STAT4 |  |  |  |  |  |  |  |
| TAP2 |  |  |  |  |  |  |  |
| TBXA2R |  |  |  |  |  |  |  |
| TFPI |  |  |  |  |  |  |  |
| TLN1 |  |  |  |  |  |  |  |
| TLR7 |  |  |  |  |  |  |  |
| TNFRSF13C |  |  |  |  |  |  |  |
| TRAF5 |  |  |  |  |  |  |  |
| TXK |  |  |  |  |  |  |  |
| VAMP8 |  |  |  |  |  |  |  |
| VDAC2 |  |  |  |  |  |  |  |
| VDAC3 |  |  |  |  |  |  |  |
| VWF |  |  |  |  |  |  |  |
| WASF3 |  |  |  |  |  |  |  |
| ZAP70 |  |  |  |  |  |  |  |

**Supplementary Table S6: Topological analysis from GSE54514 control group**

| Average Shortest Path Length | Between -ness Centrality | Closeness Centrality | Clustering Coefficient | Degree | Eccentricity | name | Neighbor- hood Connectivity | Number Of Directed Edges | Radiality | Stress | Topological Coefficient |
| --- | --- | --- | --- | --- | --- | --- | --- | --- | --- | --- | --- |
| 1.96 | 0.23 | 0.51 | 0.29 | 15.00 | 4.00 | ADCY2 | 8.00 | 15.00 | 0.84 | 1750 | 0.19 |
| 2.44 | 0.06 | 0.41 | 0.36 | 8.00 | 5.00 | ADCY6 | 7.50 | 8.00 | 0.76 | 468 | 0.26 |
| 4.83 | 0.01 | 0.21 | 0.47 | 11.00 | 12.00 | ALOX5 | 15.45 | 11.00 | 0.73 | 27244 | 0.26 |
| 4.19 | 0.01 | 0.24 | 0.45 | 27.00 | 11.00 | ASAP1 | 20.41 | 27.00 | 0.77 | 27808 | 0.23 |
| 4.47 | 0.00 | 0.22 | 0.75 | 11.00 | 11.00 | ANTXR2 | 22.73 | 11.00 | 0.75 | 134 | 0.35 |
| 2.63 | 0.08 | 0.38 | 0.29 | 7.00 | 5.00 | ASAP3 | 6.43 | 7.00 | 0.73 | 574 | 0.27 |
| 4.07 | 0.03 | 0.25 | 0.38 | 13.00 | 8.00 | ATG12 | 9.23 | 13.00 | 0.78 | 37430 | 0.26 |
| 4.00 | 0.04 | 0.25 | 0.36 | 15.00 | 8.00 | ATG5 | 9.33 | 15.00 | 0.79 | 53844 | 0.22 |
| 4.02 | 0.01 | 0.25 | 0.39 | 9.00 | 10.00 | BID | 15.89 | 9.00 | 0.78 | 8938 | 0.22 |
| 3.60 | 0.24 | 0.28 | 0.36 | 14.00 | 8.00 | BIRC2 | 12.29 | 14.00 | 0.81 | 254938 | 0.19 |
| 4.64 | 0.00 | 0.22 | 0.57 | 7.00 | 8.00 | BIRC3 | 8.71 | 7.00 | 0.74 | 2586 | 0.38 |
| 4.94 | 0.02 | 0.20 | 0.33 | 6.00 | 9.00 | BRCC3 | 6.50 | 6.00 | 0.72 | 12656 | 0.36 |
| 1.00 | 0.00 | 1.00 | 0.00 | 1.00 | 1.00 | C4A | 1.00 | 1.00 | 1.00 | 0 | 0.00 |
| 1.00 | 0.00 | 1.00 | 0.00 | 1.00 | 1.00 | C4B | 1.00 | 1.00 | 1.00 | 0 | 0.00 |
| 1.00 | 0.00 | 1.00 | 0.00 | 1.00 | 1.00 | ADCY1 | 1.00 | 1.00 | 1.00 | 0 | 0.00 |
| 1.00 | 0.00 | 1.00 | 0.00 | 1.00 | 1.00 | C8B | 1.00 | 1.00 | 1.00 | 0 | 0.00 |
| 7.08 | 0.00 | 0.14 | 0.75 | 8.00 | 11.00 | CALM1 | 7.50 | 8.00 | 0.57 | 9234 | 0.50 |
| 7.08 | 0.00 | 0.14 | 0.75 | 8.00 | 11.00 | CALM2 | 7.50 | 8.00 | 0.57 | 9234 | 0.50 |
| 6.88 | 0.01 | 0.15 | 0.64 | 9.00 | 11.00 | CALM3 | 7.11 | 9.00 | 0.58 | 10722 | 0.44 |
| 5.51 | 0.00 | 0.18 | 1.00 | 2.00 | 12.00 | CARD16 | 5.50 | 2.00 | 0.68 | 0 | 0.61 |
| 4.83 | 0.00 | 0.21 | 0.17 | 4.00 | 11.00 | CASP1 | 7.50 | 4.00 | 0.73 | 1356 | 0.29 |
| 5.93 | 0.00 | 0.17 | 0.00 | 1.00 | 10.00 | CASP3 | 6.00 | 1.00 | 0.65 | 0 | 0.00 |
| 1.00 | 0.00 | 1.00 | 0.00 | 1.00 | 1.00 | CCL14 | 1.00 | 1.00 | 1.00 | 0 | 0.00 |
| 1.00 | 0.00 | 1.00 | 0.00 | 1.00 | 1.00 | CCL15 | 1.00 | 1.00 | 1.00 | 0 | 0.00 |
| 1.00 | 0.00 | 1.00 | 1.00 | 2.00 | 1.00 | CCL3 | 2.00 | 2.00 | 1.00 | 0 | 1.00 |
| 1.00 | 0.00 | 1.00 | 1.00 | 2.00 | 1.00 | CCL3L1 | 2.00 | 2.00 | 1.00 | 0 | 1.00 |
| 1.00 | 0.00 | 1.00 | 1.00 | 2.00 | 1.00 | CCL3L3 | 2.00 | 2.00 | 1.00 | 0 | 1.00 |
| 3.39 | 0.00 | 0.30 | 1.00 | 2.00 | 6.00 | CCR8 | 6.50 | 2.00 | 0.60 | 0 | 0.72 |
| 7.84 | 0.01 | 0.13 | 0.53 | 6.00 | 12.00 | CD2 | 6.33 | 6.00 | 0.51 | 18680 | 0.53 |
| 8.83 | 0.00 | 0.11 | 1.00 | 2.00 | 13.00 | CD3D | 4.50 | 2.00 | 0.44 | 0 | 0.75 |
| 7.09 | 0.02 | 0.14 | 0.67 | 6.00 | 11.00 | CD3E | 7.17 | 6.00 | 0.56 | 9800 | 0.58 |
| 8.07 | 0.00 | 0.12 | 0.67 | 3.00 | 12.00 | CD3G | 5.33 | 3.00 | 0.50 | 4594 | 0.59 |
| 1.00 | 0.00 | 1.00 | 0.00 | 1.00 | 1.00 | CASP10 | 1.00 | 1.00 | 1.00 | 0 | 0.00 |
| 1.00 | 0.00 | 1.00 | 0.00 | 1.00 | 1.00 | CD4 | 1.00 | 1.00 | 1.00 | 0 | 0.00 |
| 8.08 | 0.01 | 0.12 | 0.00 | 2.00 | 12.00 | CD5 | 3.50 | 2.00 | 0.49 | 4902 | 0.50 |
| 4.67 | 0.00 | 0.21 | 0.43 | 8.00 | 11.00 | C5AR1 | 14.00 | 8.00 | 0.74 | 2934 | 0.28 |
| 4.92 | 0.00 | 0.20 | 1.00 | 2.00 | 11.00 | CD55 | 16.00 | 2.00 | 0.72 | 0 | 0.64 |
| 4.78 | 0.00 | 0.21 | 1.00 | 2.00 | 11.00 | CD59 | 11.00 | 2.00 | 0.73 | 0 | 0.65 |
| 1.20 | 0.70 | 0.83 | 0.17 | 4.00 | 2.00 | CD19 | 1.75 | 4.00 | 0.93 | 14 | 0.42 |
| 2.00 | 0.00 | 0.50 | 0.00 | 1.00 | 3.00 | CD72 | 4.00 | 1.00 | 0.67 | 0 | 0.00 |
| 1.40 | 0.40 | 0.71 | 0.33 | 3.00 | 2.00 | CD79A | 2.33 | 3.00 | 0.87 | 8 | 0.50 |
| 2.20 | 0.00 | 0.45 | 0.00 | 1.00 | 3.00 | CD22 | 3.00 | 1.00 | 0.60 | 0 | 0.00 |
| 3.89 | 0.04 | 0.26 | 0.24 | 12.00 | 9.00 | CHUK | 11.25 | 12.00 | 0.79 | 52878 | 0.19 |
| 2.89 | 0.04 | 0.35 | 0.33 | 3.00 | 5.00 | CLDN11 | 6.33 | 3.00 | 0.69 | 174 | 0.40 |
| 2.28 | 0.08 | 0.44 | 0.25 | 9.00 | 4.00 | CLDN16 | 7.89 | 9.00 | 0.79 | 744 | 0.26 |
| 2.30 | 0.05 | 0.44 | 0.46 | 8.00 | 4.00 | CLDN18 | 9.38 | 8.00 | 0.78 | 584 | 0.28 |
| 1.00 | 0.00 | 1.00 | 0.00 | 1.00 | 1.00 | CCL21 | 1.00 | 1.00 | 1.00 | 0 | 0.00 |
| 1.00 | 0.00 | 1.00 | 0.00 | 1.00 | 1.00 | CLDN3 | 1.00 | 1.00 | 1.00 | 0 | 0.00 |
| 5.03 | 0.02 | 0.20 | 0.33 | 7.00 | 12.00 | CARD6 | 13.43 | 7.00 | 0.71 | 22198 | 0.29 |
| 4.45 | 0.00 | 0.22 | 0.86 | 7.00 | 11.00 | CLEC4D | 24.14 | 7.00 | 0.75 | 2736 | 0.36 |
| 3.61 | 0.00 | 0.28 | 0.00 | 1.00 | 6.00 | CLEC4M | 7.00 | 1.00 | 0.56 | 0 | 0.00 |
| 4.44 | 0.00 | 0.23 | 0.49 | 10.00 | 11.00 | CLEC7A | 21.20 | 10.00 | 0.75 | 5956 | 0.30 |
| 1.98 | 0.26 | 0.50 | 0.23 | 14.00 | 4.00 | COL1A1 | 7.21 | 14.00 | 0.84 | 1846 | 0.18 |
| 2.59 | 0.01 | 0.39 | 0.50 | 4.00 | 5.00 | C7 | 10.75 | 4.00 | 0.73 | 84 | 0.43 |
| 2.83 | 0.00 | 0.35 | 0.67 | 3.00 | 5.00 | COL1A2 | 9.00 | 3.00 | 0.69 | 16 | 0.47 |
| 2.37 | 0.02 | 0.42 | 0.67 | 6.00 | 4.00 | COL3A1 | 11.00 | 6.00 | 0.77 | 290 | 0.38 |
| 4.25 | 0.01 | 0.24 | 0.44 | 29.00 | 11.00 | CR1 | 19.72 | 29.00 | 0.77 | 12822 | 0.22 |
| 3.98 | 0.01 | 0.25 | 0.49 | 16.00 | 10.00 | ARPC5 | 21.81 | 16.00 | 0.79 | 19198 | 0.27 |
| 3.92 | 0.04 | 0.26 | 0.48 | 7.00 | 8.00 | CREB1 | 12.00 | 7.00 | 0.79 | 57984 | 0.29 |
| 4.11 | 0.00 | 0.24 | 0.40 | 6.00 | 10.00 | CRK | 17.50 | 6.00 | 0.78 | 816 | 0.26 |
| 5.30 | 0.00 | 0.19 | 0.00 | 4.00 | 12.00 | CSF2RA | 5.25 | 4.00 | 0.69 | 3238 | 0.28 |
| 4.23 | 0.01 | 0.24 | 0.53 | 6.00 | 10.00 | CD46 | 19.00 | 6.00 | 0.77 | 3412 | 0.34 |
| 4.67 | 0.00 | 0.21 | 0.67 | 7.00 | 11.00 | CXCL1 | 17.57 | 7.00 | 0.74 | 132 | 0.36 |
| 2.93 | 0.00 | 0.34 | 0.00 | 2.00 | 5.00 | CXCL12 | 9.50 | 2.00 | 0.68 | 32 | 0.53 |
| 2.96 | 0.02 | 0.34 | 0.40 | 5.00 | 5.00 | CXCL2 | 5.40 | 5.00 | 0.67 | 152 | 0.33 |
| 3.52 | 0.00 | 0.28 | 0.00 | 2.00 | 5.00 | CLDN8 | 4.00 | 2.00 | 0.58 | 28 | 0.50 |
| 4.32 | 0.01 | 0.23 | 0.50 | 19.00 | 11.00 | CXCR1 | 21.74 | 19.00 | 0.76 | 21902 | 0.27 |
| 3.93 | 0.03 | 0.25 | 0.42 | 24.00 | 10.00 | CXCR2 | 19.71 | 24.00 | 0.79 | 29630 | 0.22 |
| 5.22 | 0.00 | 0.19 | 0.67 | 3.00 | 12.00 | CYBB | 14.33 | 3.00 | 0.70 | 50 | 0.51 |
| 5.02 | 0.29 | 0.20 | 0.14 | 9.00 | 9.00 | CD28 | 3.67 | 9.00 | 0.71 | 259584 | 0.17 |
| 4.47 | 0.28 | 0.22 | 0.20 | 5.00 | 8.00 | CYLD | 6.60 | 5.00 | 0.75 | 287870 | 0.26 |
| 1.00 | 0.00 | 1.00 | 1.00 | 3.00 | 1.00 | DEFA1 | 3.00 | 3.00 | 1.00 | 0 | 1.00 |
| 1.00 | 0.00 | 1.00 | 1.00 | 3.00 | 1.00 | DEFA1B | 3.00 | 3.00 | 1.00 | 0 | 1.00 |
| 1.00 | 0.00 | 1.00 | 1.00 | 3.00 | 1.00 | DEFA3 | 3.00 | 3.00 | 1.00 | 0 | 1.00 |
| 1.00 | 0.00 | 1.00 | 1.00 | 3.00 | 1.00 | DEFA4 | 3.00 | 3.00 | 1.00 | 0 | 1.00 |
| 1.00 | 0.00 | 1.00 | 0.00 | 1.00 | 1.00 | DEFB103A | 1.00 | 1.00 | 1.00 | 0 | 0.00 |
| 1.00 | 0.00 | 1.00 | 0.00 | 1.00 | 1.00 | DEFB103B | 1.00 | 1.00 | 1.00 | 0 | 0.00 |
| 3.09 | 0.00 | 0.32 | 1.00 | 3.00 | 5.00 | DEFB4A | 6.67 | 3.00 | 0.65 | 0 | 0.51 |
| 3.09 | 0.00 | 0.32 | 1.00 | 3.00 | 5.00 | DEFB4B | 6.67 | 3.00 | 0.65 | 0 | 0.51 |
| 5.26 | 0.01 | 0.19 | 0.33 | 4.00 | 9.00 | DLG1 | 4.25 | 4.00 | 0.70 | 9640 | 0.36 |
| 4.73 | 0.00 | 0.21 | 0.83 | 4.00 | 8.00 | DNM1L | 11.00 | 4.00 | 0.73 | 402 | 0.52 |
| 2.53 | 0.21 | 0.40 | 0.00 | 3.00 | 4.00 | ARRB1 | 3.00 | 3.00 | 0.75 | 84 | 0.40 |
| 1.94 | 0.76 | 0.52 | 0.00 | 5.00 | 3.00 | DNM2 | 2.80 | 5.00 | 0.84 | 254 | 0.23 |
| 2.17 | 0.14 | 0.46 | 0.29 | 12.00 | 4.00 | F11 | 7.42 | 12.00 | 0.81 | 1198 | 0.21 |
| 2.94 | 0.01 | 0.34 | 0.50 | 4.00 | 5.00 | CLDN1 | 7.25 | 4.00 | 0.68 | 128 | 0.43 |
| 3.57 | 0.00 | 0.28 | 1.00 | 2.00 | 5.00 | F3 | 6.00 | 2.00 | 0.57 | 0 | 0.67 |
| 5.78 | 0.00 | 0.17 | 1.00 | 2.00 | 13.00 | F5 | 9.50 | 2.00 | 0.66 | 0 | 0.73 |
| 1.00 | 0.00 | 1.00 | 0.00 | 1.00 | 1.00 | CXCL11 | 1.00 | 1.00 | 1.00 | 0 | 0.00 |
| 1.00 | 0.00 | 1.00 | 0.00 | 1.00 | 1.00 | F9 | 1.00 | 1.00 | 1.00 | 0 | 0.00 |
| 3.87 | 0.09 | 0.26 | 0.24 | 13.00 | 10.00 | FAS | 12.62 | 13.00 | 0.79 | 71476 | 0.16 |
| 1.60 | 0.00 | 0.63 | 1.00 | 2.00 | 2.00 | FCER2 | 3.50 | 2.00 | 0.80 | 0 | 0.70 |
| 1.00 | 0.00 | 1.00 | 0.00 | 1.00 | 1.00 | FCGR3A | 1.00 | 1.00 | 1.00 | 0 | 0.00 |
| 1.00 | 0.00 | 1.00 | 0.00 | 1.00 | 1.00 | FCGR3B | 1.00 | 1.00 | 1.00 | 0 | 0.00 |
| 2.83 | 0.00 | 0.35 | 1.00 | 2.00 | 4.00 | FGA | 10.00 | 2.00 | 0.69 | 0 | 0.59 |
| 4.00 | 0.02 | 0.25 | 0.24 | 13.00 | 10.00 | FOS | 17.69 | 13.00 | 0.79 | 13608 | 0.21 |
| 4.47 | 0.00 | 0.22 | 1.00 | 2.00 | 10.00 | CD1D | 13.00 | 2.00 | 0.75 | 0 | 0.57 |
| 6.90 | 0.00 | 0.14 | 0.76 | 7.00 | 11.00 | FYN | 7.71 | 7.00 | 0.58 | 738 | 0.55 |
| 5.90 | 0.00 | 0.17 | 0.00 | 2.00 | 13.00 | ACTN1 | 5.00 | 2.00 | 0.65 | 14760 | 0.50 |
| 4.97 | 0.01 | 0.20 | 0.10 | 7.00 | 12.00 | GAB2 | 10.86 | 7.00 | 0.72 | 30644 | 0.24 |
| 5.58 | 0.00 | 0.18 | 1.00 | 2.00 | 12.00 | GABARAPL1 | 8.00 | 2.00 | 0.67 | 0 | 0.67 |
| 5.45 | 0.11 | 0.18 | 0.19 | 7.00 | 9.00 | CBLB | 4.14 | 7.00 | 0.68 | 115040 | 0.21 |
| 5.61 | 0.00 | 0.18 | 0.67 | 3.00 | 9.00 | GATA3 | 5.00 | 3.00 | 0.67 | 5236 | 0.56 |
| 1.00 | 0.00 | 1.00 | 0.00 | 1.00 | 1.00 | GBP1 | 1.00 | 1.00 | 1.00 | 0 | 0.00 |
| 1.00 | 0.00 | 1.00 | 0.00 | 1.00 | 1.00 | GBP5 | 1.00 | 1.00 | 1.00 | 0 | 0.00 |
| 3.94 | 0.03 | 0.25 | 0.44 | 13.00 | 9.00 | GNA13 | 12.62 | 13.00 | 0.79 | 43910 | 0.24 |
| 4.21 | 0.01 | 0.24 | 0.30 | 5.00 | 10.00 | GNAI3 | 15.60 | 5.00 | 0.77 | 2690 | 0.30 |
| 3.80 | 0.04 | 0.26 | 0.37 | 32.00 | 10.00 | GNAQ | 19.56 | 32.00 | 0.80 | 62566 | 0.20 |
| 4.64 | 0.00 | 0.22 | 0.50 | 4.00 | 11.00 | CCR1 | 15.75 | 4.00 | 0.74 | 1416 | 0.38 |
| 3.99 | 0.02 | 0.25 | 0.35 | 11.00 | 10.00 | GNB4 | 18.36 | 11.00 | 0.79 | 11132 | 0.23 |
| 4.40 | 0.00 | 0.23 | 0.30 | 5.00 | 10.00 | DAPP1 | 10.40 | 5.00 | 0.76 | 490 | 0.32 |
| 6.21 | 0.01 | 0.16 | 0.00 | 2.00 | 13.00 | CTNNA1 | 2.50 | 2.00 | 0.63 | 5896 | 0.50 |
| 7.21 | 0.00 | 0.14 | 0.00 | 1.00 | 14.00 | GNG10 | 2.00 | 1.00 | 0.56 | 0 | 0.00 |
| 1.00 | 0.00 | 1.00 | 0.00 | 1.00 | 1.00 | ESAM | 1.00 | 1.00 | 1.00 | 0 | 0.00 |
| 1.00 | 0.00 | 1.00 | 0.00 | 1.00 | 1.00 | GNG11 | 1.00 | 1.00 | 1.00 | 0 | 0.00 |
| 2.93 | 0.01 | 0.34 | 0.60 | 5.00 | 5.00 | CCL11 | 7.00 | 5.00 | 0.68 | 54 | 0.41 |
| 2.61 | 0.04 | 0.38 | 0.33 | 9.00 | 5.00 | GNG4 | 7.22 | 9.00 | 0.73 | 392 | 0.27 |
| 3.47 | 0.00 | 0.29 | 0.00 | 1.00 | 5.00 | GNG7 | 3.00 | 1.00 | 0.59 | 0 | 0.00 |
| 2.78 | 0.00 | 0.36 | 1.00 | 3.00 | 5.00 | GP5 | 10.00 | 3.00 | 0.70 | 0 | 0.48 |
| 6.62 | 0.00 | 0.15 | 0.00 | 3.00 | 14.00 | GRB2 | 3.33 | 3.00 | 0.60 | 120 | 0.33 |
| 5.88 | 0.01 | 0.17 | 0.20 | 5.00 | 13.00 | CSF3R | 4.80 | 5.00 | 0.65 | 18098 | 0.28 |
| 3.46 | 0.04 | 0.29 | 0.00 | 2.00 | 5.00 | F12 | 3.50 | 2.00 | 0.59 | 214 | 0.50 |
| 4.44 | 0.00 | 0.23 | 0.00 | 1.00 | 6.00 | GRK6 | 2.00 | 1.00 | 0.43 | 0 | 0.00 |
| 2.80 | 0.05 | 0.36 | 0.00 | 3.00 | 4.00 | GUCY1A2 | 6.00 | 3.00 | 0.70 | 342 | 0.33 |
| 3.65 | 0.01 | 0.27 | 0.00 | 2.00 | 5.00 | FGB | 3.00 | 2.00 | 0.56 | 60 | 0.50 |
| 2.14 | 0.00 | 0.47 | 1.00 | 2.00 | 3.00 | CCL4 | 3.50 | 2.00 | 0.62 | 0 | 0.88 |
| 1.57 | 0.07 | 0.64 | 0.67 | 3.00 | 2.00 | GZMB | 4.00 | 3.00 | 0.81 | 8 | 0.57 |
| 6.26 | 0.00 | 0.16 | 0.00 | 1.00 | 13.00 | ARRB2 | 3.00 | 1.00 | 0.62 | 0 | 0.00 |
| 5.26 | 0.01 | 0.19 | 0.00 | 3.00 | 12.00 | HCK | 7.67 | 3.00 | 0.70 | 7810 | 0.35 |
| 1.00 | 0.00 | 1.00 | 0.00 | 1.00 | 1.00 | HLA-DQA1 | 1.00 | 1.00 | 1.00 | 0 | 0.00 |
| 1.00 | 0.00 | 1.00 | 0.00 | 1.00 | 1.00 | HLA-DQB1 | 1.00 | 1.00 | 1.00 | 0 | 0.00 |
| 1.00 | 0.00 | 1.00 | 1.00 | 2.00 | 1.00 | HLA-DRB1 | 2.00 | 2.00 | 1.00 | 0 | 1.00 |
| 1.00 | 0.00 | 1.00 | 1.00 | 2.00 | 1.00 | HLA-DRB3 | 2.00 | 2.00 | 1.00 | 0 | 1.00 |
| 1.00 | 0.00 | 1.00 | 1.00 | 2.00 | 1.00 | HLA-DRB5 | 2.00 | 2.00 | 1.00 | 0 | 1.00 |
| 9.47 | 0.00 | 0.11 | 0.00 | 1.00 | 14.00 | GRAP2 | 4.00 | 1.00 | 0.40 | 0 | 0.00 |
| 8.47 | 0.02 | 0.12 | 0.17 | 4.00 | 13.00 | HSP90AB1 | 2.75 | 4.00 | 0.47 | 19450 | 0.32 |
| 4.36 | 0.00 | 0.23 | 0.63 | 14.00 | 11.00 | HSPA1A | 21.50 | 14.00 | 0.76 | 1272 | 0.31 |
| 4.36 | 0.00 | 0.23 | 0.63 | 14.00 | 11.00 | HSPA1B | 21.50 | 14.00 | 0.76 | 1272 | 0.31 |
| 4.69 | 0.00 | 0.21 | 0.50 | 4.00 | 9.00 | HSPA4 | 7.75 | 4.00 | 0.74 | 2554 | 0.44 |
| 4.86 | 0.00 | 0.21 | 0.00 | 2.00 | 11.00 | FCGR1A | 9.50 | 2.00 | 0.72 | 2396 | 0.50 |
| 5.56 | 0.01 | 0.18 | 0.33 | 4.00 | 12.00 | IFI16 | 3.75 | 4.00 | 0.67 | 4894 | 0.35 |
| 5.63 | 0.01 | 0.18 | 0.33 | 4.00 | 12.00 | GBP2 | 3.50 | 4.00 | 0.67 | 7970 | 0.36 |
| 5.19 | 0.01 | 0.19 | 0.00 | 2.00 | 10.00 | DDX58 | 3.50 | 2.00 | 0.70 | 2262 | 0.50 |
| 6.19 | 0.00 | 0.16 | 0.00 | 1.00 | 11.00 | IFIH1 | 2.00 | 1.00 | 0.63 | 0 | 0.00 |
| 6.55 | 0.00 | 0.15 | 1.00 | 2.00 | 13.00 | IFITM1 | 4.00 | 2.00 | 0.60 | 0 | 0.80 |
| 2.35 | 0.12 | 0.43 | 0.24 | 11.00 | 4.00 | IFNA1 | 6.64 | 11.00 | 0.77 | 988 | 0.24 |
| 3.17 | 0.00 | 0.32 | 0.00 | 2.00 | 5.00 | GNG12 | 9.50 | 2.00 | 0.64 | 32 | 0.61 |
| 3.30 | 0.00 | 0.30 | 1.00 | 2.00 | 5.00 | IFNA13 | 8.00 | 2.00 | 0.62 | 0 | 0.67 |
| 3.43 | 0.00 | 0.29 | 1.00 | 2.00 | 6.00 | CXCL9 | 5.00 | 2.00 | 0.60 | 0 | 0.71 |
| 3.13 | 0.00 | 0.32 | 0.67 | 3.00 | 6.00 | IFNA14 | 5.33 | 3.00 | 0.65 | 44 | 0.48 |
| 3.91 | 0.00 | 0.26 | 0.00 | 1.00 | 6.00 | CLDN19 | 2.00 | 1.00 | 0.52 | 0 | 0.00 |
| 2.93 | 0.04 | 0.34 | 0.00 | 2.00 | 5.00 | IFNA16 | 7.50 | 2.00 | 0.68 | 178 | 0.50 |
| 3.02 | 0.00 | 0.33 | 1.00 | 3.00 | 5.00 | IFNA5 | 8.00 | 3.00 | 0.66 | 0 | 0.57 |
| 4.36 | 0.02 | 0.23 | 0.43 | 14.00 | 11.00 | IFNAR1 | 19.36 | 14.00 | 0.76 | 23988 | 0.26 |
| 3.61 | 0.00 | 0.28 | 0.00 | 1.00 | 6.00 | IFNE | 7.00 | 1.00 | 0.56 | 0 | 0.00 |
| 3.61 | 0.18 | 0.28 | 0.25 | 49.00 | 10.00 | IFNGR1 | 16.53 | 49.00 | 0.81 | 183336 | 0.14 |
| 4.23 | 0.00 | 0.24 | 1.00 | 3.00 | 10.00 | APBB1IP | 22.33 | 3.00 | 0.77 | 0 | 0.42 |
| 4.51 | 0.00 | 0.22 | 0.56 | 10.00 | 11.00 | IFNGR2 | 18.70 | 10.00 | 0.75 | 538 | 0.30 |
| 8.47 | 0.00 | 0.12 | 0.00 | 2.00 | 13.00 | BCL2 | 5.00 | 2.00 | 0.47 | 34 | 0.50 |
| 8.44 | 0.00 | 0.12 | 0.50 | 4.00 | 13.00 | IKBKB | 6.25 | 4.00 | 0.47 | 96 | 0.55 |
| 3.24 | 0.12 | 0.31 | 0.00 | 2.00 | 5.00 | GSK3A | 2.50 | 2.00 | 0.63 | 36 | 0.50 |
| 4.18 | 0.00 | 0.24 | 0.00 | 1.00 | 6.00 | IKBKG | 2.00 | 1.00 | 0.47 | 0 | 0.00 |
| 4.32 | 0.03 | 0.23 | 0.47 | 17.00 | 11.00 | IL1B | 20.41 | 17.00 | 0.76 | 27624 | 0.25 |
| 4.66 | 0.01 | 0.21 | 0.25 | 8.00 | 11.00 | IL1R1 | 12.75 | 8.00 | 0.74 | 4358 | 0.26 |
| 4.51 | 0.00 | 0.22 | 0.63 | 13.00 | 11.00 | IL1R2 | 21.54 | 13.00 | 0.75 | 2228 | 0.33 |
| 3.95 | 0.02 | 0.25 | 0.48 | 14.00 | 10.00 | IL1RAP | 21.86 | 14.00 | 0.79 | 45162 | 0.25 |
| 5.81 | 0.17 | 0.17 | 0.17 | 4.00 | 10.00 | IL21R | 5.75 | 4.00 | 0.66 | 163668 | 0.28 |
| 6.74 | 0.03 | 0.15 | 0.17 | 4.00 | 11.00 | GNB5 | 2.50 | 4.00 | 0.59 | 24166 | 0.33 |
| 4.35 | 0.02 | 0.23 | 0.38 | 19.00 | 11.00 | IL6R | 17.00 | 19.00 | 0.76 | 12732 | 0.22 |
| 6.25 | 0.00 | 0.16 | 1.00 | 2.00 | 10.00 | CCR7 | 3.50 | 2.00 | 0.63 | 0 | 0.88 |
| 5.26 | 0.01 | 0.19 | 0.50 | 4.00 | 9.00 | IL6ST | 4.75 | 4.00 | 0.70 | 9636 | 0.40 |
| 5.06 | 0.02 | 0.20 | 0.00 | 4.00 | 11.00 | IRAK4 | 3.50 | 4.00 | 0.71 | 4552 | 0.25 |
| 6.06 | 0.00 | 0.17 | 0.00 | 1.00 | 12.00 | CD36 | 4.00 | 1.00 | 0.64 | 0 | 0.00 |
| 4.70 | 0.00 | 0.21 | 0.00 | 2.00 | 10.00 | IRF1 | 11.50 | 2.00 | 0.74 | 56 | 0.58 |
| 1.63 | 0.01 | 0.62 | 0.83 | 4.00 | 3.00 | CLU | 4.75 | 4.00 | 0.84 | 2 | 0.68 |
| 1.50 | 0.07 | 0.67 | 0.67 | 4.00 | 2.00 | ITGA2B | 4.75 | 4.00 | 0.88 | 12 | 0.59 |
| 5.26 | 0.00 | 0.19 | 0.00 | 2.00 | 9.00 | ITGA4 | 6.00 | 2.00 | 0.70 | 60 | 0.56 |
| 6.01 | 0.00 | 0.17 | 1.00 | 2.00 | 10.00 | ITGA6 | 6.50 | 2.00 | 0.64 | 0 | 0.65 |
| 5.22 | 0.02 | 0.19 | 0.50 | 4.00 | 12.00 | ITGAM | 10.75 | 4.00 | 0.70 | 11788 | 0.44 |
| 6.66 | 0.03 | 0.15 | 0.00 | 2.00 | 11.00 | AKT3 | 4.00 | 2.00 | 0.60 | 3874 | 0.50 |
| 6.71 | 0.02 | 0.15 | 0.40 | 5.00 | 11.00 | ITGB1 | 6.40 | 5.00 | 0.59 | 3976 | 0.48 |
| 1.25 | 0.33 | 0.80 | 0.47 | 6.00 | 2.00 | ITGB3 | 3.67 | 6.00 | 0.94 | 28 | 0.50 |
| 2.13 | 0.00 | 0.47 | 0.00 | 1.00 | 3.00 | GP1BA | 6.00 | 1.00 | 0.72 | 0 | 0.00 |
| 1.00 | 0.00 | 1.00 | 0.00 | 1.00 | 1.00 | EZR | 1.00 | 1.00 | 1.00 | 0 | 0.00 |
| 1.00 | 0.00 | 1.00 | 0.00 | 1.00 | 1.00 | ITGB7 | 1.00 | 1.00 | 1.00 | 0 | 0.00 |
| 4.72 | 0.09 | 0.21 | 0.33 | 4.00 | 8.00 | ITK | 6.25 | 4.00 | 0.73 | 82850 | 0.32 |
| 6.89 | 0.00 | 0.15 | 0.00 | 1.00 | 11.00 | BLNK | 4.00 | 1.00 | 0.58 | 0 | 0.00 |
| 5.89 | 0.06 | 0.17 | 0.17 | 4.00 | 10.00 | ITPR1 | 4.75 | 4.00 | 0.65 | 48358 | 0.28 |
| 4.03 | 0.03 | 0.25 | 0.43 | 8.00 | 9.00 | ITPR2 | 9.63 | 8.00 | 0.78 | 27644 | 0.27 |
| 4.89 | 0.00 | 0.20 | 0.00 | 2.00 | 11.00 | JAG1 | 12.00 | 2.00 | 0.72 | 16 | 0.55 |
| 6.25 | 0.00 | 0.16 | 0.00 | 1.00 | 10.00 | JAK1 | 4.00 | 1.00 | 0.62 | 0 | 0.00 |
| 4.87 | 0.00 | 0.21 | 0.00 | 1.00 | 11.00 | JAK2 | 13.00 | 1.00 | 0.72 | 0 | 0.00 |
| 7.48 | 0.01 | 0.13 | 0.30 | 5.00 | 12.00 | CARD11 | 7.40 | 5.00 | 0.54 | 19774 | 0.41 |
| 7.50 | 0.02 | 0.13 | 0.33 | 10.00 | 12.00 | JMJD7-PLA2G4B | 5.10 | 10.00 | 0.54 | 19692 | 0.32 |
| 7.55 | 0.00 | 0.13 | 0.80 | 5.00 | 12.00 | ICAM2 | 7.60 | 5.00 | 0.53 | 20 | 0.54 |
| 1.67 | 0.00 | 0.60 | 1.00 | 4.00 | 2.00 | KIR2DL1 | 12.00 | 4.00 | 0.67 | 0 | 1.00 |
| 1.00 | 0.03 | 1.00 | 0.88 | 12.00 | 1.00 | KIR2DL2 | 10.67 | 12.00 | 1.00 | 16 | 0.89 |
| 1.00 | 0.03 | 1.00 | 0.88 | 12.00 | 1.00 | KIR2DL3 | 10.67 | 12.00 | 1.00 | 16 | 0.89 |
| 1.08 | 0.00 | 0.92 | 1.00 | 11.00 | 2.00 | KIR2DL4 | 11.36 | 11.00 | 0.96 | 0 | 0.95 |
| 1.08 | 0.00 | 0.92 | 1.00 | 11.00 | 2.00 | KIR2DL5A | 11.36 | 11.00 | 0.96 | 0 | 0.95 |
| 1.00 | 0.03 | 1.00 | 0.88 | 12.00 | 1.00 | KIR2DS1 | 10.67 | 12.00 | 1.00 | 16 | 0.89 |
| 1.08 | 0.00 | 0.92 | 1.00 | 11.00 | 2.00 | KIR2DS2 | 11.36 | 11.00 | 0.96 | 0 | 0.95 |
| 1.08 | 0.00 | 0.92 | 1.00 | 11.00 | 2.00 | KIR2DS3 | 11.36 | 11.00 | 0.96 | 0 | 0.95 |
| 1.08 | 0.00 | 0.92 | 1.00 | 11.00 | 2.00 | KIR2DS4 | 11.36 | 11.00 | 0.96 | 0 | 0.95 |
| 1.08 | 0.00 | 0.92 | 1.00 | 11.00 | 2.00 | KIR2DS5 | 11.36 | 11.00 | 0.96 | 0 | 0.95 |
| 1.00 | 0.03 | 1.00 | 0.88 | 12.00 | 1.00 | KIR3DL1 | 10.67 | 12.00 | 1.00 | 16 | 0.89 |
| 1.08 | 0.00 | 0.92 | 1.00 | 11.00 | 2.00 | KIR3DL2 | 11.36 | 11.00 | 0.96 | 0 | 0.95 |
| 1.08 | 0.00 | 0.92 | 1.00 | 11.00 | 2.00 | KIR3DL3 | 11.36 | 11.00 | 0.96 | 0 | 0.95 |
| 2.70 | 0.02 | 0.37 | 0.40 | 5.00 | 5.00 | KITLG | 7.60 | 5.00 | 0.72 | 196 | 0.35 |
| 1.00 | 0.00 | 1.00 | 0.00 | 1.00 | 1.00 | KLRC1 | 1.00 | 1.00 | 1.00 | 0 | 0.00 |
| 1.00 | 0.00 | 1.00 | 0.00 | 1.00 | 1.00 | KLRC2 | 1.00 | 1.00 | 1.00 | 0 | 0.00 |
| 1.57 | 0.00 | 0.64 | 1.00 | 4.00 | 3.00 | CX3CR1 | 4.75 | 4.00 | 0.81 | 0 | 0.79 |
| 1.57 | 0.00 | 0.64 | 1.00 | 4.00 | 3.00 | KLRC4-KLRK1 | 4.75 | 4.00 | 0.81 | 0 | 0.79 |
| 1.14 | 0.33 | 0.88 | 0.53 | 6.00 | 2.00 | KLRD1 | 4.00 | 6.00 | 0.95 | 26 | 0.57 |
| 1.57 | 0.00 | 0.64 | 1.00 | 4.00 | 3.00 | KLRK1 | 4.75 | 4.00 | 0.81 | 0 | 0.79 |
| 4.24 | 0.00 | 0.24 | 0.79 | 8.00 | 10.00 | KRAS | 16.88 | 8.00 | 0.77 | 624 | 0.31 |
| 9.08 | 0.00 | 0.11 | 0.00 | 1.00 | 13.00 | LAT | 2.00 | 1.00 | 0.42 | 0 | 0.00 |
| 7.10 | 0.00 | 0.14 | 0.93 | 6.00 | 11.00 | LCK | 8.00 | 6.00 | 0.56 | 6 | 0.67 |
| 4.20 | 0.03 | 0.24 | 0.20 | 6.00 | 9.00 | LCP2 | 8.50 | 6.00 | 0.77 | 12414 | 0.25 |
| 4.39 | 0.02 | 0.23 | 0.42 | 12.00 | 11.00 | LILRB3 | 18.50 | 12.00 | 0.76 | 17770 | 0.26 |
| 4.48 | 0.01 | 0.22 | 0.58 | 10.00 | 11.00 | LIMK2 | 20.80 | 10.00 | 0.75 | 3196 | 0.33 |
| 5.35 | 0.00 | 0.19 | 0.00 | 1.00 | 12.00 | LTBR | 14.00 | 1.00 | 0.69 | 0 | 0.00 |
| 4.36 | 0.02 | 0.23 | 0.35 | 16.00 | 11.00 | LY96 | 15.94 | 16.00 | 0.76 | 17230 | 0.22 |
| 5.36 | 0.00 | 0.19 | 0.00 | 1.00 | 12.00 | CASP5 | 16.00 | 1.00 | 0.69 | 0 | 0.00 |
| 4.17 | 0.03 | 0.24 | 0.42 | 27.00 | 11.00 | LYN | 20.37 | 27.00 | 0.77 | 42120 | 0.23 |
| 1.00 | 0.00 | 1.00 | 0.00 | 1.00 | 1.00 | CCL13 | 1.00 | 1.00 | 1.00 | 0 | 0.00 |
| 1.00 | 0.00 | 1.00 | 0.00 | 1.00 | 1.00 | MADCAM1 | 1.00 | 1.00 | 1.00 | 0 | 0.00 |
| 4.82 | 0.00 | 0.21 | 0.80 | 5.00 | 8.00 | MALT1 | 9.20 | 5.00 | 0.73 | 6 | 0.54 |
| 4.83 | 0.00 | 0.21 | 0.00 | 2.00 | 10.00 | MAML1 | 9.50 | 2.00 | 0.73 | 28 | 0.61 |
| 4.20 | 0.10 | 0.24 | 0.17 | 4.00 | 8.00 | MAML2 | 7.75 | 4.00 | 0.77 | 114978 | 0.32 |
| 2.41 | 0.42 | 0.41 | 0.00 | 4.00 | 4.00 | MAP2K2 | 2.25 | 4.00 | 0.76 | 130 | 0.25 |
| 3.35 | 0.00 | 0.30 | 0.00 | 1.00 | 5.00 | F2 | 4.00 | 1.00 | 0.61 | 0 | 0.00 |
| 3.98 | 0.01 | 0.25 | 0.46 | 14.00 | 10.00 | MAP2K4 | 20.57 | 14.00 | 0.79 | 24102 | 0.25 |
| 1.00 | 0.00 | 1.00 | 0.00 | 1.00 | 1.00 | AKT2 | 1.00 | 1.00 | 1.00 | 0 | 0.00 |
| 1.00 | 0.00 | 1.00 | 0.00 | 1.00 | 1.00 | MAP2K7 | 1.00 | 1.00 | 1.00 | 0 | 0.00 |
| 4.19 | 0.06 | 0.24 | 0.41 | 13.00 | 8.00 | MAP3K7 | 8.31 | 13.00 | 0.77 | 59114 | 0.27 |
| 4.21 | 0.00 | 0.24 | 0.67 | 4.00 | 10.00 | MAP3K8 | 20.00 | 4.00 | 0.77 | 22 | 0.37 |
| 3.97 | 0.02 | 0.25 | 0.30 | 15.00 | 10.00 | MAPK1 | 15.73 | 15.00 | 0.79 | 20506 | 0.19 |
| 4.33 | 0.02 | 0.23 | 0.36 | 20.00 | 11.00 | MAPK14 | 17.40 | 20.00 | 0.76 | 15840 | 0.23 |
| 3.26 | 0.00 | 0.31 | 0.00 | 1.00 | 5.00 | MASP1 | 9.00 | 1.00 | 0.62 | 0 | 0.00 |
| 6.78 | 0.00 | 0.15 | 1.00 | 2.00 | 11.00 | MAVS | 4.00 | 2.00 | 0.59 | 0 | 0.67 |
| 4.21 | 0.01 | 0.24 | 0.44 | 11.00 | 10.00 | MDM2 | 12.73 | 11.00 | 0.77 | 6270 | 0.24 |
| 5.21 | 0.00 | 0.19 | 0.00 | 1.00 | 11.00 | MCU | 11.00 | 1.00 | 0.70 | 0 | 0.00 |
| 6.06 | 0.00 | 0.17 | 0.00 | 1.00 | 12.00 | MFN1 | 4.00 | 1.00 | 0.64 | 0 | 0.00 |
| 4.37 | 0.01 | 0.23 | 0.44 | 19.00 | 11.00 | MME | 18.95 | 19.00 | 0.76 | 9744 | 0.26 |
| 2.22 | 0.09 | 0.45 | 0.36 | 10.00 | 4.00 | MMP2 | 8.40 | 10.00 | 0.80 | 768 | 0.25 |
| 5.31 | 0.00 | 0.19 | 0.33 | 3.00 | 12.00 | MMP9 | 9.00 | 3.00 | 0.69 | 60 | 0.48 |
| 2.00 | 0.00 | 0.50 | 0.00 | 1.00 | 3.00 | MS4A1 | 4.00 | 1.00 | 0.67 | 0 | 0.00 |
| 4.64 | 0.00 | 0.22 | 0.43 | 8.00 | 11.00 | MYD88 | 17.00 | 8.00 | 0.74 | 1788 | 0.32 |
| 5.36 | 0.00 | 0.19 | 0.00 | 3.00 | 12.00 | CTSS | 5.67 | 3.00 | 0.69 | 50 | 0.36 |
| 4.32 | 0.00 | 0.23 | 0.53 | 6.00 | 10.00 | HIF1A | 15.33 | 6.00 | 0.76 | 2332 | 0.31 |
| 6.45 | 0.00 | 0.16 | 0.00 | 1.00 | 10.00 | MYL12A | 7.00 | 1.00 | 0.61 | 0 | 0.00 |
| 2.00 | 0.00 | 0.50 | 0.00 | 1.00 | 3.00 | ARPC2 | 2.00 | 1.00 | 0.67 | 0 | 0.00 |
| 1.33 | 0.67 | 0.75 | 0.00 | 2.00 | 2.00 | MYL12B | 1.50 | 2.00 | 0.89 | 4 | 0.50 |
| 1.33 | 0.67 | 0.75 | 0.00 | 2.00 | 2.00 | HSP90AA1 | 1.50 | 2.00 | 0.89 | 4 | 0.50 |
| 1.50 | 0.07 | 0.67 | 0.67 | 4.00 | 2.00 | MYL9 | 4.75 | 4.00 | 0.88 | 12 | 0.59 |
| 2.52 | 0.12 | 0.40 | 0.20 | 6.00 | 4.00 | MYLK2 | 6.00 | 6.00 | 0.75 | 736 | 0.27 |
| 3.50 | 0.00 | 0.29 | 0.00 | 1.00 | 5.00 | MBL2 | 6.00 | 1.00 | 0.58 | 0 | 0.00 |
| 2.70 | 0.07 | 0.37 | 0.40 | 5.00 | 4.00 | MYLK4 | 7.00 | 5.00 | 0.72 | 768 | 0.38 |
| 1.00 | 0.00 | 1.00 | 0.00 | 1.00 | 1.00 | JAM2 | 1.00 | 1.00 | 1.00 | 0 | 0.00 |
| 1.00 | 0.00 | 1.00 | 0.00 | 1.00 | 1.00 | MYO10 | 1.00 | 1.00 | 1.00 | 0 | 0.00 |
| 4.22 | 0.02 | 0.24 | 0.35 | 30.00 | 11.00 | NAMPT | 17.93 | 30.00 | 0.77 | 15076 | 0.20 |
| 5.22 | 0.00 | 0.19 | 0.00 | 1.00 | 12.00 | MARCKS | 30.00 | 1.00 | 0.70 | 0 | 0.00 |
| 4.36 | 0.00 | 0.23 | 0.55 | 19.00 | 11.00 | NCF2 | 21.68 | 19.00 | 0.76 | 1420 | 0.28 |
| 5.00 | 0.01 | 0.20 | 0.25 | 8.00 | 12.00 | NCF4 | 13.50 | 8.00 | 0.71 | 21612 | 0.27 |
| 4.65 | 0.00 | 0.22 | 0.80 | 6.00 | 8.00 | NCK1 | 11.17 | 6.00 | 0.74 | 240 | 0.49 |
| 4.40 | 0.00 | 0.23 | 0.57 | 7.00 | 10.00 | NEK7 | 12.29 | 7.00 | 0.76 | 1510 | 0.35 |
| 1.00 | 0.00 | 1.00 | 0.00 | 1.00 | 1.00 | HSPA6 | 1.00 | 1.00 | 1.00 | 0 | 0.00 |
| 1.00 | 0.00 | 1.00 | 0.00 | 1.00 | 1.00 | NFKBIA | 1.00 | 1.00 | 1.00 | 0 | 0.00 |
| 4.10 | 0.01 | 0.24 | 0.64 | 9.00 | 8.00 | NFYB | 11.56 | 9.00 | 0.78 | 14834 | 0.34 |
| 4.06 | 0.00 | 0.25 | 0.00 | 1.00 | 6.00 | GNAI2 | 3.00 | 1.00 | 0.49 | 0 | 0.00 |
| 3.12 | 0.23 | 0.32 | 0.00 | 3.00 | 5.00 | NFYC | 1.67 | 3.00 | 0.65 | 70 | 0.33 |
| 4.31 | 0.02 | 0.23 | 0.48 | 18.00 | 11.00 | NLRC4 | 20.56 | 18.00 | 0.76 | 19902 | 0.24 |
| 1.00 | 0.00 | 1.00 | 0.00 | 1.00 | 1.00 | IL1A | 1.00 | 1.00 | 1.00 | 0 | 0.00 |
| 1.00 | 0.00 | 1.00 | 0.00 | 1.00 | 1.00 | NLRP1 | 1.00 | 1.00 | 1.00 | 0 | 0.00 |
| 4.45 | 0.01 | 0.22 | 0.38 | 11.00 | 11.00 | NLRP12 | 17.73 | 11.00 | 0.75 | 7454 | 0.28 |
| 1.50 | 0.00 | 0.67 | 0.00 | 1.00 | 2.00 | FERMT3 | 2.00 | 1.00 | 0.75 | 0 | 0.00 |
| 1.00 | 1.00 | 1.00 | 0.00 | 2.00 | 1.00 | NOTCH1 | 1.00 | 2.00 | 1.00 | 2 | 0.00 |
| 4.90 | 0.00 | 0.20 | 0.43 | 8.00 | 12.00 | NOTCH2 | 17.38 | 8.00 | 0.72 | 19530 | 0.33 |
| 1.00 | 0.00 | 1.00 | 0.00 | 1.00 | 1.00 | ISG15 | 1.00 | 1.00 | 1.00 | 0 | 0.00 |
| 1.00 | 0.00 | 1.00 | 0.00 | 1.00 | 1.00 | OAS2 | 1.00 | 1.00 | 1.00 | 0 | 0.00 |
| 1.00 | 0.00 | 1.00 | 0.00 | 1.00 | 1.00 | ARAF | 1.00 | 1.00 | 1.00 | 0 | 0.00 |
| 1.00 | 0.00 | 1.00 | 0.00 | 1.00 | 1.00 | ORAI1 | 1.00 | 1.00 | 1.00 | 0 | 0.00 |
| 5.93 | 0.00 | 0.17 | 0.00 | 1.00 | 10.00 | P2RY1 | 6.00 | 1.00 | 0.65 | 0 | 0.00 |
| 5.44 | 0.00 | 0.18 | 0.00 | 1.00 | 12.00 | PAK1 | 11.00 | 1.00 | 0.68 | 0 | 0.00 |
| 5.10 | 0.00 | 0.20 | 0.00 | 2.00 | 12.00 | PAK2 | 17.50 | 2.00 | 0.71 | 62 | 0.53 |
| 1.50 | 0.26 | 0.67 | 0.50 | 5.00 | 3.00 | PF4 | 3.80 | 5.00 | 0.88 | 24 | 0.60 |
| 3.90 | 0.04 | 0.26 | 0.35 | 15.00 | 10.00 | PIK3AP1 | 17.40 | 15.00 | 0.79 | 39122 | 0.20 |
| 4.15 | 0.00 | 0.24 | 0.33 | 4.00 | 9.00 | PIK3CB | 11.50 | 4.00 | 0.78 | 3728 | 0.34 |
| 5.06 | 0.01 | 0.20 | 0.20 | 5.00 | 11.00 | PIK3CD | 7.20 | 5.00 | 0.71 | 2552 | 0.30 |
| 5.63 | 0.01 | 0.18 | 0.33 | 3.00 | 9.00 | PIK3R1 | 4.00 | 3.00 | 0.67 | 3212 | 0.46 |
| 3.35 | 0.00 | 0.30 | 0.00 | 1.00 | 5.00 | PIK3R2 | 4.00 | 1.00 | 0.61 | 0 | 0.00 |
| 1.00 | 0.00 | 1.00 | 0.00 | 1.00 | 1.00 | ARPC1A | 1.00 | 1.00 | 1.00 | 0 | 0.00 |
| 1.00 | 0.00 | 1.00 | 0.00 | 1.00 | 1.00 | PIP5K1C | 1.00 | 1.00 | 1.00 | 0 | 0.00 |
| 6.02 | 0.00 | 0.17 | 0.00 | 1.00 | 13.00 | PLA2G4A | 7.00 | 1.00 | 0.64 | 0 | 0.00 |
| 7.50 | 0.02 | 0.13 | 0.33 | 10.00 | 12.00 | PLA2G4B | 5.10 | 10.00 | 0.54 | 19692 | 0.32 |
| 3.87 | 0.00 | 0.26 | 0.00 | 1.00 | 6.00 | PLAU | 3.00 | 1.00 | 0.52 | 0 | 0.00 |
| 5.31 | 0.01 | 0.19 | 0.33 | 3.00 | 12.00 | PLAUR | 9.67 | 3.00 | 0.69 | 8346 | 0.42 |
| 3.07 | 0.00 | 0.33 | 0.00 | 2.00 | 5.00 | PLCB4 | 7.00 | 2.00 | 0.65 | 16 | 0.55 |
| 1.00 | 0.00 | 1.00 | 0.00 | 1.00 | 1.00 | LIMK1 | 1.00 | 1.00 | 1.00 | 0 | 0.00 |
| 1.00 | 0.00 | 1.00 | 0.00 | 1.00 | 1.00 | PLD2 | 1.00 | 1.00 | 1.00 | 0 | 0.00 |
| 7.51 | 0.00 | 0.13 | 0.60 | 6.00 | 12.00 | POLR1C | 7.17 | 6.00 | 0.53 | 194 | 0.40 |
| 7.58 | 0.01 | 0.13 | 0.33 | 3.00 | 12.00 | POLR2H | 4.67 | 3.00 | 0.53 | 14498 | 0.39 |
| 7.56 | 0.00 | 0.13 | 0.50 | 4.00 | 12.00 | POLR3C | 5.75 | 4.00 | 0.53 | 9654 | 0.55 |
| 8.42 | 0.01 | 0.12 | 0.00 | 4.00 | 13.00 | POLR3D | 3.75 | 4.00 | 0.47 | 14602 | 0.34 |
| 7.55 | 0.00 | 0.13 | 0.50 | 4.00 | 12.00 | POLR3E | 7.00 | 4.00 | 0.53 | 5036 | 0.52 |
| 7.51 | 0.04 | 0.13 | 0.13 | 6.00 | 12.00 | POLR3H | 4.33 | 6.00 | 0.54 | 34216 | 0.25 |
| 8.67 | 0.00 | 0.12 | 0.00 | 1.00 | 13.00 | CCR6 | 2.00 | 1.00 | 0.45 | 0 | 0.00 |
| 7.67 | 0.01 | 0.13 | 0.00 | 2.00 | 12.00 | POLR3K | 3.00 | 2.00 | 0.52 | 4928 | 0.50 |
| 4.44 | 0.00 | 0.23 | 0.90 | 5.00 | 10.00 | PPP1CB | 13.60 | 5.00 | 0.75 | 64 | 0.41 |
| 3.72 | 0.07 | 0.27 | 0.27 | 18.00 | 9.00 | PPP1R12A | 11.94 | 18.00 | 0.81 | 51796 | 0.16 |
| 3.91 | 0.03 | 0.26 | 0.36 | 21.00 | 10.00 | PPP3CA | 18.38 | 21.00 | 0.79 | 43808 | 0.20 |
| 6.68 | 0.05 | 0.15 | 0.00 | 5.00 | 11.00 | PPP3CC | 3.20 | 5.00 | 0.59 | 39120 | 0.24 |
| 1.00 | 0.00 | 1.00 | 0.00 | 1.00 | 1.00 | IL3 | 1.00 | 1.00 | 1.00 | 0 | 0.00 |
| 1.00 | 0.00 | 1.00 | 0.00 | 1.00 | 1.00 | PPP3R2 | 1.00 | 1.00 | 1.00 | 0 | 0.00 |
| 4.77 | 0.00 | 0.21 | 0.17 | 4.00 | 11.00 | PREX1 | 10.75 | 4.00 | 0.73 | 3142 | 0.30 |
| 1.43 | 0.19 | 0.70 | 0.50 | 4.00 | 2.00 | PRF1 | 4.00 | 4.00 | 0.86 | 18 | 0.57 |
| 2.53 | 0.21 | 0.40 | 0.00 | 3.00 | 4.00 | PRKACA | 3.00 | 3.00 | 0.75 | 84 | 0.40 |
| 1.98 | 0.21 | 0.50 | 0.32 | 12.00 | 3.00 | PRKACG | 8.75 | 12.00 | 0.84 | 1770 | 0.21 |
| 2.96 | 0.00 | 0.34 | 0.00 | 1.00 | 4.00 | CASP12 | 12.00 | 1.00 | 0.67 | 0 | 0.00 |
| 6.00 | 0.00 | 0.17 | 0.67 | 3.00 | 10.00 | PRKCA | 5.00 | 3.00 | 0.64 | 176 | 0.50 |
| 6.06 | 0.00 | 0.16 | 0.00 | 3.00 | 13.00 | PRKCD | 3.33 | 3.00 | 0.64 | 4364 | 0.33 |
| 4.98 | 0.05 | 0.20 | 0.50 | 4.00 | 9.00 | PRKCI | 8.75 | 4.00 | 0.72 | 50964 | 0.43 |
| 6.19 | 0.00 | 0.16 | 0.50 | 4.00 | 10.00 | PRKCQ | 6.25 | 4.00 | 0.63 | 7314 | 0.39 |
| 2.38 | 0.00 | 0.42 | 0.00 | 1.00 | 4.00 | PROS1 | 5.00 | 1.00 | 0.66 | 0 | 0.00 |
| 1.00 | 1.00 | 1.00 | 0.00 | 2.00 | 1.00 | HLA-DRA | 1.00 | 2.00 | 1.00 | 2 | 0.00 |
| 1.50 | 0.00 | 0.67 | 0.00 | 1.00 | 2.00 | PSME2 | 2.00 | 1.00 | 0.75 | 0 | 0.00 |
| 1.00 | 0.00 | 1.00 | 0.00 | 1.00 | 1.00 | LSP1 | 1.00 | 1.00 | 1.00 | 0 | 0.00 |
| 1.00 | 0.00 | 1.00 | 0.00 | 1.00 | 1.00 | PSTPIP1 | 1.00 | 1.00 | 1.00 | 0 | 0.00 |
| 1.63 | 0.26 | 0.62 | 0.33 | 4.00 | 3.00 | PTGS1 | 3.75 | 4.00 | 0.84 | 28 | 0.58 |
| 2.50 | 0.00 | 0.40 | 0.00 | 1.00 | 4.00 | PF4V1 | 4.00 | 1.00 | 0.63 | 0 | 0.00 |
| 3.94 | 0.02 | 0.25 | 0.44 | 18.00 | 10.00 | PTGS2 | 20.67 | 18.00 | 0.79 | 17596 | 0.25 |
| 4.93 | 0.00 | 0.20 | 0.00 | 1.00 | 11.00 | IFNAR2 | 18.00 | 1.00 | 0.72 | 0 | 0.00 |
| 1.00 | 0.00 | 1.00 | 0.00 | 1.00 | 1.00 | HSPA5 | 1.00 | 1.00 | 1.00 | 0 | 0.00 |
| 1.00 | 0.00 | 1.00 | 0.00 | 1.00 | 1.00 | PTPN11 | 1.00 | 1.00 | 1.00 | 0 | 0.00 |
| 5.13 | 0.00 | 0.20 | 0.00 | 1.00 | 10.00 | BRAF | 4.00 | 1.00 | 0.71 | 0 | 0.00 |
| 4.13 | 0.06 | 0.24 | 0.17 | 4.00 | 9.00 | PTPRC | 6.25 | 4.00 | 0.78 | 51878 | 0.27 |
| 6.03 | 0.01 | 0.17 | 0.33 | 3.00 | 13.00 | PXN | 4.33 | 3.00 | 0.64 | 11346 | 0.44 |
| 7.02 | 0.00 | 0.14 | 0.00 | 1.00 | 14.00 | PIK3R5 | 3.00 | 1.00 | 0.57 | 0 | 0.00 |
| 4.89 | 0.00 | 0.20 | 0.00 | 1.00 | 10.00 | RAC1 | 12.00 | 1.00 | 0.72 | 0 | 0.00 |
| 1.00 | 0.00 | 1.00 | 0.00 | 1.00 | 1.00 | ACTN4 | 1.00 | 1.00 | 1.00 | 0 | 0.00 |
| 1.00 | 0.00 | 1.00 | 0.00 | 1.00 | 1.00 | RAC2 | 1.00 | 1.00 | 1.00 | 0 | 0.00 |
| 4.52 | 0.00 | 0.22 | 0.00 | 1.00 | 6.00 | C8A | 3.00 | 1.00 | 0.41 | 0 | 0.00 |
| 3.54 | 0.04 | 0.28 | 0.00 | 3.00 | 5.00 | RAPGEF4 | 2.67 | 3.00 | 0.58 | 436 | 0.33 |
| 5.32 | 0.00 | 0.19 | 1.00 | 2.00 | 12.00 | RARA | 12.00 | 2.00 | 0.69 | 0 | 0.57 |
| 8.45 | 0.00 | 0.12 | 0.33 | 3.00 | 13.00 | RASGRP2 | 8.00 | 3.00 | 0.47 | 60 | 0.64 |
| 3.90 | 0.01 | 0.26 | 0.48 | 18.00 | 10.00 | RBPJ | 21.67 | 18.00 | 0.79 | 23834 | 0.25 |
| 5.03 | 0.00 | 0.20 | 0.00 | 1.00 | 10.00 | RELB | 8.00 | 1.00 | 0.71 | 0 | 0.00 |
| 7.73 | 0.00 | 0.13 | 0.00 | 1.00 | 12.00 | RFX5 | 4.00 | 1.00 | 0.52 | 0 | 0.00 |
| 2.88 | 0.00 | 0.35 | 0.00 | 1.00 | 4.00 | RFXANK | 5.00 | 1.00 | 0.69 | 0 | 0.00 |
| 5.85 | 0.04 | 0.17 | 0.00 | 3.00 | 10.00 | RHOH | 4.00 | 3.00 | 0.65 | 13426 | 0.33 |
| 6.85 | 0.00 | 0.15 | 0.00 | 1.00 | 11.00 | IL7R | 3.00 | 1.00 | 0.58 | 0 | 0.00 |
| 4.49 | 0.00 | 0.22 | 0.67 | 7.00 | 11.00 | RNASEL | 23.14 | 7.00 | 0.75 | 5690 | 0.38 |
| 4.14 | 0.01 | 0.24 | 0.67 | 6.00 | 9.00 | ROCK2 | 10.83 | 6.00 | 0.78 | 6698 | 0.35 |
| 6.14 | 0.09 | 0.16 | 0.49 | 10.00 | 10.00 | RORA | 6.80 | 10.00 | 0.63 | 97570 | 0.34 |
| 3.92 | 0.28 | 0.26 | 0.38 | 12.00 | 7.00 | RPS6KB1 | 8.58 | 12.00 | 0.79 | 270898 | 0.26 |
| 1.50 | 0.00 | 0.67 | 0.00 | 1.00 | 2.00 | RXRA | 2.00 | 1.00 | 0.75 | 0 | 0.00 |
| 2.56 | 0.03 | 0.39 | 0.38 | 7.00 | 5.00 | SCIN | 8.43 | 7.00 | 0.74 | 328 | 0.31 |
| 2.46 | 0.06 | 0.41 | 0.38 | 7.00 | 5.00 | SERPINE1 | 8.86 | 7.00 | 0.76 | 570 | 0.31 |
| 4.06 | 0.00 | 0.25 | 0.00 | 1.00 | 6.00 | SH3BP2 | 3.00 | 1.00 | 0.49 | 0 | 0.00 |
| 6.62 | 0.00 | 0.15 | 0.00 | 1.00 | 10.00 | SIKE1 | 3.00 | 1.00 | 0.60 | 0 | 0.00 |
| 3.12 | 0.13 | 0.32 | 0.00 | 3.00 | 5.00 | SIPA1 | 2.33 | 3.00 | 0.65 | 64 | 0.44 |
| 6.60 | 0.12 | 0.15 | 0.39 | 8.00 | 11.00 | SMAD3 | 6.25 | 8.00 | 0.60 | 125548 | 0.29 |
| 4.40 | 0.00 | 0.23 | 0.52 | 7.00 | 10.00 | SNAP23 | 17.29 | 7.00 | 0.76 | 2362 | 0.35 |
| 4.57 | 0.01 | 0.22 | 0.46 | 8.00 | 8.00 | SOS1 | 8.38 | 8.00 | 0.75 | 7556 | 0.36 |
| 4.28 | 0.02 | 0.23 | 0.38 | 24.00 | 11.00 | SOS2 | 19.00 | 24.00 | 0.77 | 40258 | 0.22 |
| 3.35 | 0.00 | 0.30 | 0.00 | 1.00 | 5.00 | BAD | 3.00 | 1.00 | 0.61 | 0 | 0.00 |
| 2.41 | 0.40 | 0.41 | 0.00 | 3.00 | 4.00 | SPHK2 | 3.00 | 3.00 | 0.76 | 126 | 0.33 |
| 6.63 | 0.00 | 0.15 | 0.00 | 1.00 | 13.00 | STAT1 | 4.00 | 1.00 | 0.60 | 0 | 0.00 |
| 4.25 | 0.01 | 0.24 | 0.58 | 9.00 | 10.00 | STAT3 | 19.89 | 9.00 | 0.77 | 6458 | 0.31 |
| 7.08 | 0.02 | 0.14 | 0.50 | 8.00 | 11.00 | STAT4 | 6.50 | 8.00 | 0.57 | 23786 | 0.49 |
| 8.08 | 0.00 | 0.12 | 0.00 | 1.00 | 12.00 | CD81 | 8.00 | 1.00 | 0.49 | 0 | 0.00 |
| 5.42 | 0.00 | 0.18 | 0.00 | 2.00 | 12.00 | STAT5B | 8.50 | 2.00 | 0.68 | 16 | 0.63 |
| 5.57 | 0.00 | 0.18 | 0.00 | 2.00 | 11.00 | IFNK | 2.00 | 2.00 | 0.67 | 454 | 0.50 |
| 5.86 | 0.00 | 0.17 | 0.00 | 2.00 | 12.00 | STAT6 | 3.50 | 2.00 | 0.65 | 188 | 0.50 |
| 4.78 | 0.01 | 0.21 | 0.17 | 4.00 | 11.00 | SYK | 8.25 | 4.00 | 0.73 | 5002 | 0.32 |
| 3.61 | 0.13 | 0.28 | 0.25 | 21.00 | 9.00 | TAB2 | 13.81 | 21.00 | 0.81 | 152514 | 0.15 |
| 4.61 | 0.00 | 0.22 | 0.00 | 1.00 | 10.00 | TAB3 | 21.00 | 1.00 | 0.74 | 0 | 0.00 |
| 3.63 | 0.11 | 0.28 | 0.22 | 14.00 | 9.00 | TANK | 14.29 | 14.00 | 0.81 | 119882 | 0.17 |
| 3.92 | 0.05 | 0.25 | 0.38 | 11.00 | 9.00 | TBK1 | 11.18 | 11.00 | 0.79 | 48556 | 0.23 |
| 4.92 | 0.00 | 0.20 | 0.00 | 1.00 | 10.00 | PIK3CA | 11.00 | 1.00 | 0.72 | 0 | 0.00 |
| 4.06 | 0.00 | 0.25 | 0.00 | 1.00 | 6.00 | TBKBP1 | 3.00 | 1.00 | 0.49 | 0 | 0.00 |
| 1.29 | 0.12 | 0.78 | 0.70 | 5.00 | 2.00 | TBX21 | 4.40 | 5.00 | 0.90 | 12 | 0.63 |
| 4.18 | 0.02 | 0.24 | 0.40 | 28.00 | 11.00 | TBXAS1 | 19.89 | 28.00 | 0.77 | 35450 | 0.22 |
| 7.68 | 0.00 | 0.13 | 0.00 | 1.00 | 12.00 | TFRC | 5.00 | 1.00 | 0.52 | 0 | 0.00 |
| 4.53 | 0.00 | 0.22 | 1.00 | 2.00 | 10.00 | TGFBR1 | 19.50 | 2.00 | 0.75 | 0 | 0.72 |
| 5.27 | 0.00 | 0.19 | 1.00 | 2.00 | 12.00 | THBD | 13.00 | 2.00 | 0.69 | 0 | 0.57 |
| 1.33 | 0.00 | 0.75 | 1.00 | 2.00 | 2.00 | MYLK3 | 2.50 | 2.00 | 0.83 | 0 | 0.83 |
| 1.33 | 0.00 | 0.75 | 1.00 | 2.00 | 2.00 | THY1 | 2.50 | 2.00 | 0.83 | 0 | 0.83 |
| 3.66 | 0.08 | 0.27 | 0.23 | 13.00 | 9.00 | TICAM2 | 12.38 | 13.00 | 0.81 | 60084 | 0.15 |
| 3.33 | 0.00 | 0.30 | 0.00 | 1.00 | 5.00 | TLN2 | 11.00 | 1.00 | 0.61 | 0 | 0.00 |
| 4.17 | 0.02 | 0.24 | 0.39 | 32.00 | 11.00 | TLR1 | 19.28 | 32.00 | 0.77 | 25516 | 0.21 |
| 5.69 | 0.00 | 0.18 | 0.00 | 1.00 | 12.00 | AIM2 | 6.00 | 1.00 | 0.67 | 0 | 0.00 |
| 4.69 | 0.01 | 0.21 | 0.47 | 6.00 | 11.00 | TLR2 | 13.50 | 6.00 | 0.74 | 2106 | 0.32 |
| 4.48 | 0.00 | 0.22 | 0.52 | 7.00 | 11.00 | TLR4 | 23.43 | 7.00 | 0.75 | 3084 | 0.36 |
| 5.19 | 0.00 | 0.19 | 1.00 | 3.00 | 12.00 | TLR5 | 18.33 | 3.00 | 0.70 | 0 | 0.57 |
| 5.25 | 0.00 | 0.19 | 1.00 | 2.00 | 12.00 | TLR6 | 15.50 | 2.00 | 0.70 | 0 | 0.74 |
| 4.17 | 0.03 | 0.24 | 0.36 | 27.00 | 11.00 | TLR8 | 18.37 | 27.00 | 0.77 | 34492 | 0.19 |
| 5.95 | 0.02 | 0.17 | 0.17 | 4.00 | 10.00 | TNFRSF10A | 3.75 | 4.00 | 0.65 | 9830 | 0.30 |
| 6.95 | 0.00 | 0.14 | 0.00 | 1.00 | 11.00 | ICOS | 4.00 | 1.00 | 0.58 | 0 | 0.00 |
| 6.95 | 0.00 | 0.14 | 0.00 | 1.00 | 11.00 | NFATC1 | 4.00 | 1.00 | 0.58 | 0 | 0.00 |
| 4.65 | 0.00 | 0.21 | 0.00 | 1.00 | 10.00 | TNFRSF10B | 13.00 | 1.00 | 0.74 | 0 | 0.00 |
| 4.66 | 0.04 | 0.21 | 0.19 | 7.00 | 11.00 | TNFSF10 | 6.00 | 7.00 | 0.74 | 23356 | 0.21 |
| 4.87 | 0.00 | 0.21 | 0.00 | 2.00 | 11.00 | TNFSF13B | 9.00 | 2.00 | 0.72 | 932 | 0.50 |
| 8.44 | 0.00 | 0.12 | 1.00 | 3.00 | 13.00 | TP53BP1 | 8.00 | 3.00 | 0.47 | 0 | 0.73 |
| 8.48 | 0.00 | 0.12 | 1.00 | 3.00 | 13.00 | TRADD | 8.00 | 3.00 | 0.47 | 0 | 0.73 |
| 4.63 | 0.01 | 0.22 | 0.00 | 2.00 | 10.00 | TRAF6 | 7.50 | 2.00 | 0.74 | 1726 | 0.50 |
| 4.31 | 0.01 | 0.23 | 0.53 | 19.00 | 11.00 | TRIM25 | 21.58 | 19.00 | 0.76 | 6356 | 0.26 |
| 2.65 | 0.11 | 0.38 | 0.14 | 8.00 | 4.00 | TRIP6 | 4.88 | 8.00 | 0.73 | 870 | 0.25 |
| 3.63 | 0.00 | 0.28 | 0.00 | 1.00 | 5.00 | IFNA2 | 8.00 | 1.00 | 0.56 | 0 | 0.00 |
| 3.63 | 0.00 | 0.28 | 0.00 | 1.00 | 5.00 | TFPI | 8.00 | 1.00 | 0.56 | 0 | 0.00 |
| 9.42 | 0.00 | 0.11 | 1.00 | 2.00 | 14.00 | IRF3 | 3.00 | 2.00 | 0.40 | 0 | 0.75 |
| 9.34 | 0.00 | 0.11 | 0.33 | 3.00 | 14.00 | TRPV2 | 3.00 | 3.00 | 0.40 | 94 | 0.44 |
| 9.47 | 0.00 | 0.11 | 0.00 | 1.00 | 14.00 | TXK | 4.00 | 1.00 | 0.40 | 0 | 0.00 |
| 5.32 | 0.00 | 0.19 | 0.00 | 1.00 | 12.00 | TXN | 20.00 | 1.00 | 0.69 | 0 | 0.00 |
| 1.00 | 0.00 | 1.00 | 0.00 | 1.00 | 1.00 | CYBA | 1.00 | 1.00 | 1.00 | 0 | 0.00 |
| 1.00 | 0.00 | 1.00 | 0.00 | 1.00 | 1.00 | TXN2 | 1.00 | 1.00 | 1.00 | 0 | 0.00 |
| 3.47 | 0.00 | 0.29 | 0.00 | 1.00 | 5.00 | TYK2 | 3.00 | 1.00 | 0.59 | 0 | 0.00 |
| 1.67 | 0.00 | 0.60 | 0.00 | 1.00 | 2.00 | IL5 | 3.00 | 1.00 | 0.67 | 0 | 0.00 |
| 1.00 | 0.67 | 1.00 | 0.33 | 3.00 | 1.00 | ULBP2 | 1.67 | 3.00 | 1.00 | 4 | 0.67 |
| 1.50 | 0.00 | 0.67 | 0.00 | 1.00 | 2.00 | VAMP8 | 2.00 | 1.00 | 0.75 | 0 | 0.00 |
| 5.07 | 0.02 | 0.20 | 0.24 | 7.00 | 12.00 | VASP | 10.43 | 7.00 | 0.71 | 41400 | 0.26 |
| 4.39 | 0.02 | 0.23 | 0.30 | 5.00 | 10.00 | VAV3 | 12.00 | 5.00 | 0.76 | 3486 | 0.31 |
| 2.00 | 0.00 | 0.50 | 0.00 | 1.00 | 3.00 | VDAC3 | 2.00 | 1.00 | 0.67 | 0 | 0.00 |
| 1.00 | 0.00 | 1.00 | 0.00 | 1.00 | 1.00 | BCL2L1 | 1.00 | 1.00 | 1.00 | 0 | 0.00 |
| 1.00 | 0.00 | 1.00 | 0.00 | 1.00 | 1.00 | WASF2 | 1.00 | 1.00 | 1.00 | 0 | 0.00 |
| 8.47 | 0.01 | 0.12 | 0.33 | 3.00 | 13.00 | ZAP70 | 3.67 | 3.00 | 0.47 | 9716 | 0.42 |

**Supplementary Table S7: Topological analysis from GSE54514 sepsis group**

| **Average Shortest Path Length** | **Between -ness Centrality** | **Closeness Centrality** | **Clustering Coefficient** | **Degree** | **Eccen -tricity** | **name** | **Neighbor- hood Connectivity** | **Number Of Directed Edges** | **Radiality** | **Stress** | **Topological Coefficient** |
| --- | --- | --- | --- | --- | --- | --- | --- | --- | --- | --- | --- |
| 3.11 | 0.00 | 0.32 | 1.00 | 3.00 | 5.00 | ACTN1 | 7.33 | 3.00 | 0.74 | 0 | 0.52 |
| 2.45 | 0.17 | 0.41 | 0.25 | 9.00 | 5.00 | ACTN4 | 5.33 | 9.00 | 0.82 | 870 | 0.22 |
| 4.59 | 0.00 | 0.22 | 0.67 | 4.00 | 9.00 | ADAR | 7.50 | 4.00 | 0.67 | 18 | 0.54 |
| 4.63 | 0.00 | 0.22 | 0.67 | 3.00 | 9.00 | AIM2 | 8.33 | 3.00 | 0.67 | 10 | 0.60 |
| 3.17 | 0.00 | 0.32 | 1.00 | 3.00 | 6.00 | AKT2 | 6.33 | 3.00 | 0.73 | 0 | 0.53 |
| 1.00 | 0.00 | 1.00 | 0.00 | 1.00 | 1.00 | ADCY5 | 1.00 | 1.00 | 1.00 | 0 | 0.00 |
| 1.00 | 0.00 | 1.00 | 0.00 | 1.00 | 1.00 | ANPEP | 1.00 | 1.00 | 1.00 | 0 | 0.00 |
| 3.47 | 0.06 | 0.29 | 0.40 | 6.00 | 7.00 | ACTB | 4.17 | 6.00 | 0.69 | 302 | 0.35 |
| 3.13 | 0.09 | 0.32 | 0.30 | 5.00 | 7.00 | ARPC4 | 5.40 | 5.00 | 0.73 | 570 | 0.36 |
| 2.53 | 0.21 | 0.39 | 0.19 | 7.00 | 5.00 | BCL3 | 5.43 | 7.00 | 0.81 | 956 | 0.21 |
| 1.00 | 0.00 | 1.00 | 0.00 | 1.00 | 1.00 | C1QA | 1.00 | 1.00 | 1.00 | 0 | 0.00 |
| 1.00 | 0.00 | 1.00 | 0.00 | 1.00 | 1.00 | C1QB | 1.00 | 1.00 | 1.00 | 0 | 0.00 |
| 1.00 | 0.00 | 1.00 | 0.00 | 1.00 | 1.00 | C4A | 1.00 | 1.00 | 1.00 | 0 | 0.00 |
| 1.00 | 0.00 | 1.00 | 0.00 | 1.00 | 1.00 | C4B | 1.00 | 1.00 | 1.00 | 0 | 0.00 |
| 3.89 | 0.01 | 0.26 | 0.60 | 5.00 | 8.00 | CALM1 | 4.40 | 5.00 | 0.64 | 98 | 0.49 |
| 3.89 | 0.01 | 0.26 | 0.60 | 5.00 | 8.00 | CALM2 | 4.40 | 5.00 | 0.64 | 98 | 0.49 |
| 4.38 | 0.00 | 0.23 | 1.00 | 3.00 | 8.00 | CALM3 | 5.33 | 3.00 | 0.58 | 0 | 0.76 |
| 3.39 | 0.03 | 0.29 | 0.61 | 8.00 | 8.00 | B2M | 10.13 | 8.00 | 0.78 | 1144 | 0.36 |
| 3.39 | 0.11 | 0.29 | 0.36 | 9.00 | 8.00 | CARD16 | 7.89 | 9.00 | 0.78 | 1864 | 0.29 |
| 3.01 | 0.23 | 0.33 | 0.37 | 16.00 | 7.00 | CASP1 | 8.75 | 16.00 | 0.82 | 5994 | 0.25 |
| 1.00 | 0.00 | 1.00 | 0.00 | 1.00 | 1.00 | CCL14 | 1.00 | 1.00 | 1.00 | 0 | 0.00 |
| 1.00 | 0.00 | 1.00 | 0.00 | 1.00 | 1.00 | CCL15 | 1.00 | 1.00 | 1.00 | 0 | 0.00 |
| 1.00 | 0.00 | 1.00 | 1.00 | 2.00 | 1.00 | CCL3 | 2.00 | 2.00 | 1.00 | 0 | 1.00 |
| 1.00 | 0.00 | 1.00 | 1.00 | 2.00 | 1.00 | CCL3L1 | 2.00 | 2.00 | 1.00 | 0 | 1.00 |
| 1.00 | 0.00 | 1.00 | 1.00 | 2.00 | 1.00 | CCL3L3 | 2.00 | 2.00 | 1.00 | 0 | 1.00 |
| 1.00 | 0.00 | 1.00 | 0.00 | 1.00 | 1.00 | CCR7 | 1.00 | 1.00 | 1.00 | 0 | 0.00 |
| 1.00 | 0.00 | 1.00 | 0.00 | 1.00 | 1.00 | CD19 | 1.00 | 1.00 | 1.00 | 0 | 0.00 |
| 1.00 | 0.00 | 1.00 | 0.00 | 1.00 | 1.00 | C7 | 1.00 | 1.00 | 1.00 | 0 | 0.00 |
| 1.00 | 0.00 | 1.00 | 0.00 | 1.00 | 1.00 | CD1E | 1.00 | 1.00 | 1.00 | 0 | 0.00 |
| 5.80 | 0.02 | 0.17 | 0.60 | 6.00 | 11.00 | CD2 | 7.00 | 6.00 | 0.68 | 1690 | 0.47 |
| 5.78 | 0.00 | 0.17 | 0.60 | 6.00 | 11.00 | CD3G | 7.50 | 6.00 | 0.68 | 160 | 0.47 |
| 5.06 | 0.11 | 0.20 | 0.38 | 10.00 | 10.00 | CD247 | 6.20 | 10.00 | 0.73 | 6530 | 0.32 |
| 1.00 | 0.00 | 1.00 | 0.00 | 1.00 | 1.00 | BIN1 | 1.00 | 1.00 | 1.00 | 0 | 0.00 |
| 1.00 | 0.00 | 1.00 | 0.00 | 1.00 | 1.00 | CD79A | 1.00 | 1.00 | 1.00 | 0 | 0.00 |
| 6.68 | 0.02 | 0.15 | 0.50 | 4.00 | 12.00 | CD8A | 5.75 | 4.00 | 0.62 | 2342 | 0.61 |
| 7.67 | 0.00 | 0.13 | 0.00 | 1.00 | 13.00 | CD8B | 4.00 | 1.00 | 0.56 | 0 | 0.00 |
| 4.25 | 0.24 | 0.24 | 0.67 | 4.00 | 9.00 | CCL5 | 7.75 | 4.00 | 0.78 | 12150 | 0.34 |
| 4.25 | 0.24 | 0.24 | 0.67 | 4.00 | 9.00 | CD99 | 7.75 | 4.00 | 0.78 | 12150 | 0.34 |
| 4.65 | 0.00 | 0.22 | 0.00 | 1.00 | 7.00 | CARD6 | 5.00 | 1.00 | 0.67 | 0 | 0.00 |
| 3.66 | 0.10 | 0.27 | 0.00 | 5.00 | 6.00 | CHUK | 3.40 | 5.00 | 0.76 | 1516 | 0.22 |
| 1.00 | 0.00 | 1.00 | 0.00 | 1.00 | 1.00 | ASAP3 | 1.00 | 1.00 | 1.00 | 0 | 0.00 |
| 1.00 | 0.00 | 1.00 | 0.00 | 1.00 | 1.00 | CLDN1 | 1.00 | 1.00 | 1.00 | 0 | 0.00 |
| 6.45 | 0.05 | 0.15 | 0.33 | 4.00 | 13.00 | BCL2L1 | 3.75 | 4.00 | 0.64 | 892 | 0.35 |
| 7.42 | 0.02 | 0.13 | 0.00 | 2.00 | 14.00 | CLDN5 | 2.50 | 2.00 | 0.57 | 440 | 0.50 |
| 1.00 | 0.00 | 1.00 | 0.00 | 1.00 | 1.00 | CD44 | 1.00 | 1.00 | 1.00 | 0 | 0.00 |
| 1.00 | 0.00 | 1.00 | 0.00 | 1.00 | 1.00 | CR1 | 1.00 | 1.00 | 1.00 | 0 | 0.00 |
| 4.34 | 0.02 | 0.23 | 0.33 | 3.00 | 9.00 | CREB1 | 4.33 | 3.00 | 0.70 | 344 | 0.44 |
| 5.33 | 0.00 | 0.19 | 0.00 | 1.00 | 10.00 | CD46 | 3.00 | 1.00 | 0.61 | 0 | 0.00 |
| 3.83 | 0.03 | 0.26 | 0.00 | 2.00 | 8.00 | CARD8 | 5.50 | 2.00 | 0.74 | 468 | 0.50 |
| 4.68 | 0.02 | 0.21 | 0.00 | 2.00 | 9.00 | CRK | 2.00 | 2.00 | 0.67 | 214 | 0.50 |
| 1.00 | 0.00 | 1.00 | 0.00 | 1.00 | 1.00 | CD86 | 1.00 | 1.00 | 1.00 | 0 | 0.00 |
| 1.00 | 0.00 | 1.00 | 0.00 | 1.00 | 1.00 | CSF1R | 1.00 | 1.00 | 1.00 | 0 | 0.00 |
| 4.37 | 0.00 | 0.23 | 1.00 | 2.00 | 9.00 | CTSS | 6.00 | 2.00 | 0.69 | 0 | 0.67 |
| 1.00 | 0.00 | 1.00 | 0.00 | 1.00 | 1.00 | CXCR1 | 1.00 | 1.00 | 1.00 | 0 | 0.00 |
| 1.00 | 0.00 | 1.00 | 0.00 | 1.00 | 1.00 | CXCR2 | 1.00 | 1.00 | 1.00 | 0 | 0.00 |
| 3.22 | 0.06 | 0.31 | 0.53 | 6.00 | 7.00 | CYLD | 8.83 | 6.00 | 0.80 | 2104 | 0.35 |
| 3.73 | 0.03 | 0.27 | 0.45 | 11.00 | 8.00 | DDX58 | 8.82 | 11.00 | 0.75 | 1074 | 0.34 |
| 1.00 | 0.00 | 1.00 | 1.00 | 3.00 | 1.00 | DEFA1 | 3.00 | 3.00 | 1.00 | 0 | 1.00 |
| 1.00 | 0.00 | 1.00 | 1.00 | 3.00 | 1.00 | DEFA1B | 3.00 | 3.00 | 1.00 | 0 | 1.00 |
| 1.00 | 0.00 | 1.00 | 1.00 | 3.00 | 1.00 | DEFA3 | 3.00 | 3.00 | 1.00 | 0 | 1.00 |
| 1.00 | 0.00 | 1.00 | 1.00 | 3.00 | 1.00 | DEFA4 | 3.00 | 3.00 | 1.00 | 0 | 1.00 |
| 1.00 | 0.00 | 1.00 | 0.00 | 1.00 | 1.00 | DEFB103A | 1.00 | 1.00 | 1.00 | 0 | 0.00 |
| 1.00 | 0.00 | 1.00 | 0.00 | 1.00 | 1.00 | DEFB103B | 1.00 | 1.00 | 1.00 | 0 | 0.00 |
| 1.00 | 0.00 | 1.00 | 0.00 | 1.00 | 1.00 | DEFB4A | 1.00 | 1.00 | 1.00 | 0 | 0.00 |
| 1.00 | 0.00 | 1.00 | 0.00 | 1.00 | 1.00 | DEFB4B | 1.00 | 1.00 | 1.00 | 0 | 0.00 |
| 1.00 | 0.00 | 1.00 | 0.00 | 1.00 | 1.00 | CXCR3 | 1.00 | 1.00 | 1.00 | 0 | 0.00 |
| 1.00 | 0.00 | 1.00 | 0.00 | 1.00 | 1.00 | DLL3 | 1.00 | 1.00 | 1.00 | 0 | 0.00 |
| 3.00 | 0.17 | 0.33 | 0.07 | 6.00 | 6.00 | BAD | 3.50 | 6.00 | 0.75 | 722 | 0.20 |
| 2.74 | 0.23 | 0.36 | 0.07 | 6.00 | 5.00 | DNM2 | 4.33 | 6.00 | 0.78 | 960 | 0.20 |
| 8.41 | 0.00 | 0.12 | 0.00 | 1.00 | 15.00 | EPOR | 2.00 | 1.00 | 0.51 | 0 | 0.00 |
| 5.76 | 0.00 | 0.17 | 0.91 | 12.00 | 12.00 | CLEC1B | 17.92 | 12.00 | 0.68 | 12 | 0.81 |
| 4.92 | 0.01 | 0.20 | 0.77 | 21.00 | 11.00 | ESAM | 16.71 | 21.00 | 0.74 | 1460 | 0.60 |
| 5.64 | 0.02 | 0.18 | 0.69 | 9.00 | 12.00 | CXCL5 | 16.33 | 9.00 | 0.69 | 2696 | 0.65 |
| 5.11 | 0.02 | 0.20 | 0.76 | 15.00 | 11.00 | F13A1 | 16.87 | 15.00 | 0.73 | 1392 | 0.62 |
| 3.22 | 0.11 | 0.31 | 0.44 | 9.00 | 8.00 | FAS | 9.56 | 9.00 | 0.80 | 3116 | 0.28 |
| 4.73 | 0.02 | 0.21 | 0.00 | 2.00 | 7.00 | CDC42 | 2.50 | 2.00 | 0.66 | 462 | 0.50 |
| 5.72 | 0.00 | 0.17 | 0.00 | 1.00 | 8.00 | FCER1G | 2.00 | 1.00 | 0.57 | 0 | 0.00 |
| 3.74 | 0.02 | 0.27 | 0.53 | 10.00 | 8.00 | FCGR1A | 9.40 | 10.00 | 0.75 | 702 | 0.36 |
| 4.21 | 0.00 | 0.24 | 0.80 | 5.00 | 8.00 | DAPP1 | 10.20 | 5.00 | 0.71 | 22 | 0.51 |
| 1.00 | 0.00 | 1.00 | 0.00 | 1.00 | 1.00 | FCGR3A | 1.00 | 1.00 | 1.00 | 0 | 0.00 |
| 1.00 | 0.00 | 1.00 | 0.00 | 1.00 | 1.00 | FCGR3B | 1.00 | 1.00 | 1.00 | 0 | 0.00 |
| 4.65 | 0.00 | 0.22 | 0.00 | 1.00 | 7.00 | FGR | 5.00 | 1.00 | 0.67 | 0 | 0.00 |
| 6.82 | 0.00 | 0.15 | 0.00 | 1.00 | 12.00 | CCL28 | 5.00 | 1.00 | 0.61 | 0 | 0.00 |
| 5.83 | 0.02 | 0.17 | 0.40 | 5.00 | 11.00 | GATA3 | 5.80 | 5.00 | 0.68 | 874 | 0.43 |
| 3.39 | 0.13 | 0.29 | 0.30 | 15.00 | 7.00 | GBP1 | 7.67 | 15.00 | 0.78 | 2940 | 0.28 |
| 3.68 | 0.03 | 0.27 | 0.49 | 11.00 | 8.00 | GBP2 | 9.27 | 11.00 | 0.76 | 1178 | 0.32 |
| 4.38 | 0.00 | 0.23 | 0.00 | 1.00 | 8.00 | GBP3 | 15.00 | 1.00 | 0.69 | 0 | 0.00 |
| 4.26 | 0.00 | 0.23 | 0.60 | 5.00 | 8.00 | GBP4 | 8.60 | 5.00 | 0.70 | 86 | 0.45 |
| 3.70 | 0.02 | 0.27 | 0.53 | 11.00 | 8.00 | GBP5 | 9.82 | 11.00 | 0.75 | 764 | 0.35 |
| 3.02 | 0.12 | 0.33 | 0.10 | 5.00 | 5.00 | GNAI2 | 3.40 | 5.00 | 0.75 | 396 | 0.25 |
| 5.62 | 0.00 | 0.18 | 1.00 | 2.00 | 8.00 | CLEC4D | 3.00 | 2.00 | 0.58 | 0 | 0.75 |
| 5.62 | 0.00 | 0.18 | 1.00 | 2.00 | 8.00 | GNAI3 | 3.00 | 2.00 | 0.58 | 0 | 0.75 |
| 2.91 | 0.13 | 0.34 | 0.17 | 4.00 | 6.00 | GNB2 | 4.75 | 4.00 | 0.76 | 484 | 0.28 |
| 3.43 | 0.14 | 0.29 | 0.33 | 4.00 | 6.00 | GNB4 | 10.00 | 4.00 | 0.78 | 2576 | 0.41 |
| 4.94 | 0.00 | 0.20 | 0.81 | 20.00 | 11.00 | GNB5 | 17.15 | 20.00 | 0.74 | 800 | 0.64 |
| 5.01 | 0.00 | 0.20 | 0.90 | 15.00 | 11.00 | GNG11 | 18.13 | 15.00 | 0.73 | 394 | 0.70 |
| 1.50 | 0.00 | 0.67 | 0.00 | 1.00 | 2.00 | CCL11 | 2.00 | 1.00 | 0.75 | 0 | 0.00 |
| 1.00 | 1.00 | 1.00 | 0.00 | 2.00 | 1.00 | GNG3 | 1.00 | 2.00 | 1.00 | 2 | 0.00 |
| 5.92 | 0.00 | 0.17 | 0.00 | 1.00 | 12.00 | CCR4 | 20.00 | 1.00 | 0.67 | 0 | 0.00 |
| 4.93 | 0.03 | 0.20 | 0.74 | 20.00 | 11.00 | GP1BA | 16.45 | 20.00 | 0.74 | 1478 | 0.61 |
| 4.83 | 0.20 | 0.21 | 0.75 | 19.00 | 11.00 | GP1BB | 16.84 | 19.00 | 0.74 | 5572 | 0.52 |
| 5.59 | 0.00 | 0.18 | 0.95 | 14.00 | 12.00 | GP6 | 18.43 | 14.00 | 0.69 | 26 | 0.74 |
| 4.99 | 0.01 | 0.20 | 0.89 | 15.00 | 11.00 | GRAP2 | 17.93 | 15.00 | 0.73 | 966 | 0.64 |
| 5.49 | 0.00 | 0.18 | 0.00 | 1.00 | 8.00 | CD37 | 2.00 | 1.00 | 0.44 | 0 | 0.00 |
| 4.51 | 0.04 | 0.22 | 0.00 | 2.00 | 7.00 | GRB2 | 1.50 | 2.00 | 0.56 | 136 | 0.50 |
| 3.57 | 0.08 | 0.28 | 0.00 | 2.00 | 6.00 | GNB1 | 3.50 | 2.00 | 0.68 | 268 | 0.50 |
| 3.83 | 0.04 | 0.26 | 0.00 | 2.00 | 6.00 | F10 | 3.50 | 2.00 | 0.65 | 192 | 0.50 |
| 4.81 | 0.00 | 0.21 | 0.00 | 1.00 | 7.00 | GSK3A | 2.00 | 1.00 | 0.52 | 0 | 0.00 |
| 3.70 | 0.04 | 0.27 | 0.33 | 3.00 | 7.00 | GSN | 3.67 | 3.00 | 0.66 | 130 | 0.42 |
| 1.50 | 0.00 | 0.67 | 0.00 | 1.00 | 2.00 | CD74 | 2.00 | 1.00 | 0.75 | 0 | 0.00 |
| 1.00 | 1.00 | 1.00 | 0.00 | 2.00 | 1.00 | HLA-DMA | 1.00 | 2.00 | 1.00 | 2 | 0.00 |
| 1.50 | 0.00 | 0.67 | 0.00 | 1.00 | 2.00 | HLA-DMB | 2.00 | 1.00 | 0.75 | 0 | 0.00 |
| 1.75 | 0.50 | 0.57 | 0.00 | 2.00 | 3.00 | CD79B | 1.50 | 2.00 | 0.81 | 6 | 0.50 |
| 2.50 | 0.00 | 0.40 | 0.00 | 1.00 | 4.00 | HLA-DOA | 2.00 | 1.00 | 0.63 | 0 | 0.00 |
| 1.00 | 0.00 | 1.00 | 0.00 | 1.00 | 1.00 | HLA-DPA1 | 1.00 | 1.00 | 1.00 | 0 | 0.00 |
| 1.00 | 0.00 | 1.00 | 0.00 | 1.00 | 1.00 | HLA-DPB1 | 1.00 | 1.00 | 1.00 | 0 | 0.00 |
| 1.00 | 0.00 | 1.00 | 1.00 | 2.00 | 1.00 | HLA-DRB1 | 2.00 | 2.00 | 1.00 | 0 | 1.00 |
| 1.00 | 0.00 | 1.00 | 1.00 | 2.00 | 1.00 | HLA-DRB3 | 2.00 | 2.00 | 1.00 | 0 | 1.00 |
| 1.00 | 0.00 | 1.00 | 1.00 | 2.00 | 1.00 | HLA-DRB5 | 2.00 | 2.00 | 1.00 | 0 | 1.00 |
| 1.50 | 0.00 | 0.67 | 0.00 | 1.00 | 2.00 | HLA-B | 2.00 | 1.00 | 0.75 | 0 | 0.00 |
| 1.00 | 1.00 | 1.00 | 0.00 | 2.00 | 1.00 | HLA-G | 1.00 | 2.00 | 1.00 | 2 | 0.00 |
| 1.50 | 0.00 | 0.67 | 0.00 | 1.00 | 2.00 | HLA-E | 2.00 | 1.00 | 0.75 | 0 | 0.00 |
| 1.50 | 0.00 | 0.67 | 0.00 | 1.00 | 2.00 | CD7 | 2.00 | 1.00 | 0.75 | 0 | 0.00 |
| 1.00 | 1.00 | 1.00 | 0.00 | 2.00 | 1.00 | HRAS | 1.00 | 2.00 | 1.00 | 2 | 0.00 |
| 1.50 | 0.00 | 0.67 | 0.00 | 1.00 | 2.00 | GRK1 | 2.00 | 1.00 | 0.75 | 0 | 0.00 |
| 1.00 | 0.00 | 1.00 | 0.00 | 1.00 | 1.00 | HSP90AA1 | 1.00 | 1.00 | 1.00 | 0 | 0.00 |
| 1.00 | 0.00 | 1.00 | 0.00 | 1.00 | 1.00 | HSP90AB1 | 1.00 | 1.00 | 1.00 | 0 | 0.00 |
| 1.00 | 0.00 | 1.00 | 0.00 | 1.00 | 1.00 | HSPA1A | 1.00 | 1.00 | 1.00 | 0 | 0.00 |
| 1.00 | 0.00 | 1.00 | 0.00 | 1.00 | 1.00 | HSPA1B | 1.00 | 1.00 | 1.00 | 0 | 0.00 |
| 4.68 | 0.00 | 0.21 | 1.00 | 3.00 | 9.00 | ICAM2 | 4.00 | 3.00 | 0.75 | 0 | 0.80 |
| 3.72 | 0.02 | 0.27 | 0.58 | 10.00 | 8.00 | IFI16 | 9.90 | 10.00 | 0.75 | 870 | 0.35 |
| 4.22 | 0.00 | 0.24 | 0.50 | 4.00 | 8.00 | IFIH1 | 9.75 | 4.00 | 0.71 | 98 | 0.42 |
| 4.59 | 0.00 | 0.22 | 1.00 | 3.00 | 9.00 | IFITM1 | 10.33 | 3.00 | 0.67 | 0 | 0.74 |
| 3.65 | 0.25 | 0.27 | 0.00 | 4.00 | 8.00 | CASP5 | 4.50 | 4.00 | 0.76 | 3130 | 0.25 |
| 4.63 | 0.00 | 0.22 | 0.00 | 1.00 | 9.00 | IFNAR1 | 4.00 | 1.00 | 0.67 | 0 | 0.00 |
| 1.33 | 0.00 | 0.75 | 1.00 | 2.00 | 2.00 | CLDN16 | 2.50 | 2.00 | 0.83 | 0 | 0.83 |
| 1.33 | 0.00 | 0.75 | 1.00 | 2.00 | 2.00 | IFNE | 2.50 | 2.00 | 0.83 | 0 | 0.83 |
| 4.65 | 0.06 | 0.22 | 0.17 | 4.00 | 7.00 | IFNGR1 | 2.50 | 4.00 | 0.67 | 1022 | 0.33 |
| 3.06 | 0.05 | 0.33 | 0.30 | 5.00 | 5.00 | ARPC1B | 4.60 | 5.00 | 0.74 | 270 | 0.31 |
| 3.28 | 0.00 | 0.31 | 0.67 | 3.00 | 5.00 | IKBKG | 5.33 | 3.00 | 0.72 | 6 | 0.48 |
| 4.78 | 0.25 | 0.21 | 0.00 | 2.00 | 9.00 | CARD11 | 3.00 | 2.00 | 0.75 | 6690 | 0.50 |
| 4.09 | 0.62 | 0.24 | 0.17 | 4.00 | 8.00 | IL23A | 3.75 | 4.00 | 0.79 | 25696 | 0.30 |
| 2.50 | 0.00 | 0.40 | 0.00 | 1.00 | 4.00 | ARRB1 | 2.00 | 1.00 | 0.63 | 0 | 0.00 |
| 1.75 | 0.50 | 0.57 | 0.00 | 2.00 | 3.00 | IL27RA | 1.50 | 2.00 | 0.81 | 6 | 0.50 |
| 5.97 | 0.00 | 0.17 | 0.67 | 4.00 | 11.00 | IL2RB | 7.50 | 4.00 | 0.67 | 16 | 0.54 |
| 2.44 | 0.22 | 0.41 | 0.00 | 2.00 | 4.00 | ASAP1 | 2.50 | 2.00 | 0.71 | 20 | 0.50 |
| 1.78 | 0.49 | 0.56 | 0.17 | 4.00 | 3.00 | IL6R | 2.50 | 4.00 | 0.84 | 44 | 0.40 |
| 1.00 | 0.00 | 1.00 | 0.00 | 1.00 | 1.00 | FCER2 | 1.00 | 1.00 | 1.00 | 0 | 0.00 |
| 1.00 | 0.00 | 1.00 | 0.00 | 1.00 | 1.00 | IL9R | 1.00 | 1.00 | 1.00 | 0 | 0.00 |
| 3.93 | 0.00 | 0.25 | 1.00 | 2.00 | 6.00 | ARPC5 | 7.00 | 2.00 | 0.73 | 0 | 0.64 |
| 3.59 | 0.20 | 0.28 | 0.10 | 7.00 | 6.00 | IRAK4 | 3.29 | 7.00 | 0.76 | 2388 | 0.19 |
| 3.85 | 0.25 | 0.26 | 0.00 | 3.00 | 7.00 | ATG12 | 5.67 | 3.00 | 0.74 | 3066 | 0.33 |
| 4.57 | 0.00 | 0.22 | 0.00 | 1.00 | 7.00 | CD48 | 7.00 | 1.00 | 0.68 | 0 | 0.00 |
| 3.84 | 0.02 | 0.26 | 0.50 | 5.00 | 8.00 | IRF1 | 9.20 | 5.00 | 0.74 | 494 | 0.41 |
| 4.98 | 0.00 | 0.20 | 0.88 | 17.00 | 11.00 | ITGA2B | 17.88 | 17.00 | 0.73 | 688 | 0.66 |
| 1.00 | 0.00 | 1.00 | 0.00 | 1.00 | 1.00 | IFNK | 1.00 | 1.00 | 1.00 | 0 | 0.00 |
| 1.00 | 0.00 | 1.00 | 0.00 | 1.00 | 1.00 | ITGAL | 1.00 | 1.00 | 1.00 | 0 | 0.00 |
| 4.00 | 0.00 | 0.25 | 0.00 | 1.00 | 6.00 | ITGB2 | 5.00 | 1.00 | 0.63 | 0 | 0.00 |
| 4.92 | 0.01 | 0.20 | 0.77 | 21.00 | 11.00 | ITGB3 | 16.71 | 21.00 | 0.74 | 1460 | 0.60 |
| 7.72 | 0.00 | 0.13 | 0.00 | 1.00 | 13.00 | CD244 | 3.00 | 1.00 | 0.55 | 0 | 0.00 |
| 6.73 | 0.11 | 0.15 | 0.00 | 3.00 | 12.00 | ITGB7 | 2.33 | 3.00 | 0.62 | 2478 | 0.33 |
| 5.85 | 0.15 | 0.17 | 0.00 | 3.00 | 11.00 | FYN | 3.00 | 3.00 | 0.68 | 3442 | 0.33 |
| 7.92 | 0.00 | 0.13 | 0.00 | 1.00 | 13.00 | CBLB | 2.00 | 1.00 | 0.54 | 0 | 0.00 |
| 6.93 | 0.02 | 0.14 | 0.00 | 2.00 | 12.00 | ITK | 1.50 | 2.00 | 0.60 | 502 | 0.50 |
| 5.97 | 0.04 | 0.17 | 0.00 | 2.00 | 11.00 | IL7R | 3.50 | 2.00 | 0.67 | 1000 | 0.50 |
| 1.00 | 0.00 | 1.00 | 0.00 | 1.00 | 1.00 | ADCY3 | 1.00 | 1.00 | 1.00 | 0 | 0.00 |
| 1.00 | 0.00 | 1.00 | 0.00 | 1.00 | 1.00 | ITPR1 | 1.00 | 1.00 | 1.00 | 0 | 0.00 |
| 1.00 | 0.00 | 1.00 | 0.00 | 1.00 | 1.00 | CXCL14 | 1.00 | 1.00 | 1.00 | 0 | 0.00 |
| 1.00 | 0.00 | 1.00 | 0.00 | 1.00 | 1.00 | JAG2 | 1.00 | 1.00 | 1.00 | 0 | 0.00 |
| 2.94 | 0.06 | 0.34 | 0.00 | 3.00 | 5.00 | JAK3 | 4.67 | 3.00 | 0.76 | 278 | 0.37 |
| 3.91 | 0.00 | 0.26 | 0.00 | 1.00 | 6.00 | CYBA | 3.00 | 1.00 | 0.64 | 0 | 0.00 |
| 4.39 | 0.49 | 0.23 | 0.69 | 20.00 | 10.00 | JAM3 | 16.25 | 20.00 | 0.77 | 24454 | 0.56 |
| 7.11 | 0.01 | 0.14 | 0.83 | 9.00 | 12.00 | KIR2DL2 | 8.44 | 9.00 | 0.59 | 462 | 0.70 |
| 7.13 | 0.00 | 0.14 | 1.00 | 8.00 | 12.00 | KIR2DL3 | 9.13 | 8.00 | 0.59 | 0 | 0.76 |
| 7.11 | 0.01 | 0.14 | 0.83 | 9.00 | 12.00 | KIR2DL4 | 8.44 | 9.00 | 0.59 | 462 | 0.70 |
| 7.13 | 0.00 | 0.14 | 1.00 | 8.00 | 12.00 | KIR2DL5A | 9.13 | 8.00 | 0.59 | 0 | 0.76 |
| 7.11 | 0.00 | 0.14 | 0.86 | 9.00 | 12.00 | KIR2DS2 | 8.44 | 9.00 | 0.59 | 10 | 0.70 |
| 7.17 | 0.00 | 0.14 | 1.00 | 4.00 | 12.00 | KIR2DS1 | 9.75 | 4.00 | 0.59 | 0 | 0.81 |
| 6.24 | 0.22 | 0.16 | 0.69 | 10.00 | 11.00 | KIR2DS3 | 7.70 | 10.00 | 0.65 | 5860 | 0.63 |
| 7.11 | 0.00 | 0.14 | 0.86 | 9.00 | 12.00 | KIR2DS4 | 8.44 | 9.00 | 0.59 | 10 | 0.70 |
| 7.13 | 0.00 | 0.14 | 1.00 | 8.00 | 12.00 | KIR2DS5 | 9.13 | 8.00 | 0.59 | 0 | 0.76 |
| 8.05 | 0.00 | 0.12 | 0.67 | 4.00 | 13.00 | KIR3DL1 | 7.75 | 4.00 | 0.53 | 4 | 0.70 |
| 8.07 | 0.00 | 0.12 | 1.00 | 2.00 | 13.00 | KIR3DL2 | 7.50 | 2.00 | 0.53 | 0 | 0.68 |
| 7.09 | 0.03 | 0.14 | 0.62 | 11.00 | 12.00 | KIR3DL3 | 7.27 | 11.00 | 0.59 | 942 | 0.61 |
| 1.00 | 0.00 | 1.00 | 0.00 | 1.00 | 1.00 | GP9 | 1.00 | 1.00 | 1.00 | 0 | 0.00 |
| 1.00 | 0.00 | 1.00 | 0.00 | 1.00 | 1.00 | KLKB1 | 1.00 | 1.00 | 1.00 | 0 | 0.00 |
| 1.00 | 0.00 | 1.00 | 0.00 | 1.00 | 1.00 | KLRC1 | 1.00 | 1.00 | 1.00 | 0 | 0.00 |
| 1.00 | 0.00 | 1.00 | 0.00 | 1.00 | 1.00 | KLRC2 | 1.00 | 1.00 | 1.00 | 0 | 0.00 |
| 5.90 | 0.02 | 0.17 | 0.54 | 8.00 | 11.00 | KLRC4-KLRK1 | 6.63 | 8.00 | 0.67 | 2488 | 0.44 |
| 6.90 | 0.00 | 0.14 | 1.00 | 2.00 | 12.00 | GZMB | 5.50 | 2.00 | 0.61 | 0 | 0.79 |
| 6.83 | 0.00 | 0.15 | 0.67 | 4.00 | 12.00 | KLRD1 | 6.25 | 4.00 | 0.61 | 20 | 0.63 |
| 5.90 | 0.02 | 0.17 | 0.54 | 8.00 | 11.00 | KLRK1 | 6.63 | 8.00 | 0.67 | 2488 | 0.44 |
| 4.15 | 0.50 | 0.24 | 0.50 | 4.00 | 8.00 | LAT | 3.75 | 4.00 | 0.79 | 24090 | 0.50 |
| 5.02 | 0.23 | 0.20 | 0.20 | 5.00 | 10.00 | LCK | 4.40 | 5.00 | 0.73 | 5366 | 0.29 |
| 2.93 | 0.38 | 0.34 | 0.33 | 9.00 | 7.00 | LCP2 | 7.44 | 9.00 | 0.82 | 6642 | 0.23 |
| 5.48 | 0.00 | 0.18 | 1.00 | 2.00 | 10.00 | APBB1IP | 4.00 | 2.00 | 0.59 | 0 | 0.67 |
| 4.51 | 0.12 | 0.22 | 0.10 | 5.00 | 9.00 | LYN | 2.20 | 5.00 | 0.68 | 1354 | 0.27 |
| 5.50 | 0.00 | 0.18 | 0.00 | 1.00 | 10.00 | FCGR2A | 5.00 | 1.00 | 0.59 | 0 | 0.00 |
| 6.84 | 0.00 | 0.15 | 0.00 | 1.00 | 12.00 | MAF | 3.00 | 1.00 | 0.61 | 0 | 0.00 |
| 5.48 | 0.10 | 0.18 | 0.10 | 5.00 | 9.00 | ATG5 | 3.00 | 5.00 | 0.59 | 1146 | 0.30 |
| 6.46 | 0.00 | 0.15 | 0.00 | 1.00 | 10.00 | MALT1 | 5.00 | 1.00 | 0.50 | 0 | 0.00 |
| 6.44 | 0.02 | 0.16 | 0.00 | 2.00 | 10.00 | MAP2K1 | 3.00 | 2.00 | 0.51 | 290 | 0.50 |
| 2.70 | 0.16 | 0.37 | 0.14 | 7.00 | 5.00 | MAP2K2 | 4.71 | 7.00 | 0.79 | 808 | 0.23 |
| 6.45 | 0.05 | 0.15 | 0.17 | 4.00 | 13.00 | MAP2K7 | 3.50 | 4.00 | 0.64 | 1658 | 0.33 |
| 7.43 | 0.00 | 0.13 | 0.00 | 1.00 | 11.00 | MAP3K8 | 2.00 | 1.00 | 0.42 | 0 | 0.00 |
| 3.33 | 0.00 | 0.30 | 0.00 | 1.00 | 5.00 | MAPK1 | 2.00 | 1.00 | 0.53 | 0 | 0.00 |
| 2.68 | 0.15 | 0.37 | 0.20 | 5.00 | 5.00 | MAPKAPK2 | 5.40 | 5.00 | 0.79 | 614 | 0.26 |
| 5.51 | 0.01 | 0.18 | 0.67 | 3.00 | 10.00 | IL1R1 | 2.67 | 3.00 | 0.59 | 272 | 0.67 |
| 6.49 | 0.00 | 0.15 | 1.00 | 2.00 | 11.00 | MDM2 | 3.00 | 2.00 | 0.50 | 0 | 1.00 |
| 3.80 | 0.13 | 0.26 | 0.00 | 3.00 | 7.00 | MFN1 | 2.67 | 3.00 | 0.75 | 2292 | 0.33 |
| 3.98 | 0.00 | 0.25 | 0.00 | 1.00 | 7.00 | MFN2 | 6.00 | 1.00 | 0.63 | 0 | 0.00 |
| 1.00 | 0.00 | 1.00 | 0.00 | 1.00 | 1.00 | MICA | 1.00 | 1.00 | 1.00 | 0 | 0.00 |
| 1.00 | 0.00 | 1.00 | 0.00 | 1.00 | 1.00 | MICB | 1.00 | 1.00 | 1.00 | 0 | 0.00 |
| 1.00 | 0.00 | 1.00 | 0.00 | 1.00 | 1.00 | CD22 | 1.00 | 1.00 | 1.00 | 0 | 0.00 |
| 1.00 | 0.00 | 1.00 | 0.00 | 1.00 | 1.00 | MS4A1 | 1.00 | 1.00 | 1.00 | 0 | 0.00 |
| 1.00 | 0.00 | 1.00 | 0.00 | 1.00 | 1.00 | IFNW1 | 1.00 | 1.00 | 1.00 | 0 | 0.00 |
| 1.00 | 0.00 | 1.00 | 0.00 | 1.00 | 1.00 | MYL10 | 1.00 | 1.00 | 1.00 | 0 | 0.00 |
| 3.77 | 0.13 | 0.27 | 0.17 | 4.00 | 6.00 | MYL12A | 5.00 | 4.00 | 0.75 | 2354 | 0.30 |
| 4.95 | 0.00 | 0.20 | 0.84 | 19.00 | 11.00 | MYL9 | 17.47 | 19.00 | 0.74 | 448 | 0.65 |
| 5.18 | 0.00 | 0.19 | 0.91 | 12.00 | 11.00 | MYLK | 18.00 | 12.00 | 0.72 | 316 | 0.75 |
| 1.67 | 0.60 | 0.60 | 0.00 | 2.00 | 2.00 | CLEC7A | 3.00 | 2.00 | 0.83 | 18 | 0.50 |
| 1.83 | 0.53 | 0.55 | 0.33 | 3.00 | 3.00 | NAMPT | 2.00 | 3.00 | 0.79 | 16 | 0.56 |
| 2.50 | 0.00 | 0.40 | 1.00 | 2.00 | 4.00 | MYD88 | 2.50 | 2.00 | 0.63 | 0 | 0.83 |
| 2.50 | 0.00 | 0.40 | 1.00 | 2.00 | 4.00 | CASP8 | 2.50 | 2.00 | 0.63 | 0 | 0.83 |
| 2.50 | 0.00 | 0.40 | 1.00 | 2.00 | 4.00 | NCF2 | 2.50 | 2.00 | 0.63 | 0 | 0.83 |
| 1.00 | 0.00 | 1.00 | 0.00 | 1.00 | 1.00 | IL12A | 1.00 | 1.00 | 1.00 | 0 | 0.00 |
| 1.00 | 0.00 | 1.00 | 0.00 | 1.00 | 1.00 | NCR2 | 1.00 | 1.00 | 1.00 | 0 | 0.00 |
| 5.50 | 0.24 | 0.18 | 0.00 | 2.00 | 10.00 | NCR3 | 6.00 | 2.00 | 0.70 | 6272 | 0.50 |
| 4.39 | 0.47 | 0.23 | 0.29 | 7.00 | 9.00 | NFATC2 | 5.57 | 7.00 | 0.77 | 20410 | 0.28 |
| 1.00 | 0.00 | 1.00 | 0.00 | 1.00 | 1.00 | JAK1 | 1.00 | 1.00 | 1.00 | 0 | 0.00 |
| 1.00 | 0.00 | 1.00 | 0.00 | 1.00 | 1.00 | NOTCH2 | 1.00 | 1.00 | 1.00 | 0 | 0.00 |
| 5.50 | 0.00 | 0.18 | 0.50 | 4.00 | 9.00 | NRAS | 4.00 | 4.00 | 0.59 | 22 | 0.40 |
| 5.57 | 0.00 | 0.18 | 1.00 | 2.00 | 9.00 | F8 | 5.50 | 2.00 | 0.58 | 0 | 0.79 |
| 2.00 | 0.00 | 0.50 | 0.00 | 1.00 | 3.00 | OAS1 | 2.00 | 1.00 | 0.67 | 0 | 0.00 |
| 1.33 | 0.67 | 0.75 | 0.00 | 2.00 | 2.00 | OAS2 | 1.50 | 2.00 | 0.89 | 4 | 0.50 |
| 2.00 | 0.00 | 0.50 | 0.00 | 1.00 | 3.00 | ISG15 | 2.00 | 1.00 | 0.67 | 0 | 0.00 |
| 1.33 | 0.67 | 0.75 | 0.00 | 2.00 | 2.00 | OAS3 | 1.50 | 2.00 | 0.89 | 4 | 0.50 |
| 2.89 | 0.11 | 0.35 | 0.27 | 6.00 | 5.00 | OTUD5 | 4.67 | 6.00 | 0.76 | 528 | 0.29 |
| 5.88 | 0.02 | 0.17 | 0.33 | 3.00 | 12.00 | P2RY12 | 11.67 | 3.00 | 0.68 | 784 | 0.54 |
| 4.99 | 0.05 | 0.20 | 0.76 | 14.00 | 11.00 | PARD3 | 16.79 | 14.00 | 0.73 | 2642 | 0.62 |
| 4.98 | 0.00 | 0.20 | 0.85 | 17.00 | 11.00 | PF4 | 17.71 | 17.00 | 0.73 | 462 | 0.66 |
| 6.86 | 0.00 | 0.15 | 0.00 | 1.00 | 13.00 | PF4V1 | 3.00 | 1.00 | 0.61 | 0 | 0.00 |
| 5.57 | 0.00 | 0.18 | 1.00 | 2.00 | 9.00 | AZI2 | 4.50 | 2.00 | 0.58 | 0 | 0.64 |
| 5.57 | 0.00 | 0.18 | 1.00 | 2.00 | 9.00 | PIK3CA | 4.50 | 2.00 | 0.58 | 0 | 0.64 |
| 5.51 | 0.01 | 0.18 | 0.67 | 3.00 | 10.00 | PIK3R5 | 2.67 | 3.00 | 0.59 | 272 | 0.67 |
| 1.00 | 0.00 | 1.00 | 0.00 | 1.00 | 1.00 | JMJD7-PLA2G4B | 1.00 | 1.00 | 1.00 | 0 | 0.00 |
| 1.00 | 0.00 | 1.00 | 0.00 | 1.00 | 1.00 | PLA2G4B | 1.00 | 1.00 | 1.00 | 0 | 0.00 |
| 8.64 | 0.00 | 0.12 | 0.00 | 1.00 | 14.00 | CDK4 | 3.00 | 1.00 | 0.49 | 0 | 0.00 |
| 7.65 | 0.07 | 0.13 | 0.00 | 3.00 | 13.00 | PLCG1 | 2.00 | 3.00 | 0.56 | 1498 | 0.33 |
| 8.61 | 0.02 | 0.12 | 0.00 | 2.00 | 14.00 | IRF4 | 2.00 | 2.00 | 0.49 | 502 | 0.50 |
| 6.56 | 0.00 | 0.15 | 1.00 | 2.00 | 13.00 | IRF3 | 4.00 | 2.00 | 0.63 | 0 | 0.67 |
| 6.56 | 0.00 | 0.15 | 1.00 | 2.00 | 13.00 | POLR1D | 4.00 | 2.00 | 0.63 | 0 | 0.67 |
| 9.60 | 0.00 | 0.10 | 0.00 | 1.00 | 15.00 | POLR2H | 2.00 | 1.00 | 0.43 | 0 | 0.00 |
| 5.24 | 0.00 | 0.19 | 1.00 | 2.00 | 10.00 | POLR3E | 6.00 | 2.00 | 0.72 | 0 | 0.67 |
| 4.92 | 0.01 | 0.20 | 0.77 | 21.00 | 11.00 | PPBP | 16.71 | 21.00 | 0.74 | 1460 | 0.60 |
| 3.13 | 0.01 | 0.32 | 0.50 | 4.00 | 6.00 | PPP1CA | 5.75 | 4.00 | 0.73 | 88 | 0.46 |
| 4.57 | 0.00 | 0.22 | 0.00 | 1.00 | 7.00 | PPP1R12A | 7.00 | 1.00 | 0.68 | 0 | 0.00 |
| 1.83 | 0.53 | 0.55 | 0.33 | 3.00 | 3.00 | PPP3CA | 2.00 | 3.00 | 0.79 | 16 | 0.56 |
| 5.57 | 0.00 | 0.18 | 1.00 | 2.00 | 9.00 | PPP3CB | 5.50 | 2.00 | 0.58 | 0 | 0.79 |
| 4.73 | 0.00 | 0.21 | 1.00 | 2.00 | 9.00 | PPP3CC | 5.50 | 2.00 | 0.75 | 0 | 0.61 |
| 1.50 | 0.00 | 0.67 | 0.00 | 1.00 | 2.00 | PPP3R2 | 2.00 | 1.00 | 0.75 | 0 | 0.00 |
| 5.46 | 0.02 | 0.18 | 0.33 | 3.00 | 10.00 | PREX1 | 2.67 | 3.00 | 0.59 | 276 | 0.47 |
| 5.92 | 0.03 | 0.17 | 0.52 | 7.00 | 11.00 | PRF1 | 6.29 | 7.00 | 0.67 | 1764 | 0.45 |
| 3.64 | 0.08 | 0.27 | 0.00 | 2.00 | 6.00 | PRKACA | 4.00 | 2.00 | 0.67 | 348 | 0.50 |
| 4.57 | 0.04 | 0.22 | 0.00 | 2.00 | 7.00 | MAPK3 | 1.50 | 2.00 | 0.55 | 176 | 0.50 |
| 1.00 | 0.67 | 1.00 | 0.33 | 3.00 | 1.00 | PRKACG | 1.67 | 3.00 | 1.00 | 4 | 0.67 |
| 1.67 | 0.00 | 0.60 | 0.00 | 1.00 | 2.00 | CXCL6 | 3.00 | 1.00 | 0.67 | 0 | 0.00 |
| 4.00 | 0.00 | 0.25 | 0.00 | 1.00 | 6.00 | PRKCD | 5.00 | 1.00 | 0.63 | 0 | 0.00 |
| 4.99 | 0.00 | 0.20 | 0.88 | 17.00 | 11.00 | PROS1 | 17.94 | 17.00 | 0.73 | 686 | 0.69 |
| 4.38 | 0.03 | 0.23 | 0.29 | 8.00 | 9.00 | PSME1 | 5.88 | 8.00 | 0.69 | 1140 | 0.32 |
| 5.37 | 0.00 | 0.19 | 0.00 | 1.00 | 10.00 | PSME2 | 8.00 | 1.00 | 0.60 | 0 | 0.00 |
| 1.00 | 0.00 | 1.00 | 0.00 | 1.00 | 1.00 | LILRB3 | 1.00 | 1.00 | 1.00 | 0 | 0.00 |
| 1.00 | 0.00 | 1.00 | 0.00 | 1.00 | 1.00 | PSTPIP1 | 1.00 | 1.00 | 1.00 | 0 | 0.00 |
| 1.50 | 0.00 | 0.67 | 0.00 | 1.00 | 2.00 | ICOSLG | 2.00 | 1.00 | 0.75 | 0 | 0.00 |
| 1.00 | 1.00 | 1.00 | 0.00 | 2.00 | 1.00 | PTGIR | 1.00 | 2.00 | 1.00 | 2 | 0.00 |
| 1.50 | 0.00 | 0.67 | 0.00 | 1.00 | 2.00 | POLR3H | 2.00 | 1.00 | 0.75 | 0 | 0.00 |
| 4.95 | 0.03 | 0.20 | 0.78 | 19.00 | 11.00 | PTGS1 | 16.89 | 19.00 | 0.74 | 1516 | 0.63 |
| 3.06 | 0.06 | 0.33 | 0.33 | 4.00 | 6.00 | PTK2B | 5.00 | 4.00 | 0.74 | 258 | 0.37 |
| 4.04 | 0.00 | 0.25 | 0.00 | 1.00 | 7.00 | PIK3CD | 4.00 | 1.00 | 0.62 | 0 | 0.00 |
| 5.04 | 0.00 | 0.20 | 0.00 | 2.00 | 8.00 | PTPN11 | 3.00 | 2.00 | 0.63 | 48 | 0.50 |
| 1.00 | 0.00 | 1.00 | 0.00 | 1.00 | 1.00 | PLCG2 | 1.00 | 1.00 | 1.00 | 0 | 0.00 |
| 1.00 | 0.00 | 1.00 | 0.00 | 1.00 | 1.00 | PTPN6 | 1.00 | 1.00 | 1.00 | 0 | 0.00 |
| 2.50 | 0.00 | 0.40 | 1.00 | 2.00 | 4.00 | PTPRC | 2.50 | 2.00 | 0.63 | 0 | 0.83 |
| 3.02 | 0.08 | 0.33 | 0.10 | 5.00 | 6.00 | RAC2 | 4.80 | 5.00 | 0.75 | 596 | 0.30 |
| 2.22 | 0.08 | 0.45 | 0.00 | 2.00 | 4.00 | RARA | 3.50 | 2.00 | 0.76 | 12 | 0.63 |
| 2.33 | 0.24 | 0.43 | 0.00 | 3.00 | 4.00 | PXN | 2.00 | 3.00 | 0.73 | 24 | 0.50 |
| 6.45 | 0.00 | 0.16 | 0.00 | 1.00 | 11.00 | RASSF5 | 3.00 | 1.00 | 0.50 | 0 | 0.00 |
| 4.48 | 0.03 | 0.22 | 0.17 | 4.00 | 9.00 | RBCK1 | 5.50 | 4.00 | 0.68 | 542 | 0.36 |
| 5.46 | 0.00 | 0.18 | 0.00 | 1.00 | 10.00 | IRF7 | 4.00 | 1.00 | 0.59 | 0 | 0.00 |
| 2.77 | 0.10 | 0.36 | 0.33 | 6.00 | 6.00 | RFXANK | 5.50 | 6.00 | 0.78 | 580 | 0.27 |
| 4.79 | 0.00 | 0.21 | 0.00 | 1.00 | 8.00 | RNASEL | 3.00 | 1.00 | 0.66 | 0 | 0.00 |
| 4.57 | 0.02 | 0.22 | 0.33 | 3.00 | 9.00 | IRF9 | 5.00 | 3.00 | 0.68 | 542 | 0.52 |
| 5.56 | 0.00 | 0.18 | 0.00 | 1.00 | 10.00 | RNF31 | 3.00 | 1.00 | 0.59 | 0 | 0.00 |
| 4.98 | 0.09 | 0.20 | 0.32 | 8.00 | 10.00 | RORA | 6.00 | 8.00 | 0.73 | 3548 | 0.31 |
| 5.97 | 0.00 | 0.17 | 0.00 | 1.00 | 11.00 | RASGRP1 | 8.00 | 1.00 | 0.67 | 0 | 0.00 |
| 3.55 | 0.04 | 0.28 | 0.00 | 3.00 | 6.00 | RPS6KB2 | 4.00 | 3.00 | 0.68 | 188 | 0.38 |
| 4.53 | 0.00 | 0.22 | 0.00 | 1.00 | 7.00 | IRAK1 | 3.00 | 1.00 | 0.56 | 0 | 0.00 |
| 3.81 | 0.02 | 0.26 | 0.33 | 3.00 | 7.00 | RUNX1 | 5.00 | 3.00 | 0.65 | 144 | 0.58 |
| 7.42 | 0.02 | 0.13 | 0.00 | 2.00 | 14.00 | RXRA | 2.50 | 2.00 | 0.57 | 818 | 0.50 |
| 8.41 | 0.00 | 0.12 | 0.00 | 1.00 | 15.00 | MYLPF | 2.00 | 1.00 | 0.51 | 0 | 0.00 |
| 1.00 | 0.00 | 1.00 | 0.00 | 1.00 | 1.00 | PAK3 | 1.00 | 1.00 | 1.00 | 0 | 0.00 |
| 1.00 | 0.00 | 1.00 | 0.00 | 1.00 | 1.00 | SERPINA5 | 1.00 | 1.00 | 1.00 | 0 | 0.00 |
| 6.05 | 0.00 | 0.17 | 0.00 | 1.00 | 11.00 | SH2D1B | 10.00 | 1.00 | 0.66 | 0 | 0.00 |
| 5.58 | 0.18 | 0.18 | 0.20 | 6.00 | 12.00 | SHARPIN | 5.67 | 6.00 | 0.69 | 4226 | 0.20 |
| 3.68 | 0.00 | 0.27 | 0.00 | 1.00 | 6.00 | SHC1 | 7.00 | 1.00 | 0.66 | 0 | 0.00 |
| 4.60 | 0.22 | 0.22 | 0.19 | 7.00 | 8.00 | SIKE1 | 2.86 | 7.00 | 0.67 | 2734 | 0.25 |
| 2.53 | 0.16 | 0.39 | 0.33 | 7.00 | 5.00 | SIPA1 | 5.71 | 7.00 | 0.81 | 730 | 0.24 |
| 1.89 | 0.39 | 0.53 | 0.33 | 3.00 | 3.00 | SOS2 | 3.00 | 3.00 | 0.82 | 28 | 0.44 |
| 3.96 | 0.00 | 0.25 | 1.00 | 2.00 | 7.00 | SPHK2 | 4.00 | 2.00 | 0.63 | 0 | 0.67 |
| 1.50 | 0.00 | 0.67 | 0.00 | 1.00 | 2.00 | F2 | 2.00 | 1.00 | 0.75 | 0 | 0.00 |
| 1.00 | 1.00 | 1.00 | 0.00 | 2.00 | 1.00 | SRC | 1.00 | 2.00 | 1.00 | 2 | 0.00 |
| 1.50 | 0.00 | 0.67 | 0.00 | 1.00 | 2.00 | GNG8 | 2.00 | 1.00 | 0.75 | 0 | 0.00 |
| 3.62 | 0.05 | 0.28 | 0.50 | 9.00 | 8.00 | STAT1 | 9.44 | 9.00 | 0.76 | 1496 | 0.33 |
| 4.38 | 0.00 | 0.23 | 0.00 | 1.00 | 8.00 | STAT2 | 15.00 | 1.00 | 0.69 | 0 | 0.00 |
| 1.89 | 0.25 | 0.53 | 0.33 | 3.00 | 3.00 | STAT5B | 3.33 | 3.00 | 0.82 | 24 | 0.50 |
| 1.00 | 0.00 | 1.00 | 0.00 | 1.00 | 1.00 | NLRP6 | 1.00 | 1.00 | 1.00 | 0 | 0.00 |
| 1.00 | 0.00 | 1.00 | 0.00 | 1.00 | 1.00 | STAT6 | 1.00 | 1.00 | 1.00 | 0 | 0.00 |
| 5.55 | 0.00 | 0.18 | 0.00 | 1.00 | 8.00 | STIM1 | 2.00 | 1.00 | 0.43 | 0 | 0.00 |
| 3.28 | 0.12 | 0.30 | 0.20 | 6.00 | 7.00 | TANK | 5.50 | 6.00 | 0.79 | 2484 | 0.27 |
| 3.93 | 0.00 | 0.25 | 1.00 | 2.00 | 7.00 | JAK2 | 6.50 | 2.00 | 0.73 | 0 | 0.65 |
| 3.66 | 0.04 | 0.27 | 0.54 | 8.00 | 8.00 | TAP1 | 9.63 | 8.00 | 0.76 | 1304 | 0.36 |
| 3.83 | 0.02 | 0.26 | 0.60 | 5.00 | 8.00 | TAP2 | 8.80 | 5.00 | 0.74 | 560 | 0.38 |
| 1.00 | 0.00 | 1.00 | 0.00 | 1.00 | 1.00 | LIMK2 | 1.00 | 1.00 | 1.00 | 0 | 0.00 |
| 1.00 | 0.00 | 1.00 | 0.00 | 1.00 | 1.00 | TAPBP | 1.00 | 1.00 | 1.00 | 0 | 0.00 |
| 4.62 | 0.02 | 0.22 | 0.00 | 2.00 | 7.00 | TBK1 | 3.00 | 2.00 | 0.67 | 334 | 0.50 |
| 5.61 | 0.00 | 0.18 | 0.00 | 1.00 | 8.00 | POLR3GL | 2.00 | 1.00 | 0.58 | 0 | 0.00 |
| 4.00 | 0.00 | 0.25 | 0.00 | 1.00 | 6.00 | TBKBP1 | 5.00 | 1.00 | 0.63 | 0 | 0.00 |
| 5.09 | 0.08 | 0.20 | 0.50 | 8.00 | 10.00 | TBX21 | 7.25 | 8.00 | 0.73 | 6022 | 0.38 |
| 4.97 | 0.00 | 0.20 | 0.84 | 19.00 | 11.00 | TBXA2R | 17.37 | 19.00 | 0.74 | 734 | 0.67 |
| 5.00 | 0.00 | 0.20 | 0.88 | 16.00 | 11.00 | TFPI | 17.88 | 16.00 | 0.73 | 686 | 0.69 |
| 2.98 | 0.06 | 0.34 | 0.00 | 3.00 | 6.00 | TGFB1 | 5.67 | 3.00 | 0.75 | 262 | 0.36 |
| 3.21 | 0.23 | 0.31 | 0.24 | 7.00 | 6.00 | TGFBR1 | 5.14 | 7.00 | 0.80 | 4974 | 0.24 |
| 1.00 | 0.00 | 1.00 | 0.00 | 1.00 | 1.00 | CD1C | 1.00 | 1.00 | 1.00 | 0 | 0.00 |
| 1.00 | 0.00 | 1.00 | 0.00 | 1.00 | 1.00 | TIAM1 | 1.00 | 1.00 | 1.00 | 0 | 0.00 |
| 1.00 | 0.00 | 1.00 | 0.00 | 1.00 | 1.00 | MAP2K6 | 1.00 | 1.00 | 1.00 | 0 | 0.00 |
| 1.00 | 0.00 | 1.00 | 0.00 | 1.00 | 1.00 | TIRAP | 1.00 | 1.00 | 1.00 | 0 | 0.00 |
| 4.56 | 0.07 | 0.22 | 0.33 | 3.00 | 9.00 | TLR2 | 3.33 | 3.00 | 0.68 | 1080 | 0.50 |
| 3.44 | 0.00 | 0.29 | 0.00 | 1.00 | 5.00 | ITGAX | 2.00 | 1.00 | 0.51 | 0 | 0.00 |
| 2.56 | 0.22 | 0.39 | 0.00 | 2.00 | 4.00 | TLR6 | 2.00 | 2.00 | 0.69 | 16 | 0.50 |
| 1.00 | 0.00 | 1.00 | 0.00 | 1.00 | 1.00 | RHOH | 1.00 | 1.00 | 1.00 | 0 | 0.00 |
| 1.00 | 0.00 | 1.00 | 0.00 | 1.00 | 1.00 | TNFRSF10A | 1.00 | 1.00 | 1.00 | 0 | 0.00 |
| 1.00 | 0.00 | 1.00 | 0.00 | 1.00 | 1.00 | FASLG | 1.00 | 1.00 | 1.00 | 0 | 0.00 |
| 1.00 | 0.00 | 1.00 | 0.00 | 1.00 | 1.00 | TNFRSF13B | 1.00 | 1.00 | 1.00 | 0 | 0.00 |
| 4.34 | 0.00 | 0.23 | 0.67 | 3.00 | 9.00 | TNFSF10 | 4.67 | 3.00 | 0.70 | 4 | 0.47 |
| 6.46 | 0.00 | 0.15 | 0.00 | 1.00 | 10.00 | TNFSF13 | 5.00 | 1.00 | 0.50 | 0 | 0.00 |
| 4.27 | 0.00 | 0.23 | 0.00 | 1.00 | 8.00 | TNFSF13B | 6.00 | 1.00 | 0.70 | 0 | 0.00 |
| 2.91 | 0.07 | 0.34 | 0.17 | 4.00 | 5.00 | TOLLIP | 4.75 | 4.00 | 0.76 | 308 | 0.30 |
| 3.96 | 0.00 | 0.25 | 1.00 | 2.00 | 7.00 | TRAF2 | 4.00 | 2.00 | 0.63 | 0 | 0.67 |
| 5.50 | 0.00 | 0.18 | 0.00 | 1.00 | 10.00 | TRIM25 | 5.00 | 1.00 | 0.59 | 0 | 0.00 |
| 4.68 | 0.00 | 0.21 | 0.00 | 1.00 | 8.00 | TRPM2 | 3.00 | 1.00 | 0.54 | 0 | 0.00 |
| 1.00 | 0.00 | 1.00 | 0.00 | 1.00 | 1.00 | CTNNA3 | 1.00 | 1.00 | 1.00 | 0 | 0.00 |
| 1.00 | 0.00 | 1.00 | 0.00 | 1.00 | 1.00 | ULBP2 | 1.00 | 1.00 | 1.00 | 0 | 0.00 |
| 1.50 | 0.00 | 0.67 | 0.00 | 1.00 | 2.00 | CD33 | 2.00 | 1.00 | 0.75 | 0 | 0.00 |
| 1.00 | 1.00 | 1.00 | 0.00 | 2.00 | 1.00 | VAMP8 | 1.00 | 2.00 | 1.00 | 2 | 0.00 |
| 1.50 | 0.00 | 0.67 | 0.00 | 1.00 | 2.00 | ITGA4 | 2.00 | 1.00 | 0.75 | 0 | 0.00 |
| 3.22 | 0.00 | 0.31 | 0.00 | 1.00 | 5.00 | VASP | 3.00 | 1.00 | 0.56 | 0 | 0.00 |
| 1.00 | 0.00 | 1.00 | 0.00 | 1.00 | 1.00 | TGFBR2 | 1.00 | 1.00 | 1.00 | 0 | 0.00 |
| 1.00 | 0.00 | 1.00 | 0.00 | 1.00 | 1.00 | VAV1 | 1.00 | 1.00 | 1.00 | 0 | 0.00 |
| 1.50 | 0.67 | 0.67 | 0.00 | 2.00 | 2.00 | VAV2 | 2.00 | 2.00 | 0.88 | 8 | 0.50 |
| 6.63 | 0.00 | 0.15 | 0.00 | 1.00 | 13.00 | VCL | 9.00 | 1.00 | 0.63 | 0 | 0.00 |
| 5.78 | 0.03 | 0.17 | 0.00 | 2.00 | 12.00 | VWF | 9.00 | 2.00 | 0.68 | 1140 | 0.50 |
| 1.00 | 0.00 | 1.00 | 0.00 | 1.00 | 1.00 | NFYC | 1.00 | 1.00 | 1.00 | 0 | 0.00 |
| 1.00 | 0.00 | 1.00 | 0.00 | 1.00 | 1.00 | WAS | 1.00 | 1.00 | 1.00 | 0 | 0.00 |
| 6.51 | 0.02 | 0.15 | 0.33 | 3.00 | 13.00 | WASF2 | 3.67 | 3.00 | 0.63 | 446 | 0.48 |
| 7.50 | 0.00 | 0.13 | 0.00 | 1.00 | 14.00 | FERMT3 | 3.00 | 1.00 | 0.57 | 0 | 0.00 |
| 1.00 | 0.00 | 1.00 | 0.00 | 1.00 | 1.00 | XCL1 | 1.00 | 1.00 | 1.00 | 0 | 0.00 |
| 1.00 | 0.00 | 1.00 | 0.00 | 1.00 | 1.00 | XCL2 | 1.00 | 1.00 | 1.00 | 0 | 0.00 |

**Supplementary Table S8: Topological analysis from GSE57065 control group**

| Average Shortest Path Length | Between -ness Centrality | Closeness Centrality | Clustering Coefficient | Degree | Eccen -tricity | name | Neighbor hood Connectivity | Number Of Directed Edges | Radiality | Stress | Topological Coefficient |
| --- | --- | --- | --- | --- | --- | --- | --- | --- | --- | --- | --- |
| 5.54 | 0.00 | 0.18 | 0.93 | 6.00 | 10.00 | ARPC1A | 9.17 | 6.00 | 0.68 | 6 | 0.57 |
| 4.67 | 0.02 | 0.21 | 0.49 | 10.00 | 9.00 | ARPC1B | 8.80 | 10.00 | 0.74 | 10268 | 0.29 |
| 5.45 | 0.01 | 0.18 | 0.52 | 7.00 | 12.00 | ARPC2 | 10.00 | 7.00 | 0.68 | 6132 | 0.39 |
| 5.56 | 0.03 | 0.18 | 0.36 | 9.00 | 12.00 | ARPC3 | 7.00 | 9.00 | 0.67 | 6646 | 0.31 |
| 1.00 | 0.00 | 1.00 | 0.00 | 1.00 | 1.00 | ACTB | 1.00 | 1.00 | 1.00 | 0 | 0.00 |
| 1.00 | 0.00 | 1.00 | 0.00 | 1.00 | 1.00 | ARRB1 | 1.00 | 1.00 | 1.00 | 0 | 0.00 |
| 4.57 | 0.11 | 0.22 | 0.29 | 19.00 | 11.00 | ATG5 | 8.00 | 19.00 | 0.74 | 40132 | 0.21 |
| 4.88 | 0.03 | 0.20 | 0.38 | 7.00 | 11.00 | BIRC2 | 7.29 | 7.00 | 0.72 | 6122 | 0.28 |
| 5.46 | 0.01 | 0.18 | 0.17 | 4.00 | 12.00 | ARHGAP5 | 7.75 | 4.00 | 0.68 | 1062 | 0.36 |
| 4.76 | 0.09 | 0.21 | 0.31 | 15.00 | 11.00 | BRCC3 | 7.73 | 15.00 | 0.73 | 22104 | 0.24 |
| 1.00 | 0.00 | 1.00 | 0.00 | 1.00 | 1.00 | C4A | 1.00 | 1.00 | 1.00 | 0 | 0.00 |
| 1.00 | 0.00 | 1.00 | 0.00 | 1.00 | 1.00 | C4B | 1.00 | 1.00 | 1.00 | 0 | 0.00 |
| 1.00 | 0.00 | 1.00 | 1.00 | 2.00 | 1.00 | CALM1 | 2.00 | 2.00 | 1.00 | 0 | 1.00 |
| 1.00 | 0.00 | 1.00 | 1.00 | 2.00 | 1.00 | CALM2 | 2.00 | 2.00 | 1.00 | 0 | 1.00 |
| 1.00 | 0.00 | 1.00 | 1.00 | 2.00 | 1.00 | CALM3 | 2.00 | 2.00 | 1.00 | 0 | 1.00 |
| 5.76 | 0.01 | 0.17 | 0.00 | 2.00 | 11.00 | BTK | 2.00 | 2.00 | 0.66 | 1236 | 0.50 |
| 6.75 | 0.00 | 0.15 | 0.00 | 1.00 | 12.00 | CALML4 | 2.00 | 1.00 | 0.59 | 0 | 0.00 |
| 6.42 | 0.00 | 0.16 | 0.00 | 2.00 | 13.00 | CARD16 | 7.00 | 2.00 | 0.61 | 72 | 0.50 |
| 6.55 | 0.00 | 0.15 | 0.00 | 1.00 | 13.00 | CASP1 | 9.00 | 1.00 | 0.60 | 0 | 0.00 |
| 4.53 | 0.02 | 0.22 | 0.00 | 2.00 | 10.00 | CASP3 | 6.00 | 2.00 | 0.75 | 6664 | 0.50 |
| 1.00 | 0.00 | 1.00 | 0.00 | 1.00 | 1.00 | CCL14 | 1.00 | 1.00 | 1.00 | 0 | 0.00 |
| 1.00 | 0.00 | 1.00 | 0.00 | 1.00 | 1.00 | CCL15 | 1.00 | 1.00 | 1.00 | 0 | 0.00 |
| 5.13 | 0.00 | 0.19 | 1.00 | 2.00 | 11.00 | CASP8 | 5.00 | 2.00 | 0.70 | 0 | 0.71 |
| 4.18 | 0.34 | 0.24 | 0.14 | 7.00 | 10.00 | CCL19 | 3.00 | 7.00 | 0.77 | 782 | 0.25 |
| 1.00 | 0.00 | 1.00 | 1.00 | 2.00 | 1.00 | CCL3 | 2.00 | 2.00 | 1.00 | 0 | 1.00 |
| 1.00 | 0.00 | 1.00 | 1.00 | 2.00 | 1.00 | CCL3L1 | 2.00 | 2.00 | 1.00 | 0 | 1.00 |
| 1.00 | 0.00 | 1.00 | 1.00 | 2.00 | 1.00 | CCL3L3 | 2.00 | 2.00 | 1.00 | 0 | 1.00 |
| 4.63 | 0.05 | 0.22 | 0.30 | 5.00 | 10.00 | BIRC3 | 5.20 | 5.00 | 0.74 | 5520 | 0.28 |
| 4.93 | 0.04 | 0.20 | 0.50 | 4.00 | 11.00 | CD24 | 7.00 | 4.00 | 0.72 | 3396 | 0.36 |
| 1.00 | 0.00 | 1.00 | 0.00 | 1.00 | 1.00 | BAD | 1.00 | 1.00 | 1.00 | 0 | 0.00 |
| 1.00 | 0.00 | 1.00 | 0.00 | 1.00 | 1.00 | CD37 | 1.00 | 1.00 | 1.00 | 0 | 0.00 |
| 5.13 | 0.02 | 0.19 | 0.40 | 6.00 | 11.00 | CD3D | 5.00 | 6.00 | 0.70 | 3344 | 0.31 |
| 5.68 | 0.02 | 0.18 | 0.67 | 3.00 | 11.00 | BLNK | 6.00 | 3.00 | 0.67 | 3894 | 0.46 |
| 6.63 | 0.03 | 0.15 | 0.33 | 4.00 | 12.00 | CD79A | 2.75 | 4.00 | 0.60 | 5264 | 0.46 |
| 7.62 | 0.00 | 0.13 | 1.00 | 2.00 | 13.00 | CD22 | 3.00 | 2.00 | 0.53 | 0 | 0.75 |
| 1.00 | 0.00 | 1.00 | 0.00 | 1.00 | 1.00 | CCL22 | 1.00 | 1.00 | 1.00 | 0 | 0.00 |
| 1.00 | 0.00 | 1.00 | 0.00 | 1.00 | 1.00 | CFB | 1.00 | 1.00 | 1.00 | 0 | 0.00 |
| 5.43 | 0.02 | 0.18 | 0.20 | 5.00 | 12.00 | CFL2 | 4.60 | 5.00 | 0.68 | 1582 | 0.28 |
| 5.37 | 0.02 | 0.19 | 0.44 | 9.00 | 12.00 | CHUK | 7.67 | 9.00 | 0.69 | 960 | 0.26 |
| 6.37 | 0.00 | 0.16 | 0.00 | 1.00 | 12.00 | CCL13 | 2.00 | 1.00 | 0.62 | 0 | 0.00 |
| 5.39 | 0.05 | 0.19 | 0.00 | 2.00 | 11.00 | CLDN18 | 3.00 | 2.00 | 0.69 | 84 | 0.50 |
| 3.64 | 0.00 | 0.27 | 0.00 | 1.00 | 6.00 | ARHGEF12 | 2.00 | 1.00 | 0.56 | 0 | 0.00 |
| 2.71 | 0.14 | 0.37 | 0.00 | 2.00 | 5.00 | CLDN6 | 3.00 | 2.00 | 0.71 | 26 | 0.50 |
| 1.86 | 0.54 | 0.54 | 0.17 | 4.00 | 3.00 | ASAP3 | 3.50 | 4.00 | 0.86 | 98 | 0.30 |
| 2.00 | 0.40 | 0.50 | 0.10 | 5.00 | 4.00 | CLEC4M | 2.40 | 5.00 | 0.83 | 72 | 0.26 |
| 3.29 | 0.00 | 0.30 | 0.00 | 1.00 | 5.00 | ADCY6 | 3.00 | 1.00 | 0.62 | 0 | 0.00 |
| 2.36 | 0.38 | 0.42 | 0.00 | 3.00 | 4.00 | COL3A1 | 2.33 | 3.00 | 0.77 | 70 | 0.33 |
| 4.95 | 0.00 | 0.20 | 0.00 | 1.00 | 8.00 | ALOX5 | 4.00 | 1.00 | 0.72 | 0 | 0.00 |
| 3.97 | 0.53 | 0.25 | 0.00 | 4.00 | 7.00 | CR1 | 2.00 | 4.00 | 0.79 | 1082 | 0.25 |
| 1.00 | 0.00 | 1.00 | 0.00 | 1.00 | 1.00 | CRK | 1.00 | 1.00 | 1.00 | 0 | 0.00 |
| 1.00 | 0.00 | 1.00 | 0.00 | 1.00 | 1.00 | CSF3R | 1.00 | 1.00 | 1.00 | 0 | 0.00 |
| 1.00 | 0.00 | 1.00 | 1.00 | 3.00 | 1.00 | DEFA1 | 3.00 | 3.00 | 1.00 | 0 | 1.00 |
| 1.00 | 0.00 | 1.00 | 1.00 | 3.00 | 1.00 | DEFA1B | 3.00 | 3.00 | 1.00 | 0 | 1.00 |
| 1.00 | 0.00 | 1.00 | 1.00 | 3.00 | 1.00 | DEFA3 | 3.00 | 3.00 | 1.00 | 0 | 1.00 |
| 1.00 | 0.00 | 1.00 | 1.00 | 3.00 | 1.00 | DEFA4 | 3.00 | 3.00 | 1.00 | 0 | 1.00 |
| 1.00 | 0.00 | 1.00 | 0.00 | 1.00 | 1.00 | DEFB103A | 1.00 | 1.00 | 1.00 | 0 | 0.00 |
| 1.00 | 0.00 | 1.00 | 0.00 | 1.00 | 1.00 | DEFB103B | 1.00 | 1.00 | 1.00 | 0 | 0.00 |
| 1.00 | 0.00 | 1.00 | 0.00 | 1.00 | 1.00 | DEFB4A | 1.00 | 1.00 | 1.00 | 0 | 0.00 |
| 1.00 | 0.00 | 1.00 | 0.00 | 1.00 | 1.00 | DEFB4B | 1.00 | 1.00 | 1.00 | 0 | 0.00 |
| 1.00 | 0.00 | 1.00 | 0.00 | 1.00 | 1.00 | CCL28 | 1.00 | 1.00 | 1.00 | 0 | 0.00 |
| 1.00 | 0.00 | 1.00 | 0.00 | 1.00 | 1.00 | DLG1 | 1.00 | 1.00 | 1.00 | 0 | 0.00 |
| 3.25 | 0.27 | 0.31 | 0.33 | 3.00 | 6.00 | CASR | 3.33 | 3.00 | 0.75 | 144 | 0.50 |
| 4.00 | 0.19 | 0.25 | 0.00 | 2.00 | 7.00 | DLL4 | 2.50 | 2.00 | 0.67 | 100 | 0.50 |
| 1.67 | 0.00 | 0.60 | 0.00 | 1.00 | 2.00 | CCR2 | 3.00 | 1.00 | 0.67 | 0 | 0.00 |
| 1.00 | 1.00 | 1.00 | 0.00 | 3.00 | 1.00 | EZR | 1.00 | 3.00 | 1.00 | 6 | 0.00 |
| 2.79 | 0.00 | 0.36 | 0.00 | 1.00 | 4.00 | F12 | 4.00 | 1.00 | 0.70 | 0 | 0.00 |
| 8.24 | 0.03 | 0.12 | 0.00 | 3.00 | 13.00 | CX3CR1 | 1.67 | 3.00 | 0.48 | 3774 | 0.33 |
| 9.24 | 0.00 | 0.11 | 0.00 | 1.00 | 14.00 | FASLG | 3.00 | 1.00 | 0.41 | 0 | 0.00 |
| 7.62 | 0.00 | 0.13 | 1.00 | 2.00 | 13.00 | FCER2 | 3.00 | 2.00 | 0.53 | 0 | 0.75 |
| 1.60 | 0.00 | 0.63 | 1.00 | 2.00 | 2.00 | CXCR2 | 3.50 | 2.00 | 0.80 | 0 | 0.70 |
| 1.40 | 0.40 | 0.71 | 0.33 | 3.00 | 2.00 | FCGR3B | 2.33 | 3.00 | 0.87 | 8 | 0.50 |
| 2.20 | 0.00 | 0.45 | 0.00 | 1.00 | 3.00 | FCGR3A | 3.00 | 1.00 | 0.60 | 0 | 0.00 |
| 2.93 | 0.00 | 0.34 | 0.00 | 1.00 | 5.00 | FGA | 5.00 | 1.00 | 0.68 | 0 | 0.00 |
| 1.50 | 0.60 | 0.67 | 0.33 | 3.00 | 2.00 | CXCR1 | 2.67 | 3.00 | 0.88 | 18 | 0.47 |
| 2.00 | 0.00 | 0.50 | 1.00 | 2.00 | 3.00 | FOS | 3.00 | 2.00 | 0.75 | 0 | 0.75 |
| 7.71 | 0.05 | 0.13 | 0.00 | 3.00 | 13.00 | BCL2L1 | 1.67 | 3.00 | 0.52 | 174 | 0.67 |
| 6.84 | 0.05 | 0.15 | 0.00 | 2.00 | 12.00 | FOXO3 | 4.00 | 2.00 | 0.58 | 168 | 0.60 |
| 5.97 | 0.29 | 0.17 | 0.00 | 5.00 | 11.00 | CSF1 | 1.80 | 5.00 | 0.64 | 652 | 0.27 |
| 1.50 | 0.00 | 0.67 | 0.00 | 1.00 | 2.00 | CLDN3 | 2.00 | 1.00 | 0.75 | 0 | 0.00 |
| 1.00 | 1.00 | 1.00 | 0.00 | 2.00 | 1.00 | FOXP3 | 1.00 | 2.00 | 1.00 | 2 | 0.00 |
| 5.38 | 0.09 | 0.19 | 0.17 | 4.00 | 10.00 | CD81 | 4.00 | 4.00 | 0.69 | 17052 | 0.32 |
| 6.32 | 0.02 | 0.16 | 0.67 | 3.00 | 11.00 | FYN | 4.00 | 3.00 | 0.62 | 5610 | 0.57 |
| 2.67 | 0.00 | 0.38 | 0.00 | 1.00 | 4.00 | ARRB2 | 3.00 | 1.00 | 0.58 | 0 | 0.00 |
| 1.83 | 0.33 | 0.55 | 0.33 | 3.00 | 3.00 | GAB2 | 2.00 | 3.00 | 0.79 | 10 | 0.56 |
| 5.70 | 0.00 | 0.18 | 0.00 | 2.00 | 12.00 | GATA3 | 6.00 | 2.00 | 0.66 | 12 | 0.63 |
| 2.10 | 0.62 | 0.48 | 0.17 | 4.00 | 4.00 | GBP1 | 2.00 | 4.00 | 0.82 | 56 | 0.38 |
| 2.90 | 0.00 | 0.34 | 1.00 | 2.00 | 5.00 | GBP2 | 3.00 | 2.00 | 0.68 | 0 | 0.75 |
| 2.90 | 0.00 | 0.34 | 1.00 | 2.00 | 5.00 | GBP4 | 3.00 | 2.00 | 0.68 | 0 | 0.75 |
| 2.00 | 0.56 | 0.50 | 0.00 | 2.00 | 3.00 | GBP5 | 4.00 | 2.00 | 0.83 | 50 | 0.50 |
| 1.00 | 0.00 | 1.00 | 0.00 | 1.00 | 1.00 | EPO | 1.00 | 1.00 | 1.00 | 0 | 0.00 |
| 1.00 | 0.00 | 1.00 | 0.00 | 1.00 | 1.00 | GNAI2 | 1.00 | 1.00 | 1.00 | 0 | 0.00 |
| 5.71 | 0.04 | 0.18 | 0.30 | 5.00 | 12.00 | GNAI3 | 4.60 | 5.00 | 0.66 | 3532 | 0.32 |
| 2.70 | 0.33 | 0.37 | 0.00 | 3.00 | 6.00 | F2 | 3.33 | 3.00 | 0.81 | 176 | 0.33 |
| 2.70 | 0.29 | 0.37 | 0.33 | 4.00 | 5.00 | GNB3 | 3.50 | 4.00 | 0.81 | 170 | 0.41 |
| 6.32 | 0.00 | 0.16 | 0.00 | 2.00 | 13.00 | GNG10 | 7.00 | 2.00 | 0.62 | 18 | 0.60 |
| 2.93 | 0.00 | 0.34 | 0.00 | 1.00 | 5.00 | GNG12 | 5.00 | 1.00 | 0.68 | 0 | 0.00 |
| 6.03 | 0.05 | 0.17 | 0.00 | 2.00 | 12.00 | F11 | 2.00 | 2.00 | 0.64 | 82 | 0.50 |
| 7.00 | 0.00 | 0.14 | 0.00 | 1.00 | 13.00 | GNG4 | 2.00 | 1.00 | 0.57 | 0 | 0.00 |
| 1.00 | 0.00 | 1.00 | 0.00 | 1.00 | 1.00 | ADCY8 | 1.00 | 1.00 | 1.00 | 0 | 0.00 |
| 1.00 | 0.00 | 1.00 | 0.00 | 1.00 | 1.00 | GRK1 | 1.00 | 1.00 | 1.00 | 0 | 0.00 |
| 5.29 | 0.01 | 0.19 | 0.00 | 2.00 | 11.00 | CCL18 | 4.00 | 2.00 | 0.69 | 24 | 0.60 |
| 5.00 | 0.11 | 0.20 | 0.00 | 3.00 | 11.00 | GSK3A | 3.67 | 3.00 | 0.71 | 270 | 0.38 |
| 5.92 | 0.05 | 0.17 | 0.00 | 2.00 | 12.00 | CLDN17 | 2.00 | 2.00 | 0.65 | 124 | 0.50 |
| 1.00 | 0.00 | 1.00 | 0.00 | 1.00 | 1.00 | GNGT1 | 1.00 | 1.00 | 1.00 | 0 | 0.00 |
| 1.00 | 0.00 | 1.00 | 0.00 | 1.00 | 1.00 | GYPA | 1.00 | 1.00 | 1.00 | 0 | 0.00 |
| 5.55 | 0.00 | 0.18 | 0.52 | 7.00 | 10.00 | HCK | 7.00 | 7.00 | 0.67 | 88 | 0.49 |
[truncated: 324,590 more chars]
