# Supplementary material for: Immune gene expression networks in sepsis: A network biology approach
Source: PLoS One. 2021 Mar 5;16(3):e0247669. doi: 10.1371/journal.pone.0247669 (PMC7935325; doi:10.1371/journal.pone.0247669)
Supplement: Attachment — Submitted filename: comments.to.reviewer.docx [file pone.0247669.s002.docx]

**PONE-D-20-17102
Immune gene expression networks in sepsis: A network biology approach**

**Reviewer #1: Kim et al present a study focus on the expression of ~1000 genes associated with inflammation and immunity in the context of sepsis. They examine publicly available, retrospective gene expression datasets to define transcriptional networks. There are multiple issues with this study that limit its usefulness and originality:**

**1. The authors focus on only 998 genes. There have been multiple studies over the past nearly 20 years that have examined the full transcriptome after sepsis (with insights into inflammation and immunity, and including detailed network analyses), and so a focus on only inflammation and immunity is anachronistic at best and very incomplete at worst.**

**-->** Thank you for reading and managing this article and the precise comments.

The definition of sepsis was revised in 2016 (SEPSIS-3) that includes dysregulated host response to infection. These response includes cellular, metabolic, circulatory, neurologic, and immune dysregulation. Oxidative stress or mitochondrial dysfunction, insulin resistance, hypoxemia or hypotension, metabolic acidosis, encephalopathy or neurologic dysregulation are known dysfunctions in sepsis. Among these dysregulated host response, the authors intended to study immune dysfunction via 998 immune related gene expression in this study.

Based on the comment, we have performed full transcriptome analysis by differentially expressed genes (DEG) analysis followed by gene set enrichment for pathway analysis (Table 3). Differentially expressed gene (DEG) was performed between control and patient group with equal sample number. After t-test, genes were selected based on false discovery rate of less than 0.01 (FDR<0.01) except GSE54514, which used global *P*-value. Based on DEG, gene set enrichment for pathway analysis using GAGE package using R software was performed (Supplementary figure S14-S19, Supplementary Table S17).

Among these pathways, the authors intended to study pathway that was related to immune function. Among up regulated or down regulated pathways with statistical significance, pathways associated with immune related pathway was searched. We have found that complement and coagulation cascade pathway and Fc epsilon RI signaling or Fc gamma R mediated phagocytosis pathway was increased in 66% (2/3) and 100% (3/3) datasets from adult and paediatric patients, respectively. T cell receptor signaling pathway was decreased in 100% (3/3) and 100% (3/3) datasets from adult and paediatric patients, respectively. Antigen presentation and processing was decreased in 66% (2/3) and 66% (2/3) datasets from adult and paediatric patients, respectively.

Altogether, these findings could be interpreted along with the network analysis results. In network analysis, adaptive immunity associated with CD3 and CD8 genes were prominent among sepsis patients (Fig. 1). T cell receptor signaling pathway was decreased in 100% (6/6) of the datasets, which implies that T cell function seemed to be decreased or impaired. In network analysis, complement and coagulation cascade was prominent in sepsis survivors of GSE95233 datasets (Fig2). Complement and coagulation cascade was increased among sepsis from 83.3% (5/6) of datasets, which seemed to be a convalescence process.

--> 1.1 Following Table was added in result section

**Table 3.** Differentially expressed gene analysis between control and sepsis followed by gene set pathway analysis

|  | GSE54514^a^ | GSE57065 | GSE95233 |  | GSE64456 | GSE66099 | GSE72829 |
| --- | --- | --- | --- | --- | --- | --- | --- |
| Patient type | Adult | Adult | Adult |  | Paediatric | Paediatric | Paediatric |
| Control cases (n) | 18 | 25 | 22 |  | 19 | 49 | 52 |
| Sequential selection  of sepsis cases (n) | 18 | 25 | 22 |  | 19 | 49 | 52 |
| Differentially expressed  Genes (n) | 2180 | 7885 | 6784 |  | 3123 | 8229 | 7463 |
| Up regulated pathway (n) | 4 | 60 | 44 |  | 13 | 78 | 51 |
| Down regulated pathway (n) | 1 | 33 | 33 |  | 16 | 33 | 15 |
| Up regulated immune  pathway |  | C, Fc, RIG | C, Fc |  | Fc, NK, K, Toll, C | K, IgA, Fc, APC, C | LK, Fc, NK, B cell, Toll, K, C |
| Down regulated immune  pathway | T cell | APC, T cell, H, K | APC, IgA, T cell, K, B cell |  | IgA, APC, T cell | T cell, H | IgA, APC, T cell |

^a^Differentially expressed gene of GSE54514 was selected based on global *P*-value (0.05) and other datasets are based on FDR (<0.01).

APC, Antigen processing and presentation; B cell, B cell receptor signaling; C, Complement and coagulation; Fc, Fc gamma R mediated phagocytosis, Fc epsilon RI signaling; H, hematopoietic cell lineage; IgA, intestinal immune network for IgA production; K, chemokine signaling; LM, leukocyte endothelial migration; NK, NK cell mediated cytotoxicity; RIG, RIG-I like receptor signaling; Toll, Toll like receptor signaling; T cell, T cell receptor signaling.

--> 1.2 Following sentence was added in result section

**Gene set enrichment for pathway analysis**

After differentially expressed gene (DEG) analysis, gene set enrichment for pathway analysis was performed based on KEGG pathway. Among up regulated or down regulated pathways with statistical significance, pathways associated with immune related pathway was searched. We have found that complement and coagulation cascade pathway and Fc epsilon RI signaling or Fc gamma R mediated phagocytosis pathway was increased in 66% (2/3) and 100% (3/3) datasets from adult and paediatric patients, respectively. T cell receptor signaling pathway was decreased in 100% (3/3) and 100% (3/3) datasets from adult and paediatric patients, respectively. Antigen presentation and processing was decreased in 66% (2/3) and 66% (2/3) datasets from adult and paediatric patients, respectively (Table 3). Altogether, these findings could be interpreted along with the network analysis results. In network analysis, adaptive immunity associated with CD3 and CD8 genes were prominent and isolated in sepsis patient group (Fig. 1). T cell receptor signaling pathway was decreased in 100% (6/6) of the datasets, and these data implies that T cell function seemed to be decreased or impaired. In network analysis, complement and coagulation cascade was prominent in sepsis survivors of GSE95233 datasets (Fig2). Complement and coagulation cascade was increased among sepsis in 83.3% (5/6) of datasets, and these data implies that complement and coagulation cascade seemed to be a involved in convalescence process.

--> 1.3 Following supplementary figures were added in supplementary materials

S14 Fig. The gene set analysis result of GSE54514. Red cells indicate up regulated gene sets, whereas gene cells indicate down regulated gene sets.

S15 Fig. The gene set analysis result of GSE57065. Red cells indicate up regulated gene sets, whereas gene cells indicate down regulated gene sets.

S16 Fig. The gene set analysis result of GSE95233. Red cells indicate up regulated gene sets, whereas gene cells indicate down regulated gene sets.

S17 Fig. The gene set analysis result of GSE64456. Red cells indicate up regulated gene sets, whereas gene cells indicate down regulated gene sets.

S18 Fig. The gene set analysis result of GSE66099. Red cells indicate up regulated gene sets, whereas gene cells indicate down regulated gene sets.

S19 Fig. The gene set analysis result of GSE72829 Red cells indicate up regulated gene sets, whereas gene cells indicate down regulated gene sets.

--> 1.4 Following paragraph was added in method section

**Gene set enrichment for pathway analysis**

Differentially expressed gene (DEG) was performed for total transcriptome between control and patient group with equal sample number. After t-test using basic functions in R software, genes were selected based on false discovery rate of less than 0.01 (FDR<0.01) ^53^. Based on DEG, gene set enrichment for pathway analysis using GAGE package using R software was performed ^54^. GAGE package applies input data to KEGG pathway. Significant pathways were plotted for heatmap that was selected based on global *P* value of less than 0.05.

**2. The criteria used for defining sepsis patients are from the 1st consensus definition; we are now at the 3rd consensus definition, and thus the applicability to the clinical setting is possibly limited.**
-->Thank you for you kind comment.

Most of the public data are studied based on sepsis-1 criteria.The definition of sepsis-1 was focused on inflammation, whereas sepsis 3 was focused on organ failure. The severe sepsis in sepsis-1 criteria are comparable to sepsis in sepsis-3 criteria. Septic shock in sepsis-1 are comparable to septic shock in sepsis-3. However, this could be limitation of this study and following sentence was added in limitation section.

--> Following sentence was added in the limiation section

" Datasets are based on sepsis-1 criteria for sepsis diagnosis, which was revised to sepsis-3 criteria. This could limit the applicability to the clinical settings."

**3. The datasets used involve a mix of adult and pediatric patients. There is extensive literature documenting differences in pediatric vs. adult sepsis patients with regard to multiple inflammatory and immune responses as well as outcomes.
-->** Thank you for your kind comments.

The actual analysis was performed based on respective datasets. Adult or paediatric group are not intermingled or analyzed together. The datasets are analyzed independently by each datasets. However, as there was error in Table1, Table 2, we corrected the error in Table 1 and Table 2 as follows. Analysis of the results were corrected according to adult and paediatric datasets, which was as follows.

--> 3.1 Following paragraph was revised in result section

**Network parameter analysis**

Comparison of topologic parameters revealed that the clustering coefficient in sepsis was increased 66% (2/3) of adult datasets and 100% (3/3) in paediatric datasets (Table 2). Network heterogeneity was increased in 100% (3/3) of adult datasets, but 100% (3/3) were decreased in paediatric datasets, respectively. Modularity value was decreased in 66% (2/3) of adult datasets, whereas modularity value was increased in 100% (3/3) of paediatric datasets in the sepsis group. Average degree and shortest path were increased in the sepsis group among 66% (2/3) of adult and paediatric datasets, respectively. Other parameters did not showed significant results between the healthy control group and the sepsis group (Table 2).

--> 3.2 Table 1 was rearranged as follows

**Table 1**. Baseline characteristics of recruited datasets for adult and paediatric patient

|  | GSE54514 | GSE57065 | GSE95233 |  | GSE64456 | GSE66099 | GSE72829 |
| --- | --- | --- | --- | --- | --- | --- | --- |
| Patient type | Adult | Adult | Adult |  | Paediatric | Paediatric | Paediatric |
| Age, mean (yr) ^a,b^ | ≥18, 60.3 | ≥18, 62 | ≥18, 65 |  | ≤2 mo, 29 d | ≤10, 3.7 | ≤17, 1.9 mo |
| Sex (male %)^b^ | 40 | 67.9 | 65 |  | 52 | 58 | 62 |
| Nonsurvivor (%) | 25.7 | 17.9 (28 d) | NA |  | NA | 16.3 | 19.2 |
| Cohort description | ICU with sepsis or  septic shock | ICU with  septic shock | ICU with  septic shock |  | ED with sepsis (PECARN study) | PICU with sepsis or  septic shock | Discovery group  (IRIS study) |
| Severity evaluation | APACHEII | SOFA | SOFA |  | YOS^b^ | PRISM | NA |
| Control (n) | 18 | 25 | 22 |  | 19 | 49 | 52 |
| Sepsis or septic shock (n) | 145 | 28 | 102 |  | 89 | 198 | 94 |
| Randomized selection without  replacement (septic shock) | 18 | 25 | - |  | - | - | - |
| Randomized selection without  replacement (sepsis) | - | - | 22 |  | 18 | 49 | 52 |
| Measured time points | 5 (1, 2, 3, 4, 5 d) | 3 (0, 24, 28 hr) | 3 (1-2, 3-4, 7-10 d) |  | 1 | 1 | 1 |
| Recruited time points^c^ | 0-24 hr | 0 - 24 hr | 1-2 d |  | NA | 0 - 24 hr | 5 d |
| Platform | Illumina, GPL6947 | Affymetrix, GPL570 | Affymetrix, GPL570 |  | Illumina, GPL10558 | Affymetrix, GPL570 | Illumina, GPL10558 |
| Reference | [11] | [12] | [16] |  | [13] | [14] | [15] |

ED, emergency department; ICU, intensive care unit; NA, data not available; hr, hour; d, day.

--> 3.3 Table 2 was rearranged as follows

**Table 2**. Network topological parameters from gene expression data from GEO dataset for adult and paediatric patient

|  | GSE54514 | | GSE57065 | | GSE95233 | | GSE95233-1 | |  | GSE64456 | | GSE66099 | | GSE72829 | |
| --- | --- | --- | --- | --- | --- | --- | --- | --- | --- | --- | --- | --- | --- | --- | --- |
| Patient type | Adult | | Adult | | Adult | | Adult | |  | Paediatirc | | Paediatirc | | Paediatirc | |
| Disese type | control | septic  shock | control | sepsis | control | control | sepsis | control |  | control | septic  shock | control | septic  shock | control | sepsis |
| Clustering coefficient | 0.319 | 0.323 | 0.328 | 0.280 | 0.328 | 0.367 | 0.390 | 0.337 |  | 0.367 | 0.390 | 0.337 | 0.388 | 0.381 | 0.435 |
| Network density | 0.013 | 0.016 | 0.012 | 0.010 | 0.012 | 0.039 | 0.026 | 0.014 |  | 0.039 | 0.026 | 0.014 | 0.012 | 0.030 | 0.034 |
| Network heterogeneity | 1.039 | 1.142 | 1.151 | 1.172 | 1.151 | 1.504 | 1.358 | 1.193 |  | 1.504 | 1.358 | 1.193 | 1.091 | 1.249 | 1.129 |
| Connected components | 27 | 30 | 42 | 61 | 42 | 34 | 33 | 47 |  | 34 | 33 | 47 | 32 | 16 | 25 |
| Network diameter | 19 | 17 | 14 | 15 | 14 | 12 | 19 | 11 |  | 12 | 19 | 11 | 14 | 13 | 9 |
| Network centralization | 0.059 | 0.079 | 0.103 | 0.048 | 0.103 | 0.196 | 0.113 | 0.075 |  | 0.196 | 0.113 | 0.075 | 0.078 | 0.148 | 0.121 |
| Shortest path | 160514 | 80598 | 59638 | 17222 | 59638 | 142640 | 121152 | 11730 |  | 142640 | 121152 | 11730 | 36902 | 38092 | 22532 |
| Characteristic  path length | 7.418 | 5.267 | 5.276 | 5.088 | 5.276 | 3.862 | 7.211 | 3.482 |  | 3.862 | 7.211 | 3.482 | 4.705 | 3.92 | 2.824 |
| Average degree, <*k*> | 5.93 | 7.42 | 5.30 | 3.64 | 5.30 | 17.87 | 11.71 | 4.04 |  | 17.87 | 11.71 | 4.04 | 4.50 | 8.43 | 10.27 |
| Number of nodes, *N* | 464 | 453 | 426 | 367 | 426 | 457 | 448 | 297 |  | 457 | 448 | 297 | 380 | 284 | 299 |
| Degree correlation, μ | 0.489 | 0.508 | 0.422 | 0.810 | 0.422 | 0.403 | 0.715 | 0.546 |  | 0.403 | 0.715 | 0.546 | 0.210 | 0.475 | 0.346 |
| Modularity, *Mc* | 0.743 | 0.662 | 0.644 | 0.806 | 0.644 | 0.198 | 0.581 | 0.694 |  | 0.198 | 0.581 | 0.694 | 0.745 | 0.434 | 0.603 |

--> 3.4 Interpretation of network topology was analyzed by adult and paediatric datasets, respectively. The Following section was corrected as follows:

In that isolated cluster, *CD247* (CD3 Zeta chain), *CD8A, ITK* (tyrosine protein kinase ITK / TSK), *LAT* (linker for activation of T cells), *LCK* (leukocyte C-terminal Src kinase) were found in all 6 datasets. *CD2, FYN* (Src family tyrosine kinase), *GATA3* (GATA-binding protein 3), *IL7R, RASGRP1* (RAS guanyl-releasing protein 1) were all found in adult datasets, but 66% (2/3) were found in paediatric group. *CBLB* (E2 ubiquitin protein ligase CBL-B), *CD3D*, *CD3G*, *ZAP70* (Zeta chain-associated protein kinase70) were found in 66% (2/3) of adult datasets, but all were found in paediatric datasets.

-->3.5 Interpretation of network parameters was analyzed by adult and paediatric datasets, respectively. The Following section was corrected as follows:

Comparison of topologic parameters revealed that the clustering coefficient in sepsis was increased 66% (2/3) of adult datasets and 100% (3/3) in paediatric datasets (Table 2). Network heterogeneity was increased in 100% (3/3) of adult datasets, but 100% (3/3) were decreased in paediatric datasets, respectively. Modularity value was decreased in 66% (2/3) of adult datasets, whereas modularity value was increased in 100% (3/3) of paediatric datasets in the sepsis group. Average degree and shortest path were increased in the sepsis group among 66% (2/3) of adult and paediatric datasets, respectively. Other parameters did not showed significant results between the healthy control group and the sepsis group (Table 2).

**4. The source biological material for the transcriptomic analyses is not described. These data are could be from peripheral blood mononuclear cells or whole blood (or were they derived from other tissues?), making interpretation of this study and comparison to other studies difficult.**

**-->** Thank you for your kind comments.

We have added following sentence in the method section

--> All the datasets used transcriptome derived from whole blood.

**Reviewer #2: Dear Associate Editor,
The manuscript 'Gene expression networks in sepsis: A network biology approach' by Kim et al analyzes various immune system pathways in patients with sepsis compared to healthy controls using network analysis approach and show that adaptive immune cells are isolated and prominent in that network in patients with sepsis. The strength of this manuscript is that they have used diverse datasets for analysis and obtained comparable results across all the datasets. However, there are some concerns in this manuscript, which the authors have failed to address.**

**Major comment:
1. In the introduction, the authors nicely summarize the importance of network approach analysis in sepsis as shown by other groups, however two key elements are missing:
a. What is unique or novel about their network approach analysis compared to others and what is the rationale behind that?
b. What was their motivation to perform such an analysis in sepsis?**

--> Thank you for your kind comments.

We have added following sentences in introduction.

1-a. These studies constructed network based on measured immune associated molecules that could be regarded as actual observable characteristics of sepsis. Measuring molecules in multiplex methods are relatively unfeasible compared to that of gene expression.

1-b. As molecules could be analyzed independently, analysis of multiple molecules as a system was required. Analysis of these molecules via network analysis including topologic parameters and visualization resulted in informative outcome.

1.1 a, b We have corrected introduction as follows.

--> The immune response between healthy and diseased states is rarely attributed to single molecules but rather to complex inter-related molecules. As sepsis-related immune response molecules are complex, a network approach might enhance our understanding of the equilibrium of the immune response in sepsis^18,19^. A network approach analysis of a small group of cytokines revealed that the network constructed from sepsis had a lower network diameter and lower shortest path and characteristic path length than the control network^20^. These topologic features were related to decreased network function or modularity of the network. In addition, compared to that of day 1, the day 4 cytokine network was decreased in sepsis, which was in line with previous literature^21^. These studies constructed network based on measured immune associated molecules that could be regarded as actual observable characteristics of sepsis. As molecules could be analyzed independently, analysis of multiple molecules as a system was required. Analysis of these molecules via network analysis including topologic parameters and visualization resulted in informative outcome. However, previous studies constructed networks based on small groups of cytokine measurements that resulted in a limited size of recruited molecules. Measuring molecules in multiplex methods are relatively unfeasible compared to that of gene expression.

**2. There is a confusion in the two main figures:
a. The dataset identifiers mentioned in the results section of the manuscript are contradictory to those in figures 1 and 2. It looks like the labeling in figure 1 and 2 are swapped.
b. The survivors and non-survivors (mentioned under figure 2 in the results section) have not been labelled in any of the main figures.**
--> Thank you for your kind comments.

We have corrected the error in the figures.

**3. The discussion section is very long and the authors need to strike a balance between the results and the discussion in the way it is written. There are many elements in the discussion section that actually belongs to the results section.**

--> Thank you for your kind comments. We have revised discussion section as follows.

3.1 Deleted sentences in discussion section are as follows.

*CD247, CD8A, ITK, LAT,* and *LCK* were found in all 6 datasets, whereas *CD2, CD3D, FYN, GATA3, IL7R, RASGRP1, ZAP70, CBLB* and *CD3G* were found in 5 datasets.

3.2 Following discussion section are revised.

most of the genes were annotated to the T cell receptor signalling pathway, which implies that these functions were dysregulated or uncontrolled with other immune networks compared to those in the healthy normal control group. Altogether, adaptive immunity, especially T cell associated gene seemed to be suppressed or dysregulated^7^.

--> most of the genes were annotated to the T cell receptor signalling pathway, which implies that these functions were dysregulated or uncontrolled with other immune networks compared to those in the healthy normal control group. In addition, gene set enrichment for pathway analysis showed that T cell receptor signalling pathway was down regulated in studies all the datasets. Altogether, adaptive immunity, especially T cell associated gene seemed to be suppressed or dysregulated^7^.

3.3 Following sentences are deleted.

In the healthy control group, the gene expression network was intermingled with that of innate and adaptive immune genes, whereas genes related to adaptive immunity were isolated and prominent in the sepsis group. In addition, isolated NK cell activity in the control group tended to be integrated into the sepsis group. Isolation or clustering of the network was formed by genes correlating within the cluster and not with the genes outside. Together, these genes might be associated with immune dysregulation or an abnormal host immune response^4,5^.

3.4 Following sentences are revised as follows

Activation of adaptive immunity is required to resolve sepsis, but the isolated and prominent adaptive immune response in this study seemed to be suppressed or dysregulated. activated without control or harmony with other networks. Complement-coagulation along with platelet-related genes seemed to be activated for convalescence. The gene cluster from nonsurvivors were similar from the genes in isolated network in sepsis. The gene cluster from nonsurvivors shared 4 genes among 5 genes from 6 datasets and 6 genes out of 9 genes from 5 data sets. The gene cluster from nonsurvivors included *GATA3*, which is known to be the master regulator of CD4 Th2 cell differentiation^45^.

-> Activation of adaptive immunity is required to resolve sepsis, but the isolated and prominent adaptive immune response in this study seemed to be suppressed or dysregulated.

3.5 Following sentences are moved from discussion to result section

The gene cluster from nonsurvivors were similar from the genes in isolated network in sepsis. shared 4 genes among 5 genes from 6 datasets and 6 genes out of 9 genes from 5 data sets. The gene cluster from nonsurvivors included *GATA3*, which is known to be the master regulator of CD4 Th2 cell differentiation^45^

3.6 Following sentence was revised as follows:

Further studies are required to understand the relation between the clustering coefficient and the inflammatory response in the immune network.

**-->** Network analysis revealed that inflammatory process seemed to be activated in sepsis group and further studies are required to understand the relation between the clustering coefficient and the inflammatory response in the immune network.

3.7 Following sentence was revised as follows:

Shortest path length tended to be decreased in the sepsis group, which was in line with previous literature^20^

--> Shortest path length was decreased in 66% (2/3) of adult and paediatric datasets, respectively, which was in line with previous literature^20^

3.8 Following sentence was revised as follows

Modularity of a network represents a subgroup or a cluster within a network^28,29^. Modularity implies dense connectivity between the nodes within the same cluster but has sparse connection with other nodes outside of the cluster^32,33^. Modularity is expected to promote evolvability and multifunctionality and function as a driving biological process^29,32^. In this study, the sepsis group showed increased modularity values in 83.3% of the studied datasets. This result might be due to the prominence or isolation of the adaptive immune network. Although modularity implies the biological process of a network, the isolation and disconnection of a module with a component of a similar functional node might result in adverse reactions in the whole network.

--> Modularity of a network represents a subgroup or a cluster within a network^28,29^. Modularity implies dense connectivity between the nodes within the same cluster but has sparse connection with other nodes outside of the cluster^32,33^. Modularity is expected to promote evolvability and multifunctionality and function as a driving biological process^29,32^.

In this study, the sepsis group in paediatric datasets showed increased modularity values in 100% of the studied datasets. On the contrary, only 33% of sepsis group in adult datasets showed increased modularity. These results are consistent with the data that sepsis group from paediatric datasets showed more significant pathways compared to that of adult group (Table 3). Although modularity implies the biological process of a network, the isolation and disconnection of a module with a component of a similar functional node might result in adverse reactions in the whole network.

3.9 Following sentence was revised as follows

Shortest path length tended to be decreased in the sepsis group, which was in line with previous literature^20^. This parameter, along with network diameter, is related to the functional and biological processes of a network and increases the probability of the presence of a sub-network or module with biological functions^29,30^. In this study, these values were decreased in the sepsis group, which might have caused decreased modularity. However, isolated and prominent module formation might have enhanced modularity values in the context of decreasing path length. It has been reported that as a network evolves, complex biological functions are executed through modules, which increases the path length or network diameter^34,35^. The inconsistency of the decreased shortest path and diameter and increased modularity and clustering coefficient might be an indication of immune dysfunction, which requires further study.

**-->** Shortest path length was decreased in 66% (2/3) of adult and paediatric datasets, respectively, which was in line with previous literature^20^. This parameter, along with network diameter, is related to the functional and biological processes of a network and increases the probability of the presence of a sub-network or module with biological functions^46,47^ . It has been reported that as a network evolves, complex biological functions are executed through modules, which increases the path length or network diameter^52,53^. The inconsistency of the decreased shortest path and increased clustering coefficient might be an indication of immune dysfunction, which requires further study.

3.10 Following sentence was deleted:

Demographic features, especially sex and age of patients greatly affect prevalence and mortality of the sepsis. There is growing evidence that there are differences in immune system between male and female due to genetic and hormonal causes^54,55^. Type I interferon activity, T cell numbers and antibody responses are greater in females than in males^55^. The sex difference in sepsis was supported by the global statistics that the females showed higher incidence of sepsis, whereas males showed higher morality^56^. The sex difference could not be analyzed in this study due to the small sample sizes and further studies are required to elucidate the sex difference.

3.11 Following sentence was deleted:

*CD247*, *CD8A*, *ITK*, *LAT*, *LCK* genes are included in the isolated network in sepsis derived from both age groups. Among them, LCK are intracellular signalling molecule associated with the cytoplasmic tail of CD4 and CD8A molecules.

3.11 Following sentence was deleted:

The sex difference in regard to immune response should be considered, because there is a growing evidence that the genetic and hormonal backgrounds are related with infection^54,55^. In this study, as the number of samples was limited, the sex difference could not be analyzed.

3.12 Following sentence was revised:

**Conclusion**

Immune dysfunction might be caused by isolation and prominence of the adaptive immune response from rest of the immune network. The increased modularity and clustering coefficient imply that a modular immune response might be upregulated in the sepsis group compared to that in the normal control group, which seems to be uncontrolled or dysregulated.

**--> Conclusion**

Immune dysfunction might be caused by isolation and prominence of the adaptive immune response from rest of the immune network. The isolated gene cluster included T cell receptor signaling gene and that pathway was down regulated by gene set enrichment for pathway analysis. T cell signaling or T cell related functions seemed to be impaired or decreased in sepsis cases. Survivors of sepsis showed a prominent cluster of genes that was related to complement and coagulation cascade. As this pathway was up regulated in most of sepsis datasets, this pathway seemed to be related with convalescence process.

**4. The current notion is that IL-10 is an anti-inflammatory cytokine, although IL-10 has been shown to be increased amongst non-survivors (ref 28), there is no direct evidence to state that IL-10 is related to unfavorable prognosis. High IL-10 response in non-survivors could be the due to the result of a compensated anti-inflammatory response in sepsis, following the initial pro-inflammation. Therefore, the discussion on high IL-10 is related to unfavorable prognosis in sepsis without a proper evidence is an overstatement and not correct.**

**-->** Thank you for your kind comment.

We have deleted following sentences.

In addition, *IL-10* was also included in that cluster; *IL-10* is a cytokine that stimulates Th2 cells, and a high concentration of IL-10 along with IL-17, IL-18 and CXCL10 was related to an unfavourable prognosis in sepsis patients^28^.

**5. The discussion about the main computational finding on the role of adaptive immune cells in sepsis identified using their network analysis and their inference in the pathophysiology of SIRS or sepsis is not clear and needs to be elucidated.**

**-->** Thank you for your kind comment.

We have performed additional analysis, which was gene set enrichment for pathway analysis (Supplementary Table S17, Supplmentary Figure S14-S19). Altogether, network analysis showed isolated component or isolated cluster of genes related to adaptive immune response. The genes in the cluster were related to adaptive immune response or T cells and the pathway analysis showed that the T cell signaling pathway was decreased in 100% (3/3) in adult and 100% (3/3) in paedicatric sepsis datasets. From these findings, T cell signaling or T cell related functions seemed to be impaired or decreased in sepsis cases. Among survivor of sepsis, complement and coagulation cascade and platelet related genes was prominent in network analysis. Gene set enrichment analysis showed that complement and coagulation cascade pathway was up regulated in 66% (2/3) in adult and 100% (3/3) in paediatric sepsis cases. From these findings, complement and coagulation pathway seemed to be associated with convalescence process during sepsis process. Clustering coefficient that was related with inflammatory process was increased in 66% (2/3) of adult and 100% (3/3) in paediatric datasets.

5.1 Following sentence was added in discussion section.

--> Altogether, network analysis showed isolated component or isolated cluster of genes related to adaptive immune response. The genes in the cluster were related to adaptive immune response or T cells and the pathway analysis showed that the T cell signaling pathway was decreased in 100% (3/3) in adult and 100% (3/3) in paedicatric sepsis datasets. From these findings, T cell signaling or T cell related functions seemed to be impaired or decreased in sepsis cases. Among survivor of sepsis, complement and coagulation cascade and platelet related genes was prominent in network analysis. Gene set enrichment analysis showed that complement and coagulation cascade pathway was up regulated in 66% (2/3) in adult and 100% (3/3) in paediatric sepsis cases. From these findings, complement and coagulation pathway seemed to be associated with convalescence process during sepsis process. Clustering coefficient that was related with inflammatory process was increased in 66% (2/3) of adult and 100% (3/3) in paediatric datasets.

5.2 Following table below from gene set enrichment for pathway analysis was added as a Supplementary Table S17

**Minor comments:
1. The authors should stick to using the abbreviations instead of full form after using it for the first time and have to be consistent throughout the manuscript.
Example: GEO - line 86, KEGG - line 150, GO - line 166
-->** Thank you for your kind comment.

We have corrected the errors as commented.

**2. Are there any references for the rationale behind using such platforms? Lines 106 and 107.**
**-->** Thank you for your kind comment.

Following articles are cited.

22. Ritchie M, Dunning M, Smith M, Shi W, Lynch A. BeadArray expression analysis using bioconductor. PLoS Comp Biol 2011; 7:e1002276. https://doi.org/10.1371/journal.pcbi.1002276.

23. Jaksik R, Iwanaszko M, Rzeszowska-Wolny R, Kimmel M. Microarray experiments and factors which affect their reliability. Biol Direct 2015; 10:46 10.1186/s13062-015-0077-2.

**3. What does '(n)' in the table 1 indicate?
-->** Thank you for your kind comment.

We are sorry for confusion . The measured frequency or frequency of blood drawn are described. For example, GSE54514 datasets showed that for eacg patients, as much as 5 time of blood dare drawn at day 1, day 2, day3, day4, and day 5 after admission. Then experimented as much as 5 time per patients. 5 (1, 2, 3, 4, 5 d)

--> We have changed the Table 1 as follows.

| Measured freqeuncy, frequency (time points) | 5 (1, 2, 3, 4, 5 d) | 3 (0, 24, 28 hr) | 3 (1-2, 3-4, 7-10 d) |  | 1 | 1 | 1 |
| --- | --- | --- | --- | --- | --- | --- | --- |

**4. Why are the figure legends not listed separately and written along with the text?**
**-->** Thank you for your kind comment.

Submission guideline states as follows.

"Figure captions must be inserted in the text of the manuscript, immediately following the paragraph in which the figure is first cited (read order). Do not include captions as part of the figure files themselves or submit them in a separate document"

**5. Maintain a similar font and size throughout the manuscript, line 298 is different.**

**-->** Thank you for your kind comment.

We have corrected the error. Font size was changed to 12 and Times New Roman was used for the font.

**Additional revision**

Following Supplementary Table S17 was added, which was the result of gene set enrichment for pathway analysis. Gene set enrichment for pathway analysis data was summarized in Table 3.

**Supplmentary Table S17.**

| **GSE54514** | p.geomean | stat.mean | p.val | q.val | set.size |
| --- | --- | --- | --- | --- | --- |
| hsa04142 Lysosome | 0.025847966 | 1.269518545 | 5.09E-08 | 8.20E-06 | 114 |
| hsa00970 Aminoacyl-tRNA biosynthesis | 0.088028345 | 0.881659727 | 0.000130305 | 0.008555202 | 40 |
| hsa04141 Protein processing in endoplasmic reticulum | 0.108398782 | 0.852836674 | 0.000159414 | 0.008555202 | 152 |
| hsa00600 Sphingolipid metabolism | 0.183824309 | 0.745837522 | 0.000888854 | 0.035776374 | 33 |
| hsa00500 Starch and sucrose metabolism | 0.174616207 | 0.592419696 | 0.00683581 | 0.22011307 | 32 |
| hsa04260 Cardiac muscle contraction | 0.150475266 | 0.517270132 | 0.015534859 | 0.353170066 | 49 |
| hsa00670 One carbon pool by folate | 0.24989082 | 0.511164476 | 0.01766595 | 0.353170066 | 15 |
| hsa03030 DNA replication | 0.140145009 | 0.502213408 | 0.019475018 | 0.353170066 | 36 |
| hsa00520 Amino sugar and nucleotide sugar metabolism | 0.185600438 | 0.492848731 | 0.019742426 | 0.353170066 | 42 |
| hsa04810 Regulation of actin cytoskeleton | 0.142203717 | 0.470551959 | 0.023133818 | 0.372454467 | 168 |
| hsa04622 RIG-I-like receptor signaling pathway | 0.198576053 | 0.463690703 | 0.025639176 | 0.375264301 | 58 |
| hsa00310 Lysine degradation | 0.258921614 | 0.443101954 | 0.031635146 | 0.424438213 | 37 |
| hsa00561 Glycerolipid metabolism | 0.292506444 | 0.427968945 | 0.035771277 | 0.428628393 | 39 |
| hsa00983 Drug metabolism - other enzymes | 0.25490034 | 0.419779579 | 0.039240986 | 0.428628393 | 27 |
| hsa00564 Glycerophospholipid metabolism | 0.255687907 | 0.414859748 | 0.040160748 | 0.428628393 | 63 |
| hsa04973 Carbohydrate digestion and absorption | 0.269226767 | 0.399200271 | 0.046898393 | 0.428628393 | 31 |
| hsa00630 Glyoxylate and dicarboxylate metabolism | 0.258395943 | 0.407462628 | 0.047746415 | 0.428628393 | 16 |
| hsa02010 ABC transporters | 0.255973642 | 0.394894213 | 0.049534371 | 0.428628393 | 32 |
| hsa03060 Protein export | 0.201584586 | 0.396801811 | 0.051659627 | 0.428628393 | 23 |
| hsa00510 N-Glycan biosynthesis | 0.258174577 | 0.38240521 | 0.054661249 | 0.428628393 | 44 |
| hsa04114 Oocyte meiosis | 0.25415967 | 0.370225054 | 0.05878054 | 0.428628393 | 97 |
| hsa03410 Base excision repair | 0.26846419 | 0.370962959 | 0.06076491 | 0.428628393 | 31 |
| hsa00512 Mucin type O-Glycan biosynthesis | 0.297860294 | 0.371924262 | 0.061232628 | 0.428628393 | 18 |
| hsa04962 Vasopressin-regulated water reabsorption | 0.307990132 | 0.356276752 | 0.066901165 | 0.448795313 | 37 |
| hsa04666 Fc gamma R-mediated phagocytosis | 0.149263639 | 0.326641851 | 0.083783981 | 0.484269587 | 85 |
| hsa04670 Leukocyte transendothelial migration | 0.179800404 | 0.325133026 | 0.084901015 | 0.484269587 | 91 |
| hsa00250 Alanine, aspartate and glutamate metabolism | 0.308043361 | 0.327973968 | 0.085817892 | 0.484269587 | 21 |
| hsa04020 Calcium signaling pathway | 0.30905421 | 0.320665521 | 0.087228474 | 0.484269587 | 121 |
| hsa00534 Glycosaminoglycan biosynthesis - heparan sulfate | 0.327068995 | 0.323210599 | 0.089572826 | 0.484269587 | 17 |
| hsa04914 Progesterone-mediated oocyte maturation | 0.318424759 | 0.316995636 | 0.090236569 | 0.484269587 | 74 |
| hsa00531 Glycosaminoglycan degradation | 0.28104011 | 0.312304755 | 0.097423406 | 0.495162094 | 17 |
| hsa00052 Galactose metabolism | 0.301508758 | 0.299776925 | 0.103657516 | 0.495162094 | 23 |
| hsa00100 Steroid biosynthesis | 0.309712284 | 0.303208041 | 0.10394196 | 0.495162094 | 17 |
| hsa04722 Neurotrophin signaling pathway | 0.209032778 | 0.296981402 | 0.104568392 | 0.495162094 | 118 |
| hsa00040 Pentose and glucuronate interconversions | 0.339423254 | 0.297522093 | 0.107723493 | 0.495528066 | 17 |
| hsa04614 Renin-angiotensin system | 0.299906414 | 0.2965293 | 0.116933139 | 0.522950984 | 10 |
| hsa00010 Glycolysis / Gluconeogenesis | 0.20023476 | 0.277225272 | 0.124573091 | 0.542061287 | 53 |
| hsa00790 Folate biosynthesis | 0.341498338 | 0.262181962 | 0.140744805 | 0.568424515 | 10 |
| hsa00640 Propanoate metabolism | 0.264798564 | 0.258017879 | 0.142572288 | 0.568424515 | 29 |
| hsa00770 Pantothenate and CoA biosynthesis | 0.290883135 | 0.2552472 | 0.146246621 | 0.568424515 | 14 |
| hsa04110 Cell cycle | 0.217646458 | 0.248708696 | 0.147876265 | 0.568424515 | 113 |
| hsa04972 Pancreatic secretion | 0.338662349 | 0.247249009 | 0.148284656 | 0.568424515 | 62 |
| hsa00982 Drug metabolism - cytochrome P450 | 0.367722445 | 0.238589699 | 0.157562697 | 0.58994405 | 35 |
| hsa04971 Gastric acid secretion | 0.3592355 | 0.233274674 | 0.162455793 | 0.594440515 | 53 |
| hsa00980 Metabolism of xenobiotics by cytochrome P450 | 0.332875037 | 0.226637555 | 0.170044422 | 0.595901983 | 38 |
| hsa00140 Steroid hormone biosynthesis | 0.354305041 | 0.22741378 | 0.170257709 | 0.595901983 | 27 |
| hsa00900 Terpenoid backbone biosynthesis | 0.332436368 | 0.218545769 | 0.184698046 | 0.630739186 | 12 |
| hsa00190 Oxidative phosphorylation | 0.037330963 | 0.208137994 | 0.191092518 | 0.630739186 | 98 |
| hsa04910 Insulin signaling pathway | 0.245798672 | 0.205116192 | 0.1919641 | 0.630739186 | 122 |
| hsa04070 Phosphatidylinositol signaling system | 0.334222679 | 0.192350212 | 0.208023871 | 0.642372746 | 63 |
| hsa04977 Vitamin digestion and absorption | 0.384343117 | 0.195255211 | 0.208279084 | 0.642372746 | 14 |
| hsa04912 GnRH signaling pathway | 0.342673996 | 0.189741884 | 0.211326016 | 0.642372746 | 77 |
| hsa00260 Glycine, serine and threonine metabolism | 0.321173596 | 0.19479429 | 0.21146432 | 0.642372746 | 22 |
| hsa04975 Fat digestion and absorption | 0.369961393 | 0.179142864 | 0.22581784 | 0.673271708 | 24 |
| hsa04510 Focal adhesion | 0.278160495 | 0.173626264 | 0.230738055 | 0.675433216 | 152 |
| hsa00562 Inositol phosphate metabolism | 0.345858678 | 0.164064696 | 0.244301366 | 0.702366426 | 49 |
| hsa04120 Ubiquitin mediated proteolysis | 0.345414226 | 0.135754803 | 0.283040175 | 0.749230808 | 124 |
| hsa00565 Ether lipid metabolism | 0.393313072 | 0.13629968 | 0.283349434 | 0.749230808 | 26 |
| hsa00511 Other glycan degradation | 0.34406099 | 0.132503787 | 0.288935834 | 0.749230808 | 15 |
| hsa00360 Phenylalanine metabolism | 0.391046096 | 0.133360583 | 0.291832263 | 0.749230808 | 11 |
| hsa04964 Proximal tubule bicarbonate reclamation | 0.37667497 | 0.132046378 | 0.293578294 | 0.749230808 | 13 |
| hsa04974 Protein digestion and absorption | 0.423776406 | 0.127078542 | 0.295942314 | 0.749230808 | 46 |
| hsa04976 Bile secretion | 0.378893273 | 0.126104602 | 0.297061262 | 0.749230808 | 41 |
| hsa00592 alpha-Linolenic acid metabolism | 0.41786042 | 0.123279378 | 0.30524373 | 0.749230808 | 10 |
| hsa00270 Cysteine and methionine metabolism | 0.318554397 | 0.120569815 | 0.309732703 | 0.749230808 | 29 |
| hsa03320 PPAR signaling pathway | 0.386684884 | 0.116911974 | 0.310656418 | 0.749230808 | 49 |
| hsa00240 Pyrimidine metabolism | 0.363458028 | 0.11620759 | 0.311791703 | 0.749230808 | 86 |
| hsa01040 Biosynthesis of unsaturated fatty acids | 0.387275259 | 0.113252693 | 0.318749406 | 0.754686093 | 17 |
| hsa04210 Apoptosis | 0.305235308 | 0.098878697 | 0.338606349 | 0.776437433 | 82 |
| hsa04610 Complement and coagulation cascades | 0.353012098 | 0.098902363 | 0.340223888 | 0.776437433 | 43 |
| hsa04662 B cell receptor signaling pathway | 0.260627497 | 0.091463676 | 0.348141754 | 0.776437433 | 71 |
| hsa04742 Taste transduction | 0.419851888 | 0.092921726 | 0.348238904 | 0.776437433 | 23 |
| hsa04740 Olfactory transduction | 0.352684989 | 0.088588164 | 0.35389701 | 0.776437433 | 91 |
| hsa00380 Tryptophan metabolism | 0.391587333 | 0.08729509 | 0.356871863 | 0.776437433 | 32 |
| hsa04920 Adipocytokine signaling pathway | 0.371053644 | 0.079635611 | 0.368659921 | 0.784034854 | 60 |
| hsa00053 Ascorbate and aldarate metabolism | 0.414632821 | 0.074706156 | 0.379728253 | 0.784034854 | 12 |
| hsa04146 Peroxisome | 0.361172032 | 0.070587225 | 0.382781845 | 0.784034854 | 61 |
| hsa03450 Non-homologous end-joining | 0.435855274 | 0.071922973 | 0.384160447 | 0.784034854 | 10 |
| hsa04115 p53 signaling pathway | 0.391925676 | 0.069158745 | 0.384712754 | 0.784034854 | 58 |
| hsa00620 Pyruvate metabolism | 0.321389717 | 0.064295199 | 0.394042236 | 0.786704121 | 36 |
| hsa04010 MAPK signaling pathway | 0.313311625 | 0.062364703 | 0.395795241 | 0.786704121 | 206 |
| hsa00290 Valine, leucine and isoleucine biosynthesis | 0.334268388 | 0.061382719 | 0.401453979 | 0.788220618 | 10 |
| hsa04520 Adherens junction | 0.324247483 | 0.048347278 | 0.416929952 | 0.793369782 | 57 |
| hsa04310 Wnt signaling pathway | 0.435163792 | 0.048277858 | 0.419061166 | 0.793369782 | 113 |
| hsa00051 Fructose and mannose metabolism | 0.341772085 | 0.043156679 | 0.425408167 | 0.793369782 | 31 |
| hsa00591 Linoleic acid metabolism | 0.446939775 | 0.042645284 | 0.429284738 | 0.793369782 | 13 |
| hsa00280 Valine, leucine and isoleucine degradation | 0.317635293 | 0.041651145 | 0.431136109 | 0.793369782 | 38 |
| hsa03015 mRNA surveillance pathway | 0.431686244 | 0.039670161 | 0.433643111 | 0.793369782 | 71 |
| hsa03018 RNA degradation | 0.346366886 | 0.032491197 | 0.446251782 | 0.807264459 | 66 |
| hsa04144 Endocytosis | 0.333055224 | 0.026846334 | 0.454833815 | 0.808758859 | 166 |
| hsa00030 Pentose phosphate pathway | 0.317824725 | 0.0257338 | 0.457249015 | 0.808758859 | 24 |
| hsa04970 Salivary secretion | 0.416543972 | 0.022445557 | 0.46214792 | 0.808758859 | 53 |
| hsa03440 Homologous recombination | 0.425157786 | 0.012762907 | 0.478044595 | 0.827582578 | 24 |
| hsa04621 NOD-like receptor signaling pathway | 0.277751297 | 0.010179839 | 0.48881845 | 0.832685052 | 53 |
| hsa04960 Aldosterone-regulated sodium reabsorption | 0.437266196 | 0.00515905 | 0.4913359 | 0.832685052 | 28 |
| hsa00603 Glycosphingolipid biosynthesis - globo series | 0.449189859 | -0.006807241 | 0.508352064 | 0.84747946 | 10 |
| hsa03050 Proteasome | 0.238915289 | -0.010484047 | 0.512024664 | 0.84747946 | 41 |
| hsa04340 Hedgehog signaling pathway | 0.461833673 | -0.009327661 | 0.515857062 | 0.84747946 | 32 |
| hsa00480 Glutathione metabolism | 0.384508884 | -0.017753272 | 0.530419193 | 0.857959851 | 41 |
| hsa00830 Retinol metabolism | 0.474820452 | -0.020041995 | 0.532894318 | 0.857959851 | 28 |
| hsa04145 Phagosome | 0.306430871 | -0.025812974 | 0.543588662 | 0.862097576 | 131 |
| hsa00601 Glycosphingolipid biosynthesis - lacto and neolacto series | 0.431939299 | -0.027274556 | 0.54617362 | 0.862097576 | 17 |
| hsa04270 Vascular smooth muscle contraction | 0.418991488 | -0.032770335 | 0.554027095 | 0.865487504 | 87 |
| hsa04916 Melanogenesis | 0.438601359 | -0.035310316 | 0.559072673 | 0.865487504 | 72 |
| hsa00330 Arginine and proline metabolism | 0.387194909 | -0.038035297 | 0.565743481 | 0.867473338 | 35 |
| hsa00350 Tyrosine metabolism | 0.451928409 | -0.047513912 | 0.578971415 | 0.871664378 | 25 |
| hsa00740 Riboflavin metabolism | 0.461009902 | -0.051380624 | 0.582426007 | 0.871664378 | 10 |
| hsa00514 Other types of O-glycan biosynthesis | 0.46499724 | -0.050843399 | 0.584718961 | 0.871664378 | 29 |
| hsa00910 Nitrogen metabolism | 0.389061199 | -0.056243514 | 0.590636298 | 0.872407743 | 14 |
| hsa00450 Selenocompound metabolism | 0.469366125 | -0.059659743 | 0.598334181 | 0.875743665 | 14 |
| hsa04664 Fc epsilon RI signaling pathway | 0.331403961 | -0.067141326 | 0.606750669 | 0.878715972 | 64 |
| hsa04630 Jak-STAT signaling pathway | 0.304332486 | -0.066561794 | 0.611280676 | 0.878715972 | 112 |
| hsa04512 ECM-receptor interaction | 0.311105659 | -0.074953004 | 0.625090399 | 0.890615525 | 51 |
| hsa03430 Mismatch repair | 0.410418436 | -0.081333155 | 0.633964455 | 0.895335765 | 23 |
| hsa04730 Long-term depression | 0.448465686 | -0.087380224 | 0.643065335 | 0.899010592 | 48 |
| hsa00410 beta-Alanine metabolism | 0.429205235 | -0.093451249 | 0.650230831 | 0.899010592 | 17 |
| hsa00071 Fatty acid metabolism | 0.427905821 | -0.097695646 | 0.658523906 | 0.899010592 | 33 |
| hsa04150 mTOR signaling pathway | 0.448876505 | -0.097928081 | 0.658902173 | 0.899010592 | 45 |
| hsa04320 Dorso-ventral axis formation | 0.450974382 | -0.112685352 | 0.680401978 | 0.920543853 | 17 |
| hsa00340 Histidine metabolism | 0.420885104 | -0.124974453 | 0.699900186 | 0.939032749 | 20 |
| hsa04710 Circadian rhythm - mammal | 0.517842981 | -0.1309111 | 0.708518549 | 0.942739557 | 20 |
| hsa00920 Sulfur metabolism | 0.463099405 | -0.146929292 | 0.725639974 | 0.949930389 | 10 |
| hsa00650 Butanoate metabolism | 0.462646611 | -0.14474415 | 0.725723216 | 0.949930389 | 20 |
| hsa00860 Porphyrin and chlorophyll metabolism | 0.352093404 | -0.166428259 | 0.75483994 | 0.963093999 | 27 |
| hsa00020 Citrate cycle (TCA cycle) | 0.345764883 | -0.170516025 | 0.758009742 | 0.963093999 | 27 |
| hsa03420 Nucleotide excision repair | 0.438925925 | -0.170040315 | 0.762146338 | 0.963093999 | 40 |
| hsa04140 Regulation of autophagy | 0.501766316 | -0.174275298 | 0.766671141 | 0.963093999 | 24 |
| hsa04380 Osteoclast differentiation | 0.148156136 | -0.189624873 | 0.786435035 | 0.963093999 | 112 |
| hsa00590 Arachidonic acid metabolism | 0.503617728 | -0.197975009 | 0.797417779 | 0.963093999 | 35 |
| hsa00533 Glycosaminoglycan biosynthesis - keratan sulfate | 0.532138836 | -0.200390682 | 0.797795062 | 0.963093999 | 12 |
| hsa03008 Ribosome biogenesis in eukaryotes | 0.333180871 | -0.200270704 | 0.798234973 | 0.963093999 | 65 |
| hsa00760 Nicotinate and nicotinamide metabolism | 0.528910076 | -0.206511288 | 0.806064252 | 0.963093999 | 20 |
| hsa04623 Cytosolic DNA-sensing pathway | 0.437963673 | -0.20638293 | 0.806666663 | 0.963093999 | 41 |
| hsa04540 Gap junction | 0.441976755 | -0.206664205 | 0.807835502 | 0.963093999 | 71 |
| hsa04530 Tight junction | 0.44193829 | -0.208545097 | 0.809906484 | 0.963093999 | 99 |
| hsa04720 Long-term potentiation | 0.490372452 | -0.21137621 | 0.813545241 | 0.963093999 | 57 |
| hsa04744 Phototransduction | 0.55914291 | -0.236412336 | 0.838759444 | 0.966055827 | 18 |
| hsa03040 Spliceosome | 0.304296201 | -0.239609263 | 0.842039058 | 0.966055827 | 122 |
| hsa00230 Purine metabolism | 0.46796738 | -0.238557953 | 0.843705602 | 0.966055827 | 136 |
| hsa04330 Notch signaling pathway | 0.46873001 | -0.241488559 | 0.844589685 | 0.966055827 | 42 |
| hsa04130 SNARE interactions in vesicular transport | 0.509554977 | -0.243339648 | 0.846048892 | 0.966055827 | 31 |
| hsa00563 Glycosylphosphatidylinositol(GPI)-anchor biosynthesis | 0.56895764 | -0.251407671 | 0.854575085 | 0.968919639 | 21 |
| hsa00532 Glycosaminoglycan biosynthesis - chondroitin sulfate | 0.502124633 | -0.262852465 | 0.862913279 | 0.971531734 | 19 |
| hsa03013 RNA transport | 0.295742887 | -0.285477866 | 0.884918199 | 0.989387709 | 131 |
| hsa04360 Axon guidance | 0.5175373 | -0.304991628 | 0.900993629 | 0.992123539 | 89 |
| hsa00604 Glycosphingolipid biosynthesis - ganglio series | 0.593477566 | -0.31452579 | 0.904852663 | 0.992123539 | 12 |
| hsa04672 Intestinal immune network for IgA production | 0.21337552 | -0.31147188 | 0.905851927 | 0.992123539 | 39 |
| hsa04370 VEGF signaling pathway | 0.533095949 | -0.347139807 | 0.927802579 | 1 | 58 |
| hsa04966 Collecting duct acid secretion | 0.54307371 | -0.35534608 | 0.930671803 | 1 | 19 |
| hsa04350 TGF-beta signaling pathway | 0.566615481 | -0.389158586 | 0.949727553 | 1 | 64 |
| hsa03022 Basal transcription factors | 0.585770861 | -0.395426272 | 0.951033122 | 1 | 30 |
| hsa04012 ErbB signaling pathway | 0.554808925 | -0.400498237 | 0.95451798 | 1 | 76 |
| hsa04640 Hematopoietic cell lineage | 0.399662255 | -0.467402264 | 0.975061345 | 1 | 76 |
| hsa04620 Toll-like receptor signaling pathway | 0.368806166 | -0.475967315 | 0.976865581 | 1 | 82 |
| hsa04062 Chemokine signaling pathway | 0.344192063 | -0.474163521 | 0.977260148 | 1 | 150 |
| hsa04612 Antigen processing and presentation | 0.214081952 | -0.481110381 | 0.977825928 | 1 | 69 |
| hsa03020 RNA polymerase | 0.620754028 | -0.494288871 | 0.980686154 | 1 | 24 |
| hsa04514 Cell adhesion molecules (CAMs) | 0.255167259 | -0.610800137 | 0.994744854 | 1 | 102 |
| hsa04650 Natural killer cell mediated cytotoxicity | 0.472831909 | -0.695347858 | 0.998274891 | 1 | 112 |
| hsa04660 T cell receptor signaling pathway | 0.667202143 | -0.862374107 | 0.999861365 | 1 | 96 |
| hsa03010 Ribosome | 0.002095243 | -1.890710517 | 1 | 1 | 88 |
| hsa00061 Fatty acid biosynthesis | NA | NA | NA | NA | 6 |
| hsa00072 Synthesis and degradation of ketone bodies | NA | NA | NA | NA | 7 |
| hsa00120 Primary bile acid biosynthesis | NA | NA | NA | NA | 9 |
| hsa00130 Ubiquinone and other terpenoid-quinone biosynthesis | NA | NA | NA | NA | 5 |
| hsa00232 Caffeine metabolism | NA | NA | NA | NA | 1 |
| hsa00300 Lysine biosynthesis | NA | NA | NA | NA | 3 |
| hsa00400 Phenylalanine, tyrosine and tryptophan biosynthesis | NA | NA | NA | NA | 3 |
| hsa00430 Taurine and hypotaurine metabolism | NA | NA | NA | NA | 6 |
| hsa00460 Cyanoamino acid metabolism | NA | NA | NA | NA | 4 |
| hsa00471 D-Glutamine and D-glutamate metabolism | NA | NA | NA | NA | 2 |
| hsa00472 D-Arginine and D-ornithine metabolism | NA | NA | NA | NA | 0 |
| hsa00730 Thiamine metabolism | NA | NA | NA | NA | 3 |
| hsa00750 Vitamin B6 metabolism | NA | NA | NA | NA | 5 |
| hsa00780 Biotin metabolism | NA | NA | NA | NA | 2 |
| hsa00785 Lipoic acid metabolism | NA | NA | NA | NA | 3 |
| hsa04122 Sulfur relay system | NA | NA | NA | NA | 9 |
| hsa04660 T cell receptor signaling pathway | 0.136214253 | -0.862374107 | 0.000138635 | 0.022320274 | 96 |
| hsa04650 Natural killer cell mediated cytotoxicity | 0.116909546 | -0.695347858 | 0.001725109 | 0.138871256 | 112 |
| hsa04514 Cell adhesion molecules (CAMs) | 0.069023632 | -0.610800137 | 0.005255146 | 0.282026165 | 102 |
| hsa03020 RNA polymerase | 0.272268632 | -0.494288871 | 0.019313846 | 0.501890441 | 24 |
| hsa04612 Antigen processing and presentation | 0.081538447 | -0.481110381 | 0.022174072 | 0.501890441 | 69 |
| hsa04062 Chemokine signaling pathway | 0.135205504 | -0.474163521 | 0.022739852 | 0.501890441 | 150 |
| hsa04620 Toll-like receptor signaling pathway | 0.13883093 | -0.475967315 | 0.023134419 | 0.501890441 | 82 |
| hsa04640 Hematopoietic cell lineage | 0.165285289 | -0.467402264 | 0.024938655 | 0.501890441 | 76 |
| hsa04012 ErbB signaling pathway | 0.277142133 | -0.400498237 | 0.04548202 | 0.735805808 | 76 |
| hsa03022 Basal transcription factors | 0.298484727 | -0.395426272 | 0.048966878 | 0.735805808 | 30 |
| hsa04350 TGF-beta signaling pathway | 0.292048704 | -0.389158586 | 0.050272447 | 0.735805808 | 64 |
| hsa04966 Collecting duct acid secretion | 0.303544751 | -0.35534608 | 0.069328197 | 0.894137288 | 19 |
| hsa04370 VEGF signaling pathway | 0.286561239 | -0.347139807 | 0.072197421 | 0.894137288 | 58 |
| hsa04672 Intestinal immune network for IgA production | 0.123902817 | -0.31147188 | 0.094148073 | 0.99625161 | 39 |
| hsa00604 Glycosphingolipid biosynthesis - ganglio series | 0.358452847 | -0.31452579 | 0.095147337 | 0.99625161 | 12 |
| hsa04360 Axon guidance | 0.301663998 | -0.304991628 | 0.099006371 | 0.99625161 | 89 |
| hsa03013 RNA transport | 0.159966018 | -0.285477866 | 0.115081801 | 1 | 131 |
| hsa00532 Glycosaminoglycan biosynthesis - chondroitin sulfate | 0.323152217 | -0.262852465 | 0.137086721 | 1 | 19 |
| hsa00563 Glycosylphosphatidylinositol(GPI)-anchor biosynthesis | 0.379562063 | -0.251407671 | 0.145424915 | 1 | 21 |
| hsa04130 SNARE interactions in vesicular transport | 0.33600731 | -0.243339648 | 0.153951108 | 1 | 31 |
| hsa04330 Notch signaling pathway | 0.306869511 | -0.241488559 | 0.155410315 | 1 | 42 |
| hsa00230 Purine metabolism | 0.31234081 | -0.238557953 | 0.156294398 | 1 | 136 |
| hsa03040 Spliceosome | 0.173578172 | -0.239609263 | 0.157960942 | 1 | 122 |
| hsa04744 Phototransduction | 0.381424186 | -0.236412336 | 0.161240556 | 1 | 18 |
| hsa04720 Long-term potentiation | 0.341097583 | -0.21137621 | 0.186454759 | 1 | 57 |
| hsa04530 Tight junction | 0.294441545 | -0.208545097 | 0.190093516 | 1 | 99 |
| hsa04540 Gap junction | 0.303891541 | -0.206664205 | 0.192164498 | 1 | 71 |
| hsa04623 Cytosolic DNA-sensing pathway | 0.304797059 | -0.20638293 | 0.193333337 | 1 | 41 |
| hsa00760 Nicotinate and nicotinamide metabolism | 0.376359987 | -0.206511288 | 0.193935748 | 1 | 20 |
| hsa03008 Ribosome biogenesis in eukaryotes | 0.221009065 | -0.200270704 | 0.201765027 | 1 | 65 |
| hsa00533 Glycosaminoglycan biosynthesis - keratan sulfate | 0.386003068 | -0.200390682 | 0.202204938 | 1 | 12 |
| hsa00590 Arachidonic acid metabolism | 0.361444745 | -0.197975009 | 0.202582221 | 1 | 35 |
| hsa04380 Osteoclast differentiation | 0.098870485 | -0.189624873 | 0.213564965 | 1 | 112 |
| hsa04140 Regulation of autophagy | 0.373873992 | -0.174275298 | 0.233328859 | 1 | 24 |
| hsa03420 Nucleotide excision repair | 0.326468419 | -0.170040315 | 0.237853662 | 1 | 40 |
| hsa00020 Citrate cycle (TCA cycle) | 0.2497233 | -0.170516025 | 0.241990258 | 1 | 27 |
| hsa00860 Porphyrin and chlorophyll metabolism | 0.26448502 | -0.166428259 | 0.24516006 | 1 | 27 |
| hsa00650 Butanoate metabolism | 0.362321227 | -0.14474415 | 0.274276784 | 1 | 20 |
| hsa00920 Sulfur metabolism | 0.364393515 | -0.146929292 | 0.274360026 | 1 | 10 |
| hsa04710 Circadian rhythm - mammal | 0.418319238 | -0.1309111 | 0.291481451 | 1 | 20 |
| hsa00340 Histidine metabolism | 0.342161218 | -0.124974453 | 0.300099814 | 1 | 20 |
| hsa04320 Dorso-ventral axis formation | 0.373595592 | -0.112685352 | 0.319598022 | 1 | 17 |
| hsa04150 mTOR signaling pathway | 0.374019224 | -0.097928081 | 0.341097827 | 1 | 45 |
| hsa00071 Fatty acid metabolism | 0.360393804 | -0.097695646 | 0.341476094 | 1 | 33 |
| hsa00410 beta-Alanine metabolism | 0.365870176 | -0.093451249 | 0.349769169 | 1 | 17 |
| hsa04730 Long-term depression | 0.382943389 | -0.087380224 | 0.356934665 | 1 | 48 |
| hsa03430 Mismatch repair | 0.361918324 | -0.081333155 | 0.366035545 | 1 | 23 |
| hsa04512 ECM-receptor interaction | 0.277348567 | -0.074953004 | 0.374909601 | 1 | 51 |
| hsa04630 Jak-STAT signaling pathway | 0.275463443 | -0.066561794 | 0.388719324 | 1 | 112 |
| hsa04664 Fc epsilon RI signaling pathway | 0.269755422 | -0.067141326 | 0.393249331 | 1 | 64 |
| hsa00450 Selenocompound metabolism | 0.425995821 | -0.059659743 | 0.401665819 | 1 | 14 |
| hsa00910 Nitrogen metabolism | 0.354601714 | -0.056243514 | 0.409363702 | 1 | 14 |
| hsa00514 Other types of O-glycan biosynthesis | 0.427357067 | -0.050843399 | 0.415281039 | 1 | 29 |
| hsa00740 Riboflavin metabolism | 0.423313094 | -0.051380624 | 0.417573993 | 1 | 10 |
| hsa00350 Tyrosine metabolism | 0.4176519 | -0.047513912 | 0.421028585 | 1 | 25 |
| hsa00330 Arginine and proline metabolism | 0.369158011 | -0.038035297 | 0.434256519 | 1 | 35 |
| hsa04916 Melanogenesis | 0.411020347 | -0.035310316 | 0.440927327 | 1 | 72 |
| hsa04270 Vascular smooth muscle contraction | 0.385386751 | -0.032770335 | 0.445972905 | 1 | 87 |
| hsa00601 Glycosphingolipid biosynthesis - lacto and neolacto series | 0.413805829 | -0.027274556 | 0.45382638 | 1 | 17 |
| hsa04145 Phagosome | 0.295731261 | -0.025812974 | 0.456411338 | 1 | 131 |
| hsa00830 Retinol metabolism | 0.457726717 | -0.020041995 | 0.467105682 | 1 | 28 |
| hsa00480 Glutathione metabolism | 0.375752091 | -0.017753272 | 0.469580807 | 1 | 41 |
| hsa04340 Hedgehog signaling pathway | 0.455113761 | -0.009327661 | 0.484142938 | 1 | 32 |
| hsa03050 Proteasome | 0.222295647 | -0.010484047 | 0.487975336 | 1 | 41 |
| hsa00603 Glycosphingolipid biosynthesis - globo series | 0.441800324 | -0.006807241 | 0.491647936 | 1 | 10 |
| hsa04960 Aldosterone-regulated sodium reabsorption | 0.440602431 | 0.00515905 | 0.5086641 | 1 | 28 |
| hsa04621 NOD-like receptor signaling pathway | 0.304210931 | 0.010179839 | 0.51118155 | 1 | 53 |
| hsa03440 Homologous recombination | 0.432630488 | 0.012762907 | 0.521955405 | 1 | 24 |
| hsa04970 Salivary secretion | 0.432697161 | 0.022445557 | 0.53785208 | 1 | 53 |
| hsa00030 Pentose phosphate pathway | 0.330594907 | 0.0257338 | 0.542750985 | 1 | 24 |
| hsa04144 Endocytosis | 0.350688271 | 0.026846334 | 0.545166185 | 1 | 166 |
| hsa03018 RNA degradation | 0.371059579 | 0.032491197 | 0.553748218 | 1 | 66 |
| hsa03015 mRNA surveillance pathway | 0.463195069 | 0.039670161 | 0.566356889 | 1 | 71 |
| hsa00280 Valine, leucine and isoleucine degradation | 0.342972261 | 0.041651145 | 0.568863891 | 1 | 38 |
| hsa00591 Linoleic acid metabolism | 0.47793164 | 0.042645284 | 0.570715262 | 1 | 13 |
| hsa00051 Fructose and mannose metabolism | 0.358838557 | 0.043156679 | 0.574591833 | 1 | 31 |
| hsa04310 Wnt signaling pathway | 0.471705527 | 0.048277858 | 0.580938834 | 1 | 113 |
| hsa04520 Adherens junction | 0.339189913 | 0.048347278 | 0.583070048 | 1 | 57 |
| hsa00290 Valine, leucine and isoleucine biosynthesis | 0.372969854 | 0.061382719 | 0.598546021 | 1 | 10 |
| hsa04010 MAPK signaling pathway | 0.34800637 | 0.062364703 | 0.604204759 | 1 | 206 |
| hsa00620 Pyruvate metabolism | 0.359944739 | 0.064295199 | 0.605957764 | 1 | 36 |
| hsa04115 p53 signaling pathway | 0.436600315 | 0.069158745 | 0.615287246 | 1 | 58 |
| hsa03450 Non-homologous end-joining | 0.489929417 | 0.071922973 | 0.615839553 | 1 | 10 |
| hsa04146 Peroxisome | 0.405958937 | 0.070587225 | 0.617218155 | 1 | 61 |
| hsa00053 Ascorbate and aldarate metabolism | 0.468576595 | 0.074706156 | 0.620271747 | 1 | 12 |
| hsa04920 Adipocytokine signaling pathway | 0.426384472 | 0.079635611 | 0.631340079 | 1 | 60 |
| hsa00380 Tryptophan metabolism | 0.452409033 | 0.08729509 | 0.643128137 | 1 | 32 |
| hsa04740 Olfactory transduction | 0.408596481 | 0.088588164 | 0.64610299 | 1 | 91 |
| hsa04742 Taste transduction | 0.486023644 | 0.092921726 | 0.651761096 | 1 | 23 |
| hsa04662 B cell receptor signaling pathway | 0.294646319 | 0.091463676 | 0.651858246 | 1 | 71 |
| hsa04610 Complement and coagulation cascades | 0.424788858 | 0.098902363 | 0.659776112 | 1 | 43 |
| hsa04210 Apoptosis | 0.365273768 | 0.098878697 | 0.661393651 | 1 | 82 |
| hsa01040 Biosynthesis of unsaturated fatty acids | 0.467302308 | 0.113252693 | 0.681250594 | 1 | 17 |
| hsa00240 Pyrimidine metabolism | 0.444599654 | 0.11620759 | 0.688208297 | 1 | 86 |
| hsa03320 PPAR signaling pathway | 0.466931096 | 0.116911974 | 0.689343582 | 1 | 49 |
| hsa00270 Cysteine and methionine metabolism | 0.401737471 | 0.120569815 | 0.690267297 | 1 | 29 |
| hsa00592 alpha-Linolenic acid metabolism | 0.508458368 | 0.123279378 | 0.69475627 | 1 | 10 |
| hsa04976 Bile secretion | 0.464640387 | 0.126104602 | 0.702938738 | 1 | 41 |
| hsa04974 Protein digestion and absorption | 0.522478806 | 0.127078542 | 0.704057686 | 1 | 46 |
| hsa04964 Proximal tubule bicarbonate reclamation | 0.468711064 | 0.132046378 | 0.706421706 | 1 | 13 |
| hsa00360 Phenylalanine metabolism | 0.483639952 | 0.133360583 | 0.708167737 | 1 | 11 |
| hsa00511 Other glycan degradation | 0.42424033 | 0.132503787 | 0.711064166 | 1 | 15 |
| hsa00565 Ether lipid metabolism | 0.490879538 | 0.13629968 | 0.716650566 | 1 | 26 |
| hsa04120 Ubiquitin mediated proteolysis | 0.439426877 | 0.135754803 | 0.716959825 | 1 | 124 |
| hsa00562 Inositol phosphate metabolism | 0.452298465 | 0.164064696 | 0.755698634 | 1 | 49 |
| hsa04510 Focal adhesion | 0.364179241 | 0.173626264 | 0.769261945 | 1 | 152 |
| hsa04975 Fat digestion and absorption | 0.496570773 | 0.179142864 | 0.77418216 | 1 | 24 |
| hsa00260 Glycine, serine and threonine metabolism | 0.45311236 | 0.19479429 | 0.78853568 | 1 | 22 |
| hsa04912 GnRH signaling pathway | 0.47169064 | 0.189741884 | 0.788673984 | 1 | 77 |
| hsa04977 Vitamin digestion and absorption | 0.527591748 | 0.195255211 | 0.791720916 | 1 | 14 |
| hsa04070 Phosphatidylinositol signaling system | 0.457367461 | 0.192350212 | 0.791976129 | 1 | 63 |
| hsa04910 Insulin signaling pathway | 0.334478863 | 0.205116192 | 0.8080359 | 1 | 122 |
| hsa00190 Oxidative phosphorylation | 0.060528215 | 0.208137994 | 0.808907445 | 1 | 98 |
| hsa00900 Terpenoid backbone biosynthesis | 0.478282933 | 0.218545769 | 0.815301954 | 1 | 12 |
| hsa00140 Steroid hormone biosynthesis | 0.518148387 | 0.22741378 | 0.829742291 | 1 | 27 |
| hsa00980 Metabolism of xenobiotics by cytochrome P450 | 0.485277778 | 0.226637555 | 0.829955578 | 1 | 38 |
| hsa04971 Gastric acid secretion | 0.530311028 | 0.233274674 | 0.837544207 | 1 | 53 |
| hsa00982 Drug metabolism - cytochrome P450 | 0.545327016 | 0.238589699 | 0.842437303 | 1 | 35 |
| hsa04972 Pancreatic secretion | 0.513530861 | 0.247249009 | 0.851715344 | 1 | 62 |
| hsa04110 Cell cycle | 0.361281654 | 0.248708696 | 0.852123735 | 1 | 113 |
| hsa00770 Pantothenate and CoA biosynthesis | 0.444532334 | 0.2552472 | 0.853753379 | 1 | 14 |
| hsa00640 Propanoate metabolism | 0.425501602 | 0.258017879 | 0.857427712 | 1 | 29 |
| hsa00790 Folate biosynthesis | 0.524051286 | 0.262181962 | 0.859255195 | 1 | 10 |
| hsa00010 Glycolysis / Gluconeogenesis | 0.346032518 | 0.277225272 | 0.875426909 | 1 | 53 |
| hsa04614 Renin-angiotensin system | 0.487961985 | 0.2965293 | 0.883066861 | 1 | 10 |
| hsa00040 Pentose and glucuronate interconversions | 0.553628545 | 0.297522093 | 0.892276507 | 1 | 17 |
| hsa04722 Neurotrophin signaling pathway | 0.349423356 | 0.296981402 | 0.895431608 | 1 | 118 |
| hsa00100 Steroid biosynthesis | 0.513321879 | 0.303208041 | 0.89605804 | 1 | 17 |
| hsa00052 Galactose metabolism | 0.488136204 | 0.299776925 | 0.896342484 | 1 | 23 |
| hsa00531 Glycosaminoglycan degradation | 0.474554399 | 0.312304755 | 0.902576594 | 1 | 17 |
| hsa04914 Progesterone-mediated oocyte maturation | 0.542899538 | 0.316995636 | 0.909763431 | 1 | 74 |
| hsa00534 Glycosaminoglycan biosynthesis - heparan sulfate | 0.558033864 | 0.323210599 | 0.910427174 | 1 | 17 |
| hsa04020 Calcium signaling pathway | 0.523004003 | 0.320665521 | 0.912771526 | 1 | 121 |
| hsa00250 Alanine, aspartate and glutamate metabolism | 0.534890255 | 0.327973968 | 0.914182108 | 1 | 21 |
| hsa04670 Leukocyte transendothelial migration | 0.317449549 | 0.325133026 | 0.915098985 | 1 | 91 |
| hsa04666 Fc gamma R-mediated phagocytosis | 0.26512417 | 0.326641851 | 0.916216019 | 1 | 85 |
| hsa04962 Vasopressin-regulated water reabsorption | 0.558749396 | 0.356276752 | 0.933098835 | 1 | 37 |
| hsa00512 Mucin type O-Glycan biosynthesis | 0.551808658 | 0.371924262 | 0.938767372 | 1 | 18 |
| hsa03410 Base excision repair | 0.515470076 | 0.370962959 | 0.93923509 | 1 | 31 |
| hsa04114 Oocyte meiosis | 0.479264611 | 0.370225054 | 0.94121946 | 1 | 97 |
| hsa00510 N-Glycan biosynthesis | 0.514702769 | 0.38240521 | 0.945338751 | 1 | 44 |
| hsa03060 Protein export | 0.417899291 | 0.396801811 | 0.948340373 | 1 | 23 |
| hsa02010 ABC transporters | 0.513031406 | 0.394894213 | 0.950465629 | 1 | 32 |
| hsa00630 Glyoxylate and dicarboxylate metabolism | 0.52364952 | 0.407462628 | 0.952253585 | 1 | 16 |
| hsa04973 Carbohydrate digestion and absorption | 0.524658775 | 0.399200271 | 0.953101607 | 1 | 31 |
| hsa00564 Glycerophospholipid metabolism | 0.525247324 | 0.414859748 | 0.959839252 | 1 | 63 |
| hsa00983 Drug metabolism - other enzymes | 0.514204782 | 0.419779579 | 0.960759014 | 1 | 27 |
| hsa00561 Glycerolipid metabolism | 0.596324792 | 0.427968945 | 0.964228723 | 1 | 39 |
| hsa00310 Lysine degradation | 0.558348525 | 0.443101954 | 0.968364854 | 1 | 37 |
| hsa04622 RIG-I-like receptor signaling pathway | 0.456901567 | 0.463690703 | 0.974360824 | 1 | 58 |
| hsa04810 Regulation of actin cytoskeleton | 0.319966449 | 0.470551959 | 0.976866182 | 1 | 168 |
| hsa00520 Amino sugar and nucleotide sugar metabolism | 0.458192385 | 0.492848731 | 0.980257574 | 1 | 42 |
| hsa03030 DNA replication | 0.38849312 | 0.502213408 | 0.980524982 | 1 | 36 |
| hsa00670 One carbon pool by folate | 0.59007797 | 0.511164476 | 0.98233405 | 1 | 15 |
| hsa04260 Cardiac muscle contraction | 0.410814559 | 0.517270132 | 0.984465141 | 1 | 49 |
| hsa00500 Starch and sucrose metabolism | 0.507331557 | 0.592419696 | 0.99316419 | 1 | 32 |
| hsa00600 Sphingolipid metabolism | 0.662377407 | 0.745837522 | 0.999111146 | 1 | 33 |
| hsa04141 Protein processing in endoplasmic reticulum | 0.559852183 | 0.852836674 | 0.999840586 | 1 | 152 |
| hsa00970 Aminoacyl-tRNA biosynthesis | 0.493250441 | 0.881659727 | 0.999869695 | 1 | 40 |
| hsa04142 Lysosome | 0.402794454 | 1.269518545 | 0.999999949 | 1 | 114 |
| hsa03010 Ribosome | 1.40E-06 | -1.890710517 | 1 | 1 | 88 |
| hsa00061 Fatty acid biosynthesis | NA | NA | NA | NA | 6 |
| hsa00072 Synthesis and degradation of ketone bodies | NA | NA | NA | NA | 7 |
| hsa00120 Primary bile acid biosynthesis | NA | NA | NA | NA | 9 |
| hsa00130 Ubiquinone and other terpenoid-quinone biosynthesis | NA | NA | NA | NA | 5 |
| hsa00232 Caffeine metabolism | NA | NA | NA | NA | 1 |
| hsa00300 Lysine biosynthesis | NA | NA | NA | NA | 3 |
| hsa00400 Phenylalanine, tyrosine and tryptophan biosynthesis | NA | NA | NA | NA | 3 |
| hsa00430 Taurine and hypotaurine metabolism | NA | NA | NA | NA | 6 |
| hsa00460 Cyanoamino acid metabolism | NA | NA | NA | NA | 4 |
| hsa00471 D-Glutamine and D-glutamate metabolism | NA | NA | NA | NA | 2 |
| hsa00472 D-Arginine and D-ornithine metabolism | NA | NA | NA | NA | 0 |
| hsa00730 Thiamine metabolism | NA | NA | NA | NA | 3 |
| hsa00750 Vitamin B6 metabolism | NA | NA | NA | NA | 5 |
| hsa00780 Biotin metabolism | NA | NA | NA | NA | 2 |
| hsa00785 Lipoic acid metabolism | NA | NA | NA | NA | 3 |
| hsa04122 Sulfur relay system | NA | NA | NA | NA | 9 |
|  |  |  |  |  |  |
| **GSE57065** | p.geomean | stat.mean | p.val | q.val | set.size |
| hsa04610 Complement and coagulation cascades | 0.006323695 | 2.557470086 | 2.86E-34 | 3.61E-32 | 22 |
| hsa03320 PPAR signaling pathway | 0.009484718 | 2.423348559 | 1.51E-31 | 9.49E-30 | 23 |
| hsa04512 ECM-receptor interaction | 0.033066077 | 1.752947614 | 5.78E-18 | 2.43E-16 | 32 |
| hsa00190 Oxidative phosphorylation | 0.033995879 | 1.648845137 | 2.61E-16 | 8.22E-15 | 55 |
| hsa04810 Regulation of actin cytoskeleton | 0.04412825 | 1.619092984 | 4.58E-16 | 1.16E-14 | 87 |
| hsa00480 Glutathione metabolism | 0.073983063 | 1.440816773 | 9.07E-13 | 1.90E-11 | 22 |
| hsa00980 Metabolism of xenobiotics by cytochrome P450 | 0.09832949 | 1.261096653 | 4.55E-10 | 8.19E-09 | 17 |
| hsa04510 Focal adhesion | 0.094310615 | 1.219373069 | 6.82E-10 | 1.07E-08 | 77 |
| hsa04115 p53 signaling pathway | 0.105763393 | 1.185104354 | 2.45E-09 | 3.43E-08 | 35 |
| hsa00564 Glycerophospholipid metabolism | 0.110800687 | 1.178702969 | 2.82E-09 | 3.55E-08 | 36 |
| hsa04270 Vascular smooth muscle contraction | 0.112105143 | 1.158940797 | 4.64E-09 | 5.31E-08 | 46 |
| hsa04110 Cell cycle | 0.084875981 | 1.156511344 | 5.05E-09 | 5.31E-08 | 73 |
| hsa04114 Oocyte meiosis | 0.106659615 | 1.148173946 | 6.18E-09 | 5.99E-08 | 57 |
| hsa04621 NOD-like receptor signaling pathway | 0.117444921 | 1.131738888 | 1.34E-08 | 1.21E-07 | 24 |
| hsa04666 Fc gamma R-mediated phagocytosis | 0.120512095 | 1.079777444 | 4.43E-08 | 3.72E-07 | 48 |
| hsa00760 Nicotinate and nicotinamide metabolism | 0.135047197 | 1.093036465 | 6.12E-08 | 4.82E-07 | 12 |
| hsa04142 Lysosome | 0.122132829 | 1.062334826 | 7.07E-08 | 5.24E-07 | 51 |
| hsa03050 Proteasome | 0.104879832 | 1.077954357 | 9.06E-08 | 6.34E-07 | 20 |
| hsa04975 Fat digestion and absorption | 0.139709561 | 1.076401023 | 1.01E-07 | 6.67E-07 | 11 |
| hsa00982 Drug metabolism - cytochrome P450 | 0.132741497 | 1.047449477 | 1.63E-07 | 1.03E-06 | 18 |
| hsa00512 Mucin type O-Glycan biosynthesis | 0.145104296 | 1.03887001 | 1.86E-07 | 1.12E-06 | 16 |
| hsa00983 Drug metabolism - other enzymes | 0.153295604 | 1.017635843 | 3.37E-07 | 1.93E-06 | 14 |
| hsa00600 Sphingolipid metabolism | 0.145321237 | 0.996496531 | 5.46E-07 | 2.99E-06 | 18 |
| hsa04914 Progesterone-mediated oocyte maturation | 0.155146284 | 0.963241312 | 8.63E-07 | 4.53E-06 | 48 |
| hsa04130 SNARE interactions in vesicular transport | 0.157969674 | 0.927354124 | 3.10E-06 | 1.56E-05 | 19 |
| hsa04620 Toll-like receptor signaling pathway | 0.154301705 | 0.906496796 | 3.63E-06 | 1.76E-05 | 40 |
| hsa00010 Glycolysis / Gluconeogenesis | 0.176676377 | 0.882224723 | 6.30E-06 | 2.94E-05 | 32 |
| hsa04380 Osteoclast differentiation | 0.174794935 | 0.837186175 | 1.57E-05 | 7.08E-05 | 63 |
| hsa04540 Gap junction | 0.190927257 | 0.822060927 | 2.33E-05 | 0.000101035 | 33 |
| hsa04910 Insulin signaling pathway | 0.190240471 | 0.814558066 | 2.54E-05 | 0.000106635 | 61 |
| hsa04740 Olfactory transduction | 0.181676931 | 0.795203478 | 4.80E-05 | 0.000194961 | 21 |
| hsa04920 Adipocytokine signaling pathway | 0.208744286 | 0.77741916 | 5.86E-05 | 0.000230558 | 30 |
| hsa00260 Glycine, serine and threonine metabolism | 0.186618432 | 0.793688228 | 6.11E-05 | 0.000233218 | 13 |
| hsa00565 Ether lipid metabolism | 0.210741096 | 0.772445822 | 7.57E-05 | 0.000280648 | 15 |
| hsa04730 Long-term depression | 0.229508887 | 0.71919779 | 0.000189212 | 0.000681163 | 22 |
| hsa04145 Phagosome | 0.201873607 | 0.700067041 | 0.000250791 | 0.00087777 | 66 |
| hsa04912 GnRH signaling pathway | 0.235485716 | 0.692392594 | 0.000290031 | 0.000987672 | 38 |
| hsa04020 Calcium signaling pathway | 0.226863057 | 0.665419209 | 0.00046548 | 0.001543434 | 60 |
| hsa04622 RIG-I-like receptor signaling pathway | 0.226783165 | 0.66978891 | 0.000496501 | 0.00160408 | 19 |
| hsa04260 Cardiac muscle contraction | 0.236662851 | 0.664122781 | 0.0005113 | 0.001610595 | 26 |
| hsa00052 Galactose metabolism | 0.241525952 | 0.658613474 | 0.000603231 | 0.001853831 | 16 |
| hsa04141 Protein processing in endoplasmic reticulum | 0.21008418 | 0.64753844 | 0.000641139 | 0.001923416 | 73 |
| hsa04976 Bile secretion | 0.22367835 | 0.613242698 | 0.001270444 | 0.003722698 | 24 |
| hsa00520 Amino sugar and nucleotide sugar metabolism | 0.238195543 | 0.606883483 | 0.001355407 | 0.003881393 | 26 |
| hsa04966 Collecting duct acid secretion | 0.264173444 | 0.607748261 | 0.001447386 | 0.004052679 | 12 |
| hsa00051 Fructose and mannose metabolism | 0.275016633 | 0.556020365 | 0.003126938 | 0.008565092 | 15 |
| hsa04370 VEGF signaling pathway | 0.276809143 | 0.535029288 | 0.003947382 | 0.010582342 | 35 |
| hsa04144 Endocytosis | 0.269651939 | 0.495980721 | 0.006748619 | 0.017715124 | 83 |
| hsa04972 Pancreatic secretion | 0.297339047 | 0.483228191 | 0.008221347 | 0.021140605 | 32 |
| hsa00240 Pyrimidine metabolism | 0.284675211 | 0.466165507 | 0.010305233 | 0.025969188 | 41 |
| hsa00380 Tryptophan metabolism | 0.298913997 | 0.450467062 | 0.013536764 | 0.033443769 | 14 |
| hsa04120 Ubiquitin mediated proteolysis | 0.266374455 | 0.430095275 | 0.016161947 | 0.039116059 | 67 |
| hsa00500 Starch and sucrose metabolism | 0.242862861 | 0.438474035 | 0.016453581 | 0.039116059 | 22 |
| hsa00330 Arginine and proline metabolism | 0.307542608 | 0.413545829 | 0.020406246 | 0.047614574 | 25 |
| hsa04623 Cytosolic DNA-sensing pathway | 0.306670012 | 0.411779465 | 0.021530656 | 0.049324777 | 16 |
| hsa04670 Leukocyte transendothelial migration | 0.316107805 | 0.397233084 | 0.024100342 | 0.05422577 | 44 |
| hsa00030 Pentose phosphate pathway | 0.333170004 | 0.372203862 | 0.034033496 | 0.075231938 | 13 |
| hsa00590 Arachidonic acid metabolism | 0.323238716 | 0.351848932 | 0.040796998 | 0.08862796 | 23 |
| hsa03440 Homologous recombination | 0.335745663 | 0.353219158 | 0.041536054 | 0.088704116 | 14 |
| hsa04664 Fc epsilon RI signaling pathway | 0.337001288 | 0.346755923 | 0.04244902 | 0.089142941 | 37 |
| hsa04146 Peroxisome | 0.334309761 | 0.331725449 | 0.049778762 | 0.102821704 | 38 |
| hsa04010 MAPK signaling pathway | 0.349075014 | 0.325789019 | 0.051986252 | 0.10564948 | 115 |
| hsa00534 Glycosaminoglycan biosynthesis - heparan sulfate | 0.350372309 | 0.317377344 | 0.059747911 | 0.119495823 | 13 |
| hsa04630 Jak-STAT signaling pathway | 0.337957504 | 0.307136844 | 0.063172461 | 0.124370783 | 58 |
| hsa00670 One carbon pool by folate | 0.355395341 | 0.287184159 | 0.079626267 | 0.154352456 | 12 |
| hsa04210 Apoptosis | 0.449418067 | 0.056735492 | 0.388846053 | 0.739940075 | 46 |
| hsa04722 Neurotrophin signaling pathway | 0.431430029 | 0.054288695 | 0.393460199 | 0.739940075 | 57 |
| hsa03022 Basal transcription factors | 0.451147091 | 0.033928731 | 0.432168589 | 0.800782974 | 18 |
| hsa03060 Protein export | 0.425263488 | 0.024222386 | 0.449584292 | 0.820980012 | 10 |
| hsa04012 ErbB signaling pathway | 0.462441166 | 0.017319915 | 0.465410076 | 0.837738138 | 35 |
| hsa02010 ABC transporters | 0.459138946 | 0.002992497 | 0.495453384 | 0.879255301 | 14 |
| hsa00071 Fatty acid metabolism | 0.482006474 | -0.012001475 | 0.523663412 | 0.916410972 | 17 |
| hsa04530 Tight junction | 0.489833858 | -0.019501103 | 0.538595435 | 0.929630477 | 44 |
| hsa04360 Axon guidance | 0.460183028 | -0.041949749 | 0.582766049 | 0.992277327 | 47 |
| hsa04974 Protein digestion and absorption | 0.443277041 | -0.055794664 | 0.607741176 | 1 | 22 |
| hsa00350 Tyrosine metabolism | 0.47591606 | -0.09133216 | 0.673147094 | 1 | 18 |
| hsa00230 Purine metabolism | 0.508506455 | -0.122534621 | 0.729318147 | 1 | 68 |
| hsa04962 Vasopressin-regulated water reabsorption | 0.538837605 | -0.160162354 | 0.785621423 | 1 | 17 |
| hsa04720 Long-term potentiation | 0.551346292 | -0.180977424 | 0.815553104 | 1 | 27 |
| hsa00514 Other types of O-glycan biosynthesis | 0.555359733 | -0.213419928 | 0.852991089 | 1 | 14 |
| hsa00561 Glycerolipid metabolism | 0.554455185 | -0.21241531 | 0.853152495 | 1 | 17 |
| hsa04971 Gastric acid secretion | 0.560430398 | -0.226702517 | 0.869182106 | 1 | 22 |
| hsa04150 mTOR signaling pathway | 0.56730353 | -0.234823538 | 0.877524828 | 1 | 21 |
| hsa04742 Taste transduction | 0.553295103 | -0.256586374 | 0.893726374 | 1 | 10 |
| hsa04970 Salivary secretion | 0.557646711 | -0.253868279 | 0.89585237 | 1 | 31 |
| hsa00830 Retinol metabolism | 0.576146507 | -0.281975685 | 0.917646471 | 1 | 16 |
| hsa00640 Propanoate metabolism | 0.588731209 | -0.284453623 | 0.920097185 | 1 | 18 |
| hsa00340 Histidine metabolism | 0.556999019 | -0.310346216 | 0.934645239 | 1 | 13 |
| hsa00620 Pyruvate metabolism | 0.569929301 | -0.310559692 | 0.937656104 | 1 | 23 |
| hsa03420 Nucleotide excision repair | 0.564507152 | -0.323576625 | 0.944667413 | 1 | 25 |
| hsa04350 TGF-beta signaling pathway | 0.603247234 | -0.336026743 | 0.95255432 | 1 | 35 |
| hsa04520 Adherens junction | 0.606326925 | -0.373886164 | 0.967849527 | 1 | 24 |
| hsa04330 Notch signaling pathway | 0.59429091 | -0.379344691 | 0.969324297 | 1 | 24 |
| hsa04960 Aldosterone-regulated sodium reabsorption | 0.632741574 | -0.416445033 | 0.978932102 | 1 | 10 |
| hsa00310 Lysine degradation | 0.630090024 | -0.462072457 | 0.988689747 | 1 | 22 |
| hsa04916 Melanogenesis | 0.646485588 | -0.460283933 | 0.988879279 | 1 | 34 |
| hsa04973 Carbohydrate digestion and absorption | 0.563684556 | -0.470555615 | 0.989102108 | 1 | 18 |
| hsa00532 Glycosaminoglycan biosynthesis - chondroitin sulfate | 0.658066183 | -0.503942878 | 0.993264607 | 1 | 13 |
| hsa00270 Cysteine and methionine metabolism | 0.679659665 | -0.544775497 | 0.996385101 | 1 | 17 |
| hsa04340 Hedgehog signaling pathway | 0.678342919 | -0.600543156 | 0.998269544 | 1 | 11 |
| hsa03430 Mismatch repair | 0.698681355 | -0.630980814 | 0.998908488 | 1 | 11 |
| hsa00562 Inositol phosphate metabolism | 0.719329022 | -0.639779258 | 0.999231949 | 1 | 26 |
| hsa04070 Phosphatidylinositol signaling system | 0.752229232 | -0.78148155 | 0.999946204 | 1 | 34 |
| hsa00510 N-Glycan biosynthesis | 0.742642409 | -0.804923087 | 0.999964123 | 1 | 24 |
| hsa00563 Glycosylphosphatidylinositol(GPI)-anchor biosynthesis | 0.754347074 | -0.827410274 | 0.99996613 | 1 | 10 |
| hsa04062 Chemokine signaling pathway | 0.725827791 | -0.831323797 | 0.999982373 | 1 | 76 |
| hsa03030 DNA replication | 0.72731904 | -0.855610501 | 0.999984305 | 1 | 17 |
| hsa04640 Hematopoietic cell lineage | 0.732854786 | -0.849686685 | 0.999987777 | 1 | 58 |
| hsa04662 B cell receptor signaling pathway | 0.760958496 | -0.898786315 | 0.999995839 | 1 | 44 |
| hsa00020 Citrate cycle (TCA cycle) | 0.742066421 | -0.929314515 | 0.999996686 | 1 | 17 |
| hsa03018 RNA degradation | 0.746401894 | -0.916010176 | 0.999997028 | 1 | 40 |
| hsa00280 Valine, leucine and isoleucine degradation | 0.760691731 | -0.957904813 | 0.999998633 | 1 | 21 |
| hsa00410 beta-Alanine metabolism | 0.769873659 | -0.98251582 | 0.999998816 | 1 | 11 |
| hsa00970 Aminoacyl-tRNA biosynthesis | 0.830592233 | -1.07549933 | 0.999999915 | 1 | 15 |
| hsa03410 Base excision repair | 0.809762962 | -1.079736532 | 0.999999921 | 1 | 15 |
| hsa04310 Wnt signaling pathway | 0.829752605 | -1.075144023 | 0.999999955 | 1 | 62 |
| hsa03010 Ribosome | 0.600564096 | -1.103220314 | 0.999999967 | 1 | 50 |
| hsa03015 mRNA surveillance pathway | 0.854753573 | -1.17004162 | 0.999999997 | 1 | 43 |
| hsa03040 Spliceosome | 0.882692491 | -1.608389136 | 1 | 1 | 65 |
| hsa03008 Ribosome biogenesis in eukaryotes | 0.933492962 | -2.697995688 | 1 | 1 | 44 |
| hsa03013 RNA transport | 0.902985199 | -2.195454485 | 1 | 1 | 83 |
| hsa04514 Cell adhesion molecules (CAMs) | 0.949170919 | -2.153334239 | 1 | 1 | 57 |
| hsa04612 Antigen processing and presentation | 0.952883458 | -3.234270584 | 1 | 1 | 52 |
| hsa04650 Natural killer cell mediated cytotoxicity | 0.782493364 | -1.926989937 | 1 | 1 | 62 |
| hsa04660 T cell receptor signaling pathway | 0.928005785 | -1.862981781 | 1 | 1 | 60 |
| hsa04672 Intestinal immune network for IgA production | 0.982274511 | -2.737004834 | 1 | 1 | 29 |
| hsa00040 Pentose and glucuronate interconversions | NA | NA | NA | NA | 8 |
| hsa00053 Ascorbate and aldarate metabolism | NA | NA | NA | NA | 6 |
| hsa00061 Fatty acid biosynthesis | NA | NA | NA | NA | 2 |
| hsa00072 Synthesis and degradation of ketone bodies | NA | NA | NA | NA | 2 |
| hsa00100 Steroid biosynthesis | NA | NA | NA | NA | 6 |
| hsa00120 Primary bile acid biosynthesis | NA | NA | NA | NA | 4 |
| hsa00130 Ubiquinone and other terpenoid-quinone biosynthesis | NA | NA | NA | NA | 5 |
| hsa00140 Steroid hormone biosynthesis | NA | NA | NA | NA | 8 |
| hsa00232 Caffeine metabolism | NA | NA | NA | NA | 2 |
| hsa00250 Alanine, aspartate and glutamate metabolism | NA | NA | NA | NA | 8 |
| hsa00290 Valine, leucine and isoleucine biosynthesis | NA | NA | NA | NA | 5 |
| hsa00300 Lysine biosynthesis | NA | NA | NA | NA | 1 |
| hsa00360 Phenylalanine metabolism | NA | NA | NA | NA | 9 |
| hsa00400 Phenylalanine, tyrosine and tryptophan biosynthesis | NA | NA | NA | NA | 2 |
| hsa00430 Taurine and hypotaurine metabolism | NA | NA | NA | NA | 6 |
| hsa00450 Selenocompound metabolism | NA | NA | NA | NA | 6 |
| hsa00460 Cyanoamino acid metabolism | NA | NA | NA | NA | 4 |
| hsa00471 D-Glutamine and D-glutamate metabolism | NA | NA | NA | NA | 2 |
| hsa00472 D-Arginine and D-ornithine metabolism | NA | NA | NA | NA | 0 |
| hsa00511 Other glycan degradation | NA | NA | NA | NA | 6 |
| hsa00531 Glycosaminoglycan degradation | NA | NA | NA | NA | 5 |
| hsa00533 Glycosaminoglycan biosynthesis - keratan sulfate | NA | NA | NA | NA | 6 |
| hsa00591 Linoleic acid metabolism | NA | NA | NA | NA | 8 |
| hsa00592 alpha-Linolenic acid metabolism | NA | NA | NA | NA | 6 |
| hsa00601 Glycosphingolipid biosynthesis - lacto and neolacto series | NA | NA | NA | NA | 9 |
| hsa00603 Glycosphingolipid biosynthesis - globo series | NA | NA | NA | NA | 6 |
| hsa00604 Glycosphingolipid biosynthesis - ganglio series | NA | NA | NA | NA | 8 |
| hsa00630 Glyoxylate and dicarboxylate metabolism | NA | NA | NA | NA | 7 |
| hsa00650 Butanoate metabolism | NA | NA | NA | NA | 8 |
| hsa00730 Thiamine metabolism | NA | NA | NA | NA | 1 |
| hsa00740 Riboflavin metabolism | NA | NA | NA | NA | 4 |
| hsa00750 Vitamin B6 metabolism | NA | NA | NA | NA | 4 |
| hsa00770 Pantothenate and CoA biosynthesis | NA | NA | NA | NA | 9 |
| hsa00780 Biotin metabolism | NA | NA | NA | NA | 1 |
| hsa00785 Lipoic acid metabolism | NA | NA | NA | NA | 1 |
| hsa00790 Folate biosynthesis | NA | NA | NA | NA | 6 |
| hsa00860 Porphyrin and chlorophyll metabolism | NA | NA | NA | NA | 8 |
| hsa00900 Terpenoid backbone biosynthesis | NA | NA | NA | NA | 6 |
| hsa00910 Nitrogen metabolism | NA | NA | NA | NA | 7 |
| hsa00920 Sulfur metabolism | NA | NA | NA | NA | 5 |
| hsa01040 Biosynthesis of unsaturated fatty acids | NA | NA | NA | NA | 5 |
| hsa03020 RNA polymerase | NA | NA | NA | NA | 7 |
| hsa03450 Non-homologous end-joining | NA | NA | NA | NA | 7 |
| hsa04122 Sulfur relay system | NA | NA | NA | NA | 4 |
| hsa04140 Regulation of autophagy | NA | NA | NA | NA | 9 |
| hsa04320 Dorso-ventral axis formation | NA | NA | NA | NA | 9 |
| hsa04614 Renin-angiotensin system | NA | NA | NA | NA | 7 |
| hsa04710 Circadian rhythm - mammal | NA | NA | NA | NA | 8 |
| hsa04744 Phototransduction | NA | NA | NA | NA | 7 |
| hsa04964 Proximal tubule bicarbonate reclamation | NA | NA | NA | NA | 8 |
| hsa04977 Vitamin digestion and absorption | NA | NA | NA | NA | 6 |
| hsa04612 Antigen processing and presentation | 0.000523855 | -3.234270584 | 2.90E-55 | 3.66E-53 | 52 |
| hsa04672 Intestinal immune network for IgA production | 0.003608133 | -2.737004834 | 1.45E-39 | 9.14E-38 | 29 |
| hsa03008 Ribosome biogenesis in eukaryotes | 0.002848282 | -2.697995688 | 7.18E-39 | 3.01E-37 | 44 |
| hsa03013 RNA transport | 0.008861591 | -2.195454485 | 1.67E-27 | 5.26E-26 | 83 |
| hsa04514 Cell adhesion molecules (CAMs) | 0.013269218 | -2.153334239 | 1.64E-26 | 4.13E-25 | 57 |
| hsa04650 Natural killer cell mediated cytotoxicity | 0.016188012 | -1.926989937 | 1.29E-21 | 2.71E-20 | 62 |
| hsa04660 T cell receptor signaling pathway | 0.027671058 | -1.862981781 | 1.71E-20 | 3.08E-19 | 60 |
| hsa03040 Spliceosome | 0.043947735 | -1.608389136 | 1.03E-15 | 1.62E-14 | 65 |
| hsa03015 mRNA surveillance pathway | 0.115837243 | -1.17004162 | 3.47E-09 | 4.86E-08 | 43 |
| hsa03010 Ribosome | 0.07335328 | -1.103220314 | 3.33E-08 | 4.19E-07 | 50 |
| hsa04310 Wnt signaling pathway | 0.1326302 | -1.075144023 | 4.54E-08 | 5.20E-07 | 62 |
| hsa03410 Base excision repair | 0.131498851 | -1.079736532 | 7.90E-08 | 8.26E-07 | 15 |
| hsa00970 Aminoacyl-tRNA biosynthesis | 0.139709149 | -1.07549933 | 8.52E-08 | 8.26E-07 | 15 |
| hsa00410 beta-Alanine metabolism | 0.14997983 | -0.98251582 | 1.18E-06 | 1.07E-05 | 11 |
| hsa00280 Valine, leucine and isoleucine degradation | 0.149152517 | -0.957904813 | 1.37E-06 | 1.15E-05 | 21 |
| hsa03018 RNA degradation | 0.153556353 | -0.916010176 | 2.97E-06 | 2.34E-05 | 40 |
| hsa00020 Citrate cycle (TCA cycle) | 0.152560813 | -0.929314515 | 3.31E-06 | 2.46E-05 | 17 |
| hsa04662 B cell receptor signaling pathway | 0.164107074 | -0.898786315 | 4.16E-06 | 2.91E-05 | 44 |
| hsa04640 Hematopoietic cell lineage | 0.170597846 | -0.849686685 | 1.22E-05 | 8.11E-05 | 58 |
| hsa03030 DNA replication | 0.171875001 | -0.855610501 | 1.57E-05 | 9.89E-05 | 17 |
| hsa04062 Chemokine signaling pathway | 0.173611772 | -0.831323797 | 1.76E-05 | 0.000105764 | 76 |
| hsa00563 Glycosylphosphatidylinositol(GPI)-anchor biosynthesis | 0.197394403 | -0.827410274 | 3.39E-05 | 0.000193981 | 10 |
| hsa00510 N-Glycan biosynthesis | 0.194260047 | -0.804923087 | 3.59E-05 | 0.000196544 | 24 |
| hsa04070 Phosphatidylinositol signaling system | 0.205451724 | -0.78148155 | 5.38E-05 | 0.00028243 | 34 |
| hsa00562 Inositol phosphate metabolism | 0.253258602 | -0.639779258 | 0.000768051 | 0.003870975 | 26 |
| hsa03430 Mismatch repair | 0.252554997 | -0.630980814 | 0.001091512 | 0.005289635 | 11 |
| hsa04340 Hedgehog signaling pathway | 0.257368442 | -0.600543156 | 0.001730456 | 0.008075459 | 11 |
| hsa00270 Cysteine and methionine metabolism | 0.280645536 | -0.544775497 | 0.003614899 | 0.016267044 | 17 |
| hsa00532 Glycosaminoglycan biosynthesis - chondroitin sulfate | 0.291398593 | -0.503942878 | 0.006735393 | 0.02926412 | 13 |
| hsa04973 Carbohydrate digestion and absorption | 0.254854847 | -0.470555615 | 0.010897892 | 0.044534123 | 18 |
| hsa04916 Melanogenesis | 0.304475636 | -0.460283933 | 0.011120721 | 0.044534123 | 34 |
| hsa00310 Lysine degradation | 0.295033806 | -0.462072457 | 0.011310253 | 0.044534123 | 22 |
| hsa04960 Aldosterone-regulated sodium reabsorption | 0.324334045 | -0.416445033 | 0.021067898 | 0.080441067 | 10 |
| hsa04330 Notch signaling pathway | 0.317887841 | -0.379344691 | 0.030675703 | 0.113680548 | 24 |
| hsa04520 Adherens junction | 0.328442497 | -0.373886164 | 0.032150473 | 0.115741704 | 24 |
| hsa04350 TGF-beta signaling pathway | 0.349250592 | -0.336026743 | 0.04744568 | 0.166059879 | 35 |
| hsa03420 Nucleotide excision repair | 0.329832375 | -0.323576625 | 0.055332587 | 0.18842989 | 25 |
| hsa00620 Pyruvate metabolism | 0.342572971 | -0.310559692 | 0.062343896 | 0.206719234 | 23 |
| hsa00340 Histidine metabolism | 0.333928116 | -0.310346216 | 0.065354761 | 0.211146149 | 13 |
| hsa00640 Propanoate metabolism | 0.372682622 | -0.284453623 | 0.079902815 | 0.251693867 | 18 |
| hsa00830 Retinol metabolism | 0.364943768 | -0.281975685 | 0.082353529 | 0.253086455 | 16 |
| hsa04970 Salivary secretion | 0.367057709 | -0.253868279 | 0.10414763 | 0.311406438 | 31 |
| hsa04742 Taste transduction | 0.3658902 | -0.256586374 | 0.106273626 | 0.311406438 | 10 |
| hsa04150 mTOR signaling pathway | 0.389306737 | -0.234823538 | 0.122475172 | 0.350724356 | 21 |
| hsa04971 Gastric acid secretion | 0.388325221 | -0.226702517 | 0.130817894 | 0.366290102 | 22 |
| hsa00561 Glycerolipid metabolism | 0.394173247 | -0.21241531 | 0.146847505 | 0.394108996 | 17 |
| hsa00514 Other types of O-glycan biosynthesis | 0.393926675 | -0.213419928 | 0.147008911 | 0.394108996 | 14 |
| hsa04720 Long-term potentiation | 0.411840939 | -0.180977424 | 0.184446896 | 0.484173102 | 27 |
| hsa04962 Vasopressin-regulated water reabsorption | 0.416474537 | -0.160162354 | 0.214378577 | 0.551259198 | 17 |
| hsa00230 Purine metabolism | 0.416052114 | -0.122534621 | 0.270681853 | 0.68211827 | 68 |
| hsa00350 Tyrosine metabolism | 0.409470461 | -0.09133216 | 0.326852906 | 0.807518943 | 18 |
| hsa04974 Protein digestion and absorption | 0.403771361 | -0.055794664 | 0.392258824 | 0.950473304 | 22 |
| hsa04360 Axon guidance | 0.430196156 | -0.041949749 | 0.417233951 | 0.991914676 | 47 |
| hsa04530 Tight junction | 0.474244569 | -0.019501103 | 0.461404565 | 1 | 44 |
| hsa00071 Fatty acid metabolism | 0.472677313 | -0.012001475 | 0.476336588 | 1 | 17 |
| hsa02010 ABC transporters | 0.46215154 | 0.002992497 | 0.504546616 | 1 | 14 |
| hsa04012 ErbB signaling pathway | 0.474935018 | 0.017319915 | 0.534589924 | 1 | 35 |
| hsa03060 Protein export | 0.443777006 | 0.024222386 | 0.550415708 | 1 | 10 |
| hsa03022 Basal transcription factors | 0.474863816 | 0.033928731 | 0.567831411 | 1 | 18 |
| hsa04722 Neurotrophin signaling pathway | 0.472238096 | 0.054288695 | 0.606539801 | 1 | 57 |
| hsa04210 Apoptosis | 0.493081991 | 0.056735492 | 0.611153947 | 1 | 46 |
| hsa00670 One carbon pool by folate | 0.564890872 | 0.287184159 | 0.920373733 | 1 | 12 |
| hsa04630 Jak-STAT signaling pathway | 0.563463798 | 0.307136844 | 0.936827539 | 1 | 58 |
| hsa00534 Glycosaminoglycan biosynthesis - heparan sulfate | 0.585617582 | 0.317377344 | 0.940252089 | 1 | 13 |
| hsa04010 MAPK signaling pathway | 0.596063 | 0.325789019 | 0.948013748 | 1 | 115 |
| hsa04146 Peroxisome | 0.579722145 | 0.331725449 | 0.950221238 | 1 | 38 |
| hsa04664 Fc epsilon RI signaling pathway | 0.595183688 | 0.346755923 | 0.95755098 | 1 | 37 |
| hsa03440 Homologous recombination | 0.595740168 | 0.353219158 | 0.958463946 | 1 | 14 |
| hsa00590 Arachidonic acid metabolism | 0.573712725 | 0.351848932 | 0.959203002 | 1 | 23 |
| hsa00030 Pentose phosphate pathway | 0.608276192 | 0.372203862 | 0.965966504 | 1 | 13 |
| hsa04670 Leukocyte transendothelial migration | 0.607902507 | 0.397233084 | 0.975899658 | 1 | 44 |
| hsa04623 Cytosolic DNA-sensing pathway | 0.602867187 | 0.411779465 | 0.978469344 | 1 | 16 |
| hsa00330 Arginine and proline metabolism | 0.60728957 | 0.413545829 | 0.979593754 | 1 | 25 |
| hsa00500 Starch and sucrose metabolism | 0.531845906 | 0.438474035 | 0.983546419 | 1 | 22 |
| hsa04120 Ubiquitin mediated proteolysis | 0.550911734 | 0.430095275 | 0.983838053 | 1 | 67 |
| hsa00380 Tryptophan metabolism | 0.62017177 | 0.450467062 | 0.986463236 | 1 | 14 |
| hsa00240 Pyrimidine metabolism | 0.619162397 | 0.466165507 | 0.989694767 | 1 | 41 |
| hsa04972 Pancreatic secretion | 0.655654595 | 0.483228191 | 0.991778653 | 1 | 32 |
| hsa04144 Endocytosis | 0.623815554 | 0.495980721 | 0.993251381 | 1 | 83 |
| hsa04370 VEGF signaling pathway | 0.666834121 | 0.535029288 | 0.996052618 | 1 | 35 |
| hsa00051 Fructose and mannose metabolism | 0.678419297 | 0.556020365 | 0.996873062 | 1 | 15 |
| hsa04966 Collecting duct acid secretion | 0.704166055 | 0.607748261 | 0.998552614 | 1 | 12 |
| hsa00520 Amino sugar and nucleotide sugar metabolism | 0.656216144 | 0.606883483 | 0.998644593 | 1 | 26 |
| hsa04976 Bile secretion | 0.628433114 | 0.613242698 | 0.998729556 | 1 | 24 |
| hsa04141 Protein processing in endoplasmic reticulum | 0.644324968 | 0.64753844 | 0.999358861 | 1 | 73 |
| hsa00052 Galactose metabolism | 0.707882858 | 0.658613474 | 0.999396769 | 1 | 16 |
| hsa04260 Cardiac muscle contraction | 0.71065169 | 0.664122781 | 0.9994887 | 1 | 26 |
| hsa04622 RIG-I-like receptor signaling pathway | 0.692420987 | 0.66978891 | 0.999503499 | 1 | 19 |
| hsa04020 Calcium signaling pathway | 0.694595944 | 0.665419209 | 0.99953452 | 1 | 60 |
| hsa04912 GnRH signaling pathway | 0.734398781 | 0.692392594 | 0.999709969 | 1 | 38 |
| hsa04145 Phagosome | 0.675665804 | 0.700067041 | 0.999749209 | 1 | 66 |
| hsa04730 Long-term depression | 0.743032726 | 0.71919779 | 0.999810788 | 1 | 22 |
| hsa00565 Ether lipid metabolism | 0.747723017 | 0.772445822 | 0.999924269 | 1 | 15 |
| hsa00260 Glycine, serine and threonine metabolism | 0.704776342 | 0.793688228 | 0.999938919 | 1 | 13 |
| hsa04920 Adipocytokine signaling pathway | 0.755692937 | 0.77741916 | 0.999941446 | 1 | 30 |
| hsa04740 Olfactory transduction | 0.703626954 | 0.795203478 | 0.999952033 | 1 | 21 |
| hsa04910 Insulin signaling pathway | 0.750087893 | 0.814558066 | 0.999974611 | 1 | 61 |
| hsa04540 Gap junction | 0.756074517 | 0.822060927 | 0.999976746 | 1 | 33 |
| hsa04380 Osteoclast differentiation | 0.732672682 | 0.837186175 | 0.99998426 | 1 | 63 |
| hsa00010 Glycolysis / Gluconeogenesis | 0.776599182 | 0.882224723 | 0.999993698 | 1 | 32 |
| hsa04620 Toll-like receptor signaling pathway | 0.741048659 | 0.906496796 | 0.999996367 | 1 | 40 |
| hsa04130 SNARE interactions in vesicular transport | 0.756803545 | 0.927354124 | 0.999996903 | 1 | 19 |
| hsa04914 Progesterone-mediated oocyte maturation | 0.794016914 | 0.963241312 | 0.999999137 | 1 | 48 |
| hsa00600 Sphingolipid metabolism | 0.784293464 | 0.996496531 | 0.999999454 | 1 | 18 |
| hsa00983 Drug metabolism - other enzymes | 0.822685832 | 1.017635843 | 0.999999663 | 1 | 14 |
| hsa00512 Mucin type O-Glycan biosynthesis | 0.820575761 | 1.03887001 | 0.999999814 | 1 | 16 |
| hsa00982 Drug metabolism - cytochrome P450 | 0.789039557 | 1.047449477 | 0.999999837 | 1 | 18 |
| hsa04975 Fat digestion and absorption | 0.826605566 | 1.076401023 | 0.999999899 | 1 | 11 |
| hsa03050 Proteasome | 0.716775376 | 1.077954357 | 0.999999909 | 1 | 20 |
| hsa04142 Lysosome | 0.78760498 | 1.062334826 | 0.999999929 | 1 | 51 |
| hsa00760 Nicotinate and nicotinamide metabolism | 0.827350269 | 1.093036465 | 0.999999939 | 1 | 12 |
| hsa04666 Fc gamma R-mediated phagocytosis | 0.794899718 | 1.079777444 | 0.999999956 | 1 | 48 |
| hsa04621 NOD-like receptor signaling pathway | 0.821443303 | 1.131738888 | 0.999999987 | 1 | 24 |
| hsa04114 Oocyte meiosis | 0.805424104 | 1.148173946 | 0.999999994 | 1 | 57 |
| hsa04110 Cell cycle | 0.737795564 | 1.156511344 | 0.999999995 | 1 | 73 |
| hsa04270 Vascular smooth muscle contraction | 0.834560817 | 1.158940797 | 0.999999995 | 1 | 46 |
| hsa00564 Glycerophospholipid metabolism | 0.845579225 | 1.178702969 | 0.999999997 | 1 | 36 |
| hsa04115 p53 signaling pathway | 0.82909321 | 1.185104354 | 0.999999998 | 1 | 35 |
| hsa04510 Focal adhesion | 0.821324739 | 1.219373069 | 0.999999999 | 1 | 77 |
| hsa00980 Metabolism of xenobiotics by cytochrome P450 | 0.849546822 | 1.261096653 | 1 | 1 | 17 |
| hsa00480 Glutathione metabolism | 0.901997925 | 1.440816773 | 1 | 1 | 22 |
| hsa04810 Regulation of actin cytoskeleton | 0.888693247 | 1.619092984 | 1 | 1 | 87 |
| hsa00190 Oxidative phosphorylation | 0.839757082 | 1.648845137 | 1 | 1 | 55 |
| hsa03320 PPAR signaling pathway | 0.985572277 | 2.423348559 | 1 | 1 | 23 |
| hsa04512 ECM-receptor interaction | 0.889855434 | 1.752947614 | 1 | 1 | 32 |
| hsa04610 Complement and coagulation cascades | 0.975002192 | 2.557470086 | 1 | 1 | 22 |
| hsa00040 Pentose and glucuronate interconversions | NA | NA | NA | NA | 8 |
| hsa00053 Ascorbate and aldarate metabolism | NA | NA | NA | NA | 6 |
| hsa00061 Fatty acid biosynthesis | NA | NA | NA | NA | 2 |
| hsa00072 Synthesis and degradation of ketone bodies | NA | NA | NA | NA | 2 |
| hsa00100 Steroid biosynthesis | NA | NA | NA | NA | 6 |
| hsa00120 Primary bile acid biosynthesis | NA | NA | NA | NA | 4 |
| hsa00130 Ubiquinone and other terpenoid-quinone biosynthesis | NA | NA | NA | NA | 5 |
| hsa00140 Steroid hormone biosynthesis | NA | NA | NA | NA | 8 |
| hsa00232 Caffeine metabolism | NA | NA | NA | NA | 2 |
| hsa00250 Alanine, aspartate and glutamate metabolism | NA | NA | NA | NA | 8 |
| hsa00290 Valine, leucine and isoleucine biosynthesis | NA | NA | NA | NA | 5 |
| hsa00300 Lysine biosynthesis | NA | NA | NA | NA | 1 |
| hsa00360 Phenylalanine metabolism | NA | NA | NA | NA | 9 |
| hsa00400 Phenylalanine, tyrosine and tryptophan biosynthesis | NA | NA | NA | NA | 2 |
| hsa00430 Taurine and hypotaurine metabolism | NA | NA | NA | NA | 6 |
| hsa00450 Selenocompound metabolism | NA | NA | NA | NA | 6 |
| hsa00460 Cyanoamino acid metabolism | NA | NA | NA | NA | 4 |
| hsa00471 D-Glutamine and D-glutamate metabolism | NA | NA | NA | NA | 2 |
| hsa00472 D-Arginine and D-ornithine metabolism | NA | NA | NA | NA | 0 |
| hsa00511 Other glycan degradation | NA | NA | NA | NA | 6 |
| hsa00531 Glycosaminoglycan degradation | NA | NA | NA | NA | 5 |
| hsa00533 Glycosaminoglycan biosynthesis - keratan sulfate | NA | NA | NA | NA | 6 |
| hsa00591 Linoleic acid metabolism | NA | NA | NA | NA | 8 |
| hsa00592 alpha-Linolenic acid metabolism | NA | NA | NA | NA | 6 |
| hsa00601 Glycosphingolipid biosynthesis - lacto and neolacto series | NA | NA | NA | NA | 9 |
| hsa00603 Glycosphingolipid biosynthesis - globo series | NA | NA | NA | NA | 6 |
| hsa00604 Glycosphingolipid biosynthesis - ganglio series | NA | NA | NA | NA | 8 |
| hsa00630 Glyoxylate and dicarboxylate metabolism | NA | NA | NA | NA | 7 |
| hsa00650 Butanoate metabolism | NA | NA | NA | NA | 8 |
| hsa00730 Thiamine metabolism | NA | NA | NA | NA | 1 |
| hsa00740 Riboflavin metabolism | NA | NA | NA | NA | 4 |
| hsa00750 Vitamin B6 metabolism | NA | NA | NA | NA | 4 |
| hsa00770 Pantothenate and CoA biosynthesis | NA | NA | NA | NA | 9 |
| hsa00780 Biotin metabolism | NA | NA | NA | NA | 1 |
| hsa00785 Lipoic acid metabolism | NA | NA | NA | NA | 1 |
| hsa00790 Folate biosynthesis | NA | NA | NA | NA | 6 |
| hsa00860 Porphyrin and chlorophyll metabolism | NA | NA | NA | NA | 8 |
| hsa00900 Terpenoid backbone biosynthesis | NA | NA | NA | NA | 6 |
| hsa00910 Nitrogen metabolism | NA | NA | NA | NA | 7 |
| hsa00920 Sulfur metabolism | NA | NA | NA | NA | 5 |
| hsa01040 Biosynthesis of unsaturated fatty acids | NA | NA | NA | NA | 5 |
| hsa03020 RNA polymerase | NA | NA | NA | NA | 7 |
| hsa03450 Non-homologous end-joining | NA | NA | NA | NA | 7 |
| hsa04122 Sulfur relay system | NA | NA | NA | NA | 4 |
| hsa04140 Regulation of autophagy | NA | NA | NA | NA | 9 |
| hsa04320 Dorso-ventral axis formation | NA | NA | NA | NA | 9 |
| hsa04614 Renin-angiotensin system | NA | NA | NA | NA | 7 |
| hsa04710 Circadian rhythm - mammal | NA | NA | NA | NA | 8 |
| hsa04744 Phototransduction | NA | NA | NA | NA | 7 |
| hsa04964 Proximal tubule bicarbonate reclamation | NA | NA | NA | NA | 8 |
| hsa04977 Vitamin digestion and absorption | NA | NA | NA | NA | 6 |
|  |  |  |  |  |  |
| **GSE95233** | p.geomean | stat.mean | p.val | q.val | set.size |
| hsa00190 Oxidative phosphorylation | 0.002405527 | 2.799196786 | 5.20E-38 | 6.45E-36 | 63 |
| hsa03320 PPAR signaling pathway | 0.023874351 | 2.041525896 | 3.13E-20 | 1.94E-18 | 17 |
| hsa04610 Complement and coagulation cascades | 0.027170072 | 1.925687416 | 1.70E-18 | 7.01E-17 | 20 |
| hsa00600 Sphingolipid metabolism | 0.043531155 | 1.739475885 | 1.97E-15 | 6.09E-14 | 16 |
| hsa04115 p53 signaling pathway | 0.059317356 | 1.532608268 | 8.32E-13 | 2.06E-11 | 30 |
| hsa04260 Cardiac muscle contraction | 0.064059077 | 1.478909164 | 4.89E-12 | 1.01E-10 | 30 |
| hsa00480 Glutathione metabolism | 0.074180722 | 1.419136235 | 4.36E-11 | 7.72E-10 | 20 |
| hsa04621 NOD-like receptor signaling pathway | 0.082022662 | 1.347229837 | 2.47E-10 | 3.71E-09 | 31 |
| hsa04114 Oocyte meiosis | 0.0802321 | 1.336928384 | 2.69E-10 | 3.71E-09 | 49 |
| hsa00770 Pantothenate and CoA biosynthesis | 0.09051072 | 1.367442579 | 4.36E-10 | 5.40E-09 | 10 |
| hsa04110 Cell cycle | 0.077352396 | 1.293514089 | 9.25E-10 | 1.04E-08 | 64 |
| hsa04130 SNARE interactions in vesicular transport | 0.120251197 | 1.166149092 | 4.17E-08 | 4.31E-07 | 23 |
| hsa04966 Collecting duct acid secretion | 0.121037232 | 1.181083 | 5.34E-08 | 5.09E-07 | 10 |
| hsa00564 Glycerophospholipid metabolism | 0.11804511 | 1.137647858 | 6.87E-08 | 6.08E-07 | 31 |
| hsa00512 Mucin type O-Glycan biosynthesis | 0.12296791 | 1.128623959 | 1.37E-07 | 1.13E-06 | 15 |
| hsa04142 Lysosome | 0.147791752 | 0.96042121 | 3.92E-06 | 3.04E-05 | 52 |
| hsa03010 Ribosome | 0.101761727 | 0.952292575 | 5.58E-06 | 4.07E-05 | 57 |
| hsa03050 Proteasome | 0.155425607 | 0.916588863 | 1.18E-05 | 8.13E-05 | 28 |
| hsa04810 Regulation of actin cytoskeleton | 0.153795464 | 0.902471843 | 1.28E-05 | 8.34E-05 | 74 |
| hsa00760 Nicotinate and nicotinamide metabolism | 0.172569009 | 0.918897089 | 1.65E-05 | 0.000102243 | 11 |
| hsa00500 Starch and sucrose metabolism | 0.176279294 | 0.896219269 | 2.13E-05 | 0.00012579 | 13 |
| hsa04622 RIG-I-like receptor signaling pathway | 0.18326514 | 0.832021411 | 5.99E-05 | 0.000337554 | 25 |
| hsa04914 Progesterone-mediated oocyte maturation | 0.187167838 | 0.820907802 | 6.61E-05 | 0.000355288 | 44 |
| hsa04540 Gap junction | 0.190249119 | 0.822086341 | 6.88E-05 | 0.000355288 | 27 |
| hsa04512 ECM-receptor interaction | 0.198250317 | 0.776777836 | 0.000184832 | 0.000916769 | 16 |
| hsa04620 Toll-like receptor signaling pathway | 0.16949466 | 0.757073757 | 0.000220714 | 0.001052638 | 43 |
| hsa00983 Drug metabolism - other enzymes | 0.225893716 | 0.733123885 | 0.000357773 | 0.001643104 | 16 |
| hsa00010 Glycolysis / Gluconeogenesis | 0.223996747 | 0.71951314 | 0.000418399 | 0.001852911 | 28 |
| hsa00565 Ether lipid metabolism | 0.229568047 | 0.699884077 | 0.000680638 | 0.002910313 | 12 |
| hsa04141 Protein processing in endoplasmic reticulum | 0.226173816 | 0.681900788 | 0.000719969 | 0.002975871 | 77 |
| hsa04666 Fc gamma R-mediated phagocytosis | 0.229237667 | 0.65880283 | 0.001072398 | 0.004289592 | 45 |
| hsa03060 Protein export | 0.249549271 | 0.640082208 | 0.00175819 | 0.006812988 | 12 |
| hsa04510 Focal adhesion | 0.260932042 | 0.562041878 | 0.004324374 | 0.015891748 | 65 |
| hsa04910 Insulin signaling pathway | 0.24335982 | 0.561929824 | 0.004416662 | 0.015891748 | 53 |
| hsa04730 Long-term depression | 0.271049583 | 0.564432997 | 0.004485574 | 0.015891748 | 19 |
| hsa00240 Pyrimidine metabolism | 0.267918513 | 0.549812329 | 0.005175318 | 0.017826094 | 46 |
| hsa04912 GnRH signaling pathway | 0.280525461 | 0.546837186 | 0.005397301 | 0.018088251 | 35 |
| hsa00052 Galactose metabolism | 0.290773336 | 0.536632657 | 0.006730104 | 0.021509737 | 12 |
| hsa04120 Ubiquitin mediated proteolysis | 0.268014629 | 0.528831177 | 0.006765159 | 0.021509737 | 62 |
| hsa03022 Basal transcription factors | 0.30664421 | 0.476814175 | 0.01358191 | 0.042103921 | 19 |
| hsa00520 Amino sugar and nucleotide sugar metabolism | 0.304550057 | 0.469809265 | 0.014674645 | 0.044381854 | 20 |
| hsa04270 Vascular smooth muscle contraction | 0.292864477 | 0.459014684 | 0.016213707 | 0.04786904 | 40 |
| hsa04140 Regulation of autophagy | 0.304982413 | 0.463150401 | 0.01724683 | 0.049735045 | 10 |
| hsa04920 Adipocytokine signaling pathway | 0.31954882 | 0.419574248 | 0.025854521 | 0.07286274 | 19 |
| hsa00982 Drug metabolism - cytochrome P450 | 0.32054652 | 0.375061263 | 0.042630916 | 0.117471858 | 15 |
| hsa04145 Phagosome | 0.305152604 | 0.355111418 | 0.048713175 | 0.131313776 | 63 |
| hsa04976 Bile secretion | 0.361439108 | 0.292586915 | 0.087524971 | 0.230916944 | 19 |
| hsa00410 beta-Alanine metabolism | 0.371970908 | 0.285064325 | 0.094677734 | 0.244584147 | 10 |
| hsa00670 One carbon pool by folate | 0.364183609 | 0.262694129 | 0.114236267 | 0.289087696 | 10 |
| hsa04623 Cytosolic DNA-sensing pathway | 0.366978383 | 0.254134941 | 0.119304662 | 0.293481185 | 23 |
| hsa00590 Arachidonic acid metabolism | 0.378843844 | 0.25495875 | 0.120705971 | 0.293481185 | 11 |
| hsa00051 Fructose and mannose metabolism | 0.408307276 | 0.181185009 | 0.200480688 | 0.46459696 | 17 |
| hsa00140 Steroid hormone biosynthesis | 0.392742352 | 0.182230976 | 0.201691477 | 0.46459696 | 11 |
| hsa04144 Endocytosis | 0.385298326 | 0.178315479 | 0.202324483 | 0.46459696 | 80 |
| hsa00330 Arginine and proline metabolism | 0.400007749 | 0.174296707 | 0.209125745 | 0.471483497 | 23 |
| hsa04380 Osteoclast differentiation | 0.370299683 | 0.164313504 | 0.221069318 | 0.489510633 | 68 |
| hsa00030 Pentose phosphate pathway | 0.432383801 | 0.144500958 | 0.252945098 | 0.550266529 | 10 |
| hsa04146 Peroxisome | 0.429049467 | 0.130951373 | 0.270787726 | 0.578048747 | 31 |
| hsa04010 MAPK signaling pathway | 0.399691392 | 0.12767921 | 0.275039323 | 0.578048747 | 100 |
| hsa03420 Nucleotide excision repair | 0.421770752 | 0.111723024 | 0.301650743 | 0.623411536 | 26 |
| hsa03430 Mismatch repair | 0.425176367 | 0.105504754 | 0.314511617 | 0.639335091 | 11 |
| hsa04974 Protein digestion and absorption | 0.409096433 | 0.086254184 | 0.345501396 | 0.680383033 | 17 |
| hsa04664 Fc epsilon RI signaling pathway | 0.430407946 | 0.085270771 | 0.345678477 | 0.680383033 | 30 |
| hsa04972 Pancreatic secretion | 0.451248965 | 0.060689464 | 0.389054944 | 0.753793954 | 22 |
| hsa04962 Vasopressin-regulated water reabsorption | 0.471824615 | 0.03268503 | 0.439642548 | 0.838702707 | 19 |
| hsa04740 Olfactory transduction | 0.461171799 | 0.00897602 | 0.483279626 | 0.907979902 | 13 |
| hsa04960 Aldosterone-regulated sodium reabsorption | 0.476469895 | 0.001433239 | 0.496890405 | 0.919618063 | 14 |
| hsa03015 mRNA surveillance pathway | 0.483397082 | -0.01291453 | 0.523870469 | 0.949097087 | 28 |
| hsa00980 Metabolism of xenobiotics by cytochrome P450 | 0.46091798 | -0.017370575 | 0.532495006 | 0.949097087 | 15 |
| hsa04150 mTOR signaling pathway | 0.47292885 | -0.019289802 | 0.535780613 | 0.949097087 | 23 |
| hsa04340 Hedgehog signaling pathway | 0.462662989 | -0.031613012 | 0.557810197 | 0.970953798 | 14 |
| hsa00620 Pyruvate metabolism | 0.500093423 | -0.037140756 | 0.568413811 | 0.970953798 | 16 |
| hsa00640 Propanoate metabolism | 0.493372052 | -0.039010701 | 0.571609897 | 0.970953798 | 14 |
| hsa04330 Notch signaling pathway | 0.486978011 | -0.045831183 | 0.583663284 | 0.971335763 | 16 |
| hsa03018 RNA degradation | 0.491614107 | -0.047445635 | 0.587501469 | 0.971335763 | 31 |
| hsa00020 Citrate cycle (TCA cycle) | 0.505988796 | -0.05475885 | 0.600121557 | 0.977247166 | 17 |
| hsa00071 Fatty acid metabolism | 0.504199231 | -0.058851455 | 0.606838966 | 0.977247166 | 16 |
| hsa04020 Calcium signaling pathway | 0.516302166 | -0.153175556 | 0.762847733 | 1 | 54 |
| hsa00380 Tryptophan metabolism | 0.518422045 | -0.175853273 | 0.788310377 | 1 | 12 |
| hsa04360 Axon guidance | 0.509780843 | -0.18935995 | 0.811219373 | 1 | 41 |
| hsa04210 Apoptosis | 0.536704845 | -0.190111157 | 0.812507383 | 1 | 49 |
| hsa04973 Carbohydrate digestion and absorption | 0.546063843 | -0.196097416 | 0.817571972 | 1 | 14 |
| hsa04971 Gastric acid secretion | 0.54938446 | -0.211472955 | 0.836938788 | 1 | 24 |
| hsa04370 VEGF signaling pathway | 0.550486729 | -0.212760948 | 0.839042436 | 1 | 31 |
| hsa04720 Long-term potentiation | 0.557502193 | -0.255018734 | 0.881460693 | 1 | 29 |
| hsa03030 DNA replication | 0.539718677 | -0.280035079 | 0.902772456 | 1 | 19 |
| hsa03040 Spliceosome | 0.565471284 | -0.314034859 | 0.928633923 | 1 | 62 |
| hsa00561 Glycerolipid metabolism | 0.604228446 | -0.34748943 | 0.945763894 | 1 | 16 |
| hsa04670 Leukocyte transendothelial migration | 0.594623185 | -0.3498243 | 0.948501202 | 1 | 41 |
| hsa00230 Purine metabolism | 0.60064389 | -0.398751409 | 0.968850921 | 1 | 67 |
| hsa00280 Valine, leucine and isoleucine degradation | 0.61161551 | -0.405330006 | 0.969706615 | 1 | 22 |
| hsa03020 RNA polymerase | 0.627935963 | -0.420896642 | 0.973794645 | 1 | 15 |
| hsa00563 Glycosylphosphatidylinositol(GPI)-anchor biosynthesis | 0.629197644 | -0.425756424 | 0.973819205 | 1 | 10 |
| hsa04916 Melanogenesis | 0.62085355 | -0.424410724 | 0.97578663 | 1 | 31 |
| hsa00270 Cysteine and methionine metabolism | 0.64261013 | -0.435500686 | 0.978174685 | 1 | 18 |
| hsa04970 Salivary secretion | 0.655957267 | -0.481615545 | 0.987497686 | 1 | 29 |
| hsa00514 Other types of O-glycan biosynthesis | 0.666225585 | -0.517012131 | 0.991443934 | 1 | 14 |
| hsa04662 B cell receptor signaling pathway | 0.640081314 | -0.517485781 | 0.991968065 | 1 | 41 |
| hsa03410 Base excision repair | 0.653636769 | -0.526089022 | 0.992113123 | 1 | 14 |
| hsa00310 Lysine degradation | 0.66470473 | -0.528728928 | 0.992689874 | 1 | 17 |
| hsa04350 TGF-beta signaling pathway | 0.695985624 | -0.566944806 | 0.99582273 | 1 | 26 |
| hsa00510 N-Glycan biosynthesis | 0.668029074 | -0.573956449 | 0.996087483 | 1 | 22 |
| hsa00830 Retinol metabolism | 0.70030628 | -0.612940663 | 0.997594785 | 1 | 13 |
| hsa04710 Circadian rhythm - mammal | 0.707529521 | -0.62694447 | 0.997934098 | 1 | 10 |
| hsa04520 Adherens junction | 0.70653717 | -0.627616024 | 0.998206007 | 1 | 22 |
| hsa04722 Neurotrophin signaling pathway | 0.677203681 | -0.643469671 | 0.998638926 | 1 | 50 |
| hsa00532 Glycosaminoglycan biosynthesis - chondroitin sulfate | 0.724090055 | -0.693820692 | 0.999216523 | 1 | 10 |
| hsa04012 ErbB signaling pathway | 0.713986543 | -0.715104655 | 0.999546754 | 1 | 30 |
| hsa00562 Inositol phosphate metabolism | 0.757191001 | -0.786642798 | 0.999872327 | 1 | 31 |
| hsa04742 Taste transduction | 0.802124012 | -0.968677766 | 0.999994491 | 1 | 11 |
| hsa04630 Jak-STAT signaling pathway | 0.788789658 | -0.94752164 | 0.999995032 | 1 | 60 |
| hsa04070 Phosphatidylinositol signaling system | 0.811839185 | -0.962674659 | 0.999996316 | 1 | 42 |
| hsa04530 Tight junction | 0.802053756 | -1.008323496 | 0.99999856 | 1 | 34 |
| hsa04310 Wnt signaling pathway | 0.816982134 | -1.034466898 | 0.999999277 | 1 | 49 |
| hsa00970 Aminoacyl-tRNA biosynthesis | 0.825350021 | -1.099066804 | 0.999999584 | 1 | 10 |
| hsa04062 Chemokine signaling pathway | 0.808304824 | -1.110360608 | 0.999999888 | 1 | 76 |
| hsa04640 Hematopoietic cell lineage | 0.889669695 | -1.499443554 | 1 | 1 | 46 |
| hsa03013 RNA transport | 0.899836378 | -1.557272524 | 1 | 1 | 61 |
| hsa03008 Ribosome biogenesis in eukaryotes | 0.889365877 | -1.642285908 | 1 | 1 | 37 |
| hsa04514 Cell adhesion molecules (CAMs) | 0.99642481 | -3.492975858 | 1 | 1 | 56 |
| hsa04612 Antigen processing and presentation | 0.996742098 | -4.19632946 | 1 | 1 | 48 |
| hsa04650 Natural killer cell mediated cytotoxicity | 0.956887957 | -2.589426258 | 1 | 1 | 69 |
| hsa04660 T cell receptor signaling pathway | 0.984081578 | -2.447563982 | 1 | 1 | 57 |
| hsa04672 Intestinal immune network for IgA production | 0.986454584 | -2.868405959 | 1 | 1 | 25 |
| hsa00040 Pentose and glucuronate interconversions | NA | NA | NA | NA | 5 |
| hsa00053 Ascorbate and aldarate metabolism | NA | NA | NA | NA | 4 |
| hsa00061 Fatty acid biosynthesis | NA | NA | NA | NA | 1 |
| hsa00072 Synthesis and degradation of ketone bodies | NA | NA | NA | NA | 3 |
| hsa00100 Steroid biosynthesis | NA | NA | NA | NA | 8 |
| hsa00120 Primary bile acid biosynthesis | NA | NA | NA | NA | 2 |
| hsa00130 Ubiquinone and other terpenoid-quinone biosynthesis | NA | NA | NA | NA | 5 |
| hsa00232 Caffeine metabolism | NA | NA | NA | NA | 3 |
| hsa00250 Alanine, aspartate and glutamate metabolism | NA | NA | NA | NA | 8 |
| hsa00260 Glycine, serine and threonine metabolism | NA | NA | NA | NA | 9 |
| hsa00290 Valine, leucine and isoleucine biosynthesis | NA | NA | NA | NA | 3 |
| hsa00300 Lysine biosynthesis | NA | NA | NA | NA | 1 |
| hsa00340 Histidine metabolism | NA | NA | NA | NA | 7 |
| hsa00350 Tyrosine metabolism | NA | NA | NA | NA | 8 |
| hsa00360 Phenylalanine metabolism | NA | NA | NA | NA | 5 |
| hsa00400 Phenylalanine, tyrosine and tryptophan biosynthesis | NA | NA | NA | NA | 2 |
| hsa00430 Taurine and hypotaurine metabolism | NA | NA | NA | NA | 1 |
| hsa00450 Selenocompound metabolism | NA | NA | NA | NA | 4 |
| hsa00460 Cyanoamino acid metabolism | NA | NA | NA | NA | 3 |
| hsa00471 D-Glutamine and D-glutamate metabolism | NA | NA | NA | NA | 2 |
| hsa00472 D-Arginine and D-ornithine metabolism | NA | NA | NA | NA | 0 |
| hsa00511 Other glycan degradation | NA | NA | NA | NA | 7 |
| hsa00531 Glycosaminoglycan degradation | NA | NA | NA | NA | 7 |
| hsa00533 Glycosaminoglycan biosynthesis - keratan sulfate | NA | NA | NA | NA | 6 |
| hsa00534 Glycosaminoglycan biosynthesis - heparan sulfate | NA | NA | NA | NA | 8 |
| hsa00591 Linoleic acid metabolism | NA | NA | NA | NA | 6 |
| hsa00592 alpha-Linolenic acid metabolism | NA | NA | NA | NA | 4 |
| hsa00601 Glycosphingolipid biosynthesis - lacto and neolacto series | NA | NA | NA | NA | 9 |
| hsa00603 Glycosphingolipid biosynthesis - globo series | NA | NA | NA | NA | 6 |
| hsa00604 Glycosphingolipid biosynthesis - ganglio series | NA | NA | NA | NA | 8 |
| hsa00630 Glyoxylate and dicarboxylate metabolism | NA | NA | NA | NA | 9 |
| hsa00650 Butanoate metabolism | NA | NA | NA | NA | 5 |
| hsa00730 Thiamine metabolism | NA | NA | NA | NA | 1 |
| hsa00740 Riboflavin metabolism | NA | NA | NA | NA | 3 |
| hsa00750 Vitamin B6 metabolism | NA | NA | NA | NA | 2 |
| hsa00780 Biotin metabolism | NA | NA | NA | NA | 0 |
| hsa00785 Lipoic acid metabolism | NA | NA | NA | NA | 1 |
| hsa00790 Folate biosynthesis | NA | NA | NA | NA | 4 |
| hsa00860 Porphyrin and chlorophyll metabolism | NA | NA | NA | NA | 6 |
| hsa00900 Terpenoid backbone biosynthesis | NA | NA | NA | NA | 7 |
| hsa00910 Nitrogen metabolism | NA | NA | NA | NA | 7 |
| hsa00920 Sulfur metabolism | NA | NA | NA | NA | 4 |
| hsa01040 Biosynthesis of unsaturated fatty acids | NA | NA | NA | NA | 7 |
| hsa02010 ABC transporters | NA | NA | NA | NA | 9 |
| hsa03440 Homologous recombination | NA | NA | NA | NA | 8 |
| hsa03450 Non-homologous end-joining | NA | NA | NA | NA | 7 |
| hsa04122 Sulfur relay system | NA | NA | NA | NA | 2 |
| hsa04320 Dorso-ventral axis formation | NA | NA | NA | NA | 7 |
| hsa04614 Renin-angiotensin system | NA | NA | NA | NA | 5 |
| hsa04744 Phototransduction | NA | NA | NA | NA | 8 |
| hsa04964 Proximal tubule bicarbonate reclamation | NA | NA | NA | NA | 7 |
| hsa04975 Fat digestion and absorption | NA | NA | NA | NA | 9 |
| hsa04977 Vitamin digestion and absorption | NA | NA | NA | NA | 7 |
| hsa04612 Antigen processing and presentation | 2.27E-05 | -4.19632946 | 7.01E-78 | 8.69E-76 | 48 |
| hsa04514 Cell adhesion molecules (CAMs) | 0.000275167 | -3.492975858 | 7.12E-57 | 4.41E-55 | 56 |
| hsa04672 Intestinal immune network for IgA production | 0.002645237 | -2.868405959 | 1.68E-37 | 6.93E-36 | 25 |
| hsa04650 Natural killer cell mediated cytotoxicity | 0.003747811 | -2.589426258 | 4.69E-33 | 1.45E-31 | 69 |
| hsa04660 T cell receptor signaling pathway | 0.007193874 | -2.447563982 | 8.09E-30 | 2.01E-28 | 57 |
| hsa03008 Ribosome biogenesis in eukaryotes | 0.043118563 | -1.642285908 | 2.12E-14 | 4.38E-13 | 37 |
| hsa03013 RNA transport | 0.053971972 | -1.557272524 | 2.42E-13 | 4.30E-12 | 61 |
| hsa04640 Hematopoietic cell lineage | 0.058596705 | -1.499443554 | 1.99E-12 | 3.08E-11 | 46 |
| hsa04062 Chemokine signaling pathway | 0.114471919 | -1.110360608 | 1.12E-07 | 1.55E-06 | 76 |
| hsa00970 Aminoacyl-tRNA biosynthesis | 0.136777386 | -1.099066804 | 4.16E-07 | 5.16E-06 | 10 |
| hsa04310 Wnt signaling pathway | 0.140968001 | -1.034466898 | 7.23E-07 | 8.15E-06 | 49 |
| hsa04530 Tight junction | 0.144967977 | -1.008323496 | 1.44E-06 | 1.49E-05 | 34 |
| hsa04070 Phosphatidylinositol signaling system | 0.161567494 | -0.962674659 | 3.68E-06 | 3.51E-05 | 42 |
| hsa04630 Jak-STAT signaling pathway | 0.156512192 | -0.94752164 | 4.97E-06 | 4.40E-05 | 60 |
| hsa04742 Taste transduction | 0.164815459 | -0.968677766 | 5.51E-06 | 4.55E-05 | 11 |
| hsa00562 Inositol phosphate metabolism | 0.20563815 | -0.786642798 | 0.000127673 | 0.000989466 | 31 |
| hsa04012 ErbB signaling pathway | 0.215333087 | -0.715104655 | 0.000453246 | 0.003306029 | 30 |
| hsa00532 Glycosaminoglycan biosynthesis - chondroitin sulfate | 0.236119334 | -0.693820692 | 0.000783477 | 0.005397286 | 10 |
| hsa04722 Neurotrophin signaling pathway | 0.227714622 | -0.643469671 | 0.001361074 | 0.008882797 | 50 |
| hsa04520 Adherens junction | 0.253445218 | -0.627616024 | 0.001793993 | 0.011122755 | 22 |
| hsa04710 Circadian rhythm - mammal | 0.258587199 | -0.62694447 | 0.002065902 | 0.012198657 | 10 |
| hsa00830 Retinol metabolism | 0.259222479 | -0.612940663 | 0.002405215 | 0.013556667 | 13 |
| hsa00510 N-Glycan biosynthesis | 0.260816553 | -0.573956449 | 0.003912517 | 0.021093567 | 22 |
| hsa04350 TGF-beta signaling pathway | 0.276808272 | -0.566944806 | 0.00417727 | 0.021582563 | 26 |
| hsa00310 Lysine degradation | 0.280948854 | -0.528728928 | 0.007310126 | 0.036258226 | 17 |
| hsa03410 Base excision repair | 0.277480592 | -0.526089022 | 0.007886877 | 0.036887404 | 14 |
| hsa04662 B cell receptor signaling pathway | 0.267297743 | -0.517485781 | 0.008031935 | 0.036887404 | 41 |
| hsa00514 Other types of O-glycan biosynthesis | 0.288745698 | -0.517012131 | 0.008556066 | 0.037891148 | 14 |
| hsa04970 Salivary secretion | 0.298667629 | -0.481615545 | 0.012502314 | 0.053458172 | 29 |
| hsa00270 Cysteine and methionine metabolism | 0.31779388 | -0.435500686 | 0.021825315 | 0.090211301 | 18 |
| hsa04916 Melanogenesis | 0.307809028 | -0.424410724 | 0.02421337 | 0.096853478 | 31 |
| hsa00563 Glycosylphosphatidylinositol(GPI)-anchor biosynthesis | 0.318205481 | -0.425756424 | 0.026180795 | 0.098468607 | 10 |
| hsa03020 RNA polymerase | 0.318128709 | -0.420896642 | 0.026205355 | 0.098468607 | 15 |
| hsa00280 Valine, leucine and isoleucine degradation | 0.313740567 | -0.405330006 | 0.030293385 | 0.110356737 | 22 |
| hsa00230 Purine metabolism | 0.312231752 | -0.398751409 | 0.031149079 | 0.110356737 | 67 |
| hsa04670 Leukocyte transendothelial migration | 0.333252451 | -0.3498243 | 0.051498798 | 0.17738475 | 41 |
| hsa00561 Glycerolipid metabolism | 0.344661037 | -0.34748943 | 0.054236106 | 0.181764247 | 16 |
| hsa03040 Spliceosome | 0.33695989 | -0.314034859 | 0.071366077 | 0.232878777 | 62 |
| hsa03030 DNA replication | 0.342349169 | -0.280035079 | 0.097227544 | 0.30913373 | 19 |
| hsa04720 Long-term potentiation | 0.364436535 | -0.255018734 | 0.118539307 | 0.367471851 | 29 |
| hsa04370 VEGF signaling pathway | 0.388621546 | -0.212760948 | 0.160957564 | 0.481418815 | 31 |
| hsa04971 Gastric acid secretion | 0.388819729 | -0.211472955 | 0.163061212 | 0.481418815 | 24 |
| hsa04973 Carbohydrate digestion and absorption | 0.398617215 | -0.196097416 | 0.182428028 | 0.520195506 | 14 |
| hsa04210 Apoptosis | 0.392313712 | -0.190111157 | 0.187492617 | 0.520195506 | 49 |
| hsa04360 Axon guidance | 0.372183603 | -0.18935995 | 0.188780627 | 0.520195506 | 41 |
| hsa00380 Tryptophan metabolism | 0.388325157 | -0.175853273 | 0.211689623 | 0.570641592 | 12 |
| hsa04020 Calcium signaling pathway | 0.401436606 | -0.153175556 | 0.237152267 | 0.625678321 | 54 |
| hsa00071 Fatty acid metabolism | 0.458066187 | -0.058851455 | 0.393161034 | 1 | 16 |
| hsa00020 Citrate cycle (TCA cycle) | 0.463937459 | -0.05475885 | 0.399878443 | 1 | 17 |
| hsa03018 RNA degradation | 0.455178294 | -0.047445635 | 0.412498531 | 1 | 31 |
| hsa04330 Notch signaling pathway | 0.452373772 | -0.045831183 | 0.416336716 | 1 | 16 |
| hsa00640 Propanoate metabolism | 0.46349701 | -0.039010701 | 0.428390103 | 1 | 14 |
| hsa00620 Pyruvate metabolism | 0.471219028 | -0.037140756 | 0.431586189 | 1 | 16 |
| hsa04340 Hedgehog signaling pathway | 0.440340955 | -0.031613012 | 0.442189803 | 1 | 14 |
| hsa04150 mTOR signaling pathway | 0.458495402 | -0.019289802 | 0.464219387 | 1 | 23 |
| hsa00980 Metabolism of xenobiotics by cytochrome P450 | 0.449064886 | -0.017370575 | 0.467504994 | 1 | 15 |
| hsa03015 mRNA surveillance pathway | 0.473359166 | -0.01291453 | 0.476129531 | 1 | 28 |
| hsa04960 Aldosterone-regulated sodium reabsorption | 0.477092799 | 0.001433239 | 0.503109595 | 1 | 14 |
| hsa04740 Olfactory transduction | 0.468390194 | 0.00897602 | 0.516720374 | 1 | 13 |
| hsa04962 Vasopressin-regulated water reabsorption | 0.497051442 | 0.03268503 | 0.560357452 | 1 | 19 |
| hsa04972 Pancreatic secretion | 0.498085967 | 0.060689464 | 0.610945056 | 1 | 22 |
| hsa04664 Fc epsilon RI signaling pathway | 0.494742064 | 0.085270771 | 0.654321523 | 1 | 30 |
| hsa04974 Protein digestion and absorption | 0.47183504 | 0.086254184 | 0.654498604 | 1 | 17 |
| hsa03430 Mismatch repair | 0.504461898 | 0.105504754 | 0.685488383 | 1 | 11 |
| hsa03420 Nucleotide excision repair | 0.505818768 | 0.111723024 | 0.698349257 | 1 | 26 |
| hsa04010 MAPK signaling pathway | 0.492553192 | 0.12767921 | 0.724960677 | 1 | 100 |
| hsa04146 Peroxisome | 0.530199964 | 0.130951373 | 0.729212274 | 1 | 31 |
| hsa00030 Pentose phosphate pathway | 0.543688226 | 0.144500958 | 0.747054902 | 1 | 10 |
| hsa04380 Osteoclast differentiation | 0.483918119 | 0.164313504 | 0.778930682 | 1 | 68 |
| hsa00330 Arginine and proline metabolism | 0.530166547 | 0.174296707 | 0.790874255 | 1 | 23 |
| hsa04144 Endocytosis | 0.519600384 | 0.178315479 | 0.797675517 | 1 | 80 |
| hsa00140 Steroid hormone biosynthesis | 0.526475503 | 0.182230976 | 0.798308523 | 1 | 11 |
| hsa00051 Fructose and mannose metabolism | 0.546679988 | 0.181185009 | 0.799519312 | 1 | 17 |
| hsa00590 Arachidonic acid metabolism | 0.570427709 | 0.25495875 | 0.879294029 | 1 | 11 |
| hsa04623 Cytosolic DNA-sensing pathway | 0.557675072 | 0.254134941 | 0.880695338 | 1 | 23 |
| hsa00670 One carbon pool by folate | 0.554795924 | 0.262694129 | 0.885763733 | 1 | 10 |
| hsa00410 beta-Alanine metabolism | 0.585168381 | 0.285064325 | 0.905322266 | 1 | 10 |
| hsa04976 Bile secretion | 0.580590188 | 0.292586915 | 0.912475029 | 1 | 19 |
| hsa04145 Phagosome | 0.555574469 | 0.355111418 | 0.951286825 | 1 | 63 |
| hsa00982 Drug metabolism - cytochrome P450 | 0.592457984 | 0.375061263 | 0.957369084 | 1 | 15 |
| hsa04920 Adipocytokine signaling pathway | 0.630891889 | 0.419574248 | 0.974145479 | 1 | 19 |
| hsa04140 Regulation of autophagy | 0.642203171 | 0.463150401 | 0.98275317 | 1 | 10 |
| hsa04270 Vascular smooth muscle contraction | 0.626300305 | 0.459014684 | 0.983786293 | 1 | 40 |
| hsa00520 Amino sugar and nucleotide sugar metabolism | 0.653491632 | 0.469809265 | 0.985325355 | 1 | 20 |
| hsa03022 Basal transcription factors | 0.661041852 | 0.476814175 | 0.98641809 | 1 | 19 |
| hsa04120 Ubiquitin mediated proteolysis | 0.646291348 | 0.528831177 | 0.993234841 | 1 | 62 |
| hsa00052 Galactose metabolism | 0.687462737 | 0.536632657 | 0.993269896 | 1 | 12 |
| hsa04912 GnRH signaling pathway | 0.684718997 | 0.546837186 | 0.994602699 | 1 | 35 |
| hsa00240 Pyrimidine metabolism | 0.668038916 | 0.549812329 | 0.994824682 | 1 | 46 |
| hsa04730 Long-term depression | 0.680593855 | 0.564432997 | 0.995514426 | 1 | 19 |
| hsa04910 Insulin signaling pathway | 0.633543422 | 0.561929824 | 0.995583338 | 1 | 53 |
| hsa04510 Focal adhesion | 0.664757309 | 0.562041878 | 0.995675626 | 1 | 65 |
| hsa03060 Protein export | 0.701516917 | 0.640082208 | 0.99824181 | 1 | 12 |
| hsa04666 Fc gamma R-mediated phagocytosis | 0.691801397 | 0.65880283 | 0.998927602 | 1 | 45 |
| hsa04141 Protein processing in endoplasmic reticulum | 0.707083743 | 0.681900788 | 0.999280031 | 1 | 77 |
| hsa00565 Ether lipid metabolism | 0.718036268 | 0.699884077 | 0.999319362 | 1 | 12 |
| hsa00010 Glycolysis / Gluconeogenesis | 0.735564369 | 0.71951314 | 0.999581601 | 1 | 28 |
| hsa00983 Drug metabolism - other enzymes | 0.747265109 | 0.733123885 | 0.999642227 | 1 | 16 |
| hsa04620 Toll-like receptor signaling pathway | 0.638668924 | 0.757073757 | 0.999779286 | 1 | 43 |
| hsa04512 ECM-receptor interaction | 0.719223739 | 0.776777836 | 0.999815168 | 1 | 16 |
| hsa04540 Gap junction | 0.75049247 | 0.822086341 | 0.999931235 | 1 | 27 |
| hsa04914 Progesterone-mediated oocyte maturation | 0.746126178 | 0.820907802 | 0.999933856 | 1 | 44 |
| hsa04622 RIG-I-like receptor signaling pathway | 0.742904404 | 0.832021411 | 0.999940111 | 1 | 25 |
| hsa00500 Starch and sucrose metabolism | 0.775115118 | 0.896219269 | 0.999978697 | 1 | 13 |
| hsa00760 Nicotinate and nicotinamide metabolism | 0.778282497 | 0.918897089 | 0.999983509 | 1 | 11 |
| hsa04810 Regulation of actin cytoskeleton | 0.739967183 | 0.902471843 | 0.999987218 | 1 | 74 |
| hsa03050 Proteasome | 0.750495362 | 0.916588863 | 0.9999882 | 1 | 28 |
| hsa03010 Ribosome | 0.646488497 | 0.952292575 | 0.999994417 | 1 | 57 |
| hsa04142 Lysosome | 0.776112597 | 0.96042121 | 0.999996077 | 1 | 52 |
| hsa00512 Mucin type O-Glycan biosynthesis | 0.827482899 | 1.128623959 | 0.999999863 | 1 | 15 |
| hsa00564 Glycerophospholipid metabolism | 0.829897708 | 1.137647858 | 0.999999931 | 1 | 31 |
| hsa04966 Collecting duct acid secretion | 0.850884406 | 1.181083 | 0.999999947 | 1 | 10 |
| hsa04130 SNARE interactions in vesicular transport | 0.856609981 | 1.166149092 | 0.999999958 | 1 | 23 |
| hsa04110 Cell cycle | 0.816463858 | 1.293514089 | 0.999999999 | 1 | 64 |
| hsa00770 Pantothenate and CoA biosynthesis | 0.882714031 | 1.367442579 | 1 | 1 | 10 |
| hsa04114 Oocyte meiosis | 0.861120216 | 1.336928384 | 1 | 1 | 49 |
| hsa04621 NOD-like receptor signaling pathway | 0.870466033 | 1.347229837 | 1 | 1 | 31 |
| hsa00480 Glutathione metabolism | 0.883171355 | 1.419136235 | 1 | 1 | 20 |
| hsa04260 Cardiac muscle contraction | 0.890214928 | 1.478909164 | 1 | 1 | 30 |
| hsa04115 p53 signaling pathway | 0.902428216 | 1.532608268 | 1 | 1 | 30 |
| hsa00600 Sphingolipid metabolism | 0.936396717 | 1.739475885 | 1 | 1 | 16 |
| hsa00190 Oxidative phosphorylation | 0.987908268 | 2.799196786 | 1 | 1 | 63 |
| hsa03320 PPAR signaling pathway | 0.962964986 | 2.041525896 | 1 | 1 | 17 |
| hsa04610 Complement and coagulation cascades | 0.937569232 | 1.925687416 | 1 | 1 | 20 |
| hsa00040 Pentose and glucuronate interconversions | NA | NA | NA | NA | 5 |
| hsa00053 Ascorbate and aldarate metabolism | NA | NA | NA | NA | 4 |
| hsa00061 Fatty acid biosynthesis | NA | NA | NA | NA | 1 |
| hsa00072 Synthesis and degradation of ketone bodies | NA | NA | NA | NA | 3 |
| hsa00100 Steroid biosynthesis | NA | NA | NA | NA | 8 |
| hsa00120 Primary bile acid biosynthesis | NA | NA | NA | NA | 2 |
| hsa00130 Ubiquinone and other terpenoid-quinone biosynthesis | NA | NA | NA | NA | 5 |
| hsa00232 Caffeine metabolism | NA | NA | NA | NA | 3 |
| hsa00250 Alanine, aspartate and glutamate metabolism | NA | NA | NA | NA | 8 |
| hsa00260 Glycine, serine and threonine metabolism | NA | NA | NA | NA | 9 |
| hsa00290 Valine, leucine and isoleucine biosynthesis | NA | NA | NA | NA | 3 |
| hsa00300 Lysine biosynthesis | NA | NA | NA | NA | 1 |
| hsa00340 Histidine metabolism | NA | NA | NA | NA | 7 |
| hsa00350 Tyrosine metabolism | NA | NA | NA | NA | 8 |
| hsa00360 Phenylalanine metabolism | NA | NA | NA | NA | 5 |
| hsa00400 Phenylalanine, tyrosine and tryptophan biosynthesis | NA | NA | NA | NA | 2 |
| hsa00430 Taurine and hypotaurine metabolism | NA | NA | NA | NA | 1 |
| hsa00450 Selenocompound metabolism | NA | NA | NA | NA | 4 |
| hsa00460 Cyanoamino acid metabolism | NA | NA | NA | NA | 3 |
| hsa00471 D-Glutamine and D-glutamate metabolism | NA | NA | NA | NA | 2 |
| hsa00472 D-Arginine and D-ornithine metabolism | NA | NA | NA | NA | 0 |
| hsa00511 Other glycan degradation | NA | NA | NA | NA | 7 |
| hsa00531 Glycosaminoglycan degradation | NA | NA | NA | NA | 7 |
| hsa00533 Glycosaminoglycan biosynthesis - keratan sulfate | NA | NA | NA | NA | 6 |
| hsa00534 Glycosaminoglycan biosynthesis - heparan sulfate | NA | NA | NA | NA | 8 |
| hsa00591 Linoleic acid metabolism | NA | NA | NA | NA | 6 |
| hsa00592 alpha-Linolenic acid metabolism | NA | NA | NA | NA | 4 |
| hsa00601 Glycosphingolipid biosynthesis - lacto and neolacto series | NA | NA | NA | NA | 9 |
| hsa00603 Glycosphingolipid biosynthesis - globo series | NA | NA | NA | NA | 6 |
| hsa00604 Glycosphingolipid biosynthesis - ganglio series | NA | NA | NA | NA | 8 |
| hsa00630 Glyoxylate and dicarboxylate metabolism | NA | NA | NA | NA | 9 |
| hsa00650 Butanoate metabolism | NA | NA | NA | NA | 5 |
| hsa00730 Thiamine metabolism | NA | NA | NA | NA | 1 |
| hsa00740 Riboflavin metabolism | NA | NA | NA | NA | 3 |
| hsa00750 Vitamin B6 metabolism | NA | NA | NA | NA | 2 |
| hsa00780 Biotin metabolism | NA | NA | NA | NA | 0 |
| hsa00785 Lipoic acid metabolism | NA | NA | NA | NA | 1 |
| hsa00790 Folate biosynthesis | NA | NA | NA | NA | 4 |
| hsa00860 Porphyrin and chlorophyll metabolism | NA | NA | NA | NA | 6 |
| hsa00900 Terpenoid backbone biosynthesis | NA | NA | NA | NA | 7 |
| hsa00910 Nitrogen metabolism | NA | NA | NA | NA | 7 |
| hsa00920 Sulfur metabolism | NA | NA | NA | NA | 4 |
| hsa01040 Biosynthesis of unsaturated fatty acids | NA | NA | NA | NA | 7 |
| hsa02010 ABC transporters | NA | NA | NA | NA | 9 |
| hsa03440 Homologous recombination | NA | NA | NA | NA | 8 |
| hsa03450 Non-homologous end-joining | NA | NA | NA | NA | 7 |
| hsa04122 Sulfur relay system | NA | NA | NA | NA | 2 |
| hsa04320 Dorso-ventral axis formation | NA | NA | NA | NA | 7 |
| hsa04614 Renin-angiotensin system | NA | NA | NA | NA | 5 |
| hsa04744 Phototransduction | NA | NA | NA | NA | 8 |
| hsa04964 Proximal tubule bicarbonate reclamation | NA | NA | NA | NA | 7 |
| hsa04975 Fat digestion and absorption | NA | NA | NA | NA | 9 |
| hsa04977 Vitamin digestion and absorption | NA | NA | NA | NA | 7 |
|  |  |  |  |  |  |
| **GSE64456** | p.geomean | stat.mean | p.val | q.val | set.size |
| hsa04810 Regulation of actin cytoskeleton | 0.024980698 | 1.900312857 | 1.38E-16 | 1.58E-14 | 71 |
| hsa04380 Osteoclast differentiation | 0.023553361 | 1.784762018 | 9.44E-15 | 5.38E-13 | 65 |
| hsa04670 Leukocyte transendothelial migration | 0.035758481 | 1.760501027 | 3.10E-14 | 1.18E-12 | 40 |
| hsa04666 Fc gamma R-mediated phagocytosis | 0.045133485 | 1.649307533 | 6.41E-13 | 1.83E-11 | 47 |
| hsa04650 Natural killer cell mediated cytotoxicity | 0.072225444 | 1.368939285 | 2.04E-09 | 4.64E-08 | 44 |
| hsa04664 Fc epsilon RI signaling pathway | 0.105157532 | 1.199401081 | 1.43E-07 | 2.50E-06 | 26 |
| hsa04142 Lysosome | 0.097778324 | 1.186075718 | 1.53E-07 | 2.50E-06 | 51 |
| hsa04620 Toll-like receptor signaling pathway | 0.111964363 | 1.141764999 | 4.56E-07 | 6.50E-06 | 36 |
| hsa04510 Focal adhesion | 0.125720461 | 1.109655708 | 7.59E-07 | 9.61E-06 | 64 |
| hsa04010 MAPK signaling pathway | 0.12788067 | 1.078335 | 1.42E-06 | 1.62E-05 | 97 |
| hsa00500 Starch and sucrose metabolism | 0.147212645 | 1.060429956 | 3.46E-06 | 3.59E-05 | 12 |
| hsa04062 Chemokine signaling pathway | 0.124300391 | 1.031280151 | 4.04E-06 | 3.84E-05 | 72 |
| hsa04520 Adherens junction | 0.156783987 | 0.993747894 | 9.14E-06 | 7.85E-05 | 31 |
| hsa00051 Fructose and mannose metabolism | 0.154399738 | 1.004180356 | 9.63E-06 | 7.85E-05 | 15 |
| hsa04920 Adipocytokine signaling pathway | 0.153578219 | 0.980627912 | 1.23E-05 | 9.33E-05 | 27 |
| hsa00030 Pentose phosphate pathway | 0.134763581 | 1.000753343 | 1.46E-05 | 0.000104057 | 13 |
| hsa04910 Insulin signaling pathway | 0.17037218 | 0.922536153 | 3.23E-05 | 0.000216303 | 50 |
| hsa04530 Tight junction | 0.177743253 | 0.889621278 | 5.93E-05 | 0.000375845 | 41 |
| hsa00010 Glycolysis / Gluconeogenesis | 0.165722382 | 0.881968186 | 7.36E-05 | 0.000441622 | 30 |
| hsa04210 Apoptosis | 0.180736565 | 0.849188966 | 0.000121182 | 0.00069074 | 42 |
| hsa04145 Phagosome | 0.143549486 | 0.840558448 | 0.000139443 | 0.000756978 | 72 |
| hsa04722 Neurotrophin signaling pathway | 0.200912653 | 0.798404883 | 0.000272888 | 0.001414054 | 49 |
| hsa04971 Gastric acid secretion | 0.217670806 | 0.761693351 | 0.000529168 | 0.002519363 | 21 |
| hsa04976 Bile secretion | 0.215490917 | 0.762650629 | 0.000530392 | 0.002519363 | 19 |
| hsa03320 PPAR signaling pathway | 0.198346671 | 0.750264896 | 0.000669556 | 0.002987829 | 19 |
| hsa04144 Endocytosis | 0.207710515 | 0.738460837 | 0.000681435 | 0.002987829 | 67 |
| hsa00052 Galactose metabolism | 0.230275804 | 0.731257184 | 0.000936234 | 0.003952986 | 11 |
| hsa04973 Carbohydrate digestion and absorption | 0.24191186 | 0.701405749 | 0.001407961 | 0.005732411 | 11 |
| hsa04610 Complement and coagulation cascades | 0.245060211 | 0.659420134 | 0.002422761 | 0.009523956 | 16 |
| hsa04360 Axon guidance | 0.248083663 | 0.634750607 | 0.002988767 | 0.011357316 | 39 |
| hsa04621 NOD-like receptor signaling pathway | 0.227490129 | 0.624613615 | 0.003626699 | 0.013336891 | 30 |
| hsa04370 VEGF signaling pathway | 0.273274253 | 0.582740232 | 0.005923901 | 0.021103896 | 25 |
| hsa04730 Long-term depression | 0.27623823 | 0.56216917 | 0.00769666 | 0.026588463 | 20 |
| hsa00564 Glycerophospholipid metabolism | 0.290202996 | 0.531137092 | 0.01098721 | 0.036839469 | 27 |
| hsa00520 Amino sugar and nucleotide sugar metabolism | 0.294049878 | 0.518430093 | 0.012883231 | 0.041962525 | 18 |
| hsa00590 Arachidonic acid metabolism | 0.283143841 | 0.510081145 | 0.014354733 | 0.045456654 | 15 |
| hsa04630 Jak-STAT signaling pathway | 0.235728391 | 0.500166708 | 0.01536124 | 0.047329226 | 50 |
| hsa00983 Drug metabolism - other enzymes | 0.318271059 | 0.458430004 | 0.02518885 | 0.075333421 | 11 |
| hsa04115 p53 signaling pathway | 0.311111894 | 0.450529732 | 0.02577196 | 0.075333421 | 28 |
| hsa00600 Sphingolipid metabolism | 0.319834909 | 0.447575713 | 0.027971255 | 0.079718078 | 15 |
| hsa04540 Gap junction | 0.305646155 | 0.433600064 | 0.030430748 | 0.084612325 | 31 |
| hsa00860 Porphyrin and chlorophyll metabolism | 0.286189662 | 0.421130695 | 0.037565435 | 0.101963323 | 11 |
| hsa04150 mTOR signaling pathway | 0.340950455 | 0.397427429 | 0.04350669 | 0.113117877 | 17 |
| hsa04310 Wnt signaling pathway | 0.331469051 | 0.394569796 | 0.043659531 | 0.113117877 | 33 |
| hsa04662 B cell receptor signaling pathway | 0.303354669 | 0.340558377 | 0.070081134 | 0.177538873 | 36 |
| hsa00532 Glycosaminoglycan biosynthesis - chondroitin sulfate | 0.368164857 | 0.324222579 | 0.083395241 | 0.206631215 | 10 |
| hsa04141 Protein processing in endoplasmic reticulum | 0.331189279 | 0.316661248 | 0.085190062 | 0.206631215 | 55 |
| hsa03050 Proteasome | 0.359031213 | 0.268075669 | 0.127058766 | 0.30176457 | 15 |
| hsa04960 Aldosterone-regulated sodium reabsorption | 0.390996471 | 0.265854694 | 0.130219016 | 0.302958528 | 10 |
| hsa04146 Peroxisome | 0.389150368 | 0.23346118 | 0.156056346 | 0.355808468 | 27 |
| hsa04622 RIG-I-like receptor signaling pathway | 0.363319151 | 0.230038908 | 0.160749313 | 0.359321993 | 19 |
| hsa00562 Inositol phosphate metabolism | 0.411370149 | 0.184126116 | 0.215220712 | 0.471830023 | 16 |
| hsa04270 Vascular smooth muscle contraction | 0.401505332 | 0.175943223 | 0.223055223 | 0.479779158 | 32 |
| hsa04974 Protein digestion and absorption | 0.389797521 | 0.148973549 | 0.261125277 | 0.551264473 | 20 |
| hsa04623 Cytosolic DNA-sensing pathway | 0.36800736 | 0.124878059 | 0.300262436 | 0.622362141 | 17 |
| hsa04914 Progesterone-mediated oocyte maturation | 0.44240046 | 0.103737445 | 0.326500689 | 0.664662117 | 37 |
| hsa04512 ECM-receptor interaction | 0.436009472 | 0.061837572 | 0.392897886 | 0.777391009 | 17 |
| hsa04130 SNARE interactions in vesicular transport | 0.459979913 | 0.059400836 | 0.399168389 | 0.777391009 | 17 |
| hsa04320 Dorso-ventral axis formation | 0.460532672 | 0.056790719 | 0.406367605 | 0.777391009 | 10 |
| hsa00480 Glutathione metabolism | 0.431579726 | 0.053231095 | 0.409153163 | 0.777391009 | 18 |
| hsa04912 GnRH signaling pathway | 0.479846099 | 0.033999205 | 0.441562404 | 0.824213327 | 28 |
| hsa00330 Arginine and proline metabolism | 0.452189972 | 0.027380649 | 0.452115639 | 0.824213327 | 17 |
| hsa04640 Hematopoietic cell lineage | 0.45550092 | 0.025942863 | 0.455486312 | 0.824213327 | 49 |
| hsa04975 Fat digestion and absorption | 0.483864467 | 0.013198276 | 0.477428233 | 0.850419041 | 11 |
| hsa04962 Vasopressin-regulated water reabsorption | 0.490358214 | 0.002697565 | 0.495347886 | 0.868763984 | 15 |
| hsa00565 Ether lipid metabolism | 0.49525664 | -0.006552329 | 0.510954746 | 0.872449515 | 11 |
| hsa04916 Melanogenesis | 0.49429824 | -0.007438543 | 0.512755417 | 0.872449515 | 25 |
| hsa04972 Pancreatic secretion | 0.504725422 | -0.025481841 | 0.543806231 | 0.908570002 | 20 |
| hsa00071 Fatty acid metabolism | 0.481760323 | -0.03007917 | 0.549923948 | 0.908570002 | 14 |
| hsa04012 ErbB signaling pathway | 0.509151509 | -0.041425859 | 0.571024586 | 0.929954325 | 28 |
| hsa04340 Hedgehog signaling pathway | 0.515430457 | -0.051318852 | 0.586236862 | 0.941281722 | 11 |
| hsa04970 Salivary secretion | 0.52262667 | -0.071986586 | 0.621631808 | 0.984250362 | 16 |
| hsa04740 Olfactory transduction | 0.507316273 | -0.078865103 | 0.632770843 | 0.988162687 | 26 |
| hsa00534 Glycosaminoglycan biosynthesis - heparan sulfate | 0.528288504 | -0.093792889 | 0.655177429 | 1 | 13 |
| hsa00561 Glycerolipid metabolism | 0.533062754 | -0.099841366 | 0.667028534 | 1 | 21 |
| hsa00640 Propanoate metabolism | 0.533575311 | -0.125014024 | 0.704416472 | 1 | 12 |
| hsa04070 Phosphatidylinositol signaling system | 0.540308736 | -0.132137598 | 0.715115518 | 1 | 21 |
| hsa04720 Long-term potentiation | 0.546122583 | -0.133416835 | 0.717423883 | 1 | 23 |
| hsa03022 Basal transcription factors | 0.545041553 | -0.159747559 | 0.751115205 | 1 | 13 |
| hsa00310 Lysine degradation | 0.557897978 | -0.199252793 | 0.804005476 | 1 | 14 |
| hsa04350 TGF-beta signaling pathway | 0.56592326 | -0.209497793 | 0.816282057 | 1 | 23 |
| hsa00350 Tyrosine metabolism | 0.574378348 | -0.222558116 | 0.827279636 | 1 | 11 |
| hsa00982 Drug metabolism - cytochrome P450 | 0.577643382 | -0.258736332 | 0.864660474 | 1 | 10 |
| hsa04110 Cell cycle | 0.546692757 | -0.261309618 | 0.870768802 | 1 | 41 |
| hsa04114 Oocyte meiosis | 0.592320415 | -0.265090918 | 0.8745427 | 1 | 41 |
| hsa03015 mRNA surveillance pathway | 0.595717721 | -0.297152628 | 0.89864673 | 1 | 20 |
| hsa04020 Calcium signaling pathway | 0.604055818 | -0.295711026 | 0.899774828 | 1 | 38 |
| hsa00380 Tryptophan metabolism | 0.602509326 | -0.314256078 | 0.909773844 | 1 | 11 |
| hsa04330 Notch signaling pathway | 0.609621025 | -0.385076473 | 0.950341119 | 1 | 13 |
| hsa03440 Homologous recombination | 0.642460278 | -0.418375221 | 0.961313798 | 1 | 10 |
| hsa00240 Pyrimidine metabolism | 0.646417304 | -0.421613293 | 0.966005857 | 1 | 35 |
| hsa00340 Histidine metabolism | 0.649452139 | -0.437050794 | 0.968744737 | 1 | 11 |
| hsa00620 Pyruvate metabolism | 0.626413943 | -0.437595398 | 0.970432029 | 1 | 22 |
| hsa00280 Valine, leucine and isoleucine degradation | 0.651704934 | -0.44308936 | 0.971465917 | 1 | 18 |
| hsa00510 N-Glycan biosynthesis | 0.655610754 | -0.464390398 | 0.975679095 | 1 | 11 |
| hsa04120 Ubiquitin mediated proteolysis | 0.662805312 | -0.508989698 | 0.985995059 | 1 | 38 |
| hsa04660 T cell receptor signaling pathway | 0.618600677 | -0.531364514 | 0.989352139 | 1 | 52 |
| hsa03018 RNA degradation | 0.663623155 | -0.558974754 | 0.99204445 | 1 | 31 |
| hsa00514 Other types of O-glycan biosynthesis | 0.699843767 | -0.598736591 | 0.994492248 | 1 | 12 |
| hsa03420 Nucleotide excision repair | 0.698970853 | -0.626920967 | 0.996132789 | 1 | 14 |
| hsa04260 Cardiac muscle contraction | 0.630022903 | -0.635745402 | 0.996721052 | 1 | 19 |
| hsa00020 Citrate cycle (TCA cycle) | 0.703246737 | -0.681577417 | 0.998044929 | 1 | 12 |
| hsa03410 Base excision repair | 0.738252551 | -0.710759384 | 0.998729203 | 1 | 14 |
| hsa03030 DNA replication | 0.715694883 | -0.722475813 | 0.998874325 | 1 | 13 |
| hsa00970 Aminoacyl-tRNA biosynthesis | 0.694849798 | -0.765389945 | 0.999459538 | 1 | 16 |
| hsa03008 Ribosome biogenesis in eukaryotes | 0.718654093 | -0.82675526 | 0.999797242 | 1 | 25 |
| hsa00230 Purine metabolism | 0.766696857 | -0.926353324 | 0.99996939 | 1 | 53 |
| hsa04514 Cell adhesion molecules (CAMs) | 0.783995938 | -1.153409151 | 0.999999634 | 1 | 43 |
| hsa03040 Spliceosome | 0.818204218 | -1.211272899 | 0.999999911 | 1 | 55 |
| hsa00190 Oxidative phosphorylation | 0.518124003 | -1.286456727 | 0.99999998 | 1 | 50 |
| hsa03013 RNA transport | 0.81606492 | -1.4303563 | 1 | 1 | 52 |
| hsa04612 Antigen processing and presentation | 0.846477013 | -1.471357121 | 1 | 1 | 35 |
| hsa04672 Intestinal immune network for IgA production | 0.920134709 | -1.923000744 | 1 | 1 | 28 |
| hsa03010 Ribosome | 0.67474983 | -4.82383644 | 1 | 1 | 44 |
| hsa00040 Pentose and glucuronate interconversions | NA | NA | NA | NA | 6 |
| hsa00053 Ascorbate and aldarate metabolism | NA | NA | NA | NA | 4 |
| hsa00061 Fatty acid biosynthesis | NA | NA | NA | NA | 2 |
| hsa00072 Synthesis and degradation of ketone bodies | NA | NA | NA | NA | 1 |
| hsa00100 Steroid biosynthesis | NA | NA | NA | NA | 6 |
| hsa00120 Primary bile acid biosynthesis | NA | NA | NA | NA | 2 |
| hsa00130 Ubiquinone and other terpenoid-quinone biosynthesis | NA | NA | NA | NA | 4 |
| hsa00140 Steroid hormone biosynthesis | NA | NA | NA | NA | 5 |
| hsa00232 Caffeine metabolism | NA | NA | NA | NA | 0 |
| hsa00250 Alanine, aspartate and glutamate metabolism | NA | NA | NA | NA | 7 |
| hsa00260 Glycine, serine and threonine metabolism | NA | NA | NA | NA | 9 |
| hsa00270 Cysteine and methionine metabolism | NA | NA | NA | NA | 9 |
| hsa00290 Valine, leucine and isoleucine biosynthesis | NA | NA | NA | NA | 7 |
| hsa00300 Lysine biosynthesis | NA | NA | NA | NA | 0 |
| hsa00360 Phenylalanine metabolism | NA | NA | NA | NA | 5 |
| hsa00400 Phenylalanine, tyrosine and tryptophan biosynthesis | NA | NA | NA | NA | 1 |
| hsa00410 beta-Alanine metabolism | NA | NA | NA | NA | 7 |
| hsa00430 Taurine and hypotaurine metabolism | NA | NA | NA | NA | 1 |
| hsa00450 Selenocompound metabolism | NA | NA | NA | NA | 6 |
| hsa00460 Cyanoamino acid metabolism | NA | NA | NA | NA | 3 |
| hsa00471 D-Glutamine and D-glutamate metabolism | NA | NA | NA | NA | 0 |
| hsa00472 D-Arginine and D-ornithine metabolism | NA | NA | NA | NA | 0 |
| hsa00511 Other glycan degradation | NA | NA | NA | NA | 2 |
| hsa00512 Mucin type O-Glycan biosynthesis | NA | NA | NA | NA | 8 |
| hsa00531 Glycosaminoglycan degradation | NA | NA | NA | NA | 7 |
| hsa00533 Glycosaminoglycan biosynthesis - keratan sulfate | NA | NA | NA | NA | 4 |
| hsa00563 Glycosylphosphatidylinositol(GPI)-anchor biosynthesis | NA | NA | NA | NA | 5 |
| hsa00591 Linoleic acid metabolism | NA | NA | NA | NA | 4 |
| hsa00592 alpha-Linolenic acid metabolism | NA | NA | NA | NA | 3 |
| hsa00601 Glycosphingolipid biosynthesis - lacto and neolacto series | NA | NA | NA | NA | 9 |
| hsa00603 Glycosphingolipid biosynthesis - globo series | NA | NA | NA | NA | 4 |
| hsa00604 Glycosphingolipid biosynthesis - ganglio series | NA | NA | NA | NA | 6 |
| hsa00630 Glyoxylate and dicarboxylate metabolism | NA | NA | NA | NA | 8 |
| hsa00650 Butanoate metabolism | NA | NA | NA | NA | 7 |
| hsa00670 One carbon pool by folate | NA | NA | NA | NA | 5 |
| hsa00730 Thiamine metabolism | NA | NA | NA | NA | 1 |
| hsa00740 Riboflavin metabolism | NA | NA | NA | NA | 5 |
| hsa00750 Vitamin B6 metabolism | NA | NA | NA | NA | 2 |
| hsa00760 Nicotinate and nicotinamide metabolism | NA | NA | NA | NA | 5 |
| hsa00770 Pantothenate and CoA biosynthesis | NA | NA | NA | NA | 8 |
| hsa00780 Biotin metabolism | NA | NA | NA | NA | 0 |
| hsa00785 Lipoic acid metabolism | NA | NA | NA | NA | 2 |
| hsa00790 Folate biosynthesis | NA | NA | NA | NA | 3 |
| hsa00830 Retinol metabolism | NA | NA | NA | NA | 5 |
| hsa00900 Terpenoid backbone biosynthesis | NA | NA | NA | NA | 4 |
| hsa00910 Nitrogen metabolism | NA | NA | NA | NA | 5 |
| hsa00920 Sulfur metabolism | NA | NA | NA | NA | 6 |
| hsa00980 Metabolism of xenobiotics by cytochrome P450 | NA | NA | NA | NA | 9 |
| hsa01040 Biosynthesis of unsaturated fatty acids | NA | NA | NA | NA | 9 |
| hsa02010 ABC transporters | NA | NA | NA | NA | 7 |
| hsa03020 RNA polymerase | NA | NA | NA | NA | 8 |
| hsa03060 Protein export | NA | NA | NA | NA | 7 |
| hsa03430 Mismatch repair | NA | NA | NA | NA | 7 |
| hsa03450 Non-homologous end-joining | NA | NA | NA | NA | 3 |
| hsa04122 Sulfur relay system | NA | NA | NA | NA | 1 |
| hsa04140 Regulation of autophagy | NA | NA | NA | NA | 6 |
| hsa04614 Renin-angiotensin system | NA | NA | NA | NA | 5 |
| hsa04710 Circadian rhythm - mammal | NA | NA | NA | NA | 5 |
| hsa04742 Taste transduction | NA | NA | NA | NA | 8 |
| hsa04744 Phototransduction | NA | NA | NA | NA | 4 |
| hsa04964 Proximal tubule bicarbonate reclamation | NA | NA | NA | NA | 4 |
| hsa04966 Collecting duct acid secretion | NA | NA | NA | NA | 9 |
| hsa04977 Vitamin digestion and absorption | NA | NA | NA | NA | 4 |
| hsa03010 Ribosome | 7.77E-07 | -4.82383644 | 3.43E-78 | 3.91E-76 | 44 |
| hsa04672 Intestinal immune network for IgA production | 0.025613824 | -1.923000744 | 2.27E-16 | 1.29E-14 | 28 |
| hsa04612 Antigen processing and presentation | 0.059175179 | -1.471357121 | 1.65E-10 | 6.27E-09 | 35 |
| hsa03013 RNA transport | 0.062799083 | -1.4303563 | 3.80E-10 | 1.08E-08 | 52 |
| hsa00190 Oxidative phosphorylation | 0.039003681 | -1.286456727 | 2.00E-08 | 4.56E-07 | 50 |
| hsa03040 Spliceosome | 0.097913217 | -1.211272899 | 8.89E-08 | 1.69E-06 | 55 |
| hsa04514 Cell adhesion molecules (CAMs) | 0.101366727 | -1.153409151 | 3.66E-07 | 5.95E-06 | 43 |
| hsa00230 Purine metabolism | 0.159594793 | -0.926353324 | 3.06E-05 | 0.000436199 | 53 |
| hsa03008 Ribosome biogenesis in eukaryotes | 0.182118354 | -0.82675526 | 0.000202758 | 0.002568272 | 25 |
| hsa00970 Aminoacyl-tRNA biosynthesis | 0.199499524 | -0.765389945 | 0.000540462 | 0.006161265 | 16 |
| hsa03030 DNA replication | 0.22562444 | -0.722475813 | 0.001125675 | 0.011666083 | 13 |
| hsa03410 Base excision repair | 0.236561864 | -0.710759384 | 0.001270797 | 0.012072575 | 14 |
| hsa00020 Citrate cycle (TCA cycle) | 0.234045694 | -0.681577417 | 0.001955071 | 0.017144473 | 12 |
| hsa04260 Cardiac muscle contraction | 0.218658227 | -0.635745402 | 0.003278948 | 0.026700001 | 19 |
| hsa03420 Nucleotide excision repair | 0.257102696 | -0.626920967 | 0.003867211 | 0.029390805 | 14 |
| hsa00514 Other types of O-glycan biosynthesis | 0.268746322 | -0.598736591 | 0.005507752 | 0.039242736 | 12 |
| hsa03018 RNA degradation | 0.264004808 | -0.558974754 | 0.00795555 | 0.053348984 | 31 |
| hsa04660 T cell receptor signaling pathway | 0.256566763 | -0.531364514 | 0.010647861 | 0.067436456 | 52 |
| hsa04120 Ubiquitin mediated proteolysis | 0.289484996 | -0.508989698 | 0.014004941 | 0.084029648 | 38 |
| hsa00510 N-Glycan biosynthesis | 0.314209112 | -0.464390398 | 0.024320905 | 0.13862916 | 11 |
| hsa00280 Valine, leucine and isoleucine degradation | 0.320869632 | -0.44308936 | 0.028534083 | 0.153215852 | 18 |
| hsa00620 Pyruvate metabolism | 0.308438214 | -0.437595398 | 0.029567971 | 0.153215852 | 22 |
| hsa00340 Histidine metabolism | 0.324736621 | -0.437050794 | 0.031255263 | 0.154917388 | 11 |
| hsa00240 Pyrimidine metabolism | 0.327540915 | -0.421613293 | 0.033994143 | 0.161472178 | 35 |
| hsa03440 Homologous recombination | 0.333293763 | -0.418375221 | 0.038686202 | 0.176409082 | 10 |
| hsa04330 Notch signaling pathway | 0.32867722 | -0.385076473 | 0.049658881 | 0.217735092 | 13 |
| hsa00380 Tryptophan metabolism | 0.366118955 | -0.314256078 | 0.090226156 | 0.380954879 | 11 |
| hsa04020 Calcium signaling pathway | 0.376159732 | -0.295711026 | 0.100225172 | 0.398423199 | 38 |
| hsa03015 mRNA surveillance pathway | 0.370820501 | -0.297152628 | 0.10135327 | 0.398423199 | 20 |
| hsa04114 Oocyte meiosis | 0.387222526 | -0.265090918 | 0.1254573 | 0.475237309 | 41 |
| hsa04110 Cell cycle | 0.355021303 | -0.261309618 | 0.129231198 | 0.475237309 | 41 |
| hsa00982 Drug metabolism - cytochrome P450 | 0.383153567 | -0.258736332 | 0.135339526 | 0.48214706 | 10 |
| hsa00350 Tyrosine metabolism | 0.40522162 | -0.222558116 | 0.172720364 | 0.596670348 | 11 |
| hsa04350 TGF-beta signaling pathway | 0.404982156 | -0.209497793 | 0.183717943 | 0.615995455 | 23 |
| hsa00310 Lysine degradation | 0.406758356 | -0.199252793 | 0.195994524 | 0.638382163 | 14 |
| hsa03022 Basal transcription factors | 0.423949328 | -0.159747559 | 0.248884795 | 0.788135185 | 13 |
| hsa04720 Long-term potentiation | 0.442021952 | -0.133416835 | 0.282576117 | 0.854653447 | 23 |
| hsa04070 Phosphatidylinositol signaling system | 0.437884934 | -0.132137598 | 0.284884482 | 0.854653447 | 21 |
| hsa00640 Propanoate metabolism | 0.437630008 | -0.125014024 | 0.295583528 | 0.864013388 | 12 |
| hsa00561 Glycerolipid metabolism | 0.454933697 | -0.099841366 | 0.332971466 | 0.948968679 | 21 |
| hsa00534 Glycosaminoglycan biosynthesis - heparan sulfate | 0.455986632 | -0.093792889 | 0.344822571 | 0.958774953 | 13 |
| hsa04740 Olfactory transduction | 0.447156016 | -0.078865103 | 0.367229157 | 0.996764854 | 26 |
| hsa04970 Salivary secretion | 0.466445299 | -0.071986586 | 0.378368192 | 1 | 16 |
| hsa04340 Hedgehog signaling pathway | 0.475748551 | -0.051318852 | 0.413763138 | 1 | 11 |
| hsa04012 ErbB signaling pathway | 0.476830067 | -0.041425859 | 0.428975414 | 1 | 28 |
| hsa00071 Fatty acid metabolism | 0.459233451 | -0.03007917 | 0.450076052 | 1 | 14 |
| hsa04972 Pancreatic secretion | 0.484830862 | -0.025481841 | 0.456193769 | 1 | 20 |
| hsa04916 Melanogenesis | 0.488260643 | -0.007438543 | 0.487244583 | 1 | 25 |
| hsa00565 Ether lipid metabolism | 0.490277284 | -0.006552329 | 0.489045254 | 1 | 11 |
| hsa04962 Vasopressin-regulated water reabsorption | 0.492493948 | 0.002697565 | 0.504652114 | 1 | 15 |
| hsa04975 Fat digestion and absorption | 0.494143544 | 0.013198276 | 0.522571767 | 1 | 11 |
| hsa04640 Hematopoietic cell lineage | 0.475761832 | 0.025942863 | 0.544513688 | 1 | 49 |
| hsa00330 Arginine and proline metabolism | 0.472275028 | 0.027380649 | 0.547884361 | 1 | 17 |
| hsa04912 GnRH signaling pathway | 0.506558087 | 0.033999205 | 0.558437596 | 1 | 28 |
| hsa00480 Glutathione metabolism | 0.469997712 | 0.053231095 | 0.590846837 | 1 | 18 |
| hsa04320 Dorso-ventral axis formation | 0.503600092 | 0.056790719 | 0.593632395 | 1 | 10 |
| hsa04130 SNARE interactions in vesicular transport | 0.506161127 | 0.059400836 | 0.600831611 | 1 | 17 |
| hsa04512 ECM-receptor interaction | 0.481232165 | 0.061837572 | 0.607102114 | 1 | 17 |
| hsa04914 Progesterone-mediated oocyte maturation | 0.523878029 | 0.103737445 | 0.673499311 | 1 | 37 |
| hsa04623 Cytosolic DNA-sensing pathway | 0.455529536 | 0.124878059 | 0.699737564 | 1 | 17 |
| hsa04974 Protein digestion and absorption | 0.497807315 | 0.148973549 | 0.738874723 | 1 | 20 |
| hsa04270 Vascular smooth muscle contraction | 0.5332949 | 0.175943223 | 0.776944777 | 1 | 32 |
| hsa00562 Inositol phosphate metabolism | 0.550981155 | 0.184126116 | 0.784779288 | 1 | 16 |
| hsa04622 RIG-I-like receptor signaling pathway | 0.528116453 | 0.230038908 | 0.839250687 | 1 | 19 |
| hsa04146 Peroxisome | 0.567072048 | 0.23346118 | 0.843943654 | 1 | 27 |
| hsa04960 Aldosterone-regulated sodium reabsorption | 0.592768032 | 0.265854694 | 0.869780984 | 1 | 10 |
| hsa03050 Proteasome | 0.557960616 | 0.268075669 | 0.872941234 | 1 | 15 |
| hsa04141 Protein processing in endoplasmic reticulum | 0.564622289 | 0.316661248 | 0.914809938 | 1 | 55 |
| hsa00532 Glycosaminoglycan biosynthesis - chondroitin sulfate | 0.613941996 | 0.324222579 | 0.916604759 | 1 | 10 |
| hsa04662 B cell receptor signaling pathway | 0.524227981 | 0.340558377 | 0.929918866 | 1 | 36 |
| hsa04310 Wnt signaling pathway | 0.627708378 | 0.394569796 | 0.956340469 | 1 | 33 |
| hsa04150 mTOR signaling pathway | 0.643424277 | 0.397427429 | 0.95649331 | 1 | 17 |
| hsa00860 Porphyrin and chlorophyll metabolism | 0.565545027 | 0.421130695 | 0.962434565 | 1 | 11 |
| hsa04540 Gap junction | 0.62150259 | 0.433600064 | 0.969569252 | 1 | 31 |
| hsa00600 Sphingolipid metabolism | 0.652074913 | 0.447575713 | 0.972028745 | 1 | 15 |
| hsa04115 p53 signaling pathway | 0.646496094 | 0.450529732 | 0.97422804 | 1 | 28 |
| hsa00983 Drug metabolism - other enzymes | 0.659874293 | 0.458430004 | 0.97481115 | 1 | 11 |
| hsa04630 Jak-STAT signaling pathway | 0.558502179 | 0.500166708 | 0.98463876 | 1 | 50 |
| hsa00590 Arachidonic acid metabolism | 0.646474592 | 0.510081145 | 0.985645267 | 1 | 15 |
| hsa00520 Amino sugar and nucleotide sugar metabolism | 0.679184835 | 0.518430093 | 0.987116769 | 1 | 18 |
| hsa00564 Glycerophospholipid metabolism | 0.683459054 | 0.531137092 | 0.98901279 | 1 | 27 |
| hsa04730 Long-term depression | 0.686237904 | 0.56216917 | 0.99230334 | 1 | 20 |
| hsa04370 VEGF signaling pathway | 0.702699545 | 0.582740232 | 0.994076099 | 1 | 25 |
| hsa04621 NOD-like receptor signaling pathway | 0.65854571 | 0.624613615 | 0.996373301 | 1 | 30 |
| hsa04360 Axon guidance | 0.704632114 | 0.634750607 | 0.997011233 | 1 | 39 |
| hsa04610 Complement and coagulation cascades | 0.711659505 | 0.659420134 | 0.997577239 | 1 | 16 |
| hsa04973 Carbohydrate digestion and absorption | 0.744492304 | 0.701405749 | 0.998592039 | 1 | 11 |
| hsa00052 Galactose metabolism | 0.74752235 | 0.731257184 | 0.999063766 | 1 | 11 |
| hsa04144 Endocytosis | 0.719766219 | 0.738460837 | 0.999318565 | 1 | 67 |
| hsa03320 PPAR signaling pathway | 0.694428157 | 0.750264896 | 0.999330444 | 1 | 19 |
| hsa04976 Bile secretion | 0.744542264 | 0.762650629 | 0.999469608 | 1 | 19 |
| hsa04971 Gastric acid secretion | 0.752953653 | 0.761693351 | 0.999470832 | 1 | 21 |
| hsa04722 Neurotrophin signaling pathway | 0.75834553 | 0.798404883 | 0.999727112 | 1 | 49 |
| hsa04145 Phagosome | 0.626506377 | 0.840558448 | 0.999860557 | 1 | 72 |
| hsa04210 Apoptosis | 0.752340516 | 0.849188966 | 0.999878818 | 1 | 42 |
| hsa00010 Glycolysis / Gluconeogenesis | 0.742930964 | 0.881968186 | 0.999926396 | 1 | 30 |
| hsa04530 Tight junction | 0.782808536 | 0.889621278 | 0.999940656 | 1 | 41 |
| hsa04910 Insulin signaling pathway | 0.796855098 | 0.922536153 | 0.999967744 | 1 | 50 |
| hsa00030 Pentose phosphate pathway | 0.727141546 | 1.000753343 | 0.999985395 | 1 | 13 |
| hsa04920 Adipocytokine signaling pathway | 0.794788087 | 0.980627912 | 0.99998773 | 1 | 27 |
| hsa00051 Fructose and mannose metabolism | 0.813247994 | 1.004180356 | 0.999990366 | 1 | 15 |
| hsa04520 Adherens junction | 0.819767155 | 0.993747894 | 0.999990863 | 1 | 31 |
| hsa04062 Chemokine signaling pathway | 0.767920201 | 1.031280151 | 0.99999596 | 1 | 72 |
| hsa00500 Starch and sucrose metabolism | 0.837331324 | 1.060429956 | 0.999996538 | 1 | 12 |
| hsa04010 MAPK signaling pathway | 0.81590566 | 1.078335 | 0.999998581 | 1 | 97 |
| hsa04510 Focal adhesion | 0.835612855 | 1.109655708 | 0.999999241 | 1 | 64 |
| hsa04620 Toll-like receptor signaling pathway | 0.81025081 | 1.141764999 | 0.999999544 | 1 | 36 |
| hsa04142 Lysosome | 0.79660643 | 1.186075718 | 0.999999847 | 1 | 51 |
| hsa04664 Fc epsilon RI signaling pathway | 0.814987923 | 1.199401081 | 0.999999857 | 1 | 26 |
| hsa04650 Natural killer cell mediated cytotoxicity | 0.815759204 | 1.368939285 | 0.999999998 | 1 | 44 |
| hsa04666 Fc gamma R-mediated phagocytosis | 0.907783818 | 1.649307533 | 1 | 1 | 47 |
| hsa04670 Leukocyte transendothelial migration | 0.898114304 | 1.760501027 | 1 | 1 | 40 |
| hsa04380 Osteoclast differentiation | 0.777852778 | 1.784762018 | 1 | 1 | 65 |
| hsa04810 Regulation of actin cytoskeleton | 0.916930682 | 1.900312857 | 1 | 1 | 71 |
| hsa00040 Pentose and glucuronate interconversions | NA | NA | NA | NA | 6 |
| hsa00053 Ascorbate and aldarate metabolism | NA | NA | NA | NA | 4 |
| hsa00061 Fatty acid biosynthesis | NA | NA | NA | NA | 2 |
| hsa00072 Synthesis and degradation of ketone bodies | NA | NA | NA | NA | 1 |
| hsa00100 Steroid biosynthesis | NA | NA | NA | NA | 6 |
| hsa00120 Primary bile acid biosynthesis | NA | NA | NA | NA | 2 |
| hsa00130 Ubiquinone and other terpenoid-quinone biosynthesis | NA | NA | NA | NA | 4 |
| hsa00140 Steroid hormone biosynthesis | NA | NA | NA | NA | 5 |
| hsa00232 Caffeine metabolism | NA | NA | NA | NA | 0 |
| hsa00250 Alanine, aspartate and glutamate metabolism | NA | NA | NA | NA | 7 |
| hsa00260 Glycine, serine and threonine metabolism | NA | NA | NA | NA | 9 |
| hsa00270 Cysteine and methionine metabolism | NA | NA | NA | NA | 9 |
| hsa00290 Valine, leucine and isoleucine biosynthesis | NA | NA | NA | NA | 7 |
| hsa00300 Lysine biosynthesis | NA | NA | NA | NA | 0 |
| hsa00360 Phenylalanine metabolism | NA | NA | NA | NA | 5 |
| hsa00400 Phenylalanine, tyrosine and tryptophan biosynthesis | NA | NA | NA | NA | 1 |
| hsa00410 beta-Alanine metabolism | NA | NA | NA | NA | 7 |
| hsa00430 Taurine and hypotaurine metabolism | NA | NA | NA | NA | 1 |
| hsa00450 Selenocompound metabolism | NA | NA | NA | NA | 6 |
| hsa00460 Cyanoamino acid metabolism | NA | NA | NA | NA | 3 |
| hsa00471 D-Glutamine and D-glutamate metabolism | NA | NA | NA | NA | 0 |
| hsa00472 D-Arginine and D-ornithine metabolism | NA | NA | NA | NA | 0 |
| hsa00511 Other glycan degradation | NA | NA | NA | NA | 2 |
| hsa00512 Mucin type O-Glycan biosynthesis | NA | NA | NA | NA | 8 |
| hsa00531 Glycosaminoglycan degradation | NA | NA | NA | NA | 7 |
| hsa00533 Glycosaminoglycan biosynthesis - keratan sulfate | NA | NA | NA | NA | 4 |
| hsa00563 Glycosylphosphatidylinositol(GPI)-anchor biosynthesis | NA | NA | NA | NA | 5 |
| hsa00591 Linoleic acid metabolism | NA | NA | NA | NA | 4 |
| hsa00592 alpha-Linolenic acid metabolism | NA | NA | NA | NA | 3 |
| hsa00601 Glycosphingolipid biosynthesis - lacto and neolacto series | NA | NA | NA | NA | 9 |
| hsa00603 Glycosphingolipid biosynthesis - globo series | NA | NA | NA | NA | 4 |
| hsa00604 Glycosphingolipid biosynthesis - ganglio series | NA | NA | NA | NA | 6 |
| hsa00630 Glyoxylate and dicarboxylate metabolism | NA | NA | NA | NA | 8 |
| hsa00650 Butanoate metabolism | NA | NA | NA | NA | 7 |
| hsa00670 One carbon pool by folate | NA | NA | NA | NA | 5 |
| hsa00730 Thiamine metabolism | NA | NA | NA | NA | 1 |
| hsa00740 Riboflavin metabolism | NA | NA | NA | NA | 5 |
| hsa00750 Vitamin B6 metabolism | NA | NA | NA | NA | 2 |
| hsa00760 Nicotinate and nicotinamide metabolism | NA | NA | NA | NA | 5 |
| hsa00770 Pantothenate and CoA biosynthesis | NA | NA | NA | NA | 8 |
| hsa00780 Biotin metabolism | NA | NA | NA | NA | 0 |
| hsa00785 Lipoic acid metabolism | NA | NA | NA | NA | 2 |
| hsa00790 Folate biosynthesis | NA | NA | NA | NA | 3 |
| hsa00830 Retinol metabolism | NA | NA | NA | NA | 5 |
| hsa00900 Terpenoid backbone biosynthesis | NA | NA | NA | NA | 4 |
| hsa00910 Nitrogen metabolism | NA | NA | NA | NA | 5 |
| hsa00920 Sulfur metabolism | NA | NA | NA | NA | 6 |
| hsa00980 Metabolism of xenobiotics by cytochrome P450 | NA | NA | NA | NA | 9 |
| hsa01040 Biosynthesis of unsaturated fatty acids | NA | NA | NA | NA | 9 |
| hsa02010 ABC transporters | NA | NA | NA | NA | 7 |
| hsa03020 RNA polymerase | NA | NA | NA | NA | 8 |
| hsa03060 Protein export | NA | NA | NA | NA | 7 |
| hsa03430 Mismatch repair | NA | NA | NA | NA | 7 |
| hsa03450 Non-homologous end-joining | NA | NA | NA | NA | 3 |
| hsa04122 Sulfur relay system | NA | NA | NA | NA | 1 |
| hsa04140 Regulation of autophagy | NA | NA | NA | NA | 6 |
| hsa04614 Renin-angiotensin system | NA | NA | NA | NA | 5 |
| hsa04710 Circadian rhythm - mammal | NA | NA | NA | NA | 5 |
| hsa04742 Taste transduction | NA | NA | NA | NA | 8 |
| hsa04744 Phototransduction | NA | NA | NA | NA | 4 |
| hsa04964 Proximal tubule bicarbonate reclamation | NA | NA | NA | NA | 4 |
| hsa04966 Collecting duct acid secretion | NA | NA | NA | NA | 9 |
| hsa04977 Vitamin digestion and absorption | NA | NA | NA | NA | 4 |
|  |  |  |  |  |  |
| **GSE66099** | p.geomean | stat.mean | p.val | q.val | set.size |
| hsa04610 Complement and coagulation cascades | 0.003705049 | 2.627115289 | 3.01E-68 | 3.85E-66 | 27 |
| hsa04142 Lysosome | 0.01266978 | 2.027149078 | 2.33E-44 | 1.49E-42 | 59 |
| hsa04380 Osteoclast differentiation | 0.012269396 | 1.948024403 | 3.73E-41 | 1.59E-39 | 65 |
| hsa04810 Regulation of actin cytoskeleton | 0.017974084 | 1.886943364 | 3.68E-39 | 1.18E-37 | 77 |
| hsa04666 Fc gamma R-mediated phagocytosis | 0.029131543 | 1.692698376 | 1.66E-31 | 4.26E-30 | 45 |
| hsa03320 PPAR signaling pathway | 0.050250178 | 1.597233745 | 6.17E-28 | 1.32E-26 | 24 |
| hsa04621 NOD-like receptor signaling pathway | 0.049312688 | 1.507315834 | 5.97E-25 | 1.07E-23 | 25 |
| hsa04510 Focal adhesion | 0.042514625 | 1.478517677 | 6.67E-25 | 1.07E-23 | 76 |
| hsa00010 Glycolysis / Gluconeogenesis | 0.065143465 | 1.438619389 | 2.31E-23 | 3.28E-22 | 31 |
| hsa00983 Drug metabolism - other enzymes | 0.050625477 | 1.452537004 | 8.92E-23 | 1.14E-21 | 23 |
| hsa04620 Toll-like receptor signaling pathway | 0.054148099 | 1.404108552 | 3.23E-22 | 3.76E-21 | 35 |
| hsa04910 Insulin signaling pathway | 0.060899682 | 1.37837677 | 6.64E-22 | 7.08E-21 | 61 |
| hsa04145 Phagosome | 0.052262049 | 1.302658365 | 1.04E-19 | 1.02E-18 | 73 |
| hsa04512 ECM-receptor interaction | 0.060938568 | 1.290258349 | 6.09E-19 | 5.57E-18 | 33 |
| hsa04740 Olfactory transduction | 0.043112696 | 1.286221584 | 2.75E-18 | 2.29E-17 | 29 |
| hsa00052 Galactose metabolism | 0.093603146 | 1.272071517 | 2.87E-18 | 2.29E-17 | 16 |
| hsa00531 Glycosaminoglycan degradation | 0.098164887 | 1.257329942 | 7.38E-17 | 5.55E-16 | 10 |
| hsa00051 Fructose and mannose metabolism | 0.11236772 | 1.172167665 | 6.24E-16 | 4.44E-15 | 17 |
| hsa00480 Glutathione metabolism | 0.115759676 | 1.110178902 | 1.18E-14 | 7.92E-14 | 24 |
| hsa04920 Adipocytokine signaling pathway | 0.121086301 | 1.089798802 | 2.54E-14 | 1.58E-13 | 33 |
| hsa00190 Oxidative phosphorylation | 0.105760971 | 1.089125289 | 2.60E-14 | 1.58E-13 | 44 |
| hsa04020 Calcium signaling pathway | 0.098336386 | 1.071985624 | 5.23E-14 | 3.04E-13 | 64 |
| hsa00982 Drug metabolism - cytochrome P450 | 0.081431459 | 1.087563285 | 1.44E-13 | 7.93E-13 | 23 |
| hsa04010 MAPK signaling pathway | 0.10579043 | 1.04808766 | 1.49E-13 | 7.93E-13 | 98 |
| hsa04670 Leukocyte transendothelial migration | 0.107371889 | 1.039236046 | 3.31E-13 | 1.70E-12 | 46 |
| hsa04144 Endocytosis | 0.115257715 | 0.971773613 | 6.94E-12 | 3.42E-11 | 81 |
| hsa00564 Glycerophospholipid metabolism | 0.129797271 | 0.976771438 | 7.98E-12 | 3.78E-11 | 31 |
| hsa00140 Steroid hormone biosynthesis | 0.108621819 | 0.999920789 | 8.37E-12 | 3.83E-11 | 18 |
| hsa00860 Porphyrin and chlorophyll metabolism | 0.134304384 | 0.982959373 | 1.26E-11 | 5.54E-11 | 19 |
| hsa00980 Metabolism of xenobiotics by cytochrome P450 | 0.121269955 | 0.973182501 | 1.51E-11 | 6.46E-11 | 23 |
| hsa00600 Sphingolipid metabolism | 0.143705695 | 0.958750688 | 3.00E-11 | 1.24E-10 | 17 |
| hsa00030 Pentose phosphate pathway | 0.150939894 | 0.952746064 | 3.82E-11 | 1.53E-10 | 17 |
| hsa04320 Dorso-ventral axis formation | 0.160274512 | 0.935747774 | 1.42E-10 | 5.52E-10 | 11 |
| hsa04540 Gap junction | 0.140440959 | 0.894186191 | 3.45E-10 | 1.30E-09 | 36 |
| hsa04114 Oocyte meiosis | 0.133707655 | 0.888103923 | 3.92E-10 | 1.43E-09 | 50 |
| hsa04130 SNARE interactions in vesicular transport | 0.150579419 | 0.883380558 | 1.01E-09 | 3.61E-09 | 16 |
| hsa04270 Vascular smooth muscle contraction | 0.176069445 | 0.8137631 | 8.35E-09 | 2.89E-08 | 41 |
| hsa04912 GnRH signaling pathway | 0.170971979 | 0.808486958 | 1.05E-08 | 3.55E-08 | 39 |
| hsa04914 Progesterone-mediated oocyte maturation | 0.172100632 | 0.804150142 | 1.26E-08 | 4.13E-08 | 42 |
| hsa00512 Mucin type O-Glycan biosynthesis | 0.19059102 | 0.810585824 | 1.52E-08 | 4.85E-08 | 14 |
| hsa04722 Neurotrophin signaling pathway | 0.165526478 | 0.795151256 | 1.72E-08 | 5.37E-08 | 49 |
| hsa00500 Starch and sucrose metabolism | 0.129188063 | 0.76812641 | 8.44E-08 | 2.57E-07 | 29 |
| hsa00760 Nicotinate and nicotinamide metabolism | 0.211669604 | 0.753077767 | 1.68E-07 | 5.02E-07 | 10 |
| hsa04966 Collecting duct acid secretion | 0.207210052 | 0.740427687 | 2.33E-07 | 6.77E-07 | 12 |
| hsa00330 Arginine and proline metabolism | 0.208270356 | 0.729924788 | 2.39E-07 | 6.79E-07 | 23 |
| hsa04141 Protein processing in endoplasmic reticulum | 0.189196825 | 0.715726473 | 3.23E-07 | 8.99E-07 | 60 |
| hsa04115 p53 signaling pathway | 0.212291319 | 0.695100274 | 7.24E-07 | 1.97E-06 | 35 |
| hsa04370 VEGF signaling pathway | 0.200775123 | 0.695719348 | 7.84E-07 | 2.09E-06 | 29 |
| hsa04622 RIG-I-like receptor signaling pathway | 0.224190694 | 0.683065578 | 1.29E-06 | 3.32E-06 | 18 |
| hsa04976 Bile secretion | 0.196657044 | 0.683424679 | 1.30E-06 | 3.32E-06 | 24 |
| hsa04630 Jak-STAT signaling pathway | 0.200846678 | 0.648508842 | 3.25E-06 | 8.15E-06 | 59 |
| hsa00520 Amino sugar and nucleotide sugar metabolism | 0.222656696 | 0.622511145 | 8.59E-06 | 2.11E-05 | 29 |
| hsa04360 Axon guidance | 0.21054589 | 0.591536051 | 2.00E-05 | 4.82E-05 | 52 |
| hsa04260 Cardiac muscle contraction | 0.235501332 | 0.590618725 | 2.36E-05 | 5.59E-05 | 24 |
| hsa04975 Fat digestion and absorption | 0.25565959 | 0.578036426 | 4.33E-05 | 0.000100817 | 10 |
| hsa04664 Fc epsilon RI signaling pathway | 0.234623663 | 0.560978645 | 5.14E-05 | 0.000117457 | 31 |
| hsa04210 Apoptosis | 0.234016116 | 0.555656094 | 5.64E-05 | 0.000126666 | 46 |
| hsa00830 Retinol metabolism | 0.169950294 | 0.563733787 | 5.83E-05 | 0.000128713 | 26 |
| hsa03050 Proteasome | 0.23694637 | 0.543064357 | 0.000124335 | 0.000266461 | 13 |
| hsa04530 Tight junction | 0.263604072 | 0.526600436 | 0.000124904 | 0.000266461 | 44 |
| hsa00514 Other types of O-glycan biosynthesis | 0.237020502 | 0.4881476 | 0.000418067 | 0.000877256 | 18 |
| hsa04012 ErbB signaling pathway | 0.275335156 | 0.460884513 | 0.00069433 | 0.001433455 | 33 |
| hsa00310 Lysine degradation | 0.302109833 | 0.463259641 | 0.000740288 | 0.001504077 | 13 |
| hsa00380 Tryptophan metabolism | 0.307501964 | 0.448949223 | 0.001038137 | 0.002076273 | 13 |
| hsa04971 Gastric acid secretion | 0.280461857 | 0.439202628 | 0.001193463 | 0.002350204 | 26 |
| hsa04110 Cell cycle | 0.174672074 | 0.438409807 | 0.001290934 | 0.00250363 | 67 |
| hsa00053 Ascorbate and aldarate metabolism | 0.244022689 | 0.446737097 | 0.001436722 | 0.002744782 | 12 |
| hsa04520 Adherens junction | 0.280661701 | 0.415702695 | 0.001965147 | 0.0036991 | 31 |
| hsa04916 Melanogenesis | 0.301552839 | 0.362558429 | 0.00600789 | 0.011145072 | 33 |
| hsa00565 Ether lipid metabolism | 0.323048551 | 0.361186826 | 0.006930128 | 0.012672234 | 11 |
| hsa04962 Vasopressin-regulated water reabsorption | 0.334798032 | 0.341723509 | 0.009086183 | 0.016380725 | 19 |
| hsa00040 Pentose and glucuronate interconversions | 0.289787808 | 0.345056996 | 0.009290416 | 0.016516295 | 15 |
| hsa00561 Glycerolipid metabolism | 0.335207685 | 0.332713947 | 0.010704146 | 0.018768913 | 19 |
| hsa04150 mTOR signaling pathway | 0.337606934 | 0.320797255 | 0.01321614 | 0.022860349 | 23 |
| hsa04742 Taste transduction | 0.291028874 | 0.318718039 | 0.014402291 | 0.024579911 | 16 |
| hsa04730 Long-term depression | 0.332568569 | 0.306091725 | 0.017434524 | 0.029363409 | 21 |
| hsa00590 Arachidonic acid metabolism | 0.343245314 | 0.263932337 | 0.034058501 | 0.056616729 | 20 |
| hsa04623 Cytosolic DNA-sensing pathway | 0.354542664 | 0.24402704 | 0.04715838 | 0.077388111 | 15 |
| hsa04330 Notch signaling pathway | 0.35982807 | 0.192659157 | 0.091907442 | 0.148913325 | 20 |
| hsa03440 Homologous recombination | 0.374767582 | 0.189320952 | 0.098329508 | 0.157327212 | 14 |
| hsa04062 Chemokine signaling pathway | 0.292295649 | 0.15879599 | 0.133791923 | 0.211424273 | 89 |
| hsa04960 Aldosterone-regulated sodium reabsorption | 0.390562423 | 0.147898997 | 0.1562751 | 0.243941619 | 13 |
| hsa04973 Carbohydrate digestion and absorption | 0.331954464 | 0.146179308 | 0.158477206 | 0.244398583 | 21 |
| hsa04146 Peroxisome | 0.372717362 | 0.119390199 | 0.204114911 | 0.311032246 | 38 |
| hsa00534 Glycosaminoglycan biosynthesis - heparan sulfate | 0.442049424 | 0.093139652 | 0.261343285 | 0.393552241 | 10 |
| hsa00532 Glycosaminoglycan biosynthesis - chondroitin sulfate | 0.437849575 | 0.066691854 | 0.322068246 | 0.479357389 | 12 |
| hsa04720 Long-term potentiation | 0.440235062 | 0.039588775 | 0.391737188 | 0.576348967 | 24 |
| hsa04972 Pancreatic secretion | 0.428445132 | 0.032656772 | 0.409181868 | 0.595173626 | 33 |
| hsa00071 Fatty acid metabolism | 0.413265823 | 0.022840238 | 0.437035673 | 0.628545687 | 20 |
| hsa04974 Protein digestion and absorption | 0.400587787 | -0.00438383 | 0.512075141 | 0.728284645 | 19 |
| hsa04310 Wnt signaling pathway | 0.437700838 | -0.072120803 | 0.692043725 | 0.973424141 | 57 |
| hsa04662 B cell receptor signaling pathway | 0.36103007 | -0.07710529 | 0.703307374 | 0.978514608 | 39 |
| hsa04970 Salivary secretion | 0.512955548 | -0.202513164 | 0.919414566 | 1 | 30 |
| hsa00562 Inositol phosphate metabolism | 0.545419933 | -0.209184316 | 0.925845588 | 1 | 20 |
| hsa00340 Histidine metabolism | 0.544360095 | -0.232828907 | 0.943784408 | 1 | 11 |
| hsa00350 Tyrosine metabolism | 0.592645522 | -0.30800538 | 0.982976014 | 1 | 14 |
| hsa00240 Pyrimidine metabolism | 0.535418227 | -0.304257045 | 0.983005963 | 1 | 45 |
| hsa00640 Propanoate metabolism | 0.567389887 | -0.32318709 | 0.9869299 | 1 | 15 |
| hsa02010 ABC transporters | 0.571371839 | -0.33534975 | 0.98958063 | 1 | 19 |
| hsa04120 Ubiquitin mediated proteolysis | 0.477484887 | -0.33461628 | 0.990023117 | 1 | 60 |
| hsa04350 TGF-beta signaling pathway | 0.586336612 | -0.347386422 | 0.99214281 | 1 | 38 |
| hsa00620 Pyruvate metabolism | 0.543545294 | -0.363893801 | 0.993642594 | 1 | 16 |
| hsa00020 Citrate cycle (TCA cycle) | 0.548225146 | -0.373598925 | 0.994268151 | 1 | 10 |
| hsa00270 Cysteine and methionine metabolism | 0.582681441 | -0.366283307 | 0.994290773 | 1 | 18 |
| hsa04070 Phosphatidylinositol signaling system | 0.621304624 | -0.456833384 | 0.999213644 | 1 | 26 |
| hsa04640 Hematopoietic cell lineage | 0.560465997 | -0.468457781 | 0.999437017 | 1 | 51 |
| hsa00230 Purine metabolism | 0.624657536 | -0.501315713 | 0.999763164 | 1 | 71 |
| hsa03060 Protein export | 0.573804743 | -0.547418273 | 0.999875767 | 1 | 12 |
| hsa00563 Glycosylphosphatidylinositol(GPI)-anchor biosynthesis | 0.679893069 | -0.765756219 | 0.999999882 | 1 | 12 |
| hsa03410 Base excision repair | 0.649822419 | -0.782178347 | 0.999999944 | 1 | 15 |
| hsa03022 Basal transcription factors | 0.694202219 | -0.787481501 | 0.999999947 | 1 | 13 |
| hsa03430 Mismatch repair | 0.672068333 | -0.802861444 | 0.999999969 | 1 | 12 |
| hsa03015 mRNA surveillance pathway | 0.738013337 | -0.875760598 | 0.999999999 | 1 | 40 |
| hsa00510 N-Glycan biosynthesis | 0.735702206 | -0.926550133 | 1 | 1 | 20 |
| hsa00280 Valine, leucine and isoleucine degradation | 0.684717543 | -0.95942875 | 1 | 1 | 20 |
| hsa03420 Nucleotide excision repair | 0.637905123 | -0.963139829 | 1 | 1 | 22 |
| hsa03030 DNA replication | 0.610906963 | -0.976498889 | 1 | 1 | 18 |
| hsa00970 Aminoacyl-tRNA biosynthesis | 0.708900242 | -0.989717566 | 1 | 1 | 13 |
| hsa04514 Cell adhesion molecules (CAMs) | 0.664625853 | -1.052692299 | 1 | 1 | 59 |
| hsa04660 T cell receptor signaling pathway | 0.665854995 | -1.108616638 | 1 | 1 | 56 |
| hsa04650 Natural killer cell mediated cytotoxicity | 0.654357518 | -1.116430068 | 1 | 1 | 49 |
| hsa03008 Ribosome biogenesis in eukaryotes | 0.616574079 | -1.913281458 | 1 | 1 | 48 |
| hsa03010 Ribosome | 0.478525766 | -1.58007293 | 1 | 1 | 52 |
| hsa03013 RNA transport | 0.628940109 | -2.356686231 | 1 | 1 | 78 |
| hsa03018 RNA degradation | 0.722605049 | -1.260348082 | 1 | 1 | 35 |
| hsa03040 Spliceosome | 0.682745561 | -1.611086829 | 1 | 1 | 58 |
| hsa04612 Antigen processing and presentation | 0.834803569 | -2.239990543 | 1 | 1 | 40 |
| hsa04672 Intestinal immune network for IgA production | 0.790429155 | -1.866945746 | 1 | 1 | 31 |
| hsa00061 Fatty acid biosynthesis | NA | NA | NA | NA | 2 |
| hsa00072 Synthesis and degradation of ketone bodies | NA | NA | NA | NA | 3 |
| hsa00100 Steroid biosynthesis | NA | NA | NA | NA | 6 |
| hsa00120 Primary bile acid biosynthesis | NA | NA | NA | NA | 3 |
| hsa00130 Ubiquinone and other terpenoid-quinone biosynthesis | NA | NA | NA | NA | 4 |
| hsa00232 Caffeine metabolism | NA | NA | NA | NA | 1 |
| hsa00250 Alanine, aspartate and glutamate metabolism | NA | NA | NA | NA | 9 |
| hsa00260 Glycine, serine and threonine metabolism | NA | NA | NA | NA | 7 |
| hsa00290 Valine, leucine and isoleucine biosynthesis | NA | NA | NA | NA | 3 |
| hsa00300 Lysine biosynthesis | NA | NA | NA | NA | 0 |
| hsa00360 Phenylalanine metabolism | NA | NA | NA | NA | 5 |
| hsa00400 Phenylalanine, tyrosine and tryptophan biosynthesis | NA | NA | NA | NA | 1 |
| hsa00410 beta-Alanine metabolism | NA | NA | NA | NA | 9 |
| hsa00430 Taurine and hypotaurine metabolism | NA | NA | NA | NA | 4 |
| hsa00450 Selenocompound metabolism | NA | NA | NA | NA | 9 |
| hsa00460 Cyanoamino acid metabolism | NA | NA | NA | NA | 2 |
| hsa00471 D-Glutamine and D-glutamate metabolism | NA | NA | NA | NA | 1 |
| hsa00472 D-Arginine and D-ornithine metabolism | NA | NA | NA | NA | 0 |
| hsa00511 Other glycan degradation | NA | NA | NA | NA | 4 |
| hsa00533 Glycosaminoglycan biosynthesis - keratan sulfate | NA | NA | NA | NA | 6 |
| hsa00591 Linoleic acid metabolism | NA | NA | NA | NA | 6 |
| hsa00592 alpha-Linolenic acid metabolism | NA | NA | NA | NA | 5 |
| hsa00601 Glycosphingolipid biosynthesis - lacto and neolacto series | NA | NA | NA | NA | 9 |
| hsa00603 Glycosphingolipid biosynthesis - globo series | NA | NA | NA | NA | 6 |
| hsa00604 Glycosphingolipid biosynthesis - ganglio series | NA | NA | NA | NA | 9 |
| hsa00630 Glyoxylate and dicarboxylate metabolism | NA | NA | NA | NA | 6 |
| hsa00650 Butanoate metabolism | NA | NA | NA | NA | 9 |
| hsa00670 One carbon pool by folate | NA | NA | NA | NA | 8 |
| hsa00730 Thiamine metabolism | NA | NA | NA | NA | 0 |
| hsa00740 Riboflavin metabolism | NA | NA | NA | NA | 5 |
| hsa00750 Vitamin B6 metabolism | NA | NA | NA | NA | 4 |
| hsa00770 Pantothenate and CoA biosynthesis | NA | NA | NA | NA | 6 |
| hsa00780 Biotin metabolism | NA | NA | NA | NA | 0 |
| hsa00785 Lipoic acid metabolism | NA | NA | NA | NA | 2 |
| hsa00790 Folate biosynthesis | NA | NA | NA | NA | 5 |
| hsa00900 Terpenoid backbone biosynthesis | NA | NA | NA | NA | 4 |
| hsa00910 Nitrogen metabolism | NA | NA | NA | NA | 7 |
| hsa00920 Sulfur metabolism | NA | NA | NA | NA | 5 |
| hsa01040 Biosynthesis of unsaturated fatty acids | NA | NA | NA | NA | 8 |
| hsa03020 RNA polymerase | NA | NA | NA | NA | 9 |
| hsa03450 Non-homologous end-joining | NA | NA | NA | NA | 5 |
| hsa04122 Sulfur relay system | NA | NA | NA | NA | 3 |
| hsa04140 Regulation of autophagy | NA | NA | NA | NA | 9 |
| hsa04340 Hedgehog signaling pathway | NA | NA | NA | NA | 9 |
| hsa04614 Renin-angiotensin system | NA | NA | NA | NA | 5 |
| hsa04710 Circadian rhythm - mammal | NA | NA | NA | NA | 7 |
| hsa04744 Phototransduction | NA | NA | NA | NA | 9 |
| hsa04964 Proximal tubule bicarbonate reclamation | NA | NA | NA | NA | 6 |
| hsa04977 Vitamin digestion and absorption | NA | NA | NA | NA | 8 |
| hsa03013 RNA transport | 0.00224852 | -2.356686231 | 4.45E-58 | 5.69E-56 | 78 |
| hsa04612 Antigen processing and presentation | 0.007962322 | -2.239990543 | 2.80E-52 | 1.79E-50 | 40 |
| hsa03008 Ribosome biogenesis in eukaryotes | 0.010169063 | -1.913281458 | 9.27E-39 | 3.95E-37 | 48 |
| hsa04672 Intestinal immune network for IgA production | 0.019476949 | -1.866945746 | 8.33E-37 | 2.67E-35 | 31 |
| hsa03040 Spliceosome | 0.023118923 | -1.611086829 | 1.93E-28 | 4.93E-27 | 58 |
| hsa03010 Ribosome | 0.014619546 | -1.58007293 | 3.80E-26 | 8.11E-25 | 52 |
| hsa03018 RNA degradation | 0.06964811 | -1.260348082 | 3.16E-18 | 5.78E-17 | 35 |
| hsa04650 Natural killer cell mediated cytotoxicity | 0.082410013 | -1.116430068 | 7.03E-15 | 1.12E-13 | 49 |
| hsa04660 T cell receptor signaling pathway | 0.084384899 | -1.108616638 | 8.87E-15 | 1.26E-13 | 56 |
| hsa04514 Cell adhesion molecules (CAMs) | 0.09348904 | -1.052692299 | 1.62E-13 | 2.07E-12 | 59 |
| hsa00970 Aminoacyl-tRNA biosynthesis | 0.133072857 | -0.989717566 | 2.16E-11 | 2.52E-10 | 13 |
| hsa03030 DNA replication | 0.109155318 | -0.976498889 | 2.36E-11 | 2.52E-10 | 18 |
| hsa03420 Nucleotide excision repair | 0.115046907 | -0.963139829 | 2.97E-11 | 2.93E-10 | 22 |
| hsa00280 Valine, leucine and isoleucine degradation | 0.128816843 | -0.95942875 | 3.21E-11 | 2.93E-10 | 20 |
| hsa00510 N-Glycan biosynthesis | 0.152023099 | -0.926550133 | 1.07E-10 | 9.17E-10 | 20 |
| hsa03015 mRNA surveillance pathway | 0.165892281 | -0.875760598 | 6.44E-10 | 5.15E-09 | 40 |
| hsa03430 Mismatch repair | 0.175299738 | -0.802861444 | 3.09E-08 | 2.32E-07 | 12 |
| hsa03022 Basal transcription factors | 0.188589289 | -0.787481501 | 5.30E-08 | 3.77E-07 | 13 |
| hsa03410 Base excision repair | 0.173489164 | -0.782178347 | 5.62E-08 | 3.78E-07 | 15 |
| hsa00563 Glycosylphosphatidylinositol(GPI)-anchor biosynthesis | 0.19078658 | -0.765756219 | 1.18E-07 | 7.55E-07 | 12 |
| hsa03060 Protein export | 0.232311418 | -0.547418273 | 0.000124233 | 0.000757228 | 12 |
| hsa00230 Purine metabolism | 0.270034866 | -0.501315713 | 0.000236836 | 0.001377954 | 71 |
| hsa04640 Hematopoietic cell lineage | 0.254676694 | -0.468457781 | 0.000562983 | 0.003133122 | 51 |
| hsa04070 Phosphatidylinositol signaling system | 0.291470638 | -0.456833384 | 0.000786356 | 0.004193896 | 26 |
| hsa00270 Cysteine and methionine metabolism | 0.321081727 | -0.366283307 | 0.005709227 | 0.028218335 | 18 |
| hsa00020 Citrate cycle (TCA cycle) | 0.298123638 | -0.373598925 | 0.005731849 | 0.028218335 | 10 |
| hsa00620 Pyruvate metabolism | 0.295943726 | -0.363893801 | 0.006357406 | 0.030138814 | 16 |
| hsa04350 TGF-beta signaling pathway | 0.330278115 | -0.347386422 | 0.00785719 | 0.035918582 | 38 |
| hsa04120 Ubiquitin mediated proteolysis | 0.268074308 | -0.33461628 | 0.009976883 | 0.044035897 | 60 |
| hsa02010 ABC transporters | 0.328365142 | -0.33534975 | 0.01041937 | 0.044455978 | 19 |
| hsa00640 Propanoate metabolism | 0.335237773 | -0.32318709 | 0.0130701 | 0.053966863 | 15 |
| hsa00240 Pyrimidine metabolism | 0.326085856 | -0.304257045 | 0.016994037 | 0.066032429 | 45 |
| hsa00350 Tyrosine metabolism | 0.360644035 | -0.30800538 | 0.017023986 | 0.066032429 | 14 |
| hsa00340 Histidine metabolism | 0.37328606 | -0.232828907 | 0.056215592 | 0.211635171 | 11 |
| hsa00562 Inositol phosphate metabolism | 0.387769636 | -0.209184316 | 0.074154412 | 0.271193278 | 20 |
| hsa04970 Salivary secretion | 0.365046672 | -0.202513164 | 0.080585434 | 0.286525988 | 30 |
| hsa04662 B cell receptor signaling pathway | 0.316557592 | -0.07710529 | 0.296692626 | 1 | 39 |
| hsa04310 Wnt signaling pathway | 0.386978715 | -0.072120803 | 0.307956275 | 1 | 57 |
| hsa04974 Protein digestion and absorption | 0.398761984 | -0.00438383 | 0.487924859 | 1 | 19 |
| hsa00071 Fatty acid metabolism | 0.428667747 | 0.022840238 | 0.562964327 | 1 | 20 |
| hsa04972 Pancreatic secretion | 0.449222421 | 0.032656772 | 0.590818132 | 1 | 33 |
| hsa04720 Long-term potentiation | 0.469431029 | 0.039588775 | 0.608262812 | 1 | 24 |
| hsa00532 Glycosaminoglycan biosynthesis - chondroitin sulfate | 0.486384279 | 0.066691854 | 0.677931754 | 1 | 12 |
| hsa00534 Glycosaminoglycan biosynthesis - heparan sulfate | 0.512465415 | 0.093139652 | 0.738656715 | 1 | 10 |
| hsa04146 Peroxisome | 0.457986174 | 0.119390199 | 0.795885089 | 1 | 38 |
| hsa04973 Carbohydrate digestion and absorption | 0.430683001 | 0.146179308 | 0.841522794 | 1 | 21 |
| hsa04960 Aldosterone-regulated sodium reabsorption | 0.497436247 | 0.147898997 | 0.8437249 | 1 | 13 |
| hsa04062 Chemokine signaling pathway | 0.380277652 | 0.15879599 | 0.866208077 | 1 | 89 |
| hsa03440 Homologous recombination | 0.513956383 | 0.189320952 | 0.901670492 | 1 | 14 |
| hsa04330 Notch signaling pathway | 0.494678063 | 0.192659157 | 0.908092558 | 1 | 20 |
| hsa04623 Cytosolic DNA-sensing pathway | 0.529832268 | 0.24402704 | 0.95284162 | 1 | 15 |
| hsa00590 Arachidonic acid metabolism | 0.528832108 | 0.263932337 | 0.965941499 | 1 | 20 |
| hsa04730 Long-term depression | 0.550441643 | 0.306091725 | 0.982565476 | 1 | 21 |
| hsa04742 Taste transduction | 0.495743941 | 0.318718039 | 0.985597709 | 1 | 16 |
| hsa04150 mTOR signaling pathway | 0.570644265 | 0.320797255 | 0.98678386 | 1 | 23 |
| hsa00561 Glycerolipid metabolism | 0.576153301 | 0.332713947 | 0.989295854 | 1 | 19 |
| hsa00040 Pentose and glucuronate interconversions | 0.513728483 | 0.345056996 | 0.990709584 | 1 | 15 |
| hsa04962 Vasopressin-regulated water reabsorption | 0.584312457 | 0.341723509 | 0.990913817 | 1 | 19 |
| hsa00565 Ether lipid metabolism | 0.580006712 | 0.361186826 | 0.993069872 | 1 | 11 |
| hsa04916 Melanogenesis | 0.556507152 | 0.362558429 | 0.99399211 | 1 | 33 |
| hsa04520 Adherens junction | 0.559181787 | 0.415702695 | 0.998034853 | 1 | 31 |
| hsa00053 Ascorbate and aldarate metabolism | 0.513443249 | 0.446737097 | 0.998563278 | 1 | 12 |
| hsa04110 Cell cycle | 0.435995418 | 0.438409807 | 0.998709066 | 1 | 67 |
| hsa04971 Gastric acid secretion | 0.58475622 | 0.439202628 | 0.998806537 | 1 | 26 |
| hsa00380 Tryptophan metabolism | 0.635066091 | 0.448949223 | 0.998961863 | 1 | 13 |
| hsa00310 Lysine degradation | 0.639111986 | 0.463259641 | 0.999259712 | 1 | 13 |
| hsa04012 ErbB signaling pathway | 0.592629829 | 0.460884513 | 0.99930567 | 1 | 33 |
| hsa00514 Other types of O-glycan biosynthesis | 0.540895545 | 0.4881476 | 0.999581933 | 1 | 18 |
| hsa04530 Tight junction | 0.633836273 | 0.526600436 | 0.999875096 | 1 | 44 |
| hsa03050 Proteasome | 0.591292167 | 0.543064357 | 0.999875665 | 1 | 13 |
| hsa00830 Retinol metabolism | 0.468670082 | 0.563733787 | 0.999941677 | 1 | 26 |
| hsa04210 Apoptosis | 0.601301566 | 0.555656094 | 0.999943594 | 1 | 46 |
| hsa04664 Fc epsilon RI signaling pathway | 0.604192424 | 0.560978645 | 0.999948613 | 1 | 31 |
| hsa04975 Fat digestion and absorption | 0.652024693 | 0.578036426 | 0.99995668 | 1 | 10 |
| hsa04260 Cardiac muscle contraction | 0.637269495 | 0.590618725 | 0.999976426 | 1 | 24 |
| hsa04360 Axon guidance | 0.586093277 | 0.591536051 | 0.999980033 | 1 | 52 |
| hsa00520 Amino sugar and nucleotide sugar metabolism | 0.646334324 | 0.622511145 | 0.999991412 | 1 | 29 |
| hsa04630 Jak-STAT signaling pathway | 0.616445633 | 0.648508842 | 0.999996753 | 1 | 59 |
| hsa04976 Bile secretion | 0.635464081 | 0.683424679 | 0.999998703 | 1 | 24 |
| hsa04622 RIG-I-like receptor signaling pathway | 0.693807151 | 0.683065578 | 0.999998707 | 1 | 18 |
| hsa04370 VEGF signaling pathway | 0.656028461 | 0.695719348 | 0.999999216 | 1 | 29 |
| hsa04115 p53 signaling pathway | 0.688345993 | 0.695100274 | 0.999999276 | 1 | 35 |
| hsa04141 Protein processing in endoplasmic reticulum | 0.653812985 | 0.715726473 | 0.999999677 | 1 | 60 |
| hsa00330 Arginine and proline metabolism | 0.707152267 | 0.729924788 | 0.999999761 | 1 | 23 |
| hsa04966 Collecting duct acid secretion | 0.697630823 | 0.740427687 | 0.999999767 | 1 | 12 |
| hsa00760 Nicotinate and nicotinamide metabolism | 0.720020429 | 0.753077767 | 0.999999832 | 1 | 10 |
| hsa00500 Starch and sucrose metabolism | 0.548600828 | 0.76812641 | 0.999999916 | 1 | 29 |
| hsa04722 Neurotrophin signaling pathway | 0.663390448 | 0.795151256 | 0.999999983 | 1 | 49 |
| hsa00512 Mucin type O-Glycan biosynthesis | 0.727336763 | 0.810585824 | 0.999999985 | 1 | 14 |
| hsa04914 Progesterone-mediated oocyte maturation | 0.696081054 | 0.804150142 | 0.999999987 | 1 | 42 |
| hsa04912 GnRH signaling pathway | 0.686409867 | 0.808486958 | 0.999999989 | 1 | 39 |
| hsa04270 Vascular smooth muscle contraction | 0.71107199 | 0.8137631 | 0.999999992 | 1 | 41 |
| hsa04130 SNARE interactions in vesicular transport | 0.686226057 | 0.883380558 | 0.999999999 | 1 | 16 |
| hsa04114 Oocyte meiosis | 0.662166189 | 0.888103923 | 1 | 1 | 50 |
| hsa04540 Gap junction | 0.68400657 | 0.894186191 | 1 | 1 | 36 |
| hsa04320 Dorso-ventral axis formation | 0.750619375 | 0.935747774 | 1 | 1 | 11 |
| hsa00030 Pentose phosphate pathway | 0.753237135 | 0.952746064 | 1 | 1 | 17 |
| hsa00600 Sphingolipid metabolism | 0.734002849 | 0.958750688 | 1 | 1 | 17 |
| hsa00980 Metabolism of xenobiotics by cytochrome P450 | 0.675621088 | 0.973182501 | 1 | 1 | 23 |
| hsa00860 Porphyrin and chlorophyll metabolism | 0.728729034 | 0.982959373 | 1 | 1 | 19 |
| hsa00140 Steroid hormone biosynthesis | 0.641876744 | 0.999920789 | 1 | 1 | 18 |
| hsa00564 Glycerophospholipid metabolism | 0.70791611 | 0.976771438 | 1 | 1 | 31 |
| hsa04144 Endocytosis | 0.6613336 | 0.971773613 | 1 | 1 | 81 |
| hsa04670 Leukocyte transendothelial migration | 0.697138615 | 1.039236046 | 1 | 1 | 46 |
| hsa04010 MAPK signaling pathway | 0.707186444 | 1.04808766 | 1 | 1 | 98 |
| hsa00982 Drug metabolism - cytochrome P450 | 0.610342455 | 1.087563285 | 1 | 1 | 23 |
| hsa04020 Calcium signaling pathway | 0.695878714 | 1.071985624 | 1 | 1 | 64 |
| hsa00190 Oxidative phosphorylation | 0.737152037 | 1.089125289 | 1 | 1 | 44 |
| hsa04920 Adipocytokine signaling pathway | 0.796193616 | 1.089798802 | 1 | 1 | 33 |
| hsa00480 Glutathione metabolism | 0.786642536 | 1.110178902 | 1 | 1 | 24 |
| hsa00051 Fructose and mannose metabolism | 0.823922855 | 1.172167665 | 1 | 1 | 17 |
| hsa00531 Glycosaminoglycan degradation | 0.803065014 | 1.257329942 | 1 | 1 | 10 |
| hsa00010 Glycolysis / Gluconeogenesis | 0.849968102 | 1.438619389 | 1 | 1 | 31 |
| hsa00052 Galactose metabolism | 0.829285731 | 1.272071517 | 1 | 1 | 16 |
| hsa00983 Drug metabolism - other enzymes | 0.749096419 | 1.452537004 | 1 | 1 | 23 |
| hsa03320 PPAR signaling pathway | 0.886542926 | 1.597233745 | 1 | 1 | 24 |
| hsa04142 Lysosome | 0.818320689 | 2.027149078 | 1 | 1 | 59 |
| hsa04145 Phagosome | 0.668260621 | 1.302658365 | 1 | 1 | 73 |
| hsa04380 Osteoclast differentiation | 0.733913776 | 1.948024403 | 1 | 1 | 65 |
| hsa04510 Focal adhesion | 0.74311781 | 1.478517677 | 1 | 1 | 76 |
| hsa04512 ECM-receptor interaction | 0.69877176 | 1.290258349 | 1 | 1 | 33 |
| hsa04610 Complement and coagulation cascades | 0.899216217 | 2.627115289 | 1 | 1 | 27 |
| hsa04620 Toll-like receptor signaling pathway | 0.75078068 | 1.404108552 | 1 | 1 | 35 |
| hsa04621 NOD-like receptor signaling pathway | 0.790571784 | 1.507315834 | 1 | 1 | 25 |
| hsa04666 Fc gamma R-mediated phagocytosis | 0.781807072 | 1.692698376 | 1 | 1 | 45 |
| hsa04740 Olfactory transduction | 0.577770189 | 1.286221584 | 1 | 1 | 29 |
| hsa04810 Regulation of actin cytoskeleton | 0.811449739 | 1.886943364 | 1 | 1 | 77 |
| hsa04910 Insulin signaling pathway | 0.783235592 | 1.37837677 | 1 | 1 | 61 |
| hsa00061 Fatty acid biosynthesis | NA | NA | NA | NA | 2 |
| hsa00072 Synthesis and degradation of ketone bodies | NA | NA | NA | NA | 3 |
| hsa00100 Steroid biosynthesis | NA | NA | NA | NA | 6 |
| hsa00120 Primary bile acid biosynthesis | NA | NA | NA | NA | 3 |
| hsa00130 Ubiquinone and other terpenoid-quinone biosynthesis | NA | NA | NA | NA | 4 |
| hsa00232 Caffeine metabolism | NA | NA | NA | NA | 1 |
| hsa00250 Alanine, aspartate and glutamate metabolism | NA | NA | NA | NA | 9 |
| hsa00260 Glycine, serine and threonine metabolism | NA | NA | NA | NA | 7 |
| hsa00290 Valine, leucine and isoleucine biosynthesis | NA | NA | NA | NA | 3 |
| hsa00300 Lysine biosynthesis | NA | NA | NA | NA | 0 |
| hsa00360 Phenylalanine metabolism | NA | NA | NA | NA | 5 |
| hsa00400 Phenylalanine, tyrosine and tryptophan biosynthesis | NA | NA | NA | NA | 1 |
| hsa00410 beta-Alanine metabolism | NA | NA | NA | NA | 9 |
| hsa00430 Taurine and hypotaurine metabolism | NA | NA | NA | NA | 4 |
| hsa00450 Selenocompound metabolism | NA | NA | NA | NA | 9 |
| hsa00460 Cyanoamino acid metabolism | NA | NA | NA | NA | 2 |
| hsa00471 D-Glutamine and D-glutamate metabolism | NA | NA | NA | NA | 1 |
| hsa00472 D-Arginine and D-ornithine metabolism | NA | NA | NA | NA | 0 |
| hsa00511 Other glycan degradation | NA | NA | NA | NA | 4 |
| hsa00533 Glycosaminoglycan biosynthesis - keratan sulfate | NA | NA | NA | NA | 6 |
| hsa00591 Linoleic acid metabolism | NA | NA | NA | NA | 6 |
| hsa00592 alpha-Linolenic acid metabolism | NA | NA | NA | NA | 5 |
| hsa00601 Glycosphingolipid biosynthesis - lacto and neolacto series | NA | NA | NA | NA | 9 |
| hsa00603 Glycosphingolipid biosynthesis - globo series | NA | NA | NA | NA | 6 |
| hsa00604 Glycosphingolipid biosynthesis - ganglio series | NA | NA | NA | NA | 9 |
| hsa00630 Glyoxylate and dicarboxylate metabolism | NA | NA | NA | NA | 6 |
| hsa00650 Butanoate metabolism | NA | NA | NA | NA | 9 |
| hsa00670 One carbon pool by folate | NA | NA | NA | NA | 8 |
| hsa00730 Thiamine metabolism | NA | NA | NA | NA | 0 |
| hsa00740 Riboflavin metabolism | NA | NA | NA | NA | 5 |
| hsa00750 Vitamin B6 metabolism | NA | NA | NA | NA | 4 |
| hsa00770 Pantothenate and CoA biosynthesis | NA | NA | NA | NA | 6 |
| hsa00780 Biotin metabolism | NA | NA | NA | NA | 0 |
| hsa00785 Lipoic acid metabolism | NA | NA | NA | NA | 2 |
| hsa00790 Folate biosynthesis | NA | NA | NA | NA | 5 |
| hsa00900 Terpenoid backbone biosynthesis | NA | NA | NA | NA | 4 |
| hsa00910 Nitrogen metabolism | NA | NA | NA | NA | 7 |
| hsa00920 Sulfur metabolism | NA | NA | NA | NA | 5 |
| hsa01040 Biosynthesis of unsaturated fatty acids | NA | NA | NA | NA | 8 |
| hsa03020 RNA polymerase | NA | NA | NA | NA | 9 |
| hsa03450 Non-homologous end-joining | NA | NA | NA | NA | 5 |
| hsa04122 Sulfur relay system | NA | NA | NA | NA | 3 |
| hsa04140 Regulation of autophagy | NA | NA | NA | NA | 9 |
| hsa04340 Hedgehog signaling pathway | NA | NA | NA | NA | 9 |
| hsa04614 Renin-angiotensin system | NA | NA | NA | NA | 5 |
| hsa04710 Circadian rhythm - mammal | NA | NA | NA | NA | 7 |
| hsa04744 Phototransduction | NA | NA | NA | NA | 9 |
| hsa04964 Proximal tubule bicarbonate reclamation | NA | NA | NA | NA | 6 |
| hsa04977 Vitamin digestion and absorption | NA | NA | NA | NA | 8 |
|  |  |  |  |  |  |
| GSE72829 | p.geomean | stat.mean | p.val | q.val | set.size |
| hsa04670 Leukocyte transendothelial migration | 0.05415705 | 1.300194212 | 2.01E-20 | 2.73E-18 | 51 |
| hsa04810 Regulation of actin cytoskeleton | 0.048998803 | 1.27237948 | 5.85E-20 | 3.98E-18 | 91 |
| hsa04142 Lysosome | 0.084005127 | 1.204835267 | 3.40E-18 | 1.54E-16 | 74 |
| hsa04666 Fc gamma R-mediated phagocytosis | 0.061311067 | 1.166895344 | 6.26E-17 | 2.13E-15 | 51 |
| hsa04380 Osteoclast differentiation | 0.061344043 | 1.083941709 | 5.54E-15 | 1.51E-13 | 76 |
| hsa00030 Pentose phosphate pathway | 0.128313338 | 0.990613986 | 2.73E-12 | 6.18E-11 | 16 |
| hsa04664 Fc epsilon RI signaling pathway | 0.135860182 | 0.913638652 | 4.95E-11 | 9.62E-10 | 32 |
| hsa04145 Phagosome | 0.133686986 | 0.758400951 | 2.76E-08 | 4.69E-07 | 82 |
| hsa00010 Glycolysis / Gluconeogenesis | 0.20366167 | 0.739123308 | 6.59E-08 | 9.96E-07 | 35 |
| hsa00500 Starch and sucrose metabolism | 0.230093425 | 0.705126812 | 2.63E-07 | 3.58E-06 | 20 |
| hsa04966 Collecting duct acid secretion | 0.213661309 | 0.704455122 | 3.65E-07 | 4.51E-06 | 13 |
| hsa04650 Natural killer cell mediated cytotoxicity | 0.168056547 | 0.681314767 | 5.55E-07 | 6.29E-06 | 65 |
| hsa04976 Bile secretion | 0.228454012 | 0.676990106 | 7.91E-07 | 8.27E-06 | 19 |
| hsa00190 Oxidative phosphorylation | 0.154362526 | 0.668569919 | 9.97E-07 | 9.69E-06 | 57 |
| hsa00590 Arachidonic acid metabolism | 0.24434838 | 0.654543094 | 1.53E-06 | 1.39E-05 | 22 |
| hsa04662 B cell receptor signaling pathway | 0.154654558 | 0.651501074 | 1.75E-06 | 1.49E-05 | 48 |
| hsa04510 Focal adhesion | 0.188401339 | 0.641011783 | 2.16E-06 | 1.73E-05 | 77 |
| hsa04722 Neurotrophin signaling pathway | 0.21096455 | 0.636079059 | 2.56E-06 | 1.94E-05 | 62 |
| hsa04520 Adherens junction | 0.182161148 | 0.639337348 | 2.75E-06 | 1.97E-05 | 34 |
| hsa00531 Glycosaminoglycan degradation | 0.255402943 | 0.633352137 | 3.55E-06 | 2.42E-05 | 15 |
| hsa04910 Insulin signaling pathway | 0.253927411 | 0.602859056 | 7.45E-06 | 4.83E-05 | 64 |
| hsa00860 Porphyrin and chlorophyll metabolism | 0.232700362 | 0.62124119 | 7.93E-06 | 4.90E-05 | 12 |
| hsa00520 Amino sugar and nucleotide sugar metabolism | 0.269205274 | 0.598621439 | 9.98E-06 | 5.90E-05 | 24 |
| hsa04620 Toll-like receptor signaling pathway | 0.245260934 | 0.552207624 | 3.88E-05 | 0.000219832 | 46 |
| hsa00480 Glutathione metabolism | 0.273746242 | 0.547138149 | 5.02E-05 | 0.000273148 | 19 |
| hsa04210 Apoptosis | 0.253284213 | 0.523650063 | 8.82E-05 | 0.000461596 | 50 |
| hsa04971 Gastric acid secretion | 0.264769995 | 0.518522828 | 0.000109677 | 0.000552449 | 27 |
| hsa04920 Adipocytokine signaling pathway | 0.252463312 | 0.510432064 | 0.000131522 | 0.00063882 | 37 |
| hsa04270 Vascular smooth muscle contraction | 0.286367247 | 0.505650324 | 0.000148116 | 0.000694611 | 37 |
| hsa04062 Chemokine signaling pathway | 0.180036176 | 0.497523935 | 0.000179582 | 0.000814105 | 93 |
| hsa00770 Pantothenate and CoA biosynthesis | 0.29851147 | 0.489354291 | 0.000280391 | 0.001230102 | 11 |
| hsa00051 Fructose and mannose metabolism | 0.311024424 | 0.465713336 | 0.000453299 | 0.001926519 | 20 |
| hsa04530 Tight junction | 0.237591825 | 0.433256405 | 0.000986309 | 0.004064789 | 49 |
| hsa04730 Long-term depression | 0.285485043 | 0.427601115 | 0.001197421 | 0.004789683 | 19 |
| hsa00052 Galactose metabolism | 0.346320092 | 0.383642286 | 0.003168516 | 0.012311946 | 17 |
| hsa04962 Vasopressin-regulated water reabsorption | 0.318861612 | 0.374360219 | 0.003867763 | 0.01461155 | 20 |
| hsa03320 PPAR signaling pathway | 0.344239701 | 0.370554856 | 0.0040354 | 0.014832821 | 29 |
| hsa04622 RIG-I-like receptor signaling pathway | 0.334721092 | 0.367593145 | 0.004338377 | 0.015526822 | 24 |
| hsa00564 Glycerophospholipid metabolism | 0.353347659 | 0.349951851 | 0.006080495 | 0.021203777 | 40 |
| hsa00980 Metabolism of xenobiotics by cytochrome P450 | 0.347086045 | 0.344640139 | 0.007433866 | 0.025275145 | 12 |
| hsa04146 Peroxisome | 0.357941218 | 0.336681878 | 0.007852141 | 0.026046126 | 43 |
| hsa04010 MAPK signaling pathway | 0.303773303 | 0.326024378 | 0.009486739 | 0.030718964 | 116 |
| hsa04540 Gap junction | 0.35262357 | 0.306802879 | 0.014069983 | 0.044500412 | 33 |
| hsa00983 Drug metabolism - other enzymes | 0.369557312 | 0.307722038 | 0.014480895 | 0.044759131 | 16 |
| hsa00330 Arginine and proline metabolism | 0.369542922 | 0.296236364 | 0.017017578 | 0.051430903 | 25 |
| hsa04610 Complement and coagulation cascades | 0.332372122 | 0.293370135 | 0.018308377 | 0.054085628 | 25 |
| hsa00600 Sphingolipid metabolism | 0.382277084 | 0.291911068 | 0.018691357 | 0.054085628 | 23 |
| hsa04144 Endocytosis | 0.338752752 | 0.28779002 | 0.019277297 | 0.054619009 | 83 |
| hsa04260 Cardiac muscle contraction | 0.313122423 | 0.286526928 | 0.021167507 | 0.058750631 | 24 |
| hsa04115 p53 signaling pathway | 0.37829683 | 0.264931243 | 0.028799736 | 0.078335282 | 35 |
| hsa00760 Nicotinate and nicotinamide metabolism | 0.380364897 | 0.252157541 | 0.037371028 | 0.099656075 | 11 |
| hsa00512 Mucin type O-Glycan biosynthesis | 0.398577935 | 0.246671028 | 0.040446026 | 0.105781914 | 11 |
| hsa00982 Drug metabolism - cytochrome P450 | 0.400001444 | 0.206047237 | 0.073351914 | 0.188223778 | 10 |
| hsa04370 VEGF signaling pathway | 0.386378166 | 0.192329988 | 0.083921656 | 0.20857907 | 35 |
| hsa04973 Carbohydrate digestion and absorption | 0.418162638 | 0.193062855 | 0.08435183 | 0.20857907 | 18 |
| hsa00511 Other glycan degradation | 0.419016152 | 0.182453464 | 0.099354195 | 0.241288759 | 11 |
| hsa04975 Fat digestion and absorption | 0.438299 | 0.144159942 | 0.1534701 | 0.366174274 | 14 |
| hsa04912 GnRH signaling pathway | 0.451066488 | 0.105164164 | 0.225725679 | 0.5292878 | 34 |
| hsa04360 Axon guidance | 0.356112742 | 0.093695189 | 0.249742443 | 0.575677495 | 58 |
| hsa04630 Jak-STAT signaling pathway | 0.369759818 | 0.077546745 | 0.287398899 | 0.638999628 | 64 |
| hsa04972 Pancreatic secretion | 0.460986032 | 0.078237041 | 0.28776535 | 0.638999628 | 29 |
| hsa04621 NOD-like receptor signaling pathway | 0.417932398 | 0.076897348 | 0.291308654 | 0.638999628 | 33 |
| hsa00071 Fatty acid metabolism | 0.462888562 | 0.068292168 | 0.312546346 | 0.674703223 | 22 |
| hsa02010 ABC transporters | 0.472205352 | 0.053002746 | 0.353234104 | 0.750622471 | 13 |
| hsa00562 Inositol phosphate metabolism | 0.464587509 | 0.044131808 | 0.376372083 | 0.773007771 | 23 |
| hsa04130 SNARE interactions in vesicular transport | 0.46712152 | 0.044084385 | 0.376543896 | 0.773007771 | 22 |
| hsa04974 Protein digestion and absorption | 0.476241193 | 0.042573966 | 0.380820005 | 0.773007771 | 21 |
| hsa04150 mTOR signaling pathway | 0.461384546 | 0.02970994 | 0.415831959 | 0.831663918 | 25 |
| hsa00532 Glycosaminoglycan biosynthesis - chondroitin sulfate | 0.475815539 | 0.022498477 | 0.436063778 | 0.859488026 | 14 |
| hsa04114 Oocyte meiosis | 0.452100421 | 0.014563241 | 0.458823796 | 0.891429089 | 53 |
| hsa04960 Aldosterone-regulated sodium reabsorption | 0.4899527 | 0.006472858 | 0.481575601 | 0.915739302 | 13 |
| hsa00640 Propanoate metabolism | 0.470580566 | 0.001655696 | 0.495325236 | 0.915739302 | 18 |
| hsa01040 Biosynthesis of unsaturated fatty acids | 0.49627936 | 0.000527438 | 0.498599457 | 0.915739302 | 13 |
| hsa00380 Tryptophan metabolism | 0.489786822 | -0.000565913 | 0.501391975 | 0.915739302 | 17 |
| hsa00450 Selenocompound metabolism | 0.497639791 | -0.001773829 | 0.505003291 | 0.915739302 | 12 |
| hsa00601 Glycosphingolipid biosynthesis - lacto and neolacto series | 0.501935428 | -0.010456592 | 0.529230933 | 0.938909975 | 10 |
| hsa04623 Cytosolic DNA-sensing pathway | 0.446327511 | -0.011807675 | 0.53436575 | 0.938909975 | 22 |
| hsa04310 Wnt signaling pathway | 0.431892011 | -0.013720991 | 0.538492486 | 0.938909975 | 70 |
| hsa04320 Dorso-ventral axis formation | 0.498647009 | -0.023521742 | 0.566589646 | 0.965592759 | 11 |
| hsa00565 Ether lipid metabolism | 0.504964205 | -0.026166305 | 0.573383612 | 0.965592759 | 15 |
| hsa04914 Progesterone-mediated oocyte maturation | 0.498039317 | -0.028208371 | 0.580086656 | 0.965592759 | 42 |
| hsa04640 Hematopoietic cell lineage | 0.457126988 | -0.029081356 | 0.582195634 | 0.965592759 | 56 |
| hsa00604 Glycosphingolipid biosynthesis - ganglio series | 0.515383567 | -0.055600076 | 0.651622018 | 1 | 11 |
| hsa04110 Cell cycle | 0.469470205 | -0.067187099 | 0.68558538 | 1 | 70 |
| hsa00310 Lysine degradation | 0.518944513 | -0.071687324 | 0.695507903 | 1 | 23 |
| hsa00240 Pyrimidine metabolism | 0.511212106 | -0.077453642 | 0.710719124 | 1 | 50 |
| hsa04710 Circadian rhythm - mammal | 0.517044399 | -0.082909105 | 0.719708411 | 1 | 13 |
| hsa04512 ECM-receptor interaction | 0.520742941 | -0.081874779 | 0.720578404 | 1 | 27 |
| hsa00260 Glycine, serine and threonine metabolism | 0.521665791 | -0.08724246 | 0.73171996 | 1 | 16 |
| hsa00561 Glycerolipid metabolism | 0.529267397 | -0.091483179 | 0.743411647 | 1 | 27 |
| hsa00830 Retinol metabolism | 0.531488923 | -0.095501208 | 0.74959857 | 1 | 13 |
| hsa04012 ErbB signaling pathway | 0.531334814 | -0.108659929 | 0.781558867 | 1 | 36 |
| hsa04916 Melanogenesis | 0.536788558 | -0.116275365 | 0.79776179 | 1 | 43 |
| hsa04720 Long-term potentiation | 0.539812588 | -0.122464462 | 0.808898973 | 1 | 25 |
| hsa03022 Basal transcription factors | 0.546697669 | -0.133308704 | 0.826859015 | 1 | 14 |
| hsa04740 Olfactory transduction | 0.546439763 | -0.134106761 | 0.831112177 | 1 | 31 |
| hsa00534 Glycosaminoglycan biosynthesis - heparan sulfate | 0.553232201 | -0.143383745 | 0.844185555 | 1 | 13 |
| hsa03050 Proteasome | 0.375344809 | -0.141609659 | 0.850481622 | 1 | 23 |
| hsa00270 Cysteine and methionine metabolism | 0.513154296 | -0.151542624 | 0.859221979 | 1 | 18 |
| hsa04070 Phosphatidylinositol signaling system | 0.541313547 | -0.1573775 | 0.869816489 | 1 | 32 |
| hsa04742 Taste transduction | 0.566138028 | -0.179815313 | 0.897661298 | 1 | 13 |
| hsa00563 Glycosylphosphatidylinositol(GPI)-anchor biosynthesis | 0.565878474 | -0.181485207 | 0.898275033 | 1 | 10 |
| hsa00350 Tyrosine metabolism | 0.556096966 | -0.188749709 | 0.90935875 | 1 | 17 |
| hsa03060 Protein export | 0.516360908 | -0.198999719 | 0.920339465 | 1 | 14 |
| hsa00650 Butanoate metabolism | 0.566745718 | -0.207254297 | 0.927911669 | 1 | 13 |
| hsa04141 Protein processing in endoplasmic reticulum | 0.531407778 | -0.206011985 | 0.930676734 | 1 | 75 |
| hsa04020 Calcium signaling pathway | 0.570299867 | -0.215597936 | 0.939172555 | 1 | 58 |
| hsa04970 Salivary secretion | 0.526780031 | -0.225270502 | 0.946310651 | 1 | 23 |
| hsa04340 Hedgehog signaling pathway | 0.583940654 | -0.228833175 | 0.947791429 | 1 | 17 |
| hsa00280 Valine, leucine and isoleucine degradation | 0.579311776 | -0.235482498 | 0.953612291 | 1 | 28 |
| hsa00340 Histidine metabolism | 0.582973922 | -0.243246524 | 0.95705023 | 1 | 13 |
| hsa03020 RNA polymerase | 0.580728054 | -0.248585184 | 0.959928142 | 1 | 13 |
| hsa03420 Nucleotide excision repair | 0.554329955 | -0.252428913 | 0.96352221 | 1 | 24 |
| hsa00620 Pyruvate metabolism | 0.544684903 | -0.263946904 | 0.970046305 | 1 | 22 |
| hsa04120 Ubiquitin mediated proteolysis | 0.552684596 | -0.272689134 | 0.974702814 | 1 | 62 |
| hsa03410 Base excision repair | 0.575019955 | -0.275968942 | 0.974751055 | 1 | 19 |
| hsa00020 Citrate cycle (TCA cycle) | 0.563113544 | -0.283547069 | 0.977726892 | 1 | 16 |
| hsa04330 Notch signaling pathway | 0.598547966 | -0.285039521 | 0.978917046 | 1 | 23 |
| hsa03440 Homologous recombination | 0.609275818 | -0.308974306 | 0.984705069 | 1 | 10 |
| hsa00510 N-Glycan biosynthesis | 0.604102455 | -0.304665233 | 0.984745201 | 1 | 20 |
| hsa03430 Mismatch repair | 0.610380943 | -0.314208095 | 0.986416668 | 1 | 12 |
| hsa03030 DNA replication | 0.595469255 | -0.343968193 | 0.992555544 | 1 | 19 |
| hsa03008 Ribosome biogenesis in eukaryotes | 0.593444661 | -0.388030206 | 0.99722464 | 1 | 38 |
| hsa04350 TGF-beta signaling pathway | 0.619756171 | -0.392880015 | 0.997558426 | 1 | 41 |
| hsa00970 Aminoacyl-tRNA biosynthesis | 0.622543257 | -0.422848601 | 0.998675768 | 1 | 24 |
| hsa00230 Purine metabolism | 0.632716287 | -0.450409799 | 0.999391342 | 1 | 74 |
| hsa00514 Other types of O-glycan biosynthesis | 0.665157296 | -0.472557863 | 0.99958388 | 1 | 15 |
| hsa03015 mRNA surveillance pathway | 0.675444585 | -0.545651016 | 0.999951037 | 1 | 33 |
| hsa04660 T cell receptor signaling pathway | 0.66128095 | -0.605689364 | 0.999993102 | 1 | 68 |
| hsa03018 RNA degradation | 0.67967229 | -0.646287141 | 0.999998053 | 1 | 35 |
| hsa04514 Cell adhesion molecules (CAMs) | 0.639242597 | -0.707047442 | 0.999999778 | 1 | 57 |
| hsa03013 RNA transport | 0.679393562 | -1.044284155 | 1 | 1 | 69 |
| hsa03040 Spliceosome | 0.748589683 | -1.058781283 | 1 | 1 | 76 |
| hsa03010 Ribosome | 0.320420663 | -4.234576248 | 1 | 1 | 50 |
| hsa04612 Antigen processing and presentation | 0.743070708 | -1.247614409 | 1 | 1 | 48 |
| hsa04672 Intestinal immune network for IgA production | 0.83971879 | -1.555921998 | 1 | 1 | 29 |
| hsa00040 Pentose and glucuronate interconversions | NA | NA | NA | NA | 8 |
| hsa00053 Ascorbate and aldarate metabolism | NA | NA | NA | NA | 3 |
| hsa00061 Fatty acid biosynthesis | NA | NA | NA | NA | 4 |
| hsa00072 Synthesis and degradation of ketone bodies | NA | NA | NA | NA | 4 |
| hsa00100 Steroid biosynthesis | NA | NA | NA | NA | 6 |
| hsa00120 Primary bile acid biosynthesis | NA | NA | NA | NA | 5 |
| hsa00130 Ubiquinone and other terpenoid-quinone biosynthesis | NA | NA | NA | NA | 4 |
| hsa00140 Steroid hormone biosynthesis | NA | NA | NA | NA | 7 |
| hsa00232 Caffeine metabolism | NA | NA | NA | NA | 1 |
| hsa00250 Alanine, aspartate and glutamate metabolism | NA | NA | NA | NA | 8 |
| hsa00290 Valine, leucine and isoleucine biosynthesis | NA | NA | NA | NA | 8 |
| hsa00300 Lysine biosynthesis | NA | NA | NA | NA | 0 |
| hsa00360 Phenylalanine metabolism | NA | NA | NA | NA | 6 |
| hsa00400 Phenylalanine, tyrosine and tryptophan biosynthesis | NA | NA | NA | NA | 2 |
| hsa00410 beta-Alanine metabolism | NA | NA | NA | NA | 9 |
| hsa00430 Taurine and hypotaurine metabolism | NA | NA | NA | NA | 3 |
| hsa00460 Cyanoamino acid metabolism | NA | NA | NA | NA | 3 |
| hsa00471 D-Glutamine and D-glutamate metabolism | NA | NA | NA | NA | 1 |
| hsa00472 D-Arginine and D-ornithine metabolism | NA | NA | NA | NA | 1 |
| hsa00533 Glycosaminoglycan biosynthesis - keratan sulfate | NA | NA | NA | NA | 6 |
| hsa00591 Linoleic acid metabolism | NA | NA | NA | NA | 5 |
| hsa00592 alpha-Linolenic acid metabolism | NA | NA | NA | NA | 3 |
| hsa00603 Glycosphingolipid biosynthesis - globo series | NA | NA | NA | NA | 9 |
| hsa00630 Glyoxylate and dicarboxylate metabolism | NA | NA | NA | NA | 6 |
| hsa00670 One carbon pool by folate | NA | NA | NA | NA | 8 |
| hsa00730 Thiamine metabolism | NA | NA | NA | NA | 2 |
| hsa00740 Riboflavin metabolism | NA | NA | NA | NA | 5 |
| hsa00750 Vitamin B6 metabolism | NA | NA | NA | NA | 4 |
| hsa00780 Biotin metabolism | NA | NA | NA | NA | 1 |
| hsa00785 Lipoic acid metabolism | NA | NA | NA | NA | 2 |
| hsa00790 Folate biosynthesis | NA | NA | NA | NA | 4 |
| hsa00900 Terpenoid backbone biosynthesis | NA | NA | NA | NA | 6 |
| hsa00910 Nitrogen metabolism | NA | NA | NA | NA | 7 |
| hsa00920 Sulfur metabolism | NA | NA | NA | NA | 8 |
| hsa03450 Non-homologous end-joining | NA | NA | NA | NA | 5 |
| hsa04122 Sulfur relay system | NA | NA | NA | NA | 5 |
| hsa04140 Regulation of autophagy | NA | NA | NA | NA | 8 |
| hsa04614 Renin-angiotensin system | NA | NA | NA | NA | 7 |
| hsa04744 Phototransduction | NA | NA | NA | NA | 7 |
| hsa04964 Proximal tubule bicarbonate reclamation | NA | NA | NA | NA | 4 |
| hsa04977 Vitamin digestion and absorption | NA | NA | NA | NA | 6 |
| hsa03010 Ribosome | 9.17E-07 | -4.234576248 | 5.32E-154 | 7.24E-152 | 50 |
| hsa04672 Intestinal immune network for IgA production | 0.04881873 | -1.555921998 | 3.72E-28 | 2.53E-26 | 29 |
| hsa04612 Antigen processing and presentation | 0.073141503 | -1.247614409 | 4.33E-19 | 1.96E-17 | 48 |
| hsa03040 Spliceosome | 0.118116448 | -1.058781283 | 1.87E-14 | 6.36E-13 | 76 |
| hsa03013 RNA transport | 0.104722919 | -1.044284155 | 4.33E-14 | 1.18E-12 | 69 |
| hsa04514 Cell adhesion molecules (CAMs) | 0.181617266 | -0.707047442 | 2.22E-07 | 5.03E-06 | 57 |
| hsa03018 RNA degradation | 0.232682495 | -0.646287141 | 1.95E-06 | 3.78E-05 | 35 |
| hsa04660 T cell receptor signaling pathway | 0.239198359 | -0.605689364 | 6.90E-06 | 0.000117272 | 68 |
| hsa03015 mRNA surveillance pathway | 0.278293382 | -0.545651016 | 4.90E-05 | 0.000739888 | 33 |
| hsa00514 Other types of O-glycan biosynthesis | 0.313532887 | -0.472557863 | 0.00041612 | 0.005659234 | 15 |
| hsa00230 Purine metabolism | 0.303521584 | -0.450409799 | 0.000608658 | 0.007525229 | 74 |
| hsa00970 Aminoacyl-tRNA biosynthesis | 0.315071711 | -0.422848601 | 0.001324232 | 0.015007958 | 24 |
| hsa04350 TGF-beta signaling pathway | 0.327643526 | -0.392880015 | 0.002441574 | 0.025542624 | 41 |
| hsa03008 Ribosome biogenesis in eukaryotes | 0.315995839 | -0.388030206 | 0.00277536 | 0.026960636 | 38 |
| hsa03030 DNA replication | 0.343720855 | -0.343968193 | 0.007444456 | 0.067496399 | 19 |
| hsa03430 Mismatch repair | 0.372674369 | -0.314208095 | 0.013583332 | 0.115458325 | 12 |
| hsa00510 N-Glycan biosynthesis | 0.372324425 | -0.304665233 | 0.015254799 | 0.115561699 | 20 |
| hsa03440 Homologous recombination | 0.376001956 | -0.308974306 | 0.015294931 | 0.115561699 | 10 |
| hsa04330 Notch signaling pathway | 0.379533174 | -0.285039521 | 0.021082954 | 0.150909564 | 23 |
| hsa00020 Citrate cycle (TCA cycle) | 0.358399472 | -0.283547069 | 0.022273108 | 0.151457134 | 16 |
| hsa03410 Base excision repair | 0.370392003 | -0.275968942 | 0.025248945 | 0.156382607 | 19 |
| hsa04120 Ubiquitin mediated proteolysis | 0.355060165 | -0.272689134 | 0.025297186 | 0.156382607 | 62 |
| hsa00620 Pyruvate metabolism | 0.35635919 | -0.263946904 | 0.029953695 | 0.177117501 | 22 |
| hsa03420 Nucleotide excision repair | 0.36979153 | -0.252428913 | 0.03647779 | 0.206707474 | 24 |
| hsa03020 RNA polymerase | 0.392774662 | -0.248585184 | 0.040071858 | 0.217990907 | 13 |
| hsa00340 Histidine metabolism | 0.397288327 | -0.243246524 | 0.04294977 | 0.224660335 | 13 |
| hsa00280 Valine, leucine and isoleucine degradation | 0.398078308 | -0.235482498 | 0.046387709 | 0.233656608 | 28 |
| hsa04340 Hedgehog signaling pathway | 0.406776113 | -0.228833175 | 0.052208571 | 0.251784533 | 17 |
| hsa04970 Salivary secretion | 0.366581271 | -0.225270502 | 0.053689349 | 0.251784533 | 23 |
| hsa04020 Calcium signaling pathway | 0.403703432 | -0.215597936 | 0.060827445 | 0.275751086 | 58 |
| hsa04141 Protein processing in endoplasmic reticulum | 0.381702908 | -0.206011985 | 0.069323266 | 0.304127877 | 75 |
| hsa00650 Butanoate metabolism | 0.409377434 | -0.207254297 | 0.072088331 | 0.306375407 | 13 |
| hsa03060 Protein export | 0.377675947 | -0.198999719 | 0.079660535 | 0.328297964 | 14 |
| hsa00350 Tyrosine metabolism | 0.412770751 | -0.188749709 | 0.09064125 | 0.362565001 | 17 |
| hsa00563 Glycosylphosphatidylinositol(GPI)-anchor biosynthesis | 0.426613556 | -0.181485207 | 0.101724967 | 0.386612873 | 10 |
| hsa04742 Taste transduction | 0.427173797 | -0.179815313 | 0.102338702 | 0.386612873 | 13 |
| hsa04070 Phosphatidylinositol signaling system | 0.420503382 | -0.1573775 | 0.130183511 | 0.478512363 | 32 |
| hsa00270 Cysteine and methionine metabolism | 0.403625067 | -0.151542624 | 0.140778021 | 0.503837127 | 18 |
| hsa03050 Proteasome | 0.30919273 | -0.141609659 | 0.149518378 | 0.521397421 | 23 |
| hsa00534 Glycosaminoglycan biosynthesis - heparan sulfate | 0.44199671 | -0.143383745 | 0.155814445 | 0.529769115 | 13 |
| hsa04740 Olfactory transduction | 0.441576533 | -0.134106761 | 0.168887823 | 0.560213266 | 31 |
| hsa03022 Basal transcription factors | 0.443549634 | -0.133308704 | 0.173140985 | 0.560646998 | 14 |
| hsa04720 Long-term potentiation | 0.444469694 | -0.122464462 | 0.191101027 | 0.604412552 | 25 |
| hsa04916 Melanogenesis | 0.445697524 | -0.116275365 | 0.20223821 | 0.625099923 | 43 |
| hsa04012 ErbB signaling pathway | 0.446963275 | -0.108659929 | 0.218441133 | 0.660177645 | 36 |
| hsa00830 Retinol metabolism | 0.457717745 | -0.095501208 | 0.25040143 | 0.740317271 | 13 |
| hsa00561 Glycerolipid metabolism | 0.4576984 | -0.091483179 | 0.256588353 | 0.742468426 | 27 |
| hsa00260 Glycine, serine and threonine metabolism | 0.455112992 | -0.08724246 | 0.26828004 | 0.760126779 | 16 |
| hsa04512 ECM-receptor interaction | 0.457427865 | -0.081874779 | 0.279421596 | 0.762393123 | 27 |
| hsa04710 Circadian rhythm - mammal | 0.45427317 | -0.082909105 | 0.280291589 | 0.762393123 | 13 |
| hsa00240 Pyrimidine metabolism | 0.451698641 | -0.077453642 | 0.289280876 | 0.77141567 | 50 |
| hsa00310 Lysine degradation | 0.463147847 | -0.071687324 | 0.304492097 | 0.796363946 | 23 |
| hsa04110 Cell cycle | 0.424928972 | -0.067187099 | 0.31441462 | 0.80679978 | 70 |
| hsa00604 Glycosphingolipid biosynthesis - ganglio series | 0.472727794 | -0.055600076 | 0.348377982 | 0.8773964 | 11 |
| hsa04640 Hematopoietic cell lineage | 0.432301457 | -0.029081356 | 0.417804366 | 1 | 56 |
| hsa04914 Progesterone-mediated oocyte maturation | 0.476273347 | -0.028208371 | 0.419913344 | 1 | 42 |
| hsa00565 Ether lipid metabolism | 0.484641547 | -0.026166305 | 0.426616388 | 1 | 15 |
| hsa04320 Dorso-ventral axis formation | 0.480447046 | -0.023521742 | 0.433410354 | 1 | 11 |
| hsa04310 Wnt signaling pathway | 0.419394903 | -0.013720991 | 0.461507514 | 1 | 70 |
| hsa04623 Cytosolic DNA-sensing pathway | 0.438963293 | -0.011807675 | 0.46563425 | 1 | 22 |
| hsa00601 Glycosphingolipid biosynthesis - lacto and neolacto series | 0.493861413 | -0.010456592 | 0.470769067 | 1 | 10 |
| hsa00450 Selenocompound metabolism | 0.49627572 | -0.001773829 | 0.494996709 | 1 | 12 |
| hsa00380 Tryptophan metabolism | 0.489696095 | -0.000565913 | 0.498608025 | 1 | 17 |
| hsa01040 Biosynthesis of unsaturated fatty acids | 0.496729691 | 0.000527438 | 0.501400543 | 1 | 13 |
| hsa00640 Propanoate metabolism | 0.473172547 | 0.001655696 | 0.504674764 | 1 | 18 |
| hsa04960 Aldosterone-regulated sodium reabsorption | 0.495021092 | 0.006472858 | 0.518424399 | 1 | 13 |
| hsa04114 Oocyte meiosis | 0.466606892 | 0.014563241 | 0.541176204 | 1 | 53 |
| hsa00532 Glycosaminoglycan biosynthesis - chondroitin sulfate | 0.49316074 | 0.022498477 | 0.563936222 | 1 | 14 |
| hsa04150 mTOR signaling pathway | 0.483898981 | 0.02970994 | 0.584168041 | 1 | 25 |
| hsa04974 Protein digestion and absorption | 0.509542926 | 0.042573966 | 0.619179995 | 1 | 21 |
| hsa04130 SNARE interactions in vesicular transport | 0.501382719 | 0.044084385 | 0.623456104 | 1 | 22 |
| hsa00562 Inositol phosphate metabolism | 0.498536373 | 0.044131808 | 0.623627917 | 1 | 23 |
| hsa02010 ABC transporters | 0.513567334 | 0.053002746 | 0.646765896 | 1 | 13 |
| hsa00071 Fatty acid metabolism | 0.516120141 | 0.068292168 | 0.687453654 | 1 | 22 |
| hsa04621 NOD-like receptor signaling pathway | 0.474619639 | 0.076897348 | 0.708691346 | 1 | 33 |
| hsa04972 Pancreatic secretion | 0.522192854 | 0.078237041 | 0.71223465 | 1 | 29 |
| hsa04630 Jak-STAT signaling pathway | 0.41328336 | 0.077546745 | 0.712601101 | 1 | 64 |
| hsa04360 Axon guidance | 0.413139664 | 0.093695189 | 0.750257557 | 1 | 58 |
| hsa04912 GnRH signaling pathway | 0.533390471 | 0.105164164 | 0.774274321 | 1 | 34 |
| hsa04975 Fat digestion and absorption | 0.550249731 | 0.144159942 | 0.8465299 | 1 | 14 |
| hsa00511 Other glycan degradation | 0.55853026 | 0.182453464 | 0.900645805 | 1 | 11 |
| hsa04973 Carbohydrate digestion and absorption | 0.568165781 | 0.193062855 | 0.91564817 | 1 | 18 |
| hsa04370 VEGF signaling pathway | 0.527348675 | 0.192329988 | 0.916078344 | 1 | 35 |
| hsa00982 Drug metabolism - cytochrome P450 | 0.55530716 | 0.206047237 | 0.926648086 | 1 | 10 |
| hsa00512 Mucin type O-Glycan biosynthesis | 0.588221709 | 0.246671028 | 0.959553974 | 1 | 11 |
| hsa00760 Nicotinate and nicotinamide metabolism | 0.567379195 | 0.252157541 | 0.962628972 | 1 | 11 |
| hsa04115 p53 signaling pathway | 0.58016617 | 0.264931243 | 0.971200264 | 1 | 35 |
| hsa04260 Cardiac muscle contraction | 0.508816415 | 0.286526928 | 0.978832493 | 1 | 24 |
| hsa04144 Endocytosis | 0.543355757 | 0.28779002 | 0.980722703 | 1 | 83 |
| hsa00600 Sphingolipid metabolism | 0.608181201 | 0.291911068 | 0.981308643 | 1 | 23 |
| hsa04610 Complement and coagulation cascades | 0.541208764 | 0.293370135 | 0.981691623 | 1 | 25 |
| hsa00330 Arginine and proline metabolism | 0.595473579 | 0.296236364 | 0.982982422 | 1 | 25 |
| hsa00983 Drug metabolism - other enzymes | 0.603809476 | 0.307722038 | 0.985519105 | 1 | 16 |
| hsa04540 Gap junction | 0.579858035 | 0.306802879 | 0.985930017 | 1 | 33 |
| hsa04010 MAPK signaling pathway | 0.520784553 | 0.326024378 | 0.990513261 | 1 | 116 |
| hsa04146 Peroxisome | 0.615680193 | 0.336681878 | 0.992147859 | 1 | 43 |
| hsa00980 Metabolism of xenobiotics by cytochrome P450 | 0.603305618 | 0.344640139 | 0.992566134 | 1 | 12 |
| hsa00564 Glycerophospholipid metabolism | 0.620620284 | 0.349951851 | 0.993919505 | 1 | 40 |
| hsa04622 RIG-I-like receptor signaling pathway | 0.606541439 | 0.367593145 | 0.995661623 | 1 | 24 |
| hsa03320 PPAR signaling pathway | 0.624806784 | 0.370554856 | 0.9959646 | 1 | 29 |
| hsa04962 Vasopressin-regulated water reabsorption | 0.584585048 | 0.374360219 | 0.996132237 | 1 | 20 |
| hsa00052 Galactose metabolism | 0.63845918 | 0.383642286 | 0.996831484 | 1 | 17 |
| hsa04730 Long-term depression | 0.573727796 | 0.427601115 | 0.998802579 | 1 | 19 |
| hsa04530 Tight junction | 0.498026244 | 0.433256405 | 0.999013691 | 1 | 49 |
| hsa00051 Fructose and mannose metabolism | 0.658391143 | 0.465713336 | 0.999546701 | 1 | 20 |
| hsa00770 Pantothenate and CoA biosynthesis | 0.653033847 | 0.489354291 | 0.999719609 | 1 | 11 |
| hsa04062 Chemokine signaling pathway | 0.43477825 | 0.497523935 | 0.999820418 | 1 | 93 |
| hsa04270 Vascular smooth muscle contraction | 0.653822166 | 0.505650324 | 0.999851884 | 1 | 37 |
| hsa04920 Adipocytokine signaling pathway | 0.592032085 | 0.510432064 | 0.999868478 | 1 | 37 |
| hsa04971 Gastric acid secretion | 0.618065663 | 0.518522828 | 0.999890323 | 1 | 27 |
| hsa04210 Apoptosis | 0.610866122 | 0.523650063 | 0.999911754 | 1 | 50 |
| hsa00480 Glutathione metabolism | 0.669833063 | 0.547138149 | 0.999949789 | 1 | 19 |
| hsa04620 Toll-like receptor signaling pathway | 0.617618464 | 0.552207624 | 0.999961206 | 1 | 46 |
| hsa00520 Amino sugar and nucleotide sugar metabolism | 0.708634695 | 0.598621439 | 0.999990023 | 1 | 24 |
| hsa00860 Porphyrin and chlorophyll metabolism | 0.631675707 | 0.62124119 | 0.999992068 | 1 | 12 |
| hsa04910 Insulin signaling pathway | 0.687013218 | 0.602859056 | 0.999992549 | 1 | 64 |
| hsa00531 Glycosaminoglycan degradation | 0.711055963 | 0.633352137 | 0.999996447 | 1 | 15 |
| hsa04520 Adherens junction | 0.537875253 | 0.639337348 | 0.999997254 | 1 | 34 |
| hsa04722 Neurotrophin signaling pathway | 0.620863687 | 0.636079059 | 0.999997438 | 1 | 62 |
| hsa04510 Focal adhesion | 0.570032152 | 0.641011783 | 0.99999784 | 1 | 77 |
| hsa04662 B cell receptor signaling pathway | 0.482772868 | 0.651501074 | 0.999998246 | 1 | 48 |
| hsa00590 Arachidonic acid metabolism | 0.71040965 | 0.654543094 | 0.999998467 | 1 | 22 |
| hsa00190 Oxidative phosphorylation | 0.54379846 | 0.668569919 | 0.999999003 | 1 | 57 |
| hsa04976 Bile secretion | 0.691975954 | 0.676990106 | 0.999999209 | 1 | 19 |
| hsa04650 Natural killer cell mediated cytotoxicity | 0.539077201 | 0.681314767 | 0.999999445 | 1 | 65 |
| hsa04966 Collecting duct acid secretion | 0.677815621 | 0.704455122 | 0.999999635 | 1 | 13 |
| hsa00500 Starch and sucrose metabolism | 0.728003334 | 0.705126812 | 0.999999737 | 1 | 20 |
| hsa00010 Glycolysis / Gluconeogenesis | 0.708824854 | 0.739123308 | 0.999999934 | 1 | 35 |
| hsa04145 Phagosome | 0.494910222 | 0.758400951 | 0.999999972 | 1 | 82 |
| hsa04664 Fc epsilon RI signaling pathway | 0.654002099 | 0.913638652 | 1 | 1 | 32 |
| hsa00030 Pentose phosphate pathway | 0.689858521 | 0.990613986 | 1 | 1 | 16 |
| hsa04380 Osteoclast differentiation | 0.480740605 | 1.083941709 | 1 | 1 | 76 |
| hsa04666 Fc gamma R-mediated phagocytosis | 0.559549688 | 1.166895344 | 1 | 1 | 51 |
| hsa04142 Lysosome | 0.745016658 | 1.204835267 | 1 | 1 | 74 |
| hsa04670 Leukocyte transendothelial migration | 0.612557078 | 1.300194212 | 1 | 1 | 51 |
| hsa04810 Regulation of actin cytoskeleton | 0.560717439 | 1.27237948 | 1 | 1 | 91 |
| hsa00040 Pentose and glucuronate interconversions | NA | NA | NA | NA | 8 |
| hsa00053 Ascorbate and aldarate metabolism | NA | NA | NA | NA | 3 |
| hsa00061 Fatty acid biosynthesis | NA | NA | NA | NA | 4 |
| hsa00072 Synthesis and degradation of ketone bodies | NA | NA | NA | NA | 4 |
| hsa00100 Steroid biosynthesis | NA | NA | NA | NA | 6 |
| hsa00120 Primary bile acid biosynthesis | NA | NA | NA | NA | 5 |
| hsa00130 Ubiquinone and other terpenoid-quinone biosynthesis | NA | NA | NA | NA | 4 |
| hsa00140 Steroid hormone biosynthesis | NA | NA | NA | NA | 7 |
| hsa00232 Caffeine metabolism | NA | NA | NA | NA | 1 |
| hsa00250 Alanine, aspartate and glutamate metabolism | NA | NA | NA | NA | 8 |
| hsa00290 Valine, leucine and isoleucine biosynthesis | NA | NA | NA | NA | 8 |
| hsa00300 Lysine biosynthesis | NA | NA | NA | NA | 0 |
| hsa00360 Phenylalanine metabolism | NA | NA | NA | NA | 6 |
| hsa00400 Phenylalanine, tyrosine and tryptophan biosynthesis | NA | NA | NA | NA | 2 |
| hsa00410 beta-Alanine metabolism | NA | NA | NA | NA | 9 |
| hsa00430 Taurine and hypotaurine metabolism | NA | NA | NA | NA | 3 |
| hsa00460 Cyanoamino acid metabolism | NA | NA | NA | NA | 3 |
| hsa00471 D-Glutamine and D-glutamate metabolism | NA | NA | NA | NA | 1 |
| hsa00472 D-Arginine and D-ornithine metabolism | NA | NA | NA | NA | 1 |
| hsa00533 Glycosaminoglycan biosynthesis - keratan sulfate | NA | NA | NA | NA | 6 |
| hsa00591 Linoleic acid metabolism | NA | NA | NA | NA | 5 |
| hsa00592 alpha-Linolenic acid metabolism | NA | NA | NA | NA | 3 |
| hsa00603 Glycosphingolipid biosynthesis - globo series | NA | NA | NA | NA | 9 |
| hsa00630 Glyoxylate and dicarboxylate metabolism | NA | NA | NA | NA | 6 |
| hsa00670 One carbon pool by folate | NA | NA | NA | NA | 8 |
| hsa00730 Thiamine metabolism | NA | NA | NA | NA | 2 |
| hsa00740 Riboflavin metabolism | NA | NA | NA | NA | 5 |
| hsa00750 Vitamin B6 metabolism | NA | NA | NA | NA | 4 |
| hsa00780 Biotin metabolism | NA | NA | NA | NA | 1 |
| hsa00785 Lipoic acid metabolism | NA | NA | NA | NA | 2 |
| hsa00790 Folate biosynthesis | NA | NA | NA | NA | 4 |
| hsa00900 Terpenoid backbone biosynthesis | NA | NA | NA | NA | 6 |
| hsa00910 Nitrogen metabolism | NA | NA | NA | NA | 7 |
| hsa00920 Sulfur metabolism | NA | NA | NA | NA | 8 |
| hsa03450 Non-homologous end-joining | NA | NA | NA | NA | 5 |
| hsa04122 Sulfur relay system | NA | NA | NA | NA | 5 |
| hsa04140 Regulation of autophagy | NA | NA | NA | NA | 8 |
| hsa04614 Renin-angiotensin system | NA | NA | NA | NA | 7 |
| hsa04744 Phototransduction | NA | NA | NA | NA | 7 |
| hsa04964 Proximal tubule bicarbonate reclamation | NA | NA | NA | NA | 4 |
| hsa04977 Vitamin digestion and absorption | NA | NA | NA | NA | 6 |
