# Supplementary material for: Immune gene expression networks in sepsis: A network biology approach
Source: PLoS One. 2021 Mar 5;16(3):e0247669. doi: 10.1371/journal.pone.0247669 (PMC7935325; doi:10.1371/journal.pone.0247669)
Supplement: Attachment — Submitted filename: Comments.to.reiewer.docx [file pone.0247669.s003.docx]

**ACADEMIC EDITOR #**

**Reviewer 1 still has major concerns with the structure of the manuscript. As recommended by Reviewer 1, the global transcriptomic analysis should be presented first, and the other findings should then be contextualized with regard to these systems analyses. I concur with the reviewer's suggestions, and recommend a complete re-ordering of the manuscript rather than minor changes to a few sentences**.

**Reviewer #1: The authors have not addressed my underlying critique, which is that the paper needs to be rewritten to reflect the complete data analysis initially, and then discuss why they focused on only the immune-related genes.**

--> Thank you for your kind comments.

We have re-orderded the manuscript as commented by academic editor and Reviewer. The global transcriptomic analysis was presented first, followed by other analysis.

Correction 1. Following sentence was revised.

In this study, gene expression signatures from a public database, Gene Expression Omnibus (GEO), were analyzed. We selected 998 immune-related genes included in immune response pathways using gene expression profiles to construct a network for the healthy control and sepsis groups. Total transcriptome was analyzed for differentially expressed genes (DEGs) between healthy control and sepsis groups. We performed gene set enrichment analysis for DEGs, which revealed up- or down-regulated pathways.

--> Revised sentence

In this study, gene expression signatures from a public database, Gene Expression Omnibus (GEO), were analyzed. Total transcriptome was analyzed for differentially expressed genes (DEGs) between healthy control and sepsis groups, followed by gene set enrichment analysis for DEGs, which revealed up- or down-regulated pathways. We selected 998 immune-related genes included in immune response pathways using gene expression profiles to construct a network for the healthy control and sepsis groups...

Correction 2. Following sentence was revised.

For network construction, several appropriate R software packages were used^24^, including Bioconductor^25^, affy^26^, GEOquery^27^, lumi^28^, and limma^29^.

--> Revised sentence

For differentially expressed genes (DEG) analysis between control and patient groups based on total transcriptome, gene set enrichment for pathway analysis and network construction, several appropriate R software packages were used^24^, including Bioconductor^25^, affy^26^, GEOquery^27^, lumi^28^, and limma^29^

Correction 3. Following sentence was placed head of network analysis phrases in method section

**--> Gene set enrichment for pathway analysis**

Analysis of DEG was performed for total transcriptome . After a t-test using basic functions in R software, genes were selected based on false discovery rate of less than 0.01 (FDR < 0.01)^32^ except GSE54514, which was based on *P*-value of less than 0.05. Based on DEG analysis, gene set enrichment for pathway analysis using GAGE package, based on R language, was performed^33^. GAGE package applies input data to the Kyoto Encylopedia of Genes and Genomes (KEGG) pathways. Significant pathways were plotted for heatmap that was selected based on global *P*-value of less than 0.05.

Correction 4. Following subtitle was added.

Bioinformatics phrase in method section was divided into 2 parts, and following subtitle was added.

**--> Immune response related genes**

Correction 5. In results section, gene set enrichment analysis section was presented ahead of network analysis.

Correction 6. Table 3 was revised to Table 2 and vice versa.

Correction 7. In Discussion section, following paragraph was presented ahead.

--> The features of immune dysfunction or immune response in sepsis include defective antigen presentation and adaptive immunity (T and B cell abnormalities), abnormalities in NK cell activity, diminished immunoglobulin level, alteration in neutrophils, abnormal cytokine levels, unbalanced pro- and anti-inflammatory cytokines, complement consumption, and defective bacterial killing^4,5^. This complexity hampers understanding of immunologic features in sepsis. Immune suppression, immune paralysis and hypoinflammatory response are reported in sepsis that is intermingled with hyper inflammatory state^6-8^.

This study showed that in the adult sepsis group, there were more down-regulated pathways, whereas in the pediatric group, there were more up-regulated pathways. These data suggest that immune suppression might be more dominant in the adult sepsis group, whereas hyper-inflammatory states might be dominant in the pediatric sepsis group. The mechanism of immune response was indeed different between adult and pediatric patients in sepsis. However, down-regulation of TCR and APC pathway and up-regulation of complement-coagulation cascade pathway was found in most of adult and pediatric sepsis patients. These findings are congruent with previous reports that, in sepsis, CD4+ T cell showed cell exhaustion, increased apoptosis and decreased adhesion molecules, and CD28 and TCR diversity. In sepsis, CD8+ T cell also showed cell exhaustion, increased apoptosis, and decreased cytotoxic function, cytokine secretion, and TCR diverstiy^8^. Antigen presentation by follicular dendritic cell or dendritic cells is decreased in sepsis^8^. In the present study, isolation and prominent adaptive immune genes from the rest of the immune network were noted, and these data imply that part of the adaptive immune function was not in equilibrium or in a regulated state. As this isolated adaptive immune network was found in nonsurvivors, initial network state along with down-regulated pathways related with adaptive immunity resulted in unfavorable prognosis.

Complement and coagulation pathway was up-regulated in sepsis patients. However, network analysis showed that platelet and complement-and-coagulation-related genes were prominent and isolated in survivors, and that this initial state resulted in a favorable prognosis. On the contrary, it is reported that persistently activated complement system, including C3a and C5a, is found in sepsis, which is associated with unfavorable prognosis^6-8^. Excessive activation of complement system potentiated activated innate immune system in sepsis, and this also resulted in an unfavorable prognosis in the sepsis group^6^. These findings imply that initial inactivation of complement-coagulation pathway or persistent, uncontrolled activation of these pathways might be associated with unfavorable prognosis

Correction 8. Order of references, Supplementary Tables and Supplementary Figures were revised.

**Reviewer #2: Dear Associate Editor,
The manuscript 'Gene expression networks in sepsis: A network biology approach' by Kim et al analyzes various immune system pathways in patients with sepsis compared to healthy controls using network analysis approach and show that adaptive immune cells are isolated and prominent in that network in patients with sepsis. The strength of this manuscript is that they have used diverse datasets for analysis and obtained comparable results across all the datasets. The authors have addressed all the concerns raised earlier and they are satisfactory.**

--> Thank you for taking time reading, and correcting this article. The article was improved better than the previous ones. We unexpectedly found comparable results across the datasets. We also thank other researchers for sharing and uploading the precious data. We hope this article might help or support others to treat or elucidate sepsis.
