# Supplementary material for: Immune gene expression networks in sepsis: A network biology approach
Source: PLoS One. 2021 Mar 5;16(3):e0247669. doi: 10.1371/journal.pone.0247669 (PMC7935325; doi:10.1371/journal.pone.0247669)
Supplement: Attachment — Submitted filename: response.to.reviewers.commnet.R4.docx [file pone.0247669.s004.docx]

**Please use the space provided to explain your answers to the questions above. You may also include additional comments for the author, including concerns about dual publication, research ethics, or publication ethics. (Please upload your review as an attachment if it exceeds 20,000 characters)**

**Reviewer #3: Major comments:**

**1. The Results section subtitles are written in a method-centric manner. Changing these subtitles to informative insights resulting from the analysis would help bring out the message/insight that the authors are conveying. At this point, the manuscript reads like a report of a set of analyses run on the data. A similar undue emphasis on the step-by-step approach rather than results is reflected in the Abstract as well.**

--> Thank you for your insightful and accurate comments.

Result of gene set enrichment analysis and network analysis could be regarded as a different analysis method. We analyzed the data using different method and obtained results, respectively. Then, the result by each method was interpreted altogether. Finally and unexpectedly, we obtained some common factors from each result. Therefore, we tried to show that the results by different method had common factors. As description of result was corresponded to the method, description in manuscript might have read like an analysis run on the data.

We corrected the subtitle as follows.

Network topologic analysis was revised to "Network structure and immune function"

**1-1. A side effect of this problem are the many instances of redundancy in the Results section, where content similar to the Methods section was restated. For example, the "Network construction" section can be removed altogether. There are only a few non-redundant sentences toward the end of that section, which can be merged with the next section titled "Network topology analysis". Overall, the results section does not flow and the analyses do not flow well into biological insights.**

--> Thank you for your kind comment.

We deleted following paragraph in result section as the paragraph contains redundant contents.

--> Network Construction

Gene expression data from sepsis patients and normal controls were selected from a GEO database that was eligible for this study (Table 1, Methods section). Networks were constructed with immune-related genes from 21 pathways from the KEGG^30^ database, which are listed in S1–S2 Table. Genes were annotated with the most frequent function from the KEGG^30^, GO^31^ and Reactome^32^ databases, which included, in order of importance, adaptive immunity, innate immunity, NK cell activity, leukocyte migration, antigen presentation, complement-coagulation, platelet activity, cytokines-chemokines, signaling, and hematopoiesis (S3 Table).

**2. In the section titled "Network parameter analysis", the increase or decrease in the network topological parameters that were highlighted seemed minimal. For example, the sentence "Network heterogeneity was increased in 100% (3/3) of adult datasets, but were decreased in 100% (3/3) of pediatric datasets." --> the difference of Network heterogeneity for GSE57065 between the control (1.151) and sepsis group (1.172) is 1.8%.**

--> Thank you for your comments.

We admit that the numerical presentation of network parameters values seems to be minimal. We think that the tendency of network parameter provided more information compared to that of numerical value of parameters. Therefore, we reported the results whether the parameter was increased or decreased along with comparison of control and sepsis group. In addition, we thought that comparing the numerical values of parameters and using statistical method might not reflect exact information implied in the values of parameter due to the small datasets (3 adult and 3 children), which was available in public database.

As the description in the manuscript might exaggerate the result of parameters and might lead to misinterpretation of network parameters. Therefore, we deleted all the percentage (%) in the result section.

-->We deleted following sentences

Other parameters did not show significant differences between the healthy control group and the sepsis group.

**2-1. Additionally, for dataset GSE95233-1, non-survivors have decreased network heterogeneity compared to survivors. The reader might not be convinced that the minute differences in these topological parameters matter. Similarly, for the clustering coefficient the differences between control and sepsis groups were low. Reporting the differences in terms of percentages might help clarify the more important differences.**

--> Thank you for your comments.

As commented by reviewer, we added percentages that might clarify the result in regard to GSE95233-1.

--> Following sentence was added in result section

For GSE95233-1 analysis, difference between network parameters was calculated as follows: (case - control / control) x 100. The difference of clustering coefficient between survivor (0.390) and nonsurvivor (0.337) was -13.5%. The difference of modularity between survivor (0.581) and nonsurvivor (0.694) was 19.4%.

**3. Inconsistencies in the results text/table/abstract:
3(a) "Average degree and shortest path were increased in the sepsis group among 66% (2/3) of adult and pediatric datasets." This sentence from the Results section simply seems to be in contrast with the shortest paths reported in Table. In fact, shortest path decreased in 4 out of 6 datasets total.**
--> We are sorry for the careless errors.

We have corrected the sentence as follows.

--> Shortest path was decreased in 2/3 of adult and pediatric datasets, whereas average degree was increased in 2/3 of adult and pediatric datasets.

**3(b) This sentence in Abstract is inaccurate: The clustering coefficient and modularity parameters were increased in 5/6 datasets in the sepsis --> According to Table 3, the modularity coefficient is increased in 4/6 datasets. This is also mentioned in the Results section: "Modularity value was decreased in 66% (2/3) of adult datasets, whereas modularity value was increased in 100% (3/3) of pediatric datasets in the sepsis group." A similar mistake was made when referring to this in the Conclusions.**
--> We are sorry for the careless errors.

We have corrected the error in the abstract as follows

--> The clustering coefficient and modularity parameters were increased in 5/6 and 4/6 datasets in the sepsis group that seemed to be associated with increased functional aspect of the network.

We have corrected the error in Result section.

--> Modularity value was increased in 1/3 of adult datasets, whereas it was increased in 3/3 of pediatric datasets in the sepsis group.

**3(c) Also, was there a reason why only these two network topology parameters were highlighted (inaccurately for the clustering coefficient) in the abstract and conclusions?**
--> Thank you for insightful comment.

Among many network parameter, clustering coefficient are one of most studied parameters. In addition, from previous studies, we found out that clustering coefficient seemed to be associated with CRP in network analysis from hepatocellular carcinoma patients. These data seemed to imply that clustering coefficient was associated with increased inflammatory function of cytokine network (Jekarl DW et al, 2019;14(11):PLoS ONE e0224318). In this study, clustering coefficient was increased in 5/6 dataset of sepsis group which seemed to be associated with increased functional aspect of the network.

Modularity is a parameter that are associated with evolvability, multifunctionality and robustness of a network (Jekarl DW et al, 2019;14(11):PLos ONE e0224318). High modularity seemed to be associated with functional unit or function of a network. In this study, pediatric patient showed increased modularity, which implies that pediatric patient might have increased inflammatory function. These data are in line with gene set enrichment analysis that more of pathways are involved in pediatric patients compared adult.

In addition, these two parameters showed tendencies and clustering coefficient and modularity was increased in 5/6 and 4/6 datasets.

**4(d) Mistakes such as (a) and (b) plus the ones listed below among "minor typos and edits" are unacceptable, especially in the third revision. Other minor inconsistencies might exist that the reviewers might have not caught. The authors should take initiative in giving the manuscript a thorough last read for accuracy, consistency and correctness.**
--> Thank you for your precise comment. Although this manuscript was english language edited by specialist (Wiley Language Editing Serivce), authors are responsible for these errors. We are sorry for these error and we re-read the manuscript to find potential errors.

**Minor comments:
1. GSE95233 is repeated twice in the abstract, please remove the redundant one. There should be a total of 6 datasets studied (as listed in Table 1), not 7.**
--> Thank you for your kind comment

--> We deleted GSE95233.

**2. Where the randomized selection without replacement (to match the number of controls) for all datasets done such that the male-female percentage of control and sepsis groups were similar per dataset? Were they selected such that the mean age was comparable between the control and sepsis groups? If no, the conclusions from analysis that followed might fall apart. And if yes, please mention in the methods section that the control and sepsis groups were selected such that they were comparable with respect to age and male/female percentage. This can be added to the section in the methods where the "Cases in the cohort" were described and Table 1 was explained.**
--> Thank you for your kind comment.

As selection was peformed randomly, sex and age is not matched. The information of sex and age of the datasets in Table 1 are derived from published paper based on these datasets. As commented by other reviewers, we divided adult patient group from pediatric patient group.

--> Following sentences are added in the discussion section

As this study was a retrospective study using public datasets, sex and age was not matched for control and sepsis cases. The results might have been affected by these parameters.

**3. Table 1: For the sake of completeness, please add description for APACHEII and SOFA abbreviations in the Table 1 legend.**
--> Thank you for your kind comment

We have added followings in the Table 1 legend.

--> ED, emergency department; ICU, intensive care unit; NA, data not available; hr, hour; d, day; APACHEII, acute physiology and chronic health evaluation II; SOFA, sequential (sepsis related) organ failure assessment

**4. Methods: Gene set enrichment for pathway analysis: The authors say "After a t-test using basic functions in R software, genes were selected based on false discovery rate of less than 0.01 (FDR < 0.01) except GSE54514, which was based on P-value of less than 0.05." Why was a different metric used for this dataset? Changing the threshold for one dataset out of 6 seems arbitrary and should be justified.**

**-->**Thank you for your accurate comment.

We are not sure why GSE54514 showed smallest number of differentially expressed gene (2180 genes by P-value of less than 0.05) and after adapting FDR (P-0.01), the differentially expressed gene was 176 gene. It might be explained by smallest size of control (n=18) and patient group (n=18) compared to other datasets. Small size of data might have caused dispersion of the gene expression level, which might have led to insufficient number of genes for gene set enrichment analysis selected by FDR (*P*-0.01) values.

--> We re-analyzed the GSE54154 using same threshold and DEG was 176, which was written in the Table 2. For GAGE analysis, none of the pathway was adapted due to small size of the genes. We deleted supplementary Figure 2.

--> We changed the Table1 as follows.

**Table 2.** Differentially expressed gene analysis between control and sepsis followed by gene set pathway analysis

|  | GSE54514^a^ | GSE57065 | GSE95233 |  | GSE64456 | GSE66099 | GSE72829 |
| --- | --- | --- | --- | --- | --- | --- | --- |
| Patient type | Adult | Adult | Adult |  | Pediatric | Pediatric | Pediatric |
| Control cases (n) | 18 | 25 | 22 |  | 19 | 49 | 52 |
| Sequential selection  of sepsis cases (n) | 18 | 25 | 22 |  | 19 | 49 | 52 |
| Differentially expressed  Genes (n) | **176** | 7885 | 6784 |  | 3123 | 8229 | 7463 |
| Up regulated pathway (n) | **0** | 60 | 44 |  | 13 | 78 | 51 |
| Down regulated pathway (n) | **0** | 33 | 33 |  | 16 | 33 | 15 |
| Up regulated immune  pathway |  | C, Fc, RIG | C, Fc |  | C, Fc, NK, K, TLR | APC, C, CXC & etc, Fc, IgA, | BCR, C, Fc, CXC & etc, LK, NK, TLR |
| Down regulated immune  pathway |  | APC, TCR,  H, K | APC, BCR,  CXC & etc,  IgA, TCR |  | APC, IgA, TCR | H, TCR | APC, IgA, TCR |

^a^Differentially expressed gene was selected based on FDR (P<0.01).

APC, Antigen processing and presentation; BCR, B cell receptor signaling; C, Complement and coagulation; CXC & etc, chemokine signaling; Fc, Fc gamma R mediated phagocytosis or Fc epsilon RI signaling; H, hematopoietic cell lineage; IgA, intestinal immune network for IgA production; LM, leukocyte endothelial migration; NK, NK cell mediated cytotoxicity; RIG, RIG-I like receptor signaling; TLR, Toll like receptor signaling; TCR, T cell receptor signaling.

Following sentence was deleted.

--> except GSE54514, which was based on P value of less than 0.05

Following Supplementary Figure was deleted and following supplementary figure number was changes.

--> S2 Fig. The gene set analysis result of GSE54514. Red cells indicate up regulated gene sets, whereas green cells indicate down regulated gene sets.
